# Characterization of a new case of XMLV (Bxv1) contamination in the human cell line Hep2 (clone 2B)

**DOI:** 10.1038/s41598-020-73169-y

**Published:** 2020-09-29

**Authors:** Vincent Loiseau, Richard Cordaux, Isabelle Giraud, Agnès Beby-Defaux, Nicolas Lévêque, Clément Gilbert

**Affiliations:** 1 Université Paris-Saclay, CNRS, IRD, UMR Évolution, Génomes, Comportement et Écologie, 91198 Gif-sur-Yvette, France; 2 Université de Poitiers, CNRS UMR 7267 Laboratoire Ecologie et Biologie des Interactions, Equipe Ecologie Evolution Symbiose, 5 Rue Albert Turpain, TSA 51106, 86073 Poitiers Cedex 9, France; 3 Laboratoire de Virologie et de Mycobactériologie, CHU de Poitiers, Poitiers, France; 4 Unité de Microbiologie Moléculaire et Séquençage, CHU de Poitiers, Poitiers, France; 5 Laboratoire de Virologie et de Mycobactériologie, CHU de Poitiers, Poitiers, France; 6 EA4331-LITEC, Université de Poitiers, Poitiers, France

**Keywords:** Virology, Cellular microbiology, Bioinformatics, Sequencing

## Abstract

The use of misidentified cell lines contaminated by other cell lines and/or microorganisms has generated much confusion in the scientific literature. Detailed characterization of such contaminations is therefore crucial to avoid misinterpretation and ensure robustness and reproducibility of research. Here we use DNA-seq data produced in our lab to first confirm that the Hep2 (clone 2B) cell line (Sigma-Aldrich catalog number: 85011412-1VL) is indistinguishable from the HeLa cell line by mapping integrations of the human papillomavirus 18 (HPV18) at their expected loci on chromosome 8. We then show that the cell line is also contaminated by a xenotropic murine leukemia virus (XMLV) that is nearly identical to the mouse Bxv1 provirus and we characterize one Bxv1 provirus, located in the second intron of the pseudouridylate synthase 1 (*PUS1*) gene. Using an RNA-seq dataset, we confirm the high expression of the E6 and E7 HPV18 oncogenes, show that the entire Bxv1 genome is moderately expressed, and retrieve a Bxv1 splicing event favouring expression of the *env* gene. Hep2 (clone 2B) is the fourth human cell line so far known to be contaminated by the Bxv1 XMLV. This contamination has to be taken into account when using the cell line in future experiments.

## Introduction

Continuous cell lines are a cornerstone of cellular and molecular biology as well as of biomedical research. A long-known problem faced by researchers using such cell lines is contamination, whereby foreign cells or microorganisms are inadvertently introduced and remain unnoticed over multiple passages^1,2^. Characterizing cell culture contamination is of prime importance as contaminants may generate flawed experimental results and considerable confusion in the scientific literature^3,4^. The catalog of cell lines misidentified or cross-contaminated with another cell line contains 529 entries as of October 2019^5^. Among the 143 different contaminants listed, the most common is HeLa (118 entries), the first continuous human cell line ever established. HeLa derives from a cervical adenocarcinoma biopsy sampled in 1951 from Henrietta Lacks. Sequencing of the HeLa genome resolved a highly rearranged region on chromosome 8 containing multiple integrated partial genomes of the human papillomavirus 18 (HPV18), thought to have induced tumorigenesis^6^. In addition to standard short tandem repeat (STR) typing, known virus-HeLa cell junctions of HPV18 integrations have been used to unveil cases of cancer cell lines contamination by HeLa^7^.

Multiple microorganisms such as bacteria, fungi, viruses or yeasts have also been identified as persistent contaminants of widely-used cell lines^8–10^. Viral contaminations may be particularly problematic because viruses may present a risk for the persons handling cell cultures and unlike other organisms, viruses cannot be easily detected under light microscopy^9^. Screenings of hundreds of human cell lines have so far revealed only a relatively small proportion of them as contaminated with human viruses^11–13^. Most cases involve contamination by the Epstein Barr herpesvirus (EBV), either because primary cell cultures were established from infected patients or because EBV was intentionally used to generate transformed lymphoblastoid cell lines.

Non-human viruses have also been found in human cell lines, with murine leukemia viruses (MLV) being responsible for most cases of contaminations^11,13–19^. MLV are gammaretroviruses found under both exogenous and endogenous (proviral) forms in wild and laboratory mouse (*Mus musculus*) strains, which are associated with neoplasias of hematopoietic origin^20^. They are classified in several groups according to their host tropism, which is determined by interactions between their envelope-encoded receptor binding domain and host receptors^21^. While ecotropic MLV interact with the mCAT-1 receptor and can only infect mouse, polytropic MLV interact with the XPR1 receptor and can infect both mouse and other mammalian species. The xenotropic MLV (XMLV) also interact with the XPR1 receptor but they can only infect non-mouse species. Mouse strains are immunized against XMLV thanks to the presence of several *Xpr1* variants that restrict interaction with these viruses^22^. Most cases of human cell contaminations by MLV involve XMLV and it has been shown that contamination by XMLV often leads to important changes in cellular behavior, which can severely affect the conclusions of studies using such infected cell lines^15^.

Contamination by XMLV is known to occur either when a human tumor is xenografted in immunodeficient mice to establish or replicate human tumor cell lines, or through unintentional contact between contaminated and non-contaminated cell lines^23–25^. One of the best characterized cases of XMLV contamination is that of the so-called xenotropic murine leukemia virus-related virus (XMRV), which was originally thought to be a possible cause of prostate cancer and chronic fatigue syndrome^26^. XMRV was later shown to result from recombination between two distinct XMLV sequences integrated in the mouse genome (proviruses) and its presence in human cell lines was traced back to a contamination that occurred around 1993, when a prostate cancer cell line (CWR22Rv1) was xenotransplanted into immunocompromised mice^27^. Other well-characterized cases of XMLV contamination involve a virus originating from expression of a mouse chromosome 1 proviral locus called the B10 xenotropic virus 1 (Bxv1)^28^. This provirus is present in many severe combined immunodeficient (SCID) and nude mice strains^20^ and Bxv1 infection of human cell lines passaged onto these mice strains has been experimentally demonstrated^24^. So far, Bxv1 contamination has been detected in two prostate cancer cell lines, one B-cell line and two pancreatic β cell lines, in which it was shown to produce infectious viral particles^16,17,29^.

Here, we report a new case of a human cell line (Hep2[clone2B], Sigma-Aldrich catalog number: 85011412-1VL) contaminated by Bxv1 using high-throughput RNA- and DNA-seq. As part of a study aiming to characterize the potential presence of infectious agents in the cell lines of our laboratory, we set out to sequence total RNA and DNA extracted from Hep2 (clone 2B) cells. We characterized sequences from two viruses, the HPV18 papillomavirus and the Bxv1 XMLV. The presence of the former is consistent with the known contamination status of Hep2 (clone 2B) cell line by HeLa and the presence of the latter most likely results from mixing with a cell line or reagents contaminated with this virus.

## Materials and methods

### Cell cultures and DNA- and RNA-seq

Hep2 (clone 2B) cells were purchased from Sigma-Aldrich (catalog number: 85011412-1VL) on June 19, 2013 and cultured in Dulbecco's modified Eagle medium (DMEM; Invitrogen) supplemented with 10% fetal bovine serum and 1% penicillin–streptomycin (Pen-Strep; Life Technologies) at 37 °C in a 5% (vol/vol) CO_2_ atmosphere. Upon confluence, cells contained in a T75 flask were harvested in a 15 ml Falcon tube after trypsinization, washed in Earle's balanced salt solution (EBSS) and centrifuged at 1100*g* for 5 min. Total RNA and DNA were then extracted using the AllPrep DNA/RNA Mini Kit (Qiagen). A paired-end DNA library (mean insert size 191 bp) was constructed with the DNA sample and a stranded RNA library was constructed with the RNA sample after rRNA depletion. Both libraries were tagged and sequenced on an Illumina HiSeq2500 platform in a 2 × 125 bp configuration in High Output mode (V4 chemistry). Raw DNA- and RNA-seq fastq reads generated for this study have been deposited in dbGaP under accession number phs001944.v1.p1. All reads mapping onto the Bxv1 (JF908815) genome are provided in Supplementary Data 1 and 2.

### Bioinformatics analyses

Demultiplexing was performed by the sequencing company (Genewiz), yielding 140,313,660 reads with a mean quality score of 35.15 for the DNA sample and 57,593,594 reads with a mean quality score of 35.82 for the RNA sample. Remaining adapter sequences were removed and reads were trimmed with Trimmomatic using default parameters (“-phred33, Illuminaclip:adapter_file.fasta:2:30:10, minlen:126”, Bolger et al. 2014). To identify infectious agents potentially present in the cells, we mapped paired reads onto the human genome (UCSC genome data, human genome version hg19) using Bowtie2 in “sensitive-local” mode (Langmead and Salzberg 2012). Reads that did not map onto the human genome were assembled for each dataset. DNA-seq and RNA-seq reads were assembled with Masurca^30^ and Trinity^31^, respectively, using default settings. The resulting contigs were used as queries to perform blastn and blastx searches on the non-redundant (NR) database of GenBank. To assess sequencing depth of the two viruses identified in this study (Bxv1 XMLV and HPV18), the reads were mapped onto the genome of these viruses (accessions number: JF908815 for Bxv1 and GQ180792 for HPV18) with Bowtie2 in “sensitive-local” mode. To identify recombination breakpoints within each of the two genomes and between the two genomes and the Hep2 (clone 2B) genome we used all reads as queries to perform separate blastn searches (megablast option) on the human genome and on the Bxv1 and HPV18 genomes. Virus-virus and cell-virus junctions were then searched within reads using the pipeline described in Ref.^32^. Briefly, only reads aligning over at least sixteen bp on a genome region only and over at least sixteen bp on another genome region only were retained. Reads had to align on at least 100 bp of their length. The overlap between alignment on the virus and on the host sequences was set to involve at most 20 bp and at least -5 bp (see Supplementary Fig. S6 in Gilbert et al.^32^).

### Checking for contamination by rodent DNA

To assess the presence of rodent DNA or RNA in our samples, reads were mapped using Bowtie2 in “end-to-end” mode on the latest genome version of *Mus musculus* (GRCm38/mm10) and *Rattus norvegicus* (GRSC 6.0/rn6). Using the SAMtools depth program, the mean rodent genome coverage as well as the percentage of the genome covered by the reads were determined. The nature of regions covered by more than 8000 reads was checked in the UCSC genome browser.

### PCR verifications

To rule out the possibility that the Bxv1 contamination occurred in our laboratory, we purchased a second batch of Hep2 (clone 2B) cells from Sigma-Aldrich in May 2018 and searched by PCR for the presence of Bxv1 in this new batch and in the Hep2 (clone 2B) cells we used for the sequencing (batch ordered at Sigma-Aldrich in 2014). Amplification reactions were performed from 5 ng of DNA extracted from Hep2 (clone 2B) cells by using 10 µmol L^−1^ of each primer (Bxv1_1-F: AAGAGAAAGAGAGGGACCGC; Bxv1_1-R: TTTCCTCCAGTAGCCCCTTG), 3 mM MgCl2, 0.2 mM dNTP and 0.75 unit of DreamTaq DNA polymerase (Thermo Fischer Scientific) under a 35-cycle PCR program (95 °C for 4 min; 35 cycles of 95 °C for 30 s, 56 °C for 30 s, 72 °C for 15 s, and 72 °C for 10 min). Then a migration of the PCR products was performed on a 1.5% agarose gel at 100 V during 25 min and bands were visualized with a Bio-Rad Transilluminator Universal Hood II under UV light. To check whether regions of the Bxv1 genome not covered by our DNA-seq dataset were in fact present in Hep2 (clone 2B) cells, we PCR-amplified and Sanger-sequenced three such regions as well as another region covered by our DNA-seq dataset using the following primer pairs: Bxv1_2-F: CCCCAGAAGAGAGAGAAGAAC; Bxv1_2-R: CATTGGTCCTTATCGAGTTGG; Bxv1_3-F: TGCCTTTGAGTGGAGAGATC; Bxv1_3-R: CTAGGGTTTGTAGAAGGGCC; Bxv1_4-F: CCTTCTCAACAACCTGGGAC; Bxv1_4-R: ACAGGGTCAGCTTGTGTTG; Bxv1_5-F: CAGGCAAGCTAACTATGGGA; Bxv1_5-R: CCCAGATTACCTCGGTTTCA.

## Results and discussion

### Confirmation of Hep2 (clone 2B) contamination by HeLa based on HeLa-HPV18 characteristics

**Figure 1:**
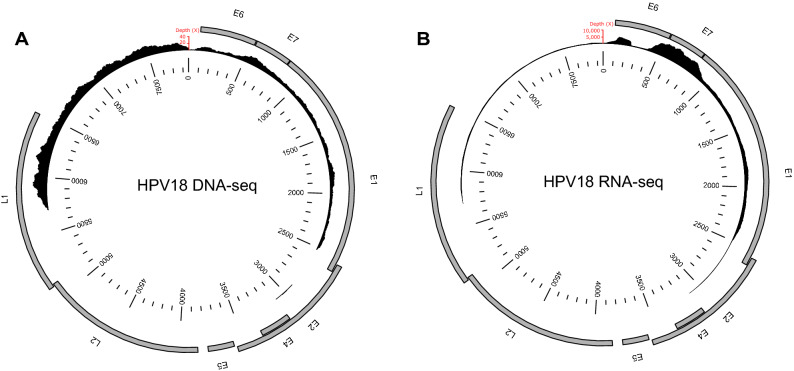
HPV18 sequencing depth by DNA-seq (**A**) and RNA-seq (**B**) reads. The coverage patterns indicate that a portion of the E2 and L1 genes as well as the entire E4, E5 and L2 genes are missing and that the E6 and E7 oncogenes are highly expressed.

As mentioned on the Sigma Aldrich website, the Hep2 (clone 2B) cell line (catalog number: 85011412-1VL) was originally derived “from tumours produced in irradiated-cortisonised weanling rats after injecting with epidermoid carcinoma tissue from the larynx of a 56-year-old male, but it was later found to be indistinguishable from HeLa by STR PCR DNA profiling.” It is a typical case of non-existent cell line that may cause important problems in cancer research^33^. Consistent with HeLa contamination, assembling of RNA- and DNA-seq reads obtained from sequencing this cell line and not mapping on the hg19 human genome yielded various contigs that were almost identical to the HPV18 (GenBank accession number: NC_001357.1). As previously reported^7^ for HPV18 sequences integrated into the HeLa genome, mapping of both RNA- and DNA-seq reads onto the HPV18 genome shows that a portion of the E2 and L1 genes as well as the entire E4, E5 and L2 genes are missing (Fig. 1). Moreover, the mapped regions displayed the same 23 SNPs between the reads and the HPV18 genome than those identified between HeLa-HPV18 and HPV18 (Cantalupo et al.^7^) (Supplementary Table 1). Mean DNA-seq depth varies from 1.3× for the partial E2 gene up to 26.4× for the L1 gene (Fig. 1A), reflecting the complex and partially duplicated structure of the integrants^6^. Mean RNA-seq depth varies from 137X for the partial E2 gene up to 4078 and 8093× for the E6 and E7 genes, respectively. This is in agreement with the known high expression of the latter two genes, which are involved in oncogenesis through neutralization of tumour suppressors^6,34,35^. Finally, our search for integration loci in DNA-seq reads revealed four virus-cell junctions supported by one or more reads and also supported by RNA-seq reads (Table 1; Supplementary Table 2). All four junctions fall within the chromosome 8 region where HPV18 is known to be integrated in HeLa cells (8q24.21; between positions 128,228,000 and 128,243,000)^6,7^. Much like in Cantalupo et al.^7^, we also identified a number of other junctions in RNA-seq reads, among which all those supported by more than one reads fall within the 8q24.21 region (Table 1; Supplementary Table 2). As noticed by Cantalupo et al.^7^, many of the virus-cell RNA junctions involve the 929 5′ splice site in E1 of HPV18, likely indicating that after read-through transcription, splicing events fused the 929 5′ donor to a downstream acceptor site located in the human genome^7,36^. Altogether, these results confirm that Hep2 (clone 2B) was contaminated by HeLa at some point during its propagation and they show that HPV18 sequences integrated in this subclone of HeLa have the same characteristics as in other subclones in terms of position in the genome and expression pattern^6,7,34^.

**Table 1:** Characteristics of HeLa–HPV18 junctions supported by both DNA- and RNA-seq reads.

Number of DNA-seq reads supporting the junction	Number of RNA-seq reads supporting the junction	Viral breakpoint position	Viral gene	Position of breakpoint in human genome	Human chr. Band
1^a^	272	929	E1	128241377	8q24.21
25	97	5735	L1	128230628	8q24.21
1	57	2497	E1	128241551	8q24.21
1	15	930	E1	128231213	8q24.21

^a^Several steps involved in the construction of an Illumina library (including cDNA library synthesis and illumina PCR) may generate artificial chimeras^46,47^. Thus, relying only on one read to identify a breakpoint is not good practice. However, this Table only reports HPV18-HeLa breakpoints that are supported by reads generated in two independent sequencing experiments (RNA-seq and DNA-seq), including one in which they are supported by multiple reads (here more than 15). For example, the first lane of the Table describes a breakpoint between position 929 of the HPV18 genome and position 128 241 377 of human chromosome 8 that is retrieved in 1 DNA-seq read and in 272 RNA-seq reads independently. The position of all breakpoints reported here is consistent with those reported in earlier studies (see text for details).

### Hep2 (clone 2B) (HeLa) cells are contaminated by Bxv1 XMLV sequences

In addition to HPV18 integrants, our assembly of non-human reads also yielded contigs 100% identical to Bxv1, an XMLV proviral locus known to generate infectious particles able to infect human cells and so far identified as a contaminant in four human cell lines^17,20,29^. As several studies have shown that the presence of XMLV in human cell lines could be due to contamination by mouse DNA^19,37^, we checked for such contamination by mapping both DNA-seq and RNA-seq reads onto the mouse genome. Given that the original Hep2 cells were obtained by passaging larynx carcinoma cells onto immune-compromised laboratory rats^38,39^, we also monitored the possible presence of rat DNA by mapping all reads onto the rat genome. Only ≃ 4% of the reads mapped onto the rodent genomes, with only ≃ 1% of the genome covered and a mean sequencing depth of 0.26× for the two species. Importantly, mapped regions corresponded exclusively to RNA genes (7SK, 7SL, U1, U2) that are known to be highly similar between mammalian species and no read was found to map onto intracisternal-A particle elements, which are rodent-specific transposable elements typically used as markers of contamination^19,37^. Thus, the presence of Bxv1 is not due to contamination by rodent DNA but rather results from mixing with a contaminated cell line or reagent. To further verify that contamination did not occur in our laboratory subsequent to reception of Hep2 (clone 2B) cells on June 19 2013 we purchased a second batch from Sigma-Aldrich on May 11, 2018 and validated the presence of Bxv1 sequences by PCR (Supplementary Fig. 1). We conclude that Hep2 (clone 2B) cells from Sigma-Aldrich (catalog number: 85011412-1VL) are not only undistinguishable from HeLa but they are also contaminated by Bxv1 XMLV sequences.

The finding of Bxv1 in the HeLa-contaminated Hep2 (clone 2B) cell line begs the question of whether the virus is present in HeLa cells. XMLVs have been searched in a large number (> 600) of human cell lines, including HeLa and nine of its derivative subclones in which it was found to be absent^18,19,29^. In agreement with this, our blastn search of the Bxv1 sequence onto two HeLa short read datasets available in the SRA database of NCBI (accession numbers: SRX699196 and ERX3188263) revealed no significant hit. This indicates that Bxv1 is likely absent from all or most HeLa cell lines. However, we cannot exclude that this virus was present in the very HeLa cells that contaminated Hep2 (clone 2B). Thus, whether Bxv1 was introduced into Hep2 (clone 2B) by HeLa cells, or whether it was introduced into Hep2 (clone 2B) before or after this cell line was contaminated by HeLa, remains an open question.

### Characterization and expression of a Bxv1 provirus

To further validate the presence of Bxv1 in the HeLa-contaminated Hep2 (clone 2B) cell line, we searched for evidence of integration of the retrovirus. We identified two virus-cell junctions covered by more than one DNA-seq read and/or also supported by an RNA-seq read (Supplementary Data 3). Interestingly, the two junctions involve the very first and very last position of the long terminal repeat of Bxv1 and the same position in the human genome, suggesting that they correspond to the 5′ and 3′ extremities of one same proviral locus. Alignment of the junctions with the corresponding region of the human genome revealed that Bxv1 generated a 5-bp target site duplication (AAACC) upon integration (Supplementary Data 3). Much like Bxv1 proviruses from two prostate cancer cell lines (LAPC4 and VCaP) which all integrated into introns^17^, the Bxv1 provirus characterized here lies within the second intron of the pseudouridylate synthase 1 (*PUS1*) gene. Furthermore, in agreement with the known propensity of MLV to preferentially target transcription start sites (TSS) and CpG islands^40,41^, the Hep2 (clone 2B) Bxv1 lies only 946 bp downstream of the nearest *PUS1* TSS, well within a 1463-bp long CpG island. Altogether, these results indicate that the Bxv1 integration identified in Hep2 (clone 2B) cells is a *bona fide* proviral locus, confirming contamination by this virus of the Hep2 (clone 2B) cell line.

**Figure 2:**
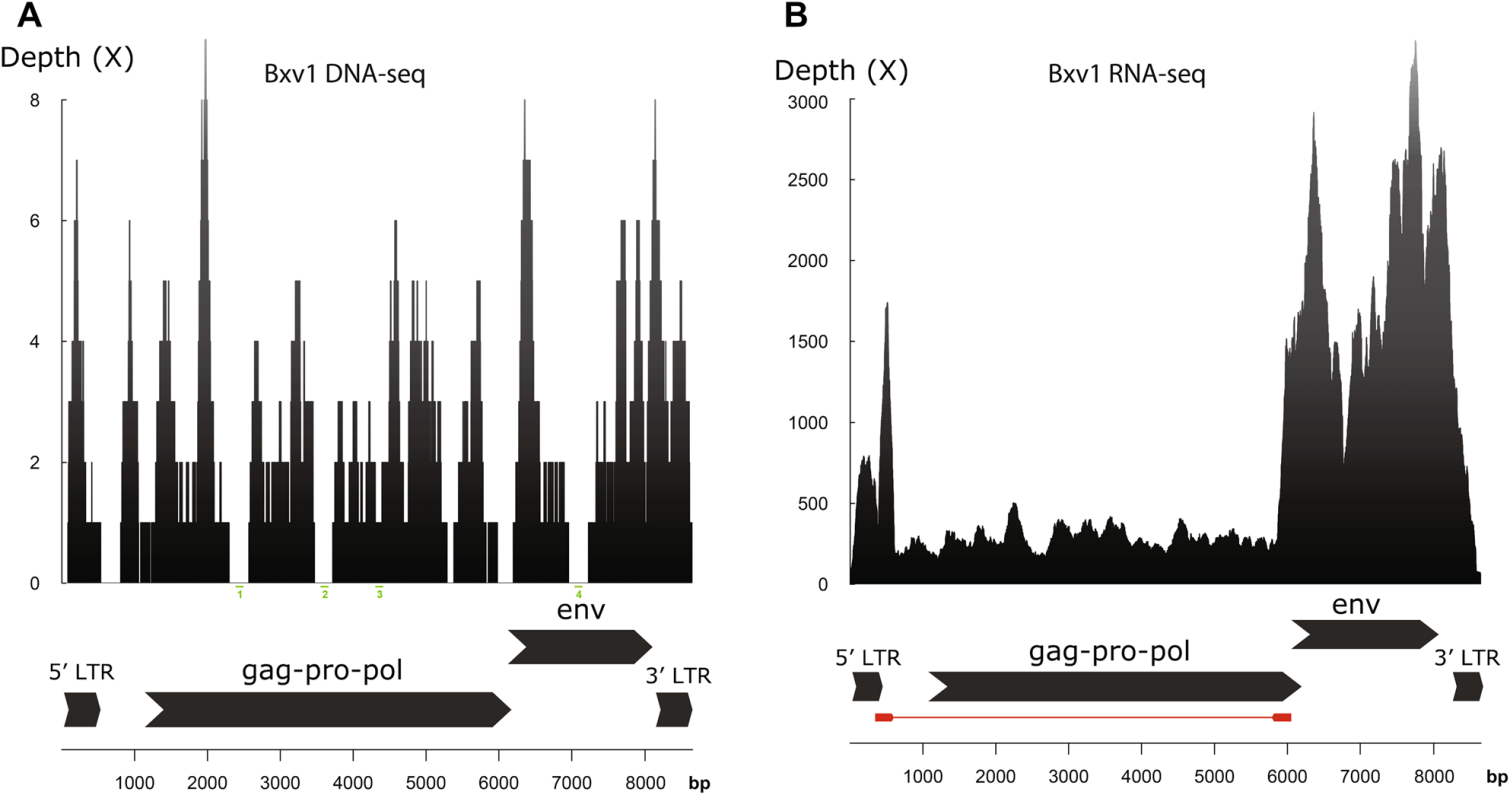
Bxv1 sequencing depth by DNA-seq (**A**) and RNA-seq (**B**) reads. The coverage patterns indicate that the entire Bxv1 genome is present in Hep2 (clone 2B) cells and that the *env* ORF is more expressed than the gag-pro-pol ORF. The green lines in (**A**) show the position of viral regions that were Sanger-sequenced. Numbers below the green lines correspond to those used to name Sanger-sequencing reads in Supplementary Data 4. The red line in (**B**) represents the spliced region between positions 587 and 5887.

To assess how many proviral loci may segregate in the cell line, we mapped all reads on the Bxv1 genome (accession number: JF908815). While several short segments amounting to 15% of the Bxv1 genome are not covered by DNA-seq reads (Fig. 2A), we believe this is due to stochastic under-representation rather than true absence of some regions because Bxv1 is fully covered by RNA-seq reads (Fig. 2B). In agreement with this, we were able to PCR-amplify and Sanger-sequence three regions not covered by DNA-seq reads (Fig. 2A, Supplementary Data 4). Thus we believe that Hep2 (clone 2B) cells contain at least one full-length Bxv1 provirus. Mean DNA-seq depth of Bxv1 is 2.2×, which is lower than that calculated over the entire human genome (6.4×). It is thus possible that the copy that we were able to map is the only one segregating in the cell line. However, we cannot exclude that multiple proviruses are present, which altogether amount to one or less than one provirus per cell (i.e. some cells may be free of integrated Bxv1). The low number of Bxv1 proviruses may be due to the low capacity of the virus to replicate in these cells. Yet, the entire Bxv1 genome is expressed in Hep2 (clone 2B) cells, including the long terminal repeats which are necessary for the virus to replicate and integrate into the host genome, with mean RNA-seq depths varying from 365× for the *gag-pro-pol* open reading frame (ORF) to 1955× for the *env* ORF (Fig. 2B). However, the level of Bxv1 expression is relatively low, with only 26,985 (or 0.1% of) RNA-seq reads mapping to the virus, which only ranks 21,906th when listing all human transcripts by decreasing number of mapped RNA-seq reads (Supplementary Table 3). This is much lower than the level of expression of Bxv1 measured in the JY B cell line, in which proviral transcripts ranked first in terms of number of aligned reads, being mapped by almost 1,000,000 reads representing 2.6% of all RNA-seq reads^16^.

In the Lin et al.^16^ study, 768 RNA-seq reads were found to contain a junction between positions 587 and 5887 of Bxv1 (accession number: JF908815), which corresponds to a splicing event leading to the expression of the *env* ORF. Our search for virus–virus junctions unveiled a nearly identical splicing event, with 757 reads (145 reads when potential PCR duplicates are removed), supporting a junction between positions 587 and 5884 (Fig. 2). The position of the splice donor site matches exactly between our study, that of Lin et al.^16^ and the annotation of the N417 MLV (accession number: HQ246218) which is 99.98% identical to Bxv1 JF908815. However, the position of the splice acceptor site differs (5884 in Lin et al.; 5887 here; 5219 in HQ246218 corresponding to 5601 in JF908815), which may be due to context-dependent variations in splicing. To assess whether the presence of Bxv1 in Hep2 (clone 2B) cells could result from mixing with the JY B cell line, we checked for the presence of RNA-seq and/or DNA-seq reads mapping onto the EBV genome, which is known to be present in JY B cells^16^. We did not find any read mapping on the EBV genome. While this suggests that the Bxv1 contamination is not due to a mix with JY B cells, we cannot exclude that a transient mix occurred between Hep2 (clone 2B) and JY B cells at some point but that JY B cells are no longer detectable. Interestingly, the RNA-seq coverage of the region corresponding to the alternative *env* transcript is three to five times higher than that of the rest of the Bxv1 sequence (Fig. 2B) showing that for some reason, the splicing generating this transcript is markedly favoured in Hep2 (clone 2B) cells. Rather than alternative splicing, the junction we found in RNA-seq reads between positions 587 and 5887 of Bxv1 could be due to transcription of a deleted Bxv1 copy, that would segregate in some cells in addition to a full length copy. To check for the presence of a deleted Bxv1 copy we designed PCR primers on both sides of the deletion. All PCRs performed using those primers were negative, suggesting that the truncated transcripts unlikely result from transcription of a deleted Bxv1 proviral copy. We have not tested whether the higher expression of the *env* ORF translates into an accumulation of the ENV protein, which has been linked to the generation of cytopathic effects in some MLV^42^. That said, we have not observed any cytopathic effect, in agreement with the fact that most MLV do not generate such effects^43^.

In their study of the JY B cell line contaminated by Bxv1, Lin et al.^16^ also found evidence of G-to-A editing likely resulting from the activity of the APOBEC3G restriction factor, which induces deamination of cytidine to uridine in single stranded DNA viral intermediates^44^. Specifically, Lin et al.^16^ found that 44 out of the 45 SNPs identified in their RNA-seq reads were G-to-A changes, indicating that they likely resulted from transcription of APOBEC3G-edited viral genomes. Here, in agreement with the known absence of APOBEC3G in HeLa cells^45^, the APOBEC3G transcript ranks only 37,052th in the list of human transcripts ordered by the number of mapped reads (Supplementary Table 3). The number of SNPs supported by more than 10 reads and having a frequency > 2% in our RNA-seq data is anyway too low to draw any conclusion on the possible activity of APOBEC3G in Hep2 (clone 2B) cells, but it is worth noting that four of the five SNPs we identified using those criteria in our RNA-seq data are G-to-A changes.

## Conclusion

In this study, we have confirmed that the Hep2 (clone 2B) cell line (Sigma-Aldrich catalog number: 85011412-1VL) is contaminated by HeLa cells based on the characterization of HPV18 integrants and expression patterns. We have further demonstrated that the cell line is also contaminated by a virus nearly identical to the Bxv1 XMLV provirus, the presence of which is likely due to direct contact between the cell line and another contaminated cell line or reagent. Based on sequencing depth, we show that the cell line might contain only one Bxv1 provirus, which we were able to map to the second intron of the *PUS1* gene. While our study does not demonstrate that Bxv1 is able to replicate in Hep2 (clone 2B) cells, it shows that it is expressed at a moderately high level, which may impact various cellular pathways. Thus, the presence of this virus in otherwise HeLa-contaminated Hep2 (clone 2B) cells will have to be taken into consideration in future studies using this cell line to avoid erroneous interpretations of experimental results. Furthermore, this study should also encourage others using this cell line and related ones, such as the (code A) 86030501-1VL, or 85020207-1VL Hep-2C (HeLa derivative) Human Negroid cervix carcinoma (code C), to check for the presence of Bxv1 using the PCR primers we provide in the materials and methods section.

## Supplementary information

Supplementary Information 1.

Supplementary Information 2.

Supplementary Information 3.

Supplementary Information 4.

Supplementary Information 5.

Supplementary Legends.

## Data Availability

In addition to the Supplementary Fig. 1 and Supplementary Data 1–4 included in this published article, the raw fastq reads generated during the current study are available in the dbGaP repository under accession number phs001944.v1.p1.
