# Supplementary material for: Characterization of a new case of XMLV (Bxv1) contamination in the human cell line Hep2 (clone 2B)
Source: Sci Rep. 2020 Sep 29;10:16046. doi: 10.1038/s41598-020-73169-y (PMC7524804; doi:10.1038/s41598-020-73169-y)
Supplement: Supplementary file 1 — Supplementary Information 1. [file 41598_2020_73169_MOESM1_ESM.docx]

@GWZHISEQ02:315:C9E6MANXX:5:1108:9651:16292

CGGACCCGCCGCTACCTGGGCGACCGCTCCTGCACCTAAACCTGAAAGACCCCACCATAAGGCTTAGCAAGCTAGCTGCAGTAACGCCATTTTGCAAGGCATGAAAAAGTACCAGAGCTGAGTTCT

+

BBBBBFFB<F7FFFFFBF<F<BFFFFFFFFFFFFFFFFFFFFFFFFFFFFFFFFFFFFFFFFFFFFFFFFFFFFFFFFFFFFFFFFFFFFFFFFFFFFFFFFFFFFFFFFFFFFFFFFFFFFFFF/

@GWZHISEQ02:315:C9E6MANXX:5:2305:13740:62280

GCCGGACCCGCCGCTACCTGGGCGACCGCTCCTGCACCTAAACCTGAAAGACCCCACCATAAGGCTTAGCAAGCTAGCTGCAGTAACGCCATTTTGCAAGGCATGAAAAAGTACCAGAGCTGAGTT

+

BBBBBFFFFFFFFFFFFFFFFFFFFFFFFFFFFFFFFFFFFFFFFFFFFFFFFFFFFFFFFFFFFFFFFFFFFFFFFFFFFFFFFFFFFFFFFFFFFFFFFFFFFFFFFFFFFFFFFFFFFFFFFF

@GWZHISEQ02:315:C9E6MANXX:5:2203:15479:47959

AAAAGTCACAAGGAAGTTTAGTTAAAGAATAAGGCTGAACAAAACTGGGACAGGGGCCAAACAGGATATCTGTGGTCGAGCACCTGGGCCCCGGCTCAGGGCCAAGAACAGATGGTACTCAGAAAA

+

</<//<//BF/</FBF<F//<<F<<FF//F</F</</<<//<FFF///F//</B//</FFF/F///FF//FFBB/FB//<//</<////<B////BBB/7/7//B/<B/F/F/FFBF<//<FB/7B

@GWZHISEQ02:315:C9E6MANXX:5:2203:15479:47959

GCTAGTTTCGCTTTATCTGAGTACCATCTGTTCTTGGCCCTGAGCCGGGGCCCAGGTGCTCGACCACAGATATCCTGTTTGGCCCCTGTCCCAGTTTTGTTCAGCCTTATTCTTTAACTAAACTTC

+

<BBB/<FF<F<FB<F//FF//<F<//FB<B<<<//<//<FFBB<BF///7/F7/BFFF<F/B/////<F//////<<FBF/////<F/</<B//</<F<BF/B7B//7/<<</<FBFFF///////

@GWZHISEQ02:315:C9E6MANXX:5:2305:13740:62280

TTTTGGGGAACTTGAAACTGAGGTGGGACTTTCCAGAAACTGTTGCTAGTTTCGCTTTATCTGAGTACCATCTGTTCTTGGCCCTGAGCCGGGGCCCAGGTGCTCGACCACAGATATCCTGTTTGG

+

BBBBBFFFFFFFFFFFFFFFFFFFFFFFFFFFFFFFFFFFFFFFFFFFFFFFFFFFFFFFFFFFFFFFFFFFFFFFFFFFFFFFFFFFFFFFFFFFFFFFFFFFFFFFFFFFFFFFFFFFFFFFFF

@GWZHISEQ02:315:C9E6MANXX:5:2113:16057:77737

CCGGCTCAGGGCCAAGAACAGATGGTACTCAGATAAAGCGAAACTAGCAACAGTTTCTGGAAAGTCCCACCTCAGTTTCAAGTTCCCCAAAAGACCGGGAAAAACCCCAAGCCTTATTTAAACTAA

+

/</BBB<</B<BFFFF/BFFF/<FFB/F/<FF/<FFFFFFFFBFBBFBFFFFF/B/BBF/FFFF<<F/FFFFFFF//<<FF<F/<//</<F/FFFF//<<BFFB/F/7///BF//FFF/BFFFF/F

@GWZHISEQ02:315:C9E6MANXX:5:1108:9651:16292

ATAGGGCTGGGGAGCAAAAAGCGCGGTTACAGAAGCGAGAAGCGAGCTGATTGGTTAGTTTAAATAAGGCTTGGGGTTTTTCCCGGTCTTTTGGGGAACTTGAAACTGAGGTGGGACTTTCCAGAA

+

BBBBBFFFFFFFFFFFFFFFFFFFFFBFFFFFFFFFFFFFFFFFFFFFFFFFFFFFFFFFFFFFFFFFFFFFFFFF<BBFFFFFFFFFFFFFFFFFBFFFFFFFFFFFFFFFFFFFFFFFFFFFFF

@GWZHISEQ02:315:C9E6MANXX:5:1201:19869:52329

TTTCAAGTTCCCCAAAAGACCGGGAAAAACCCCAAGCCTTATTTAAACTAACCAATCAGCTCGCTTCTCGCTTCTGTAACCGCGCTTTTTGCTCCCCAGCCCTATAAAAAGGGTAAAAACCCCACA

+

BBBBBFFB<FB//FFFFFFBFB7<FFFFFFFFFFFFFFBFFFFFFFFBFF<FFFFBFFFFFFFFFFFFF<FF/FBBFFFFFFFFFBFFFFFFFBFFFFFF7B/BBFFFFFFBB/FFFFF</BBF<7

@GWZHISEQ02:315:C9E6MANXX:5:1201:19869:52329

GCGCGCCGAGTGTGGGGTTTTTACCCTTTTTATAGGGCTGGGGAGCAAAAAGCGCGGGTACAGAAGCGAGAAGCGAGCTGATTGGTTAGTTTAAATAAGGCTTGGGGTTTTTCCCGGTCTTTTGGG

+

BBBBB/BBFFB</FFFFF//BFF<FBFFFFF/BFF/B/F<////<///</<7//7///<</<FF/F/B//7<7//<7BFF/<7F//7//<BF/////7BBBFBF<F///B/7/////7/77B/7/7

@GWZHISEQ02:315:C9E6MANXX:5:2113:16057:77737

CCGGGCGACTCAGTCTATAGGATGACTGGCGCGCCGAGTGTGGGGTTTTTACCCTTTTTATAGGGCTGGGGAGCAAAAAGCGCGGTTACAGAAGCGAGAAGCGAGCTGATTGGTTAGTTTTAATAA

+

BBBB/////</B/F//////</<</<//F<////</7/<F</7/<F/F/<F<<BF//////<7///7//////<//<FF/F////<B/B///<BF/FF//7F//7BFF//7//7<F</77//77B7

@GWZHISEQ02:315:C9E6MANXX:5:2314:5533:12289

AGGGTAAAAACCCCACACTCGGCGCGCCAGTCATCCGATAGACTGAGTCGCCCGGGTACCCGTGTTCCCAATAAAGCCTTTTGCTGTTTGCATCCGAAACGTGGCCTCGCTGTTCCTTGGGAGGGT

+

B<BBBFF<FFFFF//BBF/FF<FFFFBBFBFFFFFFFFFF<<FFFFFFFFFF/FBF7FFBFFFBF<BFFFB//BFB/FBFFFFFFFFF</BBBFFFFFBFF/BBFF<BFFF//7//7BBFFFFFFB

@GWZHISEQ02:315:C9E6MANXX:5:1106:17258:56835

GATCGGAAGAGCACACGTCTGAACTCCAGTCACGAGTGGATATCTCGTATGCCGTCTTCTGCTTGAAAAAAAAAAGGCCACGTTTCGGATGCAAACAGCAAAAGGCTTTATTGGCAACACGGGTAC

+

BBBBBFF/FB<FFFFFFFFFFFFFFFFFBFFBFF</<F/<<FFB/BBF<BBFFB<B</<FBF<<<<<FFFFF/<F///7<F/<F/FFFFF<B//FFB7BB<FFF/////BFFF</77F/7///BBF

@GWZHISEQ02:315:C9E6MANXX:5:1111:17027:25939

GGATATACTTATTCTCCTGACTAACAATCTTCATAATCACGCCATTTGCTGTTTGCATCCCAAACGTGGCCTCGCTGTTCCTTGGGAGGGTCTCCTCAGAGTGATTGACTACCCAGCTCGGGGGTT

+

//</</////B/<////////<</<<///<FFF//<</<///<///<<///BFF<B<</F//BFFBB<F<FFF////7B<FB<//F/F</<<</BF/BFBF/77FFFBB<B<//<<B/FFF</<F/

@GWZHISEQ02:315:C9E6MANXX:5:1106:17258:56835

GATCGGCAGAGCGTCGTGTAGGGACAGAGTGTAGATCTTGGTTGTTTGCGTACCATTCAAAAAAAAACTGTTCCTTGGGAGGGTCTCCTCAGAGTGATTGACTACCCAGCTCGGGGGTCTTTCATT

+

////<//<//</<///</</<FF////</<//<B<//<//<//<<///</F///</<//<///////</<///<//<<<F<BB/<//B//77<7FB<//B//BFB/</B///77<F777//77//7

@GWZHISEQ02:315:C9E6MANXX:5:2314:5533:12289

GCCCGGGAAGCGGCAGGTCCCGCGGGGTTTAGGTGCAGGTGCGGCCGCCGGGGAAGCGGTGGGTCCGGCTGGGTTTGAAAGACCCCCGAGCTGGGTAGTCAATCACTCTGAGGAGACCCTCCCAAG

+

//</<B<F<F//FF<B<F<B/F<BF/B</B<BFFFFF<F<FBF</BF<7<7<BB/BFB/<<BBBFFFF/<B///B/<</BF<FFF<FB//</<///7B7/7BFFFBB7BF/7/7B7/7//7BBFFB

@GWZHISEQ02:315:C9E6MANXX:5:1111:17027:25939

CCAGCGGCTTCCAGACCTCTCGTTGTACTGCTTGGAGGGCCTGTAAGTGAGCTCCCGACGGTGGGTCGGTGGTCCCTGGGCGGGGGTCTCCAAATCCCGGATGAGCCCCCAAATCTAAGTCCCCCG

+

BBBBBFFFFFFFFFFFFFFFFFFFFFFFFFBFFFFFFFFFFFFFBFFFFFFFFFFFBFFFFBFF<FFFFFFFB/7BFFFFFFFF<F<FF<7B/BB///7///<</<B/F/B<////7B7//7////

@GWZHISEQ02:315:C9E6MANXX:5:1313:2152:21250

CCGAGTCCTGACCGATTCGGACTATTTGGAGCCCCTCCTTTGTCGGAGGGGTACGTGGTTCTTTTAGGAGACGAGAGGTCCAAGCCCTCGCCGCCTCCATCTGAATTTTTGCTTTCGGTTTTTCGC

+

BBBB<FFBFF/<FFBBBFFFFF<<BBBFFFFFFFB<<F//<BFFB/FBFFF<F<</<B</B<FF/FBB/<///F/BF</77FFBB<//7F<BBBF/7BFFFB<BFFFFFF/////////777//77

@GWZHISEQ02:315:C9E6MANXX:5:2201:5223:57752

CGGACTTTTTGGAGCCCCTCCTTTGTCGGAGGGGTACGTGGTTCTTTTAGGAGACGAGAGGTCCAAGCCCTCGCCGCCTCCATCTGAATTTTTGCTTTCGGTTTTTCGCCGAAACCGCGCCGCGCG

+

BB///</B</FFBF<///<B/<//BF<7//<7<<FBFFFFF/</</BB<B/FFFFF/FBBFBF</B<F<F/FB<FBFF<<B//7F<//7/</FFF<7/<B/7//B/7</BF<FB/7/FFF/7B//7

@GWZHISEQ02:315:C9E6MANXX:5:2201:5223:57752

CTGAGACAGACAAGACGCGCGGCGCGGTTTCGGCGAAAAACCGAAAGCAAAAATTCAGATGGAGGCGGCGAGGGCTTGGACCGCTCGTCTCCTAAAAGAACCACGTACACCTCCGTCAAAGGTGGG

+

/</BBBFFFF<F<BF</FB<</F<FF<FBFFFFFF/FBFBFB<<F//7FBF/B<B7BFFF/B//B7FFFF/7//77//</BF/7/7/B<//</B////7<//F/B//7/77/<//////7///7//

@GWZHISEQ02:315:C9E6MANXX:5:1313:2152:21250

GGTGGTTACGGTCTGTCCCATATTTTTAGAAACGGTCCAAAACAATAACGAACAGACAAATGACAAAACAACACTGAGACAGACAAGACGCGCGGCGCGGTTTCGGCGAAAAACCGAAAGCAAAAA

+

BBBBBFFFFFFFFBFFFFFFFFF//FFFFFBFFFFF/BBFFFF<BBBBFFFFFFFFFFFFFFBFFFFFFFFFF<FFFF<FFFFFFFFFFFF</BFFFFFFFFFFFFFFF<F<BBBFFFFFFFFFFF

@GWZHISEQ02:315:C9E6MANXX:5:1213:7899:30738

TCGGTTTTTCGCCGAAACCGCGCCGCGCGTCTTGTCTGTCTCAGTGTTGTTTTGTCATTTGTCTGTTCGTTATTGTTTTGGACCGTTTCTAAAAATATGGGACAGACCGTAACCACCCCTCTGAGT

+

BBBBBFFFFFFFFFFFFFFBFFFFFFFFFFFFFFFFFFFFFFFFFFBFFFFFFFFFFFFFFFFFFFFFFFFFBFFFFFFFFFFFFFFFFFFFFFFFFFFFFFFFFFFFFFFFFFFFFFFFFFFFFB

@GWZHISEQ02:315:C9E6MANXX:5:1211:16469:21527

TTTTCGCCGAAACCGCGCCGCTCGTCTTGTCTGTCTCAGTGTTGTTTTGTCATTTGTCTGTTCGTTATTGTTTTGGCCCGTTTCTAAAAATATGGGACAGACCGTAACCACCCCTCTGAGTCTGAC

+

//<B/////<F///////////77<////<<F/<////<<F/</FFBFB<BFFFFF/<FF/<//<B//<////////7/<//F/B/<F/F//7<FF//<<//7/////7FBBF//77FFFFFFF//

@GWZHISEQ02:315:C9E6MANXX:5:1211:16469:21527

CTGGTTGGACGCGATGCGCTGGACGTCTCCCCAGTGTTCTAGGGTCTGACTCAGAGGGGTGGTTTCCGTCTGTTTCATTTTTGTTGTGCTGGTGAGGACTTAGAGCAAGTTACCTAACCTCTTTAT

+

/<BBBFF<FBFFB<BFFFFFBFBFBBFFFBBFF/BFFFFFFBFFBB/<FFF<FB///////<7F////<//<F7/BFFF/</////<///</<////<B/7F/FBF/<///7/<7FFB//B/7/</

@GWZHISEQ02:315:C9E6MANXX:5:1213:7899:30738

CCTGTAAAATAATGTCCAAATTAAAAGTACCATCTTGCGGCCACCCTACACCGAAAGTTGGCCACTCGGCAGAGCAGAAGGTGACCCAGCGTCTCTTCTTGACGTCCACGGACTGGTTGGACGCGA

+

BBBBBFFFFFFFFFFFFFFFFFFFFFFFFFFBFFFFFFFFBFFFFFFFFFFFFFFFFFFFFFFFFFFFBBBFFFFFFFFFFFFFFFFFFFFFFFFFFFFFFFFFFFF<FFFBFBFFFFFFFFFF</

@GWZHISEQ02:315:C9E6MANXX:5:2311:11267:90385

CCCGGTCCCCACGGACACCCGGATCAGGTCCCATACATTGTCACCTGGGAGGCTATTGCCTATGAACCCCCTCCGTGGGTCAAACCTTTTGTCTCTCCCAAACTCTCCCTCTCTCCAACCGCTCCC

+

BBBBBFFFFFFFFFFFFFFFFFFFFFFFFFFFFFFFFFFFFFFFFFFFFFFFFFFFFFFFF<FFFFFFBFFFFFFFFFFFFFFFFFFFFFFFFFFFFFFFFFFFFBFBFBF<FFFFFFFFFFBBFF

@GWZHISEQ02:315:C9E6MANXX:5:1112:14750:77661

CTATGAACCCCCTCCGTGGGTCAAACCTTTTGTCTCTCCCAAACTCTCCCTCTCTCCAACCGCTCCCATCCTCCCATCCGGTCCTTCGACCCAACCTCCGCCCCGATCTGCCCTTTACCCTGCTCT

+

BBBBBFFFFFFFFFFFFFFFFBFFFFFFFFFFFFFFFFFFBFFBFFF<FFFFFFFFFFFFFFFFFFFFFFFFFBFFFBFBBFFFFFFFFFFFFFFFFFFFFFFFFFFFFFFFBFFFFFFFFFFFF<

@GWZHISEQ02:315:C9E6MANXX:5:2101:16169:17630

GGTCAAACCTTTTGTCTCTCCCAAACTCTCCCTCTCTCCAACCGCTCCCATCCTCCCATCCGGTCCTTCGACCCAACCTCCGCCCCGATCTGCCCTTTACCCTGCTCTTACCCCCTCTATAAAACC

+

BBBBBFFFFFFFFFFFFFFFFFFFFFFFFFFFFFFFFFFFFFFFFFFFFFFFFFFFFFFFFFFFFFFFFFFFFFFFFFFFFFFFFFFFFFFFFFFFFFFFFFFFFFFFFFFFFFFBFFFFFFFFFF

@GWZHISEQ02:315:C9E6MANXX:5:2101:16169:17630

CCGCCATTATCGGAGAGAACCTGAGGTTTAGAAGGTCTGGGTTTTATAGAGGGGGTAAGAGCAGGGTAAAGGGCAGATCGGGGCGGAGGTTGGGTCGAAGGACCGGATGGGAGGATGGGAGCGGTT

+

BBBBBFFFFFFFFFFFFFFFFFFFFFFFFFFFFFFFFFFFFFFFFFFFFFFFFFF7FFFFFFFFFF<<BFFFFFFBFFFFFFFFFFFBBB/FBFFFF/7BFF<7BB77BFF77B//7FF/BFFF<7

@GWZHISEQ02:315:C9E6MANXX:5:1216:15167:34060

TGCTTCAATCATATTCAAACTCCTAGACCTTTACGTCCTTTAACTGATATCATTCTCCCATCCGGTCCTTCGACCCAACCTCCGCCCCGATCTGCCCTTTACCCTGCTCTTACCCCCTCTATAAAA

+

///</<////B//<<////<BFF///</////<<////<////<//////B/<//<FFF</BFB/F/</B</</F<//<7F</7F/7//<////</FFFFFFFFF/</BFFFFB//77B/BB<FFF

@GWZHISEQ02:315:C9E6MANXX:5:1112:14750:77661

TGAGAAGGTCAATGAGAGGTCCGCCATTATCGGAGAGAACCTGAGGTTTAGAAGGTCTGTGTTTTATAGAGGGGGTAAGAGCAGGGTAAAGGGCAGATCGGGGCGGAGGTTGGGTCGAAGGACCGG

+

BBBBBFFFFFFFFFFFFFFFFFFFFFFFFFFFFBFFFFFFFFFBFFFFFFFFFFFFFFFFF<FFFFFFFFFFFFFFFFFFFFFFFFFFFFFFFFFF/FFFFFFFFFBFFFFFFFBFFFFFFFFFFF

@GWZHISEQ02:315:C9E6MANXX:5:1216:15167:34060

CCCTGTTCTCCGTACGGCGGAGGGTCTTCTGTGAGAAGGTCAATGAGAGGTCCGCCATTATCGGAGAGAACCTGAGGTTTAGAAGGTCTGGGTTTTATAGAGGGGGTAAGAGCAGGGTAAAGGGGA

+

<B/BBFFFBFBFFFFFF/FBBBFFFFFFFFFFFF<BBFFFBF/BBFFBBFFFFFBF//FFFFFFFFFF//BFBFFBB<FFBB<F/</<BF///7</<B<<F<F///<<FFFB/777/7BF/B///7

@GWZHISEQ02:315:C9E6MANXX:5:1203:20171:3370

AAAACCCAGACCTTCTAAACCTCAGGTTCTCTCCGATAATGGCGGACCTCTCATTGACCTTCTCACAGAAGACCCTCCGCCGTACGGAGAACAGGGACCGTCCTCCTCTGACGGAGATGGCGACAG

+

BBBBBFFFFFFFFFFFFFFFFFFFFFFFFFFFFFFFFFFFFFFFFFFFFFFFFFFFFFFFFFFFFFFFFFFFFFFFFFFFFFFFFFFFFFFFFFFFFFFFFFFFFFFFFFFFFFFFFFFFFFFFFF

@GWZHISEQ02:315:C9E6MANXX:5:2311:11267:90385

GGGGGCAGGAATCTCAGGAGTGGAGGTGGCCTCTTCTCTGTCGCCATCTCCGTCAGAGGAGGACGGTCCCTGTTCTCCGTACGGCGGAGGGTCTTCTGTGAGAAGGTCAATGAGAGGTCCGCCATT

+

BBBBBFFFFFFFFFFFFFFFFFFFFFFFFFFFFFFFFFFFFFFFFFFFFFFFFFFBFFFBFFFFFFFFFFFFFFFFFFFFFFFFFFFBFFFFFFFFFFFFFFFFFFFFFFFFFFFFFFFBBBBFBB

@GWZHISEQ02:315:C9E6MANXX:5:1203:20171:3370

GAGAGGTGGTGGAAACTGCCGCGGGGGGGGTCTCTTTTGCCCCGCAAGCGAGACACCATGGGAGAGGGGGCAGGAATCTCAGGAGTGGAGGTGGCCTCTTCTCTGTCGCCATCTCCGTCAGAGGAG

+

BBBBBFFFFFFFFFFFFFFFFFFFFFFFFFFFFFFFFFFFFFFFFFFFFFFFFFFFFFFFFFFF<FFFFFFFFFFFFFFFFFFFFFFFFFFFFFFFFFFFFFFFFFFFFFFFFFFFFFFFFFFFFF

@GWZHISEQ02:315:C9E6MANXX:5:1316:19755:81700

AAGAGACCCCCCCCGCGGCAGTTTCCACCACCTCTCGGGCTTTCCCACTCCGTTTGGGGGGTAATGGTCAGTTGCAGTACTGGCCGTTTTCCTCCTCGGATCTATATAACTGGAAAAATAATAACC

+

BBBBBFFFFFFFFFFFFFFFFFFFFFFFF<FFFFFFFFBFFFFFFFFFFFFFFFFFFFFBFFFFFFFFFFFFFFFFFFFFFFFFFFFFFF7FFFFFFFB/BFFBFFFFFFFFF//7/BFFFFFFFF

@GWZHISEQ02:315:C9E6MANXX:5:2205:8901:73331

CTCGGATCTATATAACTGGAAAAATAATAACCCTTCCTTCTCTGAAGATCCAGGTAAATTGACTGCCTTAATCGAGTCTGTCCTCACCACCCACCAGCCTACTTGGGATGACTGTCAACAGTTGCT

+

BBBBBFFFFFFFFFFFFFFFFFFFFFFFFFFFFFFFFFFFFFFFFFFFFFFFFFFFFFFFFFFFFFFFFFFFFFFFFFFFFFFFFFFFFFFFFFFFFFFFFFFFFFFFFFFFFFFFFFFFFFFFFF

@GWZHISEQ02:315:C9E6MANXX:5:2111:4463:24620

CATCCATCAGCCTAGGTGGGATGACTGTCAGCAGTTGCTGGGGACTCTGCTGACAGGAGAAGAAAAGCAGCGGGTGCTCCTGGAAGCCAGAAAGGCAGTCCGGGGCGACGATGGCCGCCCCACCCA

+

<////</BFF/////////</F7F/BFF/B//F/BBFFFFBF<FFF/BF</F/F<BFF<//B/FFFFBFFF<BFFBFFBBF/BFFBFFFFFFFF<<<FFFFFFFFFF<<BFF/BBFFBFFFBBBF/

@GWZHISEQ02:315:C9E6MANXX:5:1206:17194:43466

GAAGAAAAGCAGCGGGTGCTCCTGGAAGCCAGAAAGGCAGTCCGGGGCGACGATGGCCGCCCCACCCAATTGCCCAATGAGATCGAGGCTGCCTTTCCCCTCAAACGTCCCGACTGGGACTACACC

+

BBBBBFFFFFFFFFFFFFFFFFFFFFFFFFFFFFFFFFFFFFFFFFFFFFFFFFFFFFFFFFFFFFFFFFFFFFFFFFFFFFFFFFFFFFFFFFFFFFFFFFFFFFFFFFFFFFFFFFFFFFFFFF

@GWZHISEQ02:315:C9E6MANXX:5:1316:19755:81700

ACCTCTAAGGGTGGTGTAGTCCCAGTCGGGACGTTTGAGGGGAAAGGCAGCCTCGATCTCATTGGGCAATTGGGTGGGGCGGCCATCGTCGCCCCGGACTGCCTTTCTGGCTTCCAGGAGCACCCG

+

BBBBBFFFFFFFFFFFFFFFFFFFF/FFFFFFFBFFFFFFFFFFFFFFFFFFFFFFFFFFFFFFFFFFFFFFFFFFFFFFFFBFFFFFFFFFFFFFFFFFFBFBFFFFFFFFFFFFFFFFFFBFFF

@GWZHISEQ02:315:C9E6MANXX:5:1306:1953:11533

CAGAAAGGCAGTCCGGGGCGACGATGGCCGCCCCACCCAATTGCCCAATGAGATCGAGGCTGCCTTTCCCCTCAAACGTCCCGACTGGGACTATACCACCCTTAGAGGTAGGAACCACCTAGTTCT

+

B/BBBFFFFFFFFFFBFBFFFFBBF//FFFBFB<BFFFFFFFFFFBBFFF/<FBFBFFFFFFFFFFFFF<FFFFFFFFFFFBF<FFFFFFFFFFFFFFFFFF<FFFFFFFFF7B7BFFFFFBFFF7

@GWZHISEQ02:315:C9E6MANXX:5:2111:4463:24620

CTGGTTCCTACCTCTAAGGGTGGTGTAGTCCCAGTCGGGACGTTTGAGGGGAAAGGCAGCCTCGATCTCATTGGGCAATTGGGTGGGGCGGCCAACGTCGCAGCGAACTAGCGTTCGGACGTGGAG

+

BBB<BFFFFFFFFBBFFFFF<<FFFFFFFFFFFFFFF<FFFF<B<<BFFFFFFFFFFFFFFFFF/F/FBFB<FBFB/FF/FB<///<//<///</</</<B//7////////7///7//7/B////

@GWZHISEQ02:315:C9E6MANXX:5:1306:1953:11533

CCGCCAAGAGCAGCTGGCGATAGAGAACTAGGTGGTTCCTACCTCTAAGGGTGGTATAGTCCCAGTCGGGACGTTTGAGGAGAAAGGCATCCTCGATCTCCTTGGGCAAGTGGCTGGAGCGGCCAT

+

BBBBBFFFFFFBFFFBFFFBFFFFFFFFFFFFFFFFFFFFFFFFFFFFFFFFFFFFFFBFFBFFFFFFB<FBFF//FF////<7</FFF//<B7F<///B7FFF///////////7/////7/777

@GWZHISEQ02:315:C9E6MANXX:5:2203:6010:23110

GCCCCACCCAATTGCCCAATGAGATCGAGGCTGCCTTTCCCCTCAAACGTCCCGACTGGGACTACACCACCCTTAGAGGTAGGAACCACCTAGTTCTCTATCGCCAGCTGCTCTTGGCGGGTCTCC

+

BBBBBFBFFFFFFFFFFFFFF/</<BF<FFFBFFFFFBFFFFFFFFBBFFB<FF/FBFB/<BFFFBFBFFFBBFFFFFF<BBF/<BF/7FFBFFF///BFFFBFF<FFFBBBFBF/BFFBBF<B/B

@GWZHISEQ02:315:C9E6MANXX:5:2205:8901:73331

GCATTTTGGAGACCCGCCAAGAGCAGCTGGCGATAGAGAACTAGGTGGTTCCTACCTCTAAGGGTGGTGTAGTCCCAGTCGGGACGTTTGAGGGGAAAGGCAGCCTCGATCTCATTGGGCAATTGG

+

BBBBBFFFFFFFFFFFFFFFFFFFFFFFFFFFFFFFFFFFFFFFFFFFFFFFFFFFFFFFFFFFBFFFFFFFFFFFFFFFFFFFFFFFFFFFFFFFFFFFFFFBFFFFFFFFFFFFFFFFFFFFFF

@GWZHISEQ02:315:C9E6MANXX:5:1216:18542:47234

CTCAAACGTCCCGACTGGGACTACACCACCCTTAGAGGTAGGAACCACCTAGTTCTCTATCGCCAGCTGCTCTTGGCGGGTCTCCAAAATGCGGGCAGGAGCCCCACCAATTTGGCTAAGGTAAAA

+

BBBBBFFFFFFFFFFFFFFFFFFFFFFFFFFFFFFFFFFFFFFFFFFFFFFFFFFFFFFFFFFFFFFFFFFFFFFFFFFFFFFFFFFFFFFFFFFFFFFFFFFFFFFFFFFFFFFFFFFFFFFFFF

@GWZHISEQ02:315:C9E6MANXX:5:1206:17194:43466

CTGGGTTATTCCTTTTACCTTAGCCAAATTGGTGGGGCTCCTGCCCGCATTTTGGAGACCCGCCAAGAGCAGCTGGCGATAGAGAACTAGGTGGTTCCTACCTCTAAGGGTGGTGTAGTCCCAGTC

+

BBBBBFFFFFFFFFFFFFFFFFFFFFFFFFFFFFFFFFFFFFFFFFFFFFFFFFFFFFFFFFFFFFFFFFFFFFFFFFFFFFFFFFFFFFFFFFFFFFFFFFFFFFFFFFFFFFFFFFFFFFFFFB

@GWZHISEQ02:315:C9E6MANXX:5:1216:18542:47234

GTTTCTTGCCCAGGGTCCTCAGGGTCATAAGGAGTGTATCTGCGATAGGCCTCTTTGAGTCTCTCTAGAAAGGCCGAGGGCGACTCGTTGGACCCCTGGGTTATTCCTTTTACCTTAGCCAAATTG

+

BBBBBFFFFFFFFFFFFFFFFFFFFFFFFFFFFFFFFFFFFFFFFFFFFFFFFFFFFFFFFFFFFFFFFFFFFFFFFFFFFFFFFFFFFFFFFFFFFFFFFFFFFFFFFFFFFFFFFFFFFFFFFF

@GWZHISEQ02:315:C9E6MANXX:5:2203:6010:23110

TGCTTCTCTCACTAAGTCCCCTAAGGTCTTATTTTTTAAGTCTTCTAACCGCTCTAACTTTCGACCAATGTCTGGAGCAGACTGCCAGATGAACGACATGGATACATTGGTTTCTTGCCCAGGGTC

+

<B</<<BFFFFFFFFFFBFFFFFFFBFFFFFFFFFFFFBFFFFFFFFFFFFFFFFFFFFFBFF/BFFFFFFFFF<FFFFFFFFFFFFFFFFBFBFFFB<FFBFBFFFFFFFFFBFFFFFFFFFFFF

@GWZHISEQ02:315:C9E6MANXX:5:2313:3055:47356

AAAGAAGCCACGGGGGCCCCGAGGACCGAGGCCCCAGACCTCCCTCCTGACCCTAGATGACTAGGGAGGTCAGGGTCAGGAGCCCCCCCCTGAACCCAGGATAACCCTTACTGTCGGGGGGCAACC

+

BBBBBFFFFFFFFFFFFFFFFFFFFFFFFFFFFFFFFFFFFFFFFFFFFFFBFFFFFFFFFFFFFFFFF<FFFFFFFFFFFFFFBFFFFF<FFFFFFFFFFFFFFFFFF<FFFFFFFFFFFFFFFF

@GWZHISEQ02:315:C9E6MANXX:5:1206:14347:77459

CCGAGGACCGAGGCCCCAGACCTCCCTCCTGACCCTAGATGACTAGGGAGGTCAGGGTCAGGAGCCCCCCCCTGAACCCAGGATAACCCTTACTGTCGGGGGGCAACCAGTCACCTTCCTGGTGGA

+

BBBBBFBFFFFFFFFFF<BFFFFFFFFFFFF/FFFFBFBFFFFFFFFF<FFFFFFFFFFFF/B/BFFFFFFFBB</FFFFFFFFFBF<FBF<FFFFFFBB<7BF/7B77/BFFFFFFBFFF<FBBB

@GWZHISEQ02:315:C9E6MANXX:5:1206:14347:77459

GTCAGCACGGAGTGTTGGGCCCCAGTATCCACCAGGAAGGTGACTGGTTGCCCCCCGACAGTAAGGGTTATCCTGGGTTCAGGGGGGGGCTCCTGACCCTGACCTCCCTAGTCATCTAGGGTCAGG

+

/<BBBBFB//FFFFFF<FFFBFF<FFFBFFFFFFBF//FBBFFFFB/<FFFFFFFFB<BBFFFF<<FFFF/FF<FBF//BFFF<BBB7BBBBBB</BBFFF<B/7FFFFFFBB/BFF/FFF7/FFB

@GWZHISEQ02:315:C9E6MANXX:5:2315:1445:61967

GGAGCCCCCCCCTGAACCCAGGATAACCCTTACTGTCGGGGGGCAACCAGTCACCTTCCTGGTGGATACTGGGGCCCAACACTCCGTGCTGACCCAGAACCCTGGACCCCTAAGTGACAGGTCTGC

+

BBBBBFFFFFFFF/<FFFFFBF/FFFFFFFFFFFFFFFFFFFFFF<BFBFBFFFFFFFFF<<7/BFFBB<<FFFFFF7/FFFBFF<7FFFFBBFFFFFBFFFF<F/7FFFFBFF/B/FFBFFF/7B

@GWZHISEQ02:315:C9E6MANXX:5:2315:1445:61967

CCTTACCGGTAGCCAGGTGCACCTTGCGATCTGTGGTCCAGTGATACCGCTTTCCTCCAGTAGCCCCTTGGACCCAGGCAGACCTGTCACTTAGGGGTCCAGGGTTCTGGGTCAGCACGGAGTGTT

+

BBBBBFFFFFFFFFFFFFFFBFFFFFFFFFFFFFFFFFFFFFFFFFFFFFFFFFFFFFFFFFFFFFFFFFFFFFFFFFFFFFFFFFFFFFFFFFFFFFFFFFFF7FFFFFFFFFFFFFFFBF/FFF

@GWZHISEQ02:315:C9E6MANXX:5:2313:3055:47356

TGGATCTGGGCCTTTAACTTAGTCAACAAGTCCCTTCCTAGCAAAGGATAAGGGCAGTCCGGCACATGGAGGAAAGAGTGAGTGACCTTACCGGTAGCCAGGTGCACCTTGCGATCTGTGGTCCAG

+

BBBBBFFF<BFFFFFFBFFFFFFFFFFFFFFFFFFFFFFBFFFBFBB<F<BFFFFB<F<BF<BFFFFFFBFFFFFF/<BFFFFFFBFFFFFFF/F/<FFFFFFFFFFFFFFFFF7FFBBFFFFFF<

@GWZHISEQ02:315:C9E6MANXX:5:2314:11942:17084

TTATCCTTTGCTAGGAAGGGACTTGTTGACTAAGTTAAAGGCCCAGATCCACTTCGAGGGATCGGGAGCTCAGGTTGTGGGACCAAAAGGACAGCCCCTGCAGGTGTTGACCCTTGGCATAGAGGA

+

BBBBBF<FBFBFFFFFFFFFFFFFFFFFFFFFFFFFFFFFFFFFFFFFFFFFFFFFFFFBFFFFBFBFFFFFFFFFFFFFFFFFFFFBFFFFFFFFBFFFFFFF/FFFFFFFFFFBFFFBFFFFFF

@GWZHISEQ02:315:C9E6MANXX:5:1311:3528:26442

GGGTTTCTCAGGTTCTGTGAAAAAGAGGGGAGCGCCTACAGGTGGTGACCCTTGGCATGGAGGATGAGTATCGGCTACATGAGACCTCAACAGAGCCGGATGTTTCTCTAGGGTCCACCTGGCTTT

+

///</////</F///<//<<///////<///7///////</</<//<///<</<F/F//<B//</<</<F///77//<F<<</////<FFBF//</<77B/7<<7</FBF7B77/77BFF/B//7F

@GWZHISEQ02:315:C9E6MANXX:5:2107:10520:93621

GTGTTGACCCTTGGCATAGAGGATGAGTATCGGCTACATGAGACCTCAACAGAGCCGGATGTTTCTCTAGGGTCCACCTGGCTTTCTGACTTTCCCCAGGCCTGGGCAGAAACCGGGGGCATGGGA

+

BBBBBFFFFFFFFFFFFFFFFFBFFFFFFFFFFFFFFFFFFFFFFFFFFFFFFFFFFFFFFFFFFFFFFFFFFFFFFFFFFFFFFFFFFFFFFFFFFFFFFFFFFBFFFBFFFFFFFFFFFFFFFF

@GWZHISEQ02:315:C9E6MANXX:5:1311:3528:26442

GGCTTCGTGTGACATGGGGTACTGTTTGATGGACACAGGGGTGGAGGTTGCCTTTAGAGGTATAATCAGAGGCGCTTGGCGAACTGCCAGTCTCATGCCTCCGGTTTCTGCTGAGGCCTGTGGTAG

+

BBBBBFBFFFFFFF<<FFFFBBFBF<F<BFFFBFFFFFFBFFB/<//<<F/FFFFFFBFF<FBFBF/</F/BF</BB<F//<<<FF7B/BFB/<///////<7FFFF/</////<7/7F///////

@GWZHISEQ02:315:C9E6MANXX:5:2314:11942:17084

GGACTGGCAAGGTACCAATATTCCCTGGTCCAACAGTCTCTGTATGTGGGGCTTGATCCCCAGTCTGGCTTCGTGTGACATGGGGTACTGTTTGATGGACACAGGGGTGGAGGTTGCCTTTAGAGG

+

BBBBBF<BF/B<FFFBBFFFFFFFFF/<F///FBFBFFFFBFFFFF<FFFFFFFBFFFFFFFF/<FFFF/<FFFBFFFFFFFFFFFFF<///<FFFFFFF<FBFFFFB//BFFFFFBF//7/<F/<

@GWZHISEQ02:315:C9E6MANXX:5:2206:16787:13492

TCTAAAGGCAACCTCCACCCCTATGTCCATCAAACAGTACCCCATGTCACACGAAGCCAGACTGGGGATCAAGCCCCACATACAGAGACTGTTGGACCAGGGAATATTGGTACCTTGCCAGTCCCC

+

<BBB/<F/F/B//FF/</</<<//<F<///<</<<F</BFFFB<FBFFFFFF<7FB/<FF/</</B/F/BFFBBB/FF/<<BFFFB</FFBBFB7/</<<</<FFBFF/7<//7FF//BB7F//7F

@GWZHISEQ02:315:C9E6MANXX:5:2206:16787:13492

GTTCCAGGGGGACTGGCAAGGTACCAATATTCCCTGGTCCAACAGTCTCTGTATGTGGGGCTTGATCCCCAGTCTGGCTTCGTGTGACATGGGGTACAGTTTGATGGACACAGGGCAGGTGGTCGC

+

/<//</<B<F/<7FFFFFFFFBB/BFF/FFBFFFFBFBFBFFFFFF<<FFB</FFFB<FBFFFFFF</F<//FBFB//FBF/FFFFF///</7///<//<//7</////////<///<////7//7

@GWZHISEQ02:315:C9E6MANXX:5:1204:5499:25169

CTGGGGATCAAGCCCCACATACAGAGACTGTTGGACCAGGGAATATTGGTACCTTGCCAGTCCCCCTGGCACACACCCCTGCTGCACGATAAGAAACCAGGGACTAATGATTACAGGCCTGTCCAG

+

<<<<<///B<//B<B<BF/B<FB<<B<</F<F</////B<//<</B<FBFF<//FF/B<BF<////<FB<</<</BF/7//</<///<<<7/////</<////7///B//////<<B7/7//77B/

@GWZHISEQ02:315:C9E6MANXX:5:1305:8446:21133

CTGGGGAACAAGACCCACATACAGAGACTGATATACCAGGGAATATTGGTACCTTGCCAGTCCCCCTGAAACACACACCAGCTGCGCGATAAGAAACCAGGGACTAATGATTACAAGCCCGTCCAG

+

<///<<B/<<////<<FF<B</<B</F///////<////</B/<</<B/<///</////</</FFB///<<<</F//<F/<F/FF////7/<F</BB7////<<B7FBFF//7B//7/7//77/B/

@GWZHISEQ02:315:C9E6MANXX:5:1204:5499:25169

TTTTAAATCAAGCACAGTGTACCACTGGTGGGACGGAGGGAGTCCACTTAAGAGGTTGTAAGGATTGGGCACGGTGGGGTGGATATCTTCCACCCGCTTGTTGACTTCTCTCAGATCCTGGACAGG

+

BBBBBFFFFFFFFFFFFFFFFFFFFFFFFFFFFFFFFFFFFFFFFFFFFFFFFFFFFFFFFFFFFFFFFFFFFFFFFFFFBFFFFFFFFFFFFFFFFFFBFFFFFFFFFFFFFFFFFFFFFFFFFF

@GWZHISEQ02:315:C9E6MANXX:5:1305:8446:21133

TTTTAAATCAAGCACAGTGTACCACTGGTGGGACGGAGGGAGTCCACTTAAGAGGTTGTAAGGATTGGGCACGGTGGGGTGGATATCTTCCACCCGCTTGTTGACTTCTCTCAGATCCTGGACAGG

+

B/<<BB/F<FFFBFFFFFFFFFFFF/<FFFBBFFFFFFBBB/BFBFFFFFFF<FF/BFFFBFF/<FFFFFFFFFFFFFFF<FFFFFFFF<B/FF<BFBFFFBF<FFFBFFFF//FBFFFFFFFF<F

@GWZHISEQ02:315:C9E6MANXX:5:2107:10520:93621

TCAGGCAGAAAAAGGCATCTTTTAAATCAAGCACAGTGTACCACTGGTGGGACGGAGGGAGTCCACTTAAGAGGTTGTAAGGATTGGGCACGGTGGGGTGGATATCTTCCACCCGCTTGTTGACTT

+

BBBBBFFFFFFFFFFFFFFFFFFFFFFFFFFFFFFFFFFFFFFFFFFFFFFFFFFFFFFFFFFFFFFFFFFFFFFFFFFFFFFFFFFFFFFFFFFFFFFFFBFFFFFFFFFFFFFFFFFFFFFFFF

@GWZHISEQ02:315:C9E6MANXX:5:2211:4847:23473

TCATTTTAATTCTGTGCCCCGGATACTGGGCGTCGGCCAAGAAAGCCCAAATCTGCCAGAAACAGGTTAAATACCTGGGGTACCTTCTGAGGGAGGGTCAGAGATGGCTGACTGAGGCTAGGAAAG

+

/</<///<////<///BB///<FFFF//////<////<F/F//B<//FFFF<FBFFFFFF/F//<</</FB</BFBBFFFF<FFBBF//FFF/////<7/FF7<BB/7F<F/B/BF<FFBF//7F<

@GWZHISEQ02:315:C9E6MANXX:5:2211:10494:23195

TCGATCCTATGCCATGAAAGTCGATATCTGCCAGAAACAGGCTAAATACCTGGGGTACCTTCTGAGGGAGGGTCAGAGATGGCTGACTGAGGCTAGAAAAGCGACTGTGATGGGGCAACCCGTTCC

+

<B//<//B///<FF<///<//B/<///<//<FBF///<FBF/<FBBF/<BF<<<B/7F/FFF/FBB<7<BFF<//<///<FBFB/<</FFFFB7BBFFFFF/<F7<BBB7/7///B/FFFF/7//B

@GWZHISEQ02:315:C9E6MANXX:5:1311:1262:65276

TACCTGGGGTACCTTCTGAGGGAGGGTCAGAGATGGCTGACTGAGGCTAGAAAAGAGACTGTGATGGGGCAACCCGTTCCAAAGACTCCTCGACAACTAAGGGAGTTCCTAGNNNCGGCAGGCTCC

+

BBBB/BFFBFFFFFFFFFFFFFFFFFFFFFFF<FBBFFFFF/<BFFFFFFFFFFFFFFFFFFF</FBFFFFFFFFF/</FFFFFFFFFFBFF<F<BFFFFFFFFBB/<<FF<###/77F/BF///7

@GWZHISEQ02:315:C9E6MANXX:5:1311:1262:65276

AGAAGGGCCTGTTTGATTTCTTGATAGGCCTTTTGCTGGTCTGGGCCCCAATTAAACAGAGTCCCCGTTTTGGTAAGAGGATACAAGGGGGCCGCCAGTTCCGCAAACCCAGGGATCCAGAGGCGG

+

BBB/BF<</<B/F/B/FFFFF/BFF/BF/F/F<BFBFFFF</<<<BFFB/7BB<//<FBB</F<<B</<///FB<FFFFFBFFF/<FFFFF<BFFF//</</F///7//7BF/7/7BF/FFF/7BF

@GWZHISEQ02:315:C9E6MANXX:5:2211:10494:23195

CCCAATTAAACAGAGTCCCCGTTTTGGTAAGAGGATACAAGGGGGCCGCCATTTCCGCAAACCCAGGGATCCAGTGGAGTGAAAAGGCATCGAACAGTATGAACAAGATTGGTAGGCACGGAAATA

+

BBBBBFFFFFFFFFFFFBFFFFFFFFFFFBF/BBFFFFFFFF/FFF</FBBFFF/////FFBFBBFF<FF/FF/7/<//////</<//</<///<///</7/77F///7//7/77/////7//7//

@GWZHISEQ02:315:C9E6MANXX:5:1211:5604:19626

CGGAAATGGCGGCCCCCTTGTATCCTCTTACCAAAACGGGGACTCTGTTTAATTGGGGCCCAGACCAGCAAAAGGCCTATCAAGAAATCAAACAGGCCCTTCTAACTGCCCCCGCCCTGGGATTGC

+

/<BBBFBFB<BFF////FF/FFF/<<//<B//BFFFFFF<FFFF/FFFFFFFFF</FFF////<<BFFBF/7FFFFFFFB</BBFFF<FFFF/F/</FB/FB<<FFFB//BFBFFFFFFFF/7BF/

@GWZHISEQ02:315:C9E6MANXX:5:2211:4847:23473

GGCGGGGGCAGTTAGAAGGGCCTGTTTGATTTCTTGATAGGCCTTTTGCTGGTCTGGGCCCCAATTAAACAGAGTCTCCGGTTTGATAGCATAATAGGAATTCCTTCCAAATCCACAAAGCAATGG

+

<BBBBFFFFFFFFFFFFFFFFFFFFFFFFFFFFFFFFFF//<FFFF<<</B<FF/FFF//B//<F//F7F<BFF<//</<///<7/<FF/B//<FB7/B//////7/FBF/BBF///BF<F///BF

@GWZHISEQ02:315:C9E6MANXX:5:2301:17383:92392

GGGGCCCAGACCAGCAAAAGGCCTATCAAGAAATCAAACAGGCCCTTCTAACTGCCCCCGCCCTGGGATTGCCAGATTTGACTAAGCCCTTTGAACTCTTTGTCGACGAGAAGCAGGGCTACGCCA

+

BBBBBFFFFFFFFFFFFFFFFFFFFFFFFFFFFFFFFFFFFFFFFFFFFFFFFFFFFFFFFFFFFFFFFFFFFFFFFFFFFFFFFFFFFFFFFFFFFFFFFFFFFFFFFFFFFFFFFFFFFFFFFF

@GWZHISEQ02:315:C9E6MANXX:5:1211:5604:19626

CCTGCTTCTCGTCGACAAAGAGTTCAAAGGGCTTAGTCAAATCTGGTAATCCCAGGGCTGGTCCTGTAATATATGCATGTTTGACGTTTTTATAGTTCTTTTTCTACTTTAGTTTTTTATTAAACA

+

BBBBBFFFFFFFFFFFFFFFFBBFFFFBF<FFBFFFFFFFB/FFF///<<//<B/</////<///<<////////<///<<F</</</B//<<///<<////</<//<///////<77/7/7<//<

@GWZHISEQ02:315:C9E6MANXX:5:2301:17383:92392

CCATCCGTAGGCAAGGGGGCCACCCGGCTGCCACTGGGTCTAGCTTTTTGGACAGGTAGGCCACAGGCCGACGCCAAGGTCCCAGTTTTTGCGTTAGGACGCCTTTGGCGTAGCCCTGCTTCTCGT

+

BBBBBFFFFFFFFFFFFFFFFFFFFFFFFFFFFFFFFFFFFFFFFFFFFFFFFFFFFFFFFFFFFFFFFFFFFFFFFFFFFFFFFFFBFFFFFFFFFFFFFFFFFFFFFFFFFFFFFFFFFFFFFF

@GWZHISEQ02:315:C9E6MANXX:5:2210:1275:65331

GTCCAAAAAGCTAGACCCAGTGGCAGCCGGGTGGCCCCCTTGCCTACGGATGGTAGCAGCCATTGCCGTTCTGACAAAAGATGCAGGCAAGCTAACTATGGGACAGCCGCTANNNATCCTGGCCCC

+

B<<B//B<FBBFF<BFFFF/B<<BFBBF<BFF<FBFF7FF/BF<FBFBFF<FFFFFF<B/<F<//<<FF7<//<<FFBF/B7BBBBF</BFFFB/F/B//B/<FFFFFBFBF###77<FFFF</7F

@GWZHISEQ02:315:C9E6MANXX:5:2311:17487:22491

GCTTGCTTTCCGCTGCTAGCAGCCATTGCCGTTCTGACAATAGATGCAGGCAAGCTAACTATGGGACAGCCGCTAGTCATCCTGGCCCCCCATGCAGTAGAGGCACTGGTCAAGCAACCAGATCGG

+

///<<////////<F/BF//<////<BFF<FBF</BFF<F/<<<</FFFFFF/BFFFFBFFFF<FFFF/B<BFFB<FFFBFFFFB<FB//FBF/7/FFB/F/</BFB//<<FFBFFFBFFFFBBFB

@GWZHISEQ02:315:C9E6MANXX:5:2311:17487:22491

GGTTGCTTGACCAGTGCCTCTACTGCATGGGGGGCCAGGATGACTAGCGGCTGTCCCATAGTTAGCTTGCCTGCATCTTATGGATAGATAGATGTTGCTTAGATAGATCTAGATATAGATAATATA

+

BBBBB<F<FFBFFFFFFFFFFFFFFFFFFFF<BBFFFFFFFFFFFFFBB<BBFB/F/F///7<//B/FF///////////<//////<//<7//////////<F///7/7/7B/7/<F/77BFB//

@GWZHISEQ02:315:C9E6MANXX:5:2210:1275:65331

GCTCCTTTTTCCGGTAGAGGGAGCAAGGTGGCAGGATTGAGGGCCACCACTGGTCCGAACTGAACTCGGTCAGTGTCTAGGAGCATTGCCCGGTAGCGGGTCATGCGGGCGTTGGATAGCCAGCGG

+

B/B/BBBFBFFFF<FBFBFB/FFFF/FF/FF<FFFFFFB///<BFBF/<//</FF/F//<F//FF<<F/<7F<FFF//<<//<B</BFBF/7/B/B/7B/BFB//FBFB<<F<///7/77BBBBB/

@GWZHISEQ02:315:C9E6MANXX:5:2316:7988:60423

GACCGAGTTCAGTTCGGACCAGTGGTGGCCCTCAATCCTGCCACCTTGCTCCCTCTACCGGAAAAAGGAGCCCCCCATGATTGCCTCGAGATCTTGGCTGAAACGCATGGAACCAGACCGGATCTC

+

<<BBBFFFFFFF<<FFF/BFFFFF<FFFFFFFFFBFFFFFBFFFFFFFFFBFFFFFFFFFF<<BFFFB</BBB/BBBFFBBFBBFFF//7BFFBFFFFF7<BFFF/FFFBFBFB/FFFFFFFFFFF

@GWZHISEQ02:315:C9E6MANXX:5:2316:7988:60423

TCGCTGTCCTTCTTGCAGAAAGCTGCTCCCATCGGTATACCAGGTGTGGTCGGCGTCTGGGATGGGCTGGTCGGTGAGATCCGGTCTGGTTCCATGCGTTTCAGCCAAGATCTCGAGGCAATCATG

+

BBBBBBFFFFFFFFFFFFFFFFFFFFBFFFFFFF<FFFFFB<F<FFFFFFF<BFFFFFFFFFFFFFFFFBFFFFFFFFFBFFFFFFFFFFFFF<FFFBFFFFF/7FF77//7BFFFFBFFF/7/BF

@GWZHISEQ02:315:C9E6MANXX:5:1313:19416:25969

GAAACGCATGGAACCAGACCGGATCTCACCGACCAGCCCATCCCAGACGCCGACCACACCTGGTATACCGATGGGAGCAGCTTTCTGCAAGAAGGACAGCGAAAGGCTGGGGCAGCAGTGACGACT

+

B<BBBFFFFFF/BF//F</FB//</<F<F<F//7FFFB/F<B/FFBBB<7FB//<BFFFFF<FBFFFFF/</777B<F<77FFBFFFFFFFB///7B7FFF<7FF/F//7B//7FFBBFF/BBB//

@GWZHISEQ02:315:C9E6MANXX:5:1106:12360:8939

CATGGAACCAGACCGGATCTCACCGACCAGCCCATCCCAGACGCCGACCACACCTGGTATACCGATGGGAGCAGCTTTCTGCAAGAAGGACAGCGAAAGGCTGGGGCAGCAGTGACGACTGAAACC

+

<B/B//FBFFF//FFB/<7</FFF</F7/BFB/FFFFFF</FF//B<FF/FFFFFFBFFFFFF/F<FFB</BFFFFFFFB/FB<<FFBF/FBFFFFF<B</FBFFFBFFFFFFFFFFFBBFFFFFB

@GWZHISEQ02:315:C9E6MANXX:5:2306:18487:35442

CGGATCTCACCGACCAGCCCATCCCAGACGCTGACCACCCCTGGCATTCCGATGGGAGCAGCTTTCTGCAAGCAGGACAGCGAAAGGCCCGGGCAGCAGTGCCGACTGAAACCGAGGCCATCTGGG

+

///</<F////B///<FB////</FFF//<//<7/F///<FFFF/////<F//<B//F///////<<B/////////<F/<</7/<F////7/7//7//FF/////7B////////////////7/

@GWZHISEQ02:315:C9E6MANXX:5:1313:19416:25969

GTGCGATCAGTTCGGCTCGCTGGGCTGACGTTCCAGCTGGCAGGGCCCTCGCCCAGATTACCTCGGTTTCAGTCGTCACTGCTGCCCCAGCCTTTCGCTGTCCTTCTTGCAGAAAGCTGCTCCCAT

+

BBB<<<FFFFFFFFFFFFFFFFBBFFFFFFFFFBFFFFB<FFBFFFFFBFBF/BFFF<B<FFFFFFFFFFFFFFFFFFFF<FFFFFFFFFFFFF/<BF/BFFFFBFFBFFFFFFFFFFFFFFFF</

@GWZHISEQ02:315:C9E6MANXX:5:2306:18487:35442

CTTGGGTGAGTGCGATCAGTTCGGCTCGCTGGGCTGACGTTCCAGCTGGCAGGGCCCTCGCCCAGATTACCTCGGTTTCAGTCGTCACTGCTGCACCACCCCTTCGCCGTCCTTCTTGCAGTAAGC

+

<B/<B<<FF/FFFFFFFFFBFFFFFFFFFFFBFFFFFFFF/FBFF<F/<<B//7BFFFFFB<FFFFF/7F/F/<7///</<////<//<//////<</////7BBFB//7/7B/B/////////</

@GWZHISEQ02:315:C9E6MANXX:5:1105:4869:84816

GGCAGAAGGTAAGAAGCTAAATGTTTACACTGATAGCCGCTATGCCTTCGCTACGGCCCATGTTCATGGGGAAATATATAGGAGACGGGGGTTGCTGACCTCAGAAGGCAAGGAAATCAAGAACAA

+

BBBBBFFFFFFFFFBFFFFFFFFFFFFFFFFFFFFFFFFFFFFFFFFFFFFFFFFFFFFFFFFFFFFFFFFFFFFFFFFFFFFFFFFFFFFFFFFFFFFFFFFFFFFFFFFFFFFFFFFFFBBFFF

@GWZHISEQ02:315:C9E6MANXX:5:2303:15096:58494

CTACGGCCCATGTTCATGGGGAAATATATAGGAGACGGGGGTTGCTGACCTCAGAAGGCAAGGAAATCAAGAACAAAAGCGAGATCCTAGCCTTGCTGAAAGCCCTCTTTTTGCCAAAGAGACTCA

+

BBBBBFFFFFFFFFFFFFFFFFFFFFFFFFFFFFFFFFFFFFFFFFFFFFFFFFFFFFFFFFFFFFFFFFFFFFFFFFFFFFFFFFFFFFFFFFFFFFFFFFFFFFFFFFFFFFFFFFFFFFFFFF

@GWZHISEQ02:315:C9E6MANXX:5:1106:12360:8939

ATACTGAGTCTCTTTGGCAAAAAGAGGGCTTTCAGCAAGGCTAGGATCTCGCTTTTGTTCTTGATTTCCTTGCCTTCTGAGGTCAGCAACCCCCGTCTCCTATATATTTCCCCATGAACATGGGCC

+

B/<BBF<BFB/B/F<////<<</7<<F//<BF<FF/FFBFFFBFF<F/FFFFFB/<F/<FFFFFFBFFFFFFFFFFBF</BFFB/BFF<FBFFFFFFFFBB/<FFBFF7<BFFF<F//</<B//77

@GWZHISEQ02:315:C9E6MANXX:5:2104:5238:7343

AGGAGACGGGGGTTGCTGACCTCAGAAGGCAAGGAAATCAAGAACAAAAGCGAGATCCTAGCCTTGCTGAAAGCCCTCTTTTTGCCAAAGAGACTCAGTATTATCCATTGCCCAGGACATCAGAAA

+

BBBBBFFFFFFFFFFFFFFFFFFFFFFFFFFFFFFFFFFFFFFFFFFFFFFFFFFFFFFFFFFFFFFFFFFFFFFFFFFFFFFFFFFFFFFFFFFFFFFFFFFFFFFFFFFFFF/FFFFFFFFFFF

@GWZHISEQ02:315:C9E6MANXX:5:1105:4869:84816

GCCTCTGGCTTCGGCACTGTCTCCTTTCTGATGTCCTGGGCAATGGATAATACTGAGTCTCTTTGGCAAAAAGAGGGCTTTCAGCAAGGCTAGGATCTCGCTTTTGTTCTTGATTTCCTTGCCTTC

+

BBBBBFFFFFFFFFFFFFFFFFFFFFFFFFFFFFFFFFFFFFFFFFFFFFFFFFFFFFFFFFFFFFFFFFF<FBFFFFFFFFFFFFFFFFFFFFFFFFFFFFFFFFFFFFFFFFFFFFFFFFFFF/

@GWZHISEQ02:315:C9E6MANXX:5:2101:14338:49331

GACTCAGTATTATCCATTGCCCAGGACATCAGAAAGGAGACAGTGCCGAAGCCAGAGGCAACCGTATGGCAGACCAGGCGGCCCGAGAGGCAGCCACAAAAACAGTTCCAGAAGCCTCTACACTCC

+

B/BBBFBFFFFFFFFB/</FFFFFFBFFFFFF<BFBFFFFFFFFFFFFFFFBFFFFFFFFFFFFFBFFFFBBFFFFFFFFFFFFFFBBBFFFFFFFFFFFFFFFFFFFBBFBFFFFBFBFFFFFFF

@GWZHISEQ02:315:C9E6MANXX:5:2116:13092:16999

TATTTATCATTTAGGAGACAGTGCCTAAGCCAGAGGCAACCGTATGGCAGACCATGCGGCCCGAGAGGCAGCCACAATAACAGTTCCAGAAGCCTCTACACTCCTTATAGAGGACTCGACCCCGTA

+

//////B/<////<</<<B/<<//B/<<<F/F///</BF/<<B/F////<///FB/F/</</BF//</77</<F<<F/<FFF/<F/<F/BBF/B//BFFBBFF<FB7/FB//<FF7<7B7/7/<B<

@GWZHISEQ02:315:C9E6MANXX:5:2303:15096:58494

GTTTCGGTGTAATGGAGATAGGCAGGCGTGTACGGGGTCGAGTCCTCTATAAGGAGTGTAGAGGCTTCTGGAACTGTTTTTGTGGCTGCCTCTCGGGCCGCCTGGTCTGCCATACGGTTGCCTCTG

+

BBBBBFFFFFFFFFFFFFFFFFFFFFFFFFFFFFFFFFFFFFFFFFFFFFFFFFFFFFFFFFFFFFFFFFF<FFFFFFBFFFFFFFFFFFFFFFFFFFFFBFFFFFFFFFFFFFFFFFFFBFFFFB

@GWZHISEQ02:315:C9E6MANXX:5:1214:16382:74237

CACAAAAACAGTTCCAGAAGCCTCTACACTCCTTATAGAGGACTCGACCCCGTACACGCCTGCCTATCTCCATTACACCGAAACAGATCTAAAAAGATTGCGAGAACTGGGGGCCACCTATAATCA

+

BBBBBFFFFFFFFFBFFFFFFFFFFFFFFFFFFFFFFFFFFFFFFF<FFFFFFFFFFFBFFFFFFBFFFFFFFFFFFFFFFFFFFF/7FFFFFFFFFFFFFFFB<FFFFFFFFFFFFFFFF/////

@GWZHISEQ02:315:C9E6MANXX:5:2101:14338:49331

TGCCTTGTAGGACCCAATATCCTTTTATCTGATTATAGGTGGCCCCCAGTTCTCGCAATCTTTTTAGATCTGTTTCGGTGTAATGGAGATAGGCAGGCGTGTACGGGGTCGAGTCCTCTATAAGGA

+

B<BBBFFFFFFFFFFFFFFFFFFFFFFFFFFFFFFFFFFFFFFFFFFFFFFFFFFFBFFFFFFFFFFFFFFFBFFFFFBFFFFFFF<FBFFFFFBFFFFFFFFFFFFFFFFB/<FFFFF/BFFFF/

@GWZHISEQ02:315:C9E6MANXX:5:2116:13092:16999

GGAGGGCTGAGATGGGTGAGTCTATGAAGGGAGTCTAATAATTCAAACACAAACTGATCGGGCATCACCGGCTTGCCTTGTAGGACCCAATATCCTTTATATGATTGTAGGTGGCCCCCAGTTCTC

+

B<BB//<FFFF<B<F<FB/BFBFFFFBFB<FFFFFFBFFFFFFFFFFFFFB<FFFFFFFFF/FBFFFFFFFFFFBFF<FF/F/FFBFFFFBFF//FF/<FFF7FBF/<<7/<FB/F<<BFFF/B//

@GWZHISEQ02:315:C9E6MANXX:5:1214:16382:74237

ATCTTTTGAGGGCTGAGATGGGTGAGTCTATGAAGGGAGTCTAATAATTCAAACACAAACTGATCGGGCATCACCGGCTTGCCTTGTAGGACCCAATATCCTTTTATCTGATTATAGGTGGCCCCC

+

BBBBBFFFFFFFFFFFFBFFFFFFBFFFFFFFFFFFFFF7FFFFFFFFFFFFFFFFFFFFFFFFFBFFFFFFFFFFFF<FBBF<FFFFFFBFFFFFF<FFFFFFFBFFBFFFF<FFFFF/BFBFFF

@GWZHISEQ02:315:C9E6MANXX:5:2104:5238:7343

GCAGGATTCTGCCACATACTGAAGAGTTCTGTCCCTGTTTAACATGTAGTAGGGGCTTTCTTCTCTGTCAAGGAGCGCCTTCATCTTTTGAGGGCTGAGATGGGTGAGTCTATGAAGGGAGTCTAA

+

BBBBBFFFFFFFFFFFFFFFFFFFFFFFFFFFFFFFFFFFFFFFFFFFFFFFFFFFFFFFFFFFFFFFFFFFFFFFFFFBFFFFFFFFFFFFFFFFBFFFFFFFFFFFFFBFFFFFFFFFF7FFFF

@GWZHISEQ02:315:C9E6MANXX:5:1301:8458:2829

GCAGAATCCTGCACAGTCTGTGCTCAAGTAAATGCTAGTAAAGCCAAAATCGGGGCAGGGGTACGAGTACGCGGACATCGACCAGGTACCCATTGAGATCGGAAGAGCGTCGTGTAGGGAAAGAGT

+

B/BBBFFFFFB<FF<F//<FFF<<<FFFFF/FFFFFBFFFFFFFFFFF<FBBFFFBFBBFF/FFFBFFFFFFFFBFBBFBF<F<FFFFFBFB<BBFFFFFFFBFF<FFFFFB7/7/F<BFBB/BF7

@GWZHISEQ02:315:C9E6MANXX:5:1301:8458:2829

CAATGGGTACCTGGTCGATGTCCGCGTACTCGTACCCCTGCCCCGATTTTGGCTTTACTAGCATTTACTTGAGCACAGACTGTGCAGGATTCTGCAGATCGGAAGAGCACACGTCTGAACTCCATC

+

<BB<BFBFFFFF<FBB<BFFFFBFFFFFFFFFFFFFFFFBFFFFFFFFFFFB<FFFFFBFF<FBF<F<<F<</FFFFFF<FFFB/FBB/BF/<FFFF<FFF</F///77F/7BFFFF/BFFBF///

@GWZHISEQ02:315:C9E6MANXX:5:2304:21121:41854

CACTGAAGTTAAACCAGGGCTGTACGGGTACAAGTACCTCCTGGTGTTCGTAGACACCTTCTCTGGCTGGGTGGAAGCCTTCCCAACTAAACGTGAAACTGCCAAGGTTGTGACCAAGAAGCTATT

+

/BB<<FB/FF<<F/BFBFFFFFFFFFFBFFFFFFFF<BFFFFF<FBFFFBFFBFFFFFBFFFFFFFFFFFFFFFFFFF<FFFFFFFFFFFFBFFFFFFFFFFFFFFFFF7FBFF/FFFFFFFFFFB

@GWZHISEQ02:315:C9E6MANXX:5:2102:5704:7290

CTGGCTGGGTGGAAGCCTTCCCAACTAAACGTGAAACTGCCAAGGTTGTGACCAAGAAGCTATTAGAAGAAATATTCCCAAGATTCGGGATGCCACAGGTATTGGGTTCCGATAATGGGCCTGCCT

+

BBBBBFFFFFFFFFFFFFFFFFFFFFFFFFFFFFFFFFFFFFFFFBBFFFFFFFFFFFFFFFFFFFFFFFFFFFFFFFFFFFFFFFFFFF<FFFFFFFFFFFFFFFBFBFF/FFFFF/FFFBBFFF

@GWZHISEQ02:315:C9E6MANXX:5:1201:14037:84826

TTTAGGAGAAATATTCCCAAGATTGGGTATGCTACAGGTACTGGGGTCCGTTAATGGGCCTGCGTTCGTCTCCCAGGTAAGGCAGCCGGTGGCCGATGTACTGGGGCTCGTTTGGAAATTACATTG

+

/<//////<FB/F///////B////////////<//B</<//////7//7///<F/<<///<//<//7F/F/<//</<B/F//FF/////<//7//7//////////<B//777/7///<//<///

@GWZHISEQ02:315:C9E6MANXX:5:2304:21121:41854

AACTCTGGGGTCTATAAGCACAATGTAATTTCCAATCGATCCCCAGTAAATCGGCCACCGACTGACTTACCTGGGAGACGAAGGCAGGCCCATTATCGGAACCCAATACCTGTGGCATCCAGAATC

+

BBBBBFF/FFFFFFFFFFFFFFFFFFFFFFFFFFFFFFFFFFFFFFFFBFBFFFFFFFFFFFFFFFFFFFBFFFFFFFFFFFFFFFFFFFFFFFFFFFFFFBBFFFFFFFFFFFFFFFFF/BFFBF

@GWZHISEQ02:315:C9E6MANXX:5:1201:14037:84826

AGTAGGAGTACCCAGTCTCTAGTGCCAGCTGCAAGCGTTAATTTAGTTAGAGTCTCCTTGATGGTTCTATTCATTCTTTCTACCTGACCTGAACTCTGGGGTCTATAAGCACAATGTAATCTCCAA

+

/<<BBFFFFFF<FFFFFFFF</F//BFFFFFFFFFFF<BFFFFFFFFFFBBFFFFBFBBFBF<FFFFFFFFF<FFFFBBF<F/FF//F/FFFFFBFFB/FBF/FFFF///7<<FFFFFBF/<BBFB

@GWZHISEQ02:315:C9E6MANXX:5:1310:3679:42670

GAAATTACATTGTGCTTATAGACCCCAGAGTTCAGGTCAGGTAGAAAGAATGAATAGAACCATCAAGGAGACTCTAACTAAATTAACGCTTGCAGCTGGCACTAGAGACTGGGTACTCCTACTCCC

+

BBBBBFFFFFFFFFFFFFFFFFFFFFFFFFFFFFFFFFFFFFFFFFFFFFFFFFFFFFFFFFFFFFFFFFFFFFFFFFFFFFFFFFFFFFFFFFFFFFFFFFFFFFFFFFFFFFFFFFFFFFFFFF

@GWZHISEQ02:315:C9E6MANXX:5:1316:11349:84209

CCAGAGTTCAGGTCAGGTAGAAAGAATGAATAGAACCATCAAGGAGACTCTAACTAAATTAACGCTTGCAGCTGGCACTAGAGACTGGGTACTCCTACTCCCCTTAGCCCTCTACCGAGCCCGGAA

+

BBBBBFFFFFFFBFFFFFFFFFFFFFFFFFFBFFFFFFFFFFFBFFFFFFFFFFFFFFFFFFFFF<FFFFFFFFFFFFFFFFFFFFFFFBFF<FFFFFFFFFFFF<FFFFFFFFFFFFBFFFFFFB

@GWZHISEQ02:315:C9E6MANXX:5:1310:3679:42670

GGGGGCCCGGAGTGTTCCGGGCTCGGTAGAGGGCTAAGGGGAGTAGGAGTACCCAGTCTCTAGTGCCAGCTGCAAGCGTTAATTTAGTTAGAGTCTCCTTGATGGTTCTATTCATTCTTTCTACCT

+

BBBBBFFFFFFFFFFFFFFFFFFFFFFFFFFFFFFFFFFFFFFFFFFFFFFFFFFFFFFFFFFFFFFFFFFFFFFFFFFFFFFFFFFFFFFFFFFFFFFFFFFFFFFFFFFFFFFFFFFFFFFFFF

@GWZHISEQ02:315:C9E6MANXX:5:2102:5704:7290

GGGGTGCCCCATACAGAATTTCATACGGAGTCAGTCCATGGGGGCCCGGAGTGTTCCGGGCTCGGTAGAGGGCTAAGGGGAGTAGGAGTACCCAGTCTCTAGTGCCAGCTGCAAGCGTTAATTTAG

+

BBBBBFFFFFFFFFFFFFFFFFFFFFFFFFFFFFFFFFFFFFFFFFFFFFFFBFFFFFFFFFFFFFFFFFFFFFFFFFFFBF<FFFFFFFFFFFBFFFFFBFFFFFFFFFFFFFFFF7BFFFBFFF

@GWZHISEQ02:315:C9E6MANXX:5:1316:11349:84209

TGTGGTATCACTGGCTGATCTAGCTGGTCCTGATAAGCAGCGGCCAGCGGCTTCCAGACCTCTCGTTGTACTGCTTGGAGGGCCTGTAAGTGAGCTTGGAGAGAGGGACTATTAGTTAACTTTGAC

+

BBBBBFFFFFFFFFFFFFFFFFFFFFFFFFFFFFFFFFFFFFFFFFFFFFFFFFFFFFFFFFFFFFFFFFFFFFFFFFFFFFFFFFFFFFBFFFFFFFFFFFFFFFF/BFFFFBF<FFFFFFFFFF

@GWZHISEQ02:315:C9E6MANXX:5:1211:8535:19081

TGGCCAGGCTGATCTCAAACTCCTGATCTCAGGTGACCCACCCACGGCAGGCGTAGGGTCCTTTCCAGCGAGGTGCCAAGTTCTTAGTCTGGTGCCGGCGTACCCACACGGCGTCACCGACACCGA

+

<<BBBBB/FBFFFFFFFFFFFFFFFF<F///<FBB<<//<F7/////<7/B/7//</////</<//<//<B<7</<BF/////<7FFF7/F//FBB7<7/7<FB//7FFF//7/7FB/7BFBB//<

@GWZHISEQ02:315:C9E6MANXX:5:2215:16090:100439

ATAAGATTAACCCGTGGGGCCCCCTAATAGTTATAGGGATCTTGGTGAGGGCAGGAGCCTCGGTACAACGTGACAGCCCTCACCAGGTCTTCAATGTCACTTGGAGAGTTACCAACCTAATGACAG

+

BBBBBFFFFFFFFFFFFFFFFFFFFFFFFFFFFFFFFFFFFFFFFFFFFFFFFFFFFFFFFFFFFFFFFFFFFFFFFFFFFFFFFFF<F<FFFFFFFFFFFFFFFFFFBFFFFFFFFFFFFFFFFF

@GWZHISEQ02:315:C9E6MANXX:5:2306:7179:15083

GGGATCTTGGTGAGGGCAGGAGCCTCGGTACAACGTGACAGCCCTCACCAGGTCTTCAATGTCACTTGGAGAGTTACCAACCTAATGACAGGACAAACAGCTAACGCTACCTCCCTCCTGGGGACG

+

///BBFFFBFB</<BFF/<//BFFFFF<FFBFFFFFBB/FBFFFFFBFFFFFFFFFFFFFFFFFFFFFFFFBB<FFFFFFFFFFBF<FFBBF<FFFBBFFBF<F7BFF/B//<B//<7/BFF</B/

@GWZHISEQ02:315:C9E6MANXX:5:2111:12290:20375

GCCCTCACCAGGTCTTCAATGTCACTTGGAGAGTTACCAACCTAATGACAGGACAAACAGCTAACGCTACCTCCCTCCTGGGGACGATGACAGACACCTTCCCTAAACTATATTTTGACTTGTGTG

+

B/<BB</B/<<BB<FFFFFFFF<FFFFF<BFFBFFBF/FFFBBFBBFFFFFFFFFFFFFFBFB<FFF<FF/<FFBFFBFFFF/FFFFFBFFF</7/BBBFFFFFFFFF7/<BF/<FFFBFFBFFF/

@GWZHISEQ02:315:C9E6MANXX:5:1211:15078:79981

TCAATGTCACTTGGAGAGTTACCAACCTAATGACAGGACAAACAGCTAACGCTACCTCCCTCCTGGGGACGATGACAGACACCTTCCCTAAACTAAATTTTGACTTGTGTGATTTAGTTGGAGACC

+

BBBBBFFFFFFFFFFFFFFFFFFFFFFFFFFFFFFFFFFFFFFFFFFFFFFFFFFFFFFFFFFFFFFFFFFFFFFFFFFFFFFFFFFFFFFFFFFFFFFFFFFFFFFFFFFFFFFFFFFFFFFFFF

@GWZHISEQ02:315:C9E6MANXX:5:2306:7179:15083

TTCTGGGTCATCCCAATGGTCTCCAACTAAATCACACAAGTCAAAATATAGTTTAGGGAAGGTGTCTGTCATCGTCCCCAGGAGGGAGGTAGCGTTAGCTGTTTGTCCTGCAAATAGTTAGGAAAC

+

BBBBBFFFFFFFFFFFFFFFFFBFFFFF/F</<FFFF<BFF/F<FB/<F/<BFFFFFFFF<<FFFFBB/BFF<FFFFFBFF//77/FBF/B/BF////<//<//<BF/F</////<////<<////

@GWZHISEQ02:315:C9E6MANXX:5:2216:5100:32328

TAACCTAATGACAGGACAAACAGCTAACGCTACCTCCCTCCTGGGGACGATGACAGACACCTTCCCTAAACTATATTTTGACTTGTGTGATTTAGTTGGAGACCATTGGGATGACCCAGAACCCGA

+

/<//////<FF////B</</FF//<BBB/7//7</<B<F//<F/<BFFBFFFFFF<BFBFFFFFFFFFFFFFFBFF/BF<FFFBF</FF<BFB7F7FFFFFFFFFFF<BBBB/7FFFFFFFFFFF7

@GWZHISEQ02:315:C9E6MANXX:5:2215:16090:100439

AGAGCGGCAACCATCTCCAATATCGGGTTCTGGGTCATCCCAATGGTCTCCAACTAAATCACACAAGTCAAAATATAGTTTAGGGAAGGTGTCTGTCATCGTCCCCAGGAGGGAGGTAGCGTTAGC

+

BBBBBFFFFFFFFFFFFFFFFFFFFFFFFFFFFFFFFFFFFFFFFFFFFFFFFFFFFFFFFFFFFFFFFFFFFFFFFFFFFFFFFFFFBFFFFFFFFBFFFFFFFFFFFBFFFBFFFFFFF/B<BF

@GWZHISEQ02:315:C9E6MANXX:5:2216:5100:32328

CCGGAGAGCGGCAACCATCTCCAATATCGGGTTCTGGGTCATCCCAATGGTCTCCAACTAAATCACACAAGTCAAAATATAGTTTAGGGAAGGTGTCTGTCATCGTCCCCATGAGGGAGGTTGCGA

+

BBBB/////</<7<FBB<B/BFFFBFFF<FBFBFFFFFFFBBFFFF<FF/FFBFBFFF/B<BFFFFFFFFFFB/F</<FFFBB<FFFF/<FBBFFFFB7/F/77<7F/</B/7BF/BB/F7/77B/

@GWZHISEQ02:315:C9E6MANXX:5:2116:5855:24949

CATATTCTTTTTCTTTTCTTTTCTTTTCTTTTTTTTTTTTTTTCGTTTTCTGGGTCTTCCCTTTTGTCTCCATCTAAATCACACAAGTCAAAATATAGTTTAGGGAAGGTGTCTGTCATCGTCCTT

+

BBBBBF/FFFFFFF/FBBF/<FBBF<BFB/<<FF<<////</7B/////<//<////<//<//<//<7F//<//<///B/<7/<//<</B/</</B/<7/BFB//FF</<7/7BFB/F///7///7

@GWZHISEQ02:315:C9E6MANXX:5:2111:12290:20375

GTCCTTTTTCTTCCCCCCGGAGAGCGGCAACCATCTCCAATATCGGGTTCTGGGTCATTCCAATGGTCACTAACTAAATCCCACAAATTACAATAGAGTTAAGTATATTTGTCTGTCATCAGCCCG

+

/B<BBFFFFFFFFBFFFFFFFFFFFFFFFF///BFFBF<FFFFFFB/F/</<F///<</////<////////<<F///F/////<//////</77/B//</<7//<<//<///<///7////////

@GWZHISEQ02:315:C9E6MANXX:5:2211:13854:21404

CTTCTCATTAGTGTGATACAGATGGAGACCATTGGGATGACCCATAACCCGATTTTGGAGATGGCTGCCGCGCTCCGGGGGGAAGAAACAGGACAATACTGTATGACTTCTATGTTTGCCCCGGTC

+

/<<///B///////BF///BF/<FFF//F/<///////////////<///</7/////<F/F////<///////<FB7//////<F///7B/B/7///////////<BFBF////////7///77/

@GWZHISEQ02:315:C9E6MANXX:5:1314:8994:91649

ATTTAGTTGGAGACCATTGGGATGACCCAGAACCCGATATTGGAGATGGTTGCCGCTCTCCGGGGGGAAGAAAAAGGACAAGACTGTATGACTTCTATGTTTGCCCCGGTCATACTGTACCAATAG

+

BBBBBFFFFFFFFFFFFFFFFFFFFFFFFFFFFFFFFFFFFFFFFFFFFFFFFFFFFFFFFFFFFFFFFFFFFFFFFFFFFFFFFFFFFFFFFFFFFFFFFFFFFFFFFFFFFFFFFFFFFFFFFF

@GWZHISEQ02:315:C9E6MANXX:5:1304:12737:69459

CCAGAACCCGATATTGGAGATGGTTGCCGCTCTCCGGGGGGAAGAAAAAGGACAAGACTGTATGACTTCCCCGGTCATACTGTACCAATAGGGTGTGGAGGGCCGGGAGAGGGCTCAGCTACCCCC

+

BBBBBFBF/B<FBFF/<<BFFFFFF<<<F</<BBFFBFFFFFFFFFFFBFF/B<FF/F<7BFFFFBFFBFFFFF<BFFFFFFFB/FFFFFFFFFFFFBFFFFBBFF<<BFFB//FFFFFFFBFFFF

@GWZHISEQ02:315:C9E6MANXX:5:1211:15078:79981

CCCCATTTGCCACAGTAGCCCTCTCCCGGCCCTCCACACCCTATTGGTACAGTATGACCGGGGCAAACATAGAAGTCATACAGTCTTGTCCTTTTTCTTCCCCCCGGAGAGCGGCAACCATCTCCA

+

BBBBBFFFFFFFFFFFFFFFFFFFFFFFFFFFFFFFFFFFFFFFFFFFFFFFFFFFFFFFFFFFFFFFFFFFFFFFFFFFFFFFFFFFFFFFFFFFFFFFFFFFFFFFFFFFFFFFFFFFFFFFFF

@GWZHISEQ02:315:C9E6MANXX:5:2211:13854:21404

CCCTATTGGTACAGTATGACCGGGGCAAACATAGAAGTCATACCGTCTCGTCTTTTCTCTTCCAGTTGGTACAAGAAGAGCGTTTCCAAACTGCTGCATCAAAAGAGAGCTTCCACCCTGTAAGCT

+

/</BB/BBFFFF/BFFF</FFF<//FBFFFBFF//</F/F/F////7///<7//<///<<B/F//////7////////B//7</FBBFF//<///<7B//<</</B////////7///</7/////

@GWZHISEQ02:315:C9E6MANXX:5:1314:8994:91649

CTGATCCTTAGGAGTGTTTCCTCGCTTAAGGGAAATTAGGTCCCATGATGATGATGGCTTCCAGTATGCCTGTCCAGTGGTCTCACATCCCCATTTGCCACAGTAGCCCTCTCCCGGCCCTCCACA

+

BBBBBFFFFFFFFF<FFFFFFFFFFFFFFFFF<FFFFFFFFFFFFFFFFFFFFFFFFFFFFFFFFFFFFFFFFFFFFFFFFFFFFFFFFFFFFFFFFFF<FFFFFFFFFFFFFFFFBFBFFFFFF/

@GWZHISEQ02:315:C9E6MANXX:5:1108:10506:30828

TTCCCTTAAGCGAGGAAACACTCCTAAGGATCAGGGCCCCTGTTATGATTCCTCGGTCTCCAGTGGCGTCCAGGGTGCCACACCGGGGGGTCGATGCAACCCCCTAGTCTTAGAATTCACTGACGC

+

BBBBBFFFFFFFFFFFFFFFFFFFFFFFFFFFFFFFFFFFFFFFFFFFFFFFFFFFFFFFFFFFFFFFFFFFFFFFF<FFFFFBFFFFFFFFFFFFFFFFFFFFFFFFFFFFFFFFFFFFFFBFF<

@GWZHISEQ02:315:C9E6MANXX:5:1109:20907:84801

CCAGTGGCGTCCAGGGTGCCACACCGGGGGGTCGATGCAACCCCCTAGTCTTAGAATTCACTGACGCGGGTAAAAAGGCCAGCTGGGATGCCCCCAAAGTTTGGGGACTAAGACTCTACCGATCCA

+

BBBBBFFBFFFFFFFBB7<FFFFF<BBFFF<BBFFFF/BFFBB<FFFFFFFFFFFFFFFB<FFFFFFFFFBFFFFF<<FBBFFFFFFFFFFFFFFFFFFFFFFF<F/BFFBFF<FFFFFFFFBFFF

@GWZHISEQ02:315:C9E6MANXX:5:1108:10506:30828

CAGTGATCACGGGATTAGGCCCAATGGGGACGCGGGGTCCTACATTGAGGACCTGGCGGGTCAAAGAGAACCGGGTCACCGGGTCGGCCCCCGTGGATCGGTAGAGTCTTAGTCCCCAAACTTTGG

+

BBBBBFFFFFFFFFFFFFFFFFFFFFFFFFFFFFFFF/FFFFFFFFFFFFFFFFFFFFFFBFFFFFFFFFFFFFFFFFFFFFFFFFFFFFFFFFFFFFFFFFFFFF/BBFFFFFFFF/BFFFFFFF

@GWZHISEQ02:315:C9E6MANXX:5:1109:20907:84801

GGAGGAGGATGAGGAGGCCTGGGGAGCATGATCTGCACGGGTTGGGAGGGGGGTAGCTGTTCAGTGATCACGGGATTAGGCCCAATGGGGACGCGGGGTCCTACATTGAGGACCTGGCGGGTCAAA

+

BBBBBFFFBFBFFFFFFFFFFFFFFFFFFFFFFFFFFFFFFFFFFFFFFFFFFBFFFFFFFFFFFBFFFFFBBFFFFFFF<F<BB<FFFFF<FFFBFF/FFBFFFFFFFFFFFFFFFFFFF<<F/B

@GWZHISEQ02:315:C9E6MANXX:5:2111:11653:19227

ATGGACTCTGCGTAGGAGCAGTTCCCAAAACCCATCAGGCCCTGTGTAATACCACCCAGAAGGCGAGCGACGGGTCCTACTATCTGGCTGCTCCCGCCGGGACCATCTGGGCTTGCAACACCGGGC

+

B///<///<<//<///B/<</<////FB////7BFFF/B</7FFB<FFBFB/FFFBF/<<F<F<FFBB<BBBF/7/<</BF//F<FBBBFB/7FB/FFFFBB<B/B///7/BFFFFFFBBBF//B/

@GWZHISEQ02:315:C9E6MANXX:5:2311:18611:19839

TTCTGGGCTTGCAACACCGGGCTCACTCCCTGCCTATCTACCACTGTACTCAACCTCACCACCGATTACTGTGTCCTGGTTGAGCTCTGGCCAAAGGTGACCTACCACTCCCCTGGCTATGTTTAT

+

/<<BB////</<<FFF/<FBF<B/<FFF<FBBFFFBFFFFFFFFFFFFFFFFFFFFFFFFFFF/7<FFFB<FFFFFFFFFFFBBBFFBFF/FFFFFFFBFFFFF<BFFFF<FBFF</<FFFFFBBF

@GWZHISEQ02:315:C9E6MANXX:5:1306:17522:18317

GTATCACCGGGCTCACTCCCTGCCTATCTACCACAGTACTCAACCTCACCACCGATTACTGTGTCCTGGTTGAGCTCTGGCCAAAGGTGACCTACCACTCCCCTGGTTATGTTTATGACCAGTTTG

+

</<///B<//<F//BFFFFBB/F<<F///<//<///</FF//B//</<////7//F7BFFFFFFFFF//<<7/</<BBBFFF/B///<F7FFFFFBFFBFFFFFFFFBFFBFBB/F</77/<BFFB

@GWZHISEQ02:315:C9E6MANXX:5:2111:11653:19227

GCCTTTGGCCAGAGCTCAACCAGGACACAGTAATCGGTGGTGCGGCTGAGTCCGCTTCCTGAGAAACTGGGAGTACAGCTACATGCACCCATGCCCAGCTAATGTTTTGTATTTTTAATAGAGACG

+

/BBBBBFFFFFFFFF/FFFFFFFF<FBFFF<BBFFFB/</F/////<F//<////////<FF//</B/BFFF/F//</<//<BBBBB/B7F//BB/B/</7////<//<FF/7////<<F<FF//7

@GWZHISEQ02:315:C9E6MANXX:5:1304:12737:69459

CTCCTAACAGCAGGGCCAGAGTTAATGACACCGGCTCTCTTTTATATTTGGTTTTTCTCTCAAACTGGTCATAAACATAACCAGGGGAGTGGTAGGTGTTGGGAGGGGGGTAGCTGAGCCCTCTCC

+

//B</BFB<B/FBFFFF<FF/<<F<F<FFFFFBFB//F/FF/F<FBFFB/BF/FFFFF<FFFB<FF<FFFBF//</<F<BFFFFBF<7F7BF<B<FFB/<B//7FF<FF/B/7<BF///777F/B/

@GWZHISEQ02:315:C9E6MANXX:5:2311:18611:19839

CCCAACAGCAGGGCCAGAGTTAATGACACCGGCTCTCTTTTATATTTGGTTTTTCTCGCAAACTTGTCATTAACAGAATTATGCGCTGTTTAGAACAATCATGTCCAGTGATTACTTTCGACACAT

+

BBBBBFFFFFFFFFFFFBFFFFFFFFFFFFBBBFFFFFFFFBFFF<<</</<F///<//<//<<///</<////<///////////7/</<//7//</////////<<7/7//</<//////////

@GWZHISEQ02:315:C9E6MANXX:5:2111:2999:25586

CCTGTTCCTACTCCTGCAGCTATGCCGCCCATAGTAAGTCCTCCCAACAGCAGGGCCAGAGTTAATGACACCGGCTCTCTTTTATATTTGGTTTTTAACCAGGGGAGAGTTAAATAGAGGGATAGC

+

BBBBBFBFFFFFFFFFFFFFFFFFFFFFFFFFFFFFFFFFFFFFFFFFFFFFFFFFFFFFF<FFFFFFFFFFFFFFFFFFB<FFFF/FFFF<7F/<////7/<//<///7////7/7//////7/7

@GWZHISEQ02:315:C9E6MANXX:5:2111:2999:25586

GTACCACTCCATTGGTTAAAATATAGATATTAAAGAGAGCCGGTGTCATTAACTCTGGCCCTGCTGTTGGGAGGACTTACTATGGGCGGCATAGCTGCAGGAGTAGGAACAGGAGATCGGAAGAGC

+

/<<B////<///<//<FB</F//////</</<<F/BF<7<FB/7FFBBFFB/<BF/<FBFFFFF/FFFFFFFFBFFFFBFFFFFFFFFF/FFFFFFFFFFFFFFFFFFFFFFFFFFFFFFFFFFF/

@GWZHISEQ02:315:C9E6MANXX:5:1306:17522:18317

GCCTGGAGCTGCTCGAATTGTTTGGTGGCCACTAGGGCTGTAGTCCCTGTTCCTACTCCTGCAGCTATGCCGCCCATAGTAAGTCCTCCCAACAGCAAGTCCGGAGGTAAGGAGATGGGCTGTCGT

+

<B/B///<F<//B</F<F<FB<BFFF<F//FF/FFFFFFBFFFFFFF<FF/<FFFF<<BF/FF<FF/</FFBFFBB/<B/B/<F//</<////</F///////////7/<//</<///<///////

@GWZHISEQ02:315:C9E6MANXX:5:2106:20103:14988

CTCCACACAATTCGAGCAGCTCCAGGCAGCCATACATACAGACCTTGGGGCCTTAGAAAAATCAGTCAGTGCCCTAGAAAAGTCTCTGACCTCGTTGTCTGAGGTGGTCCTACAGAACCGGAGAGG

+

//<<//</</<<<F/</F<///FF//</7B<<FBFFFFFBFB/BFF<//FFFFFBBF///FFF/BFFF/<<//FBFF/F<F/BFFBF/BBF/BBBBFFFF/F/B<BFBF</BFFFFF/<</B<B</

@GWZHISEQ02:315:C9E6MANXX:5:1302:7912:17701

CCAGGCAGCCATACATACAGACCTTGGGGCCTTAGAAAAATCAGTCAGTGCCCTAGAAAAGTCTCTGACCTCGTTGTCTGAGGTGGTCCTACAGAACCGGAGAGGATTAGATCTGCTGTTCCTAAA

+

BBBBBFFFFFFFFFFFBFFFFFFFFFFF<FFFFFFFFFFFFFFFFFFFFFFFFFFFFFFFFFFFFFFFFFFFFFFFFFFFFFFFFFFFFFFFFFFFFFFFBFFFFFFFFFFFFFBFFFFFFFB<FF

@GWZHISEQ02:315:C9E6MANXX:5:1111:4460:13156

GCCATACATACAGACCATGAGGCCTTAGAAAAATCAGTCAATGCCCTAGAAAAGTCTCTGACCTCGTTGTCTGAGGTGGCCACACAGAACGGGAGAGGATTAGAACAGCTGTTCCCAAAAGAAGGA

+

<//<<</B//B///BF//////<//<//<FF/FFFF/B/<///<///BF/////BF/<F////////////<<<///<//B///7/<BF////B///<F////</////////////<BFBFFF<F

@GWZHISEQ02:315:C9E6MANXX:5:1307:13577:22071

CATACATACAGACCTTGGGGCCTTAGAAAAATCAGTCAGTGCCCTAGAAAAGTCTCTGACCTCGTTGTCTGAGGTGGTCCTACAGAACCGGAGAGGATTAGATCTGCTGTTCCTAAAAGAAGGAGA

+

BBBBBFFFB<FFFFFFFFFFFFFFFFFBBFFFFFFFFFFFFFFFFFFFFFFFFFFFFFFFFFFFFFFFF<B<FFFFFBFFFFFFFFF/FFF77B<BBFFFFFFFFFFFFFFFFFFFFFFFFFFFFF

@GWZHISEQ02:315:C9E6MANXX:5:1307:13577:22071

CTCCTTCTTTTAGGAACAGCAGATCTAATCCTCTCCGGTTCTGTAGGACCACCTCAGACAACGAGGTCAGAGACTTTTCTAGGGCACTGACTGATTTTTCTAAGGCCCCAAGGTCTGTATGTATGA

+

<B<<BBFFBFFBFFFFFFFFFFFFFFFFFFFFFFFFFB<BF<FFFB/FFFFFFFFFFFFFFFFFFFFBBFFBFFBFFFFFFFFFFBFFFFFBFFFBFFFFFFFFFFFFFFFFFFFFFFF/BBFFBB

@GWZHISEQ02:315:C9E6MANXX:5:1111:4460:13156

TCTTACTACGCCAGTGTGGTCTGCATAGAAACAGCATTCTTCTTTTAGGGCAGCACATAATCCTCCTTCTTTTAGGAACAGCAGATCTAATCCTCTCCGGTTCTGTAGGACCACCTCAGACAACGA

+

</<BB<F///BBF<FFFFFFFFFFBFF<FBFFF//FFF<BF<B/<F/<///<//<<BB<FBF/<F<FF/<FFF</<<</BFFBFFFFFFFFFFB<FFF<BBFFFFFFFFFFB<7FFFFF/7/<</7

@GWZHISEQ02:315:C9E6MANXX:5:2106:20103:14988

TCTGGTTTAGCCTTTCTCTTAGCTTAGCCATGCTATCCCTTACTACGCCAGTGTGGTCTGCATAGAAACAGCATTCTTCTTTTAGGTCAGCAGATATGCTTTCTATTTCTCTGATCATCAGCTCTG

+

BBB/BBFFFFF<FB<FBFFFB<FFFFFFFFFBFFFFFFFFFFFFF<FFB<B/FFBF/BFF/FFFF/</<<B////<</<F<F/////</<////</////</////<F/B///<7/</FFF///</

@GWZHISEQ02:315:C9E6MANXX:5:1301:6619:31393

GGGATAGCATGGCTAAGCTAAGAGAAAGGCTAAACCAGAGGCAAAAATTGTTCGAATCAGGACAAGGGTGGTTTGAGGGACTGTTTAACAGGTCCCCATGGTTCACGACCCTGATATCCACCATTA

+

BBBBBFFFFFFFFFFFBFFFFFFFFFFFFFFFFFFFFFFFFFFFFFFFFFFFFFFFFFBFFFFFFFBFFFFFFFFFFFFFFFFBFFFFFFFFFFFFFFFFFFFFFFFFFFFFFFFFFFFFFFFBFF

@GWZHISEQ02:315:C9E6MANXX:5:1111:20137:22059

CCTGGCTAAGCTAAGAGAAAGGCTAAACCAGAGGCAAAAATTGTTCGAATCAGGACAAGGGTGGTTTGAGGGACTGTTTAACAGGTCCCCATGGTTCACGACCCTGATATCCACCATTATGGGCCC

+

//<</FF/B///<</F/<<FF///<//<FBFFBFFFFFFBB<B<FFFFF/7BFF/FFBF<F<B7<BFFFFB//B<BFFFBFFB<BFFBFFFFFFFFFBFBBFFFFFFFFFFB<B<<FBFFFFFFFF

@GWZHISEQ02:315:C9E6MANXX:5:1302:7912:17701

CGGTTGAGAATGCAGGGTCCGAGGAGTAGGATTAATAAAAGTACTATCAGAGGGCCCATAATGGTGGATATCAGGGTCGTGAACCATGGGGACCTGTTAAACAGTCCCTCAAACCACCCTTGTCCT

+

BBBBBFF/FFFFFFFF<<FFFFBFFFFFFFFFFFFFFFFFFFFFFFFBBFFFFBBB/FFBBFFFFFBFFFBFFFBBBFFBF/BBFFFFFFFF/FBFBB</FFFF<7FFFFFFFFFFFFFFFFFFFF

@GWZHISEQ02:315:C9E6MANXX:5:1111:20137:22059

CGAGGAGTAGGATTAATAAAAGTACTATCAGAGGGCCCATAATGGTGGATATCAGGGTCGTGAACCATGGGGACCTGTTAAACAGCCTCTCAAATCACCCTTGTCCGTAGCTGGGCACTACTGGTC

+

BBBBB<FFFFFFFFFFFFFFFBFFFFFFFFFFFFFFFFFFFBFFB<<FBB/FFFF/BF<F////FFF///////7/</<<BB/B//</B/B////<//<BF//<//</////<///<7F///////

@GWZHISEQ02:315:C9E6MANXX:5:2302:11762:100303

CTTTTACAAACTGGACCAAGCGGTTGAGAATGCAGGGTCCGAGGAGTAGGATTAATAAAAGTACTATCAGAGGGCCCATAATGGTGGATATCAGGGTCGTGAACCATGGGGACCTGTTAAACAGTC

+

//BBBB<BFB<F/F/FBB/FFF/<7FFFFFFB/BFF</</BFF<FFFFFF/BBFFFFFFFFFF//B<B<BF/<BFFFFF<FFB<F<<BF<FBBB//<BBBFFF//<BB</7F/BF7BFFFFBBBFF

@GWZHISEQ02:315:C9E6MANXX:5:1301:6619:31393

GAAATTCTGTCTTTTACAAACTGGACCAAGCGGTTGAGAATGCAGGGTCCGAGGAGTAGGATTAATAAAAGTACTATCAGAGGGCCCATAATGGTGGATATCAGGGTCGTGAACCATGGGGACCTG

+

BBBBBFBFFFFFFFFFFFFFFFFFFFFFFFFFFFFFFFFFFFFFFFFFFFFFFFFFFFFFFFFFFFFFFFFFFFFFFFFFFFFFFFFFFFFFFFFFFFFFFFFFFBFFFFFFFFFFFFFFFBFFFF

@GWZHISEQ02:315:C9E6MANXX:5:1309:12386:33325

CAGGCCCTGGTTCTGACCCAACAGTATCACCAACTCAAATCAATAGATCCAGAAGAAGTAGAATCGCGTGAATAAAAGATTTTATTCAGTTTCCAGAAAGAGGGGGGAATGAAAGACCCCACCATA

+

B<BB<FBFFF/</FFBF/BBBFFFFFFFFFFFFFFFF</B/BFFBFB/FFFFFFFFFFFBFFF/FFBFFBBFFFFFFFFFFFFFFBBFF<FBBFF/FFFFFF/B/B/77BB/F/FF/7BBFFFBFB

@GWZHISEQ02:315:C9E6MANXX:5:1211:8417:25846

CTGGTTAAGACCCAACAGTATCAATAACTCAAATCAATAGATCCAGAAGAAGTAGAATCGCGTGAATAAAAGATTTTATTCAGTTTCCAGAAAGAGGGGGGAATGAAAGACCCCACCATAAGGCTT

+

BBB<<//////<<B<BB/B<//B//<////F/</FFBFFF/FB//B<FB//FBFFFFFB<<B/FFBFFBFBFFFFFFFFFBFFFFF/<FFFFFFFFFFFFFFFFFFFFFFFFFFFFBFFFFFFFFF

@GWZHISEQ02:315:C9E6MANXX:5:1211:8417:25846

CTAGCTTGCTAAGCCTTATGGTGGGGTCTTTCATTCCCCCCTCTTTCTGGAAACTGAATAAAATCTTTTATTCACGCGATTCTACTTCTTCTGGATCTATTGATTTGAGTTGGTGATACTGTTGGG

+

BBBBBFFFFFFFFFFFFFFBFFFFFFFFFFFFFFFFBFFFFFFFFFFFFFFFFFFFFFFFFFFFFFFFFFFFBBFFFF/FFFFBFFFFFFFBF</FFF/<BBFFFFFFBF<<FBB//F7FF/77B/

@GWZHISEQ02:315:C9E6MANXX:5:2205:7040:66196

AACTCAAATCAATAGATCCAGAAGAAGTAGAATCGCGTGAATAAAAGATTTTATTCAGTTTCCAGAAAGAGGGGGGAATGAAAGACCCCACCATAAGGCTTAGCAAGCTAGCTGCAGTAACGCCAT

+

BBBBBFF/FFFFFFFFFBFBF/BFFFFFFFFFFFFFFFFFBFFFFFFFFFFFFFFFFFFFFFFBF<FFFFFFFFFF<BFFFFFFFFFFBBFBFFFFFFFFFFFFFFFFFFFFFFFFFFFFFFFFFF

@GWZHISEQ02:315:C9E6MANXX:5:2202:5347:97874

TTACTTTTTCATGCCTTGCAAAATGGCGTTACTGCAGCTAGCTTGCTAAGACTTATGGTGGGGTCTTTCATTCCCCCCTCTTTCTGGAAACTGAATAAAATATTTTATTCACGCGATTCTACTTCT

+

<<</BFBFBFFF//<BF//<B///B/B<7<FFF/FF<BFFFFFF///BB//</B/B/<///</<<BB/</<F///<<FBB<<B/F//</F77/<//7BF<7//<<//7//</7BB<//<////BBF

@GWZHISEQ02:315:C9E6MANXX:5:2316:5175:12032

ATTTTATTCAGTTTCCAGAAAGAGGGGGGAATGAAAGACCCCACCATAAGGCTTAGCAAGCTAGCTGCAGTAACGCCATTTTGCAAGGCATGAAAAAGTACCAGAGCTGAGTTCTCAAAAGTCACA

+

/BBBBF/<<F<F/F/B//F/</F</////<F////<</////<//F/B7/////BF</F//F/<//<//7//</<</7BBFF/////7B7/<B/BBB77FFB///77BFFFF/7/////77B7BBF

@GWZHISEQ02:315:C9E6MANXX:5:2316:5175:12032

TGAGTGGAACAAACTAAGGAAGGTTTTAGTTAGGGGAGTAATACAGTTTTGTACAGTATTATCCCTTGCGTTATATCTATACTGCTGCTAGCTTTCTAAGCCTTATGGTTGGGTCGGTCATCCCGC

+

B<BBB/BFFFB<F/BFF/<B//<<BF/<F<//////<F7F//////B///B<<///<//////F///////////////////BF/F//<FF/<///BB<////B/F///<B/////7<7///7//

@GWZHISEQ02:315:C9E6MANXX:5:1110:18712:91872

CAGAGTGATTGACTACCCAGCTCGGGGGTCTTTCATTAATGAAAGACCCCACCATAAGGCTTAGCAAGCTAGCTGCAGTAACGCCATTTTGCAAGGCATGAAAAAGTACCAGAGCTGAGTTCTCAA

+

BBBBBFFFFFFFFFFFFFFFFFFFFFFFBFFFFFFFFFFFFFFFFFFFFFFFFFFFFFFFFFFFFFFFFFFFFFBFFFFFFFFFFFFFFFFFFFFFFFFFFFFFFFFFFFFFFFFFFFFFFFFFFF

@GWZHISEQ02:315:C9E6MANXX:5:1204:7943:88338

AAAGCCTTTTGCTGTTTGCATCCGAAACGTGGCCTCGCTGTTCCTTGGGAGGGTCTCCTCAGAGTGATTGACTACCCAGCTCGGGGGTCTTTCATTAATGAAAGACCCCACCATAAGGCTTAGCAA

+

BBBBBFFFFFFFFFFFFFFFFFFFFFFFFFFFFFF/FFFFFFFFFFFFFFFFFFFFFFFFFFFF<FBFFFFFFFFFFFFFFFFFFFF<FFFFFFFFFFFFFFFFBFFFFFFFFFFFFFFFFFFFBB

@GWZHISEQ02:315:C9E6MANXX:5:1311:11580:86179

AACTTCCTTGTGACTTTTGAGAACTCAGCTCTGGTACTTTTTCATGCCTTGCAAAATGGCGTTACTGCAGCTAGCTTGCTAAGCCTTATGGTGGGGTCTTTCATTAATGAAAGACCCCCGAGCTGG

+

BBBBBFFFFFFFFFFFFBF/FFBFFFFFFBFFFFFFFFFFFFBFFFFFFFFFFFFFFBFFFFFB/FFFFFFFFFFB/BFBFFFFFFFFFBBFFFFF///<FF<FF/FFFBFBB/B//<7/7<FFFF

@GWZHISEQ02:315:C9E6MANXX:5:1204:5121:8932

GGTACTTTTTCATGCCTTGCAAAATGGCGTTACTGCAGCTAGCTTGCTAAGCCTTATGGTGGGGTCTTTCAGGTTTAGGTGCAGGAGCGGTCGCCCAGGTAGCGGCGGGTCCGGCAGGATTTAGGG

+

BBBBBFFFFFFFFFFFFFFFFFFFFFFFFFFFFFFFFFFFFFFFFFFFFFFFFFFFFFFFFFFFFFFFFFFFFFFFFFFFFFFFFFFFFFFFFFFFFFFBFFFFFFFFF/7F<F/7BFB7FFFFF/

@GWZHISEQ02:315:C9E6MANXX:5:1309:12386:33325

CCCAGTTTTGTTCAGCCTTATTCTTTAACTAAACTTCCTTGTGACTTTTGAGAACTCAGCTCTGTTACTTTTTCATGCCTTGCAAAATGGCGTTACTGCAGCTAGCTTGCTAAGCCTTATGGTGTG

+

<<B/<<FF<F/FF<FFFFFBF/BBBFFBFFBF<FF<//<FFB//BFFFF<<F/BFFFBF<FBFB///<FFFF<FFFF/<FFF<BB//<BBFF<F//<FF/B</F<<BB//<B/<7F<//BFFF</<

@GWZHISEQ02:315:C9E6MANXX:5:2111:6317:95273

CTGCAGTAACGCCATTTTGCAAGGCATGAAAAAGTACCAGAGCTGAGTTCTCAAAAGTCACAAGGAAGTCTAGGTAAAGAATAAGGCTGAACAAAACTGGGACAGGGGCCAAACAGGATATCTGTG

+

BBBBBFF<<F/</F/<BB/</F<F//BF</BFF//<BBFF/<F/FFF//FFF<FF/FFB/FB//<FFFF/</</<<<<FF/FB/<FFFFBF/FFFFF/B<BFBF/7/</F<FFB<FB/7/B/<FFF

@GWZHISEQ02:315:C9E6MANXX:5:2205:7040:66196

TGGCCCTGAGCCGGGGCCCAGGTGCTCGACCACAGATATCCTGTTTGGCCCCTGTCCCAGTTTTGTTCAGCCTTATTCTTTAACTAAACTTCCTTGTGACTTTTGAGAACTCAGCTCTGGTACTTT

+

BBBBBFFFFFFFFFFFFFFFFFFFFFFFFFFFFFFFFFFFFFFFFFFFFFFFFFFFFFFFFFFFFFFFFFFFFFFFFFFFFFFFFFFFFFFFFFFFFFFFFFFFFFFFFFFFFFFFFFFFFFFFFF

@GWZHISEQ02:315:C9E6MANXX:5:1110:18712:91872

GTTTCGCTTTATCTGAGTACCATCTGTTCTTGGCCCTGAGCCGGGGCCCAGGTGCTCGACCACAGATATCCTGTTTGGCCCCTGTCCCAGTTTTGTTCAGCCTTATTCTTTAACTAAACTTCCTTG

+

BBBBBFFFFFFFFFFFFFFFFFFFFFFFFFFFFFFFFFFFFFFFFFFFFFFFFFFFFFFFFFFFFFFFFFFFFFFFFFFFFFFFFFFFFFFFFFFFFFFFFFFFFFFFFFFFFFFFFFFFFFFFFF

@GWZHISEQ02:315:C9E6MANXX:5:1316:2081:67946

GAATAAGGCTGAACAAAACTGGGACAGGGGCCAAACAGGATATCTGTGGTCGAGCACCTGGGCCCCGGCTCAGGGCCAAGAACAGATGGTACTCAGATAAAGCGAAACTAGCAACAGTTTCTGGAA

+

BBBBBFFFFFFFFFFFFFFFFFFFFFFFFFFFFFFFFFFFFFFFFFFFFFFFFFFFFFFFFFFFFFFFFFB<FFFFBBFFFFFFFFFFFBFFFFFFFFFBFFFFFFFFFFFFFBFFFBFFFFFFFF

@GWZHISEQ02:315:C9E6MANXX:5:1204:7943:88338

TGGGGTTTTTCCCGGTCTTTTGGGGAACTTGAAACTGAGGTGGGACTTTCCAGAAACTGTTGCTAGTTTCGCTTTATCTGAGTACCATCTGTTCTTGGCCCTGAGCCGGGGCCCAGGTGCTCGACC

+

BBBBBFFFFFFFFFFFFFFFFFFFFBFFFFFFFFFFFFFFFFFFFFFFFFFFFFFFFFFFFFFFFFFFFFFFFFFFFFFFFFFFFFFFFFFFFFFBFBBFFFFBFFFBFFBFFFFFFFFF/FFBBF

@GWZHISEQ02:315:C9E6MANXX:5:1308:10160:27438

GGCCCCGGCTCAGGGCCAAGAACAGATGGTACTCAGATAAAGCGAAACTAGCAACAGTTTCTGGAAAGTCCCACCTCAGTTTCAAGTTCCCCAAAAGACCGGGAAAAACCCCAAGCCTTATTTAAA

+

BBBBBFFFFFFFFFFFFFFFFFFFFFFFFFFFFFFFFFFFFFFFFFFFFFFFFFFFFFFFFFFFFFFFFFFFFFFFFFFFFFFFFFFFFFFFFFFFFFFFFFFFFFFFFFFFFFFFFFFFFFF<FF

@GWZHISEQ02:315:C9E6MANXX:5:1311:20397:22565

GTGAGGTAAAGCGAAACTAGCAACAGTTTCTGGAAAGTCCCACCTCAGTTTCAAGTTCCCCAAAAGACCGGGAAAAACCCCAAGCCTTATTTAAACTAACCAATCAGCTCGCTTCTCGCTTCTGTA

+

///////B///////<//<F///FFF/</<//<B/FFFF<///F//FB/</<B<F<<FFF<BFFBFBFFFFFBBF/FF<<B/7/F/</7/<//7</BBF<BFB//77B7<B/B<//7<B//7/77B

@GWZHISEQ02:315:C9E6MANXX:5:1311:20397:22565

GGGGAGCAAAAAGCGCGGTTACAGAAGCGAGAAGCGAGCTGACTGGTCAGTTCAAATAAGGCGTGGGGACTTTGCGAGGTGCGCGGGGTACGGGCTACATGGGGAGCGTGTCTTCGAGGCCGGTAA

+

<BBBBB/F</</B/////7<BFF<BFFFF7B/<BFF/F/////</<//BF<B///FF//<F///////</</7///////////<///7<F//<////<<7/7//<7//7B/77</<7///7/7//

@GWZHISEQ02:315:C9E6MANXX:5:1311:11580:86179

AACCAATCAGCTCGCTTCTCGCTTCTGTAACCGCGCTTTTTGCTCCCCAGCCCTATAAAAAGGGTAAAAACCCCACACTCGGCGCGCCAGTCATCCGATAGACTGAGTCGCCCGGGTACCCGTGTT

+

BB<<BBBFFFFFFF<F<<FBFFFF</BFFFFFBFFBF<BFFFFFFBBFF//<BBFFBFF<<F/7FFBFFFFF</<FBF/<FF<BFFBB/B7<B<<FBF<F<<<FBFFFBFF7B<FFBBFBFFFFFF

@GWZHISEQ02:315:C9E6MANXX:5:1316:2081:67946

CCCAAGGAACAGCGAGGCCACGTTTCGGATGCAAACAGCAAAAGGCTTTATTGGGAACACGGGTACCCGGGCGACTCAGTCTATCGGATGACTGGCGCGCCGAGTGTGGGGTTTTTACCCTTTTTA

+

BBBBBFFFFFFFFFFFFFFBFFBFFFFFFFFFFFFFFFFFFFFFFFFFFFFFFFFFFFFFFFFFFFFFFFFFFFFFFFFFFFFFFFFFFFFFFFFFFFFFF<BB<FBBBFF7//7/BFFBBF/7//

@GWZHISEQ02:315:C9E6MANXX:5:1308:10160:27438

GTCCGGCTGGGTTTGAAAGACCCCCGAGCTGGGTAGTCAATCACTCTGAGGAGACCCTCCCAAGGAACAGCGAGGCCACGTTTCGGATGCAAACAGCAAAAGGCTTTATTGGGAACACGGGTACCC

+

BBBBBFFFFFFFFFFFFFFFFFFFFFBFFFFFFFFFFFFFBFFFFFFFFFFFFFFFFFFFFFFFFFFFFFFFBFFFFFFFBFFFFFFFFFFFFFFFFFFFFFFFBFFFFFFFFFFFFFFFFFFFFF

@GWZHISEQ02:315:C9E6MANXX:5:2111:6317:95273

GCAGGTTCTGCCGCCGGGGCAGCGGTGGGTCCTGCTGGGTTAGAAAGACCCCCGAGCTGGGTGGTCAATCACTCTGAGGAGACCCACCCAAGGAACAACGAAGCCACGTTTCGGGTGCAAACAGCA

+

B//B///</<BBB//<//</<///7<</77<F/<FFFFFFF<BFFB//<//////<<//77///7</B/7BF7/7FB////<//7//B//7B//7/7/7///7777BFFF//<///7/BFB///</
