# Supplementary material for: Characterization of a new case of XMLV (Bxv1) contamination in the human cell line Hep2 (clone 2B)
Source: Sci Rep. 2020 Sep 29;10:16046. doi: 10.1038/s41598-020-73169-y (PMC7524804; doi:10.1038/s41598-020-73169-y)
Supplement: Supplementary file 2 — Supplementary Information 2. [file 41598_2020_73169_MOESM2_ESM.docx]

@GWZHISEQ02:315:C9E6MANXX:5:1109:5434:29231

CACCAACTCAAATCAATAGATCCAGAAGAAGTAGAATCGCGTGAATAAAAGATTTTATTCAGTTTCCAGAAAGAGGGGGGAATGAAAGACCCCACCATAAGGCTTAGCAAGCCAGATCGGAAGAGC

+

BBBBBFFFFFFFFFFFFFFFFFFFFFFFFFFFFFFFFFFFFFFFFFFFFFFFFFFFFFFFFFFFFFFFFFFFFFFFFFFFFFFFFFFFFFFFFFFFFFFFFFFFFFFFFFFFFFFFFFFFFFFFFF

@GWZHISEQ02:315:C9E6MANXX:5:1110:4343:34430

CACCAACTCAAATCAATAGATCCAGAAGAAGTAGAATCGCGTGAATAAAAGATTTTATTCAGTTTCCAGAAAGAGGGGGGAATGAAAGACCCCACCATAAGGCTTAGCAAGCTAGCTGCAGTAACG

+

BBBBBFFFFFFFFFFFFFFFFFFFFFFFFFFFFFFFFFFFFFFFFFFFFFFFFFFFFFFFFFFFFFFFFFFFFFFFFFFFFFFFFFFFFFFFFFFFFFFFFFFFFFFFFFFFFFFFFFFFFFFFFF

@GWZHISEQ02:315:C9E6MANXX:5:1203:11007:60104

ATTCAGTTTCCAGAAAGAGGGGGGAATGAAAGACCCCACCATAAGGCTTAGCAAGCTAGCTGCAGTAACGCCATTTTGCAAGGCATGAAAAAGTACCAGAGAGATCGGAAGAGCGTCGTGTAGGGA

+

BBBBBFFFFFFFFFFFFFFFFFFFFFFFFFFFFFFFFBFFFFFFFFFFFFFFFFFFBFFFFFFFFFFFFFFFFFFFFFFFFBFFFFFFFFFFFFFFFFFFFFFFFFFFFFFFFFFF<FFFFFFFBF

@GWZHISEQ02:315:C9E6MANXX:5:1212:3341:43945

TGAAAGACCCCACCATAAGGCTTAGCAAGCTAGCTGCAGTAACGCCATTTTGCAAGGCATGAAAAAGTACCAGAGCTGAGTTCTCAAAAGTCACAAGGAAGTTTAGTTAAAGAATAAGGCTGAACA

+

BBBBBFFFFFFFFFFFFFFFFFFFFFFFFFFFFFFFFFFFFFFFFFFFFFFFFFFFFFFFFFFFFFFFFFFFFFFFFFFFFFFFFFFFFFFFFFFFFFFFFFFFFFFFFFFFFFFFFFFFFFFFFF

@GWZHISEQ02:315:C9E6MANXX:5:1214:14510:59539

CACCAACTCAAATCAATAGATCCAGAAGAAGTAGAATCGCGTGAATAAAAGATTTTATTCAGTTTCCAGAAAGAGGGGGGAATGAAAGACCCCACCATAAGGCTTAGCAAGCTAGCAGATCGGAAG

+

BBBBBFFFFFFFFFFFFFFFFFFFFFFFFFFFFFFFFFFFFFFFFFFFFFFFFFFFFFFFFFFFFFFFFFFFFFFFFFFFFFFFFFFFFFFFFFFFFFFFFFFFFFFFFFFFFFFFFFFFFFFBFB

@GWZHISEQ02:315:C9E6MANXX:5:1302:4425:80534

CACCAACTCAAATCAATAGATCCAGAAGAAGTAGAATCGCGTGAATAAAAGATTTTATTCAGTTTCCAGAAAGAGGGGGGAATGAAAGACCCCACCATAAGGCTTAGCAAGCTAGCTGCAGAGATC

+

BBBBBFFFFFFFFFFFBFF/<FFFFFFB/FFFFFFFFFBFBFFFFFFFFFFFF/<BFFF<FFFFFFFFFFFFFF/FBBBB7FFFFFFFFFFFFFB<FFBFBFFFFFFFBFFFB/BFF<FFFFFFFF

@GWZHISEQ02:315:C9E6MANXX:5:1305:1515:30107

CACCAACTCAAATCAATAGATCCAGAAGAAGTAGAATCGCGTGAATAAAAGATTTTATTCAGTTTCCAGAAAGAGGGGGGAATGAAAGACCCCACCATAAGGCTTAGCAAGCTAGCTGCAGAGATC

+

BBBBBFFFFFFFFFFFFFFFFFFFFFFFFFFFFFFFFFFFFFFFFFFFFFFFFFFFFFFFFFFFFFFFFFFFFFFFFFFFFFFFFFFFFFFFFFFFFFFFFFFFFFFFFFFFFFFFFFFFFFFFFF

@GWZHISEQ02:315:C9E6MANXX:5:1306:12810:7534

CGCGTGAATAAAAGATTTTATTCAGTTTCCAGAAAGAGGGGGGAATGAAAGACCCCACCATAAGGCTTAGCAAGCTAGCTGCAGTAACGCCATTTTGCAAGGCATGAAAAAGAGATCGGAAGAGCG

+

BBBBBFFFFFFFFFFFFFFFFFFFFFFFFFFFFFFFFFFFFFFFFFFFFFFFFFFFFFFFFFFFFFFFFFFFFFFFFFFFFFFFFFFFFFFFFFFFFFFFFFFFFFFFFFFFFFFFFFFFFFFFFF

@GWZHISEQ02:315:C9E6MANXX:5:1308:11979:35371

TGAAAGACCCCACCATAAGGCTTAGCAAGCTAGCTGCAGTAACGCCATTTTGCAAGGCATGAAAAAGTACCAGAGCTGAGTTCTCAAAAGTCACAAGGAAGTTTAGTTAAAGAATAAGGCTGAACA

+

BBBBBFFFFFFFFFFFFBFFFFFFFFFFFFFFFFFFFFFFFFFFBBBFFFFFFFFFFFFFFFFFFFFFFFFFFBFFFFBFFFFFFFFFFFFFFFFFFFFFFFFFFFFFFFFFFFFFFFFFFFFFFF

@GWZHISEQ02:315:C9E6MANXX:5:1308:10284:67022

CGCGTGAATAAAAGATTTTATTCAGTTTCCAGAAAGAGGGGGGAATGAAAGACCCCACCATAAGGCTTAGCAAGCTAGCTGCAGTAACGCCATTTTGCAAGGCATGAAAAAGTACCAGAGAGATCG

+

BBBBBFFFFFFFFFFFFFFFFFFFFFFFFFFFFFFFFFFFFFFFFFFFFFFFFFFFFFFFFFFFFFFFFFFFFFFFFFFFFFFFFFFFFFFFFFFFFFFFFFFFFFFFFFFFFFFFFFFFFFFFFF

@GWZHISEQ02:315:C9E6MANXX:5:1311:1312:67347

TGAAAGACCCCACCATAAGGCTTAGCAAGCTAGCTGCAGTAACGCCATTTTGCAAGGCATGAAAAAGTACCAGAGCTGAGTTCTCAAAAGTCACAAGGAAGTTTAGTTAAAGAATAAGGCTGAACA

+

BBBBBFFFFFFFFFFFFFFFFFFFFFFFFFFFFFFFFFFFFFFFFFFFFFFFFFFFFFFFFFFFFFFFFFFFFFFFFFFFFFFFFFFFFFFFFFFFFFFFFFFFFFFFFFBFFFFFFFFFBFFFFB

@GWZHISEQ02:315:C9E6MANXX:5:1312:3225:33753

TGAAAGACCCCACCATAAGGCTTAGCAAGCTAGCTGCAGTAACGCCATTTTGCAAGGCATGAAAAAGTACCAGAGCTGAGTTCTCAAAAGTCACAAGGAAGTTTAGTTAAAGAATAAGGCTGAACA

+

BBBBBFFFFFFFFFFFFFFFFFFFFFFFFFFFFFFFFFFFFFFFFFFFFFFFFFFFFF<FFFFFF<BFFFFFFFFFFFFFFFFFFFFFFFFFFFFFFFFFFFFFFFFFFFFFFFFFFFF<FFFFFF

@GWZHISEQ02:315:C9E6MANXX:5:1315:8669:57917

CACCAACTCAAATCAATAGATCCAGAAGAAGTAGAATCGCGTGAATAAAAGATTTTATTCAGTTTCCAGAAAGAGGGGGGAATGAAAGACCCCACCATAAGGCTTAGCAAGCTAGCTGCAGTAACG

+

</BB/FFFFFFBFFFBFFF/<//<///</<<B/<//<<<<FFFFFFFFFFFFBBFBF</BFFF/</<FF<FFFFBFBF/FB<F/BFFBF/BFF<B<<7////77BB<F<//BBFBBB/FFFBFFF/

@GWZHISEQ02:315:C9E6MANXX:5:2104:13610:51511

CGCGTGAATAAAAGATTTTATTCAGTTTCCAGAAAGAGGGGGGAATGAAAGACCCCACCATAAGGCTTAGCAAGCTAGCTGCAGTAACGCCATTTTGCAAGGCATGAAAAAGTACCAGAGAGATCG

+

BBBBBFFFFFFFFFFFFFFFFFFFFFFFFFFFFFFFFFFFFFFFFFFFFFFFFFFFFFFFFFFFFFFFFFFFFFFFFFFFFFFFFFFFFFFFFFFFFFFFFFFFFFFFFFFFFFFFFFFFFFFFFF

@GWZHISEQ02:315:C9E6MANXX:5:2104:15953:53584

TGAAAGACCCCACCATAAGGCTTAGCAAGCTAGCTGCAGTAACGCCATTTTGCAAGGCATGAAAAAGTACCAGAGCTGAGTTCTCGAAAGTCACAAGGAAGTTTAGTTAAAGAATAAGGCTGAACA

+

BBBBBFFFFFFFFFFFFFFFFFFFFFFFFFFFFFFFFFFFFFFFFFFFFFFFFFFFFFFFFFFFFFFFFFFFFFFFFFFFFFFFF/<FFFFFFFFFFFFFFFFFFFFFFFFFFFFFFFFFFFFFFF

@GWZHISEQ02:315:C9E6MANXX:5:2104:16459:78969

CGCGTGAATAAAAGATTTTATTCAGTTTCCAGAAAGAGGGGGGAATGAAAGACCCCACCATAAGGCTTAGCAAGCTAGCTGCAGTAACGCCATTTTGCAAGGCATGAGAGATCGGAAGAGCGTCGT

+

BBBBBFFFFFFFFFFFFFFFFFFFFFFFFFFFFFFFFFFFFFFFFFFFFFFFFFFFFFFFFFFFFFFFFFFFFFFFFFFFFFFFFFFFFFFFFFFFFFFFFFFFFFFFFFFFFFFFFFFFFFFFF7

@GWZHISEQ02:315:C9E6MANXX:5:2112:11545:80339

ATTCAGTTTCCAGAAAGAGGGGGGAATGAAAGACCCCACCATAAGGCTTAGCAAGCTAGCTGCAGTAACGCCATTTTGCAAGGCATGAAAAAGTACCAGAGCTGAGAGATCGGAAGAGCGTCGTGT

+

BBBBBFFFFFFFFFFFFFFFFFFFFFFFFFFFFFFFFFFFFFFFFFFFFFFFFFFFFFFFFFFFFFFFFFFFFFFFFFFFFFFFFFFFFFFFFFFFFFFFFFFFFFFFFFFFFFFFFFFFBFFBFB

@GWZHISEQ02:315:C9E6MANXX:5:2113:5627:43945

TGAAAGACCCCACCATAAGGCTTAGCAAGCTAGCTGCAGTAACGCCATTTTGCAAGGCATGAAAAAGTACCAGAGCTGAGTTCTCAAAAGTCACAAGGAAGTTTAGTTAAAGAATAAGGCTGAACA

+

BBBBBFFFFFFFFFFFFFFFFFFFFFFFFFFFFFFFFFFFFFFFFFFFFFFFFFFFFFFFFFFFFFFFFFFFFFFFFFFFFFFFFFFFFFFFFFFFFFFFFFFFFFFFFFFFFFFFFFFFFFFFFF

@GWZHISEQ02:315:C9E6MANXX:5:2202:13422:62213

TGAAAGACCCCACCATAAGGCTTAGCAAGCTAGCTGCAGTAACGCCATTTTGCAAGGCATGAAAAAGTACCAGAGCTGAGTTCTCAAAAGTCACAAGGAAGTTTAGTTAAAGAATAAGGCTGAACA

+

BBBBBFFFFFFFFFFFFFFFFFFFFFFFFFFFFFFFFFFFFFFFFFFFFFFFFFFFFFFFFFFFFFFFFFFFFFFFFFFFFFFFFFFFFFFFFFFFFFFFFFFFFFFFFFFFFFFFFFFFFFFFFF

@GWZHISEQ02:315:C9E6MANXX:5:2206:19098:93497

CGTGAATAAAAGATTTTATTCAGTTTCCAGAAAGAGGGGGGAATGAAAGACCCCACCATAAGGCTTAGCAAGCTAGCTGCAGTAACGCCATTTTGCAAGGCATGAAAAAGTACCAGAGAGATCGGA

+

BBBBBFFFFFFFFFFFFFFFFFFFFFFFFFFFBFFFFFFFFBFFFFFFFFFFFFFFFFFFFFFFFFFFFFFFFFFFFFFFFFFFFFBFFFFBBFFFFFFFFFFFFFFFFFFFFFFFFFFFFFFFBB

@GWZHISEQ02:315:C9E6MANXX:5:2209:17996:26852

TGAAAGACCCCACCATAAGGCTTAGCAAGCTAGCTGCAGTAACGCCATTTTGCAAGGCATGAAAAAGTACCAGAGCTGAGTTCTCAAAAGTCACAAGGAAGTTTAGTTAAAGAATAAGGCTGAACA

+

BBBBBFFF/BFFFFFFFFFFFFFFFFFFFFFFFFFFFFFFFFFFFFFFFFFFFFFFFFFFFFFFFFFFFFFFFFFFFFFF<BFFFFFFFFFFFFFFFFFFFBFFFFFFFFFFFFFFFFFFFFFFFF

@GWZHISEQ02:315:C9E6MANXX:5:2209:5230:50506

CCAGAAGAAGTAGAATCGCGTGAATAAAAGATTTTATTCAGTTTCCAGAAAGAGGGGGGAATGAAAGACCCCACCATAAGGCTTAGCAAGCTAGCTGCAGAGATCGGAAGAGCGTCGTGTAGGGAA

+

BBBBBFBFFFBBFFFFFFFFFBFFFFFFBF<FFFFFFFFFFFFF<B<F/FFFFFFF/FFF/<FFFFBFFFFFFFFFFF<BFFFFFFFFFFFFFFFFF</F<FFFF<BFFFFFFF7BF/BFFFFFBB

@GWZHISEQ02:315:C9E6MANXX:5:2212:6418:14756

TGAAAGACCCCACCATAAGGCTTAGCAAGCTAGCTGCAGTAACGCCATTTTGCAAGGCATGAAAAAGTACCAGAGCTGAGTTCTCAAAAGTCACAAGGAAGTTTAGTTAAAGAATAAGGCTGAACA

+

BBBBBFFFFFFFFFFFFFFFFFFFFFFFFFFFFFFFFFFFFFFFFFFFFFFFFFFFFFFFFFFFFFFFFFFFFFFFFFFFFFFFFFFFFFFFFFFFFFFFFFFFFFFFFFFFFFFFFFFFFFFFFF

@GWZHISEQ02:315:C9E6MANXX:5:2212:3661:72873

TGAAAGACCCCACCATAAGGCTTAGCAAGCTAGCTGCAGTAACGCCATTTTGCAAGGCATGAAAAAGTACCAGAGTTGAGTTTTCAAAAGTAACAAGGAAGTTTAGTTAAAGAATAAGCCTGAACA

+

B/<B<//<<<F<B///<FBBFBF//<FB//<<</<///</</BB//<BFFB/<</FB<F/<BFFF/7/<<BB/<//</</<//////<//<///<<F///7<B/</</</BF/<///<//</7/7B

@GWZHISEQ02:315:C9E6MANXX:5:2213:19893:65129

CACCAACTCAAATCAATAGATCCAGAAGAAGTAGAATCGCGTGAATAAAAGATTTTATTCAGTTTCCAGAAAGAGGGGGGAATGAAAGACCCCACCATAAGGCTTAGCAAGCTAGCTGCAGAGATC

+

BBBBBFBFFFFFFFFFFFFBFFFFFFFB/FFFFF<FFFFFFFFFFFFFFFFBBFFFFFFFFFFFFFFFFFFFFFFFFBFF7BF<FBFFFFBBB<FFBFF<BFFFFFFFFFFFF/BFFFFBFFFBB/

@GWZHISEQ02:315:C9E6MANXX:5:2301:4293:69538

TGAAAGACCCCACCATAAGGCTTAGCAAGCTAGCTGCAGTAACGCCATTTTGCAAGGCATGAAAAAGTACCAGAGCTGAGTTCTCAAAAGTCACAAGGAAGTTTAGTTAAAGAATAAGGCTGAACA

+

BBBBBFFFFFFFFFFFFFFFFFFFFFFFFFFFFFFFFFFFFFFFFFFFFFFFFFFFFFFFFFFFFFFFFFFFFFFFFFFFFFFFFFFFFFFFFFFFFFFFFFFFFFFFFFFFFFFFFFFFFFFFFF

@GWZHISEQ02:315:C9E6MANXX:5:2302:5717:80903

TGAAAGACCCCACCATAAGGCTTAGCAAGCTAGCTGCAGTAACGCCATTTTGCAAGGCATGAAAAAGTACCAGAGCTGAGTTCTCAAAAGTCACAAGGAAGTTTAGTTAAAGAATAAGGCTGAACA

+

/</BBFFF/</FBFFFF/BBFFFFFFFF//FBFFF/FBFFFFF<B<<BBBFFFFFF<FFFFFFFFFFFFFFFFFFFBFBFFFF/FFFFBFFFFBFBFFFFFF/FFFFFFFFFFFFFFF/BF/FBFF

@GWZHISEQ02:315:C9E6MANXX:5:2306:3553:97334

AGAAGAAGTAGAATCGCGTGAATAAAAGATTTTATTCAGTTTCCAGAAAGAGGGGGGAATGAAAGACCCCACCATAAGGCTTAGCAAGCTAGCTGCAGTAACGCCATTTTGCAAGGCATGGAGATC

+

BBBBBFFFFFFFFFFFFFFFFFFFFFFFFFFFFFFFFFFFFFFFFFFFFFFFFFFFFFFFFFFFFFFBFFFFFFFFFFFFFFFFFFFFFFFFFFFFFFFFFFFFFFFFFFFFFFFFFFFFFFFFFF

@GWZHISEQ02:315:C9E6MANXX:5:2307:21320:66058

TNAAAGACCCCACCATAAGGCTTAGCAAGCTAGCTGCAGTAACGCCATTTTGCAAGGCATGAAAAAGTACCAGAGCTGATTGCGNGNNNNNGACAAGGAATTTNNNNTAAAGAANANGNCTGAACA

+

B#<<<BFF</BFFFFFFFFFFFFFFBFFB/FBFFFF/FFFFFFF</FFFFFFBFFFF<BFFFFF/<BFFF/FFBF/FFF<<</<#/#####//<<FFFF/</<####/<7<BB/#/#<#7777BFF

@GWZHISEQ02:315:C9E6MANXX:5:2311:1716:43090

AAAAGATTTTATTCAGTTTCCAGAAAGAGGGGGGAATGAAAGACCCCACCATAAGGCTTAGCAAGCTAGCTGCAGTAACGCCATTTTGCAAGGCATGAAAAAGAGATCGGAAGAGCGTCGTGTAGG

+

BBBBBFFFFFFFFFFFFFFFFFFFFFFFFFFFFFFFFFFFFFFFFFFFFFFFFFFFFFFFFFFFFFFFFFFFFFFFFFFFFFFFFFFFFFFFFFFFFFFFFFFFFFFFFFFFFFFFFFFFBFFBFF

@GWZHISEQ02:315:C9E6MANXX:5:2314:13410:83148

TGAAAGACCCCACCATAAGGCTTAGCAAGCTAGCTGCAGTAACGCCATTTTGCAAGGCATGAAAAAGTACCAGAGCTGAGTTCTCAAAAGTCACAAGGAAGTTTAGTTAAAGAATAAGGCTGAACA

+

BBBBBFFFFF<FBBFFFFFFFFFFFFFFFFFFFFFFFFFFFFFFFFFFFFFFFFFFFFFFFFFFFFFFFFFFFFFFBFFFFFFFFFFFFFFFFFFFFFFFFFFFFFFFFFFFFFFFBFFF<FFFFF

@GWZHISEQ02:315:C9E6MANXX:5:1109:5434:29231

GGCTTGCTAAGCCTTATGGTGGGGTCTTTCATTCCCCCCTCTTTCTGGAAACTGAATAAAATCTTTTATTCACGCGATTCTACTTCTTCTGGATCTATTGATTTGAGTTGGTGAGATCGGAAGAGC

+

<BBBBFFFFFFFFFFFFFFFFFFFFFFFFFFFFFFFFFFFFFFFFFFFFFFFFFFFFFFFFFFFFFFFFFFFFFFFFFFFFFFFFFFFFFFFBFFFFFFFFFFFFFFFFFFFFFFFFFBFFFFFFF

@GWZHISEQ02:315:C9E6MANXX:5:1203:11007:60104

CTCTGGTACTTTTTCATGCCTTGCAAAATGGCGTTACTGCAGCTAGCTTGCTAAGCCTTATGGTGGGGTCTTTCATTCCCCCCTCTTTCTGGAAACTGAATAGATCGGAAGAGCACACGTCTGAAC

+

BBBBBFFFFFFFFFFFFFFFFFFFFFFFFFFFFB<FFFFFFFFFFFFFFFFFFFFFFFFFFFFFFFFFFFFFFFBF<FFFFFFFFFFFFFBFFFFFFFFFFFFFFBFFFFFFFFFF<FFFFFFFFF

@GWZHISEQ02:315:C9E6MANXX:5:1214:14510:59539

GCTAGCTTGCTAAGCCTTATGGTGGGGTCTTTCATTCCCCCCTCTTTCTGGAAACTGAATAAAATCTTTTATTCACGCGATTCTACTTCTTCTGGATCTATTGATTTGAGTTGGTGAGATCGGAAG

+

BBBBBFFFFFFFFFFFFFFFF<FFFFFFFFFFFFFFFFFFFFFFFFFFFFFFFFFFFFFFFFFFFFFFFFFFFFFFFFFFFFFFFFFFFFFFFFFFFFBFFFFFFFFBFFFFFBFFFBFFFBBFFF

@GWZHISEQ02:315:C9E6MANXX:5:1302:4425:80534

CTGCAGCTAGCTTGCTAAGCCTTATGGTGGGGTCTTTCATTCCCCCCTCTTTCTGGAAACTGAATAAAATCTTTTATTCACGCGATTCTACTTCTTCTGGATCTATTGATTTGAGTTGGTGAGATC

+

BB/BBFFFFFFFFFFFBFFFFFFFFBFFFFFBF<FFFBFFFFFFFFFFFFFFFFFFFFFFFFFFFF<F<FFFFFF<FFFFF<FFFFFFFFFFFFFFFFB/<BBBFFFFFFBFFBBFFF<FFFFFF/

@GWZHISEQ02:315:C9E6MANXX:5:1305:1515:30107

CTGCAGCTAGCTTGCTAAGCCTTATGGTGGGGTCTTTCATTCCCCCCTCTTTCTGGAAACTGAATAAAATCTTTTATTCACGCGATTCTACTTCTTCTGGATCTATTGATTTGAGTTGGTGAGATC

+

BBBBBFFFFFFFFFFFFFFFFFFFFFFFFFFFFFFFFFFFFFFFFFFFFFFFFFFFFFFFFFFFFFFFFFFFFFFFFFFFFFFF<FFFFFFFFFFFFFFFFFFFFFFFFFFFFFFFFFFFFFFFF<

@GWZHISEQ02:315:C9E6MANXX:5:1306:12810:7534

CTTTTTCATGCCTTGCAAAATGGCGTTACTGCAGCTAGCTTGCTAAGCCTTATGGTGGGGTCTTTCATTCCCCCCTCTTTCTGGAAACTGAATAAAATCTTTTATTCACGCGAGATCGGAAGAGCA

+

BBBBBFFFFFFFFFFFFFFFFFFFFFFFFFFFFFFFFFFFFFFFFFFFFFFFFFFFFFFFFFFFFFFFFFFFFFFFFFFFFFFFFFFFFFFFFFFFFFFFFFFFFFFFFFFFFFFFFFFFFFFFFF

@GWZHISEQ02:315:C9E6MANXX:5:1308:10284:67022

CTCTGGTACTTTTTCATGCCTTGCAAAATGGCGTTACTGCAGCTAGCTTGCTAAGCCTTATGGTGGGGTCTTTCATTCCCCCCTCTTTCTGGAAACTGAATAAAATCTTTTATTCACGCGAGATCG

+

BBBBBFFFFFFFFFFFFFFFFFFFFFFFFFFFFFFFFFFFFFFFFFFFFFFFFFFFFFFFFFFFFFFFFFFFFFFFFFFFFFFFFFFFFFFFFFFFFFFFFFFFFFFFFFFFFFFFFFFFBFFFFF

@GWZHISEQ02:315:C9E6MANXX:5:2104:13610:51511

CTCTGGTACTTTTTCATGCCTTGCAAAATGGCGTTACTGCAGCTAGCTTGCTAAGCCTTATGGTGGGGTCTTTCATTCCCCCCTCTTTCTGGAAACTGAATAAAATCTTTTATTCACGCGAGATCG

+

BBBBBFFFFFFFFFFFFFFFFFFFFFFFFFFFFFFFFFFFFFFFFFFFFFFFFFFFFFFFFFFFFFFFFFFFFFFFFFFFFFFFFFFFFFFFFFFFFFFFFFFFFFFFFFFFFFFFFFFFFFFFFF

@GWZHISEQ02:315:C9E6MANXX:5:2104:15953:53584

GTTCAGCCTTATTCTTTAACTAAACTTCCTTGTGACTTTTGAGAACTCAGCTCTGGTACTTTTTCATGCCTTGCAAAATGGCGTTACTGCAGCTAGCTTGCTAAGCCTTATGGTGGGGTCTTTCAA

+

BBBBBFFFFFFFFFFFFFFFFFFFFFFFFFFFFFFFFFF/FFFFFFFFFFFFFFFFFFFFFFFFFFFFFFFFFFFFFFFFFFFFFFFFFFFFFFFFFFFFFFFFFFFFFFFFFFFFFFFFFFFFFF

@GWZHISEQ02:315:C9E6MANXX:5:2104:16459:78969

CTCATGCCTTGCAAAATGGCGTTACTGCAGCTAGCTTGCTAAGCCTTATGGTGGGGTCTTTCATTCCCCCCTCTTTCTGGAAACTGAATAAAATCTTTTATTCACGCGAGATCGGAAGAGCACACG

+

BBBBBFFFFFFFFFFFFFFFFFFFFFFFFFFFFFFFFFFFFFFFFFFFFFFFFFFFFFFFFFFFFFFFFFFFFFFFFFFFFFFFFFFFFFFFFFFFFFFFFFFFFFFFFFFFFFFFFFFFFFFFF/

@GWZHISEQ02:315:C9E6MANXX:5:2109:20812:5928

CCTTATTCTTTAACTAAACTTCCTTGTGACTTTTGAGAACTCAGCTCTGGTACTTTTTCATGCCTTGCAAAATGGCGTTACTGCAGCTAGCTTGCTAAGCCTTATGGTGGGGTCTTTCAGATCGGA

+

/B<<<FFFFFFFFFFF<<BFFFFFFFF<FFFFFFF///<FBFFFFFFFFFB<FFFFFFF/FBFFFFFFFFF<FF<BBBFFFFFFFFFFFFFFFFFBFFFF/<F///B<<<B/BFFFFB7FFFFBF/

@GWZHISEQ02:315:C9E6MANXX:5:2112:11545:80339

CTCAGCTCTGGTACTTTTTCATGCCTTGCAAAATGGCGTTACTGCAGCTAGCTTGCTAAGCCTTATGGTGGGGTCTTTCATTCCCCCCTCTTTCTGGAAACTGAATAGATCGGAAGAGCACACGTC

+

BBBBBFFFFFFFFFFFFFFFFFFFFFFFFFFFFFFFFFFFFFFFFFFFFFFFFFFFFFFFFFFFFFF<FFFFFFFFFFFFFFFFFFFFFFFFFFFFFFFFFFFFFFFFFFFFFFFFFFFBFFFFFB

@GWZHISEQ02:315:C9E6MANXX:5:2206:19098:93497

CTCTGGTACTTTTTCATGCCTTGCAAAATGGCGTTACTGCAGCTAGCTTGCTAAGCCTTATGGTGGGGTCTTTCATTCCCCCCTCTTTCTGGAAACTGAATAAAATCTTTTATTCACGAGATCGGA

+

/BBBBFFFFFFFFFFFFFFFFFFFFFFFFFFFFFFFFFFFFFFFBBFFFFFFFFFFFFFFFFBFFFFFFFFFFFBFFFFFFFFBFBFFFFFFBFBBFF7BBFFFFFFFFFFFFFFBFFFFFFF<B<

@GWZHISEQ02:315:C9E6MANXX:5:2209:5230:50506

CTGCAGCTAGCTTGCTAAGCCTTATGGTGGGGTCTTTCATTCCCCCCTCTTTCTGGAAACTGAATAAAATCTTTTATTCACGCGATTCTACTTCTTCTGGAGATCGGAAGAGCACACGTCTGAACT

+

BBBBBFFFFFFFFFFFFF<FFFFF/FFFFFFFFFFFFBFBF<B/BFBFBBFFFFFFFFFFFF<FFF/FFFFFFFFFFFF/FFFFF<B<B/F/FFFFFFFFFFBFFBB/B7FFFFFFFB77FF<FFF

@GWZHISEQ02:315:C9E6MANXX:5:2213:19893:65129

CTGCAGCTAGCTTGCTAAGCCTTATGGTGGGGTCTTTCATTCCCCCCTCTTTCTGGAAACTGAATAAAATCTTTTATTCACGCGATTCTACTTCTTCTGGATCTATTGATTTGAGTTGGTGAGATC

+

BBBBBFFFFFFFFFBFFFFFFFF/BFFFFBBFFFFFFF/BFFFBFBFFBFFFFFFFBF<FFF<BF/BFFFFFFFFFFF/FFBFB/<FFFFFFFBFFFFF//BBFBFBBFFFFF/BF<F<FFF7FF/

@GWZHISEQ02:315:C9E6MANXX:5:2306:3553:97334

CCATGCCTTGCAAAATGGCGTTACTGCAGCTAGCTTGCTAAGCCTTATGGTGGGGTCTTTCATTCCCCCCTCTTTCTGGAAACTGAATAAAATCTTTTATTCACGCGATTCTACTTCTTCTAGATC

+

BBBBBFFFFFFFFFFFFFFFFFFFFFFFFFFFFFFFFFFFFFFFFFFFFFFFFFFFFFFFFFFFFFFFFFFFFFFFFFFFFFFFFFFFFFFFFFFFFFFFFFFFFFFFFFFFFFFFFFFFF<FBFF

@GWZHISEQ02:315:C9E6MANXX:5:2311:1716:43090

CTTTTTCATGCCTTGCAAAATGGCGTTACTGCAGCTAGCTTGCTAAGCCTTATGGTGGGGTCTTTCATTCCCCCCTCTTTCTGGAAACTGAATAAAATCTTTTAGATCGGAAGAGCACACGTCTGA

+

BBBBBFFFFFFFFFFFFFFFFFFFFFFFFFFFFFFFFFFFFFFFFFFFFFFFFFBFFFFFFFFFFFFFFFFFFFFFFFFFFFFFFFFFFFFFFFFFFFFFFFFFFF<FFFFFFFFFFFFFFFFFFB

@GWZHISEQ02:315:C9E6MANXX:5:1103:12691:55842

GAAAGACCCCACCATAAGGCTTAGCAAGCTAGCTGCAGTAACGCCATTTTGCAAGGCATGAAAAAGTACCAGAGCTGAGTTCTCAAAAGTCACAAGGAAGTTTAGTTAAAGAATAAGGCTGAACAA

+

BBBBBFFFBBFFBFFFFFFFFFFBFFFFFFFFFFFFFFFFFFBBBFFFFFBFBFBF<BBFFBFFF7FFFFFFBFFFBFB<FFFFFFFFFFFFFFFFFF/BFFFFFFFFFFFFBFFFFFFBFFFFFF

@GWZHISEQ02:315:C9E6MANXX:5:1113:12931:97548

GAAAGACCCCACCATAAGGCGTAGCAAGCTAGCTGCAGTAACGCCATTTTGCAAGGCATGAAAAAGTACCAGAGCTGAGTTCTCAAAAGTCACAAGGAAGTTTAGTTAAAGAATAAGGCTGAACAA

+

BBBBBFFFFFFFFFFFFFFFFFFFFFFFFFFFFFFFFFFFFFFFFFFFFFFFFFFFFFFFFFFFFFFFBBBFFFFFF<FFFFFFFFFFFFFFFFFFFFFFFFFFFFFFFFFFF/FFFFFFFFFFFF

@GWZHISEQ02:315:C9E6MANXX:5:1209:10562:24916

GAAAGACCCCACCATAAGGCTTAGCAAGCTAGCTGCAGTAACGCCATTTTGCAAGGCATGAAAAAGTACCAGAGCTGAGTTCTCAAAAGTCACAAGGAAGTTTAGTTAAAGAATAAGGCTGAACAA

+

BBBBBFFFFFFFFFFFFFFFFFFFFFFFFFFFFFFFFFFFFFFFFFFFFFFFFFFBFFFFFFFFFFFFFFFFFFFFFFFFFFFFFFFFFFFBFFFFFFFFFFFFFFFFFFFFFFFBFFFFFBFFFF

@GWZHISEQ02:315:C9E6MANXX:5:1211:6079:37674

GAAAGACCCCACCATAAGGCTTAGCAAGCTAGCTGCAGTAACGCCATTTTGCAAGGCATGAAAAAGTACCAGAGCTGAGTTCTCAAAAGTCACAAGGAAGGTTAGTTAAATAATAAGGCTGAACAA

+

BBBBBBFFFFFFFFFFFFFFFFFFFFBFFFFFFFF<FFFFFFBFFFFFFFFBFFFFFFFFFBB/<BFB/<F/FFF</</F/F//<F/<<</<<F//BF///FF/BFFFBB///<///////<B/FF

@GWZHISEQ02:315:C9E6MANXX:5:1304:20199:81624

GAAAGACCCCACCATAAGGCTTAGCAAGCTAGCTGCAGTAACGCCATTTTGCAAGGCATGAAAAAGTACCAGAGCTGAGTTCTCAAAAGTCACAAGGAAGTTTAGTTAAAGAATAAGGCTGAACAA

+

BBBBBFFFFFFFFFFFFFFFFFFFFFFFFFFFFFFFFFFFFFFFFFFFFFFFFFFFFFFFFFFFFFFFFFFFFFFFFFFFFFFFFFFFFFFFFFFFFFFFFFFFFFFFFFFFFFFFFFFFFFFFFF

@GWZHISEQ02:315:C9E6MANXX:5:1304:14364:88317

GAAAGACCCCACCATAAGGCTTAGCAAGCTAGCTGCAGTAACGCCATTTTGCAAGGCATGAAAAAGTACCAGAGCTGAGTTCTCAAAAGTCACAAGGAAGTTTAGTTAAAGAATAAGGCTGAACAA

+

BBBBBFFFFFFFFFFFFFFFFFFFFFFFFFFFFFFFFFFFFFFFFFFFFFFFFFFFFFFFFFFFFFFFFFFFFFFFFFFFFFFFFFFFFFFFFFFFFFFFFFFFFFFFFFFFFFFFFFFFFFFFFF

@GWZHISEQ02:315:C9E6MANXX:5:1310:7666:73426

GAAAGACCCCACCATAAGGCTTAGCAAGCTAGCTGCAGTAACGCCATTTTGCAAGGCATGAAAAAGTACCAGAGCTGAGTTCTCAAAAGTCACAAGGAAGTTTAGTTAAAGAATAAGGCTGAACAA

+

BBBBBFFFFFFFFFFFFFFFFFFFFFFFFFFFFFFFFFFFFFFFFFFFFFFFFFFFFFFFFBFFFFFFFFFFFFFFFFFFFFFFFFFFFFFFFFFFFFFFFFFFFFFFFFFFFFFFFFFFFFFFFF

@GWZHISEQ02:315:C9E6MANXX:5:1311:13567:66012

GAAAGACCCCACCATAAGGCTTAGCAAGCTAGCTGCAGTAACGCCATTTTGCAAGGCATGAAAAAGTACCAGAGCTGAGTTCTCAAAAGTCACAAGGAAGTTTAGTTAAAGAATAAGGCTGAACAA

+

BBBBBFFFFFFFFFFFFFFFFFFFFFFFFFFFFFFFFFFFFFFFFFFFFFFFFFFFFFFFFFFFFFFFFFFFFFFFFFFFFFFFFFFFFFFFFFFFFFFFFFFFFFFFFFFFFFFFFFFFFFFFFF

@GWZHISEQ02:315:C9E6MANXX:5:2104:7050:93720

GAAAGACCCCACCATAAGGCTTAGCAAGCTAGCTGCAGTAACGCCATTTTGCAAGGCATGAAAAAGTACCAGAGCTGAGTTCTCAAAAGTCACAAGGAAGTTTAGTTAAAGAATAAGGCTGAACAA

+

BBBBBFFFFFFFFFFFFFFFFFFFFFFFFFFFFFFFFFFFFFFFFFFFFFFFFFFFFFFFFFFFFFFFFFFFFFFFFFFFFFFFFFFFFFFFFFFFFFFFFFFFFFFFFFFFFFFFFFFFFFFFFF

@GWZHISEQ02:315:C9E6MANXX:5:2210:17763:6131

GAAAGACCCCACCATAAGGCTTAGCAAGCTAGCTGCAGTAACGCCATTTTGCAAGGCATGAAAAAGTACCAGAGCTGAGTTCTCAAAAGTCACAAGGAAGTTTAGTTAAAGAATAAGGCTGAACAA

+

BBBBBFFFFFFFFFFFFFFFFFFFFFFFFFFFFFFFFFFFFFFFFFFFFFFFFFFFFFFBFFFFFFFFFFFFFFFFFFFFFF<FFFFFFFFFFFFFFFFFFFFFFFFFFFFFFFFFFFFFFFFFFF

@GWZHISEQ02:315:C9E6MANXX:5:2215:10434:22235

GAAAGACCCCACCATAAGGCTTAGCAAGCTAGCTGCAGTAACGCCATTTTGCAAGGCATGAAAAAGTACCAGAGCTGAGTTCTCAAAAGTCACAAGGAAGTTTAGTTAAAGAATAAGGCTGAACAA

+

BBBBBFFFFFFFFFFFFFFFFFFFFFFFFFFFFFFFFFFFFFFFFFFFFFFFFFFFFFFFFFFFFFFFFFFFFFFFBFFFFFFFFFFFFFFFFFFFFFFFFFFFFFFFFFFFFFFFFFFFFFFFFF

@GWZHISEQ02:315:C9E6MANXX:5:2313:18929:50517

GAAAGACCCCACCATAAGGCTTAGCAAGCTAGCTGCAGTAACGCCATTTTGCAAGGCATGAAAAAGTACCAGAGCTGAGTTCTCAAAAGTCACAAGGAAGTTTAGTTAAAGAATAAGGCTGAACAA

+

BBBBBFFFFFFFFFFFFFFFFFFFFFFFFFFFFFFFFFBFFFFFFFFFFFFFFFFFFFFFFFFFFFFFFFFFFFFFFFFFFFFBFFFFFFFFFFFFFFFFFFFFFFFFFFFFFFFFFFFFFFFFFF

@GWZHISEQ02:315:C9E6MANXX:5:1202:10052:71133

AAAGACCCCACCATAAGGCTTAGCAAGCTAGCTGCAGTAACGCCATTTTGCAAGGCATGAAAAAGTACCAGAGCTGAGTTCTCAAAAGTCACAAGGAAGTTTAGTTAAAGAATAAGGCTGAACAAA

+

BBBBBFFFFFFFFFFFFFFFFFFFFFFFFFFFFFFFFFFFFFFBFFFFFFFFFFFFFFFFFFFFFFFFFFFFFFFFFFFFFFFFFFFFFFFFFFFFFFFFFFFFFFFFFFFFFFFFFFFFFFFFFF

@GWZHISEQ02:315:C9E6MANXX:5:1314:9836:26902

AAAGACCCCACCATAAGGCTTAGCAAGCTAGCTGCAGTAACGCCATTTTGCAAGGCATGAAAAAGTACCAGAGCTGAGTTCTCAAAAGTCACAAGGAAGTTTAGTTAAAGAATAAGGCTGAACAAA

+

BBBBBFFFFFFFFFFFFFFFFFFFFFFFFFFFFFFFFFFFFFFFFFFFFFFFFFFFFFFFFFFFFFFFFFFFFFFFFFFFFFFFFFFFFFFFFFFFFFFFBFFFFFFFFFFFFFFFFFFFFFFFFF

@GWZHISEQ02:315:C9E6MANXX:5:2210:8561:31456

GACCCCACCATAAGGCTTAGCAAGCTAGCTGCAGTAACGCCATTTTGCAAGGCATGAAAAAGTACCAGAGCTGAGTTCTCAAAAGTCACAAGGAAGTTTAGTTAAAGAATAAGGCTGAACAAAACT

+

BBBBBFFFFFFFFFFFFFFFFFFFFFFFFFFFFFFFFFFFFFFFFFFFFFFFFFFBFBFFFFFFFFFFFFFFFFFFFFFFFFFFFFFFFFFFFFFFFFFFFFFFFFFFFFBBFFFFFFFFFFFFFF

@GWZHISEQ02:315:C9E6MANXX:5:1108:10953:21747

CCCCACCATAAGGCTTAGCAAGCTAGCTGCAGTAACGCCATTTTGCAAGGCATGAAAAAGTACCAGAGCTGAGTTCTCAAAAGTCACAAGGAAGTTTAGTTAAAGAATAAGGCTGAACAAAACTGG

+

BBBBBFFFFFFFFFFFFFFFFFFFFFFFFFFFFFFFFFFFFFFFFFFFFFFFFFFFFFFFFFFFFFFFFFFFFFFFFFFFFFFFFFFFFFFFFFFFFFFFFFFFFFFFFFFFFFFFFFFFFFFFFF

@GWZHISEQ02:315:C9E6MANXX:5:1111:9159:93783

CCCCACCATAAGGCTTAGCAAGCTAGCTGCAGTAACGCCATTTTGCAAGGCATGAAAAAGTACCAGAGCTGAGTTCTCAAAAGTCACAAGGAAGTTTAGTTAAAGAATAAGGCTGAACAAAACTGG

+

BBBBBFFFFFFFFFFFFFFFFFFFFFFFFFFFFFBFFFFFFFFFFFFFFFFFFFFFFFFFFFFFFFFFFFFFF<FFFFFFFFFFFFFFFFFFFFFFFFFFFFFFFFFFFFFFFFFBFFFFFFFFFF

@GWZHISEQ02:315:C9E6MANXX:5:1112:11136:23530

CCCCACCATAAGGCTTAGCAAGCTAGCTGCAGTAACGCCATTTTGCAAGGCATGAAAAAGTACCAGAGCTGAGTTCTCAAAAGTCACAAGGAAGTTTAGTTAAAGAAATAAGGCTGAACAAAACTG

+

BBBBBFFFFFFFFFFFFFFFFFFFFFFFFFFFFFFFFFFFFFFFFFFFFFFFFFFFFFFFFFFFFFFFFFFFFFFFFFFFFFFFFFFFFFFFFFFFFFFFFFFFFFFFFFFFFFFFFFFFFFFFFF

@GWZHISEQ02:315:C9E6MANXX:5:1201:11395:83258

CCCCACCATAAGGCTTAGCAAGCTAGCTGCAGTAACGCCATTTTGCAAGGCATGAAAAAGTACCAGAGCTGAGTTCTCAAAAGTCACAAGGAAGTTTAGTTAAAGAATAAGGCTGAACAAAACTGG

+

BBBBBFFFFFFFFFFFFFFFFFFFFFFFFFFFFFFFFFFFFFFFFFFFFFFFFFFFFFFFFFFFFFFFFFFFFFFFFFFFFFFFFFFFFFFFFFFFFFFFFFFFFFFFFFFFFFFFFFFFFFFFFF

@GWZHISEQ02:315:C9E6MANXX:5:1208:16730:44962

CCCCACCATAAGGCTTAGCAAGCTAGCTGCAGTAACGCCATTTTGCAAGGCATGAAAAAGTACCAGAGCTGAGTTCTCAAAAGTCACAAGGAAGTTTAGTTAAAGAAATAAGGCTGAACAAAACTG

+

BBBBBFFFFFFFFFFFFFFFFFFFFFFFFFFFFFFFFFFFFFFFFFFFFFFFFFFFFFFFFFFFFFFFFFFFFFFFFFFFFFFFFFFFFFFFFFFFFFFFFFFFFFFFFFFFFFFFFFFFFFFFFF

@GWZHISEQ02:315:C9E6MANXX:5:1208:4021:57449

CCCCACCATAAGGCTTAGCAAGCTAGCTGCAGTAACGCCATTTTGCAAGGCATGAAAAAGTACCAGAGCTGAGTTCTCAAAAGTCACAAGGAAGTTTAGTTAAAGAATAAGGCTGAACAAAACTGG

+

BBBBBFFFFFF//BBBBFFFFFFBFFFFFFFFFFFFFFFB<FFFFFF<FBFFFBB/B//FFBBF<FFFFFFFF/FFFF<FFFFFFFFFFFFFF<<FFFFFFFFFFFFFFFFFFFFFFFBFFFFFFF

@GWZHISEQ02:315:C9E6MANXX:5:1216:5011:58502

CCCCACCATAAGGCTTAGCAAGCTAGCTGCAGTAACGCCATTTTGCAAGGCATGAAAAAGTACCAGAGCTGAGTTCTCAAAAGTCACAAGGAAGTTTAGTTAAAGAATAAGGCTGAACAAAACTGG

+

<BBBBFFFF<BFBBBBFFBFFFFFFFFFFFFFFFFFB/FFFFFFBFBFFFFF<FFBFFFFFF//F//FF/FFF<BBBFFBFFFFFFFFFFFFFF/FBFFFF/FFF/FFFBFFB/B<FFFB<BFFFF

@GWZHISEQ02:315:C9E6MANXX:5:1310:15183:91508

CCCCACCATAAGGCTTAGCAAGCTAGCTGCAGTAACGCCATTTTGCAAGGCATGAAAAAGTACCAGAGCTGAGTTCTCAAAAGTCACAAGGAAGTTTAGTTAAAGAATAAGGCTGAACAAAACTGG

+

BBBBBFFFBFFBFFFFFFFFFFFFFFFFFFFFFFFFBFBFFFFFBBF/FFF<FF<FFFFFBFFFFFFFFFFFFFFFFFBFFFFFFFFFFFFFFFFFFFFFFFBFFFFFFF<BFBFFFFFFFFFFFF

@GWZHISEQ02:315:C9E6MANXX:5:2101:9546:99585

CCCCACCATAAGGCTTAGCAAGCTAGCTGCAGTAACGCCATTTTGCAAGGCATGAAAAAGTACCAGAGCTGAGTTCTCAAAAGTCACAAGGAAGTTTAGTTAAAGAATAAGGCTGAACAAAACTGG

+

BBBBBFFFFFFFFFFFFBFFFFFFFFFFFFFFFFFFFBFFFFFFFBBFFFF<FFFFFFFFFFBFFFFFBFFFFFFFFFFFFFBFFFFFFFFFFFFFFFF<BFFFFFFBFFFFFFFBFFFFBFFFFF

@GWZHISEQ02:315:C9E6MANXX:5:2102:6303:10811

CCCCACCATAAGGCTTAGCAAGCTAGCTGCAGTAACGCCATTTTGCAAGGCATGAAAAAGTACCAGAGCTGAGTTCTCAAAAGTCACAAGGAAGTTTAGTTAAAGAATAAGGCTGAACAAAACTGG

+

BBBBBFFFFFFFFFFFFFFFFFFFFFFFFFFFFFFFFFFFFFFFFFFFFFFFFFFFFFFFFFFFFFFFFFFFFFFFFFFFFFFFFFFFFFFFFFFFFFFFFFFFFFFFFFFFFFFFFFFFFFFFFF

@GWZHISEQ02:315:C9E6MANXX:5:2115:10783:89115

CCCCACCATAAGGCTTAGCAAGCTAGCTGCAGTAACGCCATTTTGCAAGGCATGAAAAAGTACCAGAGCTGAGTTCTCAAAAGTCACAAGATCGGAAGAGCGTCGTGTAGGGAAAGAGTGTTAAGA

+

BBBBBFFFBFFFFFF/FFFFFFFFFFFFFFFFFFFFFFFFF/BFFFFFFFFFFFFFFFFFFFFFFFFFFFFFFFFFBFFFFFFFFFFFFFFFFFFFFFFFFFFFFFFFFFFFFBFFFFFFFFFFFF

@GWZHISEQ02:315:C9E6MANXX:5:2214:6053:83011

CCCCACCATAAGGCTTAGCAAGCTAGCTGCAGTAACGCCATTTTGCAAGGCATGAAAAAGTACCAGAGCTGAGTTCTCAAAAGTCACAAGGAAGTTTAGTTAAAGAATAAGGCTGAACAAAACTGG

+

BBBBBFFFFFFFFFFFFFFFFFFFFFFFFFFFFFFFFFFBFFFFFFFFFFFFFFFFFFFFFFFFFFFFFFF<B<BBFFFFB<FFFFFF/BBFFFBFFFFBB<BFFFFFFFFBBFFFFFFFBFFFFF

@GWZHISEQ02:315:C9E6MANXX:5:2307:11785:56767

CCCCACCATAAGGCTTAGCAAGCTAGCTGCAGTAACGCCATTTTGCAAGGCATGAAAAAGTACCAGAGCTGAGTTCTCAAAAGTCACAAGAGATCGGAAGAGCGTCGTGTAGGGAAAGAGTGTTAA

+

BBBBBFFFFFFFFFFFFFFFFFFFFFFFFFFFFFFFFFFFFFFFFFFFFFFFFFFFFFFFFFFFFFFFFFFFFFFFFFFFFFFFFFFFFFFFFFFFFFFFFFFFFFFFFFFFFFFFFFFFFFFFFF

@GWZHISEQ02:315:C9E6MANXX:5:2307:11785:56767

CTTGTGACTTTTGAGAACTCAGCTCTGGTACTTTTTCATGCCTTGCAAAATGGCGTTACTGCAGCTAGCTTGCTAAGCCTTATGGTGGGGAGATCGGAAGAGCACACGTCTGAACTCCAGTCACAT

+

BBBBBFFFFFFFFFFFFFFFFFFFFFFFFFFFFFFFFFFFFFFFFFFFFFFFFFFFFFFFFFFFFFFFFFFFFFFFFFFFBFFFFFFFFFFFFFFFFFFFFFFFFFFFFFFFFFFFFFFFFFFFFF

@GWZHISEQ02:315:C9E6MANXX:5:2314:13410:83148

CCAGTTTTGTTCAGCCTTATTCTTTAACTAAACTTCCTTGTGACTTTTGAGAACTCAGCTCTGGTACTTTTTCATGCCTTGCAAAATGGCGTTACTGCAGCTAGCTTGCTAAGCCTTATGGTGGGG

+

BBBBBFFFFFFFFFF<FFBBFF<BFFFFFFFFFFFFFFFFFFFFFFFFFFFBFFFFBFFFFFFFFFFFFFFF/FFFFFFFFFFF/FF<BFBFFFFFFFFFFFB<BBFFFBBFFFFFFFFFBFBBFB

@GWZHISEQ02:315:C9E6MANXX:5:1201:11395:83258

CCCAGTTTTGTTCAGCCTTATTCTTTAACTAAACTTCCTTGTGACTTTTGAGAACTCAGCTCTGGTACTTTTTCATGCCTTGCAAAATGGCGTTACTGCAGCTAGCTTGCTAAGCCTTATGGTGGG

+

BBBBBFFFFFFFFFFFFFFFFFFFFFFFFFFFFFFFFFFFFFFFFFFFFFFFFFFFFFFFFFFFFFFFFFFFFFFFFFFFFFFFFFFFFFFFFFFFFFFFFFFFFFFFFFFFFFFFFFBFFFFFFF

@GWZHISEQ02:315:C9E6MANXX:5:1112:13263:34279

CCACCATAAGGCTTAGCAAGCTAGCTGCAGTAACGCCATTTTGCAAGGCATGAAAAAGTACCAGAGCTGAGTTCTCAAAAGTCACAAGGAAGTTTAGTTAAAGAATAAGGCTGAACAAAACTGGGA

+

BBBBBFFFFFFFFFFFFFFFFFFFFFFFFFFFFFFFFBFFFFFFFFFFFFFFFFFFFFFFFFFFFFFFFFFFFFFFBFFFFFFFFFFFFFFFFFFFFFFFFFFFFFFFF<FFFFFFFFFFFFFFF<

@GWZHISEQ02:315:C9E6MANXX:5:1211:13426:46378

CCACCATAAGGCTTAGCAAGCTAGCTGCAGTAACGCCATTTTGCAAGGCATGAAAAAGTACCAGAGCTGAGTTCTCAAAAGTCACAAGGAAGTTTAGTTAAAGAATAAGGCTGAACAAAACTGGGA

+

BBBBBFFFFFFFFFFFFFFFFFFFFFFFFFFFFFFFFFFFFFFFFFFFFFFF<FF<FFFFFFFFFFFFFFFFFFFFFFFFFFFFFFFFFFFFFFFFFFFFFFFFFFFFFFFFFFFFFF/FFFFFFB

@GWZHISEQ02:315:C9E6MANXX:5:1216:11410:15090

CCACCATAAGGCTTAGCAAGCTAGCTGCAGTAACGCCATTTTGCAAGGCATGAAAAAGTACCAGAGCTGAGTTCTCAAAAGTCACAAGGAAGTTTAGTTAAAGAATAAGGCTGAACAAAACTGGGA

+

B/BBBFFFBFFFFFFFFFFFBBFFFB<FFFFFFFBFFBFFFFFFFFFFFFFFFBFFFFFFFFFFFBFFFFB<FFFFFFFFFFFFFFFFFFFBF/FFB</FFFFFFB<7FFFFB<BFFF7F<B/BB/

@GWZHISEQ02:315:C9E6MANXX:5:1302:7504:42434

CCACCATAAGGCTTAGCAAGCTAGCTGCAGTAACGCCATTTTGCAAGGCATGAAAAAGTACCAGAGCTGAGTTCTCAAAAGTCACAAGGAAGTTTAGTTAAAGAATAAGGCTGAACAAAACTGGAG

+

BBBBBFFFFFFFFFFFFFFFFFFFFFFFFFFFFFFFFFFFFFFFFFFFFFFFFFFFFFFFFFFFFFFFFFFFFFFFFFBFFFFFFFFFFFFFFFFFFFFFFFFFFFFFFFFFFFFFFFFFFFFFFF

@GWZHISEQ02:315:C9E6MANXX:5:1307:4105:47399

CCACCATAAGGCTTAGCAAGCTAGCTGCAGTAACGCCATTTTGCAAGGCATGAAAAAGTACCAGAGCTGAGTTCTCAAAAGTCACAAGGAAGTTTAGTTAAAGAATAAGGCTGAACAAAACTGGCA

+

<BB<B/<BF</<FFBBBFFB//BBBBFB/BBF<BFFFF<B/BFFFBBB</BFFFF/</</<<B/<FFFFFFB<FFFF/<FFF<//FBB//<B<BFBFFFFB//<FFB/7/7BF<FFF<<FFF<FF<

@GWZHISEQ02:315:C9E6MANXX:5:2304:15055:13190

CCACCATAAGGCTTAGCAAGCTAGCTGCAGTAACGCCATTTTGCAAGGCATGAAAAAGTACCAGAGCTGAGTTCTCAAAAGTCACAAGGAAGTTTAGTTAAAGAATAAGAGATCGGAAGAGCGTCG

+

BBBBBFFFFFFFFFFFFFFFFFFFFFFFFFFFFFFFFFFFFFFFFFFFFFFFFFFFFFFFFFFFFFFFFFFFFFFFFFFFFFFFFFFFFFFFFFFFFFFFFFFFFFFFFFFFFFFFFFFFFFFFFF

@GWZHISEQ02:315:C9E6MANXX:5:2313:19478:76976

CCACCATAAGGCTTAGCAAGCTAGCTGCAGTAACGCCATTTTGCAAGGCATGAAAAAGTACCAGAGCTGAGTTCTCAAAAGTCACAAGGAAGTTTAGTTAAAGAATAAGGCTGAACAAAACTGGGA

+

B/BBBFFFFFF<FFFF<FFFFFFFBBF<BFFFFFFFFFFFFFFFFFF<FFFFFFFFFFFF<BFFFFFFFFF<FBBBFFFFFFFFFFBFBFFFFF<BF<FFFFFFFFFFFFFFFFFFFFFFFFFFFF

@GWZHISEQ02:315:C9E6MANXX:5:1302:7504:42434

CCAGTTTTGTTCAGCCTTATTCTTTAACTAAACTTCCTTGTGACTTTTGAGAACTCAGCTCTGGTACTTTTTCATGCCTTGCAAAATGGCGTTACTGCAGCTAGCTTGCTAAGCCTTATGGTGGAG

+

BBBBBFFFFFFFFFFFFFFFFFFFFFFFFFFFFFFFFFFFFFFFFFFFFFFFFFFFFFFFFFFFFFFFFFFFFFFFFFFFFFFFFFFFFFFFFFFFFFFFFFFFFFFFFFFFFFFFFFFFBFFFFF

@GWZHISEQ02:315:C9E6MANXX:5:2304:15055:13190

CTTATTCTTTAACTAAACTTCCTTGTGACTTTTGAGAACTCAGCTCTGGTACTTTTTCATGCCTTGCAAAATGGCGTTACTGCAGCTAGCTTGCTAAGCCTTATGGTGGAGATCGGAAGAGCACAC

+

BBBBBFFFFFFFFFFFFFFFFFFFFFFFFFFFFFFFFFFFFFFFFFFFFFFFFFFFFFFFFFFFFFFFFFFFFFFFFFFFFFFFFFFFFFFFFFFFFFFFFFFFFFFFFFFFFFFFFFFFFFFFFF

@GWZHISEQ02:315:C9E6MANXX:5:1313:10142:62604

CACCATAAGGCTTAGCAAGCTAGCTGCAGTAACGCCATTTTGCAAGGCATGAAAAAGTACCAGAGCTGAGTTCTCAAAAGTCACAAGGAAGTTTAGTTAAAGAATAAGGCTGAACAAAACTGGGAC

+

BBBBBFFFFFFFFFFFFFFFFFFFFFFFFFFFFFFFFFFFFFFFFFFFFFFFFFFFFFFFFFFFFFFFFFFFFFFFFFFFFFFFFFFFFFFFFFFFFFFFFFFFFFFFFFFFFFFFFFFFFFFFFF

@GWZHISEQ02:315:C9E6MANXX:5:2104:20167:84703

CACCATAAGGCTTAGCAAGCTAGCTGCAGTAACGCCATTTTGCAAGGCATGAAAAAAGTACCAGAGCTGAGTTCTCAAAAGTCACAAGGAAGTTTAGTTAAAGAATAAGGCTGAACAAAACTGGGA

+

BBBBBFFFFFFFFFFFFFFFFFFFFFFFFFFFFFFFFFFFFFFFFFFFFFFFFFFFFFFFFFFFFFFFFFFFFFFFFFFFFFFFFFFFFFFFFFFFFFFFFFFFFFFFFFFFFFFFFFFFFFFFFF

@GWZHISEQ02:315:C9E6MANXX:5:2211:14222:5615

CACCATAAGGCTTAGCAAGCTAGCTGCAGTAACGCCATTTTGCAAGGCATGAAAAAGTACCAGAGCTGAGTTCTCAAAAGTCACAAGGAAGTTTAGTTAAAGAATAAGGCTGAACAAAACTGGGAC

+

BBBBBFFFFFFFFFFFFFFFFFFFFFFFFFFFFFFFFFFFFFFFFFFFFFFFFFFFFFFFFFFFFFFFFFFFFFFFFFFFFFFFFFFFFFFFFFFFFFFFFFFFFFFFFFFFFFFFFFFFFFFFFF

@GWZHISEQ02:315:C9E6MANXX:5:1103:10260:18448

CATAAGGCTTAGCAAGCTAGCTGCAGTAACGCCATTTTGCAAGGCATGAAAAAGTACCAGAGCTGAGTTCTCAAAAGTCACAAGGAAGTTTAGTTAAAGAATAAGGCTGAACAAAACTGGGACAGG

+

BBBBBFFFFFFFFFFFFFFFFFFFFFFFFFFFFFFFFFFFFFFFFFFFFFFFFFFFFFFFFFFFFFFFFFFFFFFFFFFFFFFFFFFFFFFFFFFFFFFFFFFFFFFFFFFFFFFFFFFFFFFFFF

@GWZHISEQ02:315:C9E6MANXX:5:1112:16323:39301

CATAAGGCTTAGCAAGCTAGCTGCAGTAACGCCATTTTGCAAGGCATGAAAAAGTACCAGAGCTGAGTTCTCAAAAGTCACAAGGAAGTTTAGTTAAAGAATAAGGCTGAACAAAACTGGGACAGG

+

BBBBBFFFFFFFFFFFFFFBFFFFFFFFFFFFFFFFFFFFFFFFFFFFFFFFFFFFFFFFFFFFFFFFFFFFF<FFFFFFFFFBFFFFFFFFFFFFBFFFFFFFFFFFFFFFFFFFFFFFFFFFFF

@GWZHISEQ02:315:C9E6MANXX:5:1305:4518:10308

CATAAGGCTTAGCAAGCTAGCTGCAGTAACGCCATTTTGCAAGGCATGAAAAAGTACCAGAGCTGAGTTCTCAAAAGTCACAAGGAAGTTTAGTTAAAGAATAAGGCTGAACAAAACTGGGACAGG

+

BBBBBFFFFFFFFFFFFFFFFFFFFFFFFFFFFFFFFFFFFFFFFFFF//FFFFFFFFFFFFFFFFFFFFFFFFFFFFFFFBFFFFFF<FFFFFF<FFFFFFFFFFFFFBFFFFFFFFFFFFFFFF

@GWZHISEQ02:315:C9E6MANXX:5:1305:14473:45019

CATAAGGCTTAGCAAGCTAGCTGCAGTAACGCCATTTTGCAAGGCATGAAAAAGTACCAGAGCTGAGTTCTCAAAAGTCACAAGGAAGTTTAGTTAAAGAATAAGGCTGAACAAAACTGGGACAGG

+

<<BBBFFBFFFB/B<FBFFF/FFFF<FFFFBFFFFFFF/F//<<B<F/<FFBFFBFFFFFFFFB</FBFFFFF<FBFFBB</<B/</FFFF/BFF<FFB<FFFFF<FFFFFFFBFBFFF/FFFB/7

@GWZHISEQ02:315:C9E6MANXX:5:1312:7242:27803

CATAAGGCTTAGCAAGCTAGCTGCAGTAACGCCATTTTGCAAGGCATGAAAAAGTACCAGAGCTGAGTTCTCAAAAGTCACAAGGAAGTTTAGTTAAAGAATAAGGCTGAACAAAACTGGGACAGG

+

BBBBBFFFFFFFFFFFFFFFFFFFFFFFFFFFFFFFFFFFFFFFFFFFFFFFFFFFFFFFFFFFFFFFFFFFFFFFFFFFFFFFFFFFFFFFFFFFFFFFFFFFFFFFFFFFFFFFFFFFFFFFFF

@GWZHISEQ02:315:C9E6MANXX:5:1315:14007:90241

CATAAGGCTTAGCAAGCTAGCTGCAGTAACGCCATTTTGCAAGGCATGAAAAAGTACCAGAGCTGAGTTCTCAAAAGCCACAAGGAAGTTTAGTTAAAGAATAAGGCTGAACAAAACTGGGACAGG

+

BBBBBFFFFFFFFFFFFFFFFFFFFFFFFFFFFFFFFFFFFFFFFFFFFFFFFFFFFFFFFFFFFFFFFFFFFFFFF/FFFFFFFFFFFFFFFFFFFFFFFFFFFFFFFFFFFFFFFFFFFFFFFF

@GWZHISEQ02:315:C9E6MANXX:5:1316:14794:10926

CATAAGGCTTAGCAAGCTAGCTGCAGTAACGCCATTTTGCAAGGCATGAAAAAGTACCAGAGCTGAGTTCTCAAAAGTCACAAGGAAGTTTAGTTAAAGAATAAGGCTGAACAAAACTGGGACAGG

+

BBBBBFFFFFFFFFFFFFFFFFFFFFFFFFFFFFFFFFFFFFFFFFFFFFFFFFFFFFFFFFFFFBFFFFFFFFFFFFFFFFFFFFFFFFFFFFFFFFFFFFFFFFFFFFFFFFFFFFFFFFFFFF

@GWZHISEQ02:315:C9E6MANXX:5:2209:12927:13938

CATAAGGCTTAGCAAGCTAGCTGCAGTAACGCCATTTTGCAAGGCATGAAAAAGTACCAGAGCTGAGTTCTCAAAAGTCACAAGGAAGTTTAGTTAAAGAATAAGGCTGAACAAAACTGGGACAGG

+

BBBBBFFFFFFFFFFFFFFFFFFFFFFFFFFFFFFFFFFFFFFFFFFFFFFFFFFFFFFFFFFFFFFFFFFFFFFFFFFFFFFFFFFFFFFFFFFFFFFFFFFFFFFFFFFFFFFFFFFFFFFFFF

@GWZHISEQ02:315:C9E6MANXX:5:2210:11414:54752

CATAAGGCTTAGCAAGCTAGCTGCAGTAACGCCATTTTGCAAGGCATGAAAAAGTACCAGAGCTGAGTTCTCAAAAGTCACAAGGAAGTTTAGTTAAAGAGATCGGAAGAGCGTCGTGTAGGGAAA

+

BBBBBFFFF<FFFFFFFFFFFBFFFFFFFFFFFFFB<FFFFFFFFFFFFFFFFFFFFFFFFFFFFFFFFFFFFFFFFFFFFFFFFFFFF<FFFFFFFFFFFFFFFFFFFFFFFFFFBFFFFFFFFF

@GWZHISEQ02:315:C9E6MANXX:5:2216:18882:14941

CATAAGGCTTAGCAAGCTAGCTGCAGTAACGCCATTTTGCAAGGCATGAAAAAGTACCAGAGCTGAGTTCTCAAAAGTCACAAGGAAGTTTAGTTAAAGAATAAGGCTGAACAAAACTGGGACAGG

+

BBBBBFFFFFFFFFFFFFFFFFFFFFFFFFFFFFFFFFFFFBFFFFFFFFFFFFFFFFFFFFFFFFFFFFFFFFFFFFFFFFFFFFFFFFFFFFFFFFFFFFFFFFFFFFFFFFFFFFFFFFFFFF

@GWZHISEQ02:315:C9E6MANXX:5:2301:5706:40903

CATAAGGCTTAGCAAGCTAGCTGCAGTAACGCCATTTTGCAAGGCATGAAAAAGTACCAGAGCTGAGTTCTCAAAAGTCACAAGGAAGTTTAGTTAAAGAATAAGGCAGATCGGAAGAGCGTCGTG

+

BBBBBFFF</FFFFFFFFFFF<FFFFFFFFFFFF<FFFFFFFFFFFFFFFFFFFBFFFFFFFFBFFFF/FFFFFFFFFFFFFBFFFFFFBFFFFBFBFFFFFFFFF<FFFFFFFFFBFFFBBFFFF

@GWZHISEQ02:315:C9E6MANXX:5:2303:12448:84720

CATAAGGCTTAGCAAGCTAGCTGCAGTAACGCCATTTTGCAAGGCATGAAAAAGTACCAGAGCTGAGTTCTCAAAAGTCACAAGGAAGTTTAGTTAAAGAATAAGGCTGAACAAAACTGGGACAGG

+

BBBBBFFFFFFFFFFFFFFFFFFFFFFFFFFFFFFFFFFFFFFFFFFFFFFFFFFFFFFFFFFFFFFFFFFFFFFFFFFFFFFFFFFFFFFFFFFFFFFFFFFFFFFFFFFFFFFFFFFFFFFFFF

@GWZHISEQ02:315:C9E6MANXX:5:2314:4855:45797

CATAAGGCTTAGCAAGCTAGCTGCAGTAACGCCATTTTGCAAGGCATGAAAAAGTACCAGAGCTGAGTTCTCAAAAGTCACAAGGAAGTTTAGTTAAAGAATAAGGCTGAACAAAACTGGGACAGG

+

BBBBBFFFFFFFBBBFFFFFFFFFFFFF<FB<<FFFFFFFFFFFFFFFFFFFFFFFFFFFBFFFFFFFFF/FFFFFFFFFFFFFFFFFFFFFFFFFFFFFFFFFFFFFFFFFFFFFBFFF<BFFFF

@GWZHISEQ02:315:C9E6MANXX:5:1307:4105:47399

CCTGTGCCAGTTTTGTTCAGCCTTATTCTTTAACTAAACTTCCTTGTGACTTTTGAGAACTCAGCTCTGGTACTTTTTCATGCCTTGCAAAATGGCGTTACTGCAGCTAGCTTGCTAAGCCTTATG

+

/BBBB//<BF/BBF/<FFFF</F<BFF</B//<FF/FFFFB//<FBFFFF<BF/<FF/BFFF/<<BBF//FFFF/<BF/BFB<//FB//FFFB//<FFBFFFB/FFFF/7<F/FFBF///BF//F/

@GWZHISEQ02:315:C9E6MANXX:5:2204:3740:10707

CCCAGTTTTGTTCAGCCTTATTCTTTAACTAAACTTCCTTGTGACTTTTGAGAACTCAGCTCTGGTACTTTTTCATGCCTTGCAAAATGGCGTTACTGCAGCTAGCTTGCTAAGCCTTATGAGATC

+

BBBBBFF/BFFF///<FFF<F<FF//BBFF<FF<FFFFFFFFFFFFFFFFBFFF<BBB<FFFFFFFBFBF<FFFFFFFFFF/FF/FB<BFFBFFFFFBFFFFFBBFBFFFFB7B/<FFFFB/<FFF

@GWZHISEQ02:315:C9E6MANXX:5:2301:5706:40903

GCCTTATTCTTTAACTAAACTTCCTTGTGACTTTTGAGAACTCAGCTCTGGTACTTTTTCATGCCTTGCAAAATGGCGTTACTGCAGCTAGCTTGCTAAGCCTTATGAGATCGGAAGAGCACACGT

+

BBBBBFF/BFFFFF/BBBFFFFFFFFFFFFFFFFFFFFFFFFBFFF<FFFFFFFFFFFFFFFFFFFFFFBFFFFFFFFFFFFFFFFFFFFFFFFFFFFBFFFFFBFFFBFFFFFFFFFFFFFFFF/

@GWZHISEQ02:315:C9E6MANXX:5:1103:15378:41606

ATAAGGCTTAGCAAGCTAGCTGCAGTAACGCCATTTTGCAAGGCATGAAAAAGTACCAGAGCTGAGTTCTCAAAAGTCACAAGGAAGTTTAGTTAAAGAATAAGAGATCGGAAGAGCGTCGTGTAG

+

BBBBBFFFFFFFFFFFFFFFFFFFFFFFFFFFFFFFFFFFFFFFFFFFFFFFFFFFFFFFFFFFFFFFFFFFFFFFFFFFFFFFFFFFFFFFFFFFFFFFFFFFFFFFFFFFFFFFFFFFFFFFFF

@GWZHISEQ02:315:C9E6MANXX:5:1110:6884:21038

ATAAGGCTTAGCAAGCTAGCTGCAGTAACGCCATTTTGCAAGGCATGAAAAAGTACCAGAGCTGAGTTCTCAAAAGTCACAGATCGGAAGAGCGTCGTGTAGGGAAAGAGTGTTAAGATTAGTGTA

+

BBBBBFFFFFFFFFFFFFFFFFFFFFFFFFFFFFFFFFFFFFFFFFFFFFFFFFFFFFFFFFFFFFFFFFFFFFFFFFFFFFFFFFFFFFFFFFFFFFFFFFFFFFFFFFFFBFFFFFFFFFFFFF

@GWZHISEQ02:315:C9E6MANXX:5:1203:19504:26078

ATAAGGCTTAGCAAGCTAGCTGCAGTAACGCCATTTTGCAAGGCATGAAAAAGTACCAGAGCTGAGTTCTCAAAAGTCACAAGGAAGTTTAGTTAAAGAATAAGGCTGAACAAAACTGGGACAGGA

+

BBBBBFFFFFFFFFFFFFFFFFFFFFFFFFFFFFFFFFFFFFFFFFFFFFFFFFFFFFFFFFFFFFFFFFFFFFFFFFFFF/FFFFFFFFFFFFFFFFFFFFFFFFFFFFFFFFFFFFFFFFFFFB

@GWZHISEQ02:315:C9E6MANXX:5:1211:4329:63767

ATAAGGCTTAGCAAGCTAGCTGCAGTAACGCCATTTTGCAAGGCATGAAAAAGTACCAGAGCTGAGTTCTCAAAAGTCACAAGGAAGTTTAGTTAAAGAATAAGGCTGAACAAAACTGGGACAGGG

+

BBBBBFFFFFFFFFFFFFFFFBFFFFFFFFFFFFFFFFFFFFBFFFFFFFFFFFFFFFFFFFFFFFFFFFFFFFFFFFFFFFFFFFFFFFFFFFFFFFFFFFFFFF/FF<FFFFFFFFFFFFFFFF

@GWZHISEQ02:315:C9E6MANXX:5:1301:10844:59011

ATAAGGCTTAGCAAGCTAGCTGCAGTAACGCCATTTTGCAAGGCATGAAAAAGTACCAGAGCTGAGTTCTCAAAAGTCACAAGGAAGTTTAGTTAAAGAATAAGGCTGAACAAAACTGGGAGATCG

+

BBBBBFFFFFFFFFFFFFFFFFFFFFFFFFFFFFFFFFFFFFFFFFFFFFFFFFFFFFFFFFFFFFFFFFFFFFFFFFFFFFFFFFFFFFFFFFFFFFFFFFFFFFFFFFFFFFFFFFFFFFFBFF

@GWZHISEQ02:315:C9E6MANXX:5:1303:15153:84384

ATAAGGCTTAGCAAGCTAGCTGCAGTAACGCCATTTTGCAAGGCATGAAAAAGTACCAGAGCTGAGTTCTCAAAAGTCACAAGGAAGTTTAGTTAAAGAATAAGGCTGAACAAAACTGGGACAGGG

+

BBBBBFFFFFFFFFFFFFFFFFFFFFFFFFFFFFFFFFFFFFFFFFFFFFFFFFFFFFFFFFFFFFFFFFFFFFFFFFFFFFFFFFFFFFFFFFFFFFFFFFFFFFFFFFFFFFFFFFFFFFFFFF

@GWZHISEQ02:315:C9E6MANXX:5:2116:3294:94309

ATAAGGCTTAGCAAGCTAGCTGCAGTAACGCCATTTTGCAAGGCATGAAAAAGTACCAGAGCTGAGTTCTCAAAAGTCACAAGGAAGTTTAGTTAAAGAATAAGGCTGAACAAAACTGGGACAGGA

+

BBBBBFFFFFFFFFFFFFFFFFFFFFFFFFFBFFFFFFFFFFFFFFFFFFFFFFFFFFFFFFFFFFFFFFFFFFFFFFFFFFFFFFFFFFFFFFFFFFFFFFFFFFFFFFFFFFFFFFFFFFFFFF

@GWZHISEQ02:315:C9E6MANXX:5:2216:4477:92091

ATAAGGCTTAGCAAGCTAGCTGCAGTAACGCCATTTTGCAAGGCATGAAAAAGTACCAGAGCTGAGTTCTCAAAAGTCACAAGGAAGTTTAGTTAAAGAATAAGGCTGAACAAAACTGGGACAGAT

+

BBBBBFFFFFFFFFFFFFFFFFFFFFFFFFFFFFFFFFFFFFFFFFFFFFFFFFFFFFFFFFFFFFFFFFFFFFFFFFFFFFFFFFFFFFFFFFFFFFFFFFFFFFFFFFFFFFFFFFFFFFFFF<

@GWZHISEQ02:315:C9E6MANXX:5:1103:15378:41606

CTTATTCTTTAACTAAACTTCCTTGTGACTTTTGAGAACTCAGCTCTGGTACTTTTTCATGCCTTGCAAAATGGCGTTACTGCAGCTAGCTTGCTAAGCCTTATAGATCGGAAGAGCACACGTCTG

+

BBBBBFFFFFFFFFFFFFFFFFFFFFFFFFFFFFFFFFFFFFFFFFFFFFFFFFFFFFFFFFFFFFFFFFFFFFFFFFFFFFFFFFFFFFFFFFFFFFFFFFFFFFFFFFFFFFFFFFFFFFFFFF

@GWZHISEQ02:315:C9E6MANXX:5:1110:6884:21038

GTGACTTTTGAGAACTCAGCTCTGGTACTTTTTCATGCCTTGCAAAATGGCGTTACTGCAGCTAGCTTGCTAAGCCTTATAGATCGGAAGAGCACACGTCTGAACTCCAGTCACATTACTCGATCT

+

BBBBBFFFFFFFFFFFFFFFFFFFFFFFFFFFFFFFFFFFFFFFFFFFFFFFFFFFFFFFFFFFFFFFFFFFFFFFFFFFFFFFFFFFFFBFFFFFFFFFFFFFFFFFFFFFFFFFFFFFFFF/<<

@GWZHISEQ02:315:C9E6MANXX:5:1202:16834:88996

CCCTGTCCCAGTTTTGTTCAGCCTTATTCTTTAACTAAACTTCCTTGTGACTTTTGAGAACTCAGCTCTGGTACTTTTTCATGCCTTGCAAAATGGCGTTACTGCAGCTAGCTTGCTAAGCCTTAT

+

BBBBBFFFFFFFFFFFFFFFFFFFFFFFFFFFFFFFFFFFFFFFFFFFFFFFFFFFFBFFFFFFFFFFFFFFFFFFFFFFFBFFBFFFFFFFFFFFFFFFFFFFBFFFFFFFFFFFFFFFFFFFFF

@GWZHISEQ02:315:C9E6MANXX:5:1203:19504:26078

CCTGTCCCAGTTTTGTTCAGCCTTATTCTTTAACTAAACTTCCTTGTGACTTTTGAGAACTCAGCTCTGGTACTTTTTCATGCCTTGCAAAATGGCGTTACTGCAGCTAGCTTGCTAAGCCTTATA

+

BBB<BFFBFFBFFFFFFFF<FFFFFFFFFFFFFFBFFFFBFFFFFFF</FFFFFFB<FFFFBFFBBBFF<FBFFFFFFB<F/FFFBFFBFF<FFFFFFFBFFFFFFFFFFFFFFFFF</</FFBFB

@GWZHISEQ02:315:C9E6MANXX:5:1212:3341:43945

CCCTGTCCCAGTTTTGTTCAGCCTTATTCTTTAACTAAACTTCCTTGTGACTTTTGAGAACTCAGCTCTGGTACTTTTTCATGCCTTGCAAAATGGCGTTACTGCAGCTAGCTTGCTAAGCCTTAT

+

BBBBBFFFFFFFFFFFFFFFFFFFFFFFFFFFFFFFFFFFFFFFFFFFFFFFFFFFFFFFFFFFFFFFFFFFFFFFFFFFFFFFFFFFFFFFFFFFFFFFFFFFFFFFFFFFFFFFFFFFFFFFFF

@GWZHISEQ02:315:C9E6MANXX:5:1301:10844:59011

CCCAGTTTTGTTCAGCCTTATTCTTTAACTAAACTTCCTTGTGACTTTTGAGAACTCAGCTCTGGTACTTTTTCATGCCTTGCAAAATGGCGTTACTGCAGCTAGCTTGCTAAGCCTTATAGATCG

+

BBBBBFFFFFFFFFFFFFFFFFFFFFFFFFFFFFFFFFFFFFFFFFFFFFFFFFFFFFFFFFFFFFFFFFFFFFFFFFFFFFFFFFFFBFFFFFFFFFFFFFFFFFFFFFFFBFFFBFFFFFFFFF

@GWZHISEQ02:315:C9E6MANXX:5:2116:3294:94309

CCTGTCCCAGTTTTGTTCAGCCTTATTCTTTAACTAAACTTCCTTGTGACTTTTGAGAACTCAGCTCTGGTACTTTTTCATGCCTTGCAAAATGGCGTTACTGCAGCTAGCTTGCTAAGCCTTATA

+

BBBBBFFFFFF<BFFFFFFFFFFFFFFFFFFFFFFFFFFFFFFFFFFFFFFFFFFFFFFFFFFFFFFFFFFFFFFFFFFFFFFFFFFFFFFFFFFFFFFFFFFFFFFFFFFFFFFFFFFFFFFFFF

@GWZHISEQ02:315:C9E6MANXX:5:2216:4477:92091

GTCCCAGTTTTGTTCAGCCTTATTCTTTAACTAAACTTCCTTGTGACTTTTGAGAACTCAGCTCTGGTACTTTTTCATGCCTTGCAAAATGGCGTTACTGCAGCTAGCTTGCTAAGCCTTATAGAT

+

BBBBBFFFFFFFFFFFFFFFFFFFFFFFFFFFFFFFFFFFFFFFFFFFFFFFFFFFFFFFFFFFFFFFFFFFFFFFFFFFFFFFFFFFFFFFFFFFFFFFFFFFFFFFFFFFFFFFFFFFFFFFFF

@GWZHISEQ02:315:C9E6MANXX:5:1208:8653:25117

TAAGGCTTAGCAAGCTAGCTGCAGTAACGCCATTTTGCAAGGCATGAAAAAGTACCAGAGCTGAGTTCTCAAAAGTCACAAGGAAGTTTAGTTAAAGAATAAGAGATCGGAAGAGCGTCGTGTAGG

+

BBBBBFFFFFFFFFFFFFFFFFFFFFFFFFFFFFFFFFFFFFFFFFFFFFFFFFFFFFFFFFFFFFFFFFFFFFFFFFFFFFFFFFFFFFFFFFFFFFFFFFFFFFFFFFFFFFFFFBFFB<FFFF

@GWZHISEQ02:315:C9E6MANXX:5:2303:18653:60500

TAAGGCTTAGCAAGCTAGCTGCAGTAACGCCATTTTGCAAGGCATGAAAAAGTACCAGAGCTGAGTTCTCAAAAGTCACAAGGAAGTTTAGTTAAAGAATAAGAGATCGGAAGAGCGTCGTGTAGG

+

BBBBBFFFFFFFFFFFFFFFFFFFFFFFFFFFFFFFFFFFFFFFFFFFFFFFFFFFFFFFFFFFFFFFFFFFFFFFFFFFFFFFFFFFFFFFFFFFFFFFFFFFFFFFFFFFFFFFFFFFBFFFFF

@GWZHISEQ02:315:C9E6MANXX:5:1113:12931:97548

CCCCTGTCCCAGTTTTGTTCAGCCTTATTCTTTAACTAAACTTCCTTGTGACTTTTGAGAACTCAGCTCTGGTACTTTTTCATGCCTTGCAAAATGGCGTTACTGCAGCTAGCTTGCTACGCCTTA

+

BBBBBFFFFFFFFBFBFFFFFFFFFF<BBFFFFBBF</F/FFFFBFFFFBFFFFFFF/F/FFFFFFFF/<FBFBFFFFFFB/<BFFFFFFBB/<FF/FBFFFFFFFFFFF7//<<FFFFFFF/<//

@GWZHISEQ02:315:C9E6MANXX:5:1208:8653:25117

CTTATTCTTTAACTAAACTTCCTTGTGACTTTTGAGAACTCAGCTCTGGTACTTTTTCATGCCTTGCAAAATGGCGTTACTGCAGCTAGCTTGCTAAGCCTTAAGATCGGAAGAGCACACGTCGAA

+

BBBBBFFFFFFFFFFFFFFFFFFFFFFFFFFFFFFFFFFFFFFFFFFFFFFFFFFFFFBF<BFFFFFFFFFFFFFFFFFFFFFFFFFFFFFFFFF/FFFFFFFFFFFF<FFFFFFFFFFFFFFFFF

@GWZHISEQ02:315:C9E6MANXX:5:2301:6915:8188

CTTATTCTTTAACTAAACTTCCTTGTGACTTTTGAGAACTCAGCTCTGGTACTTTTTCATGCCTTGCAAAATGGCGTTACTGCAGCTAGCTTGCTAAGCCTTAGATCGGAAGAGCACACGTCTGAA

+

BBBBBFFFFFFFFFFFFFFFFFFFFFFFFFFFFFFFFFFFFFFFFFFFFFFFFFFFFFFFFFFFFFFFFFFFFFFFFFFFFFFFFFFFFFFFFFFFFFFFFFFFFFFFFFFFFFFFFFFFFFFFFF

@GWZHISEQ02:315:C9E6MANXX:5:2303:18653:60500

CTTATTCTTTAACTAAACTTCCTTGTGACTTTTGAGAACTCAGCTCTGGTACTTTTTCATGCCTTGCAAAATGGCGTTACTGCAGCTAGCTTGCTAAGCCTTAAGATCGGAAGAGCACACGTCGAA

+

BBBBBFFFFFFFFFFFFFFFFFFFFFFFFFFFFFFFFFFFFFFFFFFFFFFFFFFFFFFFFFFFFFFFFFFFF/FFFFFFFFFFFFFFFFFFFFFBFBFFFFFFFBFFFFFFFFFFFFFFFFFFBB

@GWZHISEQ02:315:C9E6MANXX:5:2303:10013:99483

AAGGCTTAGCAAGCTAGCTGCAGTAACGCCATTTTGCAAGGCATGAAAAAGTACCAGAGCTGAGTTCTCAAAAGTCACAAGGAAGTTTAGTTAAAGAATAAGGCTGAACAAAACTGGGACAGGGGC

+

BBBBBFFFFFFFFFFFFFFFFFFFFFFFFFFFFFFFFFFFFFFFFFFFFFFFFFFFFFFFFFFFFFFFFFFFFFFFFFFFFFFFFFFFFFFFFFFFFFFFFFFFFFFFFFFFFFFFFFFFFFFFFF

@GWZHISEQ02:315:C9E6MANXX:5:2310:19910:36731

AAGGCTTAGCAAGCTAGCTGCAGTAACGCCATTTTGCAAGGCATGAAAAAGTACCAGAGCTGAGTTCTCAAAAGTCACAAGGAAGTTTAGTTAAAGAATAAGGCTGAACAAAACTGGGACAGGGGC

+

BBBBBFFFFFFFFFFFFFFFFFFFFFFFFFFFFFFFFFFFFFFFFFFFFFFFFFFFFFFFFFFFFFFFFFFFFFFFFFFFFFFFFFFFFFFFFFFFFFFFFFFFFFFFFFFFFFFFFFFFFFFFFF

@GWZHISEQ02:315:C9E6MANXX:5:1216:11410:15090

GCCTCTGTCCCAGTTTTGTTCAGCCTTATTCTTTAACTAAACTTCCTTGTGACTTTTGAGAACTCAGCTCTGGTACTTTTTCATGCCTTGCAAAATGGCGTTACTGCAGCTAGCTTGCTAAGCCTT

+

BBBBBFFFFFFFFFFFFFFFFFFFFFFFFFFFFFFFFFFFFFFFFFFFFFBFFFFFFBFFFFFFFFBFBF<FFFFFFFFFFFFFFFFBFF/F/<<B/FFF/BFFFB/F<F<FFFFFFFFBFBBFBF

@GWZHISEQ02:315:C9E6MANXX:5:1213:11264:34735

ATCAGGCTTAGCAAGCTAGCTGCAGTAACGCCATTTTGCAAGGCATGAAAAAGTACCAGAGCTGAGTTCTCAAAAGTCACAAGGAAGTTTAGTTAAAGAATAAGGCTGAACAAAACTGGGACAGGG

+

BBBBBBFFFFFFFFFFFFFFFFFFFFFFFFFFFFFFFFFFFFFFFFFFFFFFFFBFFFFFFFFFFF<<BFFFFFFFFFFFFFFFFFFFFFFFFFFFFFFBB/FFFFFFFFFFFFFFFFFFFFFB<F

@GWZHISEQ02:315:C9E6MANXX:5:1302:20821:19955

AGGCTTAGCAAGCTAGCTGCAGTAACGCCATTTTGCAAGGCATGAAAAAGTACCAGAGCTGAGTTCTCAAAAGTCACAAGGAAGTTTAGTTAAAGAATAAGGCTGAACAAAACTGGGACAGGGGCC

+

BBBBBFFFFFFFFFFFFFFFFFFFFFFFFFFFFFFFFFFFFFFFFFFFFFFFFFFFFFFFFFFFFFFFFFFFFFFFFFFFFFFFFFFFFFFFFFFFFFFFFFFFFFFFFFFFFFFFFFFFFFFFFF

@GWZHISEQ02:315:C9E6MANXX:5:2215:21304:88701

AGGCTTAGCAAGCTAGCTGCAGTAACGCCATTTTGCAAGGCATGAAAAAGTACCAGAGCTGAGTTCTCAAAAGTCACAAGGAAGTTTAGTTAAAGAATAAGGCTGAACAAAACTGGGACAGGGGCC

+

BBBBBFFFFFFFFFFF<FFFFFFFFFB/<FFFFFBFFFFFFFFFFFFFFBFFBFFFFFFFFFFFF<FFFFFFFFFFFFFFBFFFFFFFF/FFFFFFFFFFFFFFFFFFFFFFFFF//FFF/<<FFB

@GWZHISEQ02:315:C9E6MANXX:5:1311:1312:67347

GGCCCCTGTCCCAGTTTTGTTCAGCCTTATTCTTTAACTAAACTTCCTTGTGACTTTTGAGAACTCAGCTCTGGTACTTTTTCATGCCTTGCAAAATGGCGTTACTGCAGCTAGCTTGCTAAGCCT

+

BBBBBFFFFFFFFFFFFFFFFFFFFFFFFFFFFFFFFFFFFFFFFFFFFFFFFFFFFFFFFFFFFFFFFFFFFFFFFFFFFFFFFFFFFFFFFFFBFFBFFFFFFFFFFFFFFFFBFFFFB<FFFF

@GWZHISEQ02:315:C9E6MANXX:5:2313:18929:50517

GGCCCCTGTCCCAGTTTTGTTCAGCCTTATTCTTTAACTAAACTTCCTTGTGACTTTTGAGAACTCAGCTCTGGTACTTTTTCATGCCTTGCAAAATGGCGTTACTGCAGCTAGCTTGCTAAGCCT

+

//BBBFFFFFFFFFFFFFFFF<FFFFFFFFFFFFFBBFFFFFFFFFBFFFF/FF<BFBFFFFFFFBFFBFFF<BFFFFFFFFFFFFFBFFF<FBFBF/BBFFFBFF<BBF<FFFFF<77FFFFFFF

@GWZHISEQ02:315:C9E6MANXX:5:1106:5891:4362

GGCTTAGCAAGCTAGCTGCAGTAACGCCATTTTGCAAGGCATGAAAAAGTACCAGAGCTGAGTTCTCAAAAGTCACAAGGAAGTTTAGTTAAAGAATAAGGCTGAACAAAACTGGGACAGGGGCCA

+

BBBBBFFFFFFFFFFFFFFFFFFFFFFFFFFFFFFFFFFFFFFFFFFFFFFFFFFFFFFFFFFFFFFFFFFFFFFFFFFFFFFFFFFFFFFFFFFFFFFFFFFFFFFFFFFFFFFFFFFFFFFFFF

@GWZHISEQ02:315:C9E6MANXX:5:1111:15518:5195

GGCTTAGCAAGCTAGCTGCAGTAACGCCATTTTGCAAGGCATGAAAAAGTACCAGAGCTGAGTTCTCAAAAGTCACAAGGAAGTTTAGTTAAAGAATAAGGCTGAACAAAACTGGGACAGGGGCCA

+

BBBBBFFFFFFFFFFFFFFFFFFFFFFFFFFFFFFFFFFFFFFFFFFFFFFFFFFFFFFFFFFFFFFFFFFFFFFFFFFFFFFFFFFFFFFFFFFFFFFFFFFFFFFFFFFFFFFFFFFFFFFFFF

@GWZHISEQ02:315:C9E6MANXX:5:1201:13951:31361

GGCTTAGCAAGCTAGCTGCAGTAACGCCATTTTGCAAGGCATGAAAAAGTACCAGAGCTGAGTTCTCAAAAGTCACAAGGAAGTTTAGTTAAAGAATAAGGCTGAACAAAACTGGGACAGGGGCCA

+

BBBBBFFFFFFFFBFFFFFFFFFFFFBFFFFFFFFFFFFFFFFFFFFFFFFFFFFFFFFFFFFFFFFFFFFFFFFFFFFFFFFFFFFFFFFFFFFFFFFFFFFFFFFFFFFFFFFFFFFFFBFFFB

@GWZHISEQ02:315:C9E6MANXX:5:1208:20617:72933

GGCTTAGCAAGCTAGCTGCAGTAACGCCATTTTGCAAGGCATGAAAAAGTACCAGAGCTGAGTTCTCAAAAGTCACAAGGAAGTTTAGTTAAAGAATAAGGCTGAACAAAACTGGGACAGGGAGAT

+

BBBBBFFFFFFFFFFFFFFFFFFFFFFFFFFFFFFFFFFFFFFFFFFFFFFFFFFFFFFFFFFFFFFFFFFFFFFFFFFFFFFFFFFFFFFFFFFFFFFFFFFFFFFFFFFFFFFFFFFFFFFFFF

@GWZHISEQ02:315:C9E6MANXX:5:1210:10288:97270

GGCTTAGCAAGCTAGCTGCAGTAACGCCATTTTGCAAGGCATGAAAAAGTACCAGAGCTGAGTTCTCAAAAGTCACAAGGAAGTTTAGTTAAAGAATAAGGCTGAACAAAACTGGGACAGGGGCCA

+

BBBBBFFFFFFFFFFFFFFFFFFFFFFFFFFFFFFFFFFFBFFFFFFFFFFFFFFFFFFFFFFFFFFFFFFFFFFFFFFFFFFFFFFFFFFFFFFFFFFFFFFFFFFFFFBFFFFFFFFFFFFFF<

@GWZHISEQ02:315:C9E6MANXX:5:2108:16773:35951

GGCTTAGCAAGCTAGCTGCAGTAACGCCATTTTGCAAGGCATGAAAAAGTACCAGAGCTGAGTTCTCAAAAGTCACAAGGAAGTTTAGTTAAAGAATAAGGCTGAACAAAACTGGGACAGGGGCCA

+

BBBBBFFFFFFFFFFFFFFFFFFFFFFFFFFFFFFFFFFFFFFFFFFFFFFFFFFFFFFFFFFFFFFFFFFFFFFFFFFFFFFFFFFFFFFFFFFFFFFFFFFFFFFFFFFFFFFFFFFFFFFFFF

@GWZHISEQ02:315:C9E6MANXX:5:2113:14360:75009

GGCTTAGCAAGCTAGCTGCAGTAACGCCATTTTGCAAGGCATGAAAAAGTACCAGAGCTGAGTTCTCAAAAGTCACAAGGAAGTTTAGTTAAAGAATAAGGCTGAACAAAACTGGGACAGGGGCCA

+

BBBBBFFFFFFFFFFFFFFFFFFFFFFFFFFFFFFFFFFFFFFFFFFFFFFFFFFFFFFFFFFFFFFFFFFFFFFFFFFFFFFFFFFFFFFFFFFFFFFFFFFFFFFFFFFFFFFFFFFFFFFFFF

@GWZHISEQ02:315:C9E6MANXX:5:2114:20641:54068

GGCTTAGCAAGCTAGCTGCAGTAACGCCATTTTGCAAGGCATGAAAAAGTACCAGAGCTGAGTTCTCAAAAGTCACAAGGAAGTTTAGTTAAAGAATAAGGCTGAACAAAACTGGGACAGGGGCCA

+

BBBBBFFFFFFFFFFFFFFFFFFFFFFFFFFFFFFFFFFFFFFFFFFFFFFFFFFFFFFFFFFFFFFFFFFFFFFFFFFFFFFFFFFFFFFFFF/FFFFFFFFFFFFFFFFFFFFFFFFBFFFFFF

@GWZHISEQ02:315:C9E6MANXX:5:2114:6631:85084

GGCTTAGCAAGCTAGCTGCAGTAACGCCATTTTGCAAGGCATGAAAAAGTACCAGAGCTGAGTTCTCAAAAGTCACAAGGAAGTTTAGTTAAAGAATAAGGCTGAACAAAACTGGGACAGGGGCCA

+

BBBBBFFFFFFFFFFFFFFFFF<FFFFFFBFFFFFFFFBFFFFFFFFFFFFFFFFBFBFFFFFBBFFFFFFFFFFFFFFFFFFFFFFFFFFFFFFFFFFFFFFFFFFFFFFFFFFFFFFFFFFFFB

@GWZHISEQ02:315:C9E6MANXX:5:2215:8666:55453

GGCTTAGCAAGCTAGCTGCAGTAACGCCATTTTGCAAGGCATGAAAAAGTACCAGAGCTGAGTTCTCAAAAGTCACAAGGAAGTTTAGTTAAAGAATAAGGCTGAACAAAACTGGGACAGGGGCCA

+

BBBBBFFFFFFFFFFFFFFFFFFFFFFFFFFFFFFFFFFFFFFFFFFFFF/FFFFFFFFFFFFFFFFFFFFFFFFFFFFFFFFFFFFFFFFFFFFFFFFFFFFFFFFFBFFFFFFFFFFFFFFFF<

@GWZHISEQ02:315:C9E6MANXX:5:1208:20617:72933

CCCTGTCCCAGTTTTGTTCAGCCTTATTCTTTAACTAAACTTCCTTGTGACTTTTGAGAACTCAGCTCTGGTACTTTTTCATGCCTTGCAAAATGGCGTTACTGCAGCTAGCTTGCTAAGCCAGAT

+

BBBBBFFFFFFFFFFFFFFFFFFFFFFFFFFFFFFFFFFFFFFFFFFFFFFFFFFFFFFFFFFFFFFFFFFFFFFFFFFFFFFFFFFFFFFFBFFFFFFFFFFFFFFFFFFFFFFFFFFFFFFFFF

@GWZHISEQ02:315:C9E6MANXX:5:1103:7584:84634

GCTTAGCAAGCTAGCTGCAGTAACGCCATTTTGCAAGGCATGAAAAAGTACCAGAGCTGAGTTCTCAAAAGTCACAAGGAAGTTTAGTTAAAGAATAAGGCTGAACAAAACTGGGACAGGGAGATC

+

BBBBBFFFFFFFFFFFFFFFFFFFFFFFFFFFFFFFFFFFFFFFFFFFFFFFFFFFFFFFFFFFFFFFFFFFFFFFFFFFFFFFFFFFFFFFFFFFFFFFFFFFFFFFFFFFFFFFFFFFFFFFFF

@GWZHISEQ02:315:C9E6MANXX:5:2103:12429:65091

GCTTAGCAAGCTAGCTGCAGTAACGCCATTTTGCAAGGCATGAAAAAGTACCAGAGCTGAGTTCTCAAAAGTCACAAGGAAGTTTAGTTAAAGAATAAGGCTGAACAAAACTGGGACAGGGGCCAA

+

BBBBBFFFFFFFFFFFFFFFFFFFFFFFFFFFFFFFFFFFFFFFFFFFFFFFFFFFFFFFFFFFFFFFFFFFFFFFFFFFFFBFFFFFFFFFFFBFFFFFFFFFFFFFFFFFFFFFFFFFFFFFFF

@GWZHISEQ02:315:C9E6MANXX:5:1103:7584:84634

CCCTGTCCCAGTTTTGTTCAGCCTTATTCTTTAACTAAACTTCCTTGTGACTTTTGAGAACTCAGCTCTGGTACTTTTTCATGCCTTGCAAAATGGCGTTACTGCAGCTAGCTTGCTAAGCAGATC

+

BBBBBFFFFFFFFFFFFFFFFFFFFFFFFFFFFFFFFFFFFFFFFFFFFFFFFFFFFFFFFFFFFFFFFFFFFFFFFFFFFFFBFFFFFFFFFFFFFBFFFFFFFFFFFFFFFFFFFFFFFFFFFF

@GWZHISEQ02:315:C9E6MANXX:5:1207:2498:28706

CTTAGCAAGCTAGCTGCAGTAACGCCATTTTGCAAGGCATGAAAAAGTACCAGAGCTGAGTTCTCAAAAGTCACAAGGAAGTTTAGTTAAAGAATAAGGCTGAACAAAACTGGGACAGGGGCCAAA

+

BBBBBFFFFFFFFFFFFFFFFFFFFFFFFFFFFFFFFFFFFFFFFFFFFFFFFFFFFFFFFFFFFFFFFFFFFFFFFFFFFFFFFFFFFFFFFFFFFFFFFFFFFFFFFFFFFFFFFFFFFFBFFB

@GWZHISEQ02:315:C9E6MANXX:5:1210:3182:15340

CTTAGCAAGCTAGCTGCAGTAACGCCATTTTGCAAGGCATGAAAAAGTACCAGAGCTGAGTTCTCAAAAGTCACAAGGAAGTTTAGTTAAAGAATAAGGCTGAACAAAACTGGGACAGGGGCCAAA

+

BBBBBFFFFFFFFFFFFFFFFFFFFFFFFFFFFFFFFFFFFFFFFFFFFFFFFFFFFFFFFFFFFFFFFFFFFFFFFFFFFFFFFFFFFFFFFFFFFFFFFFFFFFFFFFFFFFFFFFFFFFFFFF

@GWZHISEQ02:315:C9E6MANXX:5:2216:2470:44291

CTTAGCAAGCTAGCTGCAGTAACGCCATTTTGCAAGGCATGAAAAAGTACCAGAGCTGAGTTCTCAAAAGTCACAAGGAAGTTTAGTTAAAGAATAAGGCTGAACAAAACTGGGACAGGGGCCAAA

+

BBB/BFF<FFFFFFFFFFFFFFFFFFFBFFFFFFFFFFFFFFFFFFFFFFFFFBFFFFFFFFFFFFFFFFF<FB/FBF/FF<FFFFFBF/BFFF<FFFFBFB//FFFFFFF//FFFBB77BB7FFF

@GWZHISEQ02:315:C9E6MANXX:5:2102:16993:11370

GCAAGCTAGCTGCAGTAACGCCATTTTGCAAGGCATGAAAAAGTACCAGAGCTGAGTTCTCAAAAGTCACAAGGAAGTTTAGTTAAAGAATAAGGCTGAACAAAACTGGGACAGGGGCCAGATCGG

+

BBBBBFFFFFFFFFFFFFFFFFFFFFFFFFFFFFFFFFFFFFFFFFFFFFFFFFFFFFFFFFFFFFFFFFFFFFFFFFFFFFFFFFFFFFFFFFFFFFFFFFFFFFFFFFFFFFFFFFFFFFFFFF

@GWZHISEQ02:315:C9E6MANXX:5:1213:11264:34735

CCTGTTTGGCCCCTGTCCCAGTTTTGTTCAGCCTTATTCTTTAACTAAACTTCCTTGTGACTTTTGAGAACTCAGCTCTGGTACTTTTTCATGCCTTGCAAAATGGCGTTACTGCAGCTAGCTTGC

+

BBBBBFFF<F<FFFFFFBFFF<FFFFFFFFFFFFFFFFFF/FBFBFFFBFFB<FFBFFF/FFFFFFFFFFFFFFFBFFFFFFFFFFFFFFFFFFFFFBF<FFBFFF<FFB7BFFBFFFFFFFFFF<

@GWZHISEQ02:315:C9E6MANXX:5:1301:21028:29302

GGCCCCTGTCCCAGTTTTGTTCAGCCTTATTCTTTAACTAAACTTCCTTGTGACTTTTGAGAACTCAGCTCTGGTACTTTTTCATGCCTTGCAAAATGGCGTTACTGCAGCTAGCTTGCAGATCGG

+

/BBBBFFFFFFFFFFFFFBFFFFFFFFFFFFFFFFFFFFFFFFFFFFFFFFFFFFFFFFFFFFFFFFFFFBBFFFFFFFFFFFFFFFFFF<FFFFFFBBFFFFFFFFFFFBFFFFFF/FFFFFFFF

@GWZHISEQ02:315:C9E6MANXX:5:1312:7242:27803

CCTGTTTGGCCCCTGTCCCAGTTTTGTTCAGCCTTATTCTTTAACTAAACTTCCTTGTGACTTTTGAGAACTCAGCTCTGGTACTTTTTCATGCCTTGCAAAATGGCGTTACTGCAGCTAGCTTGC

+

BBBBBFFFFFFFFFFFFFFFFFFFFFFFFFFFFFFFFFFFFFFFFFFFFFFFFFFFFFFFFFFFFFFFFFFFFFFFFFFFFFFFFFFFFFFFFFFFFFFFFBFFFFFFFFFFFFFFFFFFFFFFFF

@GWZHISEQ02:315:C9E6MANXX:5:1115:20963:86548

CAAGCTAGCTGCAGTAACGCCATTTTGCAAGGCATGAAAAAGTACCAGAGCTGAGTTCTCAAAAGTCACAAGGAAGTTTAGTTAAAGAATAAGGCTGAACAAAACTGGGACAGGGGCCAAACAGGA

+

BBBBBFFFFFFFFFFFFFFFFFFFFFFFFFFFFFFFFFFFF<FFFFFFFFFFFFFFFFFFFFFFFFFFFFFFFFFFFFFFFFFFFFFFFFFFFFFFBFFFFFFFFFFFFFFFFFFFFFFFFFFFFF

@GWZHISEQ02:315:C9E6MANXX:5:1311:4982:77953

CAAGCTAGCTGCAGTAACGCCATTTTGCAAGGCATGAAAAAGTACCAGAGCTGAGTTCTCAAAAGTCACAAGGAAGTTTAGTTAAAGAATAAGGCTGAACAAAACTGGGAGATCGGAAGAGCGTCG

+

BBBBBFFFFFFFFFFFFFFFFFFFFFFFFFFFFFFFFFFFFFFFFFFFFFFFFFFFFFFFFFFFFFFFFFFFFFFFFFFFFFFFFFFFFFFFFFFFFFFFFFFFFFFFFFFFFFFFFFFFFFFFFF

@GWZHISEQ02:315:C9E6MANXX:5:1313:15834:41932

CAAGCTAGCTGCAGTAACGCCATTTTGCAAGGCATGAAAAAGTACCAGAGCTGAGTTCTCAAAAGTCACAAGGAAGTTTAGTTAAAGAATAAGGCTGAACAAAACTGGGACAGGGGCCAAACAGGA

+

BBBBBFFFFFFFFFFFFFFFFFFFFFFFFFFFFFFFFFFFFFFFFFFFFFFFFFFFFFFFFFFFFFFFFFFFFFFFFFFFFFFFFFFFFFFFFFFFFFFFFFFFFFFFFFFFFFFFFFFFFFFFFF

@GWZHISEQ02:315:C9E6MANXX:5:2105:4926:16957

CAAGCTAGCTGCAGTAACGCCATTTTGCAAGGCATGAAAAAGTACCAGAGCTGAGTTCTCAAAAGTCACAAGGAAGTTTAGTTAAAGAATAAGGCTGAACAAAACTGGGAGATCGGAAGAGCGTCG

+

BBBBBFFFFFFFFFFFFFFFFFFFFFFFFFFFFFFFFFFFFFFFFFFFFFFFFFFFFFFFFFFFFFFFFFFFFFFFFFFFFFFFFFFFFFFFFFFFFFFFFFFFFFFFFFFFFFFFFFFFFFFFFF

@GWZHISEQ02:315:C9E6MANXX:5:2105:13693:63125

CAAGCTAGCTGCAGTAACGCCATTTTGCAAGGCATGAAAAAGTACCAGAGCTGAGTTCTCAAAAGTCACAAGGAAGTTTAGTTAAAGAATAAGGCTGAACAAAACTGGGACAGGGGCCAAACAGGA

+

BBBBBFFFFFFFFFFFFFFFFFFFFFFFFFFFFFFFFFFFFFFFFFFFFFFFFFFFFFFFFFFFFFFFFFFFFFFFFFFFFFFFFFFFFFFFFFFFFFFFFFFFFFFFFFFFFFFFFFFFFFFFFF

@GWZHISEQ02:315:C9E6MANXX:5:2209:10172:54744

CAAGCTAGCTGCAGTAACGCCATTTTGCAAGGCATGAAAAAGTACCAGAGCTGAGTTCTCAAAAGTCACAAGGAAGTTTAGTTAAAGAATAAGGCTGAACAAAACTGGGACAGGGGCCAAACAGGA

+

BBB/BBBFFFFFFFFFFFFBFFFFFFFFFFFFFFFFFB<FF<FFBBFFBBFFFFB//F/F<B/FFBFFFFFFFFFFFFFFFFFFFFFFFFFFFBFBFFFFFFF/BFBFFFFFBF<BFFBFFFFF/<

@GWZHISEQ02:315:C9E6MANXX:5:2210:13645:37709

CAAGCTAGCTGCAGTAACGCCATTTTGCAAGGCATGAAAAAGTACCAGAGCTGAGTTCTCAAAAGTCACAAGGAAGTTTAGTTAAAGAATAAGGCTGAACAAAACTGGGACAGGGGCCAAACAGGA

+

BBBBBFFFFFFFFFFFFFFFFBFFFFFFFFFFFFFFFFFFFFFFFFFFFFFFFFFFFFFFFFFFFFFFFFFFFFFFFFFFFFFFFFFFFFFFFBFFFFFFFFFFFFFFFFFFFFFFFFFFFFFFFF

@GWZHISEQ02:315:C9E6MANXX:5:2303:14686:96876

CAAGCTAGCTGCAGTAACGCCATTTTGCAAGGCATGAAAAAGTACCAGAGCTGAGTTCTCAAAAGTCACAAGGAAGTTTAGTTAAAGAATAAGGCTGAACAAAACTGGGACAGGGAGATCGGAAGA

+

BBBBBFFFFFFFFFFBFFFFFFFFFFFFFFFFFFFFFFFFFFFFFFFFFFFFFFFFFFFFFFFFFFFFFFFFFFFFFFFFFFFFFFFFFFFFFFFFFFFFFFFFFFFFFFFFFFFFFFBFFFFFF7

@GWZHISEQ02:315:C9E6MANXX:5:1311:4982:77953

CCCAGTTTTGTTCAGCCTTATTCTTTAACTAAACTTCCTTGTGACTTTTGAGAACTCAGCTCTGGTACTTTTTCATGCCTTGCAAAATGGCGTTACTGCAGCTAGCTTGAGATCGGAAGAGCACAC

+

BBBBBFFFFFFFFFFFFFFFFFFFFFFFFFFFFFFFFFFFFFFFFFFFFFFFFFFFFFFFFFFFFFFFFFFFFFFFFFFFFFFFFFFFFFFFFFFFFFFFFFFFFFFFFFFFFFFFFFFFFFFFFF

@GWZHISEQ02:315:C9E6MANXX:5:2105:4926:16957

CCCAGTTTTGTTCAGCCTTATTCTTTAACTAAACTTCCTTGTGACTTTTGAGAACTCAGCTCTGGTACTTTTTCATGCCTTGCAAAATGGCGTTACTGCAGCTAGCTTGAGATCGGAAGAGCACAC

+

BBBBBFFFFFFFFFFFFFFFFFFFFFFFFFFFFFFFFFFFFFFFFFFFFFFFFFFFFFFFFFFFFFFFFFFFFFFFFFFFFFFFFFFFFFFFFFFFFFFFFFFFFFFFFFFFFFFFFFFFFFFFFF

@GWZHISEQ02:315:C9E6MANXX:5:2303:14686:96876

CCCTGTCCCAGTTTTGTTCAGCCTTATTCTTTAACTAAACTTCCTTGTGACTTTTGAGAACTCAGCTCTGGTACTTTTTCATGCCTTGCAAAATGGCGTTACTGCAGCTAGCTTGAGATCGGAAGA

+

BBBBBFFFFFFFFFFFFFFFFFFFFFFFFFFFFFFFBFFFFFFFFFFFFFFFFFFFFFFFFFFFFFFFFFFFFFFFFFFFFFFFFFFFFFFFFFFFFFFFFFFFFFFFFFFFFFFFFFFFFFFFFF

@GWZHISEQ02:315:C9E6MANXX:5:1309:14924:15372

AAGCTAGCTGCAGTAACGCCATTTTGCAAGGCATGAAAAAGTACCAGAGCTGAGTTCTCAAAAGTCACAAGGAAGTTTAGTTAAAGAATAAGGCTGAACAAAACTGGGACAGGGGCCAAACAGGAT

+

BB<B<//FFFBF<FFFFFFFFF<FFFFFFF<<BFFFFF<F<FBFBFFFF<FF/<FFFF/BFBFFBFF//</<FBFFF<<F<BFFFF/FF<F/FF<FFFBFFBFFFFB<FFFF/FB<BFF//BBBFF

@GWZHISEQ02:315:C9E6MANXX:5:1314:14235:9666

AAGCTAGCTGCAGTAACGCCATTTTGCAAGGCATGAAAAAGTACCAGAGCTGAGTTCTCAAAAGTCACAAGGAAGTTTAGTTAAAGAATAAGGCTGAACAAAACTGGGACAGGGGCCAAACAGGAT

+

BBBBBFFFFFFFFFFFFFFFFFFFFFFFFFFFFFFFFFFFFFFFFFFFFFFFFFFFFFFFFFFFFFFFFFFFFFFFFFFFFFFFFFFFFFFFFFFFFFFFFFFFFFFFFFFFFFFFFFFFFFFFFF

@GWZHISEQ02:315:C9E6MANXX:5:2115:21056:33648

AAGCTAGCTGCAGTAACGCCATTTTGCAAGGCATGAAAAAGTACCAGAGCTGAGTTCTCAAAAGTCACAAGGAAGTTTAGTTAAAGAATAAGGCTGAACAAAACTGGGACAGGGGCCAAACAGGAT

+

BBBBBFFFFFFFFFFFFFFBFFFFFFFFFFFFFFFFFFFFFFFFFFFFFFFFFFF<FFFFFFFFFFFFFFFFFFF<FFFFBFFFFFFFFFFFFFFFFFFFFFFFFFFFFFFFFFFFFFFFBFFFFB

@GWZHISEQ02:315:C9E6MANXX:5:1108:18199:22165

GCTAGCTGCAGTAACGCCATTTTGCAAGGCATGAAAAAGTACCAGAGCTGAGTTCTCAAAAGTCACAAGGAAGTTTAGTTAAAGAATAAGGCTGAACAAAACTGGGACAGGGGCCAAACAGGAGAG

+

BBBBBFFFFFFFFFFFFFFFFFFFFFFFFFFFFFFFFFFFFFFFFFFFFFFFFFFFFFFFFFFFFFFFFFFFFFFFFFFFFFFFFFFFFFFFFFFFFFFFFFFFFFFFFFFFFFFFFFFFFFFFFF

@GWZHISEQ02:315:C9E6MANXX:5:2215:5750:70646

GCTAGCTGCAGTAACGCCATTTTGCAAGGCATGAAAAAGTACCAGAGCTGAGTTCTCAAAAGTCACAAGGAAGTTTAGTTAAAGAATAAGGCTGAACAAAACTGGGACAGGGGCCAAACAGGATAT

+

BBBBBFFFFFFFFFFFFFFFFFFFFFFFFFFFFFFFFFFFFFFFFFFFFFFFFFFFFFFFFFFFFFFFFFFFFFFFFFFFFFFFFFFFFFFFFFFFFFFFFFFFFFFFFFFFFFFFFFFFFFFFF<

@GWZHISEQ02:315:C9E6MANXX:5:1108:18199:22165

CTCCTGTTTGGCCCCTGTCCCAGTTTTGTTCAGCCTTATTCTTTAACTAAACTTCCTTGTGACTTTTGAGAACTCAGCTCTGGTACTTTTTCATGCCTTGCAAAATGGCGTTACTGCAGCTAGCAG

+

BBBBBFFFFFFFFFFFFFFFFFFFFFFFFFFFFFFFFFFFFFFFFFFFFFFFFFFFF<FF<FFFBFFBFF/<FFFFFFFFFFFFFFFFFFFFFFFFFFFFFFFFFF<FFFFFFFFFFFFFFFFFFF

@GWZHISEQ02:315:C9E6MANXX:5:1210:10512:66021

CTAGCTGCAGTAACGCCATTTTGCAAGGCATGAAAAAGTACCAGAGCTGAGTTCTCAAAAGTCACAAGGAAGTTTAGTTAAAGAATAAGGCTGAACAAAACTGGGACAGGGGCCAAACAGGATATC

+

BBBBBFFFFFFFFFFBFFFFFFFFFFFFFFFFFFFFFFFFFFFFFFFFFFFFFFFFFFFFFFFFFFFFFFFFFFFFFFFFFFFFFFFFFFFFFFFFFFFFFFFFFFFFFFFFFFFFFFFFFFFFFF

@GWZHISEQ02:315:C9E6MANXX:5:2215:18143:25615

CTAGCTGCAGTAACGCCATTTTGCAAGGCATGAAAAAGTACCAGAGCTGAGTTCTCAAAAGTCACAAGGAAGTTTAGTTAAAGAATAAGGCTGAACAAAACTGGGACAGGGGCCAAACAGGATATC

+

BBBBBFFFFFFFFF<BFFFFFFFFBBBBFFFFFFFFFFFFFFFFFFFFFFFFFFFFFFBFFFFFFFFFFFFFFFBFFFFFFFF/BFFFFFFFFFFFFFFFFFFFFFFFFFBFFFFFFFFFFBBFFF

@GWZHISEQ02:315:C9E6MANXX:5:1108:19561:60676

AGCTGCAGTAACGCCATTTTGCAAGGCATGAAAAAGTACCAGAGCTGAGTTCTCAAAAGTCACAAGGAAGTTTAGTTAAAGAATAAGGCTGAACAAAACTGGGACAGGGGCCAAACAGGATATCTG

+

BBBBBFFFFFFFFFFFFFFBFFFFFFFFFFFFFF<FFFBFFFFFBBBFFBFBFFFBFBFFFFBFFF<FFFFFFFFFFFFFFBFFFFFFFFF<FFFFFFFFF/FFFFF7FFBFFBFFFFF/BFFFFF

@GWZHISEQ02:315:C9E6MANXX:5:1208:10104:37920

AGCTGCAGTAACGCCATTTTGCAAGGCATGAAAAAGTACCAGAGCTGAGTTCTCAAAAGTCACAAGGAAGTTTAGTTAAAGAATAAGGCTGAACAAAACTGGGACAGGGGCCAAACAGGATATCTG

+

BBBBBFBFFFFBFFFBBFFFFF<FFBFBB//FFFFFFFFFFFFFBFFFBFFFFFFFBFFFFFFFFF<</FFFFFFFFFFFFFFFBF<<F/FFF/FFFFFFFFBFFFFFB/B7//FFFFFFFBFFF/

@GWZHISEQ02:315:C9E6MANXX:5:1210:2642:68458

AGCTGCAGTAACGCCATTTTGCAAGGCATGAAAAAGTACCAGAGCTGAGTTCTCAAAAGTCACAAGGAAGTTTAGTTAAAGAATAAGGCTGAACAAAACTGGGACAGGGGCCAAACAGGATATCTG

+

BBBBBFFFFFFFFFFFFFFFFFFFFFFFFFFFFFFFFFFFFFFFFFFFFFFFFFFFFFFFFFFFFFFFFFFFFFFFFFFFFFFFFFFFFFFBFFFFFFFFFFFFFFFFFFFFFFFFFFFFFFFFFF

@GWZHISEQ02:315:C9E6MANXX:5:2114:15063:37834

AGCTGCAGTAACGCCATTTTGCAAGGCATGAAAAAGTACCAGAGCTGAGTTCTCAAAAGTCACAAGGAAGTTTAGTTAAAGAATAAGGCTGAACAAAACTGGGACAGGGGCCAAACAGGATATCTG

+

BBBBBFFFFFFFFFFFFFFFFFFFFFFFFFFFFFFFFFFFFFFFFFFFFFFFFFFFFFFFFFFFFFFFFFFFFFFFFFFFFFFFFFFFFFFFFFFFFFFFFFFFFFFFFFFFFFFFFFFFFFFFFF

@GWZHISEQ02:315:C9E6MANXX:5:2202:8293:17784

AGCTGCAGTAACGCCATTTTGCAAGGCATGAAAAAGTACCAGAGCTGAGTTCTCAAAAGTCACAAGGAAGTTTAGTTAAAGAATAAGGCTGAACAAAACTGGGACAGGGGCCAAACAGGATATCTG

+

BBBBBFFFFFFFFBB<FFFFFFFFFFFFFFFFFFFFFFFFFFFFFFFFFFFFFFFFFFFFFFFF<FFFFBFFFFFBFFFFFBBFFFFFFFFFBBFFFF<FFFFBBFFFF<BFFFFFFFBFFFF/FF

@GWZHISEQ02:315:C9E6MANXX:5:2309:8623:76567

AGCTGCAGTAACGCCATTTTGCAAGGCATGAAAAAGTACCAGAGCTGAGTTCTCAAAAGTCACAAGGAAGTTTAGTTAAAGAATAAGGCTGAACAAAACTGGGACAGGGGCCAAACAGGATATCTG

+

BBBBBFFFFFFFFFFFFFFFFFFFFFFFFFFFFFFFFFFFFFFFFFFFFFFFFFFFFFFFFFFFFFFFFFFFFFFFFFFFFFFFFFFFFFFFFFFFFFFFFFFFFFFFFFFFFFFFFFFFFFFFFF

@GWZHISEQ02:315:C9E6MANXX:5:2314:16845:83816

AGCTGCAGTAACGCCATTTTGCAAGGCATGAAAAAGTACCAGAGCTGAGTTCTCAAAAGTCACAAGGAAGTTTAGTTAAAGAATAAGGCTGAACAAAACTGGGACAGGGGCCAAACAGGATATCTG

+

BBBBBFFFFFFFFFFFFFFFFFFFFFFFFFFFFFFFFFFFFFFFFFFFFFFFFFFFFFFFFFFFFFFFFFFFFFFFFFFFFFFFFFFFFFFFFFFFFFFFFFFFFFFFFFFFFFFFFFFFFFFFFF

@GWZHISEQ02:315:C9E6MANXX:5:2309:20805:66492

CTGCAGTAACGCCATTTTGCAAGGCATGAAAAAGTACCAGAGCTGAGTTCTCAAAAGTCACAAGGAAGTTTAGTTAAAGAATAAGGCTGAACAAAACTGGGAGATCGGAAGAGCGTCGTGTAGGGA

+

BBBBBFFFFFFFFFFFFFFFFFFFFFFFFFFFFFFFFFFFFFFFFFFFFFFFFFFFFFFFFFFFFFFFFFFFFFFFFFFFFFFFFFFFFFFFFFFFFFFFFFFFFFFFFFFFFFFFFFFFFFFFFB

@GWZHISEQ02:315:C9E6MANXX:5:1205:7224:94254

CCCCTGTCCCAGTTTTGTTCAGCCTTATTCTTTAACTAAACTTCCTTGTGACTTTTGAGAACTCAGCTCTGGTACTTTTTCATGCCTTGCAAAATGGCGTTACTGCAGATCGGAAGAGCACACGTC

+

/BBBBFFFFFFFFFFFFFFFFFFFFFFFFFFFFFFFFFFFFFFFFFFFFFFFFBFFFFFFFFFFFFFFFFFFFFF<FFFFFFFFFFFFFFFFFFFFFFFFFFFFFFFFFFFBFFBBBFFFF/FF<F

@GWZHISEQ02:315:C9E6MANXX:5:1210:10288:97270

CACAGATATCCTGTTTGGCCCCTGTCCCAGTTTTGTTCAGCCTTATTCTTTAACTAAACTTCCTTGTGACTTTTGAGAACTCAGCTCTGGTACTTTTTCATGCCTTGCAAAATGGCGTTACTGCAG

+

BBBBBFFFFFFFFFFFFFFFFFFFFFFFFFFFFFFFFFFFFFFFFFFFFFFFFFFFFFFFFFFFFFFFFFFFFFFFFFFFFFFFFFFFFFFFFFFFFFFFFBFFFFFF<FFFFFBF/FFFFFFFFF

@GWZHISEQ02:315:C9E6MANXX:5:1315:14007:90241

AACAGATATCCTGTTTGGCCCCTGTCCCAGTTTTGTTCAGCCTTATTCTTTAACTAAACTTCCTTGTGGCTTTTGAGAACTCAGCTCTGGTACTTTTTCATGCCTTGCAAAATGGCGTTACTGCAG

+

BBBBBFFFFFFFFFFFFFFFFFFFFFFFFFFFFFFFFFFFFFFFFFFFFFFFFFFFFFFFFFFFFFFF<FFFFFFFFFFFFFFFFFFFFFFFFFFFFFFFFFFFFFFFFFFFFFFFFFFFFFFFFF

@GWZHISEQ02:315:C9E6MANXX:5:2104:7050:93720

CACAGATATCCTGTTTGGCCCCTGTCCCAGTTTTGTTCAGCCTTATTCTTTAACTAAACTTCCTTGTGACTTTTGAGAACTCAGCTCTGGTACTTTTTCATGCCTTGCAAAATGGCGTTACTGCAG

+

BBBBBFFFFFFFFFFFFFFFFFFFFFFFFFFFFBBFFFFFFFFFFFFFFFFFFFFFFFFFFFFFFFFFFFFFFFFFBFFFFFFBFFFFFFFFFFFFFFFFFFFFFFFBFFFFFFFFFBFFFFFFFF

@GWZHISEQ02:315:C9E6MANXX:5:2106:21103:75904

CACAGATATCCTGTTTGGCCCCTGTCCCAGTTTTGTTCAGCCTTATTCTTTAACTAAACTTCCTTGTGACTTTTGAGAACTCAGCTCTGGTACTTTTTCATGCCTTGCAAAATGGCGTTACTGCAG

+

BBBBBFFFFFBBFBFFF<FBFFFFFBF<FBFFFFFFFFFFBFFFFFFF/FFFFFFBFFFF<FFFB<FFFFFFFFBFB/BFBFFFF<BFBFBFF<FFFF<FFBFBFFFBFFFFFFFFFFBFFFF/BF

@GWZHISEQ02:315:C9E6MANXX:5:1106:18147:16704

CACAGATATCCTGTTTGGCCCCTGTCCCAGTTTTGTTCAGCCTTATTCTTTAACTAAACTTCCTTGTGACTTTTGAGAACTCAGCTCTGGTACTTTTTCATGCCTTACATAATGTCGTTACTACAC

+

B/BBBFFFFFFBFFFFF<FFFFFFFFFFFFFFFFFFFBFFFFFFFFFF<FFFFBFFFF<FBFBFF<FFFFFFFF/<<F/FFFFF//<////</<FFFFF/B<//<F//</F<B/<///<//</<B/

@GWZHISEQ02:315:C9E6MANXX:5:2312:18027:7379

GCAGTAACGCCATTTTGCAAGGCATGAAAAAGTACCAGAGCTGAGTTCTCAAAAGTCACAAGGAAGTTTAGTTAAAGAATAAGGCTGAACAAAACTGGGACAGGGGCCAAACAGGATATCTGTGGT

+

BBBBBFFFFFFFFFFFFFFFFFFFFFFFFFFFFFFFFFFFFFFFFBFFFFFFFFFFFFFFFFFFFFFFFFFFFFFFFFFFFFFFFFFFFFFFFFFFFFFFFFFFFFFFFFFFFFFFFFFFFFFFF/

@GWZHISEQ02:315:C9E6MANXX:5:1108:10953:21747

ACCACAGATATCCTGTTTGGCCCCTGTCCCAGTTTTGTTCAGCCTTATTCTTTAACTAAACTTCCTTGTGACTTTTGAGAACTCAGCTCTGGTACTTTTTCATGCCTTGCAAAATGGCGTTACTGC

+

BBBBBFFFFFFFFFFFFFFFFFFFFFFFFFFFFFFFFFFFFFFFFFFFFFFFFFFFFFFFFFFFFFFFFFFFFFFFFFFFFFFFFFFFFFFFFFFFFFFFFFFFFFFFFFFFFFFFFFFFFFFFFF

@GWZHISEQ02:315:C9E6MANXX:5:1108:20407:14795

CAGTAACGCCATTTTGCAAGGCATGAAAAAGTACCAGAGCTGAGTTCTCAAAAGTCACAAGGAAGTTTAGTTAAAGAATAAGGCTGAACAAAACTGGGACAGGGAGATCGGAAGAGCGTCGTGTAG

+

BBBBBFFFFFFFFFFFFFFFFFFFFFFFFFFFFFFFFFFFFFFFFFFFFFFFFFFFFFFFFFFFFFFFFFFFFFFFFFFFFFFFFFFFFFFFFFFFFFFFFFFFFFFFFFBFFFFFFFFFF7BBFF

@GWZHISEQ02:315:C9E6MANXX:5:1306:1643:48303

CAGTAACGCCATTTTGCAAGGCATGAAAAAGTACCAGAGCTGAGTTCTCAAAAGTCACAAGGAAGTTTAGTTAAAGAATAAGGCTGAACAAAACTGGGACAGGGGCCAAACAGGATATCTGTGGTC

+

BBBBBFFFFFFFFBFFBFFFFFF<//<FFFBFFFB<FFFFFF/F/FFFFFBFBFB/F<FBFBFFBF/FFFFFBFFFFFFFFB//BFFFFFFFFFFFBFF<FBFFFBFFF<FFFFFFBF<FFFF<7<

@GWZHISEQ02:315:C9E6MANXX:5:1307:11760:60820

CAGTAACGCCATTTTGCAAGGCATGAAAAAGTACCAGAGCTGAGTTCTCAAAAGTCACAAGGAAGTTTAGTTAAAGAATAAGGCTGAACAAAACAGATCGGAAGAGCGTCGTGTAGGGAAAGAGTG

+

BBBBBFFFFFFFFFFFFFFFFFFFFFFFFFFFFFFFFFFFFFFFFFFFFFFFFFFFFFFFFFFFFFFFFFFFFFFFFFFFFFFFFFFFFFFFFFFFFFFFFFFFFFFFFFFFFFFFFFFFFFFFFF

@GWZHISEQ02:315:C9E6MANXX:5:2304:2985:33382

CAGTAACGCCATTTTGCAAGGCATGAAAAAGTACCAGAGCTGAGTTCTCAAAAGTCACAAGGAAGTTTAGTTAAAGAATAAGGCTGAACAAAACTGGGACAGGGAGATCGGAAGAGCGTCGTGTAG

+

BBBBBFFFFFFFFFFFFFFFFFFFFFFFFFFFFFFFFFFFFFFFFFFFFFFFFFFFFFFFFFFFFFFFFFFBFFFFFFFFFFFFFFFFFFFFFFFFFFFFFFFFFFFFFFFFFFFFFFFFFFFFFF

@GWZHISEQ02:315:C9E6MANXX:5:1108:20407:14795

CCCTGTCCCAGTTTTGTTCAGCCTTATTCTTTAACTAAACTTCCTTGTGACTTTTGAGAACTCAGCTCTGGTACTTTTTCATGCCTTGCAAAATGGCGTTACTGAGATCGGAAGAGCACACGTCTG

+

<BBBBFFFFFFFFFFFFFBF<FFFFFFFFFFFFFFFBBBFFFFFFFFFFFFFFFFFFFBFFFBFFFFFFFFFFFFFFFFFFFFFFFFFFFBFBFFFFFFFFFFFFFFFFBFFBFFFFFFFFFFFFF

@GWZHISEQ02:315:C9E6MANXX:5:1307:11760:60820

GTTTTGTTCAGCCTTATTCTTTAACTAAACTTCCTTGTGACTTTTGAGAACTCAGCTCTGGTACTTTTTCATGCCTTGCAAAATGGCGTTACTGAGATCGGAAGAGCACACGTCTGAACTCCAGTC

+

BBBBBFFFFFFFFFFFFFFFFFFFFFFFFFFFFFFFFFFFFFFFFFFFFFFFFFFFFFFFFFFFFFFFFFFFFFFFFFFFFFFFFFFFFFFFFFFFFFFFFFFFFFF<FFFFFFFFFFFFFFFFFF

@GWZHISEQ02:315:C9E6MANXX:5:2304:2985:33382

CCCTGTCCCAGTTTTGTTCAGCCTTATTCTTTAACTAAACTTCCTTGTGACTTTTGAGAACTCAGCTCTGGTACTTTTTCATGCCTTGCAAAATGGCGTTACTGAGATCGGAAGAGCACACGTCTG

+

BBBBBFFFFFFFFFFFFFFFFFFBFFFFFFFFFFFFFFFFFFFFFFFFFFFFFFFFFFFFFFFFFFFFFFFFFFFFFFFFBFFFFFFFFFFFFFFFFFFFFFFFFFFFFFFFFFFFFFF<FFFFFF

@GWZHISEQ02:315:C9E6MANXX:5:1107:6726:29354

AGTAACGCCATTTTGCAAGGCATGAAAAAGTACCAGAGCTGAGTTCTCAAAAGTCACAAGGAAGTTTAGTTAAAGAATAAGGCTGAACAAAACTGGGACAGGGGCCAAACAGGATATCTGTGGTCG

+

BBBBBFFFFFFFFFFFFFFFFFFFFFFFFFFFFFFFFFFFFFFFFFFFFFFFFFFFFFFFFFFFFFFFFFFFFFFFFFFFFFFFFFFFFFFFFFFFFFFFFFFFFFFFFFFFFFFFFFFFFFFFFF

@GWZHISEQ02:315:C9E6MANXX:5:1215:20446:29524

AGTAACGCCATTTTGCAAGGCATGAAAAAGTACCAGAGCTGAGTTCTCAAAAGTCACAAGGAAGTTTAGTTAAAGAATAAGGCTGAACAAAACTGGGACAGGGGCCAAACAGGATATCTGTGGTCG

+

BBBBBFFFFFFFFFFFFFFFFFFFFFFFFFFFFFFFFFFFFFFFFFFFFFFFBFFFFFFFFFFFFFFFFFFFFFFFFFFFFFFFFFFFFFFFFFFFFFFFFFFFFFFFFFFFFFFFBFFFFFFFFF

@GWZHISEQ02:315:C9E6MANXX:5:1216:7921:49564

AGTAACGCCATTTTGCAAGGCATGAAAAAGTACCAGAGCTGAGTTCTCAAAAGTCACAAGGAAGTTTAGTTAAAGAATAAGGCTGAACAAAACTGGGACAGGGGCCAAACAGGATATCTGTGGTCG

+

BBBBBFFFFFFFFFFFFFFFFFFFFFFFFFFFFFFFFFFFFFFFFBFFFFFFFFFFFFFFFFFFFFFFFFFFFFFFFFFFFFFFFFFFFFFFFFFBFFFFFFFFFFFFFFFFFFFFFFFFFFFFFF

@GWZHISEQ02:315:C9E6MANXX:5:2215:21304:88701

CGACCACAGATATCCTGTTTGGCCCCTGTCCCAGTTTTGTTCAGCCTTATTCTTTAACTAAACTTCCTTGTGACTTTTGAGAACTCAGCTCTGGTACTTTTTCATGCCTTGCAAAATGGCGTTACT

+

BBBBBFBFBBFFFBF/F<FFF<FFFFFFFFFFFFFFFFFFFFFFBFFFFF/BFFFFFFFFFFFFFFFFFFFFFFFFFFFFFFFFFBFF/<F/FFFFFFFFFFFFFFFFBFFBFFFFFFFFBFFFBF

@GWZHISEQ02:315:C9E6MANXX:5:2302:5717:80903

CGACCACAGATATCCTGTTTGGCCCCTGTCCCAGTTTTGTTCAGCCTTATTCTTTAACTAAACTTCCTTGTGACTTTTGAGAACTCAGCTCTGGTACTTTTTCATGCCTTGCAAAATGGCGTTACT

+

BBBBBFFFFFFFFF<FFFFFFFFFFFFFFFFFFFBFFF/FF<FFFFFFFFFFFFFBFFFBFFB<<FFFFFF/<<FFFF<<F/FBFFB</FFFFFFFFFFFFBBFFF<BFFFFF/<FFF//</B<BF

@GWZHISEQ02:315:C9E6MANXX:5:2315:17183:23612

TAACGCCATTTTGCAAGGCATGAAAAAGTACCAGAGCTGAGTTCTCAAAAGTCACAAGGAAGTTTAGTTAAAGAATAAGGCTGAACAAAACTGGGACAGGGGCCAAACAGGAGATCGGAAGAGCGT

+

BBBBBFFFFFFFFFFFFFFFFFFFFFFFFFFFFFFFFFFFFFFFFFFFFFFFFFFFFFFFFFFFFFFFFFFFFFFFFFFFFFFFFFFFFFFFFFFFFFFFFFFFFFFFFFFFFFFFFFFFFFFFF7

@GWZHISEQ02:315:C9E6MANXX:5:1106:5891:4362

CTCGACCACAGATATCCTGTTTGGCCCCTGTCCCAGTTTTGTTCAGCCTTATTCTTTAACTAAACTTCCTTGTGACTTTTGAGAACTCAGCTCTGGTACTTTTTCATGCCTTGCAAAATGGCGTTA

+

BBBBBFFFFFFFFFFFFFFFFFFFFFFFFFFFFFFFFFFFFFFFFFFFFFFFFFFFFFFFFFFFFFFFFFFFFFFFFFFFFFFFFFFFFFFFFFFFFFFFFFFFFFFFFFFFFFFFFFFFFFFFFF

@GWZHISEQ02:315:C9E6MANXX:5:1111:15518:5195

CTCGACCACAGATATCCTGTTTGGCCCCTGTCCCAGTTTTGTTCAGCCTTATTCTTTAACTAAACTTCCTTGTGACTTTTGAGAACTCAGCTCTGGTACTTTTTCATGCCTTGCAAAATGGCGTTA

+

BBBBBFFFFFFFFFFFFFFFFFFFFFFFFFFFFFFFFFFFFFFFFFFFFFFFFFFFFFFFFFFFFFFFFFFFFFFFFFFFFFFFFFFFFFFFFFFFFFFFFFFFFFFFFFFFFFFFFFFFFFFFF<

@GWZHISEQ02:315:C9E6MANXX:5:2211:14222:5615

CTCGACCACAGATATCCTGTTTGGCCCCTGTCCCAGTTTTGTTCAGCCTTATTCTTTAACTAAACTTCCTTGTGACTTTTGAGAACTCAGCTCTGGTACTTTTTCATGCCTTGCAAAATGGCGTTA

+

BBBBBFFFFFFFFFFFFFFFFFFFFFFFFFFFFFFFFFFFFFFFFFFFFFFFFFFFFFFFFFFFFFFFFFFFFFFFFFFFFFFFFFFFFFFFFFFFFFFFFFFFFFFFFFFFFFFFFFFFFFFFFF

@GWZHISEQ02:315:C9E6MANXX:5:2212:6418:14756

CTCGACCACAGATATCCTGTTTGGCCCCTGTCCCAGTTTTGTTCAGCCTTATTCTTTAACTAAACTTCCTTGTGACTTTTGAGAACTCAGCTCTGGTACTTTTTCATGCCTTGCAAAATGGCGTTA

+

BBBBBFFFFFFFFFFFFFFFFFFFFFFFFFFFFFFFFFFFFFFFFFFFFFFFFFFFFFFFFFFFFFFFFFFFFFBFFFFFFFFFFFFFFFFFFFFFFFFFFFFFFFFFFFFFFFFFFFFFFFFFF/

@GWZHISEQ02:315:C9E6MANXX:5:2315:17183:23612

CCTGTTTGGCCCCTGTCCCAGTTTTGTTCAGCCTTATTCTTTAACTAAACTTCCTTGTGACTTTTGAGAACTCAGCTCTGGTACTTTTTCATGCCTTGCAAAATGGCGTTAAGATCGGAAGAGCAC

+

BBBBBFFFFFFFFFFFFFFFFFFFFFFFFFFFFFFFFFFFFFFFFFFFFFFFFFFFFFFFFFFFFFFFFFFFFFFFFFFFFFFFFFFFFFFFFFFFFFFFFFFFFFFFFFFFFFFFFFFFFFFFFF

@GWZHISEQ02:315:C9E6MANXX:5:1103:15717:41997

AACGCCATTTTGCAAGGCATGAAAAAGTACCAGAGCTGAGTTCTCAAAAGTCACAAGGAAGTTTAGTTAAAGAATAAGGCTGAACAAAACTGGGACAGGGGCCAAACAGGATATCTGTGGTCGAGC

+

BBBBBFFFFFFFFFFFFFFFFFFFFFFFFFFFFFFFFFFFFFFFFFFFFFFFFFFFFFFFFFFFFFFFFFFFFFFFFFFFFFFFFFFFFFFFFFFFFFFFFFFFFFFFFFFFFFFFFFFFFFFFFF

@GWZHISEQ02:315:C9E6MANXX:5:1104:18595:5991

AACGCCATTTTGCAAGGCATGAAAAAGTACCAGAGCTGAGTTCTCAAAAGTCACAAGGAAGTTTAGTTAAAGAATAAGGCTGAACAAAACTGGGACAGGGGCCAAACAGGATATCTGTGGTCGAGC

+

BBBBBFFFFFFFFFFFFFFFFFFFFFFFFFFFFFFFFFFFFFFFFFFFFFFFFFFFFFFFFFFFFFFFFFFFFFFFFFFFFFFFFFFFFFFFBFFFFFFFFFFFFFFFFFFFFFBFFFFFFFFFF<

@GWZHISEQ02:315:C9E6MANXX:5:1105:10617:47337

AACGCCATTTTGCAAGGCATGAAAAAGTACCAGAGCTGAGTTCTCAAAAGTCACAAGGAAGTTTAGTTAAAGAATAAGGCTGAACAAAACTGGGACAGGGGCCAAACAGGATATCTGTGGTCGAGC

+

BBBBBFFFFFFFFFFFFFFFFFFFFFFFFFFFFFFFFFFFFFFFFFFFFFFFFFFFFFFFFFFFFFFFFFFFFFFFFFFFFFFFFFFFFFFFFFFFFFFBFFFFFFFFFFFFFFFFFFFFFFFFFF

@GWZHISEQ02:315:C9E6MANXX:5:1111:6213:12777

AACGCCATTTTGCAAGGCATGAAAAAGTACCAGAGCTGAGTTCTCAAAAGTCACAAGGAAGTTTAGTTAAAGAATAAGGCTGAACAAAACTGGGACAGGGGCCAAACAGGATATCTGTGGTCGAGC

+

BBBBBFFBFFFFFFFFFFFFFFFFFFFFFFFFFFFFFFFFFFFFFFFFFFFFFFFFFFFFFFFFFFFFFFFFFFFFFFFFFFFFFFBFFFFB<FFFFFFF<<FFFFFFFFFFFFFBFBFFFFFFFF

@GWZHISEQ02:315:C9E6MANXX:5:2107:15856:18514

AACGCCATTTTGCAAGGCATGAAAAAGTACCAGAGCTGAGTTCTCAAAAGTCACAAGGAAGTTTAGTTAAAGAATAAGGCTGAACAAAACTGGGACAGGGGCCAAACAGGATATCTGTGGTCGAGC

+

BBBBBFFFFFFFFFFFFFFFFFFFFFFFFFFFFFFFFFFFFFFFFFFFFFFFFFFFFFFFFFFFFFFFFFFFFFFFFFFFFFFFFFFFFFFFFFFFFFFFFFFFFFFFFFFFFFFFFFFFFFFFFF

@GWZHISEQ02:315:C9E6MANXX:5:2202:5267:32046

AACGCCATTTTGCAAGGCATGAAAAAGTACCAGAGCTGAGTTCTCAAAAGTCACAAGGAAGTTTAGTTAAAGAATAAGGCTGAACAAAACTGGGACAGGGGCCAAACAGGATATCTGTGGTCGAGC

+

BBBBBFFFFFFFFFFFFFFFFFFFFFFFFFFFFFFFFFFFFFFFFFFFFFFFFFFFFFFFFFFFFFFFFFFFFFFFFFFFFFFFFFFFFFFFFFFFFFFFFFFFFFFFFFFFFFFFFFFFFFFBFF

@GWZHISEQ02:315:C9E6MANXX:5:2203:2221:46029

AACGCCATTTTGCAAGGCATGAAAAAGTACCAGAGCTGAGTTCTCAAAAGTCACAAGGAAGTTTAGTTAAAGAATAAGGCTGAACAAAACTGGGACAGGGGCCAAACAGGATATCTGTGGTCGAGC

+

BBBBBFFFFFFFFFFFFFFFFFFFFFFFFFFFFFFFFFFFFFFFFFFFFFFFFFFFFFFFFFFFFFFFFFFFFFFFFFFFFFFFFFFFFFFFFFFFFFFFFFFFFFFFFFFFFFFFFFFFFFFFF<

@GWZHISEQ02:315:C9E6MANXX:5:2214:1949:21885

AACGCCATTTTGCAAGGCATGAAAAAGTACCAGAGCTGAGTTCTCAAAAGTCACAAGGAAGTTTAGTTAAAGAATAAGGCTGAACAAAACTGGGACAGGGGCCAAACAGGATATCTGTGGTCGAGC

+

BBBBBFFFFFFFFFFFFFFFFFFFFFFFFFFFFFFFFFFFFFFFFFFFFFFFFFFFFFFFFFFFFFFFFFFFFFFFFFFFFFFFFFFFFFFFFFFBFFFFFFFFFFFBFFFFFFFFFFFFFFFFFF

@GWZHISEQ02:315:C9E6MANXX:5:2201:13492:44700

ACGCCATTTTGCAAGGCATGAAAAAGTACCAGAGCTGAGTTCTCAAAAGTCACAAGGAAGTTTAGTTAAAGAATAAGGCTGAACAAAACTGGGACAGGGGCCAAACAGGATATCTGTGGTCGAGCA

+

BBBBBFFFFFFFFFFFFFFFFFFFFFFFFFFFFFFFFFFFFFFFFFFFFFFFFFFFFFFFFFFFFFFFFFFFFFFFFFFFFFFFFFFFFFFFFFFFFFFFFFFFFFFFFFFFFFFFFFFFFFFFFB

@GWZHISEQ02:315:C9E6MANXX:5:2312:7790:62311

ACGCCATTTTGCAAGGCATGAAAAAGTACCAGAGCTGAGTTCTCAAAAGTCACAAGGAAGTTTAGTTAAAGAATAAGGCTGAACAAAACTGGGACAGGGGCCAAACAGGATATCTGTGGTCGAGCA

+

BBBBBFFFFFFFFFFFFFFFFFFFFFFFFFFFFFFFFFFFFFFFFFFFFFFFFFFFFFFFFFFFFFFFFFFFFFFFFFFFFFFFFFFFFFFFFFFFFFFFFFFFFFFFFFFFFFFFFFFFFFFFFB

@GWZHISEQ02:315:C9E6MANXX:5:1313:10142:62604

CGCTCGACCACAGATATCCTGTTTGGCCCCTGTCCCAGTTTTGTTCAGCCTTATTCTTTAACTAAACTTCCTTGTGACTTTTGAGAACTCAGCTCTGGTACTTTTTCATGCCTTGCAAAATGGCGT

+

BBBBBFFFFFFFFFFFFFFFFFFFFFFFFFFFFFFFFFFFFFFFFFFFFFFFFFFFFFFFFFFFFFFFFFFFFFFFFFFFFFFFFFFFFFFFFFFFFFFFFFFFFFFFFFFFFFFFFFFFFFFFFF

@GWZHISEQ02:315:C9E6MANXX:5:1105:19756:83507

CGCCATTTTGCAAGGCATGAAAAAGTACCAGAGCTGAGTTCTCAAAAGTCACAAGGAAGTTTAGTTAAAGAATAAGGCTGAACAAAACTGGGACAGGGGCCAAACAGGATATCTGTGGTCGAGCAC

+

BBBBBFFFFFFFFFFFFFFFFFFFFFFFFFFFFFFFFFFFFFFFFFFFFFFFFFFFFFFFFFFFFFFFFFFFFFFFFFFFFFFFFFFFFFFFFFFFFFFFFFFFFFFFFFFFFFFFFFFFFFFFFF

@GWZHISEQ02:315:C9E6MANXX:5:1106:20186:58223

CGCCATTTTGCAAGGCATGAAAAAGTACCAGAGCTGAGTTCTCAAAAGTCACAAGGAAGTTTAGTTAAAGAATAAGGCTGAACAAAACTGGGAAGATCGGAAGAGCGTCGTGTAGGGAAAGAGTGT

+

BBBBBFFFFFFFFFFFFFFFFFFFFFFFFFFFFFFFFFFFFFFFFFFFFFFFFFFFFFFFFFFFFFBFFFBFFFFFFFFFFFFFFFFFFFFFBFFFFFFFFFFBFFFBFFBFF/FFFB<FFFFFFF

@GWZHISEQ02:315:C9E6MANXX:5:1107:10768:80936

CGCCATTTTGCAAGGCATGAAAAAGTACCAGAGCTGAGTTCTCAAAAGTCACAAGGAAGTTTAGTTAAAGAATAAGGCTGAACAAAACTGGGACAGGGGCCAAACAGGATATCTGTGGTCGAGCAC

+

BBBBBFFFFFFFFFFFFFFFFFFFFFFFFFFFFFFFFFFFFFFFFFFFFFFFFFFFFFFFFFFFFFFFFFFFFFFFFFFFFFFFFFFFFFFFFFFFFFFFFFFFFFFFFFFFFFFFFFFFFFFFFF

@GWZHISEQ02:315:C9E6MANXX:5:1113:8097:94569

CGCCATTTTGCAAGGCATGAAAAAGTACCAGAGCTGAGTTCTCAAAAGTCACAAGGAAGTTTAGTTAAAGAATAAGGCTGAACAAAACTGGGACAGGGGCCAAACAGGATATCTGTGGTCGAGCAC

+

BBBBBFFFFFFFFFFFFFFFFFFFFFFFFFFFFFFFFFFFFFFFFFFFFFFFFFFFFFFFFFFFFFFFFFFFFFFFFFFFFFFFFFFFFFFFFFFFFFFFFFFFFFFFFFBFFFFFFFFFFFFFFF

@GWZHISEQ02:315:C9E6MANXX:5:1207:16670:6573

CGCCATTTTGCAAGGCATGAAAAAGTACCAGAGCTGAGTTCTCAAAAGTCACAAGGAAGTTTAGTTAAAGAATAAGGCTGAACAAAACTGGGACAGGGGCCAAACAGGATATCTGTGGTCGAGCAC

+

BBBBBFFFFFFFFFFFFFFFFFFFFFFFFFFFFFFFFFFFFFFFFFFFFFFFFFFFFFFFFFFFFFFFFFFFFFFFFFFFFFFFFFFFFFFFFFFFFFFFFFFFFFFFFFFFFFFFFFFFFFFFFF

@GWZHISEQ02:315:C9E6MANXX:5:1212:15283:29145

CGCCATTTTGCAAGGCATGAAAAAGTACCAGAGCTGAGTTCTCAAAAGTCACAAGGAAGTTTAGTTAAAGAATAAGGCTGAACAAAACTGGGACAGGGGCCAAACAGGATATCTGTGGTCGAGCAC

+

BBBBBFFFFFFFFFFFFFFFFFFFFFFFFFFFFFFFFFFFFFFF<FFFFFFFFFFFFFFFFFFFFFFFFFFFFFFFFFFFFFFFFFFFFFFFFFFFFFFFFFFBFFFFFFFFFFFFFFFFFFFFFF

@GWZHISEQ02:315:C9E6MANXX:5:1216:15806:93614

CGCCATTTTGCAAGGCATGAAAAAGTACCAGAGCTGAGTTCTCAAAAGTCACAAGGAAGTTTAGTTAAAGAATAAGGCTGAACAAAACTGGGACAGGGGCCAGATCGGAAGAGCGTCGTGTAGGGA

+

BBBBBFFFFFFFFFFFFFFFFFFFFFFFFFFFFFFFFFFFFFFFFFFFFFFFFFFFFFFFFFFFFFFFFFFFFFFFFFFFFFFFFFFFFFFFFFFFFFFFFFFFFFFFFFFBFFFFFFBFBFFFBB

@GWZHISEQ02:315:C9E6MANXX:5:1302:1728:25517

CGCCATTTTGCAAGGCATGAAAAAGTACCAGAGCTGAGTTCTCAAAAGTCACAAGGAAGTTTAGTTAAAGAATAAGGCTGAACAAAACTGGGACAGGGGCCAAACAGGATATCTGTGGTCGAGCAC

+

BBBBBFFFFFFFFFFFFFFFFFFFFFFFFFFFFFFFFFFFFFFFFFFFFFFFFFFFFFFFFFFFFFFFFFFFFFFFFFFFFFFFFFFFFFFFFFFFFFFFFFFFFFFFFFFFFFFFFFFFFFFFF<

@GWZHISEQ02:315:C9E6MANXX:5:1304:5022:23101

CGCCATTTTGCAAGGCATGAAAAAGTACCAGAGCTGAGTTCTCAAAAGTCACAAGGAAGTTTAGTTAAAGAATAAGGCTGAACAAAACTGGGACAGGGGCCAAACAGGATATCTGTGGTCGAGCAC

+

BBBBBFFFFFFFFFFFFFFFFFFFFFFFFFFFFFFFFFFFFFFFFFFFFFFFFFFFFFFFFFFFFFFFFFFFFFFFFFFFFFFFFFFFFFFFFFFFFFFFFFFFFFFFFFFFFFFFFFFFFBFFF<

@GWZHISEQ02:315:C9E6MANXX:5:1311:7263:22178

CGCCATTTTGCAAGGCATGAAAAAGTACCAGAGCTGAGTTCTCAAAAGTCACAAGGAAGTTTAGTTAAAGAATAAGGCTGAACAAAACTGGGACAGGGGCCAAACAGGATATCTGTGGTCGAGCAC

+

BBBBBFFFFFFFFFFFFFFFFFFFFFFFFFFFFFFFFFFFFFFFFFFFFFFFFFFFFFFFFFFFFFFFFFFFFFFFFFFFFFFFFFFFFFFFFFFFFFFFFFFFFFFFFFFFFFFFFFFFFFFFFF

@GWZHISEQ02:315:C9E6MANXX:5:1311:6907:96522

CGCCATTTTGCAAGGCATGAAAAAGTACCAGAGCTGAGTTCTCAAAAGTCACAAGGAAGTTTAGTTAAAGAATAAGGCTGAACAAAACTGGGACAGGGGCCAAACAGGATATCTGTGGTCGAGCAC

+

BBBBBFFFFFFFFFFFFFFFFFFFFFFFFFFFFFFFFFFFFFFFFFFFFFFFFFFFFFFFFFFFFFFFFFFFFFFFFFFFFFFFFFFFFFFFFFFFFFFFFFFFFFFFFFFFFFFFFFFFFFFFFF

@GWZHISEQ02:315:C9E6MANXX:5:1312:3068:61381

CGCCATTTTGCAAGGCATGAAAAAGTACCAGAGCTGAGTTCTCAAAAGTCACAAGGAAGTTTAGTTAAAGAATAAGGCTGAACAAAACTGGGACAGGGGCCAAACAGGATATCTGTGGTCGAGCAC

+

BBBBBFFFFFFFFFFFFFFFFFFFFFFFFFFFFFFFFFFFFFFFFFFFFFFFFFFFBFFFFFFFFFFFFFFFFFFFFFFFFFFFFFFFFFFFFFFFFFFFFFFFFFFFFFFFFFFFFFFFFFFFFF

@GWZHISEQ02:315:C9E6MANXX:5:1313:19540:93948

CGCCATTTTGCAAGGCATGAAAAAGTACCAGAGCTGAGTTCTCAAAAGTCACAAGGAAGTTTAGTTAAAGAATAAGGCTGAACAAAACTGGGACAGGGGCCAAACAGGATATCTGTGGTCGAGAGA

+

BBBBBFFFFFFFFFFFFFFFFFFFFFFFFFFFFFFFFFFFFFFFFFFFFFFFFFFFFFFFFFFFFFFFFFFFFFFFFFFFFFFFFFFFFFFFFFFFFFFFFFFFFFFFFFFFFFFFFFFFFFFFFF

@GWZHISEQ02:315:C9E6MANXX:5:1314:7031:58369

CGCCATTTTGCAAGGCATGAAAAAGTACCAGAGCTGAGTTCTCAAAAGTCACAAGGAAGTTTAGTTAAAGAATAAGGCTGAACAAAACTGGGACAGGGGCCAAACAGGATATCTGTGGTCGAGCAC

+

BBBBBFFFFFFFFFFFFFFFFFFFFFFFFFFFFFF<FFFFFFFFFFFFFFFFFFFFFFFFFFFFFFFFFFFFFFFFFFFFFFFFFFFFFFFFFFFF<FFFFFFFFFFFFFFFFFFFFFFFFFFFFF

@GWZHISEQ02:315:C9E6MANXX:5:1316:6899:38926

CGCCATTTTGCAAGGCATGAAAAAGTACCAGAGCTGAGTTCTCAAAAGTCACAAGGAAGTTTAGTTAAAGAATAAGGCTGAACAAAACTGGGACAGGGGCCAAACAGGATATCTGTGGTCGAGCAC

+

BBBBBFFFFFFFFFFFFFFFFFFFFFFFFFFFFFFFFFFFFFFFFFFF<FFFFFFFFFB/FBFFBFFFFFFFFFFFBFBFFFFFBFFFFFBFFFFFFFFFFFFFFFFFFFFFFFFFFFFFFFFFFF

@GWZHISEQ02:315:C9E6MANXX:5:1316:15067:71232

CGCCATTTTGCAAGGCATGAAAAAGTACCAGAGCTGAGTTCTCAAAAGTCACAAGGAAGTTTAGTTAAAGAATAAGGCTGAACAAAACTGGGACAGGGGCCAAACAGGATATCTGTGGTCGAGCAC

+

BBBBBFFFFFFFFFFFFFFFFFFFFFFFFFFFFFFFFFFFFFFFFFFFFFFFFFFFFFFFFFFFFFFFFFFFFFFFFFFFFFFFFFFFFFFFFFFFFFFFFFFFFFFFFFFFFFFFFFFFFFFFFF

@GWZHISEQ02:315:C9E6MANXX:5:2101:17617:39420

CGCCATTTTGCAAGGCATGAAAAAGTACCAGAGCTGAGTTCTCAAAAGTCACAAGGAAGTTTAGTTAAAGAATAAGGCTGAACAAAACTGGGACAGGGGCCAAACAGGATATCTGTGGTCGAGCAC

+

BBBBBFFFFFFFFFFFFFFFFFFFFFFFFFFFFFFFFFFFFFFFFFFFFFFFFFFFFFFB/FFFFFFFFFFFFFFFFFFFFFFFFFFFFFFFFFFFFFFFFFFFFFFFFFFFFFFFFFFFFFFFFB

@GWZHISEQ02:315:C9E6MANXX:5:2105:16844:98102

CGCCATTTTGCAAGGCATGAAAAAGTACCAGAGCTGAGTTCTCAAAAGTCACAAGGAAGTTTAGTTAAAGAATAAGGCTGAACAAAACTGGGACAGGGGCCAAACAGGATATCTGTGGTCGAGCAC

+

BBBBBFFFFFFFFFFFFFFFFFFFFFFFFFFFFFFFFFFFFFFFFFFFFFFFFFFFFFFFFFFFFFFFFFFFFFFFFFFFFFFFFFFFFFFFFFFFFFFFFFFFFFFFFFFFFFFFFFFFFFFFFF

@GWZHISEQ02:315:C9E6MANXX:5:2107:2249:4108

CGCCATTTTGCAAGGCATGAAAAAGTACCAGAGCTGAGTTCTCAAAAGTCACAAGGAAGTTTAGTTAAAGAATAAGGCTGAACAAAACTGGGACAGGGGCCAAACAGGATATCTGTGGTCGAGCAC

+

BBBBBFFFFFFFFFFFFFFFFFFFFFFFFFFFFFFFFFFFFFFFFFFFFFFFFFFFFFFFFFFFFFFFFFFFFFFFFFFFFFFFFFFFFFFFFFFFFFFFFFFFFFFFFFFFFFFFFFFFFFFFFF

@GWZHISEQ02:315:C9E6MANXX:5:2107:3185:5395

CGCCATTTTGCAAGGCATGAAAAAGTACCAGAGCTGAGTTCTCAAAAGTCACAAGGAAGTTTAGTTAAAGAATAAGGCTGAACAAAACTGGGACAGAGAGATCGGAAGAGCGTCGTGTAGGGAAAG

+

BBBBBFFFFFFFFFFFFFFFFFFFFFFFFFFFFFFFFFFFFFFFFFFFFFFFFFFFFFFFFFFFFFFFFFFFFFFFFFFFFFFFFFFFFFFFFFFFFFFFFFFFFFFFFFFFFBBBFFFFFFBFFF

@GWZHISEQ02:315:C9E6MANXX:5:2107:1773:44719

CGCCATTTTGCAAGGCATGAAAAAGTACCAGAGCTGAGTTCTCAAAAGTCACAAGGAAGTTTAGTTAAAGAATAAGGCTGAACAAAACTGGGACAGGGGCCAAACAGGATATCTGTGGTCGAGCAC

+

BBBBBFFFFFFFFFFFFFFFFFFFFFFFFFFFFFFFFFFFFFFFFFFFFFFFFFFFFFFFFFFFFFFFFFFFFFFFFFFFFFFFFFFFFFFFFFFFFFFFFFFFFFFFFFFFFFFFFFFFFFFFFF

@GWZHISEQ02:315:C9E6MANXX:5:2113:17913:77591

CGCCATTTTGCAAGGCATGAAAAAGTACCAGAGCTGAGTTCTCAAAAGTCACAAGGAAGTTTAGTTAAAGAATAAGGCTGAACAAAACTGGGACAGGGGCCAAACAGGATATCTGTGGTCGAGCAC

+

BBBBBFFFFFFFFFFFFFFFFFFFFFFFFFFFFFFFFFFFFFFFFFFFFFFFFFFFFFFFFFFFFFFFFFFFFFFFFFFFBFFFFFFFFFFFFFFFFFFFFFFFFFFFFFFFFFFFFFFFFFFFFF

@GWZHISEQ02:315:C9E6MANXX:5:2204:19527:15805

CGCCATTTTGCAAGGCATGAAAAAGTACCAGAGCTGAGTTCTCAAAAGTCACAAGGAAGTTTAGTTAAAGAATAAGGCTGAACAAAACTGGGACAGGGGCCAAACAGGATATCTGTGGTCGAGCAC

+

BBBBBFFFFFFFFFFFFFFFFFFFFFFFFFFFFFFFFFFFFFFFFFFFFFFFFFFFFFFFFFFFFFFFFFFFFFFFFFFFFFFFFFFFFFFFFFFFFFFFFFFFFFFFFFFFFFFFFFFFFFFFFF

@GWZHISEQ02:315:C9E6MANXX:5:2208:17557:66807

CGCCATTTTGCAAGGCATGAAAAAGTACCAGAGCTGAGTTCTCAAAAGTCACAAGGAAGTTTAGTTAAAGAATAAGGCTGAACAAAACTGGGGCAGGGGCCAAACAGGATATCTGTGGTCGAGCAC

+

BBBBBFFFFFFFFFFFFFFFFFFFFFFFFFFFFFFFFFFFFFFFFFFFFFFFFFFFFFFFFFFFFFFFFFFFFFFFFFFFFFFFFFFFFFFF/FFFFFFFFFFFFFFFFFFFFFFFFFFFFFFFFF

@GWZHISEQ02:315:C9E6MANXX:5:2210:9813:28918

CGCCATTTTGCAAGGCATGAAAAAGTACCAGAGCTGAGTTCTCAAAAGTCACAAGGAAGTTTAGTTAAAGAATAAGGCTGAACAAAACTGGGACAGGGGCCAAACAGGATATCTGTGGTCGAGCAC

+

BB/B/FFFF<<//<//BFB//<BBFFFF/B<BFFFFFBFFBFFFFF/BF<<FFFF<BBFF<BFB<<</FFFFBFFFFFFFBF//<BFFFFFFBFBFFFFF/F/BBBFFFBFFFF/FBBFFFF<FFF

@GWZHISEQ02:315:C9E6MANXX:5:2211:2926:71016

CGCCATTTTGCAAGGCATGAAAAAGTACCAGAGCTGAGTTCTCAAAAGTCACAAGGAAGTTTAGTTAAAGAATAAGGCTGAACAAAACTGGGACAGGGGCCAAACAGGATATCTGTGGTCGAGCAC

+

BBBBBFFFFFFFBFFFFFFFFFFFFFFFFFFFFFFFFFFFFFFFFFFFFFFFFFFFFFFFFFFFFFFFFFFFFFFFFFFFFFFFFFFFFFFFFFFFFFFFFFFFFFFFFFFFFFFFFFFFFFFFFF

@GWZHISEQ02:315:C9E6MANXX:5:2211:2404:83883

CGCCATTTTGCAAGGCATGAAAAAGTACCAGAGCTGAGTTCTCAAAAGTCACAAGGAAGTTTAGTTAAAGAATAAGGCTGAACAAAACTGGGACAGGAGCCAGATCGGAAGAGCGTCGTGTAGGGA

+

BBBBBFFFFFFFFFFFFFFFFFFFFFFFFFFFFFFFFFFFFFFFFFFFFFFFFFFFFFFFBFFFFFFFFFFFFFFFFFFFFFFFFFFFFFFFFFFFFFFFFFFFFFFFFFFFFFFFFFFFFFFFF<

@GWZHISEQ02:315:C9E6MANXX:5:2212:2082:30005

CGCCATTTTGCAAGGCATGAAAAAGTACCAGAGCTGAGTTCTCAAAAGTCACAAGGAAGTTTAGTTAAAGAATAAGGCTGAACAAAACTGGGACAGGGGCCAAACAGGATATCTGTGGTCGAGCAC

+

BBBBBBFFFFFFFFFFFFFFFFFFFFFFFFFFFFFFFFFFFFFFFFFFFFFFFFFFFFFFFFFFFFFFFFBFFFFFFFFFFFFFFFFFFFFFFFFFFFFFFFFFFFFFFFFFFFFFFFFFFFFFFF

@GWZHISEQ02:315:C9E6MANXX:5:2302:15923:84753

CGCCATTTTGCAAGGCATGAAAAAGTACCAGAGCTGAGTTCTCAAAAGTCACAAGGAAGTTTAGTTAAAGAATAAGGCTGAACAAAACTGGGACAGGGGCCAAACAGGATATCTGTGGTCGAGCAC

+

BBBBBFFFFFFFFFFFFFFFFFFFFFFFFFFFFFFFFFFFFFFFFFFFFFFFFFFFFFFFFFFFFFFFFFFFFFFFFFFFFFFFFFFFFFFFFFFFFFFFFFFFFFFFFFFFFFFFFFFFFFFFFF

@GWZHISEQ02:315:C9E6MANXX:5:2303:19438:4682

CGCCATTTTGCAAGGCATGAAAAAGTACCAGAGCTGAGTTCTCAAAAGTCACAAGGAAGTTTAGTTAAAGAATAAGGCTGAACAAAACTGGGACAGGGGCCAAACAGGATATCTGTGGTCGAGCAC

+

BBBBBFFFFFFFFFFFFFFFFFFFFFFFFFFFFFFFFFFFFFFFFFFFFFFFFFFFFFFFFFFFFFFFFFFFFFFFFFFFFFFFFFFFFFFFFFFFFFFFFFFFFFFFFFFFFFFFFFFFFFFFFF

@GWZHISEQ02:315:C9E6MANXX:5:2305:17268:62653

CGCCATTTTGCAAGGCATGAAAAAGTACCAGAGCTGAGTTCTCAAAAGTCACAAGGAAGTTTAGTTAAAGAATAAGGCTGAACAAAACTGGGACAGGGGCCAAACAGGATATCTGTGGTCGAGCAC

+

BBBBBFFFFFFFFFFFFFFFFFFFFFFFFFFFFFFFFFFFFFFFFFFFFFFFFFFFFFFFFFFFFFFFFFFFFFFFFFFFFFFFFFFFFFFFFFFFFFFFFFFFFFFFFFFFFFFFFFFFFFFFFF

@GWZHISEQ02:315:C9E6MANXX:5:2305:6679:96688

CGCCATTTTGCAAGGCATGAAAAAGTACCAGAGCTGAGTTCTCAAAAGTCACAAGGAAGTTTAGTTAAAGAATAAGGCTGAACAAAACTGGGACAGGGGCCAAACAGGATATCTGTGGTCGAGCAC

+

BBBBBFFFFFFFFFFFFFFFFFFFFFFFFFFFFFFFFFFFFFFFFFFFFFFFFFFFFFFFFFFFFBFFFFFFFFFFFFFFFFFFFFFFFFFFFFFFFFFFFFFFFFFFFFFFFFFFFFFFFFFFFF

@GWZHISEQ02:315:C9E6MANXX:5:2308:7407:16534

CGCCATTTTGCAAGGCATGAAAAAGTACCAGAGCTGAGTTCTCAAAAGTCACAAGGAAGTTTAGTTAAAGAATAAGGCTGAACAAAACTGGGACAGGGGCCAAACAGGATATCTGTGGTCGAGCAC

+

BBBBBFFFFFFFFFFFFFFFFFFFFFFFFFFFFFFFFFFFFFFFFFFFFFFFFFFFFFFFFFFFFFFFFFFFFFFFFFFFFFFFFFFFFFFFFFFFFFFFF<FFFFFFFFFFFFFFFFFFFFFFFF

@GWZHISEQ02:315:C9E6MANXX:5:2311:4767:69909

CGCCATTTTGCAAGGCATGAAAAAGTACCAGAGCTGAGTTCTCAAAAGTCACAAGGAAGTTTAGTTAAAGAATAAGGCTGAACAAAACTGGGACAGGGGCCAAACAGGATATCTGTGGAGATCGGA

+

BBBBB</<FFFFF<<FFFBFFBFFFFFFFFFFFFFFFFFFFBFFFFFFFFFFFFFFFFFFFBFFB/FFFFFFFFFBFFFFFFFFFFFFFFFFFFFFFFFFFFFBFFFFFFFFFFFFFFFFFFFFFF

@GWZHISEQ02:315:C9E6MANXX:5:2314:1972:62206

CGCCATTTTGCAAGGCATGAAAAAGTACCAGAGCTGAGTTCTCAAAAGTCACAAGGAAGTTTAGTTAAAGAATAAGGCTGAACAAAACTGGGACAGGGGCCAAACAGGATATCTGTGGTCGAGCAC

+

BBBBBFFFFFFFFFFFFFFFFFFFFFFFFFFFFFFFFFFFFFFFFFFFFFFFFFFFFFFFFFFFFFFFFFFFFFFFFFFFFFFFFFFFFFFFFFFFFFFFFFFFFFFFFFFFFFFFFF<BFFFFFF

@GWZHISEQ02:315:C9E6MANXX:5:2315:9664:23113

CGCCATTTTGCAAGGCATGAAAAAGTACCAGAGCTGAGTTCTCAAAAGTCACAAGGAAGTTTAGTTAAAGAATAAGGCTGAACAAAACTGGGACAGGGGCCAAACAGGATATCTGTGGTCGAGCAC

+

BBBBBFFFFFFFFFFFFFFFFFFFFFFFFFFFFFFFFFFFFFFFFFFFFFFFFFFFFFFFFFFFFFFFFFFFFFFFFFFFFFFFFFFFFFFFFFFFFFFFFFFFFFFFFFFFFFFFFFFFFFFFFF

@GWZHISEQ02:315:C9E6MANXX:5:2316:13171:82165

CGCCATTTTGCAAGGCATGAAAAAGTACCAGAGCTGAGTTCTCAAAAGTCACAAGGAAGTTTAGTTAAAGAATAAGGCTGAACAAAACTGGGACAGGGGCCAAACAGGATATCTGTGGTCGAGCAC

+

BBBBBFFFFFFFFFFFFFFFFFFFFFFFFFFFFFFFFFFFFFFFFFFFBFFFFFFFFFFFFFFFFFFFFFFFFFFFFFFFFFFFFFFFFFFFFFFBFFFFFFFFFFFFFFFFFFFFFFFFFFBFFF

@GWZHISEQ02:315:C9E6MANXX:5:1106:20186:58223

TCCCAGTTTTGTTCAGCCTTATTCTTTAACTAAACTTCCTTGTGACTTTTGAGAACTCAGCTCTGGTACTTTTTCATGCCTTGCAAAATGGCGAGATCGGAAGAGCACACGTCTGAACTCCAGTCA

+

BBBBBFFFFFFFFFFBFFFFFFFFFFFFFFFFFFFFBFFFFFFFFFFFFFFFFFFFFFFFFFFFF<FFFFFFFFFFFFFFFFFFFFFFFFFBFFFFFFFFBFFFFFFFFFFFFFFB/BFFBBFFF/

@GWZHISEQ02:315:C9E6MANXX:5:1308:11979:35371

GTGCTCGACCACAGATATCCTGTTTGGCCCCTGTCCCAGTTTTGTTCAGCCTTATTCTTTAACTAAACTTCCTTGTGACTTTTGAGAACTCAGCTCTGGTACTTTTTCATGCCTTGCAAAATGGCG

+

BBBBBFBFFFF<FFFFFFFFFFFFFFFFFFFFFFFFFFFFFFFFFFFFFFFFF<FFFFFFFFFFBFFFFFFFFFFFFFFFFFFFFBFFFFFFFFFFFFFFFFFFFFFBFFFF/FFBFBFFFFF<FB

@GWZHISEQ02:315:C9E6MANXX:5:1313:19540:93948

CTCGACCACAGATATCCTGTTTGGCCCCTGTCCCAGTTTTGTTCAGCCTTATTCTTTAACTAAACTTCCTTGTGACTTTTGAGAACTCAGCTCTGGTACTTTTTCATGCCTTGCAAAATGGCGAGA

+

BBBBBFFFFFFFFFFFFFFFFFFFFFFFFFFFFFFFFFFFFFFFFFFFFFFFFFFFFFFFFFFFFFFFFFFFFFFFFFFFFFFFFFFFFFFFFFFFFFFFFFFFFFFFFFFFFFFFFFFFFFFFFF

@GWZHISEQ02:315:C9E6MANXX:5:2107:3185:5395

CTCTGTCCCAGTTTTGTTCAGCCTTATTCTTTAACTAAACTTCCTTGTGACTTTTGAGAACTCAGCTCTGGTACTTTTTCATGCCTTGCAAAATGGCGAGATCGGAAGAGCACACGTCTGAACTCC

+

BBBBBFFFFFFFFFFFFFFFFFFFFFFFFFFFFFFFFFFFFFFFFFFFFFFFFFFFFFFFFFFFFFFFFFFFFFFFFFFFFFFFFFFFFFFFFFFFFFFBFFFFFFFFFFFFFFFFFFFFFFFFFF

@GWZHISEQ02:315:C9E6MANXX:5:2107:15784:41289

ATCCTGTTTGGCCCCTGTCCCAGTTTTGTTCAGCCTTATTCTTTAACTAAACTTCCTTGTGACTTTTGAGAACTCAGCTCTGGTACTTTTTCATGCCTTGCAAAATGGCGAGATCGGAAGAGCACA

+

BBBBBFFFFFFFFFFFFFFFFFFFFFFFFFFFFFFFFFFFFFFFFFFFFFFFFFFFFFFFFFFFFFFFFFFFFFFFFFFFFFFFFFFFFFFFFFFFFFFFFFFFFFFFFFFFFFFFFFFFFFFFFF

@GWZHISEQ02:315:C9E6MANXX:5:2210:5613:55536

CCTGTTTGGCCCCTGTCCCAGTTTTGTTCAGCCTTATTCTTTAACTAAACTTCCTTGTGACTTTTGAGAACTCAGCTCTGGTACTTTTTCATGCCTTGCAAAATGGCGAGATCGGAAGAGCACACG

+

BBBBBFFFFFFFFFFFFFFFFFFBFBFFFFFFFFFFFFFFFFFFFFFFFFFFFFBFFFBBFFFFBBBF<FFFFFFFFFFFFFFFFFFFFFFFFFFBFFFFFFFFFFFFFFFFFFFBFFFFFBFFFF

@GWZHISEQ02:315:C9E6MANXX:5:2211:2404:83883

GGCTCCTGTCCCAGTTTTGTTCAGCCTTATTCTTTAACTAAACTTCCTTGTGACTTTTGAGAACTCAGCTCTGGTACTTTTTCATGCCTTGCAAAATGGCGAGATCGGAAGAGCACACGTCTGAAC

+

/BBBBFBBFFFFFFFFFFFFFFFFFFFFFFFFFFFFFFFFFFFFFFFFFFFFFFFFFFFFFFFFFFFFFFFFFFFFFFFFFFFFFFBFFFFFFFFFFFFFFFFFFFFFFFFFFFFB<FFFFFFFF<

@GWZHISEQ02:315:C9E6MANXX:5:2311:4767:69909

CCACAGATATCCTGTTTGGCCCCTGTCCCAGTTTTGTTCAGCCTTATTCTTTAACTAAACTTCCTTGTGACTTTTGAGAACTCAGCTCTGGTACTTTTTCATGCCTTGCAAAATGGCGAGATCGGA

+

BBBBBFFFFBFFFF/FFFBFFFFFFFFFFFFFFBFBFFFFFFFFFFFFFFFBFFFFFFFFFFFFFFFFFFFFFBBFFFFFFFFFFFFFFFBFFFFFFFFBFFFFFFF<FBBFFFFBFB7<FFFFF<

@GWZHISEQ02:315:C9E6MANXX:5:1104:1261:8340

GCCATTTTGCAAGGCATGAAAAAGTACCAGAGCTGAGTTCTCAAAAGTCACAAGGAAGTTTAGTTAAAGAATAAGGCTGAACAAAACTGGGACAGGGGCCAAACAGGATATCTGTGGTCGAGCACC

+

<</<B/FF<<</FBF</FBB/</7</FFFF/<F/FFB//</B<<</<FFF<BFFF</F<<B<F/<BFFFBB</<FFF<<B<<FFB<FBF</B/<B<B<F/FFF/BBBFBFF//<7B//77/7<<7<

@GWZHISEQ02:315:C9E6MANXX:5:1208:14587:2057

GCCATTTTGCAAGGCATGAAAAAGTACCAGAGCTGAGTTCTCAAAAGTCACAAGGAAGTTTAGTTAAAGAATAAGGCTGAACAAAACTGGGACAGGGGCCAAACAGGATATCTGTGGTCGAGCACC

+

BBBBBFFFFFFFFFFFFFFFFFFFFFFFFFFFFFFFFFFFFFFFFFFFFFFFFFFFFFFFFFFFFFFFFFFFFFFFFFFFFFFFFFFFFFFFFFFFFFFFFFFFFFFFFFFFFFFFFFFFFFFFFF

@GWZHISEQ02:315:C9E6MANXX:5:2204:5635:19937

GCCATTTTGCAAGGCATGAAAAAGTACCAGAGCTGAGTTCTCAAAAGTCACAAGGAAGTTTAGTTAAAGAATAAGGCTGAACAAAACTGGGACAGGGGCCAAACAGGATATCTGTGGTCGAGCACC

+

BBBBBFFFFFFFFFFFFFFF<<FFFFFFFFFFBFFFFFFFFFFFFFFBBB/FFFFF/FFFFFFFFFFFFFFFFF</FFFFFFFFFFFFFFFFFFFF7BFBFFFFFFFFFFFBFFFFFFF<<FFFBF

@GWZHISEQ02:315:C9E6MANXX:5:2216:15268:80413

GCCATTTTGCAAGGCATGAAAAAGTACCAGAGCTGAGTTCTCAAAAGTCACAAGGAAGTTTAGTTAAAGAATAAGGCTGAACAAAACTGGGACAGGGGCCAAACAGGATATCTGTGGAGATCGGAA

+

BBBBBFFFFFFFFFFFFFFFFBFFFFFFFFFFFFFFFFFFFFFFFFFFFFFFFFFFFFFFFFFFFFFFFFFFFFFFFFFFFFFFFFFFFFFFFFFFFFFFFFFFFFFFFFFFFFFFFFFFFFFFFF

@GWZHISEQ02:315:C9E6MANXX:5:2216:15268:80413

CCACAGATATCCTGTTTGGCCCCTGTCCCAGTTTTGTTCAGCCTTATTCTTTAACTAAACTTCCTTGTGACTTTTGAGAACTCAGCTCTGGTACTTTTTCATGCCTTGCAAAATGGCAGATCGGAA

+

BBBBBFFFFFFFFFFFFFFFFFFFFFFFFFFFFFFFFFFFFFFFFFFFFFFFFFFFFFFFFFFFFFFFFFFFFFFFFFFFFFFFFFFFFFFFFFFFFFFFFFFFFFFFFFFFFFFFFFFFFFFFFF

@GWZHISEQ02:315:C9E6MANXX:5:1213:20424:69011

CCATTTTGCAAGGCATGAAAAAGTACCAGAGCTGAGTTCTCAAAAGTCACAAGGAAGTTTAGTTAAAGAGTAAGGCTGAACAAAACTGGGACAGGGGCCAAACAGGATATCTGTGGTCGAGCACCT

+

BBBBBFFFFFFFFFFFFFFFFFFFFFFFFFFFFFFFFFFFFFFFFFFFFFFFFFFFFFFFFFFFFFFFF/FFFFFFFFFFFFFFFFFFFFFFFFFFFBFFBFFFFFFFFFFFFFFFFFFFFFFFFF

@GWZHISEQ02:315:C9E6MANXX:5:2115:18890:11954

CCATTTTGCAAGGCATGAAAAAGTACCAGAGCTGAGTTCTCAAAAGTCACAAGGAAGTTTAGTTAAAGAATAAGGCTGAACAAAACTGGGACAGGGGCCAAACAGGATATCTGTGGTCGAGCACCT

+

BBBBBFFFFFFFFFFFFFFFFFFFFFFFFFFFFFFFFFFFFFFFFFFFFFFFFFFFFFFFFFFFFFFFFFFFFFFFFFFFFFFFFFFFFFFFFFFFFFFFFFFFFFFFFFFBFFFFFFFFFFFFFB

@GWZHISEQ02:315:C9E6MANXX:5:2209:6370:36263

CCATTTTGCAAGGCATGAAAAAGTACCAGAGCTGAGTTCTCAAAAGTCACAAGGAAGTTTAGTTAAAGAATAAGGCTGAACAAAACTGGGACAGGGGCCAAACAGGATATCTGTGGTCGAGCACCT

+

BBBBBFFFFFFFFFFFFFFFFFFFFFFFFFFFFFFFFFFFFFFFFFFFFFFFFFFFFFFFFFFFFFFFFFFFFFFFFFFFFFFFFFFFFFFFFFFFFFFFFFFFFFFFFFFFFFFFFFFFFFFFFF

@GWZHISEQ02:315:C9E6MANXX:5:2209:16304:89579

CCATTTTGCAAGGCATGAAAAAGTACCAGAGCTGAGTTCTCAAAAGTCACAAGGAAGTTTAGTTAAAGAATAAGGCTGAACAAAACTGGGACAGGGGCCAAACAGGATATCTGTGGTCGAGCACCT

+

BBBBBFFFFFFFFFFFFFFFFFFFFFFFFFFFFFFFFFFFFFFFFFFFFFFFFFFFFFFFFFFFFFFFFFFFFFFFFFFFFFFFFFFFFFFFFFFFFFFFFFFFFFFFFFFFFFFFFFFFFFFFFB

@GWZHISEQ02:315:C9E6MANXX:5:1104:4984:22436

CATTTTGCAAGGCATGAAAAAGTACCAGAGCTGAGTTCTCAAAAGTCACAAGGAAGTTTAGTTAAAGAATAAGGCTGAACAAAACTGGGACAGGGGCCAAACAGGATATCTGTGGTCGAGCACCTG

+

BBBBBFFFFFFFFFFFFFFFFFFFFFFFFFFFFFFFFFFFFFFFFFFFFFFFFFFFFFFFFFFFFFFFFFFFFFFFFFFFFFFFFFFFFFFFFBFFFFFFFFFFFFFFFFFFFFFFFFFFFFFFFF

@GWZHISEQ02:315:C9E6MANXX:5:1303:11662:96265

CATTTTGCAAGGCATGAAAAAGTACCAGAGCTGAGTTCTCAAAAGTCACAAGGAAGTTTAGTTAAAGAATAAGGCTGAACAAAACTGGGACAGGGGCCAAACAGGATATCTGTGGTCGGGCACCTG

+

BBBBBFFFFFFFFFFFFFFFFFFFFFFFFFFFFFFFFFFFFFFFFFFFFFFFFFFFFFFFFFFFFFFFFFFFFFFFFFFFFFFFFFFFFFFFFFFFFFFFFFFFFFFFFFFFFFFFFFFFFFFFFF

@GWZHISEQ02:315:C9E6MANXX:5:2209:2589:83100

CATTTTGCAAGGCATGAAAAAGTACCAGAGCTGAGTTCTCAAAAGTCACAAGGAAGTTTAGTTAAAGAATAAGGCTGAACAAAACTGGAGATCGGAAGAGCGTCGTGTAGGGAAAGAGTGTTAAGA

+

BBBBBFFFFFFFFFFFFFFFFFFFFFFFFFFFFFFFFFFFFFFFFFFFFFFFFFFFFFFFFFFFFFFFFFFFFFFFFFFFFFFFFFFFFFFFFFFFFFFFFFFFFFFFFFFFBFFFFFBF<BFFFB

@GWZHISEQ02:315:C9E6MANXX:5:2210:4682:85809

CATTTTGCAAGGCATGAAAAAGTACCAGAGCTGAGTTCTCAAAAGTCACAAGGAAGTTTAGTTAAAGAATAAGGCTGAACAAAACTGGGACAGGGGCCAAACAGGATATCTGTGGTCGAGCACCTG

+

BBBBBFFFFFFFFFFFFFFFFFFFFFFFFFFFFFFFFFFFFFFFFFFFFFFFFFFFFFFFFFFFFFFFFFFFFFFFFFFFFFFFFFFFFFFFFFFFFFFBFFFFFFF/FFFFFFFFFFFFFFFFFF

@GWZHISEQ02:315:C9E6MANXX:5:2309:2687:56718

CATTTTGCAAGGCATGAAAAAGTACCAGAGCTGAGTTCTCAAAAGTCACAAGGAAGTTTAGTTAAAGAATAAGGCTGAACAAAACTGGGACAGGGGCCAAACAGGATATCTGTGGTCGAGCACCTG

+

BBBBBFFFFFFFFFFFFFFFFFFFFFFFFFFFFFFFFFFFFFFFFFFFFFFFFFFFFFFFFBFFFFFFFFFFFFFFFFFFFFFFFFFFFFFFFFFFFFFFFFFFFFFFFFFFFFFFFFFFFFFFFF

@GWZHISEQ02:315:C9E6MANXX:5:2312:10870:46087

CATTTTGCAAGGCATGAAAAAGTACCAGAGCTGAGTTCTCAAAAGTCACAAGGAAGTTTAGTTAAAGAATAAGGCTGAACAAAACTGGGACAGGGGCCAAACAGGATATCTGTGGTCGAGCACCTG

+

BBBBBFFFFFFFFFFFFFFFFFFFFFFFFFFFFFFFFFFFFFFFFFFFFFFFFFFFFFFFFFFFFFFFFFFFFFFFFFFFFFFFFFFFFFFFFFFFFFFFFFFFFFFFFFFFFFFFFFFFFBFFFF

@GWZHISEQ02:315:C9E6MANXX:5:2104:8860:90631

GGCCTCTGTCCCAGTTTTGTTCAGCCTTATTCTTTAACTAAACTTCCTTGTGACTTTTGAGAACTCAGCTCTGGTACTTTTTCATGCCTTGCAAAATGAGATCGGAAGAGCACACGTCTGAACTCC

+

BBBBBFFFFFFFFFFFFFFFFFFFFFFFFFFFFFFFFFFFFFFFFFFFFFFFFFFFFFFFFFFFFFFFFFFFFFFFFFFFFFFFFFFFFFFFFFFFFFFFBFFFFFFFFFFFFFFFFFFFFFFFFF

@GWZHISEQ02:315:C9E6MANXX:5:2209:2589:83100

CCAGTTTTGTTCAGCCTTATTCTTTAACTAAACTTCCTTGTGACTTTTGAGAACTCAGCTCTGGTACTTTTTCATGCCTTGCAAAATGAGATCGGAAGAGCACACGTCTGAACTCCAGTCACATTA

+

BBBB<<<BFFFFFFFFFFFFFFFFFFFFFFFFFFFFFFFBFB/BFFFBBFFFFFFFFFFFFFFFFFFFFFFFFFFFFBFFFFFFFFFFFFFFBFFFFFFFFFFFFFFFFFFFFFFFFFFFFFFFFF

@GWZHISEQ02:315:C9E6MANXX:5:1101:12595:11530

ATTTTGCAAGGCATGAAAAAGTACCAGAGCTGAGTTCTCAAAAGTCACAAGGAAGTTTAGTTAAAGAATAAGGCTGAACAAAACTGGGACAGGGGCCAAACAGGATATCTGTGGTCGAGCACCTGG

+

BBBBBFFFFFFFFFFFFFFFFFFFFFFFFFFFFFBFFFFFFFFFFFFFFFFFFFFFFFFFFFFFFFFFFFFFFFFFFFFFFFFFFFFFFFFFFFFFFFFFFFFFFFFFFFFFFFFFFFFFFFFFFF

@GWZHISEQ02:315:C9E6MANXX:5:1205:7253:25953

ATTTTGCAAGGCATGAAAAAGTACCAGAGCTGAGTTCTCAAAAGTCACAAGGAAGTTTAGTTAAAGAATAAGGCTGAACAAAACTGGGACAGGGGCCAAACAGGATATCTGTGGTCGAGCACCTGG

+

BBBBBFFFFFFFFFFFFFFFFFFFFFFFFFFFFFFFFFFFFFFFFFFFFFFFFFFFFFFFFFFFFFFFFFFFFFFFFFFFFFFFFFFFFFFFFFFFFFFFFFFFFFFFFFFFFFFFFFFFFFFFFF

@GWZHISEQ02:315:C9E6MANXX:5:1312:10315:18970

ATTTTGCAAGGCATGAAAAAGTACCAGAGCTGAGTTCTCAAAAGTCACAAGGAAGTTTAGTTAAAGAATAAGGCTGAACAAAACTGGGACAGGGGCCAAACAGGATATCTGTGGTCGAGCACCTGG

+

BBBBBFFFFFFFFFFFFFFFFFFFFFFFFFFFFFFFFFFFFFFFFFFFFFFFFFFFFFFFFFFFFFFFFFFFFFFFFFFFFFFFFFFFFFFFFFFFFFFFFFFFFFFFFFFFFFFFFFFFFFFFFF

@GWZHISEQ02:315:C9E6MANXX:5:1313:5194:32690

ATTTTGCAAGGCATGAAAAAGTACCAGAGCTGAGTTCTCAAAAGTCACAAGGAAGTTTAGTTAAAGAATAAGGCTGAACAAAACTGGGACAGGGGCCAAACAGGATATCTGTGAGATCGGAAGAGC

+

BBBBBFFFFFFFFFFFFFFFFFFFFFFFFFFFFFFBFFFFFFFFFFFFFFFFFFFFFFFFFFFFFFFFFFFFFFFFFFFFFFFFFFFFFFFFFFFFFFFFFFFFFFFFFFFFFFFFFFFFFFFFFB

@GWZHISEQ02:315:C9E6MANXX:5:2204:10681:83994

ATTTTGCAAGGCATGAAAAAGTACCAGAGCTGAGTTCTCAAAAGTCACAAGGAAGTTTAGTTAAAGAATAAGGCTGAACAAAACTGGGACAGGGGCCAAACAGGATATCTGTGGTCGAGCACCTGG

+

BBBBBFFFFFFFFFFFFFFFFFFFFFFFFFFFFFFFFFFFFFFFFFFFFFFFFFFFFFFFFFFFFFFFFFFFFFFFFFFFFFFFFFFFFFFFFFFFFFFFFBFFFFFFFFFFFFFFFFFFFFFFFF

@GWZHISEQ02:315:C9E6MANXX:5:2205:6467:61374

ATTTTGCAAGGCATGAAAAAGTACCAGAGCTGAGTTCTCAAAAGTCACAAGGAAGTTTAGTTAAAGAATAAGGCTGAACAAAACTGGGACAGGGGCCAAACAGGATATCTGTGGTCGAGCACCTGG

+

BBBBBFFFFFFFFFFFFFFFFFFFFFFFFFFFFFFFFFFFFFFFFFFFFFFFFFFFFFFFFFFFFFFFFFFFFFFFFFFFFFFFFFFFFFFFFFFFFFFFFFFFFFFFFFFFFFFFFFFFFFFFFF

@GWZHISEQ02:315:C9E6MANXX:5:2301:10988:61544

ATTTTGCAAGGCATGAAAAAGTACCAGAGCTGAGTTCTCAAAAGTCACAAGGAAGTTTAGTTAAAGAATAAGGCTGAACAAAACTGGGACAGGGGCCAAACAGGATATCTGTGGTCGAGCACCTGG

+

BBBBBFFFFFFFFFFFFFFFFFFFFFFFFFFFFFFFFFFFFFFFFFFFFFFFFFFFFFFFFFFFFFFFFFFFFFFFFFFFFFFFFFFFFFFFFFFFFFFFFFFFFFFFFFFFFFFFFFFFFFFFFF

@GWZHISEQ02:315:C9E6MANXX:5:2309:16271:17877

ATTTTGCAAGGCATGAAAAAGTACCAGAGCTGAGTTCTCAAAAGTCACAAGGAAGTTTAGTTAAAGAATAAGGCTGAACAAAACTGGGACAGGAGATCGGAAGAGCGTCGTGTAGGGAAAGAGTGT

+

BBBBBFFFFFFFFFFFFFFFFFFFFFFFFFFFFFFFFFFFFFFFFFFFFFFFFFFFFFFFFFFFFFFFFFFFFFFFFFFFFFFFFFFFFFFFFFFFFFFFFFFFFFFFFFFFFFFFFFFFFFFBFF

@GWZHISEQ02:315:C9E6MANXX:5:2311:2269:70059

ATTTTGCAAGGCATGAAAAAGTACCAGAGCTGAGTTCTCAAAAGTCACAAGGAAGTTTAGTTAAAGAATAAGGCTGAACAAAACTGGGACAGGGGCCAAACAGGATATCTGTGGTCGAGCACCTGG

+

BB<BBFFFFFFFFFFFFBFFFFFBFFFFFFFFFFFFFFFFFFFFFFFFFFFFFBFFFFFFFFFFFFFFFFFFFFFFFFFFFFFFFFFFFFFFFFFFFFFFFFFFFFFFFFFFFFFFFFFFFFFFFF

@GWZHISEQ02:315:C9E6MANXX:5:2312:11893:57086

ATTTTGCAAGGCATGAAAAAGTACCAGAGCTGAGTTCTCAAAAGTCACAAGGAAGTTTAGTTAAAGAATAAGGCTGAACAAAACTGGGACAGGGGCCAAACAGGATATCTGTGGTCGAGCACCTGG

+

BBBBBFF<FB</BFFFF<<</FFFFFFF/BFB//FFF/BFFBFFFBF<FFFFF/BF/FFFFFFFFFFFFFFFF<BFFFBBFFFFFFFFFFFFBFBFFFFFFFFFFF/F/FBFFFBBBBFFFFFFFF

@GWZHISEQ02:315:C9E6MANXX:5:1313:5194:32690

CACAGATATCCTGTTTGGCCCCTGTCCCAGTTTTGTTCAGCCTTATTCTTTAACTAAACTTCCTTGTGACTTTTGAGAACTCAGCTCTGGTACTTTTTCATGCCTTGCAAAATAGATCGGAAGAGC

+

BBBBBFFFFFFFFFFFFFFFFFFFFFFFFFBFFFFFFFFFFFFFFFFFFFFFFFFFFFFFFFFFFFFFFFFFFFFFF/FFBFFFFFFFFFFFFFFFFFFFFFFFFFFFFFFFFFFFFFFFFFFFF/

@GWZHISEQ02:315:C9E6MANXX:5:2309:16271:17877

CCTGTCCCAGTTTTGTTCAGCCTTATTCTTTAACTAAACTTCCTTGTGACTTTTGAGAACTCAGCTCTGGTACTTTTTCATGCCTTGCAAAATAGATCGGAAGAGCACACGTCTGAACTCCAGTCA

+

BBBBBFFFFFFFFFFFFFFFFFFFFFFFFFFFFFFFFFFFFFFFFFFFFFFFFFFBFFFFFFFFFFFFFFFFFFFFFFFFFFFFFFFFFFFFFFFFFFFFFFFFFFFFFFFFFFFFFFFFFFFFFF

@GWZHISEQ02:315:C9E6MANXX:5:1308:14049:50066

TTGCAAGGCATGAAAAAGTACCAGAGCTGAGTTCTCAAAAGTCACAAGGAAGTTTAGTTAAAGAATAAGGCTGAACAAAACTGGGACAGGGGCCAAACAGGATATCTGTGGTCGAGCACCTGGGCC

+

BBBBB<FF/FFFBFFFFFFFFFF/FFFFB<FFFFFFFFFBFF<FFFFF/BFBFFBBBFFFFFFFFFFFF<BFFFFFFFFFFFFF/FBFF//B<BFFFFFFBFFF<FBBFFFFFFFFFFFFFFFFBB

@GWZHISEQ02:315:C9E6MANXX:5:1304:5022:23101

GGCCCGGGTGCTCGACCACAGATATCCTGTTTGGCCCCTGTCCCAGTTTTGTTCAGCCTTATTCTTTAACTAAACTTCCTTGTGACTTTTGAGAACTCAGCTCTGGTACTTTTTCATGCCTTGCAA

+

BBBBBFFFFFFFFFFFFFFFFFFFFFFFFFFFFFFFFFFFFFFFFFFFFFFFFFFFFFFFFFFFFFFFFFFFFFFFFFFFFFFFFFFFFFFFFFFFFFFFFFFFFBFFFFFFFFFFF/<FFFFFFF

@GWZHISEQ02:315:C9E6MANXX:5:1303:2483:57711

TGCAAGGCATGAAAAAGTACCAGAGCTGAGTTCTCAAAAGTCACAAGGAAGTTTAGTTAAAGAATAAGGCTGAACAAAACTGGGACAGGGGCCAAACAGGATATCTGTGGTCGAGCACCTGGGCCC

+

BBBBBFFFFFFFFFFFFFFFFFFFFFFFFFFFFFFFFFFFFFFFFFFFFFFFFFFFFFFFFFFFFFFFFFFFFFFFFFFFFFFFFFFFFFFFFFFFFFFFFFFFFFFFFFFFFFFFFFFBFFFFFF

@GWZHISEQ02:315:C9E6MANXX:5:2205:8679:57794

TGCAAGGCATGAAAAAGTACCAGAGCTGAGTTCTCAAAAGTCACAAGGAAGTTTAGTTAAAGAATAAGGCTGAACAAAACTGGGACAGGGGCCAAACAGGATATCTGTGGTCGAGCACCTGGGCCC

+

BBBBBFFFFFFFFFFFFFFFFFFFFFFFFFFFFFFFFFFFFFFFFFFFFFFFFFFFFFFFFFFFFFFFFFFFFFFFFFFFFFFFFFFFFFFFFFFFFFFFFFBFFFFFFFFFFFFFFFFFFFFFFF

@GWZHISEQ02:315:C9E6MANXX:5:1201:19471:71040

CAAGGCATGAAAAAGTACCAGAGCTGAGTTCTCAAAAGTCACAAGGAAGTTTAGTTAAAGAATAAGGCTGAACAAAACTGGGACAGGGGCCAAACAGGATATCTGTGGTCGAGCACCTGGGCCCCG

+

BBBBBFFFFFFFFFFFFFFFFFFFFFFFFFFFFFFFFFFFFFFFFFFFFFFFFFFFFFFFFFFFFFFFFFFFFFFFFFFFFFFFFFFBFFFFFFFFFFFFFFFFFFFFFFFFFFFFFFFFFFFFFF

@GWZHISEQ02:315:C9E6MANXX:5:2103:11962:3817

CAAGGCATGAAAAAGTACCAGAGCTGAGTTCTCAAAAGTCACAAGGAAGTTTAGTTAAAGAATAAGGCTGAACAAAACTGGGACAGGGGCCAAACAGGATATCTGTGGTCGAGCACCTGGGCCCCG

+

BBBBBFFFFFFFFFFFFFFFFFFFFFBFFFFFFFFBFFFFFFFFFFFFFFFFFFFFFFFFFFFFFFFFFF/FFFFFFFFFFFFFFFFFFFFFFFFFFFFFFFFFFFFFFFFFFFFFFFFFFF<BBF

@GWZHISEQ02:315:C9E6MANXX:5:2111:7294:17953

CAAGGCATGAAAAAGTACCAGAGCTGAGTTCTCAAAAGTCACAAGGAAGTTTAGTTAAAGAATAAGGCTGAACAAAACTGGGACAGGGGCCAAACAGGATATCTGTGGTCGAGCACCTGGGCCCCG

+

BB</B<B<<FBFFBB<BFFFFBFFFFFFFFBFFFFFF<FFFFFFFFB/F<<BFBFF<FFBBFFFFFFFFFFFFFFFFFFFFFFFFFFFFFFFFBBFFFFFFFFFFFFFFFFFFFFFFFFFFFFFFF

@GWZHISEQ02:315:C9E6MANXX:5:1302:1728:25517

CCGGGGCCCAGGTGCTCGACCACAGATATCCTGTTTGGCCCCTGTCCCAGTTTTGTTCAGCCTTATTCTTTAACTAAACTTCCTTGTGACTTTTGAGAACTCAGCTCTGGTACTTTTTCATGCCTT

+

BBBBBFFFFFFFFFFFFFFFFFFFFFFFFFFFFFFFFFFFFFFFFFFFFFFFFFFFFFFFFFFFFFFFFFFFFFFFFFFFFFFFFFFFFFFFFFFFFFFFFFFFFFFFFFFFFFFFFFBFFFFFFF

@GWZHISEQ02:315:C9E6MANXX:5:1207:7336:67819

GGCATGAAAAAGTACCAGAGCTGAGTTCTCAAAAGTCACAAGGAAGTTTAGTTAAAGAATAAGGCTGAACAAAACTGGGACAGGGAGATCGGAAGAGCGTCGTGTAGGGAAAGAGTGTTAAGATTA

+

BBBBBFFFFFFFFFFFFFFFFFFFFFFFFFFFFFFFFFFFFFFFFFFFFFFFFFFFFFFFFFFFFFFFFFFFFFFFFFFFFFFFFFFFFFFFFFFFFFFBFFBFFFFFBFFFFFFBFFFFFFFF<B

@GWZHISEQ02:315:C9E6MANXX:5:2202:9433:78647

GGCATGAAAAAGTACCAGAGCTGAGTTCTCAAAAGTCACAAGGAAGTTTAGTTAAAGAATAAGGCTGAACAAAACTGGGACAGGGGCCAAACAGGATATCTGTGGTCGAGCACCTGGGCCCCGGCT

+

BBBBBFFFFFFFFFFFFFFFFFFFFFFFFFFFFFFFFFFFFFFFFFFFFFFFFFFFFFFFFFFFFFFFFFFFFFFFFFFFFFFFFFFFFFFFFFFFFFFFFFFFFFFFFFFFFFFFFFFFFFFFF/

@GWZHISEQ02:315:C9E6MANXX:5:1207:7336:67819

CCCTGTCCCAGTTTTGTTCAGCCTTATTCTTTAACTAAACTTCCTTGTGACTTTTGAGAACTCAGCTCTGGTACTTTTTCATGCCAGATCGGAAGAGCACACGTCTGAACTCCAGTCACATTACTC

+

<BBBBFFFFFFFFFFFFFFFFFFFFFFFFFFFFFFFFFFFFFFFFFFFFFFFFFFFFFFFFFFFFFFFFFFFFFFFFFFFFFFFFFFFFFFFFFFFFFFFFFFFFFFFFFFFFFFFFFFFFFFFFF

@GWZHISEQ02:315:C9E6MANXX:5:1107:7317:4509

GCATGAAAAAGTACCAGAGCTGAGTTCTCAAAAGTCACAAGGAAGTTTAGTTAAAGAATAAGGCTGAACAAAACTGGGACAGGGGCCAAACAGGATATCTGTGGTCGAGCACCTGGGCCCCGGCTC

+

BBBBBFFFFFFFFFFFFFFFFFFFFFFFFFFFFFFFFFFFFFFFFFFFFFFFFFFFFFFFFFFFFFFFFFFFFFFFFFFFFFFFFFFFFFFFFFFFFFFFFFFFFFFFFFFFFFFFFFFFFFFFFB

@GWZHISEQ02:315:C9E6MANXX:5:1114:14477:27444

GCATGAAAAAGTACCAGAGCTGAGTTCTCAAAAGTCACAAGGAAGTTTAGTTAAAGAATAAGGCTGAACAAAACTGGGACAGGGGCCAAACAGGATATCTGTGGTCGAGCACCTGGGCCCCGGCTC

+

BBBBBFFFFFFFFFFFFFFFFFFFFFFFFFFFFFFFFFFFFFFFFFFFFFFFFFFFFFFFFFFFFFFFFFFFFFFFFFFFFFFFFFFFFFFFFFFFFFFFFFFFFFFFFFFFFFFFFFFFFFFFFF

@GWZHISEQ02:315:C9E6MANXX:5:1310:15648:47460

GCATGAAAAAGTACCAGAGCTGAGTTCTCAAAAGTCACAAGGAAGTTTAGTTAAAGAATAAGGCTGAACAAAACTGGGACAGGGGCCAAACAGGATATCTGTGGTCGAGCACCTGGGCCCCGGCTC

+

BBBBBFFFFFFFFFFFFFFFFFFFFFFFFFFFFFFFFFFFFFFFFFFFFFFFFFFFFFFFFFFFFFFBFFFFFFFFFFFFFFFFFFFFFFFFFFFFFFFFFFFFFFFFFFFFFFFFFFFFFFFFFF

@GWZHISEQ02:315:C9E6MANXX:5:2201:17293:24588

GCATGAAAAAGTACCAGAGCTGAGTTCTCAAAAGTCACAAGGAAGTTTAGTTAAAGAATAAGGCTGAACAAAACTGGGAGATCGGAAGAGCGTCGTGTAGGGAAAGAGTGTTAAGATTAGTGTAGA

+

BBBBBFFFFFFBFFFFFFFFFFFFFFFFFFFFFFFFFFFFFFFFFFFFFFFFFFFFFFFFFFFFFFFFFFFFFFFFFFFFFFFFFFFFFFFFFFFFFFFFFFFFFFFFFFFFFFFFBFFBFFF<B/

@GWZHISEQ02:315:C9E6MANXX:5:2206:16411:4135

GCATGAAAAAGTACCAGAGCTGAGTTCTCAAAAGTCACAAGGAAGTTTAGTTAAAGAATAAGGCTGAACAAAACTGGGACAGGGGCCAAACAGGATATCTGTGGTCGAGCACCTGGGCCCCGGCTC

+

BBBBBFFFFFFFFFFFFFFFFFFFFFFFFFFFFFFFFFFFFFFFFFFFFFFFFFFFFFFFFFFFFFFFFFFFFFFFFFFFFFFFFFFFFFFFFFFFFFFFFFFFFFFFFFFFFFFFFFFFFFFFBF

@GWZHISEQ02:315:C9E6MANXX:5:2308:1258:70613

GCATGAAAAAGTACCAGAGCTGAGTTCTCAAAAGTCACAAGGAAGTTTAGTTAAAGAATAAGGCTGAACAAAACTGGGACAGGGGCCAAACAGGATATCTGTGGTCGAGCACCTGGGCCCCGGCTC

+

BBBBBFFFFFFFFFFFFFFFFFFFFFFFFFFFFFFFFFFFFFFFFFFFFFFFFFFFFFFFFFFFFFFFFFFFFFFFFFFFBFFFFBFFFFFFFFFFFFFFFFFFFFFFFFFFFFFFFFFFFFFFFF

@GWZHISEQ02:315:C9E6MANXX:5:2201:17293:24588

CCCAGTTTTGTTCAGCCTTATTCTTTAACTAAACTTCCTTGTGACTTTTGAGAACTCAGCTCTGGTACTTTTTCATGCAGATCGGAAGAGCACACGTCTGAACTCCAGTCACATTACTCGATCTCG

+

BBBBBFFFFFFFFFFFFFFFFFFFFFFFFFFFFFFFFFFFFFFFFFFFFFFFFFFFFFFFFFFFFFFFFFFFFFFFBFFFFFFFFFFFFFFFFFFFFFFFBFFFFFFFFFFFFFFFFFFFF</FFB

@GWZHISEQ02:315:C9E6MANXX:5:2113:13123:71634

ATGAAAAAGTACCAGAGCTGAGTTCTCAAAAGTCACAAGGAAGTTTAGTTAAAGAATAAGGCTGAACAAAACTGGGACAGGGGCCAAACAGGATATCTGTGGTCGAGCACCTGGGCCCCGGCTCAG

+

BBBBBFFFFFFFBFFFFFFFFFFFFFFFFFFFFFFFFFFFFFFFFFFFFFFFFFFFFFFFFFFFFFFFFFFFFFFFFFFFFFFFFFFFFFFFFFFFFFFFFFFFFFFFFFFFFFFFFFFFFFFFFF

@GWZHISEQ02:315:C9E6MANXX:5:2205:6493:43528

ATGAAAAAGTACCAGAGCTGAGTTCTCAAAAGTCACAAGGAAGTTTAGTTAAAGAATAAGGCTGAACAAAACTGGGACAGGGGCCAAACAGGATATCTGTGGTCGAGCACCTGGGCCCCGGCTCAG

+

BBBBBFFBB<BBFBFFFBBFFFFBFFFFFFFFFFFB<BF///<FFFBBFFFFFFFFFFFFFFFFFFFB<B<FFF<BFFFF<FFFFFFFFFFFFFFFFFFFFFFFFFFFBFFBFFFFFFFF<FFB7B

@GWZHISEQ02:315:C9E6MANXX:5:1111:1742:62348

TGAAAAAGTACCAGAGCTGAGTTCTCAAAAGTCACAAGGAAGTTTAGTTAAAGAATAAGGCTGAACAAAACTGGGACAGGGGCCAAACAGGATATCTGTGGTCGAGCACCTGAGAGATCGGAAGAG

+

BBBBBFFFFFFFFFFFFFFFFFFFFFFFFFFFFFFFFFFFFFFFFFFFFFFFFFFFFFFFFFFFFFFFFFFFFFFFFFFFFFFFFFFFFFFFFFFFFFFFFFFFFFFFFFFFFFFFFFFFFFFFFF

@GWZHISEQ02:315:C9E6MANXX:5:1115:4225:73866

TGAAAAAGTACCAGAGCTGAGTTCTCAAAAGTCACAAGGAAGTTTAGTTAAAGAATAAGGCTGAACAAAACTGGGACAGGGGAGATCGGAAGAGCGTCGTGTAGGGAAAGAGTGTTAAGATTAGTG

+

BBBBBFFFFFFFFFFFFFFFFFFFFFFFFFFFFFFFFFFFFFFFFFFFFFFFFFFFFFFFFFFFFFFFFFFFFFFFFFFFFFFFFFFFFFFFFFFFFFFBFFFFFFFFFFFFFFFFFFFFFFFFF<

@GWZHISEQ02:315:C9E6MANXX:5:1205:12991:67100

TGAAAAAGTACCAGAGCTGAGTTCTCAAAAGTCACAAGGAAGTTTAGTTAAAGAATAAGGCTGAACAAAACTGGGACAGGGGCCAAACAGGATATCTGTGGTCGAGCACCTGGGCCCCGGCTCAGG

+

BBBBBFFFFFFFFFFFFFFFFFFFFFFFFFFFFFFFFFFFFFFFFFFFFFFFFFFFFFFFFFFFFFFFFFFFFFFFFFFFFFFFFFFFFFFFFFFFFFFFFFFFFFFFFFFFFFFFFFFFFBBFFF

@GWZHISEQ02:315:C9E6MANXX:5:1207:11213:18432

TGAAAAAGTACCAGAGCTGAGTTCTCAAAAGTCACAAGGAAGTTTAGTTAAAGAATAAGGCTGAACAAAACTGGGACAGGGGCCAAACAGGATATCTGTGGTCGAGCACCTGGGCCCCGGCTCAGG

+

BBBBBFFFFFFFFFFFFFFFFFFFFFFFFFFFFFFFFFFFFFFFFFFFFFFFFFFFFFFFFFFFFFFFFFFFFFFFFFFFFBFFFFFFFFFFFFFFFFFFFFFFFFFFFFFFFFFFFFFFFFFFFF

@GWZHISEQ02:315:C9E6MANXX:5:1209:14849:79029

TGAAAAAGTACCAGAGCTGAGTTCTCAAAAGTCACAAGGAAGTTTAGTTAAAGAATAAGGCTGAACAAAACTGGGACAGGGGCCAAACAGGATATCTGTGGTCGAGCACCTGGGCCCCGGCTCAGG

+

BBBBBFFFFFFFFFFFFFFFFFFFFFFFFFFFFFFFFFFFFFFFFFFFFFFFFFFFFFFFFFFFFFFFFFFFFFFFFFFFFFFFFFFFFFFFFFFFFFFFFFFFFFFFFFFFFFFFFFFFFFFFFF

@GWZHISEQ02:315:C9E6MANXX:5:1211:13096:21160

TGAAAAAGTACCAGAGCTGAGTTCTCAAAAGTCACAAGGAAGTTTAGTTAAAGAATAAGGCTGAACAAAACTGGGACAGGGGCCAAACAGGATATCTGTGGTCGAGCACCTGGGCCCCGGCTCAGG

+

BBBBBFFFFFFFFFFFFFFFFFFFFFFFFFFFFFFFFFFFFFFFFFFFFFFFFFFFFFFFFFFFFFFFFFFFFFFFFFFFFFFFFFFFFFFFFFFFFFFFFFFFBFFFFFFFFFFFFFFFF/7FFF

@GWZHISEQ02:315:C9E6MANXX:5:1214:13552:78170

TGAAAAAGTACCAGAGCTGAGTTCTCAAAAGTCACAAGGAAGTTTAGTTAAAGAATAAGGCTGAACAAAACTGGGACAGGGGCCAAACAGGATATCTGTGGTCGAGCACCTGGGCCCCGGCTCAGG

+

BBBBBFFFFFFFFFFFFFFFFFFFFFFFFFFFFFFFFFFFFFFFFFFFFFFFFFFFFFFFFFFFFFFFFFFFFFFFFFFFFFFFFFFFFFFFFFFFFBFFFFFFFFFFFFFFFFFFFFFFFFFFFF

@GWZHISEQ02:315:C9E6MANXX:5:1309:9686:47321

TGAAAAAGTACCAGAGCTGAGTTCTCAAAAGTCACAAGGAAGTTTAGTTAAAGAATAAGGCTGAACAAAACTGGGACAGGGGCCAAACAGGATATCTGTGGTCGAGCACCTGGGCCCCGGCTCAGG

+

BBBBBFFFFFFFFFFFFFFFFFFFFFFFFFFFFFFFFFFFFFFFFFFFFFFFFFFFFFFFFFFFFFFFFFFFFFFFFFFFFFFFFFFFFFFFFFFFFFFFFFFFFFFFFFFFFFFFBFFFFFFFFF

@GWZHISEQ02:315:C9E6MANXX:5:2104:10924:22164

TGAAAAAGTACCAGAGCTGAGTTCTCAAAAGTCACAAGGAAGTTTAGTTAAAGAATAAGGCTGAACAAAACTGGGACAGGGGCCAAACAGGATATCTGTGGTCGAGCACCTGGGCCCCGGCTCAGG

+

BBBBBFFFFFFFFFFFFFFFFFFFFFFFFFFFFFFFFFFFFFFFFFFFFFFFFFFFFFFFFFFFFFFFFFFFFFFFFFFFFFFFFFFFFFFFFFFFFFFFFFFFFFFFFFFFFFFFFFFFFFFFFF

@GWZHISEQ02:315:C9E6MANXX:5:2106:18164:42605

TGAAAAAGTACCAGAGCTGAGTTCTCAAAAGTCACAAGGAAGTTTAGTTAAAGAATAAGGCTGAACAAAACTGGGACAGGGGCCAAACAGGATATCTGTGGTCGAGCACCTGGGCCCCGGCTCAGG

+

BBBBBFFFFFFFFFFFFFFFFFFFFFFFFFFFFFFFFFFFFFFFFFFFFFFFFFFFFFFFFFFFFFFFFFFFFFFFFFFFFFFFFFFFFFFFFFFFFFFFFFFFFFFFFFFFFFFFFFFFFFFFFF

@GWZHISEQ02:315:C9E6MANXX:5:2108:5689:73557

TGAAAAAGTACCAGAGCTGAGTTCTCAAAAGTCACAAGGAAGTTTAGTTAAAGAATAAGGCTGAACAAAACTGGGACAGGGGCCAAACAGGATATCTGTGGTCGAGCACCTGGGCCCCGGCTCAGG

+

BBBBBFFFFFFFFFFFFFFFFFFFFFFFFFFFFFFFFFFFFFFFFFFFFFFFFFFFFFFFFFFFFFFFFFFFFFFFFFFFFFFFFFFFFFFFFFFFFFFFFFFFFFFFFFFFFFFFFFFFFFFFFF

@GWZHISEQ02:315:C9E6MANXX:5:2201:3749:60623

TGAAAAAGTACCAGAGCTGAGTTCTCAAAAGTCACAAGGAAGTTTAGTTAAAGAATAAGGCTGAACAAAACTGGGACAGGGGCCAAACAGGATATCTGTGGTCGAGCACCTGGGCCCCGGCTCAGG

+

BBBBBFFFFFFFFFFFFFFFFFFFFFFFFFFFFFFFFFFFFFFFFFFFFFFFFFFFFFFFFFFFFFFFFFFFFFFFFFFFFFFFFFFFFFFFFFFFFFFFFFFFFFFFFFFFFFBFFFFFFFFFFF

@GWZHISEQ02:315:C9E6MANXX:5:2208:11802:14509

TGAAAAAGTACCAGAGCTGAGTTCTCAAAAGTCACAAGGAAGTTTAGTTAAAGAATAAGGCTGAACAAAACTGGGACAGGGGCCAAACAGGATATCTGTGGTCGAGCACCTGGGCCCCGGCTCAGG

+

BBBBBFFFFFFFFFFFFFFFFFFFFFFFFFFFFFFFFFFFFFFFFFFFFFFFFFFFFFFFFFFFFFFFFFFFFFFFFFFFFFFFFFFFFFFFFFFFFFFFFFFFFFFFFFFFFFFFFFFFFFFFFF

@GWZHISEQ02:315:C9E6MANXX:5:2215:1907:67565

TGAAAAAGTACCAGAGCTGAGTTCTCAAAAGTCACAAGGAAGTTTAGTTAAAGAATAAGGCTGAACAAAACTGGGACAGGGGCCAAACAGGATATCTGTGGTCGAGCACCTGGGCCCCGGCTCAGG

+

BB/BBBBFFFFBBFFF<<<FF<</</<B/FFFFFFFF/</<FBFFBF/B<FFFFFFFBF//FFF<FFFFFFFFFFFFFFFFFFBF/B//BFFFFBB<FFFF//<B/FFF/B/FFFBFFFFFFBFFF

@GWZHISEQ02:315:C9E6MANXX:5:2301:4829:30368

TGAAAAAGTACCAGAGCTGAGTTCTCAAAAGTCACAAGGAAGTTTAGTTAAAGAATAAGGCTGAACAAAACTGGGACAGGGGCCAAACAGGATATCTGTGGTCGAGCACCTGGGCCCCGGCTCAGG

+

BBBBBFFFFFFFFFFFFFFFFFFFFFFFFFFFFFFFFFF<FFFFFFFFFFFFFFFFFFFFFFFFFFFFFFFFFFFFFFFFBBBFFFFFFFFFFFFFFFFFFBFFFFFFFFFFFFFFFFF<FFFFFF

@GWZHISEQ02:315:C9E6MANXX:5:2308:19607:8227

TGAAAAAGTACCAGAGCTGAGTTCTCAAAAGTCACAAGGAAGTTTAGTTAAAGAATAAGGCTGAACAAAACTGGGACAGGGGCCAAACAGGATATCTGTGGTCGAGCACCTGGGCCCCGGCTCAGG

+

BBBBBFFBFFFFFFFFFFFFFFFFBFFFFFFFFFFFFFFFFFFFFFFFFFFFFFFFFFFFFFFFBFB<FFFFFFFFFFFFFFFFFFFFFFFFFFFFFFFFFBFFFBFFFFFFFFFFFFBBFFFFFF

@GWZHISEQ02:315:C9E6MANXX:5:1111:1742:62348

CTCAGGTGCTCGACCACAGATATCCTGTTTGGCCCCTGTCCCAGTTTTGTTCAGCCTTATTCTTTAACTAAACTTCCTTGTGACTTTTGAGAACTCAGCTCTGGTACTTTTTCAAGATCGGAAGAG

+

BBBBBFFFFFFFFFFFFFFFFFFFFFFFFFFFFFFFFFFFFFFFFFFFFFFFFFFFFFFFFFFFFFFFFFFFFFFFFFFFFFFFFFFFFFFFFFFFFFFFFFFFFFFFFFFFFFFFFFFFFFBFFF

@GWZHISEQ02:315:C9E6MANXX:5:1115:4225:73866

CCCCTGTCCCAGTTTTGTTCAGCCTTATTCTTTAACTAAACTTCCTTGTGACTTTTGAGAACTCAGCTCTGGTACTTTTTCAAGATCGGAAGAGCACACGTCTGAACTCCAGTCACATTACTCGAT

+

BBBBBFFFFFFFFFFFFFFFFFFFFFFFFFFFFFFFFFFFFFFFFFFFFFFFFFFFFFFFFFFFFFFFFFFFFFFFFFFFFFFFFFFFFFFFFFFFFFFFFFFFFFFFFFFFFFFFFFFFFFFFF/

@GWZHISEQ02:315:C9E6MANXX:5:1304:14364:88317

CCTGAGCCGGGGCCCAGGTGCTCGACCACAGATATCCTGTTTGGCCCCTGTCCCAGTTTTGTTCAGCCTTATTCTTTAACTAAACTTCCTTGTGACTTTTGAGAACTCAGCTCTGGTACTTTTTCA

+

BBBBBFFFFFFFFFFFFFFFFFFFFFFFFFFFFFFFFFFFFFFFF/FFFFFFFFFFFFFFFFFFFFFFFBFFFFFFFFFFFFFFFFFFFFFFFFBFFFFFFFFFFFFFFFFFFFFFFFFFFFFFFB

@GWZHISEQ02:315:C9E6MANXX:5:1104:19371:4959

GAAAAAGTACCAGAGCTGAGTTCTCAAAAGTCACAAGGAAGTTTAGTTAAAGAATAAGGCTGAACAAAACTGGGACAGGGGCCAAACAGGATATCTGTGGTCGAGCACCTGGGCCCCGGCTCAGGG

+

BBBBBFFFFFFFFFFFFFFFFFFFFFFFFFFFFFFFFFFFFFFFFFFFFFFFFFFFFFFFFFFFFFFFFFFFFFFFFFFFFFFFFFFFFFFFFFFFFFFFFFFFFFFFFFFFFFFFFFFFFFFFFF

@GWZHISEQ02:315:C9E6MANXX:5:1105:8077:46710

GAAAAAGTACCAGAGCTGAGTTCTCAAAAGTCACAAGGAAGTTTAGTTAAAGAATAAGGCTGAACAAAACTGGGACAGGGGCCAAACAGGATATCTGTGGTCGAGCACCTGGGCCCCGGCTCAGGG

+

BBBBBFFFFFFFFFFFFFFFFFFFFFFFFFFFFFFFFFFFFFFFFFFFFFFFFFFFFFFFFFFFFFFFFFFFFFFFFFFFFFFFFFFFFFFFFFFFFFFFFFFFFFFFFFFFFFFFFFFFFFFFFF

@GWZHISEQ02:315:C9E6MANXX:5:1106:13593:86780

GAAAAAGTACCAGAGCTGAGTTCTCAAAAGTCACAAGGAAGTTTAGTTAAAGCATAAGGCTGAACAAAACTGGGACAGGGGCCAAACAGGATATCTGTGGTCGAGCACCTGGGCCCCGGCTCAGGG

+

/<BBBFBBFBFBFF/FFFFFFB//FFFFB/FB/FFBFBFBF<BFBBFFFF<B/<<BFFBFFFFFFBF<FFFFFFFFFF//BF<FFBFFFFFFFFF/<<BFFFB/FFFFFFFFFBFB<F/B7BFF<F

@GWZHISEQ02:315:C9E6MANXX:5:1110:2183:16515

GAAAAAGTACCAGAGCTGAGTTCTCAAAAGTCACAAGGAAGTTTAGTTAAAGAATAAGGCTGAACAAAACTGGGACAGGGGCCAAACAGGATATCTGTGGTCGAGCACCTGGGCCCCGGCTCAGGG

+

BBBBBFFFFFFFFFFFFFFFFFFFFFFFFFFFFFFFFFFFFFFFFFFFFFFFFFFFFFFFFFFFFFFFFFFFFFFFFFFFFBFFFFFFFFFFFFFFFFFFFFBFFFFFFFFFFFFFFFFFBFFFFF

@GWZHISEQ02:315:C9E6MANXX:5:1212:19636:9542

GAAAAAGTACCAGAGCTGAGTTCTCAAAAGTCACAAGGAAGTTTAGTTAAAGAATAAGGCTGAACAAAACTGGGACAGGGGCCAAACAGGATATCTGTGGTCGAGCACCTGGGCCCCGGCTCAGGG

+

BBBBBFFFFFFFFFFFFFFFBFFFFFFFFFFFFFFFFFFFFFFFFFFFFFFFFFFFFFFFFFBFFFFFFFFFFFFFFFFFFFFFFFFFFFFFFFF<FFFFFFFFFFFFFFFFFFFBFFFFFFFFFF

@GWZHISEQ02:315:C9E6MANXX:5:1303:1981:18742

GAAAAAGTACCAGAGCTGAGTTCTCAAAAGTCACAAGGAAGTTTAGTTAAAGAATAAGGCTGAACAAAACTGGGACAGGGGCCAAACAGGATATCTGTGGTCGAGCACCTGGGCCCCGGCTTAGGG

+

BBBBBFFFFFFFFFFFFFBFFFFFFFFFFFFFFFFFFFFFFFFFFFFFFFFFFFFFFFFFFFFFFFFFFFFFFFFFFFFFFFFFFFFFFFFFFFFFFFFFFFFFFFFFFFFFFF/BFFFFFFFFFF

@GWZHISEQ02:315:C9E6MANXX:5:1312:7371:92587

GAAAAAGTACCAGAGCTGAGTTCTCAAAAGTCACAAGGAAGTTTAGTTAAAGAATAAGGCTGAACAAAACTGGGACAGGGGCCAAACAGGATATCTGTGGTCGAGCACCTGGGCCCCGGCTCAGGG

+

BBBBBFFFFFFFFFFFFFFFFFFFFFFFFFFFFFFFFFFFFFFFFFFFFFFFFFFFFFFFFFFFFFFFFFFFFFFBFFFFFFFFFFFFFFFFFFFFFFFFFFFFFFFFFFFFFFFFFFBFFBFFFF

@GWZHISEQ02:315:C9E6MANXX:5:1313:11173:56795

GAAAAAGTACCAGAGCTGAGTTCTCAAAAGTCACAAGGAAGTTTAGTTAAAGAATAAGGCTGAACAAAACTGGGACAGGGGCCAAACAGGATATCTGTGGTCGAGCACCTGGGCCCCGGCTCAGGG

+

BBBBBFFFFFFFFFFFFFFFFFFFFFFFFFFFFFFFFFFFFFFFFFFFFFFFFFFFFFFFFFFFFFFFFFFFFFFFFFFF<FFFFFFFFFFFFFFFFFFFFFFFFFFFFFBBFFBBFFFFFFFFFF

@GWZHISEQ02:315:C9E6MANXX:5:2111:1730:85505

GAAAAAGTACCAGAGCTGAGTTCTCAAAAGTCACAAGGAAGTTTAGTTAAAGAATAAGGCTGAACAAAACTGGGACAGGGGCCAAACAGGATATCTGTGGTCGAGCACCTGGGCCCCGGCTCAGGG

+

BBB<BF<FFFFFFFFFFFFFFFFFFFFFFFFFFFFFFFFFFFFFFFFFFFFFFFFFFFFFFFFFFFFFFFFFFFFFFFF/FFFFFFFFFFFFFFFFFFFFFFFFFFFFFFFFFFFFFFFFFFF7BF

@GWZHISEQ02:315:C9E6MANXX:5:2116:8920:74107

GAAAAAGTACCAGAGCTGAGTTCTCAAAAGTCACAAGGAAGTTTAGTTAAAGAATAAGGCTGAACAAAACTGGGACAGGGGCCAAACAGGATATCTGTGGTCGAGCACCTGGGCCCCGGCTCAGGG

+

BBBBBFBFFFFFFFFFFFFFFFFFFFFFFFFFFFFFFFFFFFFFBFFFFFFFFFFFFFFFFFFFFFFFFFFFFFFFFFFFFFFFFFFFFFFFFFFFFFFFFFFFFFFFFFFFFFFFFFFFFFFFFF

@GWZHISEQ02:315:C9E6MANXX:5:2201:9375:71367

GAAAAAGTACCAGAGCTGAGTTCTCAAAAGTCACAAGGAAGTTTAGTTAAAGAATAAGGCTGAACAAAACTGGGACAGGGGCCAAACAGGATATCTGTGGTCGAGCACCTGGGCCCCGGCTCAGGG

+

BBBBBFB<FBBFF/<BFFFFFFFFFFBFFFFFFFFFFBFFFFFFFFFFBFFFFFFFFF<FFF/FFF<BFFFFFFFFF</FFFFF/FFFFFBFBFFFFFF<FFFFFBFFFBBBBFFFFFFFBFFBB<

@GWZHISEQ02:315:C9E6MANXX:5:2311:15540:82160

GAAAAAGTACCAGAGCTGAGTTCTCAAAAGTCACAAGGAAGTTTAGTTAAAGAATAAGGCTGAACAAAACTGGGACAGGGGCCAAACAGGATATCTGTGGTCGAGCACCTGGGCCCCGGCTCAGGG

+

/BBBBF/FBBBBFFFFFFBFFFFFFFFFFFFFFBFFFFFBBBFFFFFFFFFFFFFFFFF/BFB<FFFFFFFFFBFFFFB<FFFFFFFFFFFFFFFFFFFBFF<BBFFFFFFFBFF7BFFF/B<BF<

@GWZHISEQ02:315:C9E6MANXX:5:1105:19756:83507

CCCTGAGCCGGGGCCCAGGTGCTCGACCACAGATATCCTGTTTGGCCCCTGTCCCAGTTTTGTTCAGCCTTATTCTTTAACTAAACTTCCTTGTGACTTTTGAGAACTCAGCTCTGGTACTTTTTC

+

BBBBBFFFFFFFFFFFFFFFFFFFFFFFFFFFFFFFFFFFFFFFFFFFFFFFFFFFFFFFFFFFFFFFFFFFFFFFFFFFFFFFFFFFFFFFFFFFFFFFFFFFFFFFFFFFFFFFFFFFFFFFFF

@GWZHISEQ02:315:C9E6MANXX:5:2316:20431:42256

AAAAAGTACCAGAGCTGAGTTCTCAAAAGTCACAAGGAAGTTTAGTTAAAGAATAAGGCTGAACAAAACTGGGACAGGGGCCAAACAGGATATCTGTGGTCGAGCACCTGGGCCCCGGCTCAGGGC

+

BBBBBFFFFFFFFFFFFFFFFFFFFFFFFFFFFFFFFFFFFFFFFFFFFFFFFFFFFFFFFFFFFFFFFFFFFFFFFFFFFFBFFFFFFFFFBBFFFFFFFFFFFBFFBFFFF/BFFFF</FFFFF

@GWZHISEQ02:315:C9E6MANXX:5:2316:6146:55622

AAAAAGTACCAGAGCTGAGTTCTCAAAAGTCACAAGGAAGTTTAGTTAAAGAATAAGGCTGAACAAAACTGGGACAGGGGCCAAACAGGATATCTGTGGTCGAGCACCTGGGCCCCGGCTCAGGGC

+

BBBBBFFFFFFFFFFFFFFFFFFFFFFFFFFFFFFFFFFFBFFFFFFFFFFFFFFFFFFFFFFFFFFFFFFFFFFFFFFFFFFFFFFFFFFFFFFFFFFFFFFFFFFFFFFFFFFFFFF<FFFFFF

@GWZHISEQ02:315:C9E6MANXX:5:1113:12099:6041

AAAAGTACCAGAGCTGAGTTCTCAAAAGTCACAAGGAAGTTTAGTTAAAGAATAAGGCTGAACAAAACTGGGACAGGGGCCAAACAGGATATCTGTGGTCGAGCACCTGGGCCCCGGCTCAGGGCC

+

BBBBBFFFFFFFFFFFFFFFFFFFFFFFFFFFFFFFFFFFFFFFFFFFFFFFFFFFFFFFFFFFFFFFFFFFFFFFFFFFFFFFFFFFFFFFFFFFFFFFFFFFFFFFFFFFFFFFFFFFFFFFFB

@GWZHISEQ02:315:C9E6MANXX:5:1316:19215:101040

AAAAGTACCAGAGCTGAGTTCTCAAAAGTCACAAGGAAGTTTAGTTAAAGAATAAGGCTGAACAAAACTGGGACAGGGGCCAAACAGGATATCTGTGGTCGAGCACCTGGGCCCCGGCTCAGGGCC

+

BBBBBFFFFFFFFFFFFFFFFFFFFFFFFFFFFFFFFFFFFFFFFFFFFFFFFFFFFFFFFFFFFFFFFFFFFFFFFFFFFFFFFFFFFFFFFFFFFFFFFFFFFFFFFFFFFFFFFFFFFFFFFF

@GWZHISEQ02:315:C9E6MANXX:5:2204:14303:20276

AAAAGTACCAGAGCTGAGTTCTCAAAAGTCACAAGGAAGTTTAGTTAAAGAATAAGGCTGAACAAAACTGGGACAGGGGCCAAACAGGATATCTGTGGTCGAGCACCTGGGCCCCGGCTCAGGGCC

+

//</<F/<//<//BB/B/F<//B/</FFF///<//<FF/</F<<<<F/F/B<F/FB//<<<BF///FB/FFFFF<F<77B/FFBF/F/F<FFBB/<<<F/F<FB/B/<F///777/777/<//7/<

@GWZHISEQ02:315:C9E6MANXX:5:1104:19868:16401

AAAGTACCAGAGCTGAGTTCTCAAAAGTCACAAGGAAGTTTAGTTAAAGAATAAGGCTGAACAAAACTGGGACAGGGGCCAAACAGGATATCTGTGGTCGAGCACCTGGGCCCCGGCTCAGGGCCA

+

BBBBBFFFFFFFFFFFFFFFFFFFFFFFFFFFFFFFFFFFFFFFFFFFFFFFFFFFFFFFFFFFFFFFFBFFFFFFFFFFFFFFFFFFFFFFFFFFFFFFBFFFFFFFFFFBBBFFFBFFFFFFFF

@GWZHISEQ02:315:C9E6MANXX:5:1107:8638:99343

AAAGTACCAGAGCTGAGTTCTCAAAAGTCACAAGGAAGTTTAGTTAAAGAATAAGGCTGAACAAAACTGGGACAGGGGCCAAACAGGATATCTGTGGTCGAGCACCTGGGCCCCGGCTCAGGGCCA

+

BBBBBFFFFFFFFFFFFFFFFFFFFFFFFFFFFFFFFFFFFFFFFFFFFFFFFFFFFFFFFFFFFBFFFFFFFFFFFFFFFFFFFFFFFFFFFFFFFFFFFFFFFFFFFFFFBB<FFFFFFFFFFF

@GWZHISEQ02:315:C9E6MANXX:5:1115:20046:3940

AAAGTACCAGAGCTGAGTTCTCAAAAGTCACAAGGAAGTTTAGTTAAAGAATAAGGCTGAACAAAACTGGGACAGGGGCCAAACAGGATATCTGTGGTCGAGCACCTGGGCCCCGGCTCAGGGCCA

+

BBBBBFFFFFFFFFFFFFFFFFFFFFFFFFFFFFFFFFFFFFFFFFFFFFFFFFFFFFFFFFFFFFFFFFFFFFFFFFFFFFFFFFFFFFFFFFFFFFFFFFFFFFFFFFFFFFFFFFFFFFFFFF

@GWZHISEQ02:315:C9E6MANXX:5:2308:19861:39434

AAAGTACCAGAGCTGAGTTCTCAAAAGTCACAAGGAAGTTTAGTTAAAGAATAAGGCTGAACAAAACTGGGACAGGGGCCAAACAGGATATCTGTGGTCGAGCACCTGGGCCCCGGCTCAGGGCCA

+

BBBBBFFFFFFFFFFFFFFBFFFFFFFFFFFFFFFFFFFFFFFFFFFFFFFFFFFFFFFFFFFFFFFFFFFFFFF<FFFF<F/FFFFFFFFFFFFFFFFFFFFFFFFFFFFFFFFFFFFFFFF<FF

@GWZHISEQ02:315:C9E6MANXX:5:2312:8945:67883

AAAGTACCAGAGCTGAGTTCTCAAAAGTCACAAGGAAGTTTAGTTAAAGAATAAGGCTGAACAAAACTGGGACAGGGGCCAAACAGGATATCTGTGGTCGAGCACCTGGGCCCCGGCTCAGGGCCA

+

BBBBBFFFFFFFFFFFFFFFFFFFFFFFFFFFFFFBFFFFFFFFFFFFFFFFFFFFFFFFFFFFFFFFFFFFFFFFFFFFFFFFFFFFFFFFFFFFFFFFFFFFFFFFFFFFFFFFFFFFFFFFFF

@GWZHISEQ02:315:C9E6MANXX:5:1109:10397:23013

AAGTACCAGAGCTGAGTTCTCAAAAGTCACAAGGAAGTTTAGTTAAAGAATAAGGCTGAACAAAACTGGGACAGGGGCCAAACAGGATATCTGTGGTCGAGCACCTGGGCCCCGGCTCAGGGCCAA

+

BBBBBFFFFFFFFFFFFFFFFFFFFFFFFFFFFFFFFFFFFFFFFFFFFFFFFFFFFFFFFFFFFFFFFFFFFFFFFFFFFFFFFFFFFFFFFFFFFFFFFFFFFFFFFFFFFFFFFFFFFFFFFF

@GWZHISEQ02:315:C9E6MANXX:5:1111:12310:67731

AAGTACCAGAGCTGAGTTCTCAAAAGTCACAAGGAAGTTTAGTTAAAGAATAAGGCTGAACAAAACTGGGACAGGGGCCAAACAGGATATCTGTGGTCGAGCACCTGGGCCCCGGCTCAGGGCCAA

+

BBBBBFFFFFFFFFFFFFFFFFFFFFFFFFFFF</FFFFFFFFFBFFFFFFFBFBFFFFFFFFFFFFFFBFFFFFBFFFFFFFFFFFFFFFFFFFFFFFFFFFFFFFFFFFFFFFFFFFFFFFFFF

@GWZHISEQ02:315:C9E6MANXX:5:1306:12624:29117

AAGTACCAGAGCTGAGTTCTCAAAAGTCACAAGGAAGTTTAGTTAAAGAATAAGGCTGAACAAAACTGGGACAGGGGCCAAACAGGATATCTGTGGTCGAGCACCTGGGCCCCGGCTCAGGGCCAA

+

BBBBBFFFFFFFFFFFFFFFFFFFFFFFFFFFFFFFFFFFFFFFFFFFFFFFFFFFFFFFFFFFFFFFFFFFFFFFFFFFFFFFFFFFFFFFFFFFFFFFFFFFFFFFFFFFFFFFFFFFFFFFFF

@GWZHISEQ02:315:C9E6MANXX:5:2303:10013:99483

CTTGGCCCTGAGCCGGGGCCCAGGTGCTCGACCACAGATATCCTGTTTGGCCCCTGTCCCAGTTTTGTTCAGCCTTATTCTTTAACTAAACTTCCTTGTGACTTTTGAGAACTCAGCTCTGGTACT

+

BBBBBFFFFFFFFFFFFFFFFFFFFFFFFFFFFFFFFFFFFFFFFFFFFFFFFFFFFFFFFFFFFFFFFFFFFFFFFFFFFFFFFFFFFFFFFFFFFFFFFFFFFFFFFFFFFFFFFFFFFFFFFF

@GWZHISEQ02:315:C9E6MANXX:5:1201:8909:31088

GTACCAGAGCTGAGTTCTCAAAAGTCACAAGGAAGTTTAGTTAAAGAATAAGGCTGAACAAAACTGGGACAGGGGCCAAACGGGATATCTGTGGTCGAGCACCTGGGCCCCGGCTCAGGGCCAAGA

+

BBBBBFFFFFFFFFFFFFFFFFFFFFFFFFFFFFFFFFFFFFFFFFFFFFFFFFFFFFFFFFFFFFFFFFFFFFFFFFFFFFFFFFFFFFFFFFFFFFFFFFFFFFFFFFFFFFFFFFFFFFFFBF

@GWZHISEQ02:315:C9E6MANXX:5:2213:10975:21543

GTACCAGAGCTGAGTTCTCAAAAGTCACAAGGAAGTTTAGTTAAAGAATAAGGCTGAACAAAACTGGGACAGGGGCCAAACAGGATATCTGTGGTCGAGCACCTGGGCCCCGGCTCAGGGCCAAGA

+

BBBBBFFFFFFFFFFFFFFFFFFFFFFFFFFFFFFFFFFFFFFFFFFFFFFFFFFFFFFFFFFFFFFFFFFFFFFFFFFFFFFFFFFFFFFFFFFFFFFFFFFFFFFFFFFFFFFFFFFFFFFFFF

@GWZHISEQ02:315:C9E6MANXX:5:1313:15834:41932

TTCTTGGCCCTGAGCCGGGGCCCAGGTGCTCGACCACAGATATCCTGTTTGGCCCCTGTCCCAGTTTTGTTCAGCCTTATTCTTTAACTAAACTTCCTTGTGACTTTTGAGAACTCAGCTCTGGTA

+

BBBBBFFFFFFFFFFFFFFFFFFFFFFFFFFFFFFFFFFFFFFFFFFFFFFFFFFFFFFFFFFFFFFFFFFFFFFFFFFFFFFFFFFFFFFFFFFFFFFFFFFFFFFFFFFFFFFFFFFFFFFFFF

@GWZHISEQ02:315:C9E6MANXX:5:1307:15783:26875

ACCAGAGCTGAGTTCTCAAAAGTCACAAGGAAGTTTAGTTAAAGAATAAGGCTGAACAAAACTGGGACAGGGGCCAAACAGGATATCTGTGGTCGAGCACCTGGGCCCCGGCTCAGGGCCAAGAAC

+

BBBBBFFFFFFFFFFFFBFBBFBFFFFFFFFFFFFFFFFFFFFFFFFFFFFFFFFFFFFFFFFFFFFFFFFFFFFFFFFFFFFFFFFFFFFFFFFFFFFFFFFFFFFFFFFFFFFFFFFFFFFBFF

@GWZHISEQ02:315:C9E6MANXX:5:1108:1795:92340

CCAGAGCTGAGTTCTCAAAAGTCACAAGGAAGTTTAGTTAAAGAATAAGGCTGAACAAAACTGGGACAGGGGCCAAACAGGATATCTGTGGTCGAGCACCTGGGCCCCGGCTCAGGGCCAAGAACA

+

BBBBBFFFFFFFFFFFFFFFFFFFFFFFFFFFFFFFFFFFFFFFFFFFFFFFFFFFFFFFFFFFFFFFFFFFFFFFFFFFFFFFFFFFFFFFFFFFFFFFFFFFFFFFFFFFFFFFFFFFFFFFFF

@GWZHISEQ02:315:C9E6MANXX:5:1211:2114:96208

CCAGAGCTGAGTTCTCAAAAGTCACAAGGAAGTTTAGTTAAAGAATAAGGCTGAACAAAACTGGGACAGGGGCCAAACAGGATATCTGTGGTCGAGCACCTGGGCCCCGGCTCAGGGCCAAGAACA

+

BBBBBFFFFFFFFFFFFFFFFFFFFFFFFFFFFFFFFFFFFFFFFFFFFFFFFBFFFFFFFFFFFFFFFFFFFFFFFFFFFFFFFFFFFFFFFFFFFFFFFFFFFFFFFFFFFFFFFBFFFFBFFF

@GWZHISEQ02:315:C9E6MANXX:5:1312:11311:25703

CCAGAGCTGAGTTCTCAAAAGTCACAAGGAAGTTTAGTTAAAGAATAAGGCTGAACAAAACTGGGACAGGGGCCAAACAGGATATCTGTGGTCGAGCACCTGGGCCCCGGCTCAGGGCCAAGAACA

+

BBBBBFFFFFFFFFFFFFFFFFFFFFFFFFFFFFFFFFFFFFFFFFFFFFFFFFFFFFFFFFFFFFFFFFFFFFFFFFFFFFFFFFFFFFFFFFFFFFFFFFFFFFFFFFFFFBFFFFFFFFFFFF

@GWZHISEQ02:315:C9E6MANXX:5:2102:13083:79901

CCAGAGCTGAGTTCTCAAAAGTCACAAGGAAGTTTAGTTAAAGAATAAGGCTGAACAAAACTGGGACAGGGGCCAAACAGGATATCTGTGGTCGAGCACCTGGGCCCCGGCTCAGGGCCAAGAACA

+

BBBBBFFFFFFFFFFFFFFFFFFFFFFFFFFFFFFFFFFFFFFFFFFFFFFFFFFFFFFFFFFFFFFFFFFFBFFFFFFFFFFFFFFFFFFFFFFFFFFFFFFFFFFFFFFFFFFFFFFFFFFFFB

@GWZHISEQ02:315:C9E6MANXX:5:2107:4831:98719

CCAGAGCTGAGTTCTCAAAAGTCACAAGGAAGTTTAGTTAAAGAATAAGGCTGAACAAAACTGGGACAGGGGCCAAACAGGATATCTGTGGTCGAGCACCTGGGCCCCGGCTCAGGGCCAAGAACA

+

BBBBBFFFFFFFFFFFFFFFFFFFFFFFFFFFFFFFFFFFFFFFFFFFFFFFFFFFFFFFFFFFFFFFFFFFFFFFFFFFFFFFFFFFFFFFFFFFFFFFFFFFFFFFFFFFFFFFFFFFFFFFFF

@GWZHISEQ02:315:C9E6MANXX:5:2214:16874:26530

CCAGAGCTGAGTTCTCAAAAGTCACAAGGAAGTTTAGTTAAAGAATAAGGCTGAACAAAACTGGGACAGGGGCCAAACAGGATATCTGTGGTCGAGCACCTGGGCCCCGGCTCAGGGCCAAGAACA

+

BBBBBFFFFFFFFFFFFFFFFFFFFFFFFFFFFFFFFFFFFFFFFFFFFFFFFFFFFFFFFFFFFFFFFFFFFFFFFFFFFFFFFFFFFFFFFFFFFFFFFFFFFFFFFFFFFFFFFFFFFFFFFF

@GWZHISEQ02:315:C9E6MANXX:5:1210:2642:68458

TGTTCTTGTCCCTGAGCCGGGGCCCAGGTGCTCGACCACAGATATCCTGTTTGGCCCCTGTCCCAGTTTTGTTCAGCCTTATTCTTTAACTAAACTTCCTTGTGACTTTTGAGAACTCAGCTCTGG

+

BBBBBFFFFFFFFFFFFFFFFFFFFFFFFFFFFFFFFFFFFFFFFFFFFFFFBBFFFFFFFFFFFFFFFFFFFFFFFFFFFFFFFFFFFFFFFFFFFFFFFFFFFFFFFFFBF//FFFFFFFFFFF

@GWZHISEQ02:315:C9E6MANXX:5:1102:13270:93125

CAGAGCTGAGTTCTCAAAAGTCACAAGGAAGTTTAGTTAAAGAATAAGGCTGAACAAAACTGGGACAGGGGCCAAACAGGATATCTGTGGTCGAGCACCTGGGCCCCGGCTCAGGGCCAAGAACAG

+

BBBBBFFFFFFFFFFFFFFFFFFFFFFFFFFFFFFFFFFFFFFFFFFFFFFFBFFFFFFFFFFFFFFFFFBFFFFFFFFFFFFFFFFFFFFFFFFFFFFFFFFFFFFFFFFFFFFFFFFFFFFFFB

@GWZHISEQ02:315:C9E6MANXX:5:1106:3269:40329

CAGAGCTGAGTTCTCAAAAGTCACAAGGAAGTTTAGTTAAAGAATAAGGCTGAACAAAACTGGGACAGGGGCCAAACAGGATATCTGTGGTCGAGCACCTGGGCCCCGGCTCAGGGCCAAGAACAG

+

BBBBBFFFFFFFFFFFFFFFFFFFFFFFFFFFFFFFFFFFFFFFFFFFFFFFFFFFBFFFFFFFFFFFFFFFFFFFFFFFFFF/<FFFFFFFFBFFFFFFFFFFFFFFFFFFFFFFFFFFFBBFFF

@GWZHISEQ02:315:C9E6MANXX:5:1213:17560:11820

CAGAGCTGAGTTCTCAAAAGTCACAAGGAAGTTTAGTTAAAGAATAAGGCTGAACAAAACTGGGACAGGGGCCAAACAGGATATCTGTGGTCGAGCACCTGGGCCCCGGCTCAGGGCCAAGAACAG

+

BBBBBFFFFFFFFFFFFFFFFFFFFFFFFFFFFFFFFFFFFFFFFFFFFFFFFFFFFFFFFFFFFFFFFFFFFFFFFFFFFFFFFFFFFFFFFFFFFFFFFFFFFFFFFFBFFFFFFFFFFFFFFF

@GWZHISEQ02:315:C9E6MANXX:5:2203:10931:5061

CAGAGCTGAGTTCTCAAAAGTCACAAGGAAGTTTAGTTAAAGAATAAGGCTGAACAAAACTGGGACAGGGGCCAAACAGGATATCTGTGGTCGAGCACCTGGGCCCCGGCTCAGGGCCAAGAACAG

+

///<BFFF<FFFFFFFFBFFFBFFFFFB</F<BFFFFFFFFFFBFFFFFFFFFFFFBFFFFFFFFFFFFFFFFFFFFFFFFFFFFFBFFFFFFBFFFFFFFFBBBFF<F/B<FFFFFFFFFFFFFF

@GWZHISEQ02:315:C9E6MANXX:5:2207:17984:34083

CAGAGCTGAGTTCTCAAAAGTCACAAGGAAGTTTAGTTAAAGAATAAGGCTGAACAAAACTGGGACAGGGGCCAAACAGGATATCTGTGGTCGAGCACCTGGGCCCCGGCTCAGGGCCAAGAACAG

+

BBBBBFFFBFFFFFFFFFFFFFFFFFFFFFFFFFFFFFFFFFFFFFFFFFFFFFFFFFFFFFFFFFFFFFFFFFFFFFFFFFFFFFFFFFFFFFFFFBFFFFFFFFFFFFFFFFFFFFFFFFFFF<

@GWZHISEQ02:315:C9E6MANXX:5:2303:19195:82842

CAGAGCTGAGTTCTCAAAAGTCACAAGGAAGTTTAGTTAAAGAATAAGGCTGAACAAAACTGGGACAGGGGCCAAACAGGATATCTGTGGTCGAGCACCTGGGCCCCGGCTCAGGGCCAAGAACAG

+

BBBBBFFFFFFFFFFFFFFFFFFFFFFFFFFFBFFFFFFFFFFFFFFFFFFFFFFFFFFFFFFFFFFFFFFFFFFFFFFFBFFFFBBFFFFFFFFFFFFFFBFFFFFFFFFFFFFFFFFBFFFFFF

@GWZHISEQ02:315:C9E6MANXX:5:2308:5092:54063

CAGAGCTGAGTTCTCAAAAGTCACAAGGAAGTTTAGTTAAAGAATAAGGCTGAACAAAACTGGGACAGGGGCCAAACAGGATATCTGTGGTCGAGCACCTGGGCCCCGGCTCAGGGCCAAGAACAG

+

BBBBBFFFFFFFFFFFFFFFFFFFFFFFFFFFFFFFFFFFFFFFFFFFFFFFFFFFFFFFFFFFFFFFFFFFFFFFFFFFFFFFFFFFFFFFFFFFFFFFFFFFFFFFFFFFFFFFFFFFF7FFFF

@GWZHISEQ02:315:C9E6MANXX:5:1115:20046:3940

CTGTTCTTGGCCCTGAGCCGGGGCCCAGGTGCTCGACCACAGATATCCTGTTTGGCCCCTGTCCCAGTTTTGTTCAGCCTTATTCTTTAACTAAACTTCCTTGTGACTTTTGAGAACTCAGCTCTG

+

BBBBBFFFFFFFFFFFFFFFFFFFFFFFFFFFFFFFFFFFFFFFFFFFFFFFFFFFFFFFFFFFFFFFFFFFFFFFFFFFFFFFFFFFFFFFFFFFFFFFFFFFFFFFFFFFBFFFFFFFFFFFFF

@GWZHISEQ02:315:C9E6MANXX:5:1208:14587:2057

CTGTTCTTGGCCCTGAGCCGGGGCCCAGGTGCTCGACCACAGATATCCTGTTTGGCCCCTGTCCCAGTTTTGTTCAGCCTTATTCTTTAACTAAACTTCCTTGTGACTTTTGAGAACTCAGCTCTG

+

BBBBBFFFFFFFFFFFFFFFFFFFFFFFFFFFFFFFFFFFFFBFFFFFFFFFFFFFFFFFFFFFFFFFFFFFFFFFFFFFFFFFFFFFFFFFFFFFFFFFFFFFFFFFFFFBFFFFFFFFFFFFFF

@GWZHISEQ02:315:C9E6MANXX:5:1208:10104:37920

CTGTTCTTGGCCCTGAGCCGGGGCCCAGGTGCTCGACCACAGATATCCTGTTTGGCCCCTGTCCCAGTTTTGTTCAGCCTTATTCTTTAACTAAACTTCCTTGTGACTTTTGAGAACTCAGCTCTG

+

/BB/<BBF///BB<FBB<BB7/<BF7FFF<FFF/BFFFFFFBFFFFFFF/FBFB/<FB<<FF<FFFFBFBB/FBFF/FFFFF<BFFFFF/BFBF<FFFF<FFFFF</BFFFFBFFFFFF7/B7FBF

@GWZHISEQ02:315:C9E6MANXX:5:1211:4329:63767

CTGTTCTTGGCCCTGAGCCGGGGCCCAGGTGCTCGACCACAGATATCCTGTTTGGCCCCTGTCCCAGTTTTGTTCAGCCTTATTCTTTAACTAAACTTCCTTGTGACTTTTGAGAACTCAGCTCTG

+

BBBBBFFFFFFFFF<BFFFFFFFFFFFFFFFFFFFBBBBFFFFFFF<FBFFFFF<FFFFFFFFFFFFFFFFFFFFFFF/BFFFFBFFFFBFFF/7BFFFBBFFBB/BFFFB/FF7FFFF/BFF/F<

@GWZHISEQ02:315:C9E6MANXX:5:1213:17560:11820

CTGTTCTTGGCCCTGAGCCGGGGCCCAGGTGCTCGACCACAGATATCCTGTTTGGCCCCTGTCCCAGTTTTGTTCAGCCTTATTCTTTAACTAAACTTCCTTGTGACTTTTGAGAACTCAGCTCTG

+

BBBBBFFFFFFFFFFFFFFFFFFFFFFFFFFFFFFFFFFFFFFFFFFFFFFFFFFFFFFFFFFFFFFFFFFFFFFFFFFFFFFFFFFFFFFBFFFFFFFFFFFFFFFFFFFFFFFFFFFFFFFFFF

@GWZHISEQ02:315:C9E6MANXX:5:1311:6907:96522

CTGTTCTTGGCCCTGAGCCGGGGCCCAGGTGCTCGACCACAGATATCCTGTTTGGCCCCTGTCCCAGTTTTGTTCAGCCTTATTCTTTAACTAAACTTCCTTGTGACTTTTGAGAACTCAGCTCTG

+

BBBBBFFFFFFFFFFFFFFFFFFFFFFFFFFFFFFFFFFFFFFFFFFFFFFFFFFFFFFFFFFFFFFFFFFFFFFFFFFFFFFFFFFFFFFFFFFFFFFFFFFFFFFFFFFFFFFFFFFFFFFFFF

@GWZHISEQ02:315:C9E6MANXX:5:1312:10315:18970

CTGTTCTTGGCCCTGAGCCGGGGCCCAGGTGCTCGACCACAGATATCCTGTTTGGCCCCTGTCCCAGTTTTGTTCAGCCTTATTCTTTAACTAAACTTCCTTGTGACTTTTGAGAACTCAGCTCTG

+

BBBBBFFFFFFFFFFFFFFFFFBFFFFFFFFFFFFFFFFFFFBFFFFFFFFFFFFFFFFFFFFFFFFFBFFFFFFFFFFFFFFFFFFFFFFFFFFFFFFFFFFFFFFFFFFBFFFFFFFFFFFFFF

@GWZHISEQ02:315:C9E6MANXX:5:2101:17617:39420

CTGTTCTTGGCCCTGAGCCGGGGCCCAGGTGCTCGACCACAGATATCCTGTTTGGCCCCTGTCCCAGTTTTGTTCAGCCTTATTCTTTAACTAAACTTCCTTGTGACTTTTGAGAACTCAGCTCTG

+

BBBBBFFFFFFFFFFFFFFFFFFFFFFFFFFFFFFFFFFFFFFFFFFFFFFFFFBFFFFFFFFFFBFFFFFFFFFFFFFFFFFFFFFBFFFFFFFBFFFFFFFFFFFFFFFFFFFFBFFFF<BBFF

@GWZHISEQ02:315:C9E6MANXX:5:2102:6303:10811

CTGTTCTTGGCCCTGAGCCGGGGCCCAGGTGCTCGACCACAGATATCCTGTTTGGCCCCTGTCCCAGTTTTGTTCAGCCTTATTCTTTAACTAAACTTCCTTGTGACTTTTGAGAACTCAGCTCTG

+

BBBBBFFFFFFFFFFFFFFFFFFFFFFFFFFFFFFFFFFFFFFFFFFFFFFBFFFFFFFFFFFFFFFFFFFFFFFFFFFFFFFFFFFFFFFFFFFFFFFFFFFFFFFFFFFFFFFFFFFFFFFFFF

@GWZHISEQ02:315:C9E6MANXX:5:2107:1773:44719

CTGTTCTTGGCCCTGAGCCGGGGCCCAGGTGCTCGACCACAGATATCCTGTTTGGCCCCTGTCCCAGTTTTGTTCAGCCTTATTCTTTAACTAAACTTCCTTGTGACTTTTGAGAACTCAGCTCTG

+

BBBBBFFFFFFFFFFFFFFFFFFFFFFFFFFFFFFFFFFFFFFFFFFFFFFFFFFFFFFFFFFFFFFFFFFFFFFFFFFFFFFFFFFFFFFFFFFFFFFFFFFFFFFFFFFFFFFFFFFFFFFFFF

@GWZHISEQ02:315:C9E6MANXX:5:2116:8920:74107

CTGTTCTTGGCCCTGAGCCGGGGCCCAGGTGCTCGACCACAGATATCCTGTTTGGCCCCTGTCCCAGTTTTGTTCAGCCTTATTCTTTAACTAAACTTCCTTGTGACTTTTGAGAACTCAGCTCTG

+

BBBBBFFFFFFFFFFFFFFFFFFFFFFFFFFFFFFFFFFFFFFFFFFFFFFFFFFFFFFFFFFFFFFF<FFFFFFFFFFFFFFFFFFFFFF/7FFFFFFFFFFFFFFFFFFF7BFFFBBFF7/7FB

@GWZHISEQ02:315:C9E6MANXX:5:2201:9375:71367

CTGTTCTTGGCCCTGAGCCGGGGCCCAGGTGCTCGACCACAGATATCCTGTTTGGCCCCTGTCCCAGTTTTGTTCAGCCTTATTCTTTAACTAAACTTCCTTGTGACTTTTGAGAACTCAGCTCTG

+

BBBBBFFFF<FFFFBF/BBF7/FFBFFFFFFBFBF<FF<BFFFFFBFFFBFFFBFBBFFFFFBF/FB/FBFF<BFFB<//FFBF/BFFFFFFFFFBFFF<FBFFBFFFFFFFFFFFB//7BF/FFF

@GWZHISEQ02:315:C9E6MANXX:5:2202:13422:62213

CTGTTCTTGGCCCTGAGCCGGGGCCCAGGTGCTCGACCACAGATATCCTGTTTGGCCCCTGTCCCAGTTTTGTTCAGCCTTATTCTTTAACTAAACTTCCTTGTGACTTTTGAGAACTCAGCTCTG

+

BBBBBFFFFFFFFFFFFFFFFFFFFFFFFFFFFFFFFFFFFFFFFFFFFFFFFFFFFFFFFFFFFFFFFFFFFFFFFFFFFFFFFFFFFFFFFFFFFFFFFFFFFFFFFFFFFFFFFFFFFFFFFF

@GWZHISEQ02:315:C9E6MANXX:5:2204:14303:20276

CTGTTCTTGGCCCTGAGCCGGGGCCCAGGTGCTCGACCACAGATATCCTGTTTGGCCCCTGTCCCAGTTTTGTTCAGCCTTATTCTTTAACTAAACTTCCTTGTGACTTTTGAGAACTCAGCACTG

+

/<<<</B//</</<FFFFF/F7/////FF</<BB7FFF/FFBBFBFFB/FBF<FF</FFFF//<BFFF/FF<BBB/F<F/B////<//<F/<//<7<F////<<B/<<<F<</7//BBBFBF/7B/

@GWZHISEQ02:315:C9E6MANXX:5:2206:16411:4135

CTGTTCTTGGCCCTGAGCCGGGGCCCAGGTGCTCGACCACAGATATCCTGTTTGGCCCCTGTCCCAGTTTTGTTCAGCCTTATTCTTTAACTAAACTTCCTTGTGACTTTTGAGAACTCAGCTCTG

+

BBBBBFFFFFFFFFFFFFFFFFFFFFFFFFFFFFFFFFFFFFFFFFFFFFFFFFFFFFFFFFFFFFFFFFFFFFFFFFFFFFFFFFFFFFFFFFFFFFFFFFFFFFFFFFFFFF<FFFFFFFFFFF

@GWZHISEQ02:315:C9E6MANXX:5:2208:17557:66807

CTGTTCTTGGCCCTGAGCCGGGGCCCAGGTGCTCGACCACAGATATCCTGTTTGGCCCCTGCCCCAGTTTTGTTCAGCCTTATTCTTTAACTAAACTTCCTTGTGACTTTTGAGAACTCAGCTCTG

+

BBBBBFFFFFFFFFFFFFFFFBFFFFFFFFFFFFFFFFFFFFFFFFFFFFFFFFFFFFFFF<FFFFFFFFFFFFFFFFFFFFFFFFFFFFFFFFFFFFFFFFFFFBFFFFFFFFFFFFFFFFFFFF

@GWZHISEQ02:315:C9E6MANXX:5:2209:12927:13938

CTGTTCTTGGCCCTGAGCCGGGGCCCAGGTGCTCGACCACAGATATCCTGTTTGGCCCCTGTCCCAGTTTTGTTCAGCCTTATTCTTTAACTAAACTTCCTTGTGACTTTTGAGAACTCAGCTCTG

+

BBBBBFBFFFFFFFFFFFFFFFFFFFFFFFFFFFFFFFFFFFFFFFFFFFFFFFBFFFFFFFFFFFFFFFFFFFFFFFFFFFFFFFFFFFFFFFFFFFFFFFFFFFFFFFFFBFFFFFFFFFFFFF

@GWZHISEQ02:315:C9E6MANXX:5:2209:17996:26852

CTGTTCTTGGCCCTGAGCCGGGGCCCAGGTGCTCGACCACAGATATCCTGTTTGGCCCCTGTCCCAGTTTTGTTCAGCCTTATTCTTTAACTAAACTTCCTTGTGACTTTTGAGAACTCAGCTCTG

+

BBBBBFFFFBFFFFFFFFFFFF<FFFFFFFFFFFFFFFFFFF<FFFFFFFF/FFBFFFFFFFFFFFF<FFFFBFFFFBFFF<FFFFBBFFFFFFFFFFFFFFFFFBFBBFFF<FBFB<FFFFFFFF

@GWZHISEQ02:315:C9E6MANXX:5:2210:8561:31456

CTGTTCTTGGCCCTGAGCCGGGGCCCAGGTGCTCGACCACAGATATCCTGTTTGGCCCCTGTCCCAGTTTTGTTCAGCCTTATTCTTTAACTAAACTTCCTTGTGACTTTTGAGAACTCAGCTCTG

+

BBBBBFFFFFFFFFFFFFFFBF<FFFFFFFFFFFFFFFFFFFFFFFFFFFFFFFFFFFFFFFFFFFF<FFFFFFFFFFFFFFFFFFFFFFFFFFFFFFFFFBFFFFFFFFFFFFFFFFFFFFFFF<

@GWZHISEQ02:315:C9E6MANXX:5:2211:2926:71016

CTGTTCTTGGCCCTGAGCCGGGGCCCAGGTGCTCGACCACAGATATCCTGTTTGGCCCCTGTCCCAGTTTTGTTCAGCCTTATTCTTTAACTAAACTTCCTTGTGACTTTTGAGAACTCAGCTCTG

+

BBBBBFFFFFFFFFFFFFFFFFFFFFFFFFFFFFFFFFFFFFFFFFFFFFFFFFFFFFFFFFFFFFFFFFFFFFFFFFFFFFFFFFFFF/BFFF<FFFFFFFFFFFFFFFFBFFFFFFFFFFFFFF

@GWZHISEQ02:315:C9E6MANXX:5:2211:5449:95735

CTGTTCTTGGCCCTGAGCCGGGGCCCAGGTGCTCGACCACAGATATCCTGTTTGGCCCCTGTCCCAGTTTTGTTCAGCCTTATTCTTTAACTAAACTTCCTTGTGACTTTTGAGAACTCAGCTCTG

+

BBBBBFFFFFFFFFFFFFFBBFFFFFFFFFFFFFFBFFFFFFFFFFFFFFFFF<FFFFFFFFFFFFFFBFFFFFFFFFFFFFFFFFFFBFFFFBFFFFFFFFFFFFFFFFFFFF<FFFFBFFFFFF

@GWZHISEQ02:315:C9E6MANXX:5:2215:10434:22235

CTGTTCTTGGCCCTGAGCCGGGGCCCAGGTGCTCGACCACAGATATCCTGTTTGGCCCCTGTCCCAGTTTTGTTCAGCCTTATTCTTTAACTAAACTTCCTTGTGACTTTTGAGAACTCAGCTCTG

+

BBBBBFFFFFFFFFFFFFFFFFFFFFFFFFFFFFFFFFFFFFFFFFFFFFFFFFFFFFBFFFFFFFFFFFFFFFFFFFFFFFFFFFFFFFFFFFFFFFFFFFFFFFFFFFFFFFFFFFFFFFFFFF

@GWZHISEQ02:315:C9E6MANXX:5:2301:4293:69538

CTGTTCTTGGCCCTGAGCCGGGGCCCAGGTGCTCGACCACAGATATCCTGTTTGGCCCCTGTCCCAGTTTTGTTCAGCCTTATTCTTTAACTAAACTTCCTTGTGACTTTTGAGAACTCAGCTCTG

+

BBBBBFFFFBFFFBFFFFFFFFFFFFFFFFFFFFFFFFFFFFF<FFFFFFFFBFFFFFFFFFFFFFF/FFFFFBFBB<FF<F<FFFFFFFFFFFFFFFFFFFFFFFFF<FFF<FFFFFFF<FFFFF

@GWZHISEQ02:315:C9E6MANXX:5:2308:7407:16534

CTGTTCTTGGCCCTGAGCCGGGGCCCAGGTGCTCGACCACAGATATCCTGTTTGGCCCCTGTCCCAGTTTTGTTCAGCCTTATTCTTTAACTAAACTTCCTTGTGACTTTTGAGAACTCAGCTCTG

+

BBBBBFFFFFFFFFBFFBFFFFFFFFFFFFFFFFFFFFFFFFFFFFFFFFFFFBFFFFFFFFFFFFFFFFFFFFFFFFFFFFFFFFFFFFFFFFFFFFFFFFFFFFFFFFFFFFFFFFFFFFFFFF

@GWZHISEQ02:315:C9E6MANXX:5:2308:1258:70613

CTGTTCTTGGCCCTGAGCCGGGGCCCAGGTGCTCGACCACAGATATCCTGTTTGGCCCCTGTCCCAGTTTTGTTCAGCCTTATTCTTTAACTAAACTTCCTTNTGACTTTTNNNNACTCAGCTCTG

+

BB/BBFFFFFFFFFFFFFFFFFFFFFFFFFFF/BBBFFFFF</FFFFBBBBBBBFFFFFFFFFFFFFFBFFFF/FFFFFFFFFFFFFFFFFFFFFFFFFFFF#/</F/FFF####<<77FFBFFFF

@GWZHISEQ02:315:C9E6MANXX:5:2312:11893:57086

CTGTTCTTGGCCCTGAGCCGGGGCCCAGGTGCTCGACCACAGATATCCTGTTTGGCCCCTGTCCCAGTTTTGTTCAGCCTTATTCTTTAACTAAACTTCCTTGTGACTTTTGAGAACTCAGCTCTG

+

</B<//<F</F/FFB<<BF<FBF<F<FF/<//BBFBBFFFFFBFFFFFFFFFFF//F/<F/FFFFBB<FB/FFFFFFBBF<FFFBFFFFF<F/FFFFFFFFF<FF/<7BFFFFFB/FFF/FBFBB<

@GWZHISEQ02:315:C9E6MANXX:5:2314:16845:83816

CTGTTCTTGGCCCTGAGCCGGGGCCCAGGTGCTCGACCACAGATATCCTGTTTGGCCCCTGTCCCAGTTTTGTTCAGCCTTATTCTTTAACTAAACTTCCTTGTGACTTTTGAGAACTCAGCTCTG

+

BBBBBFFFFFFFFF<FFFFFFFFFFFFFFFFFFFFFFFFFFFFFFFFBFBFFFFFFFFFFFFFFFFFFFFFFFFFFFFFFFFFFFFFFFFFFBFFFFFFFFFFFFFFFFFFFFFFFFFFFFFFFFF

@GWZHISEQ02:315:C9E6MANXX:5:1202:12709:27306

AGAGCTGAGTTCTCAAAAGTCACAAGGAAGTTTAGTTAAAGAAAAAGGCTGAACAAAACTGGGACAGGGGCCAAACAGGATATCTGTGGTCGAGCACCTGGGCCCCGGCTCAGGGCCAAGAACAGA

+

BBBBBFFFFFFFFFFFFFFFFFFFFFFFFFFFFFFFFFFFFFFFFFFFFFFFFFFFFFFFFFFFFFFFFBFFFFFFFFFBFFFFFFFFFFFFFFFFFFFFFFFFFFFBFFFFFFFFFFFFFFFFF7

@GWZHISEQ02:315:C9E6MANXX:5:2113:9606:29266

AGAGCTGAGTTCTCAAAAGTCACAAGGAAGTTTAGTTAAAGAATAAGGCTGAACAAAACTGGGACAGGGGCCAAACAGGATATCTGTGGTCGAGCACCTGGGCCCCGGCTCAGGGCCAAGAACAGA

+

<<BBBFF/BFFFFF<F<FFFFFFFFFFFFBFFFFFFFFFFFFFFFFBBFFFFFFFFFFFFFBFFFFFB/B/FFBBFFFBFFFFFFFFFFFFFFFFFFFFFFFFFFFFFFFFFFFFFFFFFBFFFFB

@GWZHISEQ02:315:C9E6MANXX:5:2305:4909:42083

AGAGCTGAGTTCTCAAAAGTCACAAGGAAGTTTAGTTAAAGAATAAGGCTGAACAAAACTGGGACAGGGGCCAAACAGGATATCTGTGGTCGAGCACCTGGGCCCCGGCTCAGGGCCAAGAACAGA

+

BBBBBFFFFFFFFFFFFFFFFFFFFFFFFFFFFFFFFFFFFFFFFFFFFFFFFFFFFFFFFFFFFFFFFFFFFFFFFFFFFFFFFFFFFFFFFFFFFFFFFFFFFFFFFFFFFFFFFFFFFFFFFF

@GWZHISEQ02:315:C9E6MANXX:5:1204:18174:43750

GAGCTGAGTTCTCAAAAGTCACAAGGAAGTTTAGTTAAAGAATAAGGCTGAACAAAACTGGGACAGGGGCCAAACAGGATATCTGTGGTCGAGCACCTGGGCCCCGGCTCAGGGCCAAGAACAGAT

+

BBBBBFFFFFFFFFFFFFFFFFFFFFFFFFFFFFFFFFFFFFFFFFFFFFFFFFFFFFFFFFFFFFFFFFFFFFFFFFFFFFFFFFFFFFFFFFFFFFFFFFFFFFFFFFFFFFFFFFFFFFFFFF

@GWZHISEQ02:315:C9E6MANXX:5:1210:16530:56598

GAGCTGAGTTCTCAAAAGTCACAAGGAAGTTTAGTTAAAGAATAAGGCTGAACAAAACTGGGACAGGGGCCAAACAGGATATCTGTGGTCGAGCACCTGGGCCCCGGCTCAGGGCCAAGAACAGAT

+

BBBBBFFFFFFFFFFFFFFFBFFFBFFFFFFFFFFFFFFFFFFFFFFFFFFFFFFFFFFFFFFFFFFFBFBFFBBFFFF<FFFFFFFFFFFFFFFFFFFFFFBFFFFFFFFFFFFFFFFFFFFFF/

@GWZHISEQ02:315:C9E6MANXX:5:1213:11410:80714

GAGCTGAGTTCTCAAAAGTCACAAGGAAGTTTAGTTAAAGAATAAGGCTGAACAAAACTGGGACAGGGGCCAAACAGGATATCTGTGGTCGAGCACCTGGGCCCCGGCTCAGGGCCAAGAACAGAT

+

BBBBBFFFFFFFFFFFFFFFFFFFFFFFFFFFFFFFFFFFFFFFFFFFFFFFFFFFFFFFFFFFFFFFFFFFFFFFFFFFFFFFFFFFFFFFFFFFFFFFFFFFFFFFFFFFFFFFFFFFFFFFFF

@GWZHISEQ02:315:C9E6MANXX:5:2305:19809:56298

GAGCTGAGTTCTCAAAAGTCACAAGGAAGTTTAGTTAAAGAATAAGGCTGAACAAAACTGGGACAGGGGCCAAACAGGATATCTGTGGTCGAGCACCTGGGCCCCGGCTCAGGGCCAAGAACAGAT

+

BBBBBF/FFFBFFFFF</F<FFFFFFFFFFFFFFFFFFFFF/FFFFFFFFFBFFFFFFFFFFBFF</FFFFFFFFFFFFFFFFFFFFBBFFFFFFFFFFBFF<FFFFFFFFF<FBFFFFF<FFFF/

@GWZHISEQ02:315:C9E6MANXX:5:1110:6610:2315

CTGTTCTTGGCCCTGAGCCGGGGCCCAGGTGCTCGACCACAGATATCCTGTTTGGCCCCTGTCCCAGTTTTGTTCAGCCTTATTCTTTAACTAAACTTCCTTGTGACTTTTGAGAACTCAGCTCAG

+

BBBBBFFFFFFFFFFFFFFFFFFFFFFFFFFFFFFFFFFFFFFFFFFFFFFFFFFFFFFFFFFFFFFFFFFFFFFFFFFFFFFFFFFFFFFFFFFFFFFFFFFFFFFFFFFFFFFFFFFFFFFFFF

@GWZHISEQ02:315:C9E6MANXX:5:1210:10512:66021

ATCTGTTCTTGGCCCTGAGCCGGGGCCCAGGTGCTCGACCACAGATATCCTGTTTGGCCCCTGTCCCAGTTTTGTTCAGCCTTATTCTTTAACTAAACTTCCTTGTGACTTTTGAGAACTCAGCTC

+

BBBBBFFFFFFFFFFFFFFFFFFFFFFFFFFFFFFFFFFFFFFFFFFFFFFFFFFFFFFFFFFFFFFFFFBFFFFFFFFFFFFFFFFFFFFFFFFFFFFFFFFFFFFFFFFFFFFFFFFFFFFFFF

@GWZHISEQ02:315:C9E6MANXX:5:1216:5011:58502

ATCTGTTCTTGGCCCTGAGCCGGGGCCCAGGTGCTCGACCACAGATATCCTGTTTGGCCCCTGTCCCAGTTTTGTTCAGCCTTATTCTTTAACTAAACTTCCTTGTGACTTTTGAGAACTCAGCTC

+

/</<B/F<FFF/<<FFBFFBF</B<FBFF<<FFF/FBFFBB<FFFFFFFFFFFFFB//FFF/<FBFFFF<FBBBFFFBFBFFFFFFFFBBFFF<FFFFF/BFFFBFBBFFBFFB<F<FFBB/F<FF

@GWZHISEQ02:315:C9E6MANXX:5:2201:3749:60623

ATCTGTTCTTGGCCCTGAGCCGGGGCCCAGGTGCTCGACCACAGATATCCTGTTTGGCCCCTGTCCCAGTTTTGTTCAGCCTTATTCTTTAACTAAACTTCCTTGTGACTTTTGAGAACTCAGCTC

+

BBBBBFFFFFFFFBFBFFFFFFFFFFFFFFFFFFFFFFFFFFFFFFFFFFFFFFFFFFFFFFFFFFFBFFFFFFFFFFFFFFFFFFFFFFFFFFFFFFFFFFFFFFFFFFFFFBFFBFFFFFFFFF

@GWZHISEQ02:315:C9E6MANXX:5:2215:18143:25615

ATCTGTTCTTGGCCCTGAGCCGGGGCCCAGGTGCTCGACCACAGATATCCTGTTTGGCCCCTGTCCCAGTTTTGTTCAGCCTTATTCTTTAACTAAACTTCCTTGTGACTTTTGAGAACTCAGCTC

+

BB/BBFFFFFFFF<BFFFFF/BB</BFFFFFFFFFFFFFBF</<<BBFBFFFFFFBBFFF<FFFF/FBBFFFFFFBB/FF/FFFBFFBFFFFFFFFFFFFBFFFFFF7FFBFFFFF<FFFFFF//7

@GWZHISEQ02:315:C9E6MANXX:5:1102:2091:83184

AGCTGAGTTCTCAAAAGTCACAAGGAAGTTTAGTTAAAGAATAAGGCTGAACAAAACTGGGACAGGGGCCAAACAGGATATCTGTGGTCGAGCACCTGGGCCCCGGCTCAGGGCCAAGAACAGATG

+

BBBBBFFFFFFFFFFFFFFFFFFFFFFFFFFFFFFFFFFFFFFFFFFFFFFFFFFFFFFFFFFFFFFFFFFFFFFFFFFFFFFFFFFFFFFFFFFFFFFFFFFFFFFFFFFFFFFFFFFFFFFFFF

@GWZHISEQ02:315:C9E6MANXX:5:1106:6134:23612

AGCTGAGTTCTCAAAAGTCACAAGGAAGTTTAGTTAAAGAATAAGGCTGAACAAAACTGGGACAGGGGCCAAACAGGATATCTGTGGTCGAGCACCTGGGCCCCGGCTCAGGGCCAAGAACAGATG

+

BBBBBFFFFFFFFFFFFFFFFFFFFFFFFFFFFFFFFFFFFFFFFFFFFFFFFFFFFFFFFFFFFFFFFFFFFFFFFFFFFFFFFFFFFFFFFFFFFFFFFFFFFFFFFFFFFFFFFFFFFFFFFF

@GWZHISEQ02:315:C9E6MANXX:5:1207:10791:79950

AGCTGAGTTCTCAAAAGTCACAAGGAAGTTTAGTTAAAGAATAAGGCTGAACAAAACTGGGACAGGGGCCAAACAGGATATCTGTGGTCGAGCACCTGGGCCCCGGCTCAGGGCCAAGAACAGATG

+

BBBBBFFFFFFFFFFFFFFFFFFFFFFFFFFFFFFFFFFFFFFFFFFFFFFFFFFFFFFFFFFFFFFFFFFFFFFFFFFFFFFFFFFFFFFFFFFFFFFFFFFFFFBFFFFFFFFFFFFFFFFFFF

@GWZHISEQ02:315:C9E6MANXX:5:1305:3635:72311

AGCTGAGTTCTCAAAAGTCACAAGGAAGTTTAGTTAAAGAATAAGGCTGAACAAAACTGGGACAGGGGCCAAACAGGATATCTGTGGTCGAGCACCTGGGCCCCGGCTCAGGGCCAAGAACAGATG

+

BBBBBFFFFFFFFFFFFFFFFFFFFFFFFFFFFFFFFFFFFFFFFFFFFFFFFFFFFFFFFFFFFFFFFFFFFFFFFFFFFFFFFFFFFFFFFFFFFFFFFFFFFFFFFFFFFFFFFFFFFFFFFF

@GWZHISEQ02:315:C9E6MANXX:5:1309:1712:83244

AGCTGAGTTCTCAAAAGTCACAAGGAAGTTTAGTTAAAGAATAAGGCTGAACAAAACTGGGACAGGGGCCAAACAGGATATCTGTGGTCGAGCACCTGGGCCCCGGCTCAGGGCCAAGAACAGATG

+

BBBBBFFFFFFFFFFFFFFFFFFFFFFFFFFFFFFFFFFFFFFFFFFFFFFFFFBFFFFFFFFFFFFFFFFFFFFFFFFFFFFFFFFFFFFFFFFFFFFFFFFFFFFFFFFFFF/FFFFFFFFFFF

@GWZHISEQ02:315:C9E6MANXX:5:2109:11577:30851

AGCTGAGTTCTCAAAAGTCACAAGGAAGTTTAGTTAAAGAATAAGGCTGAACAAAACTGGGACAGGGGCCAAACAGGATATCTGTGGTCGAGCACCTGGGCCCCGGCTCAGGGCCAAGAACAGATG

+

BB<BBFFFFFFFFFFFBFBB/FFFFFFFFFFFFF<FBFBF/FFBFFFFFFFFFF/BFFFFF<FFFBFB<FFBFFFFFFFB<FFFFFFFBFFFFBF<FBBBBFFFFFFF/<FFFFFFFFBFBFFFBF

@GWZHISEQ02:315:C9E6MANXX:5:2110:8027:40438

AGCTGAGTTCTCAAAAGTCACAAGGAAGTTTAGTTAAAGAATAAGGCTGAACAAAACTGGGACAGGGGCCAAACAGGATATCTGTGGTCGAGCACCTGGGCCCCGGCTCAGGGCCAAGAACAGATG

+

BBBBBFFFFFFFFFFFFFFFFFFFFFFFFFFFFFFFFFFFFFFFFFFFFFFFFFFFFFFFFFFFFFFFFFFFFFFFFFFFFFFFFFFFFFFFFFFFFFFFFFFFFFFFFFFFFFFFFFFFFFFFFF

@GWZHISEQ02:315:C9E6MANXX:5:2206:2092:20976

AGCTGAGTTCTCAAAAGTCACAAGGAAGTTTAGTTAAAGAATAAGGCTGAACAAAACTGGGACAGGGGCCAAACAGGATATCTGTGGTCGAGCACCTGGGCCCCGGCTCAGGGCCAAGAACAGATC

+

BBBBBFFFF<FFFFFFFFFFFFFFFFFFFFFFFF<FFFBFFFFFFFFFFFFFFF<FFFFFFFFFFFBFFFFFFFFFFFFFFFFFFFFFFFFFFFFFFFFFFFBFFFFFFFFFFFFFFFBFFFFFFF

@GWZHISEQ02:315:C9E6MANXX:5:2207:3378:3589

AGCTGAGTTCTCAAAAGTCACAAGGAAGTTTAGTTAAAGAATAAGGCTGAACAAAACTGGGACAGGGGCCAAACAGGATATCTGTGGTCGAGCACCTGGGCCCCGGCTCAGGGCCAAGAACAGATG

+

BBBBBFFFFFFFFFFFFFFFFFFFFFFFFFFFFFFFFFFFFFFFFFFFFFFFFFFFFFFFFFFFFFFFFFFFFFFFFFFFFFFFFFFFFFFFFFFFFFFFFFFFFFFFFFFFFFFFFFFFFFFFFF

@GWZHISEQ02:315:C9E6MANXX:5:2309:20283:61867

AGCTGAGTTCTCAAAAGTCACAAGGAAGTTTAGTTAAAGAATAAGGCTGAACAAAACTGGGACAGGGGCCAAACAGGATATCTGTGGTCGAGCACCTGGGCCCCGGCTCAGGGCCAAGAACAGATG

+

BBBBBFFFFFFFFFFFFFFFFFFFFFFFFFFFFFFFFFFFFFFFFFFFFFFFFFFFFFFFFFFFFFFFFFFFFFFFFFFFFFFFFFFFFFFFFFFFFFFFFFFFFFFFFFFFFFFFFFFFFFFBFF

@GWZHISEQ02:315:C9E6MANXX:5:2314:20755:20151

AGCTGAGTTCTCAAAAGTCACAAGGAAGTTTAGTTAAAGAATAAGGCTGAACAAAACTGGGACAGGGGCCAAACAGGATATCTGTGGTCGAGCACCTGGGCCCCGGCTCAGGGCCAAGAACAGATG

+

BBBBBFFFFFFFFFFFFFFFFFFFFFFFFFFFFFFFFFFFFFFFFFFFFFFFFFFFFFFFFFFFFFFFFF<FFFFFFFFFFFFFFFFFFFFFFFFFFFFFFFFFFFBBFF<FFFFFFFFFFFFFFF

@GWZHISEQ02:315:C9E6MANXX:5:2206:2092:20976

GTTCTTGGCCCTGAGCCGGGGCCCAGGTGCTCGACCACAGATATCCTGTTTGGCCCCTGTCCCAGTTTTGTTCAGCCTTATTCTTTAACTAAACTTCCTTGTGACTTTTGAGAACTCAGCTAGATC

+

BBBBBFFFFFFFFFFFFFFFFFFFFFFFFFFFFFFFFFFFFFFFFFFFFFFFFFFFFFFFFFFFFFBBFFFFFFFFFFFFFFFFFFFFFFFFFFFFFFFFFFFFFFFFFFFFFFFFFFFFFFFFFF

@GWZHISEQ02:315:C9E6MANXX:5:1205:16446:72163

GCTGAGTTCTCAAAAGTCACAAGGAAGTTTAGTTAAAGAATAAGGCTGAACAAAACTGGGACAGGGGCCAAACAGGATATCTGTGGTCGAGCACCTGGGCCCCGGCTCAGGGCCAAGAACAGATGG

+

BBBBBFFFFFFFFFFFFFFFFFFFFFFFFFFFFFFFFFFFFFFFFFFFFFFFFFFFFFFFFFFFFFFFFFFFFFFFFFFFFFFFFFFFFFFFFFFFFFFFFFFFFFFFFFFFFFFFFFFFFFFFFF

@GWZHISEQ02:315:C9E6MANXX:5:1306:1643:48303

CCATCTGTTCTTGGCCCTGAGCCGGGGCCCAGGTGCTCGACCACAGATATCCTGTTTGGCCCCTGTCCCAGTTTTGTTCAGCCTTATTCTTTAACTAAACTTCCTTGTGACTTTTGAGAACTCAGC

+

BBBB<BBFFFFFFFFBFFFFFFBFFBBBFBFFF<BFFFFFFFFFFFFFFFFFFFFFFF/FFFFFBFFFFFBBFB<BFB</7<//<<B7BFBFFFFBFFFFBFBBFFFFFBF<FBFF/<B7/7/FBF

@GWZHISEQ02:315:C9E6MANXX:5:1311:4301:20251

CTGTTCTTGGCCCTGAGCCGGGGCCCAGGTGCTCGACCACAGATATCCTGTTTGGCCCCTGTCCCAGTTTTGTTCAGCCTTATGCTTTAACTAAACTTCCTTGTGACTTTTGAGAACGCAGCAGAT

+

BBBBBFFFFFFFFFFFFFFFFFFFFFFFFFFFFFFFFFFFFBFFFFFFF/BFF<FB/</BFFFFFFBFFBFBF/<F/BB/F/</</7F7//7FF/<B/7F/<F/7<</<BB/777F7//77BF/7F

@GWZHISEQ02:315:C9E6MANXX:5:2310:19910:36731

CCATCTGTTCTTGGCCCTGAGCCGGGGCCCAGGTGCTCGACCACAGATATCCTGTTTGGCCCCTGTCCCAGTTTTGTTCAGCCTTATTCTTTAACTAAACTTCCTTGTGACTTTTGAGAACTCAGC

+

BBBBBFFFFFFFFFFFFFFFFFFFFFFFFFFFFFFFFFFFFFFFFFFFFFFFFFFFFFFFFFFFFFFFFFFFFFFFFFFFFFFFFFFFFFFFFFFFFFFFFFFFFFFFFFFFFFFFFFFFFFFFFF

@GWZHISEQ02:315:C9E6MANXX:5:1205:4359:29236

CTGAGTTCTCAAAAGTCACAAGGAAGTTTAGTTAAAGAATAAGGCTGAACAAAACTGGGACAGGGGCCAAACAGGATATCTGTGGTCGAGCACCTGGGCCCCGGCTCAGGGCCAAGAACAGATGGT

+

BBBBBFFFFFFFFFFFFFFFFFBFFFFFFFFFFFFFFFFFFFBFFFFFFFFFFFFBFFBFFFFFFFFFFFFFFFFFFFFFFFFFFFFFFFFFFFFFFFFFFFFBFFBFFFFFFFFFFFFFF7BF//

@GWZHISEQ02:315:C9E6MANXX:5:2215:6294:71442

CTGAGTTCTCAAAAGTCACAAGGAAGTTTAGTTAAAGAATAAGGCTGAACAAAACTGGGACAGGGGCCAAACAGGATATCTGTGGTCGAGCACCTGGGCCCCGGCTCAGGGCCAAGAACAGATGGT

+

BBBBBFFFFFFFFFFFFFFFFFFFFFFFFFFFFFFFFFFFFFFFFFFFFFFFFFFFFFFFFFFFFFFFFFFFFFFFFFFFFFFFFFFFFFFFFFFFFFFFFFFFFFFFFFFFFFFFFFFFFFFFF7

@GWZHISEQ02:315:C9E6MANXX:5:2215:10533:74465

CTGAGTTCTCAAAAGTCACAAGGAAGTTTAGTTAAAGAATAAGGCTGAACAAAACTGGGACAGGGGCCAAACAGGATATCTGTGGTCGAGCACCTGGGCCCCGGCTCAGGGCCAAGAACAGATGGT

+

BBBBBFFFFFBFFFFBFFFFFFFFFFFFFFFFFFFF/FFBF/F<FF<BFFFFBFFFBFFBFFFFFFB<FBFF<FBBFF/<<BF<FFBFFFFFFFBB<FFFFFFFF7FF<<FFF</F/FB/FBFFF7

@GWZHISEQ02:315:C9E6MANXX:5:1103:12691:55842

GCCATCTGTTCTTGGCCCTGAGCCGGGGCCCAGGTGCTCGACCACAGATATCCTGTTTGGCCCCTGTCCCAGTTTTGTTCAGCCTTATTCTTTAACTAAACTTCCTTGTGACTTTTGAGAACTCAG

+

BBBBBFFFFBFBFFFBFFFFF<FFFFFBBBFBFFFFFFFFFFFFFFFFFFFFFFFFFFFFFFFFFFFFFFBFFFFFFFF<FFFFFFFFFFFFFFFFFFFFFFFFFFFFFBF7FFFFFFFFF<FFF<

@GWZHISEQ02:315:C9E6MANXX:5:1209:10562:24916

GCCATCTGTTCTTGGCCCTGAGCCGGGGCCCAGGTGCTCGACCACAGATATCCTGTTTGGCCCCTGTCCCAGTTTTGTTCAGCCTTATTCTTTAACTAAACTTCCTTGTGACTTTTGAGAACTCAG

+

/BBBBFFFFFFFFFFFFFFFFFFFFFBFFFFBFFFFFFFFFFFBFFFFFFFFFFFFFFFFFFFFFFFFFFFFFFFFFFFFFFFFFFFFFFFFFFFFFFFBFFFFFFFFFFFFFFFFFB/FFFFFFF

@GWZHISEQ02:315:C9E6MANXX:5:1211:6079:37674

GCCATCTGTTCTTGGCCCTGAGCCGGGGCCCAGGTGCTCGACCACAGATATCCTGTTTGGCCCCTGTCCCAGTTTTGTTCAGCCTTATTCTTTAACTAAACTTCCTTGTGACTTTTGAGAACTCAG

+

BB<<BFFFFFFFFBBFFFFFF<FFF<FFBFFFFFFFFFFFFFFFFFF/F/BF<BFFFFFFF/B<FFFBF<FFFFFFFFFFBFFFFFFBFFFFFFFFBFF<BFFFFFFFFFFFFFFFBBFB<<FBB/

@GWZHISEQ02:315:C9E6MANXX:5:1303:11662:96265

GCCATCTGTTCTTGGCCCTGAGCCGGGGCCCAGGTGCCCGACCACAGATATCCTGTTTGGCCCCTGTCCCAGTTTTGTTCAGCCTTATTCTTTAACTAAACTTCCTTGTGACTTTTGAGAACTCAG

+

BBBBBFFFFFFFFFFFFFFFFFFFFFFFFFFBFFFFFFFFFFFFFFFFFFFFFFFFFFFFFFFFFFFFFFFFFFFFFFBFFFFFFFFFFFFFFFBFFFFFFFFFFFFFFFFFFFFFFFFFFFFFBB

@GWZHISEQ02:315:C9E6MANXX:5:1305:4518:10308

ACCATCTGTTCTTGGCCCTGAGCCGGGGCCCAGGTGCTCGACCACAGATATCCTGTTTGGCCCCTGTCCCAGTTTTGTTCAGCCTTATTCTTTAACTAAACTTCCTTGTGACTTTTGAGAACTCAG

+

BBBBBFFFFFFFFFFFFFFFFFFFF/FFFFF/BFFFFFFFFFFFF/<FFFFFFFFFFFFBFFFFFFFFFFFFFFFFFFFFFFFFBFFFFFFBFFFFFFFFF<BFBBFFBF/7FFFFFFF<FFFB/B

@GWZHISEQ02:315:C9E6MANXX:5:1312:3068:61381

ACCATCTGTTCTTGGCCCTGAGCCGGGGCCCAGGTGCTCGACCACAGATATCCTGTTTGGCCCCTGTCCCAGTTTTGTTCAGCCTTATTCTTTAACTAAACTTCCTTGTGACTTTTGAGAACTCAG

+

BBBBBFFFFFFFFFFFFFFFFFFFFFFFFFFFFFFFFFFFFFFFFFBFFFFFFFFFFFFFFFFFFFFFFFFFFFFFFFFFFFFFFFFFFFFFFFFFFFFFFFFFFFFFFFFFFFFFF/FFBFFFBB

@GWZHISEQ02:315:C9E6MANXX:5:2113:13123:71634

ACCATCTGTTCTTGGCCCTGAGCCGGGGCCCAGGTGCTCGACCACAGATATCCTGTTTGGCCCCTGTCCCAGTTTTGTTCAGCCTTATTCTTTAACTAAACTTCCTTGTGACTTTTGAGAACTCAG

+

<BBBBFFFFFFFFFFFFFFFFFFFFFBFFFFFFFFFFFFFFFFFFBFFFFFFFFFFFFBBFFFFFFFFFFBFBFFFFFFFBF<FFFFFFFFFFFFFFFFFFFFFFFFFFBFFFFFFFFFFFFFFF/

@GWZHISEQ02:315:C9E6MANXX:5:2314:4855:45797

ACCATCTGTTCTTGGCCCTGAGCCGGGGCCCAGGTGCTCGACCACAGATATCCTGTTTGGCCCCTGTCCCAGTTTTGTTCAGCCTTATTCTTTAACTAAACTTCCTTGTGACTTTTGAGAACTCAG

+

BBBBBFFFFFFFFBB/FFFFFFFFFFFBFFFFFFFFFFF/BF<F<FFB///FBFFFFFB<FFB//F<F/FB</<B/FB/7FFFFFFFFFFFFFBFBFFBFFF<FFFFFFFBFF/F/<7F/BFFFFB

@GWZHISEQ02:315:C9E6MANXX:5:2211:8591:66546

TGAGTTCTCAAAAGTCACAAGGAAGTTTAGTTAAAGAATAAGGCTGAACAAAACTGGGACAGGGGCCAAACAGGATATCTGTGGTCGAGCACCTGGGCCCCGGCTCAGGGCCAAGAACAGATGGTA

+

BBBBBFFFFFFFFFFFFFFFFFFFFFFFFFFFFFFFFFFFFFFFFFFFFFFFFFFFFFFFFFFFFFFFFFFFFFFFFFFFFFFFFFFFFFFFFFFFFFFFFFFFFFFFFFFFFFFFFFFFFFFFFB

@GWZHISEQ02:315:C9E6MANXX:5:1115:5890:100226

GAGTTCTCAAAAGTCACAAGGAAGTTTAGTTAAAGAATAAGGCTGAACAAAACTGGGACAGGGGCCAAACAGGATATCTGTGGTCGAGCACCTGGGCCCCGGCTCAGGGCCAAGAACAGATGGTAC

+

BBBBBFFFBFBFBBFFFFFFFFFFFFFFFFFFFFFFFFFFFFFFFFFFFFFFFFFFFFFFFFFFFFFFFFFFFFFFFFFFFFFFFFFFFFFFFFFFFFFFFFFFFFFFFFFFFFFFFFFFFFFFFF

@GWZHISEQ02:315:C9E6MANXX:5:1316:19215:101040

GTACCATCTGTTCTTGGCCCTGAGCCGGGGCCCAGGTGCTCGACCACAGATATCCTGTTTGGCCCCTGTCCCAGTTTTGTTCAGCCTTATTCTTTAACTAAACTTCCTTGTGACTTTTGAGAACTC

+

BBBBBFFFFFFFFFFFFFFFFFFFFFFFFFFFFFFFFFFFFFFFFFFFFFFFFFFFFFFFFFFFFFFFFFFFFFFFFFFFFFFFFFFFFFFFFFFFFFFFFFFFFFFFFFFFFFFFFFFFFFFFFF

@GWZHISEQ02:315:C9E6MANXX:5:2303:19438:4682

GTACCATCTGTTCTTGGCCCTGAGCCGGGGCCCAGGTGCTCGACCACAGATATCCTGTTTGGCCCCTGTCCCAGTTTTGTTCAGCCTTATTCTTTAACTAAACTTCCTTGTGACTTTTGAGAACTC

+

BBBBBFFFFFFFFFFFFFFFFFFFFFFFFFFFFFFFFFFFFFFFFFFFFFFFFFFFFFFFFFFFFFFFFFFFFFFFFFFFFFFFFFFFFFFFFFFFFFFFFFFFFFFFFFFFFFFFFFFFFFFFFF

@GWZHISEQ02:315:C9E6MANXX:5:1108:14681:20551

AGTTCTCAAAAGTCACAAGGAAGTTTAGTTAAAGAATAAGGCTGAACAAAACTGGGACAGGGGCCAAACAGGATATCTGTGGTCGAGCACCTGGGCCCCGGCTCAGGGCCAAGAACAGATGGTACT

+

BBBBBFFFFFFFFFFFFFFFFFFFFFFFFFFFFFFFFFFFFFFFFFFFFFFFFFFFFFFFFFFFFFFFFFFFFFFFFFFFFFFFFFFFFFFFFFFFFFFFFFFFFFFFFFFFFFFFFFFFFFFFFF

@GWZHISEQ02:315:C9E6MANXX:5:1313:15152:39199

AGTTCTCAAAAGTCACAAGGAAGTTTAGTTAAAGAATAAGGCTGAACAAAACTGGGACAGGGGCCAAACAGGATATCTGTGGTCGAGCACCTGGGCCCCGGCTCAGGGCCAAGAACAGAGATCGGA

+

BBBBBFFFFFFFFFFFFFFFFFFFFFFBFFFFFFFFFFFFFFFFFFFFFFFFFFBBFFFFFFFFFBFFFFFFFFFFFFFFFFFFFFFFFFFFFFFFBFFFFFFFFFFFFFFFFFFBFFFFFFBFBB

@GWZHISEQ02:315:C9E6MANXX:5:2210:6867:12009

AGTTCTCAAAAGTCACAAGGAAGTTTAGTTAAAGAATAAGGCTGAACAAAACTGGGACAGGGGCCAAACAGGATATCTGTGGTCGAGCACCTGGGCCCCGGCTCAGGGCCAAGAACAGATGGTACT

+

BBBBBFFFBBFFFFFFFFFFFFFFFFFFFFFFFFFFFFFFFFFFFFFFFFFFFFFFFFFFFFFFFFFFFFFFFFFFFFFFFFFFFFFFFFFFFFFFFFFFFFFFFFFFFFFFF<FFFFFFFFFFFB

@GWZHISEQ02:315:C9E6MANXX:5:2214:19993:83030

AGTTCTCAAAAGTCACAAGGAAGTTTAGTTAAAGAATAAGGCTGAACAAAACTGGGACAGGGGCCAAACAGGATATCTGTGGTCGAGCACCTGGGCCCCGGCTCAGGGCCAAGAACAGATGGTACT

+

BBBBBFFFFFFFFFFFFFFFFFFFFFFFFFFFFFFFFFFFFFFFFFFFFFFFFFFFFFFFFFFFFFFFFFFFFFFFFFFFFFFFFFFFFFFFFFFFFFFFFFFFFFFFFFFFFFFFFFFFFFFFFF

@GWZHISEQ02:315:C9E6MANXX:5:2311:11799:57206

AGTTCTCAAAAGTCACAAGGAAGTTTAGTTAAAGAATAAGGCTGAACAAAACTGGGACAGGGGCCAAACAGGATATCTGTGGTCGAGCACCTGGGCCCCGGCTCAGGGCCAAGAACAGATGGTACT

+

BBBBBFFFFFFFFFFFFFFFFFFFFFFFFFFFFFFFFFFFFFFFFFFFFFFFFFFFFFFFFFFFFFFFFFFFFFFFFFFFFFFFFFFFFFFFFFFFFFFFFFFFFFFFFFFFFFFFFFFFFFFFFF

@GWZHISEQ02:315:C9E6MANXX:5:1313:15152:39199

CTGTTCTTGGCCCTGAGCCGGGGCCCAGGTGCTCGACCACAGATATCCTGTTTGGCCCCTGTCCCAGTTTTGTTCAGCCTTATTCTTTAACTAAACTTCCTTGTGACTTTTGAGAACTAGATCGGA

+

BBBBBFFFFFFFFFFFF<FFFBFFFFFFFFFFFFFFFFFFFFFFFFFFFFFFFFFFFFFFFFFFFFBBBFFFFFFFFFFFBBFFFFFFFFFFFBFFF/FFFFFFFFFFFFFFFFFFFFFFFFFFFF

@GWZHISEQ02:315:C9E6MANXX:5:1104:15995:79633

GTTCTCAAAAGTCACAAGGAAGTTTAGTTAAAGAATAAGGCTGAACAAAACTGGGACAGGGGCCAAACAGGATATCTGTGGTCGAGCACCTGGGCCCCGGCTCAGGGCCAAGAACAGATGGTACTC

+

BBBBBFFFFFFFFFFFFFFFFFFFFFFFFFFFFBFFFFFFFFFFFFFFFFFFFFBFFFFFFFFFFFFFFFFFFFFFFFFFFFFFFFFFFFFFFFFFFFFFFFFFFFFFFFFFFFFFFFFFFFFFFF

@GWZHISEQ02:315:C9E6MANXX:5:1114:14270:48131

GTTCTCAAAAGTCACAAGGAAGTTTAGTTAAAGAATAAGGCTGAACAAAACTGGGACAGGGGCCAAACAGGATATCTGTGGTCGAGCACCTGGGCCCCGGCTCAGGGCCAAGAACAGATGGTACTC

+

BBBBBFFFFFFFFFFFFFFFFFFFFFFFFFFFFFFFFFFFFFFFFFFFFFFFFFFFFFFFFFFFFFBBFFFFFFFFFFFFFFFFFFFFFFFFFFFFBFFFBFFFFFFFFFFFFFFFFBB<FFFFFF

@GWZHISEQ02:315:C9E6MANXX:5:1204:18825:89239

GTTCTCAAAAGTCACAAGGAAGTTTAGTTAAAGAATAAGGCTGAACAAAACTGGGACAGGGGCCAAACAGGATATCTGTGGTCGAGCACCTGGGCCCCGGCTCAGGGCCAAGAACAGATGGTACTC

+

BBBBBFFFFFFFFFFFFFFFFFFFFFFFFFFFFFFFFFFFFFFFFFFFFFFFFFFFFFFFFFFFFFFFFFFFFFFFFFFFFFFFFFFFFFFFFFFFFFFFFFFFFFFFFFFFFFFFFFFFFFFFFF

@GWZHISEQ02:315:C9E6MANXX:5:1213:4154:55352

GTTCTCAAAAGTCACAAGGAAGTTTAGTTAAAGAATAAGGCTGAACAAAACTGGGACAGGGGCCAAACAGGATATCTGTGGTCGAGCACCTGGGCCCCGGCTCAGGGCCAAGAACAGATGGTACTC

+

BBBBBFFFFFFFFFFFFFFFFFFFFFFFFFFFFFFFFFFFFFFFFFFFFFFFFFFFFFFFFFFFFFFFFFFFFFFFFFFFFFFFFFFFFFFFFFFFFFFFFFFBFFFFFFFFFFFFFFFFFFFFFF

@GWZHISEQ02:315:C9E6MANXX:5:1304:18666:17895

GTTCTCAAAAGTCACAAGGAAGTTTAGTTAAAGAATAAGGCTGAACAAAACTGGGACAGGGGCCAAACAGGATATCTGTGGTCGAGCACCTGGGCCCCGGCTCAGGGCCAAGAACAGATAGATCGG

+

BBBBBFFFFFFFFFFFFFFFFFFFFFFFFFFFFFFFFFFFFFFFFFFFFFFFFFFFFFFFFFFFFFFFFFFFFFFFFFFFFFFFFFFFFFFFFFFFFFFFFFFFFFFFFFFFFFFFFFFFFFFFFF

@GWZHISEQ02:315:C9E6MANXX:5:1307:19212:13659

GTTCTCAAAAGTCACAAGGAAGTTTAGTTAAAGAATAAGGCTGAACAAAACTGGGACAGGGGCCAAACAGGATATCTGTGGTCGAGCACCTGGGCCCCGGCTCAGGGCCAAGAACAGATGGTACTC

+

BBBBBFFFFFFFFFFFFFFFFFFFFFFFFFFFFFFFFFFFFFFFFFFFFFFFFFFFFFFFFFFFFFFFFFFFFFFFFFFFFFFFFFFFFFFFFFFFFFFFFFFFFFFFFFFFFFFFFFFFFFFFFF

@GWZHISEQ02:315:C9E6MANXX:5:1309:3196:88870

GTTCTCAAAAGTCACAAGGAAGTTTAGTTAAAGAATAAGGCTGAACAAAACTGGGACAGGGGCCAAACAGGATATCTGTGGTCGAGCACCTGGGCCCCGGCTCAGGGCCAAGAACAGATGGTACTC

+

BBBBBFFFFFFFFFFFFFFFFFFFFFFFFFFFFFFFFFFFFFFFFFFFFFFFFFFFFFFFFFFFFFFFFFFFFFFFFFFFFFFFFFFFFFFFFFFFFFFFFFFFFFFFFFFFFFFFFFFFFFFFFF

@GWZHISEQ02:315:C9E6MANXX:5:1310:13304:87546

GTTCTCAAAAGTCACAAGGAAGTTTAGTTAAAGAATAAGGCTGAACAAAACTGGGACAGGGGCCAAACAGGATATCTGTGGTCGAGCACCTGGGCCCCGGCTCAGGGCCAAGAACAGATGGTAGAT

+

BBBBBFFFFFFFFFFFFFFFFFFFFFFFFFFFFFFFFFFFFFFFFFFFFFFFFFFFFFFFFFFFFFFFFFFFFFFFFFFFFFFFFFFFFFFFFFFFFFFFFFFFFFFFFFFFFFFFFFFFFFFFFF

@GWZHISEQ02:315:C9E6MANXX:5:1314:4506:66792

GTTCTCAAAAGTCACAAGGAAGTTTAGTTAAAGAATAAGGCTGAACAAAACTGGGACAGGGGCCAAACAGGATATCTGTGGTCGAGCACCTGGGCCCCGGCTCAGGGCCAAGAACAGATGGTACTC

+

BBBBBFFFFFFFFFFFFFFFFFFFFFFFFFFFFFFFFFFFFFFFFFFFFFFFFFFFFFFFFFFFFFFFFFFFFFFFFFFFFFFFFFFFFFFFFFFFFFFFFBFBF<FBFFFFFFFFFFFFFFFFFF

@GWZHISEQ02:315:C9E6MANXX:5:2113:7415:68006

GTTCTCAAAAGTCACAAGGAAGTTTAGTTAAAGAATAAGGCTGAACAAAACTGGGACAGGGGCCAAACAGGATATCTGTGGTCGAGCACCTGGGCCCCGGCTCAGGGCCAAGAACAGATGGTACTC

+

BBBBBFFFFFFFFFFFFFFFFFFFFFFFFFFFFFFFFFFFFFFFFFFFFFFFFFFFFFFFFFFFFFFFFFFFFFFFFFFFFFFFBFFFFFFFFFFFFFFFFFFFFFFFFFFFFFFFFFFFFFFFFF

@GWZHISEQ02:315:C9E6MANXX:5:2205:15828:46876

GTTCTCAAAAGTCACAAGGAAGTTTAGTTAAAGAATAAGGCTGAACAAAACTGGGACAGGGGCCAAACAGGATATCTGTGGTCGAGCACCTGGGCCCCGGCTCAGGGCCAAGAACAGATGGTACTC

+

BBBBBFFFFFFFFFFFFFFFFFFFFFFFFFFFFFFFFFFFFFFFFFFFFFFFFFFFFFFFFFFFFFFFFFFFFFFFFFFFFFFFBFFFFFFFFFFFFFFFFBFFFFFFFFFFFFFFFFFFFFFFFF

@GWZHISEQ02:315:C9E6MANXX:5:2306:15614:22511

GTTCTCAAAAGTCACAAGGAAGTTTAGTTAAAGAATAAGGCTGAACAAAACTGGGACAGGGGCCAAACAGGATATCTGTGGTCAAGCACCTGGGCCCCGGCTCAGGGCCAAGAACAGATGGTACTC

+

<BBB<BB//<<FBB</<FFFFF<BF<F/F<///<BFF/</FFFFFFBFBBFFFF<FB///BFF<B/<//<//FBF//<//<FFFFFFFFFBFF//<FFFF<FF<<BFF<FFFFFBFBF<BFFBF/7

@GWZHISEQ02:315:C9E6MANXX:5:2308:15881:88285

GTTCTCAAAAGTCACAAGGAAGTTTAGTTAAAGAATAAGGCTGAACAAAACTGGGACAGGGGCCAAACAGGATATCTGTGGTCGAGCACCTGGGCCCCGGCTCAGGGCCAAGAACAGATGGTACTC

+

BBBBBFFFFFFFFFFFFFFFFFFFFFFFFFFFFFFFFFFFFFFFFFFFFFFFFFFFFFFFFFFFFFFFFFFBFFFFFFFFFFFFFFFFFFFFFFFFFFFFFFFFFFFFFFFFFFFFFFFFFFFFFF

@GWZHISEQ02:315:C9E6MANXX:5:1304:18666:17895

ATCTGTTCTTGGCCCTGAGCCGGGGCCCAGGTGCTCGACCACAGATATCCTGTTTGGCCCCTGTCCCAGTTTTGTTCAGCCTTATTCTTTAACTAAACTTCCTTGTGACTTTTGAGAACAGATCGG

+

BBBBBFFFFFFFFFFFFFFFFFFFFFFFFFFFFFFFFFFFFFFFFFFFFFFFFFFFFFFFFFFFFFFFFFFFFFFFFFFFFFFFFFFFFFFFFFFFFFFFFFFFBFFFBBFFF/7FFFFF<FFFFF

@GWZHISEQ02:315:C9E6MANXX:5:2311:9546:57512

CTGTTCTTGGCCCTGAGCCGGGGCCCAGGTGCTCGACCACAGATATCCTGTTTGGCCCCTGTCCCAGTTTTGTTCAGCCTTATTCTTTAACTAAACTTCCTTGTGACTTTTGAGAACAGATCGGAA

+

BBBBBFFFFFFFFFFFFFFFFFFFFFFFFFFFFFFFFFFFFFFFFFFFFFFFFFFFFFFFFFFFFFFFFFFFFFFFFFFFB<FFFFFFFFFFFFFFFFFFFFFFFFFFFFFFFFFFFFFFFFFFFF

@GWZHISEQ02:315:C9E6MANXX:5:2311:15540:82160

GAGTACCATCTGTTCTTGGCCCTGAGCCGGGGCCCAGGTGCTCGACCACAGATATCCTGTTTGGCCCCTGTCCCAGTTTTGTTCAGCCTTATTCTTTAACTAAACTTCCTTGTGACTTTTGAGAAC

+

BBBBBFFFF/FFFFFFFB/BFBFFFFFFFFFFFFFF<<FFFFFBBF/7FFB</FB//FFFFFFF/FFFFBFFFFBF<F<F//777FB<BFFFFFFFFFFFFFFFFFF/BFFFFFFFFFFFB/7FF<

@GWZHISEQ02:315:C9E6MANXX:5:2215:8666:55453

TGAGTACCATCTGTTCTTGGCCCTGAGCCGGGGCCCAGGTGCTTGACCACAGATATCCTGTTTGGCCCCTGTCCCAGTTTTGTTCAGCCTTATTCTTTAACTAAACTTCCTTGTGACTTTTGAGAA

+

BBBBBBFFFFFFFFFFFFFFFFFFFFFFFFFFFFFFFFFFFFFFFFFFFFBFFFFFFFFFFFFFFFFFFFFFFFFFFFFFFFFFFFFFFFFFFFFFFFFFFFFFFFFFFFFFFFFFFFFFFBFFFF

@GWZHISEQ02:315:C9E6MANXX:5:2305:6679:96688

TGAGTACCATCTGTTCTTGGCCCTGAGCCGGGGCCCAGGTGCTCGACCACAGATATCCTGTTTGGCCCCTGTCCCAGTTTTGTTCAGCCTTATTCTTTAACTAAACTTCCTTGTGACTTTTGAGAA

+

BBBBBFFFFFFFFFFFFFFFFFFFFFFFFFFFFFFFFFFFFFFFFFFFFFFFFFFFFFFFFFFFBFFFFFFFFFFFFFFFFFFFFFFFFFFFFFFFFFFFFFFFFFFFFFFFFFFFFFFFFFFFF<

@GWZHISEQ02:315:C9E6MANXX:5:1112:16460:5750

TCTCAAAAGTCACAAGGAAGTTTAGTTAAAGAATAAGGCTGAACAAAACTGGGACAGGGGCCAAACAGGATATCTGTGGTCGAGCACCTGGGCCCCGGCTCAGGGCCAAGAACAGATGGTACTCAG

+

BBBBBFFFFFFFFFFFFFFFFFFFFFFFFFFFFFFFFFFFFFFFFFFFFFFFFFFFFFFFFFFFFFFFFFFFFFFFFFFFFFFFFFFFFFFFFFFFFFFFFFFFFFFFFFFFFFFFFFFFFFFFFF

@GWZHISEQ02:315:C9E6MANXX:5:2202:13219:36832

TCTCAAAAGTCACAAGGAAGTTTAGTTAAAGAATAAGGCTGAACAAAACTGGGACAGGGGCCAAACAGGATATCTGTGGTCGAGCACCTGGGCCCCGGCTCAGGGCCAAGAACAGATGGTACTCAG

+

BBBBBFFFFFFFFFFFFFFFFFFFFFFFFFFFFFFFFFFFFFFFFFFFFFFFFFFFFFFFFFFFFFFFFFFFFFFFFFFFFFFFFFFFFFFFFFFFFFFBFFFFFFFFFFFFFFFFFFFFFFFFFF

@GWZHISEQ02:315:C9E6MANXX:5:1114:12731:93627

CATCTGTTCTTGGCCCTGAGCCGGGGCCCAGGTGCTCGACCACAGATATCCTGTTTGGCCCCTGTCCCAGTTTTGTTCAGCCTTATTCTTTAACTAAACTTCCTTGTGACTTTTGAGAGATCGGAA

+

BBBBBFFFFFFFFFFFFFFFFFFFFFFFFFFFFFFFFFFFFFFFFFFFFFFFFFFFFFFFFFFFFFFFFFFFFFFFFFFFFFFFFFFFFFFFFFFFFFFFFFFFFFFFFFFFFFFFFFFFFFFFFF

@GWZHISEQ02:315:C9E6MANXX:5:1314:9836:26902

CTGAGTACCATCTGTTCTTGGCCCTGAGCCGGGGCCCAGGTGCTCGACCACAGATATCCTGTTTGGCCCCTGTCCCAGTTTTGTTCAGCCTTATTCTTTAACTAAACTTCCTTGTGACTTTTGAGA

+

BBBBBFFFFFFFFFFFFFFFFFFFFFFFFFFFFFFFFFFFFFFFFFFFFFFFFFFFFFFFFFFFFFFFFFFFFFFFFFFFFFFFFFFFFFFFFFFFFFFFFFFFFFFFFFFFFFFFFFFFFFFFFF

@GWZHISEQ02:315:C9E6MANXX:5:2113:14360:75009

CTGAGTACCATCTGTTCTTGGCCCTGAGCCGGGGCCCAGGTGCTCGACCACAGATATCCTGTTTGGCCCCTGTCCCAGTTTTGTTCAGCCTTATTCTTTAACTAAACTTCCTTGTGACTTTTGAGA

+

BBBBBFFFFFFFFFFFFFFFFFFFFFFFFFFFFFFFFFFFFFFFFFFFFFFFFFFFFFFFFFFFFFFFFFFFFFFFFFFFFFFFFFFFFFFFFFFFFFFFFFFFFFFFFFFFFFFFFFFFFF/BFF

@GWZHISEQ02:315:C9E6MANXX:5:2204:19527:15805

CTGAGTACCATCTGTTCTTGGCCCTGAGCCGGGGCCCAAGTGCTCGACCACAGATATCCTGTTTGGCCCCTGTCCCAGTTTTGTTCAGCCTTATTCTTTAACTAAACTTCCTTGTGACTTTTGAGA

+

BBBBBFFFFFFFFFFFFFFFFFFFFFFFFFFFFFFFFFFFFFFFFFFFFFFFFFFFFFFFFFFFFFFFFFFFFFFFFFFFFFFFFFFFFFFFFFFFFFFFFFFFFFFFFFFFFFFFFFFFFFFFFB

@GWZHISEQ02:315:C9E6MANXX:5:2207:3378:3589

CTGAGTACCATCTGTTCTTGGCCCTGAGCCGGGGCCCAGGTGCTCGACCACAGATATCCTGTTTGGCCCCTGTCCCAGTTTTGTTCAGCCTTATTCTTTAACTAAACTTCCTTGTGACTTTTGAGA

+

BBBBBFFFFFFFFFFFFFFFFFFFFFFFFFFFFFFFFFFFFFFFFFFFFFFFFFFFFFFFFFFFFFFFFFFFFFFFFFFFFFFFFFFFFFFFFFFFFFFFFFFFFFFFFFFFFFFFFFFFFFFFFF

@GWZHISEQ02:315:C9E6MANXX:5:2212:3661:72873

CTGAGTACCATCTGTTCTTGGCCCTGAGCCGGGGCCCAGGTGCTCGACCACAGATATCCTGTTTGGCCCCTGTCCCAGTTTTGTTCAGCCTTATTCTTTAACTAAACTTCCTTGTGACTTTTGAGA

+

BB/BBFBFFFFFFFFFFFFF/<FBF<FFBFF<BFF77FFF<BFBFFFFBBFFFFFFFB<<FFB<FFFFBBFF<<BBFFFBFFFFFFBFBFFFFFFBB/<FBFFFFFFFFF/<FBBBFFBFFF<<B<

@GWZHISEQ02:315:C9E6MANXX:5:2304:4725:100927

ATCTGTTCTTGGCCCTGAGCCGGGGCCCAGGTGCTCGACCACAGATATCCTGTTTGGCCCCTGTCCCAGTTTTGTTCAGCCTTATTCTTTAACTAAACTTCCTTGTGACTTTTGAGATCGGAAGAG

+

BBBBBFFFFF<BFFFFFFFFFFFFFFFFFFFFFFFFFFFFFFFFFFFFFFFFFBFFFFFFFFFFFFFFFFFFFFFFFFBFFFFFFFFFFFBFFBFFFFFFFFFFFFF<FFFFFFF/<FFFFFFBFB

@GWZHISEQ02:315:C9E6MANXX:5:1107:16531:31165

CTCAAAAGTCACAAGGAAGTTTAGTTAAAGAATAAGGCTGAACAAAACTGGGACAGGGGCCAAACAGGATATCTGTGGTCGAGCACCTGGGCCCCGGCTCAGGGCCAAGAACAGATGGTACTCAGA

+

BBBBBFFFFFFFFFFFFFFFFFFFFFFFFFFFFFFFFFFFFFFFFFFFFFFFFFFFFFFFFFFFFFFFFFFFFFFFFFFFFFFFFFFFFFFFFFFFFFFFFFFFFFFFFFFFFFFFFFF/FFFFFF

@GWZHISEQ02:315:C9E6MANXX:5:1114:12731:93627

CTCAAAAGTCACAAGGAAGTTTAGTTAAAGAATAAGGCTGAACAAAACTGGGACAGGGGCCAAACAGGATATCTGTGGTCGAGCACCTGGGCCCCGGCTCAGGGCCAAGAACAGATGAGATCGGAA

+

BBBBBFFFFFFFFFFFFFFFFFFFFFFFFFFFFFFFFFFFFFFFFFFFFFFFFFFFFFFFFFFFFFFFFFFFFFFFFFFFFFFFFFFFFFFFFFFFFFFFFFFFFFFFFFFFFFFFFFFFFFFFFB

@GWZHISEQ02:315:C9E6MANXX:5:1202:9891:19398

CTCAAAAGTCACAAGGAAGTTTAGTTAAAGAATAAGGCTGAACAAAACTGGGACAGGGGCCAAACAGGATATCTGTGGTCGAGCACCTGGGCCCCGGCTCAGGGCCAAGAACAGATGGTACTCAGA

+

BBBBBFFFFFFFFFFFFFFFFFFFFFFFFFFFFFFFFFFFFFFFFFFFFFFFFFFFFFFFFFBFFFFFBFFFFFFFFFFFFBFFFFFFFFFFBFFFBFFFFFFFFFFFFFFFFFFFFFFFFFFFF<

@GWZHISEQ02:315:C9E6MANXX:5:1205:15307:7059

CTCAAAAGTCACAAGGAAGTTTAGTTAAAGAATAAGGCTGAACAAAACTGGGACAGGGGCCAAACAGGATATCTGTGGTCGAGCACCTGGGCCCCGGCTCAGGGCCAAGAACAGATGGTACTCAGA

+

BBBBBFFFFFFFFFFFFFFFFFFFFFFFFFFFFFFFFFFFFFFFFFFFFFFFFFFFFFFFFFFFFFFFFFFFFFFFFFFFFFFFFFFFFFFFFFFFFFFFFFFFFFFFFFFFFFFFFFFFFFFFF7

@GWZHISEQ02:315:C9E6MANXX:5:1205:20228:97386

CTCAAAAGTCACAAGGAAGTTTAGTTAAAGAATAAGGCTGAACAAAACTGGGACAGGGGCCAAACAGGATATCTGTGGTCGAGCACCTGGGCCCCGGCTCAGGGCCAAGAACAGATGGTACTCAGA

+

BBBBBFFFFFFFFFFFFFFFFFFFFFFFFFFFFFFFFFFFFFFFFFFFFFFFFFFFFFFFFFFFFFFFFFFFFFFFFFFFFFFFFFFFFFFFFFFFFFFFFFFFFFFFFFFFFFFFFFFFFFFFFF

@GWZHISEQ02:315:C9E6MANXX:5:1206:5190:3612

CTCAAAAGTCACAAGGAAGTTTAGTTAAAGAATAAGGCTGAACAAAACTGGGACAGGGGCCAAACAGGATATCTGTGGTCGAGCACCTGGGCCCCGGCTCAGGGCCAAGAACAGATGGTACTCAGA

+

BBBBBFFFFFFFBFFFFFFFFFFFFFFFFFFFFFFFFFFFFFFFFFFFFFFFFFFFFFFFFFFFFFFFFFFFFFFFFFFFFFFFFFFFFFFF<FFFFFFFFFFFFFBFFFFFFFFFFFFBFFFFFF

@GWZHISEQ02:315:C9E6MANXX:5:1206:2619:72349

CTCAAAAGTCACAAGGAAGTTTAGTTAAAGAATAAGGCTGAACAAAACTGGGACAGGGGCCAAACAGGATATCTGTGGTCGAGCACCTGGGCCCCGGCTCAGGGCCAAGAACAGATGGTACTCAGA

+

/BBBBBFFFFBFFFFFFFFBFFFFFFFFFFFFFFFFBFFFFFF/<FBFFFBFFFFBFBF<7F<FFFFF<BFFFFFFBFFFFBB7FFFFFFB7FF<BBF7FBFFBFFF<FFBBFFFFFFFBF<FFF<

@GWZHISEQ02:315:C9E6MANXX:5:1211:16689:18532

CTCAAAAGTCACAAGGAAGTTTAGTTAAAGAATAAGGCTGAACAAAACTGGGACAGGGGCCAAACAGGATATCTGTGGTCGAGCACCTGGGCCCCGGCTCAGGGCCAAGAGATCGGAAGAGCGTCG

+

//</BFBF<FFFFFFBFFBBFFFFFFFFFFBFFFFFFFFFFFFFFFFFFFFFFBFFFBFBFFFFFFFFFFBBBFFFFFFFFFFFFFFFFFFFFFFFFFFFFFFB<FFFBFFFFFFFFFFFFFFF</

@GWZHISEQ02:315:C9E6MANXX:5:1213:16826:10618

CTCAAAAGTCACAAGGAAGTTTAGTTAAAGAATAAGGCTGAACAAAACTGGGACAGGGGCCAAACAGGATATCTGTGGTCGAGCACCTGGGCCCCGGCTCAGGGCCAAGAACAGATGGTACTCAGA

+

BBBBB/BFFFFFFFFFFFFFFFFFFFFFFFFFFFFFFFFFFFFFFFFFFFFFFFFFFFFFFFFFFFFFFFFFFFFFFFFFFFFFFFFFFFFFFFFFFFFFFFFFFFFFFFFFFFFFFFFFFFFFFF

@GWZHISEQ02:315:C9E6MANXX:5:1304:20049:71667

CTCAAAAGTCACAAGGAAGTTTAGTTAAAGAATAAGGCTGAACAAAACTGGGACAGGGGCCAAACAGGATATCTGTGGTCGAGCACCTGGGCCCCGGCTCAGGGCCAAGAACAGATGGTACTCAGA

+

BBBBBFFFFFFFFFFFFFFFFFFFFFFFFFFFFFFFFFFFFFFFFFFFFFFFFFFFFBFFFFFFFFFFFFFFFFFFFFFFFFFFFFFFFFFFFFFFFFFFFFFFFFFFFFFFFFFFFFFFFFFFFF

@GWZHISEQ02:315:C9E6MANXX:5:1305:18895:56453

CTCAAAAGTCACAAGGAAGTTTAGTTAAAGAATAAGGCTGAACAAAACTGGGACAGGGGCCAAACAGGATATCTGTGGTCGAGCACCTGGGCCCCGGCTCAGGGCCAAGAACAGATGGTACTCAGA

+

BBBBBFFFFFFFFFFFFFFFFFFFFFFFFFFFFFFFFFFFFFFFFFFFFFFFFFFFFFFFFFFFFFFFFFFFFFFFFBFFFFFFFFFFFFFFFFFFFFFFFFFFFFFBFFFFFFFFFFFFFFFFFF

@GWZHISEQ02:315:C9E6MANXX:5:1313:17226:19993

CTCAAAAGTCACAAGGAAGTTTAGTTAAAGAATAAGGCTGAACAAAACTGGGACAGGGGCCAAACAGGATATCTGTGGTCGAGCACCTGGGCCCCGGCTCAGGGCCAAGAACAGATGGTACTCAGA

+

BBBBBFFFFFFFFFFFFFFFFFFFFFFFFFFFFFFFFFFFFFFFFFFFFFFFFFFFFFFFFFFFFFFFFFFFFFFFFFFFFFFFFFFFFFFFFFFFFFFFFFFFFFFFFFFFFFFFFFFFFFFFFF

@GWZHISEQ02:315:C9E6MANXX:5:2108:7432:95949

CTCAAAAGTCACAAGGAAGTTTAGTTAAAGAATAAGGCTGAACAAAACTGGGACAGGGGCCAAACAGGATATCTGTGGTCGAGCACCTGGGCCCCGGCTCAGGGCCAAGAACAGATGGTACTCAGA

+

BBBBBFFFFFFFFFFFFFFFFFFFFFFFFFFFFFFFFFFFFFFFFFFFFFFFFFFFFFFFFFFFFFFFFFFFFFFFFFFFFFFFFFFFFFFFFFFFFFFFFFFFFFFFFFFFFFFFFFFFFFFFFF

@GWZHISEQ02:315:C9E6MANXX:5:2110:18008:38538

CTCAAAAGTCACAAGGAAGTTTAGTTAAAGAATAAGGCTGAACAAAACTGGGACAGGGGCCAAACAGGATATCTGTGGTCGAGCACCTGGGCCCCGGCTCAGGGCCAAGAACAGATGGTACTCAGA

+

BBB/<//BF<F<FFFFFFFFFF<FBB/B<F<BFFFFFFFFFFFFFFFFFFFFFFFFFBBBBFFFFBFBBFFFBF/FFFB/<F/BFFFFFFBB7FFFFFFFFF</BFFFFFFFFFBFFFFF<F7/BF

@GWZHISEQ02:315:C9E6MANXX:5:2112:9119:60585

CTCAAAAGTCACAAGGAAGTTTAGTTAAAGAATAAGGCTGAACAAAACTGGGACAGGGGCCAAACAGGATATCTGTGGTCGAGCACCTGGGCCCCGGCTCAGGGCCAAGAACAGATGGTACTCAGA

+

BBBBBFFFFFFFFFFFFFFFFFFFFFFFFFFFFFFFFFFFFFFFFFFFFFFFFFFFFFFFFFFFFFFFFFFFFFFFFFFFFFFFFFFFFFFFFFFFFFFFFFFFFFFFFFFFFFFFFFFFFFFF<7

@GWZHISEQ02:315:C9E6MANXX:5:2112:1925:87577

CTCAAAAGTCACAAGGAAGTTTAGTTAAAGAATAAGGCTGAACAAAACTGGGACAGGGGCCAAACAGGATATCTGTGGTCGAGCACCTGGGCCCCGGCTCAGGGCCAAGAACAGATGGTACTCAGA

+

BBB/BFFFFFBFBFFFBFFFFFFFFFFFFFBFFFFFFFFFFFFFFFFFFFFFBFFFFFBFF/BFFFFFBFFFFBFFFBF/7FBFFFFFFFF/FFFFFBFFFFFB<///FFFFFBFFFFFFFFF/7B

@GWZHISEQ02:315:C9E6MANXX:5:2115:19519:25736

CTCAAAAGTCACAAGGAAGTTTAGTTAAAGAATAAGGCTGAACAAAACTGGGACAGGGGCCAAACAGGATATCTGTGGTCGAGCACCTGGGCCCCGGCTCAGGGCCAAGAACAGATGGTACTCAGA

+

BBBBBFFFFFFFFFFFFFFFFFFFFFFFFFFFFFFFFFFFFFFFFFFFFFFFFFFFFFFFFFFFFFFFFFFFFFFFFFFFFFFFFFFFFFFFFFFFFFFFFFFFFFFFFFFFFFFFFFBFFFFFFB

@GWZHISEQ02:315:C9E6MANXX:5:2116:5561:44151

CTCAAAAGTCACAAGGAAGTTTAGTTAAAGAATAAGGCTGAACAAAACTGGGACAGGGGCCAAACAGGATATCTGTGGTCGAGCACCTGGGCCCCGGCTCAGGGCCAAGAACAGATGGTACTCAGA

+

BBBBBFFFFFFFFFFFFFFFFFFFFFFFFFFFFFFFFFFFFFFFFFFFFFFFFFFFFFFFFFFFBBBBFF</FBBF/FFFFFFFFB/FFFFB</<B<///<</<7/7BBBFB77BFBBB/FFFBB7

@GWZHISEQ02:315:C9E6MANXX:5:2203:4944:21266

CTCAAAAGTCACAAGGAAGTTTAGTTAAAGAATAAGGCTGAACAAAACTGGGACAGGGGCCAAACAGGATATCTGTGGTCGAGCACCTGGGCCCCGGCTCAGGGCCAAGAACAGATGGTACTCAGA

+

BBBBBFFFFFFFFFFFFFFFFFFFFFFFFFFFFFFFFFFFFFFFFFFFFFFFFFFFFFFFFFFFFFFFFFFFFFFFFFFFFFFFFFFFFFFFFFFFFFFFFFFFFFFFFFFFFFFFFFFFFFFFF<

@GWZHISEQ02:315:C9E6MANXX:5:2209:17763:28545

CTCAAAAGTCACAAGGAAGTTTAGTTAAAGAATAAGGCTGAACAAAACTGGGACAGGGGCCAAACAGGATATCTGTGGTCGAGCACCTGGGCCCCGGCTCAGGGCCAAGAACAGATGGTACTCAGA

+

BBBBBFFFFFFFFFFFFFFFFFFFFFFFFFFFFFFFFFFFFFFFFFFFFFFFFFFFFFFFFFFFFFFFBFFFFFFFFFFFBFFFFFFFFFFFFFFFFFFFFFFFFFFFBFFFFFFFFFFFFFFFFF

@GWZHISEQ02:315:C9E6MANXX:5:2211:15640:16372

CTCAAAAGTCACAAGGAAGTTTAGTTAAAGAATAAGGCTGAACAAAACTGGGACAGGGGCCAAACAGGATATCTGTGGTCGAGCACCTGGGCCCCGGCTCAGGGCCAAGAACAGATGGTACTCAGA

+

BBBBBFFFFFFFFFFFFFFFFFFFFFFFFFFFFFFFBFFFFFFFFFFFFFFFFFFFFFFFFFFFFFFFFFFFFFFFFFFFFFFFFFFFFFFFFFFFFFFFFFFFFFFFFFFFFFFFFFFFFFFFFB

@GWZHISEQ02:315:C9E6MANXX:5:2215:6475:79526

CTCAAAAGTCACAAGGAAGTTTAGTTAAAGAATAAGGCTGAACAAAACTGGGACAGGGGCCAAACAGGATATCTGTGGTCGAGCACCTGGGCCCCGGCTCAGGGCCAAGAACAGATGGTACTCAGA

+

BBBBBFFFFFFFFFFFFFFFFFFFFFFFFFFFFFFFFFFFFFFFFFFFFFFFFFFFFFFFFFFFFFFFFFFFFFFFFFFFFFFFFFFFFFFFFFFFFFFFFFFFFFFFFFFFFFFFFFFFFFFFFF

@GWZHISEQ02:315:C9E6MANXX:5:2307:14537:37655

CTCAAAAGTCACAAGGAAGTTTAGTTAAAGAATAAGGCTGAACAAAACTGGGACAGGGGCCAAACAGGATATCTGTGGTCGAGCACCTGGGCCCCGGCTCAGGGCCAAGAACAGATGGTACTCAGA

+

<B/</B//<BF<FFFF/<F//F/BFFFF/FFF</F<<BFFBFFB/BFB</B/FFF//7F7F<BF/</B/<<FFBFFFBFFFFFFFBB/FFFF7FBFF/7BFFF/FFF<7FFBFFF<FB7/BFBFBF

@GWZHISEQ02:315:C9E6MANXX:5:2310:8706:93818

CTCAAAAGTCACAAGGAAGTTTAGTTAAAGAATAAGGCTGAACAAAACTGGGACAGGGGCCAAACAGGATATCTGTGGTCGAGCACCTGGGCCCCGGCTCAGGGCCAAGAGATCGGAAGAGCGTCG

+

BBBBBFFFFFFFFFFFFFFFFFFFFFFFFFFFFFFFFFFFFFFFFFFFFFFFFFFFFFFFFFFFFFFFFFFFFFFFFFFFFFFFFFFFFFFFFFFFFFFFFFFFFFFFFFFFFFFFFFFBFFFBFF

@GWZHISEQ02:315:C9E6MANXX:5:2311:5551:45095

CTCAAAAGTCACAAGGAAGTTTAGTTAAAGAATAAGGCTGAACAAAACTGGGACAGGGGCCAAACAGGATATCTGTGGTCGAGCACCTGGGCCCCGGCTCAGGGCCAAGAACAGAGATCGGAAGAG

+

BBBBBFFFBFFFFFFFFFBFFFBBFFFFFFFFFFFBFBF<FFFFFFFFFFFFFFFFBFFBFFF<FFFFBFFFFFFF//FFFBBFFBFFFFFFFFFFFF/BF/FFFFFFFFFFFBB<7FFF<BFFFF

@GWZHISEQ02:315:C9E6MANXX:5:1112:11136:23530

CTGAGTACCATCTGTTCTTGGCCCTGAGCCGGGGCCCAGGTGCTCGACCACAGATATCCTGTTTGGCCCCTGTCCCAGTTTTGTTCAGCCTTATTTCTTTAACTAAACTTCCTTGTGACTTTTGAG

+

BBBBBFFFFFFFFFFFFFFFFFBFFFBFFFFFFFFFFFFFFFFFFFFFFFFFFFFFFFFFFFFFFFFFFFFFFFFFFFBFFFFFFFFFFFFFFFFFFFFFFFFFBFFFFFFFFBFFFFFFFFFFFF

@GWZHISEQ02:315:C9E6MANXX:5:1208:16730:44962

CTGAGTACCATCTGTTCTTGGCCCTGAGCCGGGGCCCAGGTGCTCGACCACAGATATCCTGTTTGGCCCCTGTCCCAGTTTTGTTCAGCCTTATTTCTTTAACTAAACTTCCTTGTGACTTTTGAG

+

BBBBBFFFFFFFFFFFFFFFFFFFFFFFFFFFFFFFFFFFFFFFFFFFFFFBFFFFFFFFFFFFFFFFFFFFFFFFFFFFFFFFFFFFFFFFFFFFFFFFFFFFFFFFFFFFFFFFFFFFFFFFFF

@GWZHISEQ02:315:C9E6MANXX:5:1313:11541:35231

TCAAAAGTCACAAGGAAGTTTAGTTAAAGAATAAGGCTGAACAAAACTGGGACAGGGGCCAAACAGGATATCTGTGGTCGAGCACCTGGGCCCCGGCTCAGGGCCAAGAACAGATGGTACTCAGAT

+

BBBBBFFFFFFFFFFFFFFFFFFFFFFFFFBFFFFFFFFFFFFFFFFFFFFFFFFFFFFFFFFFFFFFFFFFFFFFFFFFFFFFFFFFFFFFFFFFFBFFFFFFFFFFFFFFFFFFFFFBFFFFFF

@GWZHISEQ02:315:C9E6MANXX:5:2104:16704:89631

TCAAAAGTCACAAGGAAGTTTAGTTAAAGAATAAGGCTGAACAAAACTGGGACAGGGGCCAAACAGGATATCTGTGGTCGAGCACCTGGGCCCCGGCTCAGGGCCAAGAACAGATGGTACTCAGAT

+

BBBBBFFFFFFFFFFFFFFFFFFFFFFFFFFFFFFFFFFFFFFFFFFFFFFFFFFFFFFFFFFFFFFFFFFFFFFFFFFFFFFFFFFFFFFFFFFFFFFFFFFFFFFFFFFFFFFFFFFFFFFFFF

@GWZHISEQ02:315:C9E6MANXX:5:2309:7114:22801

TCAAAAGTCACAAGGAAGTTTAGTTAAAGAATAAGGCTGAACAAAACTGGGACAGGGGCCAAACAGGATATCTGTGGTCGAGCACCTGGGCCCCGGCTCAGGGCCAAGAGATCGGAAGAGCGTCGT

+

BBBBBFFFFFFFFFFFFFFFFFFFFFFFFFFFFFFFFFFFFFFFFFFFFFFFFFFFFFFFFFFFFFFFFFFFFFFFFFFFFFFFFFFFFFFFFFFFFFFFFFFFFFFFFFFFFFFFFFFFFF7<B/

@GWZHISEQ02:315:C9E6MANXX:5:1105:5382:11628

CAAAAGTCACAAGGAAGTTTAGTTAAAGAATAAGGCTGAACAAAACTGGGACAGGGGCCAAACAGGATATCTGTGGTCGAGCACCTGGGCCCCGGCTCAGGGCCAAGAACAGATGGTACTCAGATA

+

BBBBBFFFFFFFFFFFFFFFFFFFFFFFFFFFFFFFFFFFFFFFFFFFFFFFFFFFFFFFFFFFFFFFFFFFFFFFFFFFFFFFFFFFFFFFFFFFFFFFFFFFFFFFFFFFFFFFFFFFFFFFFF

@GWZHISEQ02:315:C9E6MANXX:5:1116:15217:4051

CAAAAGTCACAAGGAAGTTTAGTTAAAGAATAAGGCTGAACAAAACTGGGACAGGGGCCAAACAGGATATCTGTGGTCGAGCACCTGGGCCCCGGCTCAGGGCCAAGAACAGATGGTACTCAGATA

+

BBBBBFFFFFFFFFFFFFFFFFFFFFFFFFFFFFFFFFFFFFFFFFFFFFFFFFFFBFFFFFFFFFFFFFFBFFFFFFFFFFFFFFFFFFFFFFFFFFFFFFBFFFFFFFFFBFFFF<FFFFFFFF

@GWZHISEQ02:315:C9E6MANXX:5:1314:20001:54065

CAAAAGTCACAAGGAAGTTTAGTTAAAGAATAAGGCTGAACAAAACTGGGACAGGGGCCAAACAGGATATCTGTGGTCGAGCACCTGGGCCCCGGCTCAGGGCCAAGAACAGATGGTACTCAGATA

+

BBBBBF/BFFFF<FFBFFFFFF<<F<<<FFFFFFFF<FFFFFFFFFFFFFF<FFFFB<B<FF/FFFFF//<</<FBF<7BFFFFF<<<BFF/<<BB//7FFB/BF/FBFFFFFF7B7F/77/F7BF

@GWZHISEQ02:315:C9E6MANXX:5:2101:15922:42710

CAAAAGTCACAAGGAAGTTTAGTTAAAGAATAAGGCTGAACAAAACTGGGACAGGGGCCAAACAGGATATCTGTGGTCGAGCACCTGGGCCCCGGCTCAGGGCCAAGAACAGATGGTACTCAGATA

+

BBBBBFFFFFFFFFFFFFFFFFFFFFFFFFFFFFFFFFFFFFFFFFFFFFFFFFFFFFFFFFFFFFFFFFFFFFFFFFFFFFFFFFFFFFFFFFFFFFFFFFFFFFFFFFFFFFFFFFFFFFFFFF

@GWZHISEQ02:315:C9E6MANXX:5:2105:7771:13053

CAAAAGTCACAAGGAAGTTTAGTTAAAGAATAAGGCTGAACAAAACTGGGACAGGGGCCAAACAGGATATCTGTGGTCGAGCACCTGGGCCCCGGCTCAGGGCCAAGAACAGATGGTACTCAGATA

+

BBBBBFFFFFFFFFFFFFFFFFFFFFFFFFFFFFFFFFFFFFFFFFFFFFFFFFFFFFFFFFFFFFFFFFFFFFFFFFFFFFFFFFFFFFFFFFFFFFFFFFFFFFFFFFFFFFFFFFFFFFFFFF

@GWZHISEQ02:315:C9E6MANXX:5:2106:3979:39088

CAAAAGTCACAAGGAAGTTTAGTTAAAGAATAAGGCTGAACAAAACTGGGACAGGGGCCAAACAGGATATCTGTGGTCGAGCACCTGGGCCCCGGCTCAGGGCCAAGAACAGATGGTACTCAGATA

+

BBBBBFFFFFFFFFFFFFFFFFFFFFFFFFFFFFFFFFFFFFFFFFFFFFFFFFFFFFFFFFFFFFFFFFFFFFFFFFFFFFFFFFFFFFFFFFFFFFFFFFFFFFFFFFFFFFFBFFFFFFFFFF

@GWZHISEQ02:315:C9E6MANXX:5:2207:16891:47313

CAAAAGTCACAAGGAAGTTTAGTTAAAGAATAAGGCTGAACAAAACTGGGACAGGGGCCAAACAGGATATCTGTGGTCGAGCACCTGGGCCCCGGCTCAGGGCCAAGAACAGATGGTACTCAGATA

+

BBBBBFFFFFFFFFFFFFFFFFFFFFFFFFFFFFFFFFFFFFFFFFFFFFFFFFFFFFFFFFFFFFFFFFFFFFFFFFFFFFFFFFFFFFFFFFFFFFFFFFFFFFFFFFFFFFFFFFFFFFFFFF

@GWZHISEQ02:315:C9E6MANXX:5:2208:19567:92842

CAAAAGTCACAAGGAAGTTTAGTTAAAGAATAAGGCTGAACAAAACTGGGACAGGGGCCAAACAGGATATCTGTGGTCGAGCACCTGGGCCCCGGCTCAGGGCCAAGAACAGATGGTACTCAGATA

+

BBBBBFFFFFFFFFFFFFFFFFFFFFFFFFFFFFFFFFFFFFFFFFFFFFFFFFFFFFFFFFFFFFFFFFFFFFFFFFFFFFFFFFFFFFFFFFFFFFFFFFFFFFFFFFFFBFFFFFFFFFFFFF

@GWZHISEQ02:315:C9E6MANXX:5:2216:2301:31107

CAAAAGTCACAAGGAAGTTTAGTTAAAGAATAAGGCTGAACAAAACTGGGACAGGGGCCAAACAGGATATCTGTGGTCGAGCACCTGGGCCCCGGCTCAGGGCCAAGAACAGATGGTACTCAGATA

+

BBBBBFFFFFFFFFFFFFFFFFFBFFFFFFFFFFFFFFFFFFFFFFFFFFFFFFFFFFFFFFFFFFFFFFFFFFFFFFFFFFFFFFFFFFFFFFFBFFFFFFFFFFFFFFFFFFFFFBFFFBFFFF

@GWZHISEQ02:315:C9E6MANXX:5:2309:3936:4803

CAAAAGTCACAAGGAAGTTTAGTTAAAGAATAAGGCTGAACAAAACTGGGACAGGGGCCAAACAGGATATCTGTGGTCGAGCACCTGGGCCCCGGCTCAAGGCCAAGAACAGATGGTACTCAGATA

+

BBBBBFFFFFFFFFFFFFFFFFFFFFFFFFFFFFFFFFFFFFFFFFFFFFFFFFFFFFFFFFFFFFFFFFFFFFFFFFFFFFFFFFFFFFFFFFFFFFF/FFFFFFFFFFFFFFFFFFFFFFFFFF

@GWZHISEQ02:315:C9E6MANXX:5:2309:10736:89745

CAAAAGTCACAAGGAAGTTTAGTTAAAGAATAAGGCTGAACAAAACTGGGACAGGGGCCAAACAGGATATCTGTGGTCGAGCACCTGGGCCCCGGCTCAGGGCCAAGAACAGAGATCGGAAGAGCG

+

BBBBBFFFFFFFFFFBFFFFFFFFFFFFFFFFFFFFFFFFFFFFFFFFFFFFFFFFFFFFFFFFFFFFFFFFFFFFFFFFFFFFFFFFFFFFFFFFFFFFFFBFFFFFFFFFFFFFFFFFFFFFFF

@GWZHISEQ02:315:C9E6MANXX:5:2314:17221:75139

CAAAAGTCACAAGGAAGTTTAGTTAAAGAATAAGGCTGAACAAAACTGGGACAGGGGCCAAACAGGATATCTGTGGTCGAGCACCTGGGCCCCGGCTCAGGGCCAAGAACAGATGGTACTCAGATA

+

BBBBBFFFFFFFFFFFFFFFFFFFFFFFFFFFFFFFFFBFFFFFFFFFFFFFFFFFFFFFFFFFFFFFFFFFFFFFFFFFFFFFFFFFFFFFFFFBBFFFFFFFFFFFFFFFFFFFFBFBFFFFFF

@GWZHISEQ02:315:C9E6MANXX:5:1115:17522:15151

AAAAGTCACAAGGAAGTTTAGTTAAAGAATAAGGCTGAACAAAACTGGGACAGGGGCCAAACAGGATATCTGTGGTCGAGCACCTGGGCCCCGGCTCAGGGCCAAGAACAGATGGTACTCAGATAA

+

BBBBBFFFFFFFFFFFFFFFFF<FFFFFFFFFFFFFFFFFFFFFFFFFFFFFFFFFFFFFFFFFFFFFFFFFFFFFFFFFFFFFFFFFFFFFFFFFFFFFFBFFFBBFFFFBFFFFFFFFFFFFFF

@GWZHISEQ02:315:C9E6MANXX:5:1202:14849:70562

AAAAGTCACAAGGAAGTTTAGTTAAAGAATAAGGCTGAACAAAACTGGGACAGGGGCCAAACAGGATATCTGTGGTCGAGCACCTGGGCCCCGGCTCAGGGCCAAGAACAGATGGTACTCAGATAA

+

BBBBBFFFFFFFFFFFFFFFFFFFFFFFFBFFFFFFFFFFFFFFFFFFFFFFFFFFFFFFFFFFFFFFFFFFFFFFFFFFFFFFFFFFFFFFFFFFFFFFFFFFFFFFFFFFFFFFFFFFFFBFFF

@GWZHISEQ02:315:C9E6MANXX:5:2108:19842:11314

AAAAGTCACAAGGAAGTTTAGTTAAAGAATAAGGCTGAACAAAACTGGGACAGGGGCCAAACAGGATATCTGTGGTCGAGCACCTGGGCCCCGGCTCAGGGCCAAGAACAGATGGTACTCAGATAA

+

BBBBBFFFFFFFFFFFFFFFFFFFFFFFFFFFFFFFFFFFFFFFFFFFFFFFFFFFFFFFFFFFFFFFFFFFFFFFFFFFFFFFFFFFFFFFFFFFFFFFFFFFFFFFFFFFFFFFFFFFFFFFFF

@GWZHISEQ02:315:C9E6MANXX:5:2107:20444:75980

AAAGTCACAAGGAAGTTTAGTTAAAGAATAAGGCTGAACAAAACTGGGACAGGGGCCAAACAGGATATCTGTGGTCGAGCACCTGGGCCCCGGCTCAGGGCCAAGAACAGATGGTACTCAGATAAA

+

BBBBBFFFFFFFFFFFFFFFFFFFFFFFFFFFFFFFFFFFFFFFFFFFFFFFFFFFFFFFFFFFFFFFFFFFFFFFFFFFFFFFFFFFFFFFFFFFFFFFFFFFFFFFFFFFFFFFFFFFFFFFFF

@GWZHISEQ02:315:C9E6MANXX:5:1212:19636:9542

CTTTATCTGAGTACCATCTGTTCTTGGCCCTGAGCCGGGGCCCAGGTGCTCGACCACAGATATCCTGTTTGGCCCCTGTCCCAGTTTTGTTCAGCCTTATTCTTTAACTAAACTTCCTTGTGACTT

+

/B/<BFFFFFFFFFFFFFFFFFFFFFFFFFFFBFFBFBFFFFFFFFFFFFFFFFFFFFFFF<FFFFFFFFFFBFFFFFFFFFFFFFFFFFFFFFFFFFFFFFFFFBFFFFFFFFFFFFFFF7/BFF

@GWZHISEQ02:315:C9E6MANXX:5:2203:2221:46029

CTTTATCTGAGTACCATCTGTTCTTGGCCCTGAGCCGGGGCCCAGGTGCTCGACCACAGATATCCTGTTTGGCCCCTGTCCCAGTTTTGTTCAGCCTTATTCTTTAACTAAACTTCCTTGTGACTT

+

BBBBBFFFFFFFFFFFFFFFFFFFFFFFFFFFFFFFFFFFFFFFFFFFFFFFFFFFFFFFFFFFFFFFFFFFFFFFFFFFFFFFFFFFFFFFFFFFFFFFFFBFFBFFFF/FFFFFFFFFFFFFFF

@GWZHISEQ02:315:C9E6MANXX:5:2205:6467:61374

CTTTATCTGAGTACCATCTGTTCTTGGCCCTGAGCCGGGGCCCAGGTGCTCGACCACAGATATCCTGTTTGGCCCCTGTCCCAGTTTTGTTCAGCCTTATTCTTTAACTAAACTTCCTTGTGACTT

+

BBBBBFFFFFFFFFFFFFFFFFFFFFFFFFFFFFFFFFFFFFFFFFFFFFFFFFFFFFFFFFFFFFFFFFFFFFFFFFFFFFBF/FFFFFFFFFFFFFFFFFFFFFFFFFFFFFFFFFFFFFFFFF

@GWZHISEQ02:315:C9E6MANXX:5:1103:10260:18448

GCTTTATCTGAGTACCATCTGTTCTTGGCCCTGAGCCGGGGCCCAGGTGCTCGACCACAGATATCCTGTTTGGCCCCTGTCCCAGTTTTGTTCAGCCTTATTCTTTAACTAAACTTCCTTGTGACT

+

BB/BBFFFFFFFFFFFFFFFFFFFFFFFFFFFFFFFFFFFFFFFBFFFFFFFFFFFFFFFFFFFFFFFFFFFFFFFFFFFFFFFBFFFFFFBFFFFFFF7FFFFFFFFFFFFFFFFF<BFFFFFFF

@GWZHISEQ02:315:C9E6MANXX:5:1305:14473:45019

GCTTTATCTGAGTACCATCTGTTCTTGGCCCTGAGCCGGGGCCCAGGTGCTCGACCACAGATATCCTGTTTGGCCCCTGTCCCAGTTTTGTTCAGCCTTATTCTTTAACTAAACTTCCTTGTGACT

+

BBBBB<<FF/BFFFFFFFFF/<B/FFB/FBBFBFBBBFF/7/FFF/BFBFFFF<BFFF/BBFBFF//FF/FF/F/FFFBF/<FBFFBFFFFF/FFF/BF/BF7FFF7/<7BFFFFF<BFBFFF/77

@GWZHISEQ02:315:C9E6MANXX:5:2303:12448:84720

GCTTTATCTGAGTACCATCTGTTCTTGGCCCTGAGCCGGGGCCCAGGTGCTCGACCACAGATATCCTGTTTGGCCCCTGTCCCAGTTTTGTTCAGCCTTATTCTTTAACTAAACTTCCTTGTGACT

+

BBBBBFFFFFFFFFFFFFFFFFFFFFFFFFFFFFFFFFFFFFFFFFFFFFFFFFFFFFFFFFFFFFFFFFFFFFFFFFFFFFFFFFFFFFFFFFFFFFFBFFFFFFFFFFFFFFFFFFFFFFFBFF

@GWZHISEQ02:315:C9E6MANXX:5:2307:20137:57600

GTCACAAGGAAGTTTAGTTAAAGAATAAGGCTGAACAAAACTGGGACAGGGGCCAAACAGGATATCTGTGGTCGAGCACCTGGGCCCCGGCTCAGGGCCAAGAACAGATGGTACTCAGATAAAGCG

+

BBBBBFFBBFFFFFFFFFFFFFFFFFFFFFFFF<FFFFFFFFF/FFFFFFFFFFFFFFFFFFFFFFFFFFFFFF/FFFFFF<FFFFFFFFFFFFFFFFFFFBFFFFFFFFFBFFFFFFFFFFFFFF

@GWZHISEQ02:315:C9E6MANXX:5:1107:7317:4509

CGCTTTATCTGAGTACCATCTGTTCTTGGCCCTGAGCCGGGGCCCAGGTGCTCGACCACAGATATCCTGTTTGGCCCCTGTCCCAGTTTTGTTCAGCCTTATTCTTTAACTAAACTTCCTTGTGAC

+

BBBBBFFFFFFFFFFFFFFFFFFFFFFFFFFFFFFFFFFFFFFFFFFFFFFFFFFFFFFFFFFFFFFFFFFFFFFFFFFFFFFFFFFFFFFFFFFFFFFBFFFFFFFFFFFFFFFFFFFFBFFFFF

@GWZHISEQ02:315:C9E6MANXX:5:1302:20821:19955

CGCTTTATCTGAGTACCATCTGTTCTTGGCCCTGAGCCGGGGCCCAGGTGCTCGACCACAGATATCCTGTTTGGCCCCTGTCCCAGTTTTGTTCAGCCTTATTCTTTAACTAAACTTCCTTGTGAC

+

BBBBBFFFFFFFFFFFFFFFFFFFFFFFFFFFFFFFFFFFFFFFFFFFFFFFFFFFFBFFFFFFFFFFFFFFFFFFFFFFFFFFFFFFFFFFFFFFFFFFFFFFFFFFFFFFFFFFFFFFFFFFFF

@GWZHISEQ02:315:C9E6MANXX:5:1312:3225:33753

CGCTTTATCTGAGTACCATCTGTTCTTGGCCCTGAGCCGGGGCCCAGGTGCTCGACCACAGATATCCTGTTTGGCCCCTGTCCCAGTTTTGTTCAGCCTTATTCTTTAACTAAACTTCCTTGTGAC

+

<BBBBFBFBFFFFFFFFFFFFBFFFFFFFFFFFFFFFFFFFFFFFFFFFFFFFFFFFBFFFFFFFFFFFFFFFFFFFFFFFFFFFFFFFBFFFFFFFB<FFFFBFFFFFFFFFFFF/<<BFFFFBF

@GWZHISEQ02:315:C9E6MANXX:5:2202:9433:78647

CGCTTTATCTGAGTACCATCTGTTCTTGGCCCTGAGCCGGGGCCCAGGTGCTCGACCACAGATATCCTGTTTGGCCCCTGTCCCAGTTTTGTTCAGCCTTATTCTTTAACTAAACTTCCTTGTGAC

+

BBBBBFFFFFFFFFFFFFFFFFFFFFFFFFFFFFFFFFFFFFFFFFFFFFFFFFFFFFFFFFFFFFFFFFFFFFFFFFFFFFFFFFFFFFFFFFFFFFFFFFFFFFFFFFFFFFFFFFBFFFFFBF

@GWZHISEQ02:315:C9E6MANXX:5:2311:2269:70059

CGCTTTATCTGAGTACCATCTGTTCTTGGCCCTGAGCCGGGGCCCAGGTGCTCGACCACAGATATCCTGTTTGGCCCCTGTCCCAGTTTTGTTCAGCCTTATTCTTTAACTAAACTTCCTTGTGAC

+

BBBBBFFFFFFFFFFFFFFFFFFFFFFBFFFFFFFFFFFFFFFFFFFFFFFFFFFFFFFFFFFFFFFFFFFFFFFFFFFFFFFFFFFFFFFFFFFFFFFBFFFFBFFFFFFFFFFFBFFFFFFFFF

@GWZHISEQ02:315:C9E6MANXX:5:2202:7432:49833

TCACAAGGAAGTTTAGTTAAAGAATAAGGCTGAACAAAACTGGGACAGGGGCCAAACAGGATATCTGTGGTCGAGCACCTGGGCCCCGGCTCAGGGCCAAGAACAGATGGTACTCAGATAAAGCGA

+

BBBBBFFFFBFFFFFFFFFFFFFFFFFFFFFFFFFFFFFFFFFFFFFFFFFFFFFFFFFFFFFFFFFFFFFBFFFFFFFFFFFFFFFFFFFFFFFFFFFFFBFFFFB<FFFFFFFFFFFFFFFFFF

@GWZHISEQ02:315:C9E6MANXX:5:2208:19907:97882

TCACAAGGAAGTTTAGTTAAAGAATAAGGCTGAACAAAACTGGGACAGGGGCCAAACAGGATATCTGTGGTCGAGCACCTGGGCCCCGGCTCAGGGCCAAGAACAGATGGTACTCAGATAAAGCGA

+

BBBBBFFFFFFFFFFFFFFFFFFFFFFFFFFFFFFFFFFFFFFFFFFFFFFFFFFFFFFFFFFFFFFFFFFFFFFFFFFFFFFFFFFFFFFFFFFFFFFFFFFFFFFFFFFFFFFFFFFFFFFFFB

@GWZHISEQ02:315:C9E6MANXX:5:2109:14777:91063

CTGAGTACCATCTGTTCTTGGCCCTGAGCCGGGGCCCAGGTGCTCGACCACAGATATCCTGTTTGGCCCCTGTCCCAGTTTTGTTCAGCCTTATTCTTTAACTAAACTTCCTTGTGAGATCGGAAG

+

BBBBBFFFFFFFFFFFFFFFFFFFFFFFFFFFFFFFFFFFFFFFFFFFFFFFFFFFFFFFFFFFFFFFFFFFFFFFFFFFFFFFFFFFFFFFFFFFFFFFFFFFFFFFFFFFFFFFFFFFFFFFFF

@GWZHISEQ02:315:C9E6MANXX:5:1314:9496:46989

CACAAGGAAGTTTAGTTAAAGAATAAGGCTGAACAAAACTGGGACAGGGGCCAAACAGGATATCTGTGGTCGAGCACCTGGGCCCCGGCTCAGGGCCAAGAACAGATGGTACTCAGATAAAGCGAA

+

BBBBBFFFFFFFFFFFFFFFFFFFB/B/BFF/FFFFFFF/FFFFFFFFFF<FFFBFFFFFFFFFFFFBFFFFFFFFFF<//BFFFFBFFBBFFB<B/7FFFFFFFFFFFFFB<FFFFFFFFFFFBF

@GWZHISEQ02:315:C9E6MANXX:5:2112:5326:71613

CACAAGGAAGTTTAGTTAAAGAATAAGGCTGAACAAAACTGGGACAGGGGCCAAACAGGATATCTGTGGTCGAGCACCTGGGCCCCGGCTCAGGGCCAAGAACAGATGGTACTCAGATAAAGCGAA

+

BBBBBFFFFFFFFFFFFFFFFFFFFFFFFFFFFFFFFFFFFFFFFFFFFFFFFFFFFFFFFFFFFFFFFFFFFFFFFFFFFFFFFFFFFFFFFFFFFFFFFFFFFFFFFFFFFFFFFFFFFFFFFF

@GWZHISEQ02:315:C9E6MANXX:5:1108:19561:60676

CTCGCTTTATCTGAGTACCATCTGTTCTTGGCCCTGAGCCGGGGCCCAGGTGCTCGACCACAGATATCCTGTTTGGCCCCTGTCCCAGTTTTGTTCAGCCTTATTCTTTAACTAAACTTCCTTGTG

+

BBB/BFBFFFBFFFFFFFFFFFFFFFFFF<<FFFFFFFFFF/BFBFFFFFFFFBFFFFFFFFFFFFBFFFFFFFFBFFBBFFFFFFFFF<BFFFFB/FF/BFFBFBFFBBFBFFFFF<FBFFFFFB

@GWZHISEQ02:315:C9E6MANXX:5:1205:7253:25953

TTCGCTTTATCTGAGTACCATCTGTTCTTGGCCCTGAGCCGGGGCCCAGGTGCTCGACCACAGATATCCTGTTTGGCCCCTGTCCCAGTTTTGTTCAGCCTTATTCTTTAACTAAACTTCCTTGTG

+

BBBBBFFFFFFFFFFFFFFFFFFFFFFFFFFFFFFFFFFFFFFFFFFFFFFFFFFFFFFFFFFFFFFFFFFFFFFFFFFFFFFFFFFFFFFFFFFFFFFFFFFFFFFFFFFFFFFFFFFFFFFFFB

@GWZHISEQ02:315:C9E6MANXX:5:1111:12310:67731

TTTCGCTTTATCTGAGTACCATCTGTTCTTGGCCCTGAGCCGGGGCCCAGGTGCTCGACCACAGATATCCTGTTTGGCCCCTGTCCCAGTTTTGTTCAGCCTTATTCTTTAACTAAACTTCCTTGT

+

BBBBBFFFFFFFFFFFFFFFFFFFFFFFFFFFFFFFFFFFFFFFFFFFFFFFFFFF<BFFBFFFFFFFFFFFFFFFFFFFFFBFFFFFFBFFFFFFFFFFFFFFFFFFFFFFFFFFFFFFFFFFFF

@GWZHISEQ02:315:C9E6MANXX:5:1207:2498:28706

CTTCGCTTTATCTGAGTACCATCTGTTCTTGGCCCTGAGCCGGGGCCCAGGTGCTCGACCACAGATATCCTGTTTGGCCCCTGTCCCAGTTTTGTTCAGCCTTATTCTTTAACTAAACTTCCTTGT

+

BBBBBFFFFFFFFFFFFFFFFFFFFFFFFFFFFFFFFFFFFFFFFFFFFFFFFFFFFFFFFFFFFFFFFFFFFFFFFFFFFFFFFFFFFFFFFFFFFFFFFFFFFFFFFFFFFFFFFFFFFFFFBF

@GWZHISEQ02:315:C9E6MANXX:5:2107:2249:4108

TTTCGCTTTATCTGAGTACCATCTGTTCTTGGCCCTGAGCCGGGGCCCAGGTGCTCGACCACAGATATCCTGTTTGGCCCCTGTCCCAGTTTTGTTCAGCCTTATTCTTTAACTAAACTTCCTTGT

+

BBBBBFFFFFFFFFFFFFFFFFFFFFFFFFFFFFFFFFFFFFFFFFFFFFFFFFFFFFFFFFFFFFFFFFFFFFFFFFFFFFFFFFFFFFFFFFFFFFFFFFFFFFFFFFFFFFFFFFFFFFFFFF

@GWZHISEQ02:315:C9E6MANXX:5:2113:5627:43945

TTTCGCTTTATCTGAGTACCATCTGTTCTTGGCCCTGAGCCGGGGCCCAGGTGCTCGACCACAGATATCCTGTTTGGCCCCTGTCCCAGTTTTGTTCAGCCTTATTCTTTAACTAAACTTCCTTGT

+

BBBBBFFFFFFFFFFFFFFFFFFFFFFFFFFFFFFFFFFFFFFFFFFFFFFFFFFFFFFFFFFFFFFFFFFFFFFFFFFFFFFFFFFFFBFFFFFFFFFFFFFFFFFFFFFFFFFFFFFFFFFFFF

@GWZHISEQ02:315:C9E6MANXX:5:2216:2301:31107

TTTCGCTTTATCTGAGTACCATCTGTTCTTGGCCCTGAGCCGGGGCCCAGGTGCTCGACCACAGATATCCTGTTTGGCCCCTGTCCCAGTTTTGTTCAGCCTTATTCTTTAACTAAACTTCCTTGT

+

BBBBBFFFFFFFFFFFFFFFFFFFFFFFFFFFFFFFFFFFFFFFFFFFFFFFFFFFFFFFFFFFFFBFFFFFFFFFFFFFFFFFFFFFFFF/7BFBFFFFFFFFFFFFFFFFFFFFFFFFFFFFF<

@GWZHISEQ02:315:C9E6MANXX:5:1109:12719:33839

CAAGGAAGTTTAGTTAAAGAATAAGGCTGAACAAAACTGGGACAGGGGCCAAACAGGATATCTGTGGTCGAGCACCTGGGCCCCGGCTCAGGGCCAAGAACAGATGGTACTCAGATAAAGCGAAAC

+

BBBBBFFFFFFFFFFFFFFFFFFFFFBFFFFFFFFFFFBFFFFFFFFFFFFFFFFFFFFBFFFFFFBF<FFBBFFFFBBBFBBFFBB<FF/7FFFFFFFFFFFFF/BBFFFFFFFFFFFFFFFBBB

@GWZHISEQ02:315:C9E6MANXX:5:1316:14730:71747

CAAGGAAGTTTAGTTAAAGAATAAGGCTGAACAAAACTGGGACAGGGGCCAAACAGGATATCTGTGGTCGAGCACCTGGGCCCCGGCTCAGGGCCAAGAACAGATGGTACTCAGATAAAGCGAAAC

+

/////BBFFFFF/<FBB/B/<</<F</F/<FFF/FFFFF//FFFFFFFFBFFF//FBFFFBFFFFBFFFFFBF//</</BFBFFFFFFFF</<F//<FFFBB<FBBFFFFFF/77/<7BFBBBFF/

@GWZHISEQ02:315:C9E6MANXX:5:1109:10397:23013

GTTTCGCTTTATCTGAGTACCATCTGTTCTTGGCCCTGAGCCGGGGCCCAGGTGCTCGACCACAGATATCCTGTTTGGCCCCTGTCCCAGTTTTGTTCAGCCTTATTCTTTAACTAAACTTCCTTG

+

<BBBBFFFFFFFFFFFFFFFFFFFFFFFFFFFFFFFFFFFFFFFFFFFFFFFFFFFFFFFFFFFFFFFFFFFFFFFFFFFFFFFFFFFFFFFFFFFFFFFFFFFFFFFFFFFFFFFFFFFFFFFFF

@GWZHISEQ02:315:C9E6MANXX:5:2310:4398:94265

GTTTCGCTTTATCTGAGTACCATCTGTTCTTGGCCCTGAGCCGGGGCCCAGGTGCTCGACCACAGATATCCTGTTTGGCCCCTGTCCCAGTTTTGTTCAGCCTTATTCTTTAACTAAACTTCCTTG

+

BBBBBFFFFFFFFFFFFFFFFFFFFFFFFFFFFFFFFBFFFFF<FFFFFFFFFFFFFBFFFFFFFFFFF<FBFFFFFBFFFFFFFFFFFFFFFFFBFFFFFFFFFFFFFFFFFFFFFFFFFFFFFB

@GWZHISEQ02:315:C9E6MANXX:5:1308:15870:37104

AAGGAAGTTTAGTTAAAGAATAAGGCTGAACAAAACTGGGACAGGGGCCAAACAGGATATCTGTGGTCGAGCACCTGGGCCCCGGCTCAGGGCCAAGAACAGATGGTACTCAGATAAAGCGAAACT

+

BBBBBFFFFFFFFFFFFFFFFFFFFFFFFFFFFFFFFFFFFFFFFFFFFFFFFFFFFFFFFFFFFFFFFFFFFFFFFFFFFFFFFFFFFFFFFFFFFFFFFFFFFFFFFFFFFFFFFFFFFFFBFB

@GWZHISEQ02:315:C9E6MANXX:5:2112:3563:23570

CTGTTCTTGGCCCTGAGCCGGGGCCCAGGTGCTCGACCACAGATATCCTGTTTGGCCCCTGTCCCAGTTTTGTTCAGCCTTATTCTTTAACTAAACTTCCTTAGATCGGAAGAGCACACGTCTGAA

+

BBBBBB<<FFFFFFFFFFFFFFFFFFFFFFFFFFFFFFFFFFFFFFFFFFFFFFBFFFFBFFFFFFFF<FBFFFFFFFFFFFFFFFFFFFFFFFFFFFFFFFFFFFFFFFFFFFFFFFFFFFFF/B

@GWZHISEQ02:315:C9E6MANXX:5:2116:14499:27774

CTGTTCTTGGCCCTGAGCCGGGGCCCAGGTGCTCGACCACAGATATCCTGTTTGGCCCCTGTCCCAGTTTTGTTCAGCCTTATTCTTTAACTAAACTTCCTTAGATCGGAAGAGCACACGTCTGAA

+

BBBBBFFFFFFFFFBFFFFFFFFBBFFFFFFFFFFFFFFFFFFFFFFFFFFFFFFFFFFFBFFFFFFFFBFBFFF/BFFBFF<<FFBB/<FFFFFFBFFBFBFFF/<F<F<BFFFF</7BFFFB//

@GWZHISEQ02:315:C9E6MANXX:5:1214:12157:47539

AGGAAGTTTAGTTAAAGAATAAGGCTGAACAAAACTGGGACAGGGGCCAAACAGGATATCTGTGGTCGAGCACCTGGGCCCCGGCTCAGGGCCAAGAACAGATGGTACTCAGATAAAGCGAAACTA

+

BBBBBFFFFFFFFFFFFFFFFFFFFFFFFFFFFFFFFFFFFFFFFFFFFFFFFFFFFFFFFFFFFFFFFFFFFFFFFFFFFFFFFFFFFFFFFFFFFFFFFFFFFFFFFFFFFFFFFFFFFFFFFF

@GWZHISEQ02:315:C9E6MANXX:5:1306:7167:2741

AGGAAGTTTAGTTAAAGAATAAGGCTGAACAAAACTGGGACAGGGGCCAAACAGGATATCTGTGGTCGAGCACCTGGGCCCCGGCTCAGGGCCAAGAACAGATGGTACTCAGATAAAGCGAAACTA

+

BBBBBFFFFFFFFFFFFFFFFFFFFFFFFFFFFFFFFFFFFFFFFFFFFFFFFFFFFFFFFFFFFFFFFFFFFFFFFFFFFFFFFFFFFFFFFFFFFFFFFFFFFFFFFFFFFFFFFFFFFFFFFF

@GWZHISEQ02:315:C9E6MANXX:5:2101:20238:81845

AGGAAGTTTAGTTAAAGAATAAGGCTGAACAAAACTGGGACAGGGGCCAAACAGGATATCTGTGGTCGAGCACCTGGGCCCCGGCTCAGGGCCAAGAACAGATGGTACTCAGATAAAGCGAAACTA

+

BBBBBFFFFFFFFFFFFFFFFFFBFFFBFFFF<F<<B/B<FFFFBFFFBBFFBFF<FBFFFFFFFFFFFFFFFFFFFFFFFFFFFFFFBFFFFFFFFFFFFFFFFFBFFFFFFFFFFFFFFBBBF<

@GWZHISEQ02:315:C9E6MANXX:5:2313:10962:19006

AGGAAGTTTAGTTAAAGAATAAGGCTGAACAAAACTGGGACAGGGGCCAAACAGGATATCTGTGGTCGAGCACCTGGGCCCCGGCTCAGGGCCAAGAACAGATGGTACTCAGATAAAGCGAAACTA

+

BBBBBFFFFFFFFFFFFFFFFFFBFFFFFFFFFFFFFBFFFFFFFFFFFFFFFFFFFFFFFFFFFFFFFFFFFFFFFFFFFFFFFFFFFFFFFFFFFFFFFFFFFFBFFFFFFFFFFFFFFFFFFF

@GWZHISEQ02:315:C9E6MANXX:5:1109:1741:24065

GGAAGTTTAGTTAAAGAATAAGGCTGAACAAAACTGGGACAGGGGCCAAACAGGATATCTGTGGTCGAGCACCTGGGCCCCGGCTCAGGGCCAAGAACAGATGGTACTCAGATAAAGCGAGATCGG

+

BBBBBFFFFFFFFFFFFFFBBFFFFFFFFFFFFFFFFFFFFFFFFFFBBBFFF<FFFFFBFFFFFFFFFFFFFFFFFFFFFFFF/7/77FFFFBB<FFFFFFFFF<FBF/BFF7BFFFFBFFFFFF

@GWZHISEQ02:315:C9E6MANXX:5:1113:5322:19475

GGAAGTTTAGTTAAAGAATAAGGCTGAACAAAACTGGGACAGGGGCCAAACAGGATATCTGTGGTCGAGCACCTGGGCCCCGGCTCAGGGCCAAGAACAGATGGTACTCAGATAAAGCGAAACTAG

+

BBBBBFFFFFFFFFFFFFFFFFFFFFFFFFFFFFFFFFFFFFFFFFFFFFFFFFFFFFFFFFFFFFFFFFFFFFFFFFFFFFFFFFFFFFFFFFFFFFFFFFFFFFFFFFFFFFFFFFFFFFFFFB

@GWZHISEQ02:315:C9E6MANXX:5:1114:21325:45825

GGAAGTTTAGTTAAAGAATAAGGCTGAACAAAACTGGGACAGGGGCCAAACAGGATATCTGTGGTCGAGCACCTGGGCCCCGGCTCAGGGCCAAGAACAGATGGTACTCAGATAAAGCGAAACTAG

+

BBBBBFFFFFFFFFFFFFFFFFFFFFFFFFFFFFFBFFFFFFFFBBFFFFFFFFFFFFFFFFF<FFFFFFFFFFF/FFFFFFFFFFFFFFFFFFFFFFFFFFFFFFFFFFFFFFFFFFFBFBBFFB

@GWZHISEQ02:315:C9E6MANXX:5:1202:18747:64379

GGAAGTTTAGTTAAAGAATAAGGCTGAACAAAACTGGGACAGGGGCCAAACAGGATATCTGTGGTCGAGCACCTGGGCCCCGGCTCAGGGCCAAGAACAGATGGTACTCAGATAAAGCGAAACTAG

+

BBB<B//<FF<FFF</BB/F/<BFFFBBFFF<FFFFFFBF<FBFF//FBFFFFBFBFFF/<FFF/FFFFFFFBFF<//<7BFFFBFFFFBFFFFFFBBBFFBF///</7<B/F/BFFFFBBFBFFB

@GWZHISEQ02:315:C9E6MANXX:5:1207:4579:21389

GGAAGTTTAGTTAAAGAATAAGGCTGAACAAAACTGGGACAGGGGCCAAACAGGATATCTGTGGTCGAGCACCTGGGCCCCGGCTCAGGGCCAAGAACAGATGGTACTCAGATAAAGCGAAACTAG

+

BBBBBFFFFFFFFFFFFFFFFFFFFFFFFFFFFFFFFFFFFFFFFFFFFFFFFFFFFFFFFFFFFFFFFFFFFFFFFFFFFFFFFFFFFFFFFFFFFFFFFFFFFFFFFFFFFFFFFFFFFFFFFF

@GWZHISEQ02:315:C9E6MANXX:5:1215:7984:41574

CCGGAAGTTTAGTTAAAGAATAAGGCTGAACAAAACTGGGACAGGGGCCAAACAGGATATCTGTGGTCGAGCACCTGGGCCCCGGCTCAGGGCCAAGAACAGATGGTACTCAGATAAAGCGAAACT

+

BBBBBFFFFFFFFFFFFFFFFFFFFFFFFFFFFFFFFFFFFFFFBFFFFFFFFFFFFFFFFFFFFFFFFFFFFFFFFFFFFFFFFFFFFFFFFFFFFFFFFFFFFFFFFFFFFFFFFFFFFFFFFF

@GWZHISEQ02:315:C9E6MANXX:5:1314:8978:33792

GGAAGTTTAGTTAAAGAATAAGGCTGAACAAAACTGGGACAGGGGCCAAACAGGATATCTGTGGTCGAGCACCTGGGCCCCGGCTCAGGGCCAAGAACAGATGGTACTCAGATAAAGCGAAACTAG

+

BBBBBBFFFFFFFFFFFFFFFFFFFFFFFFFFFFFFFFFFFFFFFFFFFFFFFFFFFFFFF<FFFFFFFFFFFFFFFFFFFFFFFFFFFFFFFFFFFFFFFFFFFFBFFFFFFFFFFF<BFFFFFB

@GWZHISEQ02:315:C9E6MANXX:5:2105:16271:24216

GGAAGTTTAGTTAAAGAATAAGGCTGAACAAAACTGGGACAGGGGCCAAACAGGATATCTGTGGTCGAGCACCTGGGCCCCGGCTCAGGGCCAAGAACAGATGGTACTCAGATAAAGCGAAACTAG

+

BBBBBFFFFFFFFFFFFFFFFFFFFFFFFFFFFFFFFFFFFFFFFFFFFFFFFFFFFFFFFFFFFFFFFFFFFFFFFFFFBFFFFFFFFFFFFFFFFFFFFFFFFFFFFFFFFFFFFFFFFFFFF<

@GWZHISEQ02:315:C9E6MANXX:5:2105:7210:48016

GGAAGTTTAGTTAAAGAATAAGGCTGAACAAAACTGGGACAGGGGCCAAACAGGATATCTGTGGTCGAGCACCTGGGCCCCGGCTCAGGGCCAAGAACAGATGGTACTCAGATAAAGCGAAACTAG

+

BBBBBBFFFFFFFFF/FFFFFFBFFFBFFFFFFFFFFFFFFFBFFFFFFBFFFFFFFFFFFFFFFFFFFFFFFFFFFFFFFFFFFFFFFFFFBFFFFFFFFFBB<FFFFFFFFFFBBFFFFBFFFB

@GWZHISEQ02:315:C9E6MANXX:5:2106:11632:35773

GGAAGTTTAGTTAAAGAATAAGGCTGAACAAAACTGGGACAGGGGCCAAACAGGATATCTGTGGTCGAGCACCTGGGCCCCGGCTCAGGGCCAAGAACAGATGGTACTCAGATAAAGCGAAACTAG

+

BBBBBFFFFFFFFFFFFFFFFFFFFFFFFFFFFFFFFFFFFFFFFFFFFFFFFFFFFFFFFFFFFFFFFFFFFFFFFFFFFFFFFFFFFFFFFFFFFFFFFFFFFFFFFFFFFFFFFFFFFFFFFF

@GWZHISEQ02:315:C9E6MANXX:5:2106:6278:75812

GGAAGTTTAGTTAAAGAATAAGGCTGAACAAAACTGGGACAGGGGCCAAACAGGATATCTGTGGTCGAGCACCTGGGCCCCGGCTCAGGGCCAAGAACAGATGGTACTTAGATAAAGCGAAACTAG

+

BBBBBFFFFFFFFFFFFFFFFFFFFFFFFFFFFFFFFFFFFFFFFFFFFFFFFFFFFFFFFFFFFFFFFFFFFFFFFFFFFFFFFFFFFFFFFFFFFFFFFFFFFFFFFFFFFFFFFFFFFFFFFF

@GWZHISEQ02:315:C9E6MANXX:5:2108:16904:24657

GGAAGTTTAGTTAAAGAATAAGGCTGAACAAAACTGGGACAGGGGCCAAACAGGATATCTGTGGTCGAGCACCTGGGCCCCGGCTCAGGGCCAAGAACAGATGGTACTCAGATAAAGCGAAACTAG

+

BBBBBFFFFFFFFFFFFFFFFFFFFFFFFFFFFFFFFFFFFFFFFFFFFFFFFFFFFFFFFFFFFFFFFFFFFFFFFFFFFFFFFFFFFFFFFFFFFFFFFFFFFFFFFFFFFFF<FFFFFFFFFF

@GWZHISEQ02:315:C9E6MANXX:5:2114:13553:89329

GGAAGTTTAGTTAAAGAATAAGGCTGAACAAAACTGGGACAGGGGCCAAACAGGATATCTGTGGTCGAGCACCTGGGCCCCGGCTCAGGGCCAAGAACAGATGGTACTCAGATAAAGCGAAACTAG

+

BBBBBFFFFFFFFFFFFFFFFFFFFFFFFFFFFFFFFFFFFFFFFFFFFFFFFFFFFFFFFFFFFFFFFFFFFFFFFFFFFFFFFFFBFFFFFFFFFFFFFFFFFFFFFFFFFFFFFFFFFFFFFF

@GWZHISEQ02:315:C9E6MANXX:5:2201:7305:18024

GGAAGTTTAGTTAAAGAATAAGGCTGAACAAAACTGGGACAGGGGCCAAACAGGATATCTGTGGTCGAGCACCTGGGCCCCGGCTCAGGGCCAAGAACAGATGGTACTCAGATAAAGCGAAACTAG

+

BBBBBFFFFFFFFFFFFFFFFFFFFFFFFFFFFFFBFFFFFFFFFFFFBFFFFFFFFFFFFFFFFFFFFFFFFFFF/FFFBFFF<F7FBFFFFFFFFFFFFFFFFFFFFFFFFFBFF<FFFFFFFF

@GWZHISEQ02:315:C9E6MANXX:5:2202:12634:9318

GGAAGTTTAGTTAAAGAATAAGGCTGAACAAAACTGGGACAGGGGCCAAACAGGATATCTGTGGTCGAGCACCTGGGCCCCGGCTCAGGGCCAAGAACAGATGGTACTCAGATAAAGCGAAACTAG

+

BBBBBFFF/<FFFFFF<FFFFFF/FF<BFFBFB/<FFFFFFF<F///FFFFFFFFFFFFFF<FFFFBFFBFFFFBF/B/FFFFFFFFFFFBBFFFFFFFFFFBFFFFFBFBFFFF<F<<FFF<FFF

@GWZHISEQ02:315:C9E6MANXX:5:2202:1734:68759

GGAAGTTTAGTTAAAGAATAAGGCTGAACAAAACTGGGACAGGGGCCAAACAGGATATCTGTGGTCGAGCACCTGGGCCCCGGCTCAGGGCCAAGAACAGATGGTACTCAGATAAAGCGAAACTAG

+

BBBBBFFFFFFFFFFFFFFFFFFFFFFFFFFFFFFFFFFFFFFFFFFFFFFFFFFFFFFFFFFFFFFFFFFFFFFFFFFFFFFFFFFFFFFFFFFFFFFFFFFFFFFFFFFFFFFFFFFFBFFFFF

@GWZHISEQ02:315:C9E6MANXX:5:2211:12723:23181

GGAAGTTTAGTTAAAGAATAAGGCTGAACAAAACTGGGACAGGGGCCAAACAGGATATCTGTGGTCGAGCACCTGGGCCCCGGCTCAGGGCCAAGAACAGATGGTACTCAGATAAAGCGAAACTAG

+

/BBBBFFFFFFFFFFFFFFFFFFFFFFFFFFFFFFFFFFFFFFFFFFFFFFFFFFFFFF<FFFFFFFFFFFFFFFFFFFFFFFFFFFFFFFFFFFFFFFFFFFFFFFFFFFFFFFFFFFFFFFFFF

@GWZHISEQ02:315:C9E6MANXX:5:2305:11302:34061

GGAAGTTTAGTTAAAGAATAAGGCTGAACAAAACTGGGACAGGGGCCAAACAGGATATCTGTGGTCGAGCACCTGGGCCCCGGCTCAGGGCCAAGAACAGATGGTACTCAGATAAAGCGAAACTAG

+

BBBBBFFFFFFFFFFFFFFFFFFFFFFFFFFFFFFFFFFFFFFFFFFFFFFFFFFFFFFFFFFFFFFFFFFFFFFFFFFFFFFFFFFFFFFFFFFFFFFFFFFFFFFFFFFFFFFFFFFFFFFFFF

@GWZHISEQ02:315:C9E6MANXX:5:2309:5424:29166

GGAAGTTTAGTTAAAGAATAAGGCTGAACAAAACTGGGACAGGGGCCAAACAGGATATCTGTGGTCGAGCACCTGGGCCCCGGCTCAGGGCCAAGAACAGATGGTACTCAGATAAAGCGAAACTAG

+

BBBBBFFFFFFFFFFFFFFFFFFFFFFFFFFFFFFFFFFFFFFFFFFFFFFFFFFFFFFFFFFFFFFFFFFFFFFFFFFFFFBFFFFFFFFFFFFFFFFFFFFFFFFFFFFFFFFFFFFFFFFFFF

@GWZHISEQ02:315:C9E6MANXX:5:2309:15614:35542

GGAAGTTTAGTTAAAGAATAAGGCTGAACAAAACTGGGACAGGGGCCAAACAGGATATCTGTGGTCGAGCACCTGGGCCCCGGCTCAGGGCCAAGAACAGATGGTACTCAGATAAAGCGAAACTAG

+

/BBBBFFFF<FFBFBFFFFFFFFFFFFFFFFFFFFFFBFF<FFFFFFFFBFFFBFFFFFFFFFFFFFFFFFFFFFFFFFFFFFFFFFFFFFFFFFFFFFF/BFFFFFFFFFFF/BFBFFBFFFFFF

@GWZHISEQ02:315:C9E6MANXX:5:2311:4114:14454

GGAAGTTTAGTTAAAGAATAAGGCTGAACAAAACTGGGACAGGGGCCAAACAGGATATCTGTGGTCGAGCACCTGGGCCCCGGCTCAGGGCCAAGAACAGATGGTACTCAGATAAAGCGAAACTAG

+

BBBBBFFFFFFFFFFFFFFFFFFFFFFFFFFFFFFFFFFFFFFFFFFFFFFFFFFFFFFFFFFFFFFFFFFFFFFFFFFFFFFFFFFFFFFFFFFFFFFFFFFFFFFFFFFFFFFFFFFFFFFFFF

@GWZHISEQ02:315:C9E6MANXX:5:2314:13705:63221

GGAAGTTTAGTTAAAGAATAAGGCTGAACAAAACTGGGACAGGGGCCAAACAGGATATCTGTGGTCGAGCACCTGGGCCCCGGCTCAGGGCCAAGAACAGATGGTACTCAGATAAAGCGAAACTAG

+

BBBBBFFFFFFFFFFFFFFFFFFFFFFFFFFFFFFFFFFFFFFFFFFFFFFFFFFFFFFFFFFFFFFFFFFFFFFFFFFFFFFFFFFFFFFFFFFFFFFFFFFFFFFFFFFFFFFFFFFFFFFFFF

@GWZHISEQ02:315:C9E6MANXX:5:2315:8063:95745

GGAAGTTTAGTTAAAGAATAAGGCTGAACAAAACTGGGACAGGGGCCAAACAGGATATCTGTGGTCGAGCACCTGGGCCCCGGCTCAGGGCCAAGAACAGATGGTACTCAGATAAAGCGAAACTAG

+

BBBBBFFFFFFFFFFFFFFFFFFFFFFFFFFFFFFFFFFFFFFFFFFFFFFFFFFFFFFFFFFFFFFFFFFFFFFFFFFFFFFFFFFFFFFFFFFFFFFFFFFFFFFFFFFFFFFFFFFFFFFFFB

@GWZHISEQ02:315:C9E6MANXX:5:2316:21253:72570

GGAAGTTTAGTTAAAGAATAAGGCTGAACAAAACTGGGACAGGGGCCAAACAGGATATCTGTGGTCGAGCACCTGGGCCCCGGCTCAGGGCCAAGAACAGATGGTACTCAGATAAAGCGAAACTAG

+

BBBBBFFFFFFFFFFFFFFFFFFFFFFFFFFFFFFFFFFFFFFFFFFFFFFFFFFFFFFF<FFFFFBFFFFFFFFFBFFFFFFFFFFFFFFFFFFFFFFFFFFFFFFFFFFFFFFFFFFFFFFFFF

@GWZHISEQ02:315:C9E6MANXX:5:1113:5322:19475

CTAGTTTCGCTTTATCTGAGTACCATCTGTTCTTGGCCCTGAGCCGGGGCCCAGGTGCTCGACCACAGATATCCTGTTTGGCCCCTGTCCCAGTTTTGTTCAGCCTTATTCTTTAACTAAACTTCC

+

BBBBBFFFFFFFFFFFFFFFFFFFFFFFFFFFFFFFFFFFFFFFFFFFFFFFFFFFFFFFFFFFFFFFFFFFFFFFFFFFFFFFFFFFFFFFFFFFFFFFFFFFFFFFFFFFFFFFFFFFFFFFFF

@GWZHISEQ02:315:C9E6MANXX:5:2312:5613:51042

CTGTTCTTGGCCCTGAGCCGGGGCCCAGGTGCTCGACCACAGATATCCTGTTTGGCCCCTGTCCCAGTTTTGTTCAGCCTTATTCTTTAACTAAACTTCCAGATCGGAAGAGCACACGTCTGAACT

+

BBBBBFF<BBBFBFFFFFFFFFFFFFFFFFBFFFFFFFFFFFFFFFFFFFFBFFBFFFFFFFBBFFFF<FFFFFFFFFB7FFFBFBFFFBBFFFFFFFFFFFFFFFFFFFFFFFFFFFFFFFFFFB

@GWZHISEQ02:315:C9E6MANXX:5:2212:15501:83468

GAAGTTTAGTTAAAGAATAAGGCTGAACAAAACTGGGACAGGGGCCAAACAGGATATCTGTGGTCGAGCACCTGGGCCCCGGCTCAGGGCCAAGAACAGATGGTACTCAGATAAAGCGAAACTAGC

+

BBBBBFFFFFFFFFFFFFFFFFFFFFFFFFFFFFFFFFFFFFFFFFFFFFFFFFFFFFFFFFFFFFFFFFFFFFFFFFFFFFFFFFFFFFFFFFFFFFFFFFFFFFFFFFFFFFFFFFFFFFFFFF

@GWZHISEQ02:315:C9E6MANXX:5:2316:5539:11062

ACGAAGTTTTGTTAAAGAATAAGGCTGAACAAAACTGGGACAGGGGCCAAACAGGATATCTGTGGTCGAGCACCTGGGCCCCGGCTCAGGGGCAAGAACAGATGGTACACAGATAAAGCGAAACTA

+

<//<//B<//<FFFF/<FFFB////</F<FFFFFFFFFFF//<</BB/F/B/F</</F/F/F<BBF//FF<///<BFF<</7FBB/<B//<//7B7<BBFFBF//777//7BB/77/7/77/7/77

@GWZHISEQ02:315:C9E6MANXX:5:1106:13593:86780

GCTAGTTTCGCTTTATCTGAGTACCATCTGTTCTTGGCCCTGAGCCGGGGCCCAGGTGCTCGACCACAGATATCCTGTTTGGCCCCTGTCCCAGTTTTGTTCAGCCTTATTCTTTAACTAAACTTC

+

B/<B/FF/<</<<<//B/</BFFFB<B/FF<FFF///F//F///FFB<F/FFBB<FFFFFFFFFF/F<B/</FFF/FF/BFF/</FFFBFBBFFBFB/BF</BBFFBFFFFB/BF/BFFFFF<FFF

@GWZHISEQ02:315:C9E6MANXX:5:1113:12099:6041

GCTAGTTTCGCTTTATCTGAGTACCATCTGTTCTTGGCCCTGAGCCGGGGCCCAGGTGCTCGACCACAGATATCCTGTTTGGCCCCTGTCCCAGTTTTGTTCAGCCTTATTCTTTAACTAAACTTC

+

BBBBBFFFF<FFFFFFFFFFFFFFFFFFFFFFFFFFFFFFFFFFFFFFFFFFFFFFFFFFFFFFFFFFFFFFFFFFFFBFFFFFFFFFFFFFFFBBFFFFFFFFFFFFFFFFFFFFFFFFFFFFFF

@GWZHISEQ02:315:C9E6MANXX:5:1201:19471:71040

GCTAGTTTCGCTTTATCTGAGTACCATCTGTTCTTGGCCCTGAGCCGGGGCCCAGGTGCTCGACCACAGATATCCTGTTTGGCCCCTGTCCCAGTTTTGTTCAGCCTTATTCTTTAACTAAACTTC

+

BBBBBFFFFFFFFFFFFFFFFFFFFFFFFFFFFFFFFFFFFFFFFFFFFFFFFFFFFFFFFFFFFFFFFFFFFFFFFFFFF<FFFFFFFFFFFFFFFFFFFFFFFFFFFFFFFFFBFFFF<FFFFF

@GWZHISEQ02:315:C9E6MANXX:5:2103:11962:3817

GCTAGTTTCGCTTTATCTGAGTACCATCTGTTCTTGGCCCTGAGCCGGGGCCCAGGTGCTCGACCACAGATATCCTGTTTGGCCCCTGTCCCAGTTTTGTTCAGCCTTATTCTTTAACTAAACTTC

+

BB/BBBFBF</<FFFFFF<F<<BFFFFFFFFFFB<B/FFFFF/<BBFBB/7/BFF/</FFFFFFFBFF<BFBFFBFFFFFFFFFFFFFFFFFB/<FFFFBFFFFFFFFFFFFFFFF<BF/FFFFFB

@GWZHISEQ02:315:C9E6MANXX:5:2204:10681:83994

GCTAGTTTCGCTTTATCTGAGTACCATCTGTTCTTGGCCCTGAGCCGGGGCCCAGGTGCTCGACCACAGATATCCTGTTTGGCCCCTGTCCCAGTTTTGTTCAGCCTTATTCTTTAACTAAACTTC

+

BBBBBFFFFFFFFFFFFFFFFFFFFFFFFFFFFFFFFFFFFFFFFFFFFBFFFFFFFFFFFFFFFFFFFFFFFFFFFFFFFFFFFFFFFFFFFFFFFFFFFFFFFFFFFFFFFFFFFFFFFFFFFF

@GWZHISEQ02:315:C9E6MANXX:5:2205:6493:43528

GCTAGTTTCGCTTTATCTGAGTACCATCTGTTCTTGGCCCTGAGCCGGGGCCCAGGTGCTCGACCACAGATATCCTGTTTGGCCCCTGTCCCAGTTTTGTTCAGCCTTATTCTTTAACTACACTTC

+

BBB//FF/<FF<FFFBFF/<<FF//BFB</<BF<BB<FFFBF/<B<FF<BBFFFFF/F/FF/B</<FFBFFFFFF/FB<<<</FFFFFFFFBF<<BFFFFF/BFFFF/<//<BF/FBBF7//77BF

@GWZHISEQ02:315:C9E6MANXX:5:2210:13645:37709

GCTAGTTTCGCTTTATCTGAGTACCATCTGTTCTTGGCCCTGAGCCGGGGCCCAGGTGCTCGACCACAGATATCCTGTTTGGCCCCTGTCCCAGTTTTGTTCAGCCTTATTCTTTAACTAAACTTC

+

BBBBBFFFBFFFFFFFFFFFFFFFFFF<FFF/FFFFFFBFFFFFFFFFFFFFBFBFFF/FFFBFBBFFFFFFFFFFFFFFBFBFFFBFFFFFFBFFFFFFFFFFFFFFFFFFFBF/FFFFBFBFBB

@GWZHISEQ02:315:C9E6MANXX:5:2301:10988:61544

GCTAGTTTCGCTTTATCTGAGTACCATCTGTTCTTGGCCCTGAGCCGGGGCCCAGGTGCTCGACCACAGATATCCTGTTTGGCCCCTGTCCCAGTTTTGTTCAGCCTTATTCTTTAACTAAACTTC

+

BBBBBFFFFFFFFFFFFFFFFFFFFFFFFFFFFFFFFFFFFF<FFFFFFFFFFFFFFFFFFFFFFFFFFFFBFFFFFFFFFFFFBFFFFFFFFFFBFFFBFF<BFFFFFFFFFFBFFFFF/FFFFF

@GWZHISEQ02:315:C9E6MANXX:5:1102:13770:13336

AAGTTTAGTTAAAGAATAAGGCTGAACAAAACTGGGACAGGGGCCAAACAGGATATCTGTGGTCGAGCACCTGGGCCCCGGCTCAGGGCCAAGAACAGATGGAAGATCGGAAGAGCGTCGTGTAGG

+

BBBBBFFFFFFFFFFFFFFFFFFFFFFFFFFFFFFFFFFFFFFFFFFFFFFFFFFFFFFFFFFFFFFFFFFFFFFFFFFFFFFFFFFFFFFFFFFFFFFFFFFFFFFFFFFFFFFFFBFFBFFFFF

@GWZHISEQ02:315:C9E6MANXX:5:1102:13770:13336

TCCATCTGTTCTTGGCCCTGAGCCGGGGCCCAGGTGCTCGACCACAGATATCCTGTTTGGCCCCTGTCCCAGTTTTGTTCAGCCTTATTCTTTAACTAAACTTAGATCGGAAGAGCACACGTCTGA

+

BBBBBFFFFFFFFFFFFFFFFFFFFFFFFFFFFFFFFFFFFFFFFFFFFFFFFFFFFFFFFFFFFFFFFFFFFFFFFFFFFFFFFFFFFFFFFFFFFFFFFFFFFFFFFFFFFFFFFFFFFFFFFF

@GWZHISEQ02:315:C9E6MANXX:5:1304:20199:81624

TGCTAGTTTCGCTTTATCTGAGTACCATCTGTTCTTGGCCCTGAGCCGGGGCCCAGGTGCTCGACCACAGATATCCTGTTTGGCCCCTGTCCCAGTTTTGTTCAGCCTTATTCTTTAACTAAACTT

+

BBBBBFFFFFFFFFFFFFFFFFFFFFFFFFFFFFFFFFFFFFFFFFFFFFFFFFFFFFFFFFFFFFFFFFFFFFFFFFFFFFFFFFFFFFFFFFFBFFFFFFFFFFFFFBFFFFFFFFFBFFFFFF

@GWZHISEQ02:315:C9E6MANXX:5:2104:10924:22164

GGCTAGTTTCGCTTTATCTGAGTACCATCTGTTCTTGGCCCTGAGCCGGGGCCCAGGTGCTCGACCACAGATATCCTGTTTGGCCCCTGTCCCAGTTTTGTTCAGCCTTATTCTTTAACTAAACTT

+

BBBBBFFFFFFFFFFFFFFFFFFFFFFFFFFFFFFFFFFFFFFFFFFFFFFFFFFFFFFFFFFFFFFFFFFFFFFFFFFFFFFFFFFFFFFFFFFFFFFFFFFBFFFFFFFFFFFFFFFFFFFFFF

@GWZHISEQ02:315:C9E6MANXX:5:2308:18771:45161

AGTTTAGTTAAAGAATAAGGCTGAACAAAACTGGGACAGGGGCCAAACAGGATATCTGTGGTCGAGCACCTGGGCCCCGGCTCAGGGCCAAGAACAGATGGTACTCAGATAAAGCGAAACTAGCAA

+

BBBBBFFFFFFFFFFFFFFFFFFFFFFFFFFFFFFFFFFFFFFFFFFFFFFFFFFFFFFFFFFFFFFFFFFFFFFFFFFFFFFFFFFFFFFFFFFFFFFFFFFFFFFFFFFFFFFFFFFFFFFFFF

@GWZHISEQ02:315:C9E6MANXX:5:2309:8623:76567

CTGCTAGTTTCGCTTTATCTGAGTACCATCTGTTCTTGGCCCTGAGCCGGGGCCCAGGTGCTCGACCACAGATATCCTGTTTGGCCCCTGTCCCAGTTTTGTTCAGCCTTATTCTTTAACTAAACT

+

BBBBBFFFFFFFFFFFFFFFFFFFFFFFFFFFFFFFFFFFFFFFFFFFFFFFFFFFFFFFFFFFFFFFFFFFFFFFFFFFFFFFFFFFFFFFFFFFFFFFFFFFFFFFFFFFFFFFFFFFFFFFFF

@GWZHISEQ02:315:C9E6MANXX:5:1108:19029:18865

GTTTAGTTAAAGAATAAGGCTGAACAAAACTGGGACAGGGGCCAAACAGGATATCTGTGGTCGAGCACCTGGGCCCCGGCTCAGGGCCAAGAACAGATGGTACTCAGATAAAGCGAAACTAGCAAC

+

BBBBBFFFFFFFFFFFFFFFFFFFFFFFFFFFFFFFFFFFFFFFFFFFFFFFFFFFFFFFFFFFFFFFFFFFFFFFFFFFFFFFFFFFFFFFFFFFFFFFFFFFFFFFFFFFFFFFFFFFFFFFFF

@GWZHISEQ02:315:C9E6MANXX:5:1113:8243:73876

GTTTAGTTAAAGAATAAGGCTGAACAAAACTGGGACAGGGGCCAAACAGGATATCTGTGGTCGAGCACCTGGGCCCCGGCTCAGGGCCAAGAACAGATGGTACTCAGATAAAGCGAAACTAGCAAC

+

BBBBBFFFFBFFFFFFFFFFFFFFFFFFFFFBFBBFF/BBFFFFFFFFFFFFFFFFFFFFFFFFFFFFFFFFFFFFFFFFFFFFFFFFFFFBFFFFFFFFFFFFFFFFFFFFFFFFF7FFFFFFFF

@GWZHISEQ02:315:C9E6MANXX:5:1113:13534:88029

GTTTAGTTAAAGAATAAGGCTGAACAAAACTGGGACAGGGGCCAAACAGGATATCTGTGGTCGAGCACCTGGGCCCCGGCTCAGGGCCAAGAACAGATGGTACTCAGATAAAGCGAAACTAGCAAC

+

BBBBBFFFFFFFFFFFFFFFFFFFFFFFFFFFFFFFFFFFBBBFFFFFFFFFFFFBFFFFFFFFFFFFFFF<FFFBFFFFFFFFFFBFFFBFFFFFBFFFFFFFFFFFFFFFFFFBFFFFFFFFBF

@GWZHISEQ02:315:C9E6MANXX:5:1209:12801:45957

GTTTAGTTAAAGAATAAGGCTGAACAAAACTGGGACAGGGGCCAAACAGGATATCTGTGGTCGAGCACCTGGGCCCCGGCTCAGGGCCAAGAACAGATGGTACTCAGATAAAGCGAAACTAGCAAC

+

BBBBBFFFFFFFFFFFFFFFFFFFFFFFFFFFFFFFFFFFFFFFFFFFFFFFFFFFFFFFFFFFFFFFFFFFFFFFFFFFFFFFFFFFFFFFFFFFFFFFFFFFFFFFFFFFFFFFFFFFFFFFF<

@GWZHISEQ02:315:C9E6MANXX:5:1303:9442:51307

GTTTAGTTAAAGAATAAGGCTGAACAAAACTGGGACAGGGGCCAAACAGGATATCTGTGGTCGAGCACCTGGGCCCCGGCTCAGGGCCAAGAACAGATGGTACTCAGATAAAGCGAAACTAGCAAC

+

BBBBBFFFFFFFFFFFFFFFFFB<FBFF/B<<<BFFFF/BBFFFFFFFFFFFFFFFFFFFBFFFFFFFFFFBFFFFFFFFBFFF<<BFFFFF/FFFFFFBB<BBFFFFFF/BFFB<FFFBFFF7FF

@GWZHISEQ02:315:C9E6MANXX:5:2103:7588:16647

GTTTAGTTAAAGAATAAGGCTGAACAAAACTGGGACAGGGGCCAAACAGGATATCTGTGGTCGAGCACCTGGGCCCCGGCTCAGGGCCAAGAACAGATGGTACTCAGATAAAGCGAAACTAGCAAC

+

BBBBBFFFFFFFFFFFFFFFFFFFFFFFFFFFFFFFFFFFFFFFFFFFFFFFFFFFFFFFFFFFFFFFFFFFFFFFFFFFFFFFFFFFFFFFFFFFFFFFFFFFFFFFFFFFFFFFFFFFFFFFFF

@GWZHISEQ02:315:C9E6MANXX:5:2107:11158:18706

GTTTAGTTAAAGAATAAGGCTGAACAAAACTGGGACAGGGGCCAAACAGGATATCTGTGGTCGAGCACCTGGGCCCCGGCTCAGGGCCAAGAACAGATGGTACTCAGATAAAGCGAAACTAGCAAC

+

BBBBBFFFFFFFFFFFFFFFFFFFFFFFFFFFFFFFFFFFFFFFFFFFFFFFFFFFFFFFFFFFFFFFFFFFFFFFFFFFFFFFFFFFFFFFFFFFFFFFFFFFFFFFFFFFFFFFFFFFFFFFFF

@GWZHISEQ02:315:C9E6MANXX:5:2116:10570:51302

GTTTAGTTAAAGAATAAGGCTGAACAAAACTGGGACAGGGGCCAAACAGGATATCTGTGGTCGAGCACCTGGGCCCCGGCTCAGGGCCAAGAACAGATGGTACTCAGATAAAGCGAAACTAGCAGA

+

BBBBBFFFFFFFFFFFFFFFFFFFFFFFFFFFFFFFFFFFFFFFFFFFFFFFFFFFFFFFFFFFFFFFFFFFFFFFFFFF/FFFFFFFFFFFFFFFFFFFFFFFFFFFFFFFFFFFFFFFFFFFFF

@GWZHISEQ02:315:C9E6MANXX:5:2116:6205:78406

GTTTAGTTAAAGAATAAGGCTGAACAAAACTGGGACAGGGGCCAAACAGGATATCTGTGGTCGAGCACCTGGGCCCCGGCTCAGGGCCAAGAACAGATGGTACTCAGATAAAGCGGAGATCGGAAG

+

BBBBBFFFFFFFFFFFFFFFFFFFFFFFFFFFFFFFFFFFFBFFFFFFFFFFFFFFFFFFFFFFFFBFFFFFF<<BFFF<FFFFFFBFFFFFFFFFFFBFFBFFFFFFFF<FFFFFBF</FF<BFB

@GWZHISEQ02:315:C9E6MANXX:5:2116:3813:98025

GTTTAGTTAAAGAATAAGGCTGAACAAAACTGGGACAGGGGCCAAACAGGATATCTGTGGTCGAGCACCTGGGCCCCGGCTCAGGGCCAAGAACAGATGGTACTCAGATAAAGCGAAACTAGCAAC

+

BBBBBFFFFFFFFFFFFFFFFFFFFFFFFFFFFFFFFFFBFFFFFFFFFFBFFFFFFFFFFFFFFFFFFFFFFFFFFFFFFFFFFFFFFFFFFFFFFFFFFFFFFFFFFFFFFFFFFFFFFFF/FB

@GWZHISEQ02:315:C9E6MANXX:5:2313:4809:10184

GTTTAGTTAAAGAATAAGGCTGAACAAAACTGGGACAGGGGCCAAACAGGATATCTGTGGTCGAGCACCTGGGCCCCGGCTCAGGGCCAAGAACAGATGGTACTCAGATAAAGCGAAACTAGCAAC

+

BBBBBFFF/<FFFFFFFFBFFFFFFFFFFFFFFFFFFFFBBFF<FFFFFFBFFFFFFFFFFFFFFFFFFF<BFFFFFFF/FBFFFFFFB//BFF<FBFFFFFFBFFBFFFFFFFBBFFFFFFFFFB

@GWZHISEQ02:315:C9E6MANXX:5:1209:12801:45957

GTTGCTAGTTTCGCTTTATCTGAGTACCATCTGTTCTTGGCCCTGAGCCGGGGCCCAGGTGCTCGACCACAGATATCCTGTTTGGCCCCTGTCCCAGTTTTGTTCAGCCTTATTCTTTAACTAAAC

+

BBBBBFFFFFFFFFFFFFFFFFFFFFFFFFFFFFFFFFFFFFFFFFFFFFFFFFFFFFFFFFFFFFFFFFFFFFFFFFFFFFFFFFFFFFFFFFFFFFFFFFFFFFFFFFFFFFFFFFFFFFFFF7

@GWZHISEQ02:315:C9E6MANXX:5:2115:6436:13646

CTGAGTACCATCTGTTCTTGGCCCTGAGCCGGGGCCCAGGTGCTCGACCACAGATATCCTGTTTGGCCCCTGTCCCAGTTTTGTTCAGCCTTATTCTTTAACTAAACAGATCGGAAGAGCACACGT

+

BBBBB<<FBFFFFFBFBFBFBFFFFFFFFFFBF<FFFFFFFFFFFFBFFFFFFF<F<FF<FFBFFFFFFFFFFFFFFFFF/F/<FFBFFFFFFFFFFFFFBFFFFFFBFFFFFFFFBFF<FFFFFF

@GWZHISEQ02:315:C9E6MANXX:5:2116:6205:78406

CCGCTTTATCTGAGTACCATCTGTTCTTGGCCCTGAGCCGGGGCCCAGGTGCTCGACCACAGATATCCTGTTTGGCCCCTGTCCCAGTTTTGTTCAGCCTTATTCTTTAACTAAACAGATCGGAAG

+

BBBBBFFFFFFFFFFFFFFFFFFFFFFFFFFFFFFFFFFFFFFFFFFFFFFFFFFFF<FFFFFFFFFFFFFFFFBFFFFFFFFFFFFFBFFFFFFFFFFFFFFFFFFFFFFFFFFFFFFFFFF/BF

@GWZHISEQ02:315:C9E6MANXX:5:1205:9081:85286

TTTAGTTAAAGAATAAGGCTGAACAAAACTGGGACAGGGGCCAAACAGGATATCTGTGGTCGAGCACCTGGGCCCCGGCTCAGGGCCAAGAACAGATGGTACTCAGATAAAGCGAAACTAGCAACA

+

BBBBBFFFFFFFFFFFFFFFFFFFFFFFFFFFFFFFFFFFFFFFFFFFFFFFFFFFFFFFFFFFFFFFFFFFFFFFFFFFFFFFFFFFFFFFFFFFFFFFFFFFFFFFFFFFFFFFFFFFFFFFFF

@GWZHISEQ02:315:C9E6MANXX:5:1213:4996:3552

TTTAGTTAAAGAATAAGGCTGAACAAAACTGGGACAGGGGCCAAACAGGATATCTGTGGTCGAGCACCTGGGCCCCGGCTCAGGGCCAAGAACAGATGGTACTCAGATAAAGCGAAACTAGCAACA

+

BBBBBFFFFFFFFFFFBFFFFFFFFFFFFFFFFFFFFFFFFFFFBFFFFF<FFFFFFFBFFFFFFFFFFFFFFFFFFBFFFFFFFFFFFFFFFFFFFFFFFFFFFFFFFFF/FFFFFFFFBFFBFF

@GWZHISEQ02:315:C9E6MANXX:5:1215:9333:46976

TTTAGTTAAAGAATAAGGCTGAACAAAACTGGGACAGGGGCCAAACAGGATATCTGTGGTCGAGCACCTGGGCCCCGGCTCAGGGCCAAGAACAGATGGTACTCAGATAAAGCGAAACTAGCAACA

+

BBBBBFFFFFFFFFFFFFFFFFFFFF<FFFFFFFFFFFFFFFFFFFFFFFFFFFFFFFFFFFFFFFFFFFFFFBFFFFFFFFFFFFFBBFFFFFFFFFFFFFFFFFFF/FFFFBBBFFFFFFFFF/

@GWZHISEQ02:315:C9E6MANXX:5:2109:1936:69565

TTTAGTTAAAGAATAAGGCTGAACAAAACTGGGACAGGGGCCAAACAGGATATCTGTGGTCGAGCACCTGGGCCCCGGCTCAGGGCCAAGAACAGATGGTACTCAGATAAAGCGAAACTAGCAACA

+

BBBBBFFFFBFBFFB///FFFFFFFFFFFFFBBFFFFBFBBFBF</FFFFFFFFBFFFFFFFFFBBFFFFFFFFBFFFF<BFB<FFFF/BFFFFFFBB<FFFFFFFF7FFFFFFFBFFFFFFFFFF

@GWZHISEQ02:315:C9E6MANXX:5:2312:19625:36789

TTTAGTTAAAGAATAAGGCTGAACAAAACTGGGACAGGGGCCAAACAGGATATCTGTGGTCGAGCACCTGGGCCCCGGCTCAGGGCCAAGAACAGATGGTACTCAGATAAAGCGAAACTAGCAACA

+

/<B</BFF/<BF/FF///<F//<//<<<B<B<FFFB/<FFBBFFFFBFF</FFF//BB</<7BFFFFFFF/<</FFFFF<BFF<<F/<FFFBFF<B/FFFFFFF/7/7FFBBBBF<BFFFFF/BB/

@GWZHISEQ02:315:C9E6MANXX:5:1101:2792:72839

GTTAAAGAATAAGGCTGAACAAAACTGGGACAGGGGCCAAACAGGATATCTGTGGTCGAGCACCTGGGCCCCGGCTCAGGGCCAAGAACAGATGGTACTCAGATAAAGCGAAAAGATCGGAAGAGC

+

BBBBBFFFFFFFFFFFFFFFFFFFFFFFFFFFFFFFFFFFFFFFFFFFFFFFFFFFFFFFFBFFFFFFFFFFFFFFFFFFFFFFFFFFFFFFFFFFFFFF<FFFFFFFFFFFFFFFFFFFFFFFFF

@GWZHISEQ02:315:C9E6MANXX:5:1105:4452:95639

GTTAAAGAATAAGGCTGAACAAAACTGGGACAGGGGCCAAACAGGATATCTGTGGTCGAGCACCTGGGCCCCGGCTCAGGGCCAAGAACAGATGGTACTCAGATAAAGCGAAACTAGCAACAGTTT

+

BBBBBFFFFFFFFFFFFFFFFFFFFFFFFFFFFFFFFFFFFFFFFFFFFFFFFFFFFFFFFFFFFFFFFFFFFFFFFFFFFFFFFFFFFFFFFFFFFFFFFFFFFFFFFFFFFFFFFFFFFFFFFB

@GWZHISEQ02:315:C9E6MANXX:5:1111:7052:30188

GTTAAAGAATAAGGCTGAACAAAACTGGGACAGGGGCCAAACAGGATATCTGTGGTCGAGCACCTGGGCCCCGGCTCAGGGCCAAGAACAGATGGTACTCAGATAAAGCGAAACTAGCAACAGTTT

+

BBBBBFFFFFFFFFFFFFFFFFFFFFFFFFFFFFFFFFFFFFFFFFFFFFFFFFFFFFFFFFFFFFFFFFFFFFFFFFFFFFFFFFFFFFFFFFFFFFFFFFFFFFFFFFFFFFFFFFFFFFFFFF

@GWZHISEQ02:315:C9E6MANXX:5:1210:18688:73023

GTTAAAGAATAAGGCTGAACAAAACTGGGACAGGGGCCAAACAGGATATCTGTGGTCGAGCACCTGGGCCCCGGCTCAGGGCCAAGAACAGATGGTACTCAGATAAAGCGAAACTAGCAACAGTTT

+

BBBBBFBFFFFFFFFFFFFFFFFFFFFFFFFFFFFBFFFFFFFFFBFFFFFFFFFFFFFFFFFFFFFFFFFFFB<BFFFFFFFBFFFFFFFFF<FFFFFFFFFFFFFFFFFBFFBFFFFFFFFFF<

@GWZHISEQ02:315:C9E6MANXX:5:1215:14667:5803

GTTAAAGAATAAGGCTGAACAAAACTGGGACAGGGGCCAAACAGGATATCTGTGGTCGAGCACCTGGGCCCCGGCTCAGGGCCAAGAACAGATGGTACTCAGATAAAGCGAAGATCGGAAGAGCGT

+

BBBBBFFFFFFFFFFFFFFFFFFFFFFFFFFFFFFFFFFFFFFFFFFFFFFFFFFFFFFFFFFFFFFFFFFFFFFFFFFFFFFFFFFFFFFFFFFFFFFFFFFFFFFFFFFFFFFFFFFFFFFFF7

@GWZHISEQ02:315:C9E6MANXX:5:1306:20349:8180

GTTAAAGAATAAGGCTGAACAAAACTGGGACAGGGGCCAAACAGGATATCTGTGGTCGAGCACCTGGGCCCCGGCTCAGGGCCAAGAACAGATGGTACTCAGATAAAGCGAAACTAGCAACAGTTT

+

BBBBBFFFFFFFFFFFFFFFFFFFFFFFFFFFFFFFFFFFFFFFFFFFFFFFFFFFFFFFFFFFFFFFFFFFFFFFFFFFFFFFFFFFFFFFFFFFFFFFFFFFFFFFFFFFFFFFFFFFFFFBFF

@GWZHISEQ02:315:C9E6MANXX:5:1308:10391:36827

GTTAAAGAATAAGGCTGAACAAAACTGGGACAGGGGCCAAACAGGATATCTGTGGTCGAGCACCTGGGCCCCGGCTCAGGGCCAAGAACAGATGGTACTCAGATAAAGCGAAACTAGCAACAGTTT

+

BBBBBFFFFFFFFFFFFFFFFFFFFFFFFFFFFFFFFFFFFFFFFFFFFFFFFFFFFFBFFFFFFFFFFFFFFFFFFFFFFFFFFFFFFFFFFFFFFFFFFFFFFFFFFFFFFFFFFFFFFFFFFF

@GWZHISEQ02:315:C9E6MANXX:5:2102:19793:73202

GTTAAAGAATAAGGCTGAACAAAACTGGGACAGGGGCCAAACAGGATATCTGTGGTCGAGCACCTGGGCCCCGGCTCAGGGCCAAGAACAGATGGTACTCAGATAAAGCGAAACTAGCAACAGTTT

+

BBBBBFFFFFFFFFFFFFFFFFFFFFFFFFFFFFFFFFFFFFFFFFFFFFFFFFFFFFFFFFFFFFFFFFFFFFFFFFFF<FFFFFFFFFFFFFFFFFFFFFFFFFFFFFFFFFFFFFFFFFFBFB

@GWZHISEQ02:315:C9E6MANXX:5:2116:19037:99769

GTTAAAGAATAAGGCTGAACAAAACTGGGACAGGGGCCAAACAGGATATCTGTGGTCGAGCACCTGGGCCCCGGCTCAGGGCCAAGAACAGATGGTACTCAGATAAAGCGAAACTAGCAACAGTTT

+

BBBBBFFFFFFFFFFFFFFFFFFFFFFFFFFFFFFFFFFFFFFFFFFFFFFFFFFFFFFFFFFFFFFFFFFFFFFFFFFFFFFFFFFFFFFFFFFFFFFFFFFFFFFFFFFFFFFFFFFFFFFFFF

@GWZHISEQ02:315:C9E6MANXX:5:2205:19306:33977

GTTAAAGAATAAGGCTGAACAAAACTGGGACAGGGGCCAAACAGGATATCTGTGGTCGAGCACCTGGGCCCCGGCTCAGGGCCAAGAACAGATGGTACTCAGATAAAGCGAAACTAGCAACAGTTT

+

BBBBBFFFFFFFFFFFFFFFFFFFFFFFFFFFFFFFFFFFFFFFFFFFFFFFFFFFFFFFFFFFFFFFFFFFFFFFFFFFFFFFFFFFFFFFFFFFFFFFFFFFFFFFFFFFFFFFFFFFFFFFFF

@GWZHISEQ02:315:C9E6MANXX:5:2307:19349:73564

GTTAAAGAATAAGGCTGAACAAAACTGGGACAGGGGCCAAACAGGATATCTGTGGTCGAGCACCTGGGCCCCGGCTCAGGGCCAAGAACAGATGGTACTCAGATAAAGCGAAACTAGCAACAGTTT

+

BBBBBFFFFFFFFFFFFFFFFFFFFFFFFFFFFFFFFFFFFFFFFFFFFFFFFFFFFFFFFFFFFFFFFFFFFFFFFFFFFFFFFFFFFFFFFFFFFFFFFFFFFFFFFFFFFFFFFFFFFFFFFF

@GWZHISEQ02:315:C9E6MANXX:5:2308:11556:97386

GTTAAAGAATAAGGCTGAACAAAACTGGGACAGGGGCCAAACAGGATATCTGTGGTCGAGCACCTGGGCCCCGGCTCAGGGCCAAGAACAGATGGTACTCAGATAAAGCGAAACTAGCAACAGTTT

+

BBBBBFFFFFFFFFFFFFFFFFFFFFFFFFFFFFFFFFFFFFFFFFFFFFFFFFF<FFFFFFFFFFFFFFFFFFFFFFFFFFFFFFF<FBFFFFFFFFFFFFFFFFFFFFFFFFFFFFFFFFFFFF

@GWZHISEQ02:315:C9E6MANXX:5:2315:19202:9020

GTTAAAGAATAAGGCTGAACAAAACTGGGACAGGGGCCAAACAGGATATCTGTGGTCGAGCACCTGGGCCCCGGCTCAGGGCCAAGAACAGATGGTACTCAGATAAAGCGAAACTAGCAACAGTTT

+

BBBBBFFFFFFFFFFFFBFFFFFFFFFFFFFFFFFFFFFFFFFFFFFFFFFFFFFFFFFFFBFFFFFFFFFFFFFFFFFFFFFFFFFFFFFFFFFFFFFFFFFFFFFFFFFBFFFFFFFFFBFFFF

@GWZHISEQ02:315:C9E6MANXX:5:2315:9001:72303

GTTAAAGAATAAGGCTGAACAAAACTGGGACAGGGGCCAAACAGGATATCTGTGGTCGAGCACCTGGGCCCCGGCTCAGGGCCAAGAACAGATGGTACTCAGATAAAGCGAAACTAGCAACAGTTT

+

BBBBBFFFFFFFFFFFFFFFFFFFFFFFFFFFFFFFFFFFFFFFFFFFFFFFFFFFFFFFFFFFFFFFFFFFFFFFFFFFFFFFFF<FFFFFFFFFFF/FFFBFFFFFFFBFFFFFFFFFFFFFFF

@GWZHISEQ02:315:C9E6MANXX:5:1101:2792:72839

TTTCGCTTTATCTGAGTACCATCTGTTCTTGGCCCTGAGCCGGGGCCCAGGTGCTCGACCACAGATATCCTGTTTGGCCCCTGTCCCAGTTTTGTTCAGCCTTATTCTTTAACAGATCGGAAGAGC

+

BBBBBFFFFFFFFFFFFFFFFFFFBFFFFFFFFFFFFFFFFFFFFFFFFFFFFFFFFFFFFFFFFFFFFFFFFFFFBFFFFFFFFFFFFFFFFFFFFFFFFFFFFFFFFFFFFFFFFFFFFFFFFF

@GWZHISEQ02:315:C9E6MANXX:5:2114:16434:39879

TTAAAGAATAAGGCTGAACAAAACTGGGACAGGGGCCAAACAGGATATCTGTGGTCGAGCACCTGGGCCCCGGCTCAGGGCCAAGAACAGATGGTACTCAGATAAAGCGAAACTAGCAACAGTTTC

+

BB/BBF/FFFFFFFFFBB<BFFBFFF<FFFB/FBB/<FF<<<F<FFFFFFFF/FFFFFFBFFFB/<FF/<</BB/7/F<FFF<FBFF<FFF/FFBF/<BFF//</7B<BBFBFB/FFFBFFF/7B7

@GWZHISEQ02:315:C9E6MANXX:5:2201:13575:58005

TTAAAGAATAAGGCTGAACAAAACTGGGACAGGGGCCAAACAGGATATCTGTGGTCGAGCACCTGGGCCCCGGCTCAGGGCCAAGAACAGATAGATCGGAAGAGCGTCGTGTAGGGAAAGAGTGTT

+

BBBBBFFFFFFFFFFFFFFFFFFFFFFFFFFFF<<B<FFFFFFFFFFFFFBFFFBFFBFFFFBFFBFFFFFFFFFFFFFFFFFFFFFFFFFFFFFFFFBFFFFFFFBFF7BFFFFFFFFFFF7BFF

@GWZHISEQ02:315:C9E6MANXX:5:1303:20853:16378

CTGAGTACCATCTGTTCTTGGCCCTGAGCCGGGGCCCAGGTGCTCGACCACAGATATCCTGTTTGGCCCCTGTCCCAGTTTTGTTCAGCCTTATTCTTTAAGATCGGAAGAGCACACGTCTGAACT

+

BBBBBFFFFFFFFFFFFFFFFFFFFFFFFFFFFFFFFFFFFFFFFFFFFFFFFFFFFFFBFFFFFBFFFFFFFFFFFFBFFFFFFFFFFFFFFFFFFFFFFBFFFBFFFFFFFFFFFFFFFF<FFB

@GWZHISEQ02:315:C9E6MANXX:5:2201:13575:58005

ATCTGTTCTTGGCCCTGAGCCGGGGCCCAGGTGCTCGACCACAGATATCCTGTTTGGCCCCTGTCCCAGTTTTGTTCAGCCTTATTCTTTAAAGATCGGAAGAGCACACGTCTGAACTCCAGTCAC

+

BBBBBFFFFFFB<FFFFF/FFFFF<FFFFF<FFFFFFFFBFFFFFFFFFFFFFFBFBBFFFBBFFFFFF<FFFFFFFFFFFF<FF7FFFFFFFFFFFFFFFF/FFFFBFFBBBFFFFFFFFFFFF7

@GWZHISEQ02:315:C9E6MANXX:5:1108:10353:37217

TAAAGAATAAGGCTGAACAAAACTGGGACAGGGGCCAAACAGGATATCTGTGGTCGAGCACCTGGGCCCCGGCTCAGGGCCAAGAACAGATGGTACTCAGATAAAGCGAAACTAGCAACAGTTTCT

+

BBBBBFFFFFFFFFFFFFFFFFFFFFFFFFFFFFFFFFFFFFFFFFFFFFFFFFFFFFFFFFFFFFFFFFFFFFFFFFFFFFFFFFFFFFFFFFFFFF<FFF/FFFFFFFFFFFFFFFFFFFBFFF

@GWZHISEQ02:315:C9E6MANXX:5:1101:18410:96935

AAAGAATAAGGCTGAACAAAACTGGGACAGGGGCCAAACAGGATATCTGTGGTCGAGCACCTGGGCCCCGGCTCAAGGCCAAGAACAGATGGTACTCAGATAAAGCGAAACTAGCAACAGTTTCTG

+

BBBBBFFFFFFFFFFFFFFFFFFFFFFFFFFFFFFFFFFFFFFFFFFFFFFFBFFFFFBFFFFFFFBBFFFFFFFFFFFFFFFFFFFFFFFFFFFFFFFFFFFFFF<<<BFBBFFFFFBBF<FFFB

@GWZHISEQ02:315:C9E6MANXX:5:1309:9248:81282

AAAGAATAAGGCTGAACAAAACTGGGACAGGGGCCAAACAGGATATCTGTGGTCGAGCACCTGGGCCCCGGCTCAGGGCCAAGAACAGATGGTACTCAGATAAAGCGAAACTAGCAACAGTTTCTG

+

BBBBBFFFFFFFFFFFFFFFFFFFFFFFFFFFFFFFFFFFFFFFFFFFFFFFFFFFFFFFFFFFFFFFFFFFFFFFFFFFFFFFFFFFFFFFFFFFFFFFFFFFFFFFFFFFFFFFFFFFFFFFFF

@GWZHISEQ02:315:C9E6MANXX:5:2210:1726:96254

AAAGAATAAGGCTGAACAAAACTGGGACAGGGGCCAAACAGGATATCTGTGGTCGAGCACCTGGGCCCCGGCTCAGGGCCAAGAACAGATGGTACTCAGATAAAGCGAAACTAGCAACAGTTTCTG

+

BBBB<FFFFFFFFFBBFBFFFFFFFFFFFFFFBFFFFFFFFFFFFFFFFFFFFFFFFFFFFFFFFFFFFFFFFFFFFFFFFFFFFFFFFFFFFFFFFFFFFFFFFFFFFFFFFFBFFF/FBFFFFF

@GWZHISEQ02:315:C9E6MANXX:5:2314:1971:2383

AAAGAATAAGGCTGAACAAAACTGGGACAGGGGCCAAACAGGATATCTGTGGTCGAGCACCTGGGCCCCGGCTCAGGGCCAAGAACAGATGGTACTCAGATAAAGCGAAACTAGCAACAGTTTCTG

+

BBBBBFFFFFFFFFFFFFFFFFFFFFFFFFFFFFFFFFFFFFFFFFFFFFFFFFFFFFFFFFFFFFFFFFFFFFFFFFFFFFFFFFFFFFFFFFFFFFFFFFFFFFFFFFFFFFFFFFFFFFFFFF

@GWZHISEQ02:315:C9E6MANXX:5:1313:11173:56795

CAGAAACTGTTGCTAGTTTCGCTTTATCTGAGTACCATCTGTTCTTGGCCCTGAGCCGGGGCCCAGGTGCTCGACCACAGATATCCTGTTTGGCCCCTGTCCCAGTTTTGTTCAGCCTTATTCTTT

+

BBBBBFFFFFFFFFFFFBFFFFFFFFFFFFFFFFFFFFFFFFFFFFFFFFFFFFFFFFFFFFFFFFFFFFFFFFFFFFFFFFFFFFFFFFFBBFFFFFFFFFFBFFFFFFFFFFFFFFF7FFFBBB

@GWZHISEQ02:315:C9E6MANXX:5:2111:1730:85505

CAGAAACTGTTGCTAGTTTCGCTTTATCTGAGTACCATCTGTTCTTGGCCCTGAGCCGGGGCCCAGGTGCTCGACCACAGATATCCTGTTTGGCCCCTGTCCCAGTTTTGTTCAGCCTTATTCTTT

+

BBBBBFFFFFFFFFFFFFFFFFFFFFFFBBFFBBBF/<FFFBFFFFFFFFFFFFFFFFF/BFFBFFFFBFFFFFFF/<BFFF/FFFFFB7BF/<BFFFFFFBFFF/<FFF/<B/B7FBFFBFFFFF

@GWZHISEQ02:315:C9E6MANXX:5:1316:14794:10926

CCAGAAACTGTTGCTAGTTTCGCTTTATCTGAGTACCATCTGTTCTTGGCCCTGAGCCGGGGCCCAGGTGCTCGACCACAGATATCCTGTTTGGCCCCTGTCCCAGTTTTGTTCAGCCTTATTCTT

+

BBBBBFFFFFFFFFFFFFFFFFFFFFFFFFFFFFFFFFFFFFFFFFFFFFFFFFFFFFFFFFFFFFFFFBFBFFFFFFFFFFFFFFFFFFFFFFFFFFFFFFFFFFBBBF<FFFFFFFFFFFFFFF

@GWZHISEQ02:315:C9E6MANXX:5:2215:10533:74465

CCAGAAACTGTTGCTAGTTTCGCTTTATCTGAGTACCATCTGTTCTTGGCCCTGAGCCGGGGCCCAGGTGCTCGACCACAGATATCCTGTTTGGCCCCTGTCCCAGTTTTGTTCAGCCTTATTCTT

+

</<</<B/FF/<FFFFFBBF<F<<FFFFFFFFFFFFFFFFFFFFFFFF/FFFF<F/FFFFFBF/FFFFF/BFFFFFFBFFF/FFFFFFFFFFBF<FFFFBFFBFFBF/FBBFBFFFFF/B/<BFFF

@GWZHISEQ02:315:C9E6MANXX:5:1314:19519:94660

GAATAAGGCTGAACAAAACTGGGACAGGGGCCAAACAGGATATCTGTGGTCGAGCACCTGGGCCCCGGCTCAGGGCCAAGAACAGATGGTACTCAGATAAAGCGAAACTAGCAACAGTTTCTGGAA

+

BBBBBFFFFFFFFFFFFFFFFFFFFFFFFFFFFFFFFFFFFFFFFFFFFFFFBFFFFFFFFFFFFFFFFFFFFFFFFFFFFFFFFFFFFFFFFFFFFFFFFFFFFFFFFFFFFFFFFBFFFFFFFF

@GWZHISEQ02:315:C9E6MANXX:5:2103:16764:63463

GAATAAGGCTGAACAAAACTGGGACAGGGGCCAAACAGGATATCTGTGGTCGAGCACCTGGGCCCCGGCTCAGGGCCAAGAACAGATGGTACTCAGATAAAGCGAAACTAGCAACAGTTTCTGGAA

+

BBBBBFFFFFFFFFFFFFFFFFFFFFBFFFFFFFFFFFFFFFFFFFFFFFFFFFBFFFFFFFFFFFFFFFFFFFFFFFFFFFFFFFFFFFFFFFFFFFFFFFFFF<BFFFFFFFFFFFFFFFFFFF

@GWZHISEQ02:315:C9E6MANXX:5:2213:8949:44091

GAATAAGGCTGAACAAAACTGGGACAGGGGCCAAACAGGATATCTGTGGTCGAGCACCTGGGCCCCGGCTCAGGGCCAAGAACAGATGGTACTCAGATAAAGCGAAACTAGCAACAGTTTCTGGAA

+

BBBBBFFFFFFFFFFFFFFFFFFFFFFFFFFFFFFFFFFFFFFFFFFFFFFFFFFFFFFFFFFFFFFFFFFFFFFFFFFFFFFFFFFFFFFFFFFFFFFFFFFFFFFFFFFFFFFFFFFFFFFFFF

@GWZHISEQ02:315:C9E6MANXX:5:1104:17532:90459

ATAAGGCTGAACAAAACTGGGACAGGGGCCAAACAGGATATCTGTGGTCGAGCACCTGGGCCCCGGCTCAGGGCCAAGAACAGATGGTACTCAGATAAAGCGAAACTAGCAACAGTTTCTGGAAAG

+

BBBBBFFFFFFFFFFFFFFFFFFFFFFFFFFFFFFFFFFFFFFFFFFFFFFFFFFFFFFFFFFFFFFFFFFFFFFFFFFFFFFFFFFFFFFFFFFFFFFFFFFFFFFFFFFFFFFFFFFFFFFFFF

@GWZHISEQ02:315:C9E6MANXX:5:1210:14367:69585

ATAAGGCTGAACAAAACTGGGACAGGGGCCAAACAGGATATCTGTGGTCGAGCACCTGGGCCCCGGCTCAGGGCCAAGAACAGATGGTACTCAGATAAAGCGAAACTAGCAGATCGGAAGAGCGTC

+

BBBBBFFFFFFBFBFFFFFFFFFFFFBFFFFFFFBFFFFFFFFFFFFFFFFFB<FFFFFFFFFFFFFFFFFFFFFFFFFFFFFBFFFFFFFFFFFFFFFFFFFFFFFFFFFFBBFFFFFF/7BFFB

@GWZHISEQ02:315:C9E6MANXX:5:1302:1495:66245

ATAAGGCTGAACAAAACTGGGACAGGGGCCAAACAGGATATCTGTGGTCGAGCACCTGGGCCCCGGCTCAGGGCCAAGAACAGATGGTACTCAGAGATCGGAAGAGCGTCGTGTAGGGAAAGAGTG

+

<BB/<BFFFFFBFF/<FFFFFFFFFFFFFFFFFFFFFFFFFFF/<FFFBFFFFFFFFB/FF<<<FBFFF<BF/BFFF7<<//FFFFFFFFFFFFFFFBBFFFFFF<7BB/BB</7BFFF<7/7///

@GWZHISEQ02:315:C9E6MANXX:5:1210:14367:69585

GCTAGTTTCGCTTTATCTGAGTACCATCTGTTCTTGGCCCTGAGCCGGGGCCCAGGTGCTCGACCACAGATATCCTGTTTGGCCCCTGTCCCAGTTTTGTTCAGCCTTATAGATCGGAAGAGCACA

+

/BBBBFFFFFFFFFFFFFFFFFFFFFFFFFFFFFFFFFFFFFFFBFFF<FFFFFFFFFFFFFFFFFFFFBFFFFFFFFFFF/FFFFFFFFFFFFF/FFBFFFFFFFFBFFFFFFFFBBBFBFFFBF

@GWZHISEQ02:315:C9E6MANXX:5:1211:2114:96208

CTTTCCAGAAACTGTTGCTAGTTTCGCTTTATCTGAGTACCATCTGTTCTTGGCCCTGAGCCGGGGCCCAGGTGCTCGACCACAGATATCCTGTTTGGCCCCTGTCCCAGTTTTGTTCAGCCTTAT

+

BBBBBFFFFFFFFFFFFFFFFFFFFFFFFFFFFFFFFFFFFFFFFFFBFFFFBFFFFFFFFFFFFFFFFFFFFFFFFFFFFFFFFFFFFFFFFFFFBBBFFBFFFFFFFFFFFFFFFFFFFFFFFF

@GWZHISEQ02:315:C9E6MANXX:5:1302:1495:66245

CTGAGTACCATCTGTTCTTGGCCCTGAGCCGGGGCCCAGGTGCTCGACCACAGATATCCTGTTTGGCCCCTGTCCCAGTTTTGTTCAGCCTTATAGATCGGAAGAGCACACGTCTGAACTCCAGTC

+

BBB//<FFFBFFFFBFFBBBBF/FFFFBFFFFFFBFBBFFFFFFFFFFFFF///<FFFFF<BFFFFBFFFFFFFFBFFFFFFFF//<B//<FFFFFFFFFBFFBFF<<F//77FFF/FFFFF/77F

@GWZHISEQ02:315:C9E6MANXX:5:2109:1936:69565

CTTTCCAGAAACTGTTGCTAGTTTCGCTTTATCTGAGTACCATCTGTTCTTGGCCCTGAGCCGGGGCCCAGGTGCTCGACCACAGATATCCTGTTTGGCCCCTGTCCCAGTTTTGTTCAGCCTTAT

+

<BBBBFFBFFFFBFFFFFFFFFFFFFFFFFFFFFFFFBFFFFFFFFFFFFFFFFBFFF<FFBFFB<FFFFFFFFF<BFFFFFFBFFFFFFFFFFFF/BBBFFFFFFFFBFFFFFBFFFFFFFFFBF

@GWZHISEQ02:315:C9E6MANXX:5:2207:16891:47313

CTTTCCAGAAACTGTTGCTAGTTTCGCTTTATCTGAGTACCATCTGTTCTTGGCCCTGAGCCGGGGCCCAGGTGCTCGACCACAGATATCCTGTTTGGCCCCTGTCCCAGTTTTGTTCAGCCTTAT

+

BBBBBFFFFFFFFFFFFFFFFFFFFFFFFFFFFFFFFFFFFFFFFFFFFFFFFFFFFFFFFFFFFFFFFFFFFFFFFFFFFFFFFFFFFFFFFFFFFFFFFFFFFFFFFFFFFFFFFFFFFFFFFF

@GWZHISEQ02:315:C9E6MANXX:5:2309:15614:35542

CTTTCCAGAAACTGTTGCTAGTTTCGCTTTATCTGAGTACCATCTGTTCTTGGCCCTGAGCCGGGGCCCAGGTGCTCGACCACAGATATCCTGTTTGGCCCCTGTCCCAGTTTTGTTCAGCCTTAT

+

BBBBBFFFFFFFFFFFFFFFFFFFFFFFFFFFFFFFFFFFFFFFFFFFFFFFBFFFFFFFFFFFFFFFFFFFFFFBFBFFFFFFFFFFFFFFFFFFFBFFFFFFFFFFFFFFFFFBFFFFFFFFBF

@GWZHISEQ02:315:C9E6MANXX:5:2315:19202:9020

CTTTCCAGAAACTGTTGCTAGTTTCGCTTTATCTGAGTACCATCTGTTCTTGGCCCTGAGCCGGGGCCCAGGTGCTCGACCACAGATATCCTGTTTGGCCCCTGTCCCAGTTTTGTTCAGCCTTAT

+

BBBBBFFFFFFFFFFBFFFBFFFFFFFFFF/FFFF/FFFFFFFFFFFFFFFFBFFFFF<FFFFFFFFFBFFFFFFFFFBFFFFFF/FBFFFFFFFFFFFFFFFFBFFFBFF<BFBFFFFFFFFFFF

@GWZHISEQ02:315:C9E6MANXX:5:2315:9664:23113

CTTTCCAGAAACTGTTGCTAGTTTCGCTTTATCTGAGTACCATCTGTTCTTGGCCCTGAGCCGGGGCCCAGGTGCTCGACCACAGATATCCTGTTTGGCCCCTGTCCCAGTTTTGTTCAGCCTTAT

+

BBBBBFFFFFFFFFFFFFFFFFFFFFFFFFFFFFFFFFFFFFFFFFFFFFFFFFFFFFFFFFFFFFFFFFFFFFFFFFFFFFFFFFFFFFFFFFFF/BFFFFFFFFFFFFFFFFFFFFFFFFFFFF

@GWZHISEQ02:315:C9E6MANXX:5:2105:16844:98102

GTCTTTCCAGAAACTGTTGCTAGTTTCGCTTTATCTGAGTACCATCTGTTCTTGGCCCTGAGCCGGGGCCCAGGTGCTCGACCACAGATATCCTGTTTGGCCCCTGTCCCAGTTTTGTTCAGCCTT

+

/BBBBFFFFFFFFFFFFFFFFFFFFFFFFFFFFFFFFFFFFFFFFFFFFFFFFFFFFFFFFFFFFFFBFFFFFFFFFFFFBFFFFFFFFFFFFFFFFFFBFFFFFFFFFF<FFFFFFBFFBFFFFF

@GWZHISEQ02:315:C9E6MANXX:5:1208:4021:57449

GGACTTTCCAGAAACTGTTGCTAGTTTCGCTTTATCTGAGTACCATCTGTTCTTGGCCCTGAGCCGGGGCCCAGGTGCTCGACCACAGATATCCTGTTTGTCCCCTGTCCCAGTTTTGTTCAGCCT

+

BBBBBFF<FFFBFFFFFFF/FFFFFFFFFFFFFFFFFFBFFFFB//FFFBFFFFFFFBF<F/<BBFFF</FB///</</<F/<BF<//<FFB//<BFFFF//<FBF/7//<//B<FF/<7/BBFB7

@GWZHISEQ02:315:C9E6MANXX:5:2108:16773:35951

GGACTTTCCAGAAACTGTTGCTAGTTTCGCTTTATCTGAGTACCATCTGTTCTTGGCCCTGAGCCGGGGCCCAGGTGCTCGACCACAGATATCCTGTTTGGCCCCTGTCCCAGTTTTGTTCAGCCT

+

BBBBBFFFFFFFFFFFFFFFFFFFFFFFFFFFFFFFFFFFFFFFFFFFFFFFFFFFFFFFFFFFFFFFFFFFFFFFFFFFFFFFFFFFFFFFFFFFFFFFFFFFFFFFFFFFFFFFFFFFFFFFFF

@GWZHISEQ02:315:C9E6MANXX:5:2211:12723:23181

GGACTTTCCAGAAACTGTTGCTAGTTTCGCTTTATCTGAGTACCATCTGTTCTTGGCCCTGAGCCGGGGCCCAGGTGCTCGACCACAGATATCCTGTTTGGCCCCTGTCCCAGTTTTGTTCAGCCT

+

BBBBBFFFFFFFFFFFFFFFFFFFFFFFFFFFFFFFFFFFFFFFFFFFFFFFFFFFFFFFFFFFFFFBFFFFFFFFFFFFFFFFFFFFFFFFFFFFFFFFFFFFFFFFFFFFFFFFFFFFFFFFFB

@GWZHISEQ02:315:C9E6MANXX:5:2216:18882:14941

GGACTTTCCAGAAACTGTTGCTAGTTTCGCTTTATCTGAGTACCATCTGTTCTTGGCCCTGAGCCGGGGCCCAGGTGCTCGACCACAGATATCCTGTTTGGCCCCTGTCCCAGTTTTGTTCAGCCT

+

BBBBBFFFFFFFFFFFFFFFFFFFFFFFFFFFFFFFFFFFFFFFFFFFFFFFFFFFFFFFFFFFFFFFFFFFFFFFFFFFFFFFFFFFFFFFFFFBFFFFFFFFFFFFFFFFF77FFFF<FFFBFB

@GWZHISEQ02:315:C9E6MANXX:5:1107:1408:79724

GGCTGAACAAAACTGGGACAGGGGCCAAACAGGATATCTGTGGTCGAGCACCTGGGCCCCGGCTCAGGGCCAAGAACAGATGGTACTCAGATAAAGCGAAACTAGCAACAGTTTCTGGAAAGTCCC

+

BBBBBFFFFFFFFFFFFFFFFFFFFFFFFFFFFFFFFFFFFFFFFFFFFFFFFFFFFFFFFFFFFFFFFFFFFFFFFFFFFFFFFFFFFFFFFFFFFBFFFFF<FFFFFFF7BFFFFFFFFFFFFF

@GWZHISEQ02:315:C9E6MANXX:5:1114:20550:86973

GGCTGAACAAAACTGGGACAGGGGCCAAACAGGATATCTGTGGTCGAGCACCTGGGCTCCGGCTCAGGGCCAAGAACAGATGGTACTCAGATAAAGCGAAACTAGCAACAGTTTCTGGAAAGTCCC

+

BBBBBFFFFFFFFFFFFFFFFFFFFFFFFFFFFFFFFFFFFFFFFFFFFFFFFFF<FFFBBBFBBFFBFFFBFFFFFFFBFFFFFFFFFFFBFFFFFFFFFFFFFFFFFFFFBFFFFFBFFFFFFB

@GWZHISEQ02:315:C9E6MANXX:5:1116:20058:27010

GGCTGAACAAAACTGGGACAGGGGCCAAACAGGATATCTGTGGTCGAGCACCTGGGCCCCGGCTCAGGGCCAAGAACAGATGGTACTCAGATAAAGCGAAACTAGCAACAGTTTCTGGAAAGTCCC

+

BBBBBFFFFFFFFFFFFFFFFFFFFFFFFFFFFFFFFFFFFFFFFFFFFFFFFFFFFFFFFFFFFFFFFFFFFFFFFFFFFFFFFFFFFFFFFFFFFFFFFFFFFFFFFFFFFFFFFFFFFFFFFF

@GWZHISEQ02:315:C9E6MANXX:5:1206:20232:79051

GGCTGAACAAAACTGGGACAGGGGCCAAACAGGATATCTGTGGTCGAGCACCTGGGCCCCGGCTCAGGGCCAAGAACAGATGGTACTCAGATAAAGCGAAACTAGCAACAGTTTCTGGAAAGTCCC

+

BBBBBFFFFFFFFFFFFFFFFFFFFFFFFFFFFFFFFFFFFFFFFFFFFFFFFFFFFFFFFFFFFFFFFFFFFFFFFFFFFFFFFFFFFFFFFFFFFFFFFFFFFFFFFFFFFFFFFFFFFFFFFF

@GWZHISEQ02:315:C9E6MANXX:5:1213:3664:82190

GGCTGAACAAAACTGGGACAGGGGCCAAACAGGATATCTGTGGTCGAGCACCTGGGCCCCGGCTCAGGGCCAAGAACAGATGGTACTCAGATAAAGCGAAACTAGCAACAGTTTCTGGAAAGTCCC

+

BBBBBFFFFFFFFFFFBFFFFFFFFFFFFFFFFFFFFFFFFFFFFFFFFFFFFFFFFFFFFFFFFBFFFFFFFFFFFFFFFFFFFFFFFF<FFFFFFFFFFFFFFFFFFFF/7FFFFF/FFFFFFF

@GWZHISEQ02:315:C9E6MANXX:5:2104:9546:96084

GGCTGAACAAAACTGGGACAGGGGCCAAACAGGATATCTGTGGTCGAGCACCTGGGCCCCGGCTCAGGGCCAAGAACAGATGGTACTCAGATAAAGCGAAACTAGCAGATCGGAAGAGCGTCGTGT

+

BBBBBFFFFFFFFFFFFFFFFFFFFFFFFFFFFFFFFFFFFFFFFFFFFFFFFFFFFFFFFFFFFFFFFFFFFFFFFFFFFFFFFFFFFFFFFFFFFBFBFFFFFFFFFFFFFBFFFFFFBFFFF7

@GWZHISEQ02:315:C9E6MANXX:5:2106:16797:46760

GGCTGAACAAAACTGGGACAGGGGCCAAACAGGATATCTGTGGTCGAGCACCTGGGCCCCGGCTCAGGGCCAAGAACAGATGGTACTCAGATAAAGCGAAACTAGCAACAGTTTCTGGAAAGTCCC

+

BBBBBBFBFFF/FFFFFFFFFFF/<FFFFFFBFFFFFBF<FFFFFFF<B/BFFFFF<FBBFBFFFFFFFFFFFFFFFFF</FFFFFFFFF<FFFFFFFBFFFFFFFFFF<F7BF<FFFFFFFFF/B

@GWZHISEQ02:315:C9E6MANXX:5:2109:5403:68958

GGCTGAACAAAACTGGGACAGGGGCCAAACAGGATATCTGTGGTCGAGCACCTGGGCCCCGGCTCAGGGCCAAGAACAGATGGTACTCAGATAAAGCGAAACTAGCAACAGTTTCTGGAAAGTCCC

+

BBBBBBFFFBFBFFFFFFFFFFFFFFFFFFFFFFFFFFFFFFFFFFFFFFFFFFFFFFFFFBFBFFBFFFF<FF7FBFFFFFBFBFBFFF<BFFFFFFFFFFFFFF<FF/FF/FFFF//7FFFFFF

@GWZHISEQ02:315:C9E6MANXX:5:2112:12317:76027

GGCTGAACAAAACTGGGACAGGGGCCAAACAGGATATCTGTGGTCGAGCACCTGGGCCCCGGCTCAGGGCCAAGAACAGATGGTACTCAGATAAAGCGAAACTAGCAACAGTTTCTGGAAAGTCCC

+

BBBBBFFFFFFFFFFFFFFFFFF<FFFFFFFFFFFFFFFFFFFFFFFFFFFFFFFFFFFFFFFFFFFFFFFFFFFFFFFFBFFFFFFFFFFFFFFFFFFFFFFFFFFFFFFFFFFFFFFFFFFFBF

@GWZHISEQ02:315:C9E6MANXX:5:2207:7520:33100

GGCTGAACAAAACTGGGACAGGGGCCAAACAGGATATCTGTGGTCGAGCACCTGGGCCCCGGCTCAGGGCCAAGAACAGATGGTACTCAGATAAAGCGAAACTAGCAACAGTTTCTGGAAAGTCCC

+

BBBBBFFFFFFFFFFFFFFFFFFFFFFFFFFFFFFFFFFFFFFFFFFFFFFFFFFFFFFFFBFFFFFFFFFFFFFFFFFBFFFFFFFFFFFFFFFFFFFFFFFFFFFFFFFFFFFFFFFFFFFFFF

@GWZHISEQ02:315:C9E6MANXX:5:2308:15628:40060

GGCTGAACAAAACTGGGACAGGGGCCAAACAGGATATCTGTGGTCGAGCACCTGGGCCCCGGCTCAGGGCCAAGAACAGATGGTACTCAGATAAAGCGAAACTAGCAAGATCGGAAGAGCGTCGTG

+

BBBBBFFFFFFFFFFFFFFFFFFFFFFFFFFFFFFFFFFFFFFFFFFFFFFFFFFFFFFFFFFFFFFFFFFFFFFFFFFFFFFFFFFFFFFBFFFFFFBFFFFFFFFFFFFFFFFFFFFFFBFF/B

@GWZHISEQ02:315:C9E6MANXX:5:1108:14681:20551

GGGACTTTCCAGAAACTGTTGCTAGTTTCGCTTTATCTGAGTACCATCTGTTCTTGGCCCTGAGCCGGGGCCCAGGTGCTCGACCACAGATATCCTGTTTGGCCCCTGTCCCAGTTTTGTTCAGCC

+

BBBBBFFFFFFFFFFFFFFFFFFFFFFFFFFFFFFFFFFFFFFFFFFFFFFFFFFFFFFFFFFFFFFFFFFFFFFFFFFFFFFFFFF<FFFFFFFFFFFFFFFFFFFFFFFFFFFFFFFFFFFFFF

@GWZHISEQ02:315:C9E6MANXX:5:1211:16689:18532

CTTGGCCCTGAGCCGGGGCCCAGGTGCTCGACCACAGATATCCTGTTTGGCCCCTGTCCCAGTGTTGTTGAGCCATTTCCAAAAGAGAATGGTCTTGGTGGATCTTGCGAGCTCGGACTGGAACAA

+

BBB<BFFFFFFFFFFFFFFFFFFFFFFFFFFFFFFFFBBFFFFFFFF<F7FFB/<</<F///B/<<<FF/BF///////B////////<////<////////<//<//<///</7///////7///

@GWZHISEQ02:315:C9E6MANXX:5:1211:13096:21160

GGGACTTTCCAGAAACTGTTGCTAGTTTCGCTTTATCTGAGTACCATCTGTTCTTGGCCCTGAGCCGGGGCCCAGGTGCTCGACCACAGATATCCTGTTTGGCCCCTGTCCCAGTTTTGTTCAGCC

+

BBBBBFFFFFFFFFFFFFFFFFFFFFFFFFFFFFFFFFFFFFFFFFFFFFFFFFFFFFFFFFFFFFFFFFFFFFFFFFFFFFFFF//<F/BB/FF/7BFFFBFFFFFFFFFFBBBFFF<FFF//B/

@GWZHISEQ02:315:C9E6MANXX:5:2104:9546:96084

GCTAGTTTCGCTTTATCTGAGTACCATCTGTTCTTGGCCCTGAGCCGGGGCCCAGGTGCTCGACCACAGATATCCTGTTTGGCCCCTGTCCCAGTTTTGTTCAGCCAGATCGGAAGAGCACACGTC

+

BBBBBFFFFFFFFFFFFFFFFFFFFFFFFFFFFFFFFFFFFFFFFFFFFFFFFFFFFFFFFFFFFFFFFBFFFFFFFFFFFFFFFFFFFFFFFFFFFFFFFFFFFFFFFFFFFBFFFFFFFFFFFF

@GWZHISEQ02:315:C9E6MANXX:5:2211:8591:66546

GGGACTTTCCAGAAACTGTTGCTAGTTTCGCTTTATCTGAGTACCATCTGTTCTTGGCCCTGAGCCGGGGCCCAGGTGCTCGACCACAGATATCCTGTTTGGCCCCTGTCCCAGTTTTGTTCAGCC

+

BBBBBFFFFFFFFFFFFFFFFFFFFFFFFFFFFFFFFFFFFFFFFFFFFFFFFFFFFFFFFFFFFFFFFFFFFFFFFFFFFFFFFFFFFFFFFFFFFFFFFFFFFFFFFFFFFFFFFFFFFFFFFF

@GWZHISEQ02:315:C9E6MANXX:5:2306:15614:22511

GGGACTTTCCAGAAACTGTTGCTAGTTTCGCTTTATCTGAGTACCATCTGTTCTTGGCCCTGAGCCGGGGCCCAGGTGCTTGACCACAGATATCCTGTTTGGCCCCTGTCCCAGTTTTGTTCAGCC

+

/BBBBB<F//FFF//BF/BB//</<F////FFBFBFFBFFFB/BF/<<F<</<FFF/BFFFF<///////<B<<FFFBFF/<FB/<FBB/7<FF//<<FFF/BF/7//7<BFB/7FBF/BBFBFBF

@GWZHISEQ02:315:C9E6MANXX:5:2308:15628:40060

TGCTAGTTTCGCTTTATCTGAGTACCATCTGTTCTTGGCCCTGAGCCGGGGCCCAGGTGCTCGACCACAGATATCCTGTTTGGCCCCTGTCCCAGTTTTGTTCAGCCAGATCGGAAGAGCACACGT

+

BBBBBFFFFFFFFFFFFFFFFFFFFFFFFFFFFFFFFFFFFFFFFFFFFFFFFFFFFBFFFFFFFFFFFFFFFFFFFFFFFFFFFFFFFFFFFFFFFFFFFFFFFFFFFFFFFB<BFBFFFFFFFF

@GWZHISEQ02:315:C9E6MANXX:5:2311:11799:57206

GGGACTTTCCAGAAACTGTTGCTAGTTTCGCTTTATCTGAGTACCATCTGTTCTTGGCCCTGAGCCGGGGCCCAGGTGCTCGACCACAGATATCCTGTTTGGCCCCTGTCCCAGTTTTGTTCAGCC

+

BBBBBFFFFFFFFFFFFFFFFFFFFFFFFFFFFFFFFFFFFFFFFFFFFFFFFFFFFFFFFFFFFFFFFFFFFFFFFFFFFFFFFFFFFFFFFFFFFFFFFFFFFFFFFFFFFFFFFFFFFFFFFF

@GWZHISEQ02:315:C9E6MANXX:5:2315:9001:72303

GGGACTTTCCAGAAACTGTTGCTAGTTTCGCTTTATCTGAGTACCATCTGTTCTTGGCCCTGAGCCGGGGCCCAGGTGCTCGACCACAGATATCCTGTTTGGCCCCTGTCCCAGTTTTGTTCAGCC

+

BBBBBFFFFFFFFFFFFFFFFFFFFFFFFFFFFFFFFFFFFFFFFFFFFFFFFFFFFFFFFFFFFFFFBBFFFFFFFFFFFFFFFFFFFFFFFFFFFFFFFFFFFB<FFFFFBFBFFFFFF<BFFF

@GWZHISEQ02:315:C9E6MANXX:5:1204:15600:7609

GCTGAACAAAACTGGGACAGGGGCCAAACAGGATATCTGTGGTCGAGCACCTGGGCCCCGGCTCAGGGCCAAGAACAGATGGTACTCAGATAAAGCGAAACTAGCAACAGTTTCTGGAAAGTCCCA

+

BBBBBFFFFFFFFFFFFFFFFFFFFFFFFFFFFFFFFFFFFFFFFFFFFFFFFFFFFFFFFFFFFFFFFFFFFFFFFFFFFFFFFFFFFFFFFFFFFFFFFFFFFFFFFFFFFFFFFFFFFFFFFF

@GWZHISEQ02:315:C9E6MANXX:5:1204:15301:77383

GCTGAACAAAACTGGGACAGGGGCCAAACAGGATATCTGTGGTCGAGCACCTGGGCCCCGGCTCAGGGCCAAGAACAGATGGTACTCAGATAAAGCGAAACTAGCAACAGTTTCTGGAAAGTCCCA

+

BBBBBFFFFFFFFFFFFFFFFFFFFFFFFFFFFFFFFFFFFFFFFFFFFFFFFFFFFFFFFFFFFFFFFFFFFFFFFFFFFFFFFFFFFFFFFFFFFFFFFFFFFFFFFFFFFFFFFFFFFFFFF<

@GWZHISEQ02:315:C9E6MANXX:5:1313:9767:69879

GCTGAACAAAACTGGGACAGGGGCCAAACAGGATATCTGTGGTCGAGCACCTGGGCCCCGGCTCAGGGCCAAGAACAGATGGTACTCAGATAAAGCGAAACTAGCAACAGTTTCTGGAAAGTCCAG

+

BBBBBFFFBFFFFFFFFFFFFFFFBFFFFFFFFFFFFFFFFFFFFFFFFFFFFFFFFFFFFF<BBFFFFFFFFFFFFBBFFFBBFFFFFBBFFFFFF<FFFF/7FFBFBBBBF<<BFF/FFF/7FF

@GWZHISEQ02:315:C9E6MANXX:5:2307:15132:56651

GCTGAACAAAACTGGGACAGGGGCCAAACAGGATATCTGTGGTCGAGCACCTGGGCCCCGGCTCAGGGCCAAGAACAGATGGTACTCAGATAAAGCGAAACTAGCAACAGTTTCTGGAAAGTCCCA

+

<<BBBFFFFFFFFBF<FFFFFBFBF<FFFFFFFFFFFFFFB/FFFF<F<FFF<//FFFBFFFFFFFFFFFFFFFFFFFFFFF7BFFFFFFFFFFBBBFFFFFFFFFFFFFFFFFBFFFFFFFFBFF

@GWZHISEQ02:315:C9E6MANXX:5:2309:12165:87666

GCTGAACAAAACTGGGACAGGGGCCAAACAGGATATCTGTGGTCGAGCACCTGGGCCCCGGCTCAGGGCCAAGAACAGATGGTACTCAGATAAAGCGAAACTAGCAACAGTTTCTGGAAAGTCCCA

+

BBBB<FFFFFFFFFFFFFFBFB<FF<FFFFFFFFFFFFFFFFFFFBFFFFFFBFFFFFFFFFFFFFFFBFFFFFFFFFFFFFFFFFBFFFFFFFFFFF<FFFFFFBFFFFFFFFFFFFFFFFFFFF

@GWZHISEQ02:315:C9E6MANXX:5:1104:19371:4959

TGGGACTTTCCAGAAACTGTTGCTAGTTTCGCTTTATCTGAGTACCATCTGTTCTTGGCCCTGAGCCGGGGCCCAGGTGCTCGACCACAGATATCCTGTTTGGCCCCTGTCCCAGTTTTGTTCAGC

+

BBBBBFFFFFFFFFFFFFFFFFFFFFFFFFFFFFFFFFFFFFFFFFFFFFFFFFFFFFFFFFFFFFFFFFFFFFFFFFFFFFFFFFFFFFFFFFFFFFFFFFFFFFFFFFFFFFFFFFFFFFFF<F

@GWZHISEQ02:315:C9E6MANXX:5:1214:12157:47539

TGGGACTTTCCAGAAACTGTTGCTAGTTTCGCTTTATCTGAGTACCATCTGTTCTTGGCCCTGAGCCGGGGCCCAGGTGCTCGACCACAGATATCCTGTTTGGCCCCTGTCCCAGTTTTGTTCAGC

+

BBBBBFFFFFFFFFFFFFFFFFFFFFFFFFFFFF<FFFFFFFFFFFFFFFFFFFFFFFFFFFFFFFFFFFFFFFFFFFFFFFFFFFFFFFFFFFFFFFFFFFFFFFFFFFFFFFFFFFFFFFFFFF

@GWZHISEQ02:315:C9E6MANXX:5:1313:9767:69879

GGACTTTCCAGAAACTGTTGCTAGTTTCGCTTTATCTGAGTACCATCTGTTCTTGGCCCTGAGCCGGGGCCCAGGTGCTCGACCACAGATATCCTGTTTGGCCCCTGTCCCAGTTTTGTTCAGCAG

+

BBBBBBFFFB<FFF<FFFFFFFFFFFFFFF<FFFFBBF/<FFFBFFFFB/FFBF<FF<FFFBBBFF</BFBBBFBFFFFBFFFBFB<<FBBF/FFBFFF////<FFF<FFFFF/FBFFFBBFFFFF

@GWZHISEQ02:315:C9E6MANXX:5:2204:19857:46791

CTGAACAAAACTGGGACAGGGGCCAAACAGGATATCTGTGGTCGAGCACCTGGGCCCCGGCTCAGGGCCAAGAACAGATGGTACTCAGATAAAGCGAAACTAGCAACAGTTTCTGGAAAGTCCCAC

+

BBBBBFFFFFFFFFFFFFFFFFFFFFFFFFFFFFFFFFFFFFFFFFFFFFFFFFFFFFFFFFFFFFFFFFFFFFFFFFFFFFFFFFFFFFFFFFFFFFFFFFFFFFFFFFFFFFFFFFFFFFFFFF

@GWZHISEQ02:315:C9E6MANXX:5:1103:15717:41997

GTGGGACTTTCCAGAAACTGTTGCTAGTTTCGCTTTATCTGAGTACCATCTGTTCTTGGCCCTGAGCCGGGGCCCAGGTGCTCGACCACAGATATCCTGTTTGGCCCCTGTCCCAGTTTTGTTCAG

+

BBBBBFFFFFFFFFFFFFFFFFFFFBFFFFFFFFFFFFFFFFFFFFFFFFFFFFFFFF<FFFFFFFFFFFFFFFFFFFFFFFFFFFFFFFFFFFFFFFFFFFFFFFFFFFFFFFFFFFFFFFFFFF

@GWZHISEQ02:315:C9E6MANXX:5:1104:1261:8340

GTGGGACTTTCCAGAAACTGTTGCTAGTTTCGCTTTATCTGAGTACCATCTGTTCTTGGCCCTGAGCCGGGGCCCAGGTGCTCGACCACAGATATCCTGTTTNGCCCCTGTCNNNGNNTTGTTCAG

+

<<<BB<BFFF<FFFFFFFFFFFF/<<F///<7//<<<FFBFFFFFF<BFFBF<FBFFBFF/<<FBFFBB77/</F/BFFF///<F</FB/B/FFF//<B<</#<<77BFBBF###7##77777/7B

@GWZHISEQ02:315:C9E6MANXX:5:1108:10353:37217

GTGGGACTTTCCAGAAACTGTTGCTAGTTTCGCTTTATCTGAGTACCATCTGTTCTTGGCCCTGAGCCGGGGCCCAGGTGCTCGACCACAGATATCCTGTTTGGCCCCTGTCCCAGTTTTGTTCAG

+

BBBBBFFFFFFFFFFFFFFFFFFFFFFFFFFFFFFFBFFFFFFFFFFFFFFFFFFFFFFFFFFFFFFFFFFFFFFFFFFFFFFFFFFFFBFFFFFFFFFF<FFFFFFFFFFFFFBFFFFFFFFFFF

@GWZHISEQ02:315:C9E6MANXX:5:1205:12991:67100

GTGGGACTTTCCAGAAACTGTTGCTAGTTTCGCTTTATCTGAGTACCATCTGTTCTTGGCCCTGAGCCGGGGCCCAGGTGCTCGACCACAGATATCCTGTTTGGCCCCTGTCCCAGTTTTGTTCAG

+

B<<BBFFFFFFFFFFFFFFFFFFFFFFFFFFFFFFFFFFFFFFFFFFF<FFFFFFFFBFFFFFFFFFFFFFFFFFFFFFBFFFFFFFFFBFFFFFFFFFFFFFFFFBFFFFFFFFFFFFFFFBFFF

@GWZHISEQ02:315:C9E6MANXX:5:1206:20232:79051

GTGGGACTTTCCAGAAACTGTTGCTAGTTTCGCTTTATCTGAGTACCATCTGTTCTTGGCCCTGAGCCGGGGCCCAGGTGCTCGACCACAGATATCCTGTTTGGCCCCTGTCCCAGTTTTGTTCAG

+

BBBBBFFFFFFFFFFFFFFFFFFFFFFFFFFFFFFFFFFFFFFFFFFFFFFFFFFFFFFFFFFFFFFFFFFFFFFFFFFFFFFFFFFFFFFFFFFFFFFFFFFFFFFFFFFBFFFFFBFFFFFFFF

@GWZHISEQ02:315:C9E6MANXX:5:1207:10791:79950

GTGGGACTTTCCAGAAACTGTTGCTAGTTTCGCTTTATCTGAGTACCATCTGTTCTTGGCCCTGAGCCGGGGCCCAGGTGCTCGACCACAGATATCCTGTTTGGCCCCTGTCCCAGTTTTGTTCAG

+

BBBBBFFFFFFFFFFFFFFFFFFFFFFFFFFFFFFFFFFFFFFFFFFFFFFFFFFFFFFFFFFFFFFFFFFFFFFFFFFFFFFFFFFFFFFFFFFFFFFFFFFFFFFFFFFFFFFFFFFFFFFFFF

@GWZHISEQ02:315:C9E6MANXX:5:1214:13552:78170

GTGGGACTTTCCAGAAACTGTTGCTAGTTTCGCTTTATCTGAGTACCATCTGTTCTTGGCCCTGAGCCGGGGCCCAGGTGCTCGACCACAGATATCCTGTTTGGCCCCTGTCCCAGTTTTGTTCAG

+

BBBBBFFFFFFFFFFFFFFFFFFFFFFFFFFFFFFFFFFFFFFFFFFFFFFFFFFFFFFFFFFFFFFFFFFFFFFFFFFFFFFFFFFFFFFFFFFFFFFFFFFFFFFFFFFFFFFFFFFFFFFFFF

@GWZHISEQ02:315:C9E6MANXX:5:1305:3635:72311

GTGGGACTTTCCAGAAACTGTTGCTAGTTTCGCTTTATCTGAGTACCATCTGTTCTTGGCCCTGAGCCGGGGCCCAGGTGCTCGACCACAGATATCCTGTTTGGCCCCTGTCCCAGTTTTGTTCAG

+

BBBBBFFFFFFFFFFFFFFFFFFFFFFFFFFFFFFFFFFFFFFFFFFFFFFFFFFFFFFFFFFFFFFFFFFFFFFFFFFFFFFFFFFFFFFFFFFFFFFFFFFFFFFFFFFFFFFFFFFFFFFFFF

@GWZHISEQ02:315:C9E6MANXX:5:1307:19212:13659

GTGGGACTTTCCAGAAACTGTTGCTAGTTTCGCTTTATCTGAGTACCATCTGTTCTTGGCCCTGAGCCGGGGCCCAGGTGCTCGACCACAGATATCCTGTTTGGCCCCTGTCCCAGTTTTGTTCAG

+

BBBBBFFFFFFFFFFFFFFFFFFFFFFFFFFFFFFFFFFFFFFFFFFFFFFFFFFFFFFFFFFFFFFFFFFFFFFFFFFFFFFBFFFFFFFBFFFFFFFFFFFFFFFFFFFFFFFFFFFFFF/F77

@GWZHISEQ02:315:C9E6MANXX:5:1309:14924:15372

GTGGGACTTTCCAGAAACTGTTGCTAGTTTCGCTTTATCTGAGTACCATCTGTTCTTGGCCCTGAGCCGGGGCCCAGGTGCTCGACCACAGATATCCTGTTTGGCCCCTGTCCCAGTTTTGTTCAG

+

/BBB/FBFFBFFFFFFFFFFFFBFFFFFFFFBFFFFFFFFF/BFF<<<BBFF<FFFBFFFFFFFFFBF/FF///<FF</<<<<FFFFB/BB/<<BFFB/FFFB<BFBB<FF////<FBBFFFF7BB

@GWZHISEQ02:315:C9E6MANXX:5:1309:1712:83244

GTGGGACTTTCCAGAAACTGTTGCTAGTTTCGCTTTATCTGAGTACCATCTGTTCTTGGCCCTGAGCCGGGGCCCAGGTGCTCGACCACAGATATCCTGTTTGGCCCCTGTCCCAGTTTTGTTCAG

+

BBBBBFFFFFFFFFFFF<BFFFFFFFFFFF<BFB/FFFFFFFFFFFFFFFFFFBFBFFFFFFFFFFFBFFFFFBFFFFFFFFFFFFF<FFFFFFFFFFFFFFFBFFFFFFBB<F/B/FBBFFFFFF

@GWZHISEQ02:315:C9E6MANXX:5:1311:13567:66012

GTGGGACTTTCCAGAAACTGTTGCTAGTTTCGCTTTATCTGAGTACCATCTGTTCTTGGCCCTGAGCCGGGGCCCAGGTGCTCGACCACAGATATCCTGTTTGGCCCCTGTCCCAGTTTTGTTCAG

+

<BBBBFFFFFFFFFFFFFFFFFFFFFFFFFFBFFFFFFFFFFFFFFFFFFFFFFFFFFFFFFFFFFFFBFFBBBFFFFFBFFFFFFF<FFFFFBFFFFFFFBFBBBFFBBBFFF/<7BF<//FBF<

@GWZHISEQ02:315:C9E6MANXX:5:1312:11311:25703

GTGGGACTTTCCAGAAACTGTTGCTAGTTTCGCTTTATCTGAGTACCATCTGTTCTTGGCCCTGAGCCGGGGCCCAGGTGCTCGACCACAGATATCCTGTTTGGCCCCTGTCCCAGTTTTGTTCAG

+

BBBBBFFFFFFFFFFFFFFFFFFFFFFFFFFFFFFFFFFFFFFFFFFFFFFFFFFFFFFFFFFFFFFFFFFFFFFFFFFFFFFFFFFFFFFFFFFFFFFFFFFFFFFFFFFFFFFFFFFFFFFFFF

@GWZHISEQ02:315:C9E6MANXX:5:1314:14235:9666

GTGGGACTTTCCAGAAACTGTTGCTAGTTTCGCTTTATCTGAGTACCATCTGTTCTTGGCCCTGAGCCGGGGCCCAGGTGCTCGACCACAGATATCCTGTTTGGCCCCTGTCCCAGTTTTGTTCAG

+

BBBBBFFFFFFFFFFFFFFFFFFFFFFFFFFFFFFFFFFFFFFFFFFFFFFFFFFFFFFFFFFFFFFFFFFFFFFFFFFFFFFBFFFFFFFFFFFFFFFFFFFFFFFFFFFFFFFFFFFFFFFFFF

@GWZHISEQ02:315:C9E6MANXX:5:1316:6899:38926

GTGGGACTTTCCAGAAACTGTTGCTAGTTTCGCTTTATCTGAGTACCATCTGTTCTTGGCCCTGAGCCGGGGCCCAGGTGCTCGACCACAGATATCCTGTTTGGCCCCTGTCCCAGTTTTGTTCAG

+

BBBBBFFFFFFFFFFFFFFFFFFFFFFFFFFFFFFFFFFFFFFFFFFFFFFFFFFFFFFFFFFFFFFFFFFFFFFFFFFFFFFFFFFFFFFFFFFFFFFFFFFFFFFFFFFFFFFFFFFFFFFFFB

@GWZHISEQ02:315:C9E6MANXX:5:2110:8027:40438

GTGGGACTTTCCAGAAACTGTTGCTAGTTTCGCTTTATCTGAGTACCATCTGTTCTTGGCCCTGAGCCGGGGCCCAGGTGCTCGACCACAGATATCCTGTTTGGCCCCTGTCCCAGTTTTGTTCAG

+

BBBBBFFFFFFFFFFFFFFFFFFFFFFFFFFFFFFFFFFFFFFFFFFFFFFFFFFFFFFFFFFFFFFFFFFFFFFFFFFFFFFFFFFFFFFFFFFFFFFFFFFFFFFFFFFFFFFFFFFFFFFFFF

@GWZHISEQ02:315:C9E6MANXX:5:2208:19567:92842

GTGGGACTTTCCAGAAACTGTTGCTAGTTTCGCTTTATCTGAGTACCATCTGTTCTTGGCCCTGAGCCGGGGCCCAGGTGCTCGACCACAGATATCCTGTTTGGCCCCTGTCCCAGTTTTGTTCAG

+

BBBBBFFFFFFFFFFFFFFFFFFFFFFFFFFFFFFFFFFFFFFFFFFFFFFFFFFFFFFFFFBFBFFFFFFFFFFFFFFFFFFFFFFFFFFFFFFFFFFFFFF<FFFFFFFFFFF<77FFFBFFFB

@GWZHISEQ02:315:C9E6MANXX:5:2212:2082:30005

GTGGGACTTTCCAGAAACTGTTGCTAGTTTCGCTTTATCTGAGTACCATCTGTTCTTGGCCCTGAGCCGGGGCCCAGGTGCTCGACCACAGATATCCTGTTTGGCCCCTGTCCCAGTTTTGTTCAG

+

BBBBBFFFFFFFFFFFFFFFFFFFFFFFFFFFFFFFFFFFFFFFFFFFFFFFFFFFFFFFFFFFFFFFFFFFFFFFFFFFFFFFFFFFFFFFFFFFFFFFFFFFFFFFFFFFFFFFF<FFBFFFFF

@GWZHISEQ02:315:C9E6MANXX:5:2305:11302:34061

GTGGGACTTTCCAGAAACTGTTGCTAGTTTCGCTTTATCTGAGTACCATCTGTTCTTGGCCCTGAGCCGGGGCCCAGGTGCTCGACCACAGATATCCTGTTTGGCCCCTGTCCCAGTTTTGTTCAG

+

BBBBBFFFFFFFFFFFFFFFFFFFFFFFFFFFFFFFFFFFFFFFFFFFFFFFFFFFFFFFFFFFFFFFFFFFFFFFFFFFFFFFFFFFFFFFFFFFFFFFFFFFFFFFFFFFFFFFFFFFFFFFFB

@GWZHISEQ02:315:C9E6MANXX:5:2307:21320:66058

GTGGGACTTTCCAGAAACTGTTGCTAGTTTCGCTTTATCTGAGTACCATCTGTTCTTGGCCCTGAGCCGGGGCCCAGGTGCTCGACCACAGATATCCTGTTTGGCCCCTGTCCCAGTTTTGTTCAG

+

B<BB<FFBFFBBFFFFFFFFFFF/FFFFFF/FBBFFFFFFBFFBFFFBF//FFFFFFFFFFFFFFFBFFFFB/BFFFFFFFF/FFB/F/FBFFFF/<F7FFFFF/FF<FFBF/</<7BBFFBB/BF

@GWZHISEQ02:315:C9E6MANXX:5:2312:7790:62311

GTGGGACTTTCCAGAAACTGTTGCTAGTTTCGCTTTATCTGAGTACCATCTGTTCTTGGCCCTGAGCCGGGGCCCAGGTGCTCGACCACAGATATCCTGTTTGGCCCCTGTCCCAGTTTTGTTCAG

+

BBBBBFFFFFFFFFFFFFFFFFFFFFFFFFFFFFFFFFFFFFFFFFFFFFFFFFFFFFFFFFFFFFFFFFFFFFFFFFFFFFFFFFFFFFFFFFFFFFFFFFFFFFFBFFFFFFFFFFFFFFFFFB

@GWZHISEQ02:315:C9E6MANXX:5:1204:20407:95267

TGAACAAAACTGGGACAGGGGCCAAACAGGATATCTGTGGTCGAGCACCTGGGCCCCGGCTCAGGGCCAAGAACAGATGGTACTCAGATAAAGCGAAACTAGCAACAGTTTCTGGAAAGTCCCACC

+

BBBBBFFFFFFFFFFFFFFFFFFFFFFFFFFFFFFFFFFFFFFFFFFFFFFFFFFFFFFFFFFFFFFFFFFFFFFFFFFFBFFFFFFFFFFFFFFFBFFFFFFFFFFFBFF<FFFFFFFFFFFFFF

@GWZHISEQ02:315:C9E6MANXX:5:1103:4577:59282

AACAAAACTGGGACAGGGGCCAAACAGGATATCTGTGGTCGAGCACCTGGGCCCCGGCTCAGGGCCAAGAACAGATGGTACTCAGATAAAGCGAAACTAGCAACAGTTTCTGGAAAGTCCCACCTC

+

BBBBBFFFFFFFFFFFFFFFFFFFFFFFFFFFFFFFFFFFFFFFFFFFFFFFFFFFFFFFFFFFFFFFFFFFFFFFFFFFFFFFFFFFFFFFFFFFFFFFFFFFFFFFFFFFFFFFFFFFFFFFFF

@GWZHISEQ02:315:C9E6MANXX:5:1204:7759:33176

ACAAAACTGGGACAGGGGCCAAACAGGATATCTGTGGTCGAGCACCTGGGCCCCGGCTCAGGGCCAAGAACAGATGGTACTCAGATAAAGCGAAACTAGCAACAGTTTCTGGAAAGTCCCCCCCCG

+

BBBBBFFFFFFFFFFFFFFFFFFFFFFFFFFFFFFFFFFFBFFFFFFFFFFFFFFFFFFFFFFFFFFFFFFFFBFFFFFFFFFFFFFFFFFFFFFFFFFFFBFFFFFFFFFFFFFF/FF7/7/7//

@GWZHISEQ02:315:C9E6MANXX:5:1209:2877:70477

ACAAAACTGGGACAGGGGCCAAACAGGATATCTGTGGTCGAGCACCTGGGCCCCGGCTCAGGGCCAAGAACAGATGGTACTCAGATAAAGCGAAACTAGCAACAGTTTCTGGAAAGTCCCACAGAT

+

BBBBBFFFBFBFFBFFFFFFFFFFFFFFFFFFFFFFFFFFFFFFFFFFFFFFFBFFBFFFFFFFFFFFFFFFFFFFFFFFFFFFBFFFFFFFFFFFFFFFFFFFFFFFFFFBFFBFFFFFFFFFFF

@GWZHISEQ02:315:C9E6MANXX:5:1214:19777:38170

ACAAAACTGGGACAGGGGCCAAACAGGATATCTGTGGTCGAGCACCTGGGCCCCGGCTCAGGGCCAAGAACAGATGGTACTCAGATAAAGCGAAACTAGCAACAGTTTCTGGAAAGTCCCACCTCA

+

B/<BBFFFF///BFFBFBF/FFFFF<FF/<BBFBFFFFFFF/B/F///FF/</7/<<FBBF<///7<BBFFFF//7/</7BFBFB/7/<FFFFFFBBFFF/BF//BBBFFFB/F/FF/BF<FFFFF

@GWZHISEQ02:315:C9E6MANXX:5:2108:16569:79553

ACAAAACTGGGACAGGGGCCAAACAGGATATCTGTGGTCGAGCACCTGGGCCCCGGCTCAGGGCCAAGAACAGATGGTACTCAGATAAAGCGAAACTAGCAACAGTTTCTGGAAAGTCCCACCTCA

+

BBBBBFFFFFFFFFFFFFFFFFFFFFF<FFFFFFFFFFFFFFFFFFFF/FBFBFBFFFBFFFBFFFBFFFF<FFFFFFBFBFFFFFFFFFFFBFFFFFFFFFFFF7BF<FFFFFFFFFB<BBBFF/

@GWZHISEQ02:315:C9E6MANXX:5:2111:19450:19648

ACAAAACTGGGACAGGGGCCAAACAGGATATCTGTGGTCGAGCACCTGGGCCCCGGCTCAGGGCCAAGAACAGATGGTACTCAGATAAAGCGAAACTAGCAACAGTTTCTGGAAAGTCCCACCTCA

+

/BBBBBFFF<<<FFFFFFFFFFFFFFFFFFFFFFFFFFFFBFFFFFFFBFBFFFFBFFFFFFFFFFFFFFFFFFFFFFFFFFFFFFFFFFFB<FFFFFFFFFFFFFFFFFFFFFFFFFFFFFFFFB

@GWZHISEQ02:315:C9E6MANXX:5:2304:4458:98602

ACAAAACTGGGACAGGGGCCAAACAGGATATCTGTGGTCGAGCACCTGGGCCCCGGCTCAGGGCCAAGAACAGATGGTACTCAGATAAAGCGAAACTAGCAACAGTTTCTGGAAAGTCCCACCTCA

+

BBBBBFFFFFFFFFFFFFFFFFFFFFFFFFFFFFFFFFFFFFFFFFFFFFFFFFFFFBFFFFFFFFFFFFFBFBFFFFFFFFFFFFFFFFFFFFFFFFFFFFFFFFFFFFFFFFBFFFFFFFFFF/

@GWZHISEQ02:315:C9E6MANXX:5:1209:2877:70477

GTGGGACTTTCCAGAAACTGTTGCTAGTTTCGCTTTATCTGAGTACCATCTGTTCTTGGCCCTGAGCCGGGGCCCAGGTGCTCGACCACAGATATCCTGTTTGGCCCCTGTCCCAGTTTTGTAGAT

+

<BB/<BFFFFFFFFFF<FBFFFFFFFFFFF/FBBBFFFFFFFFFFFBFFFFFFFFFFBB</<FBFFFFFFFFBFFFFFFFFBFBFFFFFFFFFFFFFFFFFFFFFFFFF<FFBBFFFBFFFFFFFF

@GWZHISEQ02:315:C9E6MANXX:5:1108:17597:97742

CAAAACTGGGACAGGGGCCAAACAGGATATCTGTGGTCGAGCACCTGGGCCCCGGCTCAGGGCCAAGAACAGATGGTACTCAGATAAAGCGAAACTAGCAACAGTTTCTGGAAAGTCCCACCTCAG

+

BBBBBFFFFFFFFFFFFFFFFFFFFFFFFFFFFFFFFFFFFFFFFFFFFFFFFFFFFFFFFFFFFFFFFFFFFFFFFFFFFFFFFFFFFFFFFFFFFFFFFFFFFFFFFFFFFFFFFFFFFFFFFF

@GWZHISEQ02:315:C9E6MANXX:5:1114:15769:13282

CAAAACTGGGACAGGGGCCAAACAGGATATCTGTGGTCGAGCACCTGGGCCCCGGCTCAGGGCCAAGAACAGATGGTACTCAGATAAAGCGAAACTAGCAACAGTTTCTGGAAAGTCCCACCTCAG

+

BBBBBFFFFFFFFFFFFFFFFFFFFFFFFFFFFFFFFFFFFFFFFFFFFFFFFFFFFFFFFFFFFFFFFFFFFFFFFFFFFFFFFFFFFFFFFFFFFFFFFFFFFFFFFFFFFFFFFFFFFFFFFF

@GWZHISEQ02:315:C9E6MANXX:5:1115:11161:59782

CAAAACTGGGACAGGGGCCAAACAGGATATCTGTGGTCGAGCACCTGGGCCCCGGCTCAGGGCCAAGAACAGATGGTACTCAGATAAAGCGAAACTAGCAACAGTTTCTGGAAAGTCCCACCTCAG

+

BBBBBFFFBBFFFFFFFFFFFFBFFFFFFFFFFFFFFFFFFFFFFFFFFFFFFFFFFFFFFFFFFFFFFFFFFFFFFFFFFFFFFFFFFFFFFFFFFFFFFFFF/<FFBF<BFFFFFFFF<FBFFF

@GWZHISEQ02:315:C9E6MANXX:5:1206:15835:37813

CAAAACTGGGACAGGGGCCAAACAGGATATCTGTGGTCGAGCACCTGGGCCCCGGCTCAGGGCCAAGAACAGATGGTACTCAGATAAAGCGAAACTAGCAACAGTTTCTGGAAAGTCCCACCTCAG

+

BBBBBFF<F<F<FFFBFFFFFFFFFFB/F</FBFFFFFFFF/FBB/<//<BFF/7<</FFFF/77BFFFFBF<<FF/7B7FFF/FFFFFFFF/B<FFFFF/FFF/BF7/FFFFFFB/BFBF//B7F

@GWZHISEQ02:315:C9E6MANXX:5:1306:3869:42987

CAAAACTGGGACAGGGGCCAAACAGGATATCTGTGGTCGAGCACCTGGGCCCCGGCTCAGGGCCAAGAACAGATGGTACTCAGATAAAGCGAAACTAGCAACAGTTTCTGGAAAGTCCCACCTCAG

+

BBBBBFFFFFFFFFFFFFFFFFFFFFFFFFFFFFFFFFFFFFFFFFFFFFFFFFFFFFFFFFFFFFFFFFFFFFFFFFFFFFFFFFFFFFFFFFFFFFFFFFFFFFFFFFFFFFFFFFFFFFFFFF

@GWZHISEQ02:315:C9E6MANXX:5:2112:15622:73943

CAAAACTGGGACAGGGGCCAAACAGGATATCTGTGGTCGAGCACCTGGGCCCCGGCTCAGGGCCAAGAACAGATGGTACTCAGATAAAGCGAAACTAGCAACAGTTTCTGGAAAGTCCCACCTCAG

+

BBBBBFFFFFFFFFFFFFFFFBFFFFFFFFFFFFFFFFFFFFFFFFFFFFFFFFFFFFFFFFFFFFFFFFFFFFFFFFFFFFFFFFFFFFFFFFFFFFFFFFFFFFFFFFFFBFFFFFFFFFFFFF

@GWZHISEQ02:315:C9E6MANXX:5:2114:7225:92847

CAAAACTGGGACAGGGGCCAAACAGGATATCTGTGGTCGAGCACCTGGGCCCCGGCTCAGGGCCAAGAACAGATGGTACTCAGATAAAGCGAAACTAGCAACAGTTTCTGGAAAGTCCCACCTCAG

+

BBBBBFFFFFFFFFFFFFFFFFFFFFFBFFFFFFFFFFFFFFFFFFFFFFFFFFFFFFFFFFFFFFFBFFFFFFFFFFFFFFFFFFFFFFFFFFFFFFFFFFFFFFFFFFFFFFFFFFFFFFFFFF

@GWZHISEQ02:315:C9E6MANXX:5:2203:13453:58925

CAAAACTGGGACAGGGGCCAAACAGGATATCTGTGGTCGAGCACCTGGGCCCCGGCTCAGGGCCAAGAACAGATGGTACTCAGATAAAGCGAAACTAGCAACAGTTTCTGGAAAGTCCCACCTCAG

+

/BBBBFFFFFFFFFF//<F/<FFFFFFFFFBFFFBBFF/7B<F/FFFFFFFFFFFBFFFBFFFFB/B<FFFFFFBFFFFFFBB/B7BBFFBBFFFFFFBFFFFFFBFFBB/7FFFF<FFFFBFFBF

@GWZHISEQ02:315:C9E6MANXX:5:2213:13398:6161

CAAAACTGGGACAGGGGCCAAACAGGATATCTGTGGTCGAGCACCTGGGCCCCGGCTCAGGGCCAAGAACAGATGGTACTCAGATAAAGCGAAACTAGCAACAGTTTCTGGAAAGTCCCACCTCAG

+

BBBBBFFFFFFFFFFFFFFFFFFFFFFFFFFFFFFFFFFFFFFFFFFFFFFFFFFFFFFFFFFFFFFFFFFFFFFFFFFFFFFFFFFFFFFFFFFFFFFFFFFFFFFFFFFFFFFFFFFFFFFFFF

@GWZHISEQ02:315:C9E6MANXX:5:2216:7147:41425

CAAAACTGGGACAGGGGCCAAACAGGATATCTGTGGTCGAGCACCTGGGCCCCGGCTCAGGGCCAAGAACAGATGGTACTCAGATAAAGCGAAACTAGCAACAGTTTCTGGAAAGTCCCACAGATC

+

BBBBBFFFFFFFFFFFFFFFFFFFFFFFFFFFFFFFFFFFFFFFFFFFFFFFFFFFFFFFFFFFFFFFFFFFF<FFFFFFFFFFFFFFFFFFFFFFFFFFFFFFFFFFFBFFFFFFFFFFFFFFFF

@GWZHISEQ02:315:C9E6MANXX:5:2307:1128:78535

CAAAACTGGGACAGGGGCCAAACAGGATATCTGTGGTCGAGCACCTGGGCCCCGGCTCAGGGCCAAGAACAGATGGTACTCAGATAAAGCGAAACTAGCAACAGTTTCTGGAAAGTCCCACCTCAG

+

BBBBBFFFFFFFFFFFFFFFFFFFFFFFFFFFFFFFFFFFFFFFFFFFFFFFFFFFFFFFFFFFFFFFFFFFFFFFFFFFFFFFFFFFFFFFFFFFFFFFFFFFBFFFFFFFFFFFFFFFFFFFFB

@GWZHISEQ02:315:C9E6MANXX:5:2312:17634:35491

CAAAACTGGGACAGGGGCCAAACAGGATATCTGTGGTCGAGCACCTGGGCCCCGGCTCAGGGCCAAGAACAGATGGTACTCAGATAAAGCGAAACTAGCAACAGTTTCTGGAAAGTCCCACCTCAG

+

BBBBBFFFFFFFFFFFFFFFFFFFFFFFFFFFFFFFFFFFFFFFFFFFFFFFFFFFFFFFFFFFFFFFFFFFBFFFFFFFFFFFFFFFFFFFBFFFFFFFFFFFFFFFFFFFFFFFFFFFFFFFFF

@GWZHISEQ02:315:C9E6MANXX:5:2314:4382:98224

CAAAACTGGGACAGGGGCCAAACAGGATATCTGTGGTCGAGCACCTGGGCCCCGGCTCAGGGCCAAGAACAGATGGTACTCAGATAAAGCGAAACTAGCAACAGTTTCTGGAAAGTCCCACCTCAG

+

BBBBBFFFFFFFFFBFFFFFFFFFFFFFFFFFFFFFFFFFFFFFFFFFFFFFFFFFFFFFFFFFFFFFFFFFFFFFFFFFFFFFFFFFFFFFFFFFFFFFFFFFBBFFFFFFFFFFFFFFFFFFFF

@GWZHISEQ02:315:C9E6MANXX:5:2213:10975:21543

CTGAGGTGGGACTTTCCAGAAACTGTTGCTAGTTTCGCTTTATCTGAGTACCATCTGTTCTTGGCCCTGAGCCGGGGCCCAGGTGCTCGACCACAGATATCCTGTTTGGCCCCTGTCCCAGTTTTG

+

BBBBBFFFFFFFFFFFFFFFFFFFFFFFFFFFFFFFFFFFFFFFFFFFFFFFFFFFFFFFFFFFFFFFFFFFFFFFFFFFFFFFFFFFFBFFFFFFFFFFFFFFFFFFFFFFFFFFFFFFFF<FFF

@GWZHISEQ02:315:C9E6MANXX:5:2216:7147:41425

GTGGGACTTTCCAGAAACTGTTGCTAGTTTCGCTTTATCTGAGTACCATCTGTTCTTGGCCCTGAGCCGGGGCCCAGGTGCTCGACCACAGATATCCTGTTTGGCCCCTGTCCCAGTTTTGAGATC

+

BBBBBFFFFFFFFFFFFFFFFFFFFFFFFFFFFFFFFFFFFFFFFFFFFFFFFFFFFFFFFFFFFFFFFFFFFFFFFFFFFFFFFFFFFFFFFFFFFFFFFFFFFFFFFFFFFFFFFFFFFFFFFF

@GWZHISEQ02:315:C9E6MANXX:5:2112:5479:45384

AAAACTGGGACAGGGGCCAAACAGGATATCTGTGGTCGAGCACCTGGGCCCCGGCTCAGGGCCAAGAACAGATGGTACTCAGATAAAGCGAAACTAGCAACAGTTTCTGGAAAGTCCCACCTCAGT

+

BBBBBFFFFFFFFFFFFFFFFFFFFFFFFFFFFFFFFFFFFFFFFFFFFFFFFFFFFFFFFFFFFFFFFFFFFFFFFFFFFFFFFFFFFFFFFFFFFFFFFFFFFFFFFFFFFFFFFFFFFFFFFB

@GWZHISEQ02:315:C9E6MANXX:5:2113:11164:58389

AAAACTGGGACAGGGGCCAAACAGGATATCTGTGGTCGAGCACCTGGGCCCCGGCTCAGGGCCAAGAACAGATGGTACTCAGATAAAGCGAAACTAGCAACAGTTTCTGGAAAGTCCCACCTCAGT

+

BBBBBFBFF/BFFFFBBFFFFFFFFFFFFBFFFFFFFFFFFFFFFFFBFFFFBFFFFFFFFBB<FFB<FFFFFFFFFBBFFB/B<FFFFFFFFFFBFFFFFFFFFF<FFFFFFFFFFFBFF7FFF/

@GWZHISEQ02:315:C9E6MANXX:5:2302:20404:7798

AAAACTGGGACAGGGGCCAAACAGGATATCTGTGGTCGAGCACCTGGGCCCCGGCTCAGGGCCAAGAACAGATGGTACTCAGATAAAGCGAAACTAGCAACAGTTTCTGGAAAGTCCCACCTCAGT

+

BBBBBFFBFFFFB/<<FFFFFFFFFFFFF<FFF<BFFFFFFFFFFFFFFFBFFFFFFFF<<FFFFFFFFFFFFFFFBBFFFFFFFFFFFB<B<FFFFFFBBFFBFFFFBBFFFFBFFFFBBF/BFF

@GWZHISEQ02:315:C9E6MANXX:5:2316:4967:54600

AAACTGGGACAGGGGCCAAACAGGATATCTGTGGTCGAGCACCTGGGCCCCGGCTCAGGGCCAAGAACAGATGGTACTCAGATAAAGCGAAACTAGCAACAGTTTCTGGAAAGTCCCACCTCAGTT

+

BBBBBFFFFFFFFFFFFFFFFFFFFFFFFFFFFFFFFFFFFFFFFFFFFFFFFFFFFFFFFFFFFFFFFFFFFFFFFFFFFFFFFFFFFFFFFFFFFFFFFFFFFFFFFFFFFFFFFFFFFFFFFF

@GWZHISEQ02:315:C9E6MANXX:5:2316:10084:68325

AACTGGGACAGGGGCCAAACAGGATATCTGTGGTCGAGCACCTGGGCCCCGGCTCAGGGCCAAGAACAGATGGTACTCAGATAAAGCGAAACTAGCAACAGTTTCTGGAAAGTCCCACCTCAGTTT

+

BBBBBFFFFFFFFFFFFFFFFFFFFFFFFFFFFFFFFFFFFFFFFFFFFFFFFFFFFFFFFFFFFFFFFFFFFFFFFFFFFFFFFFFFFFFFFFFFFFFFFFFFFFFFFFFFFFFFFFFFFFFFFF

@GWZHISEQ02:315:C9E6MANXX:5:2213:18766:56929

ACTGGGACAGGGGCCAAACAGGATATCTGTGGTCGAGCACCTGGGCCCCGGCTCAGGGCCAAGAACAGATGGTACTCAGATAAAGCGAAACTAGCAACAGTTTCTGGAAAGTCCCACCTCAGTTTC

+

BBBBBFFFFFFFFFFFFFFFFFFFFFFFFFFFFFFFFFFFFFFFFFFFFFFFFFFFFFFFFFFFFFFFFFFFFFFFFFFFFFFFFFFFFFFFFFFFFFFFFFFFFFFFFFFFFFFFFFFFFFFFFF

@GWZHISEQ02:315:C9E6MANXX:5:1109:10681:90856

CTGGGACAGGGGCCAAACAGGATATCTGTGGTCGAGCACCTGGGCCCCGGCTCAGGGCCAAGAACAGATGGTACTCAGATAAAGCGAAACTAGCAACAGTTTCTGGAAAGTCCCAAGATCGGAAGA

+

BBBBBFFFFFFFFFFFFFFFFFFFFFFFFFFFFFFFFFFFFFFFFFFFFFFFFFFFFFFFFFFFFFFFFFFFFFFFFFFFFFFFFFFFFFFFFFFFFFFFFFFFFFFFFFFFFFFFFFFFFFFFF/

@GWZHISEQ02:315:C9E6MANXX:5:1112:14419:88454

CTGGGACAGGGGCCAAACAGGATATCTGTGGTCGAGCACCTGGGCCCCGGCTCAGGGCCAAGAACAGATGGTACTCAGATAAAGCGAAACTAGCAACAGTTTCTGGAAAGTCCCACCTCAGTTTCA

+

BB/BBFBFFFFFFFFFFBFFBFFB<F/FFFBFF/</FBBBBF//FBFFFB7FFB<FBBBBFFFFFBFF<BFFFFFBFFFFFFF//7/</<FFBB/</FFF</<BFBB/BBFFFFFFFFF/777FFF

@GWZHISEQ02:315:C9E6MANXX:5:1202:12717:38672

CTGGGACAGGGGCCAAACAGGATATCTGTGGTCGAGCACCTGGGCCCCGGCTCAGGGCCAAGAACAGATGGTACTCAGATAAAGCGAAACTAGCAACAGTTTCTGGAAAGTCCCACCTCAGTTTCA

+

BBBBBFFFFFFFFFFFFFFFFFFFFFFFFFFFFFFFFFFFFFFFFFFFFFFFFFFFFFFFFFFFFFFFFFFFFFFFFFFFFFFFFFFFFFFFFFFFFFFFFFFFFFFFFFFFFFFFFFFFFBFFFF

@GWZHISEQ02:315:C9E6MANXX:5:1205:15264:72651

CTGGGACAGGGGCCAAACAGGATATCTGTGGTCGAGCACCTGGGCCCCGGCTCAGGGCCAAGAACAGATGGTACTCAGATAAAGCGAAACTAGCAACAGTTTCTGGAAAGTCCCACCTCAGTTTCA

+

BBBBBFFFFFFFFFFFFFFFFFFFFFFFFFFFFFFFFFFFFFFFFFFFFFFFFFFFFFFFFFFFFFFFFFFFFFFFFFFFFFFFFFBFFFFFFFFFFFFFFFFFFFFFFFFFFFFFFFFFFFFFFF

@GWZHISEQ02:315:C9E6MANXX:5:1208:18788:49528

CTGGGACAGGGGCCAAACAGGATATCTGTGGTCGAGCACCTGGGCCCCGGCTCAGGGCCAAGAACAGATGGTACTCAGATAAAGCGAAACTAGCAACAGTTTCTGGAAAGTCCCACCTCAGTTTCA

+

BBBBBFFFFFFFFFFFFFFBFBFFFFFFFFFFFFFFFFFFFF</FFF<FBBFFFFFFFFFFFF</BFF/<<FFFBFFFBFFFFFFFBBFFFBFF/7F/FFFBFF/<FBF/FFBFB/B<FFFFFFF/

@GWZHISEQ02:315:C9E6MANXX:5:1210:20123:67521

CTGGGACAGGGGCCAAACAGGATATCTGTGGTCGAGCACCTGGGCCCCGGCTCAGGGCCAAGAACAGATGGTACTCAGATAAAGCGAAACTAGCAACAGTTTCTGGAAAGTCCCACCTCAGTTTCA

+

BBBBBFFFFFFFFFFFFFFFFFFFFFFFFFFFFFFFFFFFFFFFFFFFFFFFFFFFFFFFFFFFFFFFFFFFFFFFFFFFFFFFFFFFFFFFFFFFFFFFFFFFFFFFFFFFFFFFFFFFFFFFFF

@GWZHISEQ02:315:C9E6MANXX:5:1212:18958:97124

CTGGGACAGGGGCCAAACAGGATATCTGTGGTCGAGCACCTGGGCCCCGGCTCAGGGCCAAGAACAGATGGTACTCAGATAAAGCGAAACTAGCAACAGTTTCTGGAAAGTCCCACCTCAGTTTCA

+

BBBBBFFFFBFFFFFFFFFFFFFFFFFFFFFFFFFFFFFFFFFFFFFFFFFFFFFFFFFFFFFFFFBFFFFFFFFFFFFBFFFFFFFFFFFFFFFFFFFFFFFFFFFFFFFFBFFFFFFFFF/FBF

@GWZHISEQ02:315:C9E6MANXX:5:1301:1600:73471

CTGGGACAGGGGCCAAACAGGATATCTGTGGTCGAGCACCTGGGCCCCGGCTCAGGGCCAAGAACAGATGGTACTCAGATAAAGCGAAACTAGCAACAGTTTCTGGAAAGTCCCACCTCAGTTTCA

+

BBBBBFFFFFFFFFFFFFFFFBFFFFFFFFFFFFFFFFFFFFFFFFFFFFFFFFFFFFFFFFFFFFBFBFF<BFFF//B<FFFFFFF77FFFFFFFFFF<BFFFFFFFFFFFFFFFFFFFF7B<F<

@GWZHISEQ02:315:C9E6MANXX:5:1306:11712:47504

CTGGGACAGGGGCCAAACAGGATATCTGTGGTCGAGCACCTGGGCCCCGGCTCAGGGCCAAGAACAGATGGTACTCAGATAAAGCGAAACTAGCAACAGTTTCTGGAAAGTCCCACCTCAGTTTCA

+

BBBBBFFFFFFFFFFFFFFFFFFFFFFFFFFFFFFFFFFFFFFFFFFFFFFFFFFFFFFFFFFFFFFFFFFFFFFFFFFFFFFFFFFFFFFFFFFFFFFFFFFFFFFFFFFFBFFFFFFFFFFFFF

@GWZHISEQ02:315:C9E6MANXX:5:1307:2898:6339

CTGGGACAGGGGCCAAACAGGATATCTGTGGTCGAGCACCTGGGCCCCGGCTCAGGGCCAAGAACAGATGGTACTCAGATAAAGCGAAACTAGCAACAGTTTCTGGAAAGTCCCACCTCAGTTTCA

+

BBBBBFFFFFFFFFFFFFFFFFFFFFFFFFFFFFFFFFFFFFFFFFFFFFFFFFFFFFFFFFFFFFFFFFFFFFFFFFFFFFFFFFFFFFFFFFFFFFFFFFFFFFFFFFFFFFFFFFFFFFFFFF

@GWZHISEQ02:315:C9E6MANXX:5:2101:18471:55813

CTGGGACAGGGGCCAAACAGGATATCTGTGGTCGAGCACCTGGGCCCCGGCTCAGGGCCAAGAACAGATGGTACTCAGATAAAGCGAAACTAGCAACAGTTTCTGGAAAGTCCCACCTCAGTTTCA

+

BBBBBFFFFFFFFFFFFFFFFFFFFFFFFFFFFFFFFFFFFFFFFFFFFFFFFFFFFFFFFFFFFFFFFFFFFFFFFFFFFFFFFFFFFFFFFFFFFFFFFFFFFFFFFFFFFFFFFFFFFFFFFF

@GWZHISEQ02:315:C9E6MANXX:5:2110:9124:85678

CTGGGACAGGGGCCAAACAGGATATCTGTGGTCGAGCACCTGGGCCCCGGCTCAGGGCCAAGAACAGATGGTACTCAGATAAAGCGAAACTAGCAACAGTTTCTGGAAAGTCCCACCTCAGTTTCA

+

BBBBBFFFFFFFFFFFFFFFFFFFFFFFFFFFFFFFFFFFFFFFFFFFFFFFFFFFFFFFFFFFFFFFFFFFFFFFFFFFFFFFBFFFBFFBF<FF/7FFF<FFFFFFFBFFBBBFF/7/FFFFFF

@GWZHISEQ02:315:C9E6MANXX:5:2113:5379:63999

CTGGGACAGGGGCCAAACAGGATATCTGTGGTCGAGCACCTGGGCCCCGGCTCAGGGCCAAGAACAGATGGTACTCAGATAAAGCGAAACTAGCAACAGTTTCTGGAAAGTCCCACCTCAGTTTCA

+

BBBBBFFFFFFFFFFFFFFFFFFFFFFFFFFFFFFFFFFFFFFFFFFFFFFFFFFFFFFFFFFFFFFFFFFFFFFFFFFFFFFFFFFFFFFFFFFFFFFBFFFFFFFFFFFFFFFFFFFFFFFFFF

@GWZHISEQ02:315:C9E6MANXX:5:2209:3654:92298

CTGGGACAGGGGCCAAACAGGATATCTGTGGTCGAGCACCTGGGCCCCGGCTCAGGGCCAAGAACAGATGGTACTCAGATAAAGCGAAACTAGCAACAGTTTCTGGAAAGTCCAGATCGGAAGAGC

+

BBBBBFFFFFFFFFFFFFFFFFFFFFFFFFFFFFFFFFFFFFFFFFFFFFFFFFFFFFFFFFFFFFFFFFFFFFFFFFFFFFFFFFFFFFFFFFFFFFFFFFFFFFFFFFFFFFFFFFFFFFFFFB

@GWZHISEQ02:315:C9E6MANXX:5:2305:15684:85793

CTGGGACAGGGGCCAAACAGGATATCTGTGGTCGAGCACCTGGGCCCCGGCTCAGGGCCAAGAACAGATGGTACTCAGATAAAGCGAAACTAGCAACAGTTTCTGGAAAGTCCCACCTCAGTTTCA

+

BBBBBFFFFFFFFFFFFFFFFFFFFFFFFFFFFFFFFFFFFFFFFFFFFFFFFFFFFFFFFFFFFFFFFFFFFFFFFFFFFFFFFFBFFFFFFFFFFFFFFFFFFFFFFFFFFFFFFFFFFBFFFF

@GWZHISEQ02:315:C9E6MANXX:5:2310:3467:29881

CTGGGACAGGGGCCAAACAGGATATCTGTGGTCGAGCACCTGGGCCCCGGCTCAGGGCCAAGAACAGATGGTACTCAGATAAAGCGAAACTAGCAACAGTTTCTGGAAAGTCCCACCTCAGTTCAA

+

BBBBBFBF<FFFFFBFFFBBBFFFFFFFFFFFFBFFF<FFFF/FF<BBBFFFFFBFFFFFFFFBFFFFFFFFFFFFFF<<FFFFFFF<BFFFFF<FFFFFBFBFFFFFFB7FBBFFBF<FB7BB/7

@GWZHISEQ02:315:C9E6MANXX:5:2315:8004:78607

CTGGGACAGGGGCCAAACAGGATATCTGTGGTCGAGCACCTGGGCCCCGGCTCAGGGCCAAGAACAGATGGTACTCAGATAAAGCGAAACTAGCAACAGTTTCTGGAAAGTCCCACCTCAGTTTCA

+

BBBBBFFFFFB<FFFFFFFFFFFBFFFFFFBFBFFFFBF/FFBF<FFFBFFFFFFFFFFFFFBFF<BFFFFFFFFFFFFFFFFFFFFFFFFFFFFFFFFFFFFF/BB77BFFFFFFFFFFFFFFFF

@GWZHISEQ02:315:C9E6MANXX:5:1109:10681:90856

TGGGACTTTCCAGAAACTGTTGCTAGTTTCGCTTTATCTGAGTACCATCTGTTCTTGGCCCTGAGCCGGGGCCCAGGTGCTCGACCACAGATATCCTGTTTGGCCCCTGTCCCAGAGATCGGAAGA

+

BBBBBFFFFFFFFFFFFFFFFFFFFFFFFFFFFFFFFFFFFFFFFFFFFFFFFFFFFFFFFFFFFFFFFFFFFFFFFFFFFFFFFFFFFFFFFFFFFFFFFFFFFFFFFFFFFFFFFFFFFBBFFB

@GWZHISEQ02:315:C9E6MANXX:5:1201:17157:21484

TTCGCTTTATCTGAGTACCATCTGTTCTTGGCCCTGAGCCGGGGCCCAGGTGCTCGACCACAGATATCCTGTTTGGCCCCTGTCCCAGAGATCGGAAGAGCACACGTCTGAACTCCAGTCACATTA

+

BBBBBFFFFFFFFFFFFFFFFFFFFFFFFFFFFFFFFFFFFFBFFFFFFFFFFFFFFFFFFFFFFFBFFFFFFFFBFFFFFFFFFFFFFFFFFFFFFFFFFFFFFF77B<FFF<FFFFFFFFFBFF

@GWZHISEQ02:315:C9E6MANXX:5:2209:3654:92298

GGACTTTCCAGAAACTGTTGCTAGTTTCGCTTTATCTGAGTACCATCTGTTCTTGGCCCTGAGCCGGGGCCCAGGTGCTCGACCACAGATATCCTGTTTGGCCCCTGTCCCAGAGATCGGAAGAGC

+

BBBBBFFFFFFFFFFFFFFFFFFFFFFFFFFFFFFFFFFFFFFFFFFFFFFFFFFFFFFFFFFFFFBFFFFFFFFFFFFFFFFFFFFFFFFFFFFFFFFFFFFFFFFFFFFFFFFFFFFFFFFFFF

@GWZHISEQ02:315:C9E6MANXX:5:1305:10902:27407

TGGGACAGGGGCCAAACAGGATATCTGTGGTCGAGCACCTGGGCCCCGGCTCAGGGCCAAGAACAGATGGTACTCAGATAGAGCGAAACTAGCAACAGTTTCTGGAAAGTCCCACCTCAGTTTCAA

+

BBBBBFFFFFFFFFFFFFFFFFFFFFFFFFFFFFFFFFFFFFFFFFFFFFFFFFFFFFFFFFFFFFFFFFFFFFFFFFFFFFFFFFFFFFFFFFFFFFFFFFFFFFFFFFFFFFFFFFFFFFBFFF

@GWZHISEQ02:315:C9E6MANXX:5:1104:18595:5991

CTTGAAACTGAGGTGGGACTTTCCAGAAACTGTTGCTAGTTTCGCTTTATCTGAGTACCATCTGTTCTTGGCCCTGAGCCGGGGCCCAGGTGCTCGACCACAGATATCCTGTTTGGCCCCTGTCCC

+

BBBBBBFFFFFFFFFFFFFFFFFFFFFFFFFFFFFFFFFFFFFFFFFFFFFFFFFFFFFFFFFFFFFFFFFFFFFFFFFFFBFFFFFFF<FFFFFFFFFFFFFFFFFFFFFFFFFFFFFFFFFBFF

@GWZHISEQ02:315:C9E6MANXX:5:1113:8097:94569

CTTGAAACTGAGGTGGGACTTTCCAGAAACTGTTGCTAGTTTCGCTTTATCTGAGTACCATCTGTTCTTGGCCCTGAGCCGGGGCCCAGGTGCTCGACCACAGATATCCTGTTTGGCCCCTGTCCC

+

BBBBBFFFFFFFFFFFFFFFFFFFFFFBFFFFFFFFFFFFFFFFFFFFFFFFFFFFFFFFFFFFFFFFFFFFFFFFFFFFFFFFFFFFFFFFFFFFFFFFFFFFFFFFFFFFFFFFFFFFFFFFFF

@GWZHISEQ02:315:C9E6MANXX:5:1202:10052:71133

CTTGAAACTGAGGTGGGACTTTCCAGAAACTGTTGCTAGTTTCGCTTTATCTGAGTACCATCTGTTCTTGGCCCTGAGCCGGGGCCCAGGTGCTCGACCACAGATATCCTGTTTGGCCCCTGTCCC

+

BBBBBFFFFFFFFFFFFFFFFFFFFFFFFFFFFFFFFFFFFFFFFFFFFFFFFFFFFFFFFFFFFFFFFFFFFFFFFFFFFFFFFFFFFFFFFFFFFFFFF<FFFFFFFFFFFFFFFFFFFFFFFF

@GWZHISEQ02:315:C9E6MANXX:5:2114:15063:37834

CTTGAAACTGAGGTGGGACTTTCCAGAAACTGTTGCTAGTTTCGCTTTATCTGAGTACCATCTGTTCTTGGCCCTGAGCCGGGGCCCAGGTGCTCGACCACAGATATCCTGTTTGGCCCCTGTCCC

+

BBBBBFFFFFFFFFFFFFFFFFFFFFFFFFFFFFFFFFFFFFFFFFFFFFFFFFFFFFFFFFFFFFFFFFFFFFFFFFFFFFFFFFFFFFFFFFFFFFFFFFFFFFFFFFFFFFFFFFFFFFFBFF

@GWZHISEQ02:315:C9E6MANXX:5:1302:11880:98208

CAGGGGCCAAACAGGATATCTGTGGTCGAGCACCTGGGCCCCGGCTCAGGGCCAAGAACAGATGGTACTCAGATAAAGCGAAACTAGCAACAGTTTCTGGAAAGTCCCACCTCAGTTTCAAGTTCC

+

BBBBBF<BFFFFBBF<<FBF<FFFFFFFFFFFFFFFFFFFF<FFFFBFFFFFFF</FFFFB<F<F/FFFFFFFFBFFFFF<FFFBFFFFFFFFFFFF/FFFFFFFFFFFFFFFF</77BBFBF<FF

@GWZHISEQ02:315:C9E6MANXX:5:2207:12786:78317

CAGGGGCCAAACAGGATATCTGTGGTCGAGCACCTGGGCCCCGGCTCAGGGCCAAGAACAGATGGTACTCAGATAAAGCGAAACTAGCAACAGTTTCTGGAAAGTCCCACCTCAGTTTCAAGTTCC

+

BBBBBFFFFFFFFFFFFFFFFFFFFFFFFFFFFFFFFFFFFFFFFFFFFFFFFBFFFFFFFFFFFFFFFFFFFFFBFFFFFFFFFFFFFFFFFFFFFFFFFFFFFFFFFFFFFFFBBFFFFFFFFF

@GWZHISEQ02:315:C9E6MANXX:5:1303:15153:84384

GGGACTTGAAACTGAGGTGGGACTTTCCAGAAACTGTTGCTAGTTTCGCTTTATCTGAGTACCATCTGTTCTTGGCCCTGAGCCGGGGCCCAGGTGCTCGACCACAGATATCCTGTTTGGCCCCTG

+

BBBBBFFFFFFFFFFFFFFFFFFFFFFFFFFFFFFFFFFFFFFFFFFFFFFFFFFFFFFFFFFFFFFFFFFFFFFFFFFFFFFFFFFFFFFFFFFFFFFFFFFFFFFFFFFFFFFFFFFFFFFFFB

@GWZHISEQ02:315:C9E6MANXX:5:2202:6628:46459

AGGGGCCAAACAGGATATCTGTGGTCGAGCACCTGCGCCCCGGCTCAGGGCCAAGAACAGATGGTACTCAGATAAAGCGAAACTAGCAACAGTTTCTGGAAAGTCCCACCTCAGTTTCAAGTTCCC

+

B//<//<//</<<FFFF<BB<<//<F/<//7FF/////7/7//F//<<FFF7/</<F//<FBFF/FFFFBFBFF/FB///<BBB///<///B//</</B7/<<7FFFF/B/B/7////777//7/7

@GWZHISEQ02:315:C9E6MANXX:5:2307:13412:76181

AGGGGCCAAACAGGATATCTGTGGTCGAGCACCTGGGCCCCGGCTCAGGGCCAAGAACAGATGGTACTCAGATAAAGCGAAACTAGCAACAGTTTCTGGAAAGTCCCACCTCAGTTTCAAGTTCCC

+

BB/B<FFFFFFFFF/F<B<FFFBFFFF/7FBFFFF<BFFFFF/BBFBBFFBFFFF/BFFFFFFF<FFFFFBFBBFFFFFFFBFFFBFFFFFBFFFFFBFFFFFFFBFFF<FFFBFFFFFFFF/7BF

@GWZHISEQ02:315:C9E6MANXX:5:1205:20228:97386

GGGAACTTGAAACTGAGGTGGGACTTTCCAGAAACTGTTGCTAGTTTCGCTTTATCTGAGTACCATCTGTTCTTGGCCCTGAGCCGGGGCCCAGGTGCTCGACCACAGATATCCTGTTTGGCCCCT

+

BBBBBFFFFFFFFFFFFFFFFFFFFFFFFFFFFFFFFFFFFFFFFFFFFFFFFFFFFFFFFFFFFFFFFFFFFFFFFFFFFFFFFFBBFFFFBFFFFFFFBFFFFFFFFFFFFFFFFFFFFFFFFF

@GWZHISEQ02:315:C9E6MANXX:5:1206:5190:3612

GGGAACTTGAAACTGAGGTGGGACTTTCCAGAAACTGTTGCTAGTTTCGCTTTATCTGAGTACCATCTGTTCTTGGCCCTGAGCCGGGGCCCAGGTGCTCGACCACAGATATCCTGTTTGGCCCCT

+

BB<<BFFFFFFFFFFFFFFFFFFFFFFFFFFFFFFFFFFFFFFFFFFFF<F/F<BBFF<FFFFFB<FF<FFFFFFFFFFFFBFFFFB/BFFFF/F7FFBFF<FF7BF/7FBFFF//BFFFFFFFFB

@GWZHISEQ02:315:C9E6MANXX:5:1206:2619:72349

GGGAACTTGAAACTGAGGTGGGACTTTCCAGAAACTGTTGCTAGTTTCGCTTTATCTGAGTACCATCTGTTCTTGGCCCTGAGCCGGGGCCCAGGTGCTCGACCACAGATATCCTGTTTGGCCCCT

+

<B/BB<FF<BFFFF<FF<FF/FFFFFFBFFF/FFFF<FFFFBFBFFF<BF<F/FFFFFFF<FF/BB/FFFFFFF</F<BFF<FF/F////BFFFFFF//BFFBFFB<B<<<FBF<F7BFB7/7BBB

@GWZHISEQ02:315:C9E6MANXX:5:1213:4154:55352

GGGAACTTGAAACTGAGGTGGGACTTTCCAGAAACTGTTGCTAGTTTCGCTTTATCTGAGTACCATCTGTTCTTGGCCCTGAGCCGGGGCCCAGGTGCTCGACCACAGATATCCTGTTTGGCCCCT

+

BBBBBFFFFFFFFFFFFFFFFFFFFFFFFFFFFFFFFFFFFFFFFFFFFFFFFFFFFFFFFFFFFFFFFFFFFFFFFFFFFFFFFFFFFFFFFFFFFFFFFFFFFF<FFFFFFFFFFFFFFFFFFF

@GWZHISEQ02:315:C9E6MANXX:5:1215:7984:41574

GGGAACTTGAAACTGAGGTGGGACTTTCCAGAAACTGTTGCTAGTTTCGCTTTATCTGAGTACCATCTGTTCTTGGCCCTGAGCCGGGGCCCAGGTGCTCGACCACAGATATCCTGTTTGGCCCCT

+

BBBBBFFFFFFFFFFFFFFFFFFFFFFFFFFFFFFFFFFFFFFFFFFFFFFFFFFFFFFFFFFFFFFFFFFFFFFFFFFFFFFFFFFFFFFFFFFFFFFFFFFFFFFFFFFFFFFFFFFFFFFFFF

@GWZHISEQ02:315:C9E6MANXX:5:1308:10391:36827

GGGAACTTGAAACTGAGGTGGGACTTTCCAGAAACTGTTGCTAGTTTCGCTTTATCTGAGTACCATCTGTTCTTGGCCCTGAGCCGGGGCCCAGGTGCTCGACCACAGATATCCTGTTTGGCCCCT

+

BBBBBFFFFFFFFFFFFFFFFFFFFFFFFFFFFFFFFFFFFFFFFFFFFFFFFFFFFFFFFFFFFFFFFFFFFFFFFFFFFFFFFFFFFFFFFFFFFFFFFFFFFFFFFFFFFFFFFFFFFFFFF/

@GWZHISEQ02:315:C9E6MANXX:5:1311:7263:22178

GGGAACTTGAAACTGAGGTGGGACTTTCCAGAAACTGTTGCTAGTTTCGCTTTATCTGAGTACCATCTGTTCTTGGCCCTGAGCCGGGGCCCAGGTGCTCGACCACAGATATCCTGTTTGGCCCCT

+

BBBBBFFFFFFFFFFFFFFFFFFFFFFFFFFFFFFFFFFFFFFFFFFFFFFFFFFFFFFFFFFFFFFFFFFFFFFFBF<FFBFFFFFF//BBFFFFFFFFFFFFFF<BFBFFBFFFFBF/7BBFFF

@GWZHISEQ02:315:C9E6MANXX:5:1316:15067:71232

GGGAACTTGAAACTGAGGTGGGACTTTCCAGAAACTGTTGCTAGTTTCGCTTTATCTGAGTACCATCTGTTCTTGGCCCTGAGCCGGGGCCCAGGTGCTCGACCACAGATATCCTGTTTGGCCCCT

+

BBBBBFFFFFFFFFFFFFFFFFFFFFFFFFFFFFFFFFFFFFFFFFFFFFFFFFFFFFFFFFFFFFFFFFFFFFFFFBFFFFFFFFBFFFFFFFFFFFFFFFFFFFFFFFFFFBFFFFFFBFFFFF

@GWZHISEQ02:315:C9E6MANXX:5:2114:6631:85084

GGGAACTTGAAACTGAGGTGGGACTTTCCAGAAACTGTTGCTAGTTTCGCTTTATCTGAGTACCATCTGTTCTTGGCCCTGAGCCGGGGCCCAGGTGCTCGACCACAGATATCCTGTTTGGCCCCT

+

<BBBBFFF/FFFFFFFFFFF<FFFFFFFFFFFFFFFFFFFFFFFFFFFFFFFFFFFFFFFFFFFFFFFFBFFFFFFFFFFFFFFFFFFFFFFFFFFFFFFFFFF<FFFFFFFFFFFFFFBBFFFFF

@GWZHISEQ02:315:C9E6MANXX:5:2116:5561:44151

GGGAACTTGAAACTGAGGTGGGACTTTCCAGAAACTGTTGCTAGTTTCGCTTTATCTGAGTACCATCTGTTCTTGGCCCTGAGCCGGGGCCCAGGTGCTCGACCACAGATATCCTGTTTGGCCCCT

+

BBBBBFFFFFFFFFFFFFFFFFFFFFFFFFFFFFFFFFFFFFFFFFFFFFFFFFFFFFFFFFFFFFFFFFFFFFFFFFFFFFFFFFFFFFFFFFFFFFFFFFFFFFFFFFFFFFFFFFFFFFFFF<

@GWZHISEQ02:315:C9E6MANXX:5:2202:5267:32046

GGGAACTTGAAACTGAGGTGGGACTTTCCAGAAACTGTTGCTAGTTTCGCTTTATCTGAGTACCATCTGTTCTTGGCCCTGAGCCGGGGCCCAGGTGCTCGACCACAGATATCCTGTTTGGCCCCT

+

BBBBBFFFFFFFFFFFFFFFFFFFFFFFFFFFFFFFFFFFFFFFFFFFFFFFFFFFFFFFFFFFFFFFFFFFFFFFFFFFFFFFFFFFBFFFFFFFFFFFFFFFFFFFFFFFFFFFFFFFFFFFF<

@GWZHISEQ02:315:C9E6MANXX:5:2210:6867:12009

GGGAACTTGAAACTGAGGTGGGACTTTCCAGAAACTGTTGCTAGTTTCGCTTTATCTGAGTACCATCTGTTCTTGGCCCTGAGCCGGGGCCCAGGTGCTCGACCACAGATATCCTGTTTGGCCCCT

+

BBBBBFFFFFFFFFFFFFFFFFFFFFFFFFFFFFFFFFFFFFFFFFFFFFFFFFFFFFFFFFFFFFFFFFFFFFFFFFFFFFFFFFFFFBBFFFFFFFFFFFFFFFFFFFFFFFFFFFFFFFFFFF

@GWZHISEQ02:315:C9E6MANXX:5:2301:4829:30368

GGGGACTTGAAACTGAGGTGGGACTTTCCAGAAACTGTTGCTAGTTTCGCTTTATCTGAGTACCATCTGTTCTTGGCCCTGAGCCGGGGCCCAGGTGCTCGACCACAGATATCCTGTTTGGCCCCT

+

B/BBB<FFFFFFFFFFFFFFFFFFFFFF<FFFFFFFFFFFFFFFFFFFFFFFFBFFFFF<FFFFFFFFFFFFF///<BBBF/FFBBF<</FFF<7</F</7BB/7////7F/FFF<FB//7/7BBB

@GWZHISEQ02:315:C9E6MANXX:5:2303:19195:82842

GGGAACTTGAAACTGAGGTGGGACTTTCCAGAAACTGTTGCTAGTTTCGCTTTATCTGAGTACCATCTGTTCTTGGCCCTGAGCCGGGGCCCAGGTGCTCGACCACAGATATCCTGTTTGGCCCCT

+

/BBBBFFFFFFFFFFFFFFFFFFFFFFFFFFFFFFFFFFFFFFFFFFFFFFFFFFFFFFFFFFF<FFFFFFFFFFFFFFFFFFFFFFFFFFFFFFFFFFFFFFFFFFFFFFFFFFFFFFF<BFFFF

@GWZHISEQ02:315:C9E6MANXX:5:2312:17634:35491

GGGAACTTGAAACTGAGGTGGGACTTTCCAGAAACTGTTGCTAGTTTCGCTTTATCTGAGTACCATCTGTTCTTGGCCCTGAGCCGGGGCCCAGGTGCTCGACCACAGATATCCTGTTTGGCCCCT

+

BBBBBFFFFFFFFFFFFFFFFFFFFFFFFFFFFFFFFFFFFFFFFFFFFFFFFFFFFFFFFFFFFFFFFFFFFFFBFFFFFFFFFFFFFFFFFFFFFFFFFFFFFFFFFFFFFFFFFFFFFFFFF/

@GWZHISEQ02:315:C9E6MANXX:5:2316:4967:54600

GGGAACTTGAAACTGAGGTGGGACTTTCCAGAAACTGTTGCTAGTTTCGCTTTATCTGAGTACCATCTGTTCTTGGCCCTGAGCCGGGGCCCAGGTGCTCGACCACAGATATCCTGTTTGGCCCCT

+

/BBBBFFFFFFFFFFFFFFFFFFFFFFFFFFFFFFFFFFFFFFFFFFFFFFFFFFFFFFFFFFFFFFFFFFFFFFFFFFFFFFFFFFFFFFFFFFFFFFFFFFFFFFFFFFFFFFFFFFFFFFFFB

@GWZHISEQ02:315:C9E6MANXX:5:2316:10084:68325

GGGAACTTGAAACTGAGGTGGGACTTTCCAGAAACTGTTGCTAGTTTCGCTTTATCTGAGTACCATCTGTTCTTGGCCCTGAGCCGGGGCCCAGGTGCTCGACCACAGATATCCTGTTTGGCCCCT

+

<BB<BFFFFFFFFFFFFFFFFFFFFFFFFFFFFFFFFFFFFFFFFFFFFFFFFFFFFFFFFFFFFFFFFFFFFFFFFFFFFFFFFFFFFFFFFFFFFFFFFFFFFFFFFFFFFFFFFFFFFFFFFF

@GWZHISEQ02:315:C9E6MANXX:5:2107:14549:74976

GGGGCCAAACAGGATATCTGTGGTCGAGCACCTGGGCCCCGGCTCAGGGCCAAGAACAGATGGTACTCAGATAAAGCGAAACTAGCAACAGTTTCTGGAAAGTCCCACCTCAGTTTCAAGTTCCCC

+

BBBBBFF<<BF<<FFFFFFFF<<FF/FBFFFFFFFBFFFF</BBFFFFFFF/FF</B///<</<BFF<F<BFBFFBF<FFFFF/BBB/<7/F/<<7<BB77BBF<<FFFF/F777BFFFFFFFFFB

@GWZHISEQ02:315:C9E6MANXX:5:2206:3423:69151

GGGGCCAAACAGGATATCTGTGGTCGAGCACCTGGGCCCCGGCTCAGGGCCAAGAACAGATGGTACTCAGATAAAGCGAAACTAGCAACAGTTTCTGGAAAGTCCCACCTCAGTTTCAAGTTCCCC

+

BBBBBFFFFFFFFFFFFFFFFFFFFFFFFFFFFFFFFFFFFFFFFFFFFFFFFFFFFFFFFFFFFFFFFFFFFFFFFFFFFFFFFFFFFFFFFFFFFFFFFFFFFFFFFFFFFFFFFFFFFFFFFB

@GWZHISEQ02:315:C9E6MANXX:5:1107:10768:80936

GGGGAACTTGAAACTGAGGTGGGACTTTCCAGAAACTGTTGCTAGTTTCGCTTTATCTGAGTACCATCTGTTCTTGGCCCTGAGCCGGGGCCCAGGTGCTCGACCACAGATATCCTGTTTGGCCCC

+

BBBBBFFFFFFFFFFFFFFFFFFFFFFFFFFFFFFFFFFFFFFFFFFFFFFFFFFFFFFFFFFFFFFFFFFFFFFFFFFFFFFFFFFFFFFFFBFFFFFFFFFFFFFFFFFFFFFFFFFFFFFFFF

@GWZHISEQ02:315:C9E6MANXX:5:1309:9248:81282

GGGGAACTTGAAACTGAGGTGGGACTTTCCAGAAACTGTTGCTAGTTTCGCTTTATCTGAGTACCATCTGTTCTTGGCCCTGAGCCGGGGCCCAGGTGCTCGACCACAGATATCCTGTTTGGCCCC

+

BBBBBFFFFFFFFFFFFFFFFFFFFFFFFFFFFFFFFFFFFFFFFFFFFFFFFFFFFFFFFFFFFFFFFFFFFFFFFFFFFFFFFFFFFFFFFFFFFFFFFFFFFFFFFFFFFFFFFFFFFFFFFF

@GWZHISEQ02:315:C9E6MANXX:5:2213:8949:44091

GGGGAACTTGAAACTGAGGTGGGACTTTCCAGAAACTGTTGCTAGTTTCGCTTTATCTGAGTACCATCTGTTCTTGGCCCTGAGCCGGGGCCCAGGTGCTCGACCACAGATATCCTGTTTGGCCCC

+

BBBBBFFFFFFFFFFFFFFFFFFBFFFFFFFFFFFFFFFFFFFFFFFFFFFFFFFFFFFFFFFFFFFFFFFFFFFFFFFFFFBFFFFFFFFFFFFFFFFFFFFFFFFFFFFFFFFFFFFFFFFFFF

@GWZHISEQ02:315:C9E6MANXX:5:2314:1971:2383

GGGGAACTTGAAACTGAGGTGGGACTTTCCAGAAACTGTTGCTAGTTTCGCTTTATCTGAGTACCATCTGTTCTTGGCCCTGAGCCGGGGCCCAGGTGCTCGACCACAGATATCCTGTTTGGCCCC

+

BBBBBFFFFFFFFFFFFFFFFFFFFFFFFFFFFFFFFFFFFFFFFFFFFFFFFFFFFFFFFFFFFFFFFFFFFFFFFFFFFFFFFFFFFFFFFBFFFFFFFFFFFFFFFFFFFFFFFFFFFFFFFF

@GWZHISEQ02:315:C9E6MANXX:5:2206:8548:21415

AGGGCCAAACAGGATATCTGTGGTCGAGCACCTGGGCCCCGGCTCAGGGCCAAGAACAGATGGTACTCAGATAAAGCGAAACTAGCAACAATTTCTGGAAAGTCCCACCTCAGTTTCAAGTTCCCC

+

BBBB/B/<FB//F/BB/<<F//F////////FF/FF/FFF/F//<</FF/</<<<FF////<F/B/<//<///BF////<F/<///7FF///<F///F7<FB//<B//<///7/7FBBB//B/FFF

@GWZHISEQ02:315:C9E6MANXX:5:1105:5382:11628

TGGGGAACTTGAAACTGAGGTGGGACTTTCCAGAAACTGTTGCTAGTTTCGCTTTATCTGAGTACCATCTGTTCTTGGCCCTGAGCCGGGGCCCAGGTGCTCGACCACAGATATCCTGTTTGGCCC

+

BBBBBFFFFFFFFFFFFFFFFFFFFFFFFFFFFFFFFFFFFFFFFFFFFFFFFFFFFFFFFFFFFFFFFFFFFFFFFFFFFFFFFFFFFFFFFFFFFFFFFFFFFFFFFFFFBFFFFFFFFFFFFF

@GWZHISEQ02:315:C9E6MANXX:5:1116:9541:40485

GGCCAAACAGGATATCTGTGGTCGAGCACCTGGGCCCCGGCTCAGGGCCAAGAACAGATGGTACTCAGATAAAGCGAAACTAGCAACAGTTTCTGGAAAGTCCCACCTCAGTTTCAAGTTCCCCAA

+

BBBBBFFFFFFFFFFFFFFFFFFFFFFFFFFFFFFFFFFFFBFFFFFFFFFFFFFF<FBFFFFF//BFFFFFFF/7FFFBFFF/7<FFFF/BFBBFFFFFFF7BB7BBBBF//7BFFBF77F//BF

@GWZHISEQ02:315:C9E6MANXX:5:1203:6709:42987

GGCCAAACAGGATATCTGTGGTCGAGCACCTGGGCCCCGGCTCAGGGCCAAGAACAGATGGTACTCAGATAAAGCGAAACTAGCAACAGTTTCTGGAAAGTCCCACAGATCGGAAGAGCGTCGTGT

+

BBBBB<FFFFFFFFFFFFFFFFFFFFFFFFFFFFFFFFFBFFFFFF<FFFFFFFFFFFFFFFFF/FFFFFFFFFFFFFFBFFBFFFFFFFFFFFFFFFBFFF/BFFFFFBBFFFFFFF<<7/7BB7

@GWZHISEQ02:315:C9E6MANXX:5:1207:12789:6758

GGCCAAACAGGATATCTGTGGTCGAGCACCTGGGCCCCGGCTCAGGGCCAAGAACAGATGGTACTCAGATAAAGCGAAACTAGCAACAGTTTCTGGAAAGTCCCACCTCAGTTTCAAGTTCCCCAA

+

BBBBBFFFFFFFFFFFFFFFFFFFFFFFFFFFFFFFFFFFFFFFFFFFFFFFFFFFFBFFFFFFFFFFFFFFFFFFFFFFFFFFFFFFFFFFFFFFFFFFFFFFFFFFFFFFFFFFFFFFFFFFFF

@GWZHISEQ02:315:C9E6MANXX:5:1211:15361:49553

GGCCAAACAGGATATCTGTGGTCGAGCACCTGGGCCCCGGCTCAGGGCCAAGAACAGATGGTACTCAGATAAAGCGAAACTAGCAACAGTTTCTGGAAAGTCCCACCTCAGTTTCAAGTTCCCCAA

+

BBBBBFFFFFFFFFFFFFFFFFFFFFFFFFFFFFFFFFFFFFFFFFFFFFFFFFFFFFFFFFFFFFFFFFFFFFFFFFFFFFFFFFFFFFFFFFFFFFFFFFFFFFFFFFFFFFFFFFFFFFFFFF

@GWZHISEQ02:315:C9E6MANXX:5:1212:13207:100653

GGCCAAACAGGATATCTGTGGTCGAGCACCTGGGCCCCGGCTCAGGGCCAAGAACAGATGGTACTCAGATAAAGCGAAACTAGCAACAGTTTCTGGAAAGTCCCACCTCAGTTTCAAGTTCCCCAA

+

BBBBBFFFFFFFF<BFFFFFFFFFFBFFFFFFFBFFFFFFFFFFFBFFFFFFFFBFF<FFFFBFFFFFFFFFFFFFFFFFFF<BFFFFFFBBFFBBFFBBFFFFBFFFFFF//7BF/F/BFFFFFF

@GWZHISEQ02:315:C9E6MANXX:5:1302:6000:50497

GGCCAAACAGGATATCTGTGGTCGAGCACCTGGGCCCCGGCTCAGGGCCAAGAACAGATGGTACTCAGATAAAGCGAAACTAGCAACAGTTTCTGGAAAGCCCCAGATCGGAAGAGCGTCGTGTAG

+

BBBBBFFFFFFFFFFFFFFFFFFFFFFFFFFFFFFFFFFFFFFFFFFFFFFFFFFFFFFFFFFFFFFFFFFFFFFFFBFFFFFFFFFFFFFFFFFFFFFFFFFFFFFFFFFFFBFFFFBBFFBBFF

@GWZHISEQ02:315:C9E6MANXX:5:1304:12728:45211

GGCCAAACAGGATATCTGTGGTCGAGCACCTGGGCCCCGGCTCAGGGCCAAGAACAGATGGTACTCAGATAAAGCGAAACTAGCAACAGTTTCTGGAAAGTCCCACCTCAGTTTCAAGTTCCCCAA

+

BBBBBFFFFFFFFFFFFFFFFFFFFFFFFFFFFFFFFFFFFFFFFFFFFFFFFFFFFFFFFFFFFFFFFFFFFFFFFFFFFFFFFFFFFFFFFFFFFFFFFFFFFFFFFFFFFFFFFFFFFFFFFF

@GWZHISEQ02:315:C9E6MANXX:5:2102:5874:27564

GGCCAAACAGGATATCTGTGGTCGAGCACCTGGGCCCCGGCTCAGGGCCAAGAACAGATGGTACTCAGATAAAGCGAAACTAGCAACAGTTTCTGGAAAGTCCCACCTCAGTTTCAAGTTCCCCAA

+

BBBBBFFFFFFFFFFFFFFBFFFFBFFFFFBFFFFFFFF<BFFFF<FFFFFFFFFFFBFFFFFFFFFFFFFFFFFFFBFFFFFFFFFFFFFFFFFFFBFFFFFFFFFFFFF<FFFFFFFFBFFBBB

@GWZHISEQ02:315:C9E6MANXX:5:2204:11421:18256

GGCCAAACAGGATATCTGTGGTCGAGCACCTGGGCCCCGGCTCAGGGCCAAGAACAGATGGTACTCAGATAAAGCGAAACTAGCAACAGTTTCTGGAAAGTCCCACCTCAGTTTCAAGTTCCCCAA

+

BBBBBFFFFFFFFBFBFFFFFFFFFFFBFFFFFFFFFFFFFFFFFFFBFFFFFFFBFBFFFFFFFFFB<FFFFFFFFFFFFFFFFFFFFFFFFFFBFFFFFFBFFFFFFFBFFFFBFFFFFFFFFF

@GWZHISEQ02:315:C9E6MANXX:5:2205:1390:29796

GGCCAAACAGGATATCTGTGGTCGAGCACCTGGGCCCCGGCTCAGGGCCAAGAACAGATGGTACTCAGATAAAGCGAAACTAGCAACAGTTTCTGGAAAGTCCCACCTCAGTTTCAAGTTCCCCAA

+

BBBB/<FFFBBFF<FFF<FFFFFBFFBFFFFFFFFFFFBB<7FB<FFFFFFFFFFFFBFBFB/F//FF/FBFFFFFFFFFFF<FFBFFFFF/FFF/BBFFFFB/BFFFFFFF<FFFFFB7FBFFFF

@GWZHISEQ02:315:C9E6MANXX:5:2209:14243:44014

GGCCAAACAGGATATCTGTGGTCGAGCACCTGGGCCCCGGCTCAGGGCCAAGAACAGATGGTACTCAGATAAAGCGAAACTAGCAACAGTTTCTGGAAAGTCCCACAGATCGGAAGAGCGTCGTGT

+

BBBBBFFFFFFFFFFFFFFFFFFFFFFFFFFFFFFFFFFFFFFFFFFFFFFFFFFFFFFFFFFFFFFFFFFFFFFFFFFFFFFFFFFFFFFFFFFFFFFFFFFFFFFFFFFFFFFFFFFFBFBBF7

@GWZHISEQ02:315:C9E6MANXX:5:2209:10872:72280

GGCCAAACAGGATATCTGTGGTCGAGCACCTGGGCCCCGGCTCAGGGCCAAGAACAGATGGTACTCAGATAAAGCGAAACTAGCAACAGTTTCTGGAAAGTCCCACCTCAGTTTCAAGTTCCCCAA

+

BBBBBFFFFFFFFFFFFFFFFFFFFFFFFFFFFFFFFFFFFFFFFFFFFFFFFFFFFFFFFFFFFFFFFFFFFFFFFFFFFFFFFFFFFFF///F/FF///<F<BF//7/<FFB/F//B/FFF/BF

@GWZHISEQ02:315:C9E6MANXX:5:2213:14818:78245

GGCCAAACAGGATATCTGTGGTCGAGCACCTGGGCCCCGGCTCAGGGCCAAGAACAGATGGTACTCAGATAAAGCGAAACTAGCAACAGTTTCTGGAAAGTCCCACCTCAGTTTCAAGTTCCCCAA

+

BBBBBFFFFFFFFFFFFFFFFFFFFFFFFFFFFFFFFFFFFFFFFFFFFFFFFFFFFFFFFFFFFFBFFFFFFFFFFFFFFFFFFFFFFBFFFFFFFFFFFFFFFFFFFFFFFFFFFFFFFFFFFF

@GWZHISEQ02:315:C9E6MANXX:5:2302:12464:77266

GGCCAAACAGGATATCTGTGGTCGAGCACCTGGGCCCCGGCTCAGGGCCAAGAACAGATGGTACTCAGATAAAGCGAAACTAGCAACAGTTTCTGGAAAGTCCCAAGATCGGAAGAGCGTCGTGTA

+

BBBBBFFFFFFFFFFFFFFFFFFFFFFFFFFFFFFFFFFFFFFFFFFFFFFFFFFFFFFFFFFFFFFFFFFFFFFFFFFFFFFFFFFFFFFFFFFFFFFFFFFFFFFFFFFFFFFFFFFBFFBFFF

@GWZHISEQ02:315:C9E6MANXX:5:2303:8899:68577

GGCCAAACAGGATATCTGTGGTCGAGCACCTGGGCCCCGGCTCAGGGCCAAGAACAGATGGTACTCAGATAAAGCGAAACTAGCAACAGTTTCTGGAAAGTCCCACCTCAGTTTCAAGTTCCCCAA

+

BBBBBFFFFFFFFFFFFFFFFFFFFFFFFFFFFFFFFFFFFFFFFFFFFFFFFFFFFFFFFFFFFFFFFFFFFFFFFFFFFFFFFFFFFFFFFFFFFFFFFFFFFFFFFFFFFFFFFFFFFFFFFF

@GWZHISEQ02:315:C9E6MANXX:5:2305:14829:12933

GGCCAAACAGGATATCTGTGGTCGAGCACCTGGGCCCCGGCTCAGGGCCAAGAACAGATGGTACTCAGATAAAGCGAAACTAGCAACAGTTTCTGGAAAGTCCCACCTCAGTTTCAAGTTCCCCAA

+

BBBBBFFFFFFFFFFFFFFFFFFFFFFFFFFFFFFFFFFFFFFFFFFFFFFFFFFFFFFFFFFFFFFFFFFFFFFFFFFFFFFFFFFFFFFFFFFFFFFFFFFFFFFFFFFBFFFFFFFFFFFFFF

@GWZHISEQ02:315:C9E6MANXX:5:2306:15690:77181

GGCCAAACAGGATATCTGTGGTCGAGCACCTGGGCCCCGGCTCAGGGCCAAGAACAGATGGTACTCAGATAAAGCGAAACTAGCAACAGTTTCTGGAAAGTCCCACCTCAGTTTCAAGTTCCCCAA

+

BBBBBBBBFFBBF<FFFFFFFFFFFFFFFFFFFFFFFFFFFFFFFFFFFFFFFFFFFFFFFFFFFFFFFFFFFFFFFFFFFFFFFFFFFBFBFFFFFFFFFFFFFFFFFFFFFFFFFFFFFFFFFF

@GWZHISEQ02:315:C9E6MANXX:5:2311:12966:39637

GGCCAAACAGGATATCTGTGGTCGAGCACCTGGGCCCCGGCTCAGGGCCAAGAACAGATGGTACTCAGATAAAGCGAAACTAGCAACAGTTTCTGGAAAGTCCCACCTCAGTTTCAAGTTCCCCAA

+

BBBBBF<FFB<FFFFFFFFFFBFFFBBFF<FFB<<BBFBBFFFFBF//B<FBF/B<BBFFFBBFBBBFFFFFBBF<</FFFBBF/77BBFFFBF/F/BFB7//<F7//<F//7B/7/777//BF//

@GWZHISEQ02:315:C9E6MANXX:5:1203:6709:42987

GTGGGACTTTCCAGAAACTGTTGCTAGTTTCGCTTTATCTGAGTACCATCTGTTCTTGGCCCTGAGCCGGGGCCCAGGTGCTCGACCACAGATATCCTGTTTGGCCAGATCGGAAGAGCACACGTC

+

BB<<BFFFFFFFFFFFFFFFFFFFFFFFFFFFFFFFFF<B<BFFFFFFFFFFFFFFFF//FFFFFFFFFFF<F<BB<FFFBFFBFFFFFFFBFFFFBF<F</BBBFFFFFF<FFBFBFFF<FFFBF

@GWZHISEQ02:315:C9E6MANXX:5:1302:6000:50497

GGGGCTTTCCAGAAACTGTTGCTAGTTTCGCTTTATCTGAGTACCATCTGTTCTTGGCCCTGAGCCGGGGCCCAGGTGCTCGACCACAGATATCCTGTTTGGCCAGATCGGAAGAGCACACGTCTG

+

BBBBBFFFFFFFFFFFFFFFFFFFFFFFFFFFFFFFFFFFFFFFFFFFFFFFFFFFFFFFFFFFFFFFFFFFFFFFFFFFFFFFFFFFFFFFFFFFFFFFFFFFFFFFFFFFFFFFFFFFFFFFFF

@GWZHISEQ02:315:C9E6MANXX:5:2209:14243:44014

GTGGGACTTTCCAGAAACTGTTGCTAGTTTCGCTTTATCTGAGTACCATCTGTTCTTGGCCCTGAGCCGGGGCCCAGGTGCTCGACCACAGATATCCTGTTTGGCCAGATCGGAAGAGCACACGTC

+

BBBBBFFFFFFFFFFFFFFFFFFFFFFFFFFFFFBFFFFFFFFFFFFFFFFFFFFFFFFFFFFFFFFFFFFFFFFFFFFFFFFFFFFFFFFFFFFFFFFFFFFFFFFFFFFFFFFFFFFFFFFFFF

@GWZHISEQ02:315:C9E6MANXX:5:2302:12464:77266

TGGGACTTTCCAGAAACTGTTGCTAGTTTCGCTTTATCTGAGTACCATCTGTTCTTGGCCCTGAGCCGGGGCCCAGGTGCTCGACCACAGATATCCTGTTTGGCCAGATCGGAAGAGCACACGTCT

+

BBBBBFFFFFFFFFFFFFFFFFFFFFFFFFFFFFFFFFFFFFFFFFFFFFFFFFFFFFFFFFFFFFFFFFFFFFFFFFFFFFFFFFFFFFFFFFFFFFFFFFFFFFFFFFFFFFFFFFFFFFFFF<

@GWZHISEQ02:315:C9E6MANXX:5:1104:18434:7063

GCCAAACAGGATATCTGTGGTCGAGCACCTGGGCCCCGGCTCAGGGCCAAGAACAGATGGTACTCAGATAAAGCGAAACTAGCAACAGTTTCTGGAAAGTCCCACCTCAGTTTCAAGTTCCCCAAA

+

BBBBBFFFFFFFFFFFFFFFFFFFFFFFFFFFFFFFFFFFFFFFFFFFFFFFFFFFFFFFFFFFFFFFFFFFFFFFFFFFFFFFFFFFBFFFFFFFFFFFFFFFFFFFFFFFFFFFFFFFFFFFFF

@GWZHISEQ02:315:C9E6MANXX:5:1114:15399:88022

GCCAAACAGGATATCTGTGGTCGAGCACCTGGGCCCCGGCTCAGGGCCAAGAACAGATGGTACTCAGATAAAGCGAAACTAGCAACAGTTTCTGGAAAGTCCCACCTCAGTTTCAAGTTCCCCAAA

+

BBBBBFFFFFFFFFFFFFFFFFFFFFFFFFFFFFFFFFFFFFFFFFFFFFFFFFFFFFFFFFFFFFFFFFFFFFFFFFFFFFFFFFFFFFFFFFFFFFFFFFFFFFFFFFFFFFFFFFFFFFFFFF

@GWZHISEQ02:315:C9E6MANXX:5:1115:9517:44989

GCCAAACAGGATATCTGTGGTCGAGCACCTGGGCCCCGGCTCAGGGCCAAGAACAGATGGTACTCAGATAAAGCGAAACTAGCAACAGTTTCTGGAAAGTCCCACCTCAGTTTCAAGTTCCCCAAA

+

BBBBBFFFFFFFFFFFFFFFFFFFFFFFFFFFFFFFFFFFFFFFFFFFFFFFFFFFFFFFFFFFFFFFFFFFFFFFFFFFFFFFFFFFFFFFFFFFFFFFFFFFFFFFFFFFFFFFFBFFFFFFFF

@GWZHISEQ02:315:C9E6MANXX:5:1304:14171:43004

GCCAAACAGGATATCTGTGGTCGAGCACCTGGGCCCCGGCTCAGGGCCAAGAACAGATGGTACTCAGATAAAGCGAAACTAGCAACAGTTTCTGGAAAGTCCCACCTCAGTTTCAAGTTCCCCAAA

+

BBBBBFFFFFFFFFFFFFFFFFFFFFFFFFFFFFFFFBFFFFFFFFFFFFFFFFFFFFFFFFFFFFFFFFFFFFFFFFFFFFFFFFFFFFFFFFFFFFFFFFFFFFFFFFFBFFFFFFFFFFFFFF

@GWZHISEQ02:315:C9E6MANXX:5:2206:14787:40608

GCCAAACAGGATATCTGTGGTCGAGCACCTGGGCCCCGGCTCAGGGCCAAGAACAGATGGTACTCAGATAAAGCGAAACTAGCAACAGTTTCTGGAAAGTCCCACCTCAGTTTCAAGTTCCCCAAA

+

BBBBBFFFFFFFFFFFFFFFFFFFFFFFFFFFFFFFFFFFFFFFFFFFFFFFFFFFFFFFFFFFFFFFFFFFFFFFFFFFFFFFFFFFFFFFFFFFFFFFFFFFFFFFFFFFFFFFFFFFFFFFFF

@GWZHISEQ02:315:C9E6MANXX:5:2207:14972:85364

GCCAAACAGGATATCTGTGGTCGAGCACCTGGGCCCCGGCTCAGGGCCAAGAACAGATGGTACTCAGATAAAGCGAAACTAGCAACAGTTTCTGGAAAGTCCCACCTCAGTTTCAAGTTCCCCAAA

+

BBBBBFFFFFFFFFFFFFFFFFFFFFFFFFFFFFFFFFFFFFFFFFFFFFFFFFFFFFFFFFFFFFFFFFFFFFFFFFFFFFFFFFFFFFFFFFFFFFFFFFFFFFFFFFBFFFBFFFFFFFFFFF

@GWZHISEQ02:315:C9E6MANXX:5:2207:6165:89695

GCCAAACAGGATATCTGTGGTCGAGCACCTGGGCCCCGGCTCAGGGCCAAGAACAGATGGTACTCAGATAAAGCGAAACTAGCAACAGTTTCTGGAAAGTCCCACCTCAGTTTCAAGTTCCCCAAA

+

////<BFFBFFFFFFFFFFFFB/////</FB<F<FFFF/BFFFBB<FBFB<FFFFF/FFFFFBFFFFF//FB/<7FBFFFBFBFFF/7<FBFFFFFFFFFFFFBFFBFBFFFFF/BF7FFFFFFFB

@GWZHISEQ02:315:C9E6MANXX:5:2316:5668:44924

CCAAACAGGATATCTGTGGTCGAGCACCTGGGCCCCGGCTCAGGGCCAAGAACAGATGGTACTCAGATAAAGCGAAACTAGCAACAGTTTCTGGAAAGTCCCACCTCAGTTTCAAGTTCCCCAAAA

+

B/BBB/<<F/F/BBFBF<FFFFBB<FFFFFFFFFFFFFBFFFBBFFFFFFFFFFFFFFFFFFFFFFFFFFFFFFFFFFFFFFFFFFFFFFBBFFFFFFFF/BFBFFFFF<BBFFBBFFF//BBBFF

@GWZHISEQ02:315:C9E6MANXX:5:1104:5422:23091

CAAACAGGATATCTGTGGTCGAGCACCTGGGCCCCGGCTCAGGGCCAAGAACAGATGGTACTCAGATAAAGCGAAACTAGCAACAGTTTCTGGAAAGTCCCACCTCAGTTTCAAGTTCCCCAAAAG

+

BBBBBFFFFFFFFFFFFFFFFFFFFFFFFFFFFFFFFFFFFFFFFFFFFFFFFFFFFFFFFFFFFFFFFFFFFFFFFFFFFFFFFFFFFFFFFFFFFFFFFFFFFFFFFFFFFFFFFFFFFFFFFF

@GWZHISEQ02:315:C9E6MANXX:5:1205:4572:65745

CAAACAGGATATCTGTGGTCGAGCACCTGGGCCCCGGCTCAGGGCCAAGAACAGATGGTACTCAGATAAAGCGAAACTAGCAACAGTTTCTGGAAAGTCCCACCTCAGTTTCAAGTTCCCCAAAAG

+

BBBBBFFFFFFFFFFFFFFFFFFFFFFFFFFFFFFFFFFFFFFFFFFFFFFFFFFFFFFFFFFFFFFFFFFFFFFFFFFFFFFFFFFFFFFFFFFFFFFFFFFFFFFFFFFFFFFFFFFFFFFFF/

@GWZHISEQ02:315:C9E6MANXX:5:1104:15995:79633

CTTTTGGGGAACTTGAAACTGAGGTGGGACTTTCCAGAAACTGTTGCTAGTTTCGCTTTATCTGAGTACCATCTGTTCTTGGCCCTGAGCCGGGGCCCAGGTGCTCGACCACAGATATCCTGTTTG

+

BBBBBFBFFFFFFFFFFFFFFFFFFF<BFFFFFFFFFFFFFFFFFFFFFFFFFFFFFFFFBBFFFFFFFFFFFFBFFFFFFFFFFFFFBFFFBB/FFFFF<FFFFFFFFFFFFFFFFFFFFFFFFF

@GWZHISEQ02:315:C9E6MANXX:5:1106:6134:23612

CTTTTGGGGAACTTGAAACTGAGGTGGGACTTTCCAGAAACTGTTGCTAGTTTCGCTTTATCTGAGTACCATCTGTTCTTGGCCCTGAGCCGGGGCCCAGGTGCTCGACCACAGATATCCTGTTTG

+

BBBBBFFFFFFFFFFFFFFFFFFFFFFFFFFFFFFFFFFFFFFFFFFFFFFFFFFFFFFFFFFFFFFFFFFFFFFFFFFFFFFFFFFFFFFFFFFFFFFFFFFFFFFFFFFFFFFFFFFFFFFFFF

@GWZHISEQ02:315:C9E6MANXX:5:1201:8909:31088

CTTTTGGGGAACTTGAAACTGAGGTGGGACTTTCCAGAAACTGTTGCTAGTTTCGCTTTATCTGAGTACCATCTGTTCTTGGCCCTGAGCCGGGGCCCAGGTGCTCGACCACAGATATCCCGTTTG

+

/BBBBFFFFFFFFFFFFFFFFFFFFFFFFFFFFFFFFFFFFFFFFFFFFFFFFFFFFFFFFFFFFFFFFFFFFFFFFFFFFFFFFFFFFFFFFFFFFFFFFFFFFFFFFFBFFFFFFFFFFFFFFF

@GWZHISEQ02:315:C9E6MANXX:5:1204:18174:43750

CTTTTGGGGAACTTGAAACTGAGGTGGGACTTTCCAGAAACTGTTGCTAGTTTCGCTTTATCTGAGTACCATCTGTTCTTGGCCCTGAGCCGGGGCCCAGGTGCTCGACCACAGATATCCTGTTTG

+

BBBBBFFFFFFFFFFFFFFFFFFFFFFFFFFFFFFFFFFFFFFFFFFFFFFFFFFFFFFFFFFFFFFFFFFFFFFFFFFFFFFFFFFFFFFFFFFFFFFFFFFFFFFFFFFFFFFFFFFFFFFFFF

@GWZHISEQ02:315:C9E6MANXX:5:1314:4506:66792

CTTTTGGGGAACTTGAAACTGAGGTGGGACTTTCCAGAAACTGTTGCTAGTTTCGCTTTATCTGAGTACCATCTGTTCTTGGCCCTGAGCCGGGGCCCAGGTGCTCGACCACAGATATCCTGTTTG

+

BBBBBFFFFFFFFFFFFFFFFFFFFFFFFFFFFFFFFFFFFFFFFFFFFFFFFFFFFFFFFFFFFFFFFFFFFFFFFFFFFFFFFFFF<FFFFBFFBFFFFFFFFFFFFFFFFFFFFFFFFFFFFF

@GWZHISEQ02:315:C9E6MANXX:5:2202:13219:36832

CTTTTGGGGAACTTGAAACTGAGGTGGGACTTTCCAGAAACTGTTGCTAGTTTCGCTTTATCTGAGTACCATCTGTTCTTGGCCCTGAGCCGGGGCCCAGGTGCTCGACCACAGATATCCTGTTTG

+

BBBBBFFFFFFFFFFFFFFFFFFFFFFFFFFFFFFFFFFFFFFFFFFFFFFFFFFFFFFFFFFFFFFFFFFFFFFFFFFFFFFFFFFFFFFFFFFFFFFFFFFFFFFFFFFFFFFFBFFFFFFFFF

@GWZHISEQ02:315:C9E6MANXX:5:2302:15923:84753

CTTTTGGGGAACTTGAAACTGAGGTGGGACTTTCCAGAAACTGTTGCTAGTTTCGCTTTATCTGAGTACCATCTGTTCTTGGCCCTGAGCCGGGGCCCAGGTGCTCGACCACAGATATCCTGTTTG

+

BBBBBFFFFFFFFFFFFFFFFFFFFFFFFFFFFFFFFFFFFFFFFFFFFFFFFFFFFFFFFFFFFFFFFFFFFFFFFFFFFFFFFFFFFFFFFFFFFFFFFFFFFFFFFFFFFFFFBFFFFFFFFB

@GWZHISEQ02:315:C9E6MANXX:5:2309:3936:4803

CTTTTGGGGAACTTGAAACTGAGGTGGGACTTTCCAGAAACTGTTGCTAGTTTCGCTTTATCTGAGTACCATCTGTTCTTGGCCTTGAGCCGGGGCCCAGGTGCTCGACCACAGATATCCTGTTTG

+

BBBBBFFFFFFFFFFFFFFFFFFFFFFFFFFFFFFFF<FFFFFFFFFFFFFFFFFBF<FFFFFFFFFFFFFFFFFFFFFFFFFF<BFFFFFFFFFFFFFFFFFFFFFFFFFFFF<FFFFFFFFFFF

@GWZHISEQ02:315:C9E6MANXX:5:2309:20283:61867

CTTTTGGGGAACTTGAAACTGAGGTGGGACTTTCCAGAAACTGTTGCTAGTTTCGCTTTATCTGAGTACCATCTGTTCTTGGCCCTGAGCCGGGGCCCAGGTGCTCGACCACAGATATCCTGTTTG

+

BBBBBFFFFFFFFFFFFFFFFFFFFFFFFFFFFFFFFFFFFFFFFFFFFFFFFFFFFFFFFFFFFFFFFFFFFFFFFFFFFFFFFFFFFFFFFFFFFFFFFFFFFFFFFFFFFFFFFFFFFFFFFF

@GWZHISEQ02:315:C9E6MANXX:5:2313:4809:10184

CTTTTGGGGAACTTGAAACTGAGGTGGGACTTTCCAGAAACTGTTGCTAGTTTCGCTTTATCTGAGTACCATCTGTTCTTGGCCCTGAGCCGGGGCCCAGGTGCTCGACCACAGATATCCTGTTTG

+

BBBBBFB/<FFFB/FFFFFBBFFFFFBBFFB<FBFFFFFF<BFFFFFFFFFFFFFFFFFFFFFFBFF<BFFFFFFFFF<FFFF/<BFFFFFFFFFFBBFB<FBFFF<BBFFFFF<FFFFFFFFFFF

@GWZHISEQ02:315:C9E6MANXX:5:1206:11588:14340

AAACAGGATATCTGTGGTCGAGCACCTGGGCCCCGGCTCAGGGCCAAGAACAGATGGTACTCAGATAAAGCGAAACTAGCAACAGTTTCTGGAAAGTCCCACCTCAGTTTCAAGTTCCCCAAAAGA

+

BB<BBFFFFFFBFFFFFFFFFFFFFFFFFFFFFFFFFFFFFFFFFFFFFFFFFFFBF<FFFFFFFFFFFFFFFFFFFFFFFFFFFFFFFFFFFFFFF<BFFFFFFFFBFFFFFFFFFFFFFFFFFF

@GWZHISEQ02:315:C9E6MANXX:5:2210:6051:65317

AAACAGGATATCTGTGGTCGAGCACCTGGGCCCCGGCTCAGGGCCAAGAACAGATGGTACTCAGATAAAGCGAAACTAGCAACAGTTTCTGGAAAGTCCCACCTCAGTTTCAAGTTCCCCAAAAGA

+

BBBBBFFFFFFFFFFBFFFFFFFFFFFFFFBFFFFFFFFFFFFFFFFFFFFFFFFFFFFFFFFFFFFFFFFFFFFFFFFFFFFFFFFFFFFFFFFFFFFFFFFFFFFFFFF<FFBBFFFBFFFFFF

@GWZHISEQ02:315:C9E6MANXX:5:2201:8647:61634

AACAGGATATCTGTGGTCGAGCACCTGGGCCCCGGCTCAGGGCCAAGAACAGATGGTACTCAGATAAAGCGAAACTAGCAACAGTTTCTGGAAAGTCCCACCTCAGTTTCAAGTTCCCCAAAAGAC

+

BBBBBFFFFFFFFFFFFFFFFFFFFFFFFFFFFFFFFFFFFFFFFFFFFFFFFFFFFFFFFFFFFFFFFFFFFFFFFFFFFFFFFFFFFFFFFFFFFFFFFFFFFFFFFFFFFFFFFFFFFFFFFF

@GWZHISEQ02:315:C9E6MANXX:5:2213:10020:18336

AACAGGATATCTGTGGTCGAGCACCTGGGCCCCGGCTCAGGGCCAAGAACAGATGGTACTCAGATAAAGCGAAACTAGCAACAGTTTCTGGAAAGTCCCACCTCAGTTTCAAGTTCCCCAAAAGAC

+

BBBBBFFFFFFFFFFFFFFFFFFFFFFFFFFFFFFFFFFFFFFFFFFFFFFFFFFFFFFFFFFFFFFFBFFFFFFFFFFFFFFFFFFFFFFFFFFFFFFFFFFFFFF<FFFFFFFFFFFFBFFFFF

@GWZHISEQ02:315:C9E6MANXX:5:1112:16815:79486

ACAGGATATCTGTGGTCGAGCACCTGGGCCCCGGCTCAGGGCCAAGAACAGATGGTACTCAGATAAAGCGAAACTAGCAACAGTTTCTGGAAAGTCCCACCTCAGTTTCAAGTTCCCCAAAAGACC

+

BBBBBFFFFFFBFFFFBFFFFFFFFFFFF<FBFFF<BFFFFFFBFFFFBF<BFFFFFFFFBFFFFFFFFFFFFBFFFBFFBFB/FBFFFFFFFFFFFFBFFFFFF/BFFFBFBF/F/FB<FFFFFB

@GWZHISEQ02:315:C9E6MANXX:5:1115:8837:78257

ACAGGATATCTGTGGTCGAGCACCTGGGCCCCGGCTCAGGGCCAAGAACAGATGGTACTCAGATAAAGCGAAACTAGCAACAGTTTCTGGAAAGTCCCACCTCAGTTTCAAGTTCCAGATCGGAAG

+

BBBBBFFFFFFFFFFFFFFFFFFFFFFFFFFFFFFFFFFFFFFFFFFFFFFFFFFFFFFFFFFFFFFFFFFFFFFFFFFFFFFFFFFFFFFFFFFFFFFFFFFFFFFFFFFFFFFFBFFFFFFFFF

@GWZHISEQ02:315:C9E6MANXX:5:1208:10539:25233

ACAGGATATCTGTGGTCGAGCACCTGGGCCCCGGCTCAGGGCCAAGAACAGATGGTACTCAGATAAAGCGAAACTAGCAACAGTTTCTGGAAAGTCCCACCTCAGTTTCAAGTTCCCCAAAAGACC

+

BBBBBFFFFFFFFFFFFFFFFFFFFFFFFFFFFFFFFFFFFFFFFFFFFFFFFFFFFFFFFFFFFFFFFFFFFFFFFFFFFFFFFFFFFFFFFFFFFFFFFFFFFFFBFFFFFFFFFFFFFFFFFF

@GWZHISEQ02:315:C9E6MANXX:5:2116:11447:52196

ACAGGATATCTGTGGTCGAGCACCTGGGCCCCGGCTCAGGGCCAAGAACAGATGGTACTCAGATAAAGCGAAACTAGCAACAGTTTCTGGAAAGTCCCACCTCAGTTTCAAGTTCCCCAAAAGACC

+

BBBBBFFFFFFFFBFFFFFFFFFFFFFFFFFFFBFFFFFFFFFFFBFFFFFFFFFFFFFFFFFFFFFFFFFFFFFFFFFFFFF7FFFFFFFFFFFFFFFFFBFFB<FFFFFFFFFFFFFFFFFFFF

@GWZHISEQ02:315:C9E6MANXX:5:2211:13280:65644

ACAGGATATCTGTGGTCGAGCACCTGGGCCCCGGCTCAGGGCCAAGAACAGATGGTACTCAGATAAAGCGAAACTAGCAACAGTTTCTGGAAAGTCCCACCTCAGTTTCAAGTTCCCAGATCGGAA

+

/BBBBBFFFFFFBFFFFFB<BFFFFFFFFFBBBB/FFFFFFFFFFFBFFFFFFFFFFFFFFFFFFFFFFF<FFFFFFFFFFFFFFBFFFFBFFFFFFFFFFFFBFFFFFFFFBFFFFBFFFFFF<B

@GWZHISEQ02:315:C9E6MANXX:5:1115:8837:78257

GGAACTTGAAACTGAGGTGGGACTTTCCAGAAACTGTTGCTAGTTTCGCTTTATCTGAGTACCATCTGTTCTTGGCCCTGAGCCGGGGCCCAGGTGCTCGACCACAGATATCCTGTAGATCGGAAG

+

BBBBBFFFFFFFFFFFFFFFFFFFFFFFFFFFFFFFFFFFFFFFFFFFFFFFFFFFFFFFFFFFFFFFFFFFFFFFFFFFFFFFFFFFFFFBFFFFFFFFFFFFFFFFFFFFFFFFFFFFFF<FFB

@GWZHISEQ02:315:C9E6MANXX:5:2211:13280:65644

GGGAACTTGAAACTGAGGTGGGACTTTCCAGAAACTGTTGCTAGTTTCGCTTTATCTGAGTACCATCTGTTCTTGGCCCTGAGCCGGGGCCCAGGTGCTCGACCACAGATATCCTGTAGATCGGAA

+

B///B/BBFFFFFFFFFFFFBFFFFFFFFFB/FBFFFFFFFBBFFFFFFFFBF<FFF</F/</BFFFFFFFFF<FBFFF<FFFFFFFFFFFB7<BFFFFFFBBF<FFFFFFFFFFBFFBFFFFF<B

@GWZHISEQ02:315:C9E6MANXX:5:1103:9561:81366

CAGGATATCTGTGGTCGAGCACCTGGGCCCCGGCTCAGGGCCAAGAACAGATGGTACTCAGATAAAGCGAAACTAGCAACAGTTTCTGGAAAGTCCCACCTCAGTTTCAAGTTCCCAGATCGGAAG

+

BBBBBFFFFFFFFFFFFFFFFFFFFFFFFFFFFFFFFFFFFFFFFFFFFFFFFFFFFFFFFFFFFFFFFBFFFFFFFFFFFFFFFFFFFFFFFFFFFFFFFFFFFFFFFFFFFFFFFFFFFFFFBF

@GWZHISEQ02:315:C9E6MANXX:5:1105:3431:43401

CAGGATATCTGTGGTCGAGCACCTGGGCCCCGGCTCAGGGCCAAGAACAGATGGTACTCAGATAAAGCGAAACTAGCAACAGTTTCTGGAAAGTCCCACCTCAGTTTCAAGTTCCCCAAAAGACCG

+

BBBBBFFFFFFFFFFFFFFFFFFFFFFFFFFFFFFFFFFFFFFFFFFFFFFFFFFFFFFFFFFFFFFFFFFFFFFFFBFFFFFFFFFFFFFFFFFFFFFFFFFFF/FFFFFFFFFFFFFFFFFFFF

@GWZHISEQ02:315:C9E6MANXX:5:1109:13409:37214

CAGGATATCTGTGGTCGAGCACCTGGGCCCCGGCTCAGGGCCAAGAACAGATGGTACTCAGATAAAGCGAAACTAGCAACAGTTTCTGGAAAGTCCCACCTCAGTTTCAAGTTCCCCAAAAGACCG

+

BBBBBFFFFFFFFFFFFFFFFFFFFFFFFFFFFFFFFFFFFFFFFFFFFFFFFFFFFFFFFFFFFFFFFFFFFFFFFFFFFFFFFFFFFFFFFFFFFFFFFFFFFFFFFFFFFFFFFFFFFFFFFF

@GWZHISEQ02:315:C9E6MANXX:5:1115:5679:10011

CAGGATATCTGTGGTCGAGCACCTGGGCCCCGGCTCAGGGCCAAGAACAGATGGTACTCAGATAAAGCGAAACTAGCAACAGTTTCTGGAAAGTCCCACCTCAGTTTCAAGTTCCCCAAAAGACCG

+

<</BB/FF<F/</<<FB<BFBFFF/<F/<<<<B<FFF//FBFFF/FFF<F/FFFFFFB/FF<BBFFFF<B//<BFBFFB/FBFFBBFFBF<BB7BBFFFFFF/<<//BFBFBFFFBFBBF//BBFF

@GWZHISEQ02:315:C9E6MANXX:5:1204:14033:10006

CAGGATATCTGTGGTCGAGCACCTGGGCCCCGGCTCAGGGCCAAGAACAGATGGTACTCAGATAAAGCGAAACTAGCAACAGTTTCTGGAAAGTCCCACCTCAGTTTCAAGTTCCCCAAAAGACCG

+

BBBBBFFFFFFFFFFFFFFFFFFFFFFFFFFFFFFFFFFFFFFFFFFFFFFFFFFFFFFFFFFFFFFFFBBFFFFFFFFFFFFFFFFFFFFFFFFFFFFFFFFFFFFFFFFFFFFFFFFFFFFFFF

@GWZHISEQ02:315:C9E6MANXX:5:1207:16651:87437

CAGGATATCTGTGGTCGAGCACCTGGGCCCCGGCTCAGGGCCAAGAACAGATGGTACTCAGATAAAGCGAAACTAGCAACAGTTTCTGGAAAGTCCCACCTCAGTTTCAAGAGATCGGAAGAGCGT

+

BBBBBBFFFFFFFFFFFFFFFFFFFFFFFFFFFFFBFFFFFFFFFFFFFFFFFFFFFFFFFFFFFFFFFFFFFFFFFFFFFFFFFFFFFFFFFFFFFFFFFFFFFFFFFFFFFFFFFFFFFFFFFF

@GWZHISEQ02:315:C9E6MANXX:5:1211:11759:31406

CAGGATATCTGTGATCGAGCACCTGGGCCCCGGCTCAGGGCAAAGAACAGATGTTATTCAGATAAAGCGAAACTAGCAACAGTTTCTGGAAAGTCCCCCCTCAGTTTCAAGTTCCCCAAAAGACCG

+

BBBBBFB/</FF///</B////<//<///</<B/</<B//<///<FF/<//<//<//B/</<</<FF/F//////<</</BBFF7<7/FFBF/7<F//</FB///7FF/<F//7/FF/BF/7B//7

@GWZHISEQ02:315:C9E6MANXX:5:1308:6274:94727

CAGGATATCTGTGGTCGAGCACCTGGGCCCCGGCTCAGGGCCAAGAACAGATGGTACTCAGATAAAGCGAAACTAGCAACAGTTTCTGGAAAGTCCCACCTCAGTTTCAAGTTCCCCAAAAGACCG

+

BBBBBFFFFFFFFFFFFF/FFFFFFFFFFFFFFFFFFFFFFFFFFFFFFFFFFFFFFFFFFFFFFFFFFFBFFBFFFFFFFFFFFFFFFFFFFFFFFFFFFFFFFFFFFBFFFFFFFFFFFFFFFB

@GWZHISEQ02:315:C9E6MANXX:5:1311:18126:73550

CAGGATATCTGTGGTCGAGCACCTGGGCCCCGGCTCAGGGCCAAGAACAGATGGTACTCAGATAAAGCGAAACTAGCAACAGTTTCTGGAAAGTCCCACCTCAGTTTCAAGTTCCCCAAAAGACCG

+

BBBBBFFFFFFFFFFFFBBBBFFFFFFFFFFFFFFFFFFFFFFFFFFFFFFBFFFFFFFFFFFFFFFFFFFFFFFFFFFFFFFFFFFFFFFFFFFFFFFFFFFFFFFBF<B<FFFFFFFFFFFFFF

@GWZHISEQ02:315:C9E6MANXX:5:1316:18288:52633

CAGGATATCTGTGGTCGAGCACCTGGGCCCCGGCTCAGGGCCAAGAACAGATGGTACTCAGATAAAGCGAAACTAGCAACAGTTTCTGGAAAGTCCCACCTCAGTTTCAAGTTCCCCAAAAGACCG

+

BBBBBFFFFFFFFFFFFFFFFFFFFFFFFFFFFFFFFFFFFFFFFFFFFFFFFFFFFFFFFFFFFFFFFFFFFFFFFFFFFFFFFFFFFFFFFFFFFFFFFFFFFFFFFFFFFFFFFFFFFFFFFF

@GWZHISEQ02:315:C9E6MANXX:5:2102:3884:21063

CAGGATATCTGTGGTCGAGCACCTGGGCCCCGGCTCAGGGCCAAGAACAGATGGTACTCAGATAAAGCGAAACTAGCAACAGTTTCTGGAAAGTCCCACCTCAGTTTCAAGTTCCCCAAAAGACCG

+

BBBBBFFFFFFFFFFFFFFFFFFFFFFFFFFFFFFFFFFBFFFFFFFFFFFFFFFFFFFFFFFFFFFFFFFFFFFFFFFFFFFFFFFFFFFFFFFFFFFFFFFFFFFFFFFFFFFFFFFFFFFFFF

@GWZHISEQ02:315:C9E6MANXX:5:2203:3544:53951

CAGGATATCTGTGGTCGAGCACCTGGGCCCCGGCTCAGGGCCAAGAACAGATGGTACTCAGATAAAGCGAAACTAGCAACAGTTTCTGGAAAGTCCCACCTCAGTTTCAAGTTCCCCAAAAGACCG

+

BBBBBFFFFFFFFFFFFFFFFFFFFFFFFFFFFFFFFFFFFFFFFFFFFFFFFFFFFFFFFFFFFFFFFFFFFFFFFFFFFFFFFFFFFFFFFFFFFFFFFFFFBFFFFFFFFFFFFFFFFFFFFF

@GWZHISEQ02:315:C9E6MANXX:5:2203:14901:58192

CAGGATATCTGTGGTCGAGCACCTGGGCCCCGGCTCAGGGCCAAGAACAGATGGTACTCAGATAAAGCGAAACTAGCAACAGTTTCTGGAAAGTCCCACCTCAGTTTCAAGTTCCCCAAAAGACCG

+

BBBBBFFFFFFFFFFFFFFFFFFFFFFFBFFFBFF<<FBBFFFBBFFFFFBFFFFFFFFBFFFBFFFFFFFFFFFFFFFFFFFFFFBFFFFFFFFFFFFFFFFFFFFFFFFFFBFFFFBFFFFFFF

@GWZHISEQ02:315:C9E6MANXX:5:2211:20272:13631

CAGGATATCTGTGGTCGAGCACCTGGGCCCCGGCTCAGGGCCAAGAACAGATGGTACTCAGATAAAGCGAAACTAGCAACAGTTTCTGGAAAGTCCCACCTCAGTTTCAAGTTCCCCAAAAGACCG

+

BBBBBFFFFFFFFFFFBFFFFFFFFFFFFFFFFBBFFFFFFFFFFFFFFFFFFFFFFFFFFFFFFFFFFFFFFFFFFFFFFFFFFFFFFFFFFFFFFFFFFFFFFBFFFFFF/FFFFF/FFFFFFF

@GWZHISEQ02:315:C9E6MANXX:5:2308:19649:94326

CAGGATATCTGTGGTCGAGCACCTGGGCCCCGGCTCAGGGCCAAGAACAGATGGTACTCAGATAAAGCGAAACTAGCAACAGTTTCTGGAAAGTCCCACCTCAGTTTCAAGTTCCCCAAAAGACCG

+

BBBBBFFFFFFFFFFFFFFFFFFFFFFFFFFFFFFFFFFFFFFFFFFFFFFFFFFFFFFFFFFFFFFFFFFFFFFFFFFFFFFFFFFFFFFFFFFFFFFFFFFFFFFFFFFFFFFFFFFFFFFFFF

@GWZHISEQ02:315:C9E6MANXX:5:1103:9561:81366

GGGAACTTGAAACTGAGGTGGGACTTTCCAGAAACTGTTGCTAGTTTCGCTTTATCTGAGTACCATCTGTTCTTGGCCCTGAGCCGGGGCCCAGGTGCTCGACCACAGATATCCTGAGATCGGAAG

+

BBBBBFFFFFFFFFFFFFFFFFFFFFFFFFFFFFFFFFFFFFFFFFFFFFFFFFFFFFFFFFFFFFFFFFFFFFFFFFFFFFFFFFFFFFFFBFFFFFFFFFFFFFFFFFFFFFFFFFFFFFFFFF

@GWZHISEQ02:315:C9E6MANXX:5:1109:12719:33839

CGGTCTTTTGGGGAACTTGAAACTGAGGTGGGACTTTCCAGAAACTGTTGCTAGTTTCGCTTTATCTGAGTACCATCTGTTCTTGGCCCTGAGCCGGGGCCCAGGTGCTCGACCACAGATATCCTG

+

<B/</<BFBFFFFFF/<FBBBFBBFFFBFB<F/FFF<<BF//F//BFFF/BBFFFFFFF<FFF<F<F/</FBFFFFFFFFFBFFBBFF/BF/<FFF</B</<BFF/BFFF<FF/B<FFFF<FBBBF

@GWZHISEQ02:315:C9E6MANXX:5:1207:16651:87437

CTTGAAACTGAGGTGGGACTTTCCAGAAACTGTTGCTAGTTTCGCTTTATCTGAGTACCATCTGTTCTTGGCCCTGAGCCGGGGCCCAGGTGCTCGACCACAGATATCCTGAGATCGGAAGAGCAC

+

BBBBBFFFFFFFFFFFFFFFFFFFFFFFFFFFFFFFFFFFFFFFFFFFFFFFFFFFFFFFFFFFFFFFFFFFFFFFBFFFFFFFFFFFFFFFFFFFFFFFFFFFFFFFFFFFFFFFFFFFFFFFFF

@GWZHISEQ02:315:C9E6MANXX:5:2204:19857:46791

CGGTCTTTTGGGGAACTTGAAACTGAGGTGGGACTTTCCAGAAACTGTTGCTAGTTTCGCTTTATCTGAGTACCATCTGTTCTTGGCCCTGAGCCGGGGCCCAGGTGCTCGACCACAGATATCCTG

+

BBBBBFFFFFFFFFFFFFFFFFFFFFFFFFFFFFFFFFFF<FFFFFFFFFFFFFFFFFFFFFFFFFFFFFFFFFFFFFFFFFFFFFFFFFFFFFFFFFFFFFFFFFFFFFFFFFBBFFBFFFFFFF

@GWZHISEQ02:315:C9E6MANXX:5:2209:16304:89579

CGGTCTTTTGGGGAACTTGAAACTGAGGTGGGACTTTCCAGAAACTGTTGCTAGTTTCGCTTTATCTGAGTACCATCTGTTCTTGGCCCTGAGCCGGGGCCCAGGTGCTCGACCACAGATATCCTG

+

BBBBBFFFFFFFFFFFFFFFFFFFFFFFFFFFFFFFFFFFFFFFFFFFFFFFFFFFFFFFFFFFFFFFFFFFFFFFFFFFFFFFFFFFFFFFFFFFBFBFFFFFFFFFFFFFFFFFFFBFFFFFFF

@GWZHISEQ02:315:C9E6MANXX:5:2211:3595:63158

CTTTTGGGGAACTTGAAACTGAGGTGGGACTTTCCAGAAACTGTTGCTAGTTTCGCTTTATCTGAGTACCATCTGTTCTTGGCCCTGAGCCGGGGCCCAGGTGCTCGACCACAGATATCCTGAGAT

+

BBBBB<///FFFFF<FBFFFFBFFFFFBFFFFFFFFFFFBFFFFFFFFFBFFFFFFFFFFFFFFF/FFF<<FFFFFFFFFFBFBFFFFFFFFFFFF<<BFFFFFFFFFFFFFBF<BBFFFFFFFFF

@GWZHISEQ02:315:C9E6MANXX:5:1205:16918:32046

AGGATATCTGTGGTCGAGCACCTGGGCCCCGGCTCAGGGCCAAGAACAGATGGTACTCAGATAAAGCGAAACTAGCAACAGTTTCTGGAAAGTCCCACCTCAGTTTCAAGTTCCCCAAAAGACCGG

+

BBBBBFFFFFFFFFFFFFFFFFFFFFFFFFFFFFFFFFFFFFFFFFFFFFFFFFFFFFFFFFFFFFFFFFFFFFFFFFFFFFFFFFFFFFFFFFFFFFFFFFFFFFFFFFFFFFFFFFFFFFFFFF

@GWZHISEQ02:315:C9E6MANXX:5:1208:3320:91728

AGGATATCTGTGGTCGAGCACCTGGGCCCCGGCTCAGGGCCAAGAACAGATGGTACTCAGATAAAGCGAAACTAGCAACAGTTTCTGGAAAGTCCCACCTCAGTTTCAAGTTCCCCAAAAGACCGG

+

BBBBBFFFFFFFFFFFFFFFFFFFFFFFFFFFFFFFFFFFFFFFFFFFFFFFFFFFFFFFFFFFFFFFFFFFFFFFFFFFFFFFFFFFFFBFFFFFFFFFFFFFFFFFFFFFFBFFF<BFFFFFFF

@GWZHISEQ02:315:C9E6MANXX:5:1213:16634:84767

AGGATATCTGTGGTCGAGCACCTGGGCCCCGGCTCAGGGCCAAGAACAGATGGTACTCAGATAAAGCGAAACTAGCAACAGTTTCTGGAAAGTCCCACCTCAGTTTCAAGTTCCCCAAAAGACCGG

+

BBBBBFFFFFFFFFFFFFFFFFFFFFFFFFFFFFFFFFFFFFFFFFFFFFFFFFFFFFFFFFFFFFFFFFFFFFFFFFFFFFFFFFFFFFFFFFFFFFFFFFFFBFFFFFFFFFFFFFFFFFFFFF

@GWZHISEQ02:315:C9E6MANXX:5:1303:17600:6395

AGGATATCTGTGGTCGAGCACCTGGGCCCCGGCTCAGGGCCAAGAACAGATGGTACTCAGATAAAGCGAAACTAGCAACAGTTTCTGGAAAGTCCCACCTCAGTTTCAAGTTCCCCAAAAGACCGG

+

BBBBBFFFFFFFFBFFFFFFFFFFFFFFFFFFFFFFFFFFFFFFFFFFFFFFFFFFFFFFFFFFFFFFFFFFFFFFFFFFFFFFFFFFFFFFFFFFFFFFFFFFBFFFFFFFFFFFFFFFFFFFFF

@GWZHISEQ02:315:C9E6MANXX:5:2103:10059:45330

AGGATATCTGTGGTCGAGCACCTGGGCCCCGGCTCAGGGCCAAGAACAGATGGTACTCAGATAAAGCGAAACTAGCAACAGTTTCTGGAAAGTCCCACAGATCGGAAGAGCGTCGTGTAGGGAAAG

+

BBBBBFFFFFFFFFFFFFFFFFFFFFFFFFFFFFFFFFFFFFFFFFFFF<FFFFFFFFFFFFFFFFFFBFFFFFFFFFFFFFFFFFFFFFFFFFFFFFFFFFFFFFFFFFFFFFFBFFFFFFF/FF

@GWZHISEQ02:315:C9E6MANXX:5:2105:18032:11712

AGGATATCTGTGGTCGAGCACCTGGGCCCCGGCTCAGGGCCAAGAACAGATGGTACTCAGATAAAGCGAAACTAGCAACAGTTTCTGGAAAGTCCCACCTCAGTTTCAAGTTCCCCAAAAGACCGG

+

<BBBBFFFFFFFFFFFFFFFFFFFFFFFFFFFFFFFFFFFFFFFFFFFFFFFFFFFFFFFFFFFFFFFFFFFFFFFFFFFFFFFFFFFFFFFFFFFFFFFFFFFFFFFFFFFFFFFFFFFFFFFFF

@GWZHISEQ02:315:C9E6MANXX:5:1210:3182:15340

CTGGTCTTTTGGGGAACTTGAAACTGAGGTGGGACTTTCCAGAAACTGTTGCTAGTTTCGCTTTATCTGAGTACCATCTGTTCTTGGCCCTGAGCCGGGGCCCAGGTGCTCGACCACAGATATCCT

+

BBBBBFFFFFFFFFFFFFFFFFFFFFFFFFFFFFFFFFFFFFFFFFFFFFFFFFFFFFFFFFFFFFFFFFFFFFFFFFFFFFFFFFFFFFFFFFFFFFFFFFFFFFFFFFFFFFFFFFFFFFFFFF

@GWZHISEQ02:315:C9E6MANXX:5:1210:20123:67521

CCGGTCTTTTGGGGAACTTGAAACTGAGGTGGGACTTTCCAGAAACTGTTGCTAGTTTCGCTTTATCTGAGTACCATCTGTTCTTGGCCCTGAGCCGGGGCCCAGGTGCTCGACCACAGATATCCT

+

BBBBBBFFFFFFFFFFFFFFFFFFFFFFFFFFFFFFFFFFFFFFFFFFFFFFFFFFFFFFFFFFFFFFFFFFFFFFFFFFFFFFFFFFFFFFFFFFFFFFFFFFFFBFFFFFFFFFFFFFFFFFFF

@GWZHISEQ02:315:C9E6MANXX:5:1210:18688:73023

CCGGTCTTTTGGGGAACTTGAAACTGAGGTGGGACTTTCCAGAAACTGTTGCTAGTTTCGCTTTATCTGAGTACCATCTGTTCTTGGCCCTGAGCCGGGGCCCAGGTGCTCGACCACAGATATCCT

+

B/BBBFFFFFF<BBFFFFFFFFFFFFFFFFFFFFFFFFFFFFFFFFFFFFFFFFFFFFFFFFBFFFFFFFFFFFFFFFFF<BFFF<BBFFFFFFFFFFFFFFFFFFFFFFFFFFFFFFFFFFFFF/

@GWZHISEQ02:315:C9E6MANXX:5:1306:7167:2741

CCGGTCTTTTGGGGAACTTGAAACTGAGGTGGGACTTTCCAGAAACTGTTGCTAGTTTCGCTTTATCTGAGTACCATCTGTTCTTGGCCCTGAGCCGGGGCCCAGGTGCTCGACCACAGATATCCT

+

BBBBBFFFFFFFFFFFFFFFFFFFFFFFFBFFFFFFFFFFFFFFFFFFFFFFFFFFFFFFFFFFFFFFFFFFFFFFFFFFFFFFFFFFFFFFFFFFFBFFFFFFFFFFFFFFFFF/FFFFFFFFFB

@GWZHISEQ02:315:C9E6MANXX:5:1307:2898:6339

CCGGTCTTTTGGGGAACTTGAAACTGAGGTGGGACTTTCCAGAAACTGTTGCTAGTTTCGCTTTATCTGAGTACCATCTGTTCTTGGCCCTGAGCCGGGGCCCAGGTGCTCGACCACAGATATCCT

+

BBBBBFFFFFFFFFFFFFFFFFFFFFFFFFFFFFFFFFFFFFFFFFFFFFFFFFFFFFFFFFFFFFFFFFFFFFFFFFFFFFFFFFFFFFFFFFFFFFFFFFFFFFFFFFFFFFFFFFFFFFFFFB

@GWZHISEQ02:315:C9E6MANXX:5:1309:9686:47321

CCGGTCTTTTGGGGAACTTGAAACTGAGGTGGGACTTTCCAGAAACTGTTGCTAGTTTCGCTTTATCTGAGTACCATCTGTTCTTGGCCCTGAGCCGGGGCCCAGGTGCTCGACCACAGATATCCT

+

BBBBBFFFFFFFBFFFFFFFFFFFFFFFFFFFFFFFFFFFFFFFFFFFFFFFFFFFFFFFFFFFFFFFFFFFFFFFFFFFFFFFFFFFFFFFFFFFFFFFFFFFFFFFFFFFBF<7BFFBFFFFFF

@GWZHISEQ02:315:C9E6MANXX:5:1310:7666:73426

CCGGTCTTTTGGGGAACTTGAAACTGAGGTGGGACTTTCCAGAAACTGTTGCTAGTTTCGCTTTATCTGAGTACCATCTGTTCTTGGCCCTGAGCCGGGGCCCAGGTGCTCGACCACAGATATCCT

+

BBBBBFFFFFFFFFFFFFF<FFFFFFFFFFBFFFFFFFFFFF<FFFFFFFFFFFFFFFFFFFFFFFFFFFBFBFBFFFFFFFFFB//FFFFFFFFFFFFBFFFFFFFFFFFFFFFFFFBFFFFFF/

@GWZHISEQ02:315:C9E6MANXX:5:2103:10059:45330

GTGGGACTTTCCAGAAACTGTTGCTAGTTTCGCTTTATCTGAGTACCATCTGTTCTTGGCCCTGAGCCGGGGCCCAGGTGCTCGACCACAGATATCCTAGATCGGAAGAGCACACGTCTGAACTCC

+

BBBBBFFFFFFFFFFFFFFFFFFFFFFFFFFFFFFFFFFFFFFFFFFFFFFFFFFFFFFFFFFFFFFFFFFFFFFFFFFFFFFFFFFFFFFFFFFFFFFFFFFFFFFFFFFFFFFFBFFFFFFFFF

@GWZHISEQ02:315:C9E6MANXX:5:2114:7225:92847

CCGGTCTTTTGGGGAACTTGAAACTGAGGTGGGACTTTCCAGAAACTGTTGCTAGTTTCGCTTTATCTGAGTACCATCTGTTCTTGGCCCTGAGCCGGGGCCCAGGTGCTCGACCACAGATATCCT

+

BBBBBFFFFFFFFFFFFFFFFFFFFFFFFFFFFFFFBFFFFFFFFFFFFFFFFFFBFFFFFFFFFFFFFFFFFFFFFFFFFFFFFFFFFFFFFFFFFFFFFFFFFFFFFFFFFFFFFFFFFFFFFB

@GWZHISEQ02:315:C9E6MANXX:5:2201:13492:44700

CCGGTCTTTTGGGGAACTTGAAACTGAGGTGGGACTTTCCAGAAACTGTTGCTAGTTTCGCTTTATCTGAGTACCATCTGTTCTTGGCCCTGAGCCGGGGCCCAGGTGCTCGACCACAGATATCCT

+

BBBBBFFFFFFFFFFFFFFFFFFFFFFFFFFFFFFFFFFFFFFFFFFFFFFFFFFFFFFFFFFFFFFFFFFFFFFFFFFFFFFFFFFFFFFFFFFFFFFFFFFFFFFFFFFFFFFFFFFFFFFFFF

@GWZHISEQ02:315:C9E6MANXX:5:2208:11802:14509

CCGGTCTTTTGGGGAACTTGAAACTGAGGTGGGACTTTCCAGAAACTGTTGCTAGTTTCGCTTTATCTGAGTACCATCTGTTCTTGGCCCTGAGCCGGGGCCCAGGTGCTCGACCACAGATATCCT

+

BBBBBFFFFFFFFFFFFFFFFFFFFFFFFFFFFFFFFFFFFFFFFFFFFFFFFFFFFFFFFFFFFFFFFFFFFFFFFFFFFFFFFFFFFFFFFFFFFFFFFFFFFFFFFFFFFFFFFFFFFFFFFB

@GWZHISEQ02:315:C9E6MANXX:5:2305:14829:12933

CCGGTCTTTTGGGGAACTTGAAACTGAGGTGGGACTTTCCAGAAACTGTTGCTAGTTTCGCTTTATCTGAGTACCATCTGTTCTTGGCCCTGAGCCGGGGCCCAGGTGCTCGACCACAGATATCCT

+

BBBBBFFFFFFFFFFFFFFFFFFFFFFFFFFFFFFFFFFFFFFFFFFFFFFFFFFFFFFFFFFFFFFFFFFFFFFFFFFFFFFFFFFFFFFF<FFFFFFFFFFBFFFFFFFFFFFFFFFFFFFFFF

@GWZHISEQ02:315:C9E6MANXX:5:2307:14537:37655

CCGGTCTTTTGGGGAACTTGAAACTGAGGTGGGACTTTCCAGAAACTGTTGCTAGTTTCGCTTTATCTGAGTACCATCTGTTCTTGGCCCTGAGCCGGGGCCCAGGTGCTCGACCACAGATATCCT

+

B<BBB<</FBB<///<//<FF/FBF//<FF//F/B/FFFFF<//<F<BFF////<<BBFFFFFFFFFFFFFBBFBFF<FFFFFFBFF</<FB//<//</<FFB7FFFFFFBFF<BFFFFFF77BB/

@GWZHISEQ02:315:C9E6MANXX:5:2307:19349:73564

CCGGTCTTTTGGGGAACTTGAAACTGAGGTGGGACTTTCCAGAAACTGTTGCTAGTTTCGCTTTATCTGAGTACCATCTGTTCTTGGCCCTGAGCCGGGGCCCAGGTGCTCGACCACAGATATCCT

+

BBBBBFFFFFFFFFFFFFFFFFFFFFFFFFFFFFFFFFFFFFFFFFFFFFFFFFFFFFFFFFFFFFFFFFFFFFFFFFFFFFFFFFFFFFFFFFFFFFFBFFFFFFFFFFFFFFFFFFFFFFFFFF

@GWZHISEQ02:315:C9E6MANXX:5:2310:3187:4304

GGGGAACTTGAAACTGAGGTGGGACTTTCCAGAAACTGTTGCTAGTTTCGCTTTATCTGAGTACCATCTGTTCTTGGCCCTGAGCCGGGGCCCAGGTGCTCGACCACAGATATCCTAGATCGGAAG

+

BBBBBFFFFFFFFFFFFFFFFFFFFFFFFFFFFFFFFFFFFFFFFFFFFFFFFFFFFFFFFFFFFFFFFFFFFFFFFFFFFFFFFFFFFFFFFFFFFFFFFFFFBFFFFFFFFFFFFFFFFFFFFF

@GWZHISEQ02:315:C9E6MANXX:5:2316:20431:42256

CCGGTCTTTTGGGGAACTTGAAACTGAGGTGGGACTTTCCAGAAACTGTTGCTAGTTTCGCTTTATCTGAGTACCATCTGTTCTTGGCCCTGAGCCGGGGCCCAGGTGCTCGACCACAGATATCCT

+

BBBBBFFFFFFFFFFFFFFFFFFFFFFFFFFFFFFFFFFFFFFFFFFFFFFFFFFFFFFFFFFFFFFFFFFFFFFFFFFFFFFFFFFFFFFFFFFFFFFFFFFFFFFFFFFFFFFFFFFFFFFFFF

@GWZHISEQ02:315:C9E6MANXX:5:2316:6146:55622

CCGGTCTTTTGGGGAACTTGAAACTGAGGTGGGACTTTCCAGAAACTGTTGCTAGTTTCGCTTTATCTGAGTACCATCTGTTCTTGGCCCTGAGCCGGGGCCCAGGTGCTCGACCACAGATATCCT

+

BBBBB<FFFFFFFFFFFFFFFFFFFFFFFFFFFFFFFFFFFFFFFFFFFFFFFFFFFFFFFFFFFFFFFFFFFFFFFFFFFFFFFFFFFFFFFFFFFFFFFFFFFFFFFFFFFFFFF<FBFFFFFF

@GWZHISEQ02:315:C9E6MANXX:5:2110:4155:78371

GGATATCTGTGGTCGAGCACCTGGGCCCCGGCTCAGGGCCAAGAACAGATGGTACTCAGATAAAGCGAAACTAGCAACAGTTTCTGGAAAGTCCCACCTCAGTTTCAAGTTCCCCAAAAGACCGGG

+

BBB/BFFFFFFFFFFFFFFFFFFFFFFFFFFFFFFFFFFFFFFFFFFF<FFFFFFFFFFFFFFFFFFFFFFFFFFFFFFFBFFFFFFFFFFFFFFFFFFFFFFFFFFFFFFFFFFFFFFFFFFBFF

@GWZHISEQ02:315:C9E6MANXX:5:2111:3925:43223

GGATATCTGTGGTCGAGCACCTGGGCCCCGGCTCAGGGCCAAGAACAGATGGTACTCAGATAAAGCGAAACTAGCAATAGTTTCTGGAAAGTCCCACCTCAGTTTCAAGTTCCCCAAAAGACCGGG

+

BBBBBFFFFFFBFFFFFFFFFFF<BFFFFFFFFFFFFFFFFFFFFFFF<FFFFBFFFFFFFFFFFFFFFFFFFFFFFFFFFFFFFF<FFFFFFFFFFFFFFFFFFFFFFBFFFFFFBFFFFFFFFF

@GWZHISEQ02:315:C9E6MANXX:5:2201:8020:66515

GGATATCTGTGGTCGAGCACCTGGGCCCCGGCTCAGGGCCAAGAACAGATGGTACTCAGATAAAGCGAAACTAGCAACAGTTTCTGGAAAGTCCCACCTCAGTTTCAAGTTCCCAGATCGGAAGAG

+

BBBBBFFFFFFFFFFFFFFFFFFFFFFFFFFFFFFFFFFFFFFFFFFFFFFFFFFFFFFFFFFFFFFFFFFFFFFFFFFFFFFFFFFFFFFFFFFFFFFFFFFFFFFFFFFFFFFFFFFFFFFFFF

@GWZHISEQ02:315:C9E6MANXX:5:2302:14053:79565

GGATATCTGTGGTCGAGCACCTGGGCCCCGGCTCAGGGCCAAGAACAGATGGTACTCAGATAAAGCGAAACTAGCAACAGTTTCTGGAAAGTCCCACCTCAGTTTCAAGTTCCCCAAAAGACCGGG

+

BBBBBFFFFFFFFFFFFFFFFFFFFFFFFFFFFFFFFFFFFFFFFFFFFFFFFFFFFFFFFFFFFFFFFFFFFFFFFFFFFFFFFFFFFFFFFFFFFFFFFFFFFFFFFFFFFFFFFFFFFFFFFF

@GWZHISEQ02:315:C9E6MANXX:5:2307:14468:45567

GGATATCTGTGGTCGAGCACCTGGGCCCCGGCTCAGGGCCAAGAACAGATGGTACTCAGATAAAGCGAAACTAGCAACAGTTTCTGGAAAGTCCCACCTCAGTTTCAAGTTCCCCAAAAGACCGGG

+

BBBBBFFFFFFFFFFFFFFFFFFFFFFFFFFFFFFFFFFFFFFFFFFFFFFFFFFFFFFFFFFFFFFFFFFFFFFFFFFFFFFFFFFFFFFFFFFFFFFFFF<FFFFFFFFFFFFFFFFFFFFFFB

@GWZHISEQ02:315:C9E6MANXX:5:1111:9159:93783

CCCGGTCTTTTGGGGAACTTGAAACTGAGGTGGGACTTTCCAGAAACTGTTGCTAGTTTCGCTTTATCTGAGTACCATCTGTTCTTGGCCCTGAGCCGGGGCCCAGGTGCTCGACCACAGATATCC

+

BBBBBFFFFFFFFFBFFFFFFFFFFFFFFFBFFFFFFFFFFFFFBFFFBFFFFFFFFFFFFFFFFBFFFFFFFFFFFFFFFFFFFFFBFFFFFFFFFFFFFFFBFFFFFFFFFFFFFFFFFFFFFF

@GWZHISEQ02:315:C9E6MANXX:5:1114:20550:86973

CCCGGTCTTTTGGGGAACTTGAAACTGAGGTGGGACTTTCCAGAAACTGTTGCTAGTTTCGCTTTATCTGAGTACCATCTGTTCTTGGCCCTGAGCCGGAGCCCAGGTGCTCGACCACAGATATCC

+

/<BBBFFFFFFFFFFBFFFFFFFFFFFFFFFFFFFFFFFFFFFFFFFFFFFFFFBFFFFFFFFFFFFFFFFFFFFFFFFFFFFFFFBFFFFFFFFFFFFFFFFFFFFFFFFFFFFBBFFFFF<BFF

@GWZHISEQ02:315:C9E6MANXX:5:1207:12789:6758

CCCGGTCTTTTGGGGAACTTGAAACTGAGGTGGGACTTTCCAGAAACTGTTGCTAGTTTCGCTTTATCTGAGTACCATCTGTTCTTGGCCCTGAGCCGGGGCCCAGGTGCTCGACCACAGATATCC

+

BBBBBFFFFFFFFFFFFFFFFFFFFFFFFFFFFFFFFFFFFFFFFFFFFFFFFFFFFFFFFFFFFFFFFFFFFFFFFFFFFFFFFFFBFFFFFFFFFFFFFFFFFFFFFFFFFFFFFFFFFFFFFF

@GWZHISEQ02:315:C9E6MANXX:5:1213:16826:10618

CCCGGTCTTTTGGGGAACTTGAAACTGAGGTGGGACTTTCCAGAAACTGTTGCTAGTTTCGCTTTATCTGAGTACCATCTGTTCTTGGCCCTGAGCCGGGGCCCAGGTGCTCGACCACAGATATCC

+

BBBBBFFFFFFFFFFFFFFFFFFFFFFFFFFFFFFFFFFFFFFFFFFFFFFFFFFFFFFFFFFFFFFFFFFFFFFFFFFFFFFFFFFFFFFFFFFFFFFFFFFFFFFFFFFFFFFFFFFFFF<FFF

@GWZHISEQ02:315:C9E6MANXX:5:2101:9546:99585

CCCGGTCTTTTGGGGAACTTGAAACTGAGGTGGGACTTTCCAGAAACTGTTGCTAGTTTCGCTTTATCTGAGTACCATCTGTTCTTGGCCCTGAGCCGGGGCCCAGGTGCTCGACCACAGATATCC

+

BBB/BFFFFFFFF/BFBFFFFFFFFFFFFFFFFFFFFFFFFFFF/FFFBFFFFFFFFBFFF<<FFBFBF/BBBFF<F<FFFFFFFF<//<BF<<<FFFFBFFFB<//FFFFF<B7BF<F77B/7FF

@GWZHISEQ02:315:C9E6MANXX:5:2201:7305:18024

CCCGGTCTTTTGGGGAACTTGAAACTGAGGTGGGACTTTCCAGAAACTGTTGCTAGTTTCGCTTTATCTGAGTACCATCTGTTCTTGGCCCTGAGCCGGGGCCCAGGTGCTCGACCACAGATATCC

+

BBBBBFFFFFFFFFFFFFFFFFFFFFFFFFFFFFFFFFFFFFFFFFFFFFFFFFFFFFFFFFFFBFFFFFFFFFFFFFFFFFFFFFFFFFFFFFFFFFFFFFFFFFFFFBFFFFFFFFFFFFFFFF

@GWZHISEQ02:315:C9E6MANXX:5:2201:8020:66515

GGGAACTTGAAACTGAGGTGGGACTTTCCAGAAACTGTTGCTAGTTTCGCTTTATCTGAGTACCATCTGTTCTTGGCCCTGAGCCGGGGCCCAGGTGCTCGACCACAGATATCCAGATCGGAAGAG

+

BBBBBFFFFFFFFFFFFFFFFFFFFFFFFFFFFFFFFFFFFFFFFFFFFFFFFFFFFFFFFFFFFFFFFFFFFFFFFFFFFFFFFFFFBFFFFFFBFBFBFFFFFFFFFFFFFF<F<B7FBBFFF<

@GWZHISEQ02:315:C9E6MANXX:5:2203:3544:53951

CCCGGTCTTTTGGGGAACTTGAAACTGAGGTGGGACTTTCCAGAAACTGTTGCTAGTTTCGCTTTATCTGAGTACCATCTGTTCTTGGCCCTGAGCCGGGGCCCAGGTGCTCGACCACAGATATCC

+

BBBBBFFFFFFFFFFFFFFFFFFFFFFFFFFFFFFFFFFFFFFFFFFFFFFFFFFFFFFFFFFFFFFFFFFFFFFFFFFFFFFFFFFFFFFFFFFFFFFFFFFFFFFFFFFFFFFFFFFFFFFFFF

@GWZHISEQ02:315:C9E6MANXX:5:2213:18766:56929

CCCGGTCTTTTGGGGAACTTGAAACTGAGGTGGGACTTTCCAGAAACTGTTGCTAGTTTCGCTTTATCTGAGTACCATCTGTTCTTGGCCCTGAGCCGGGGCCCAGGTGCTCGACCACAGATATCC

+

BBBBBFFFFFFFFFFFFFFFFFFFFFFFFFFFFFFFFFFFFFFFFFFFFFFFFFFFFFFFFFFFFFFFFFFFFFFFFFFFFFFFFFFFFFFFFFFFFFBFFFFFFFFFFFFFFFFFFFFFFFFFFF

@GWZHISEQ02:315:C9E6MANXX:5:2302:14053:79565

CCCGGTCTTTTGGGGAACTTGAAACTGAGGTGGGACTTTCCAGAAACTGTTGCTAGTTTCGCTTTATCTGAGTACCATCTGTTCTTGGCCCTGAGCCGGGGCCCAGGTGCTCGACCACAGATATCC

+

BBBBBFFFFFFFFFFFFFFFFFFFFFFFFFFFFFFFFFFFFFFFFFFFFFFFFFFFFFFFFFFFFFFFFFFFFFFFFFFFFFFFFFFFFFFFFFFFFFFFFFFFBFFFFFFFFFFFFFFFFFFFFF

@GWZHISEQ02:315:C9E6MANXX:5:2313:19478:76976

CCCGGTCTTTTGGGGAACTTGAAACTGAGGTGGGACTTTCCAGAAACTGTTGCTAGTTTCGCTTTATCTGAGTACCATCTGTTCTTGGCCCTGAGCCGGGGCCCAGGTGCTCGACCACAGATATCC

+

/<B//FBFFFFFFF<BBFFF<F/FFFF/F<//<BBFFFFFFFFB<FFFFFBFB<F<BBBFFFFF<FFBBFFBFFFFFFFFFF<FFFFFFFFFFFFFFFF</<B/BFFFF/FFF/</B/FBB<BBF/

@GWZHISEQ02:315:C9E6MANXX:5:1310:9304:66918

GATATCTGTGGTCGAGCACCTGGGCCCCGGCTCAGGGCCAAGAACAGATGGTACTCAGATAAAGCGAAACTAGCAACAGTTTCTGGAAAGTCCCACCTCAGTTTCAAGTTCCCCAAAAGACCGGGA

+

BBBBBFFFFFFFFFFFFFFFFFFFFFFFFFFFFFBFFBFFFFFFFFFFFFFFFFFFFFFFFFFFFFFFFFFFFBBFFFFFFFFFFFFFFFFFFFFFFFFFFFFFFBFFFFFFFFFFFFFFFFFFFF

@GWZHISEQ02:315:C9E6MANXX:5:1104:12297:96529

GGGAACTTGAAACTGAGGTGGGACTTTCCAGAAACTGTTGCTAGTTTCGCTTTATCTGAGTACCATCTGTTCTTGGCCCTGAGCCGGGGCCCAGGTGCTCGACCACAGAGATCGGAAGAGCACACG

+

BB<BBFFFFFFFFFFFBFFFFFFFFFFFBFFFFFFFFFFFFFFFFFFFFFFFFFFFFFFFFFFFFFFFBFFBFFF<FFFFBFFFFFF<F<FFFFFFFFFFBFFFFFF/BBBFFFBB7BFFBFFFF<

@GWZHISEQ02:315:C9E6MANXX:5:1213:19124:95831

GTGGGACTTTCCAGAAACTGTTGCTAGTTTCGCTTTATCTGAGTACCATCTGTTCTTGGCCCTGAGCCGGGGCCCAGGTGCTCGACCACAGAGATCGGAAGAGCACACGTCTGAACTCCAGTCACA

+

BBBBBFFFFFFFFFFFFFFFFFFFFFFFFFFFFFFFFFFFFFFFFFFFFFFFFFFFFFFFFFFFFFFFFFFFFFFFFFFFFFFFFFFFFFFFFFFFFFFFFFFFFFFFFFFFFFFFFFFFFFFFFF

@GWZHISEQ02:315:C9E6MANXX:5:2106:18164:42605

CCCCGGTCTTTTGGGGAACTTGAAACTGAGGTGGGACTTTCCAGAAACTGTTGCTAGTTTCGCTTTATCTGAGTACCATCTGTTCTTGGCCCTGAGCCGGGGCCCAGGTGCTCGACCACAGATATC

+

BBBBBFFFFFFFFFFFFFFFFFFFFFFFFFFFFFFFFFFFFFFFFFFFFFFFFFFFFFFFFFFFFFFFFFFFFFFFFFFFFFFFFFFFFFFFFFFFFFFFFFFFFFFFFFFFFFFFFFFFFFFFFF

@GWZHISEQ02:315:C9E6MANXX:5:2206:4990:53625

CCCCGGTCTTTTGGGGAACTTGAAACTGAGGTGGGACTTTCCAGAAACTGTTGCTAGTTTCGCTTTATCTGAGTACCATCTGTTCTTGGCCCTGAGCCGGGGCCCAGGTGCTCGACCACAGAGATC

+

BBBBBFFFFFFFFFFFFFFFFFFFFFFFFFFFFFFFFFFFFFFFFFFFFFFFFFFFFFFFFFFFFFFFFFFFFFFFFFFFFFFFFFFBFFFFFFFFFFFFFFFFFFFFFFFFFFFFFFFFFFFFFF

@GWZHISEQ02:315:C9E6MANXX:5:2205:19306:33977

TTCCCGGTCTTTTGGGGAACTTGAAACTGAGGTGGGACTTTCCAGAAACTGTTGCTAGTTTCGCTTTATCTGAGTACCATCTGTTCTTGGCCCTGAGCCGGGGCCCAGGTGCTCGACCACAGATAT

+

BBBBBFFFFFFFFFFFFFFFFFFFFFFFFFFFFFFFFFFFFFFFFFFFFFFFFFFFFFFFFFFFFFFFFFFFFFFFFFFFFFFFFFFFB<FFFFFFFFFFBFFFFFBFBFFFFFFFFFFFFFFFFF

@GWZHISEQ02:315:C9E6MANXX:5:2212:17334:58796

CCCGGTCTTTTGGGGAACTTGAAACTGAGGTGGGACTTTCCAGAAACTGTTGCTAGTTTCGCTTTATCTGAGTACCATCTGTTCTTGGCCCTGAGCCGGGGCCCAGGTGCTCGACCACAGATAGAT

+

BBBBBFFFFFFFFFFFFBBFFFFFFFFFFFFFFFFFFFFFFFBBFFFFFFFFFFFFFBFFBFFFFFFFFFFFFFFFFFFFFFFFFFFFFFFFFFFFFFF<BFB<<BB/BFFFBFBFFF7BFFFFFF

@GWZHISEQ02:315:C9E6MANXX:5:2212:17334:58796

ATCTGTGGTCGAGCACCTGGGCCCCGGCTCAGGGCCAAGAACAGATGGTACTCAGATAAAGCGAAACTAGCAACAGTTTCTGGAAAGTCCCACCTCAGTTTCAAGTTCCCCAAAAGACCGGGAGAT

+

BBBBBFFFFFFFFFFFFFFFFFFFFFFFFFFFFFFFFFFFFFFFFFFFFFFFFFFFFFFFFFFFFFFFFFFFBFFFFFFFFFFFFFFFFFFFFFFFFFFFFFFFFFFFFFFFFFFFFFFFFFBFF7

@GWZHISEQ02:315:C9E6MANXX:5:2211:14665:13185

TGAGTACCATCTGTTCTTGGCCCTGAGCCGGGGCCCAGGTGCTCGACCACAGACTTGGCTCACTGCAATCTCCGCCTCCCCGGTTCAAGCAATTTTCCTGCCTCAGCTTCCTGTGTAGCTGGGATT

+

//B/B/B<FBFFFFFFFF/<BFFFBB/BFBBB/F<BBFFFFBFB</FF//<<B<<<B/FB<<FFFFFFFFF/BFFFFF/FFFFFFFFFFFFFFFFFFFFFFFFFFFFFFFFFFBBFFFFFF<<FFF

@GWZHISEQ02:315:C9E6MANXX:5:1102:19783:8401

CTGTGGTCGAGCACCTGGGCCCCGGCTCAGGGCCAAGAACAGATGGTACTCAGATAAAGCGAAACTAGCAACAGTTTCTGGAAAGTCCCACCTCAGTTTCAAGTTCCCCAAAAGACCGGGAAAAAC

+

BBBBBFFFFFBB/BF/<F/FFFFFF/FFFFB</FBFF<FFFFF<FBF/<</B<<FFFBFBFFFFFFFBFFFFBB<<BFFFFFFFB<</<FFBFFFFF//<FFFBFFF/7/<BFBFFF/777BFFFF

@GWZHISEQ02:315:C9E6MANXX:5:1104:12297:96529

CTGTGGTCGAGCACCTGGGCCCCGGCACAGGGCCAAGAACAGATGGTACTCAGATAAAGCGAAACTAGCAACAGTTTCTGGAAAGTCCCACCTCAGTTTCAAGTTCCCAGATCGGAAGAGCGTCGT

+

B<BBBFFFF<FBFB<B</FF///BBF/<B<B<7FFFFFFFFFFFFFFFBFBFFFFFFFFFFBBFFBFB/FFFFFB/B/F/BFBFFFFF/FF/BBBF/<FF/7FF<7//7//BBF/B<<FFFF7B77

@GWZHISEQ02:315:C9E6MANXX:5:1303:3281:77902

CTGTGGTCGAGCACCTGGGCCCCGGCTCAGGGCCAAGAACAGATGGTACTCAGATAAAGCGAAACTAGCAACAGTTTCTGGAAAGTCCCACCTCAGTTTCAAGTTCCCCAAAAGACCGGGAAAAAC

+

BBBBBFFFFFFFBFFFFFFFFFFFFFFFFFFFFFFFFFFFFFFFFFFFFFFFFFFFFFFFFFFFFFFFFFFFFFFFFFFFFFFFFFFFFFFFFFFFFFFFFFFFFFFFFFFFFFFFFFFFFFFFFF

@GWZHISEQ02:315:C9E6MANXX:5:2104:18858:41098

CTGTGGTCGAGCACCTGGGCCCCGGCTCAGGGCCAAGAACAGATGGTACTCAGATAAAGCGAAACTAGCAACAGTTTCTGGAAAGTCCCACCTCAGTTTCAAGTTCCCCAAAAGACCGGGAAAAAC

+

BBBBBFFFFBBBFFFFF</FFFFFFFFBFFFFFFFFFFFFFFFFFFFFFFFFFFFFFFFFFFFFFFFFFFFFFFFFFFFFFFFFFFFFFFFFFFFFFFFFFFFFFBFFFFFFFFFFFFFFFFFFFF

@GWZHISEQ02:315:C9E6MANXX:5:2109:2450:83344

CTGTGGTCGAGCACCTGGGCCCCGGCTCAGGGCCAAGAACAGATGGTACTCAGATAAAGCGAAACTAGCAACAGTTTCTGGAAAGTCCCACCTCAGTTTCAAGTTCCCCAAAAGACCGGGAAAAAC

+

B/BBBFFFF////B//<////////77<FFFFFFFFFFFFFFFFFFFFF/F/BFFFFFBFFFFFFFBF<FFFFF/FFFFFFFFFFFFFFFFFFFFBFBFFFFF/FFFFFFFFFFFFF/7BBBFFFF

@GWZHISEQ02:315:C9E6MANXX:5:2201:2172:98716

CTGTGGTCGAGCACCTGGGCCCCGGCTCAGGGCCAAGAACAGATGGTACTCAGATAAAGCGAAACTAGCAACAGTTTCTGGAAAGTCCCACCTCAGTTTCAAGTTCCCCAAAAGACCGGGAAAAAC

+

BBBBBFFFFFFBBFFFFF/FFFFBBFFFFFFFFFFFFFFFFFFFFFFFFFFFFFFFFFFFFFFFFFFFFFFFFFFFFFFFFFFFFFFFFFFFFFFFFFFFFFFFFFFFFF<FFFFFFFFFFFFFFF

@GWZHISEQ02:315:C9E6MANXX:5:2205:9966:52055

CTGTGGTCGAGCACCTGGGCCCCGGCTCAGGGCCAAGAACAGATGGTACTCAGATAAAGCGAAACTAGCAACAGTTTCTGGAAAGTCCCACCTCAGTTTCAAGTTCCCCAAAAGACCGGGAAAAAC

+

BBBBBFFFFFFB<FFFB//</FBFFBF<FFFFFFFFFFFFFFFFFFFFFFFFFFFFFFFFFFFFFFFFFFFFFFFFFFFFBFFFFFFFBFFFFFFFFFFFFFFFFFFFFFFFFFFFFFFFFFFFF<

@GWZHISEQ02:315:C9E6MANXX:5:2205:6488:58448

CTGTGGTCGAGCACCTGGGCCCCGGCTCAGGGCCAAGAACAGATGGTACTCAGATAAAGCGAAACTAGCAACAGTTTCTGGAAAGTCCCACCTCAGTTTCAAGTTCCCCAAAAGACCGGGAAAAAC

+

BBBBBFFFFBFF<BBB</BBFBF//BBBFFFFFFFFFFFFFFFFFFFFFFFFFFFFFFFFFFFFFFFFFFFFFF<BBFFFFFFFFFFFFFFFFFFFFFFFFFFFFFFFFFFFFFFFFFFFFF/FF<

@GWZHISEQ02:315:C9E6MANXX:5:2206:4990:53625

CTGTGGTCGAGCACCTGGGCCCCGGCTCAGGGCCAAGAACAGATGGTACTCAGATAAAGCGAAACTAGCAACAGTTTCTGGAAAGTCCCACCTCAGTTTCAAGTTCCCCAAAAGACCGGGGAGATC

+

BBBBBFFFFFFFFBFFFFFFFFFFBFFFFFFFFFFFFFFFFFFFFFFFFFFFFFFFFFFFFFFFFFFFFFFFFFFFFFFFFFFFFFFFFFFFFFFFFFFFFFFFFFFFFFF/FFFFFFFFBBFFFF

@GWZHISEQ02:315:C9E6MANXX:5:2208:18986:51368

CTGTGGTCGAGCACCTGGGCCCCGGCTCAGGGCCAAGAACAGATGGTACTCAGATAAAGCGAAACTAGCAACAGTTTCTGGAAAGTCCCACCTCAGTTTCAAGTTCCCCAAAAGACCGGGAAAAAC

+

BBBBBFFFF/<<B/<<//////7<//<FFFFFFFBFFFFFFFFFFFFFFFFBFFFFFFFFFFFFFFFF<FFFFF/<<FFFFBFFFBFBFFB<FBFF<FFFFFF<FFBFBF<FF/BFBB</BFF<BB

@GWZHISEQ02:315:C9E6MANXX:5:2303:2281:12173

CTGTGGTCGAGCACCTGGGCCCCGGCTCAGGGCCAAGAACAGATGGTACTCAGATAAAGCGAAACTAGCAACAGTTTCTGGAAAGTCCCACCTCAGTTTCAAGTTCCCCAAAAGACCGGGAAAAAC

+

BBBBBFFFFFF<FFFBFB/BFFF<7BBBFFFFFFFFFFFFFFFFFFFFFFFFFFFFFFFFFFFFFFFFFFFFFFFFFFFFFFFFFFFFFFFFFFFFFFFFFFFFFFFFFFFFFFFFFFFFFFFFFB

@GWZHISEQ02:315:C9E6MANXX:5:2312:2898:20365

CTGTGGTCGAGCACCTGGGCCCCGGCTCAGGGCCAAGAACAGATGGTACTCAGATAAAGCGAAACTAGCAACAGTTTCTGGAAAGTCCCACCTCAGTTTCAAGTTCCCCAAAAGACCGGGAAAAAC

+

B/BBBFFFFBB//<</////<7///777FFFFFFFFFFFFFFFFFFFFFFFFFFFFFFFFFFFFFFFFFFFFFFFFFFFFFFFFFFFFFFFFFFFFBBFFF<F/<BFFFFFF<FBFFFBFBFBBFF

@GWZHISEQ02:315:C9E6MANXX:5:1115:20963:86548

GTTTTTCCCGGTCTTTTGGGGAACTTGAAACTGAGGTGGGACTTTCCAGAAACTGTTGCTAGTTTCGCTTTATCTGAGTACCATCTGTTCTTGGCCCTGAGCCGGGGCCCAGGTGCTCGACCACAG

+

BB<BBFFFFFFFFFFFFFFFFFFFFFFFFFFFFFFFFFFFFFFFFFFFFFFFFFFFFFFFFFFFF/FFFFFFFFFFF<BFFFFFFFFFFFFFFFFFFFBFFFFFFFFFFFFFFFFFFFBFFFFFFF

@GWZHISEQ02:315:C9E6MANXX:5:2101:18471:55813

GTTTTTCCCGGTCTTTTGGGGAACTTGAAACTGAGGTGGGACTTTCCAGAAACTGTTGCTAGTTTCGCTTTATCTGAGTACCATCTGTTCTTGGCCCTGAGCCGGGGCCCAGGTGCTCGACCACAG

+

BBBBBFFFFFFFFFFFFFFFFFFFFFFFFFFFFFFFFFFFFFFFFFFFFFFFFFFFFFFFFFFFFFFFFFFFFFFFFFFFFFFFFFFFFFFFFFFFFFFFFFFFFFFFFFFFFFFFFFFFFFFF</

@GWZHISEQ02:315:C9E6MANXX:5:2105:13693:63125

GTTTTTCCCGGTCTTTTGGGGAACTTGAAACTGAGGTGGGACTTTCCAGAAACTGTTGCTAGTTTCGCTTTATCTGAGTACCATCTGTTCTTGGCCCTGAGCCGGGGCCCAGGTGCTCGACCACAG

+

BBBBBFFFFFFFFFFFFFBFFFFFFFFFFFFFFFFFFFFFFFFFFFFFFFFFFFFFFFFFFFFFFFFFFFFBFFFFFFFFFFFFFFFFFFFFFFFFFFFFFFFFFFFFFFFFFFFFFFFFFFFFFF

@GWZHISEQ02:315:C9E6MANXX:5:1205:15307:7059

GGTTTTTCCCGGTCTTTTGGGGAACTTGAAACTGAGGTGGGACTTTCCAGAAACTGTTGCTAGTTTCGCTTTATCTGAGTACCATCTGTTCTTGGCCCTGAGCCGGGGCCCAGGTGCTCGACCACA

+

BBBBBFFFFFFFFFFFFFFFFFFFFFFFFFFFFFFFFFFFFFFFFFFFFFFFFFFFFFFFFFFFFFFFFFFFFFFFFFFFFFFFFFFFFFFFFFFFFFFFFFFFFFFFFFFBFFFFB<FFFFFFF<

@GWZHISEQ02:315:C9E6MANXX:5:1302:14151:54421

GTGGTCGAGCACCTGGGCCCCGGCTCAGGGCCAAGAACAGATGGTACTCAGATAAAGCGAAACTAGCAACAGTTTCTGGAAAGTCCCACCTCAGTTTCAAGTTACCCAAAAGAGATCGGAAGAGCG

+

BBB<//<FFBFF</<//<<F///<B<FB<B<FFF/<BB<B<BBFBBFBBFFF<FFFFFF/<</<F</BFFFF<F/<BFF</BF7/F/</7BF<77/77F7/FB/7//B7FFFB7FB/7BFFFBBFB

@GWZHISEQ02:315:C9E6MANXX:5:1112:18442:24735

TGGTCGAGCACCTGGGCCCCGGCTCAGGGCCAAGAACAGATGGTACTCAGATAAAGCGAAACTAGCAACAGTTTCTGGAAAGTCCCACCTCAGTTTCAAGTTCCCCAAAAGACCGGGAAAAACCCC

+

BBBBBFFFFFFFFFFFFFFFFFFFFFFFFFFFFFFFFFFBFFFFFFFFFFFFFFFFFFFFFFFFFFFFFFFFFFFFFFFFFFFFFFFFFFFFFFFFFFFFFFFFFFFFFBFFFFFFFFFFFFFFFF

@GWZHISEQ02:315:C9E6MANXX:5:2201:7057:4700

TGGTCGAGCACCTGGGCCCCGGCTCAGGGCCAAGAACAGATGGTACTCAGATAAAGCGAAACTAGCAACAGTTTCTGGAAAGTCCCACCTCAGTTTCAAGTTCCCCAAAAGACCGGGAAAAACCCC

+

BBBBBFFFFFFFFFFFFFFFFFFFFFFFFFFFFFFFFFFFFFFFFFFFFFFFFFFFFFFFFFFFFFFFFFFFFFFFFFFFFFFFFFFFFFFFFFFFFFFFFFFFFFFFFFFFFFFFFFFFFFFFFF

@GWZHISEQ02:315:C9E6MANXX:5:2209:2555:42375

TGGTCGAGCACCTGGGCCCCGGCTCAGGGCCAAGAACAGATGGTACTCAGATAAAGCGAAACTAGCAACAGTTTCTGGAAAGTCCCACCTCAGTTTCAAGTTCCCCAAAAGACCGGGAAAAACCCC

+

/<BBBF/B<FFF<B/FFFFFFFFF<FFFFFFFB/FFFFFFFF/BBF///<<FFFFBFFFBFFFFFF/B/FFB<BBFFBFFFFFFFFBB<FFFBFFFFFFFFFBFFFFBB////</77BF/BB<<77

@GWZHISEQ02:315:C9E6MANXX:5:1104:5422:23091

GGGGTTTTTCCCGGTCTTTTGGGGAACTTGAAACTGAGGTGGGACTTTCCAGAAACTGTTGCTAGTTTCGCTTTATCTGAGTACCATCTGTTCTTGGCCCTGAGCCGGGGCCCAGGTGCTCGACCA

+

BBBBBFFFFFFFFFFFFFFFFFFFBFFFFFFFFFFFFFFFFFFFFFFFFFFFFFFFFFFFFFFFFFFFFFFFFFFFFFFFFFFFFFFFFFFFFFFFFFFFFFFFFFFFFFFFFFFFFFFFFFFFB/

@GWZHISEQ02:315:C9E6MANXX:5:1112:18442:24735

GGGGTTTTTCCCGGTCTTTTGGGGAACTTGAAACTGAGGTGGGACTTTCCAGAAACTGTTGCTAGTTTCGCTTTATCTGAGTACCATCTGTTCTTGGCCCTGAGCCGGGGCCCAGGTGCTCGACCA

+

<BBBBBFFFFFFFFFFB<FFFFFFBFFFFFFFFFFFFFFFFFFBFFFFFFFFFFFFFFFFFFFFFFFFFFFFFFFFFFFFFFFFFFFFFFFFFFFFFFFFFFBBFFFFFFFFFBFFFFBFFFFFFF

@GWZHISEQ02:315:C9E6MANXX:5:1205:9081:85286

GGGGTTTTTCCCGGTCTTTTGGGGAACTTGAAACTGAGGTGGGACTTTCCAGAAACTGTTGCTAGTTTCGCTTTATCTGAGTACCATCTGTTCTTGGCCCTGAGCCGGGGCCCAGGTGCTCGACCA

+

BBBBBFFFFFFFFFBFFFFFFFFFFFFFFFFFFFFFFFFFFFFFFFFFFFFFFFFFFFFFFFFFFFFFFFFFFFFFFFFFFFFFFFFFFFFFFFFFFFFFFFFFFFFFFFFFFBFFFFFBFFFFBB

@GWZHISEQ02:315:C9E6MANXX:5:1213:20424:69011

GGGGTTTTTCCCGGTCTTTTGGGGAACTTGAAACTGAGGTGGGACTTTCCAGAAACTGTTGCTAGTTTCGCTTTATCTGAGTACCATCTGTTCTTGGCCCTGAGCCGGGGCCCAGGTGCTCGACCA

+

BBBBBFFFFFFFFFFFFFFFFFFFFFFFFFFFFFFF<FFFFFBFFFFFFBFFFFFFFFFFFFFFFFFFFFFFFFFFFBBFFFFFFBFBFFFFFFFB<BFFFFFFFF7BFFFFFFFFFFFFFBFFF<

@GWZHISEQ02:315:C9E6MANXX:5:1304:12728:45211

GGGGTTTTTCCCGGTCTTTTGGGGAACTTGAAACTGAGGTGGGACTTTCCAGAAACTGTTGCTAGTTTCGCTTTATCTGAGTACCATCTGTTCTTGGCCCTGAGCCGGGGCCCAGGTGCTCGACCA

+

BBBBBFFFFFFFFFFFFFFFFBFFFFFFFFFFFFFFFFFFFFFFFFFFFFFFFFFFFFFFFFFFFFFFFFFFFFFFFFFFFFFFFFFFFFFFFFFBFFFFFFFFFFFFFFFFFFFFFFFFFFFFF/

@GWZHISEQ02:315:C9E6MANXX:5:2105:7210:48016

GGGGTTTTTCCCGGTCTTTTGGGGAACTTGAAACTGAGGTGGGACTTTCCAGAAACTGTTGCTAGTTTCGCTTTATCTGAGTACCATCTGTTCTTGGCCCTGAGCCGGGGCCCAGGTGCTCGACCA

+

B/BBBFFFFFFFFFFBFFFFFFFFFFFFFBFFFFBFF/</FFB<FBFFFFFFF/FFFBFF<BBFFFFFFFF/FFFFFFFFBFFFFFFFFFFFFFFFBFFFFBBF7FFFFBFFF<FFF<B/B<<FFB

@GWZHISEQ02:315:C9E6MANXX:5:2113:5379:63999

GGGGTTTTTCCCGGTCTTTTGGGGAACTTGAAACTGAGGTGGGACTTTCCAGAAACTGTTGCTAGTTTCGCTTTATCTGAGTACCATCTGTTCTTGGCCCTGAGCCGGGGCCCAGGTGCTCGACCA

+

BBBBBFFFFFFFFFFFFFFFFFFBFFFFFFFFFFFFFFFFFFFBFFFFFFFFFFFFBBFFFFFFFFFFFFFFFFFFFFFFFFFFFFFFBFFFFFFFFFFFFFFFFFB<FFFFFFFFFFFFFFFFFB

@GWZHISEQ02:315:C9E6MANXX:5:2115:21056:33648

GGGGTTTTTCCCGGTCTTTTGGGGAACTTGAAACTGAGGTGGGACTTTCCAGAAACTGTTGCTAGTTTCGCTTTATTTGAGTACCATCTGTTCTTGGCCCTGAGCCGGGGCCCAGGTGCTCGACCA

+

BBBBBFFFFFFFFFFFFFFFFFFFFB<FFFFFFFFFFFFFFBFFFFFFF<//FF/<<<F<<///<FFF/7/FFFF///F/<</FF/////FF/<<BBFF<//7/F/<FF//BB/<FF///7//77/

@GWZHISEQ02:315:C9E6MANXX:5:2201:7057:4700

GGGGTTTTTCCCGGTCTTTTGGGGAACTTGAAACTGAGGTGGGACTTTCCAGAAACTGTTGCTAGTTTCGCTTTATCTGAGTACCATCTGTTCTTGGCCCTGAGCCGGGGCCCAGGTGCTCGACCA

+

BBBBBFFFFFFFFFFFFFFFFFFFFFFFFFFFFFFFFFFFFFF<FFFFFFFFFFFFFFFFFFFFFFFFFFF<BF/FFFFFFFFFFFFFFBFFFFFBFFFFFBFFFFBFFFFFFBBBFFFFFFBFFF

@GWZHISEQ02:315:C9E6MANXX:5:2204:5635:19937

GGGGTTTTTCCCGGTCTTTTGGGGAACTTGAAACTGAGGTGGGACTTTCCAGAAACTGTTGCTAGTTTCGCTTTATCTGAGTACCATCTGTTCTTGGCCCTGAGCCGGGGCCCAGGTGCTCGACCA

+

<BBBBFFF</</<B<BFFFF<<BF/<<FFFFFBFFFFFFB//B/FFFF/FBFFFFFFBFF<B/B/FFB/F</FFFFBFFF/BFFF<FFF/BFBFBF/F/FF//<//7BF</77BFFBBBF<7/BBB

@GWZHISEQ02:315:C9E6MANXX:5:2314:13705:63221

GGGGTTTTTCCCGGTCTTTTGGGGAACTTGAAACTGAGGTGGGACTTTCCAGAAACTGTTGCTAGTTTCGCTTTATCTGAGTACCATCTGTTCTTGGCCCTGAGCCGGGGCCCAGGTGCTCGACCA

+

BBBBBFFFFFFFFFFFFFFFFFFF<FBFFFFFF<FFFFFFFFFFFFFFFFFFFFFFFFFFFFFFFFFFFFFFFFFFFFFFFFFFFFFFFFFFFFFFFFFFFFFFFFFFFFFFFFFFFFFFFFFFF/

@GWZHISEQ02:315:C9E6MANXX:5:1205:16446:72163

CGGGGTTTTTCCCGGTCTTTTGGGGAACTTGAAACTGAGGTGGGACTTTCCAGAAACTGTTGCTAGTTTCGCTTTATCTGAGTACCATCTGTTCTTGGCCCTGAGCCGGGGCCCAGGTGCTCGACC

+

BBBBBFFFFFFFFFFFFFFFFFFFFFFFFFFFFFFFFFFFFFFFFFFFFFFFFFFFFFFFFFFFFFFFFFFFFFFFFFFFFFFFFFFFFFFFFFFFFFFFFFBFFFFFFFFFFFFFFFFFFFFFFF

@GWZHISEQ02:315:C9E6MANXX:5:1303:3281:77902

GGGGGTTTTTCCCGGTCTTTTGGGGAACTTGAAACTGAGGTGGGACTTTCCAGAAACTGTTGCTAGTTTCGCTTTATCTGAGTACCATCTGTTCTTGGCCCTGAGCCGGGGCCCAGGTGCTCGACC

+

BBBBBFFFFFFFFFFFFFFFFFFFFFFFFFFFFFFFFFFFFFFFFFFFFFFFFFFFFFFFFFFFFFFFFFFFFFFFFFFFFFFFFFFFFFFFFFFFBFFFFFFFFFFFFFFFFFFFFFFFFFFFFF

@GWZHISEQ02:315:C9E6MANXX:5:2112:5326:71613

TGGGGTTTTTCCCGGTCTTTTGGGGAACTTGAAACTGAGGTGGGACTTTCCAGAAACTGTTGCTAGTTTCGCTTTATCTGAGTACCATCTGTTCTTGGCCCTGAGCCGGGGCCCAGGTGCTCGACC

+

BBBBBFFFFFFFFFFFFFFFFFFFFFFFFFFFFFFFFFFFFFFFFFFFFFFFFFFFFFFFFFFFFFFFFFFFFFFFFFFFFFFFFFFFFFFFFFFFFFFFFFFBFFFFFFFFFFFFFFFFFBFFFF

@GWZHISEQ02:315:C9E6MANXX:5:2202:1734:68759

CGGGGTTTTTCCCGGTCTTTTGGGGAACTTGAAACTGAGGTGGGACTTTCCAGAAACTGTTGCTAGTTTCGCTTTATCTGAGTACCATCTGTTCTTGGCCCTGAGCCGGGGCCCAGGTGCTCGACC

+

BBBBBFFFFFFFFFFFFFFFFFFFFFFFFFFFFFFFFFFFFFFFFFFFFFFFFFFFFFFFFFFFFFFFFFFFFFFFFFFFFFFFFFFFFFFFFFFFFFFFFFFFFFFFFFFFFFBBF7FF<F//BF

@GWZHISEQ02:315:C9E6MANXX:5:2215:5750:70646

TGGGGTTTTTCCCGGTCTTTTGGGGAACTTGAAACTGAGGTGGGACTTTCCAGAAACTGTTGCTAGTTTCGCTTTATCTCAGTACCATCTGTTCTTGGCCCTGAGCCGGGGCCCAGGTGCTCGACC

+

BBBBBFFFFFFFFFFFFFFFFFFFFFFFFFFFFFFFFFFFFFFFFFFFFFFFFFFFFFFFFFFFFFFFFFFFFFFFFFF/<FFFFFFFFFFFFFFFFFFFFFFFFFFFFFFFFFFFFFFFFFFFFF

@GWZHISEQ02:315:C9E6MANXX:5:1108:17597:97742

TTGGGGTTTTTCCCGGTCTTTTGGGGAACTTGAAACTGAGGTGGGACTTTCCAGAAACTGTTGCTAGTTTCGCTTTATCTGAGTACCATCTGTTCTTGGCCCTGAGCCGGGGCCCAGGTGCTCGAC

+

BBBBBBFFFFFFFFFFFFFFFFFFFFFFFFFFFFFFFFFFFFFFFFFFFFFFFFFFFFFFFFFFFFFFFFFFFFFFFFFFFFFFFFFFFFFFFBBFFFFFFFFFFFFFFFFFFFFFFFFFFFFFFF

@GWZHISEQ02:315:C9E6MANXX:5:1213:4996:3552

TTGGGGTTTTTCCCGGTCTTTTGGGGAACTTGAAACTGAGGTGGGACTTTCCAGAAACTGTTGCTAGTTTCGCTTTATCTGAGTACCATCTGTTCTTGGCCCTGAGCCGGGGCCCAGGTGCTCGAC

+

BBBBBFFFFFFFFFFFFFFFFFFFFFFFFFFFFFFBFFFBFFFFFFFFFFFFFFFFFFFFFFFFFFFFFFFFFFFFFFFFBFBBFFFFFFFFBFFFFFFFFFFFFFFFFFFFBBFFFFFBFFFFFF

@GWZHISEQ02:315:C9E6MANXX:5:2110:4155:78371

TTGGGGTTTTTCCCGGTCTTTTGGGGAACTTGAAACTGAGGTGGGACTTTCCAGAAACTGTTGCTAGTTTCGCTTTATCTGAGTACCATCTGTTCTTGGCCCTGAGCCGGGGCCCAGGTGCTCGAC

+

BBBBBFFFFFFFBFFFFFFFFFFFFBBBFFFFFFFFFF<BFFFFFFFFFFFFFFFFFFFFFFFFFBFBFFFFFFFFFFFFFFFFFFBFFFFFFFFFF<BFFFFBBFFFBBFFFFFFFFFF<FF<FF

@GWZHISEQ02:315:C9E6MANXX:5:2111:7294:17953

GCTAGTTTCGCTTTATCTGAGTACCATCTGTTCTTGGCGCTGAGCCGAGCCGCCGGTGCTTGACGAAACAGACCTTGGCATCAGTGGGCCGGGGGTTGGTTGTGGATGGTACCTTCACTATACATC

+

BBBBBFFFFFFFFFFFFFFFFFFFFFFFFFFFBBFF/</<BBF//<//</////////<///<//////<//////</////</7/////7/7///<//77//<///////////////</////7

@GWZHISEQ02:315:C9E6MANXX:5:2307:14468:45567

TTGGGGTTTTTCCCGGTCTTTTGGGGAACTTGAAACTGAGGTGGGACTTTCCAGAAACTGTTGCTAGTTTCGCTTTATCTGAGTACCATCTGTTCTTGGCCCTGAGCCGGGGCCCAGGTGCTCGAC

+

BBBBBFFFFFFFFFFFFFFFFFFFFFFFFFFFFFFFFFFFFFFFFFFFFFFFFFFFFFFFFFFFFFFFFFFFFFFFFFFFFBFFFFFFFBFFFFFFFBBFFFFFFFFFBFFFFFFFFFFFFFFFFF

@GWZHISEQ02:315:C9E6MANXX:5:1112:16460:5750

CTTGGGGTTTTTCCCGGTCTTTTGGGGAACTTGAAACTGAGGTGGGACTTTCCAGAAACTGTTGCTAGTTTCGCTTTATCTGAGTACCATCTGTTCTTGGCCCTGAGCCGGGGCCCAGGTGCTCGA

+

BBBBBFFFFFFFFFFFFFFFFFFFFFFFFFFFFFFFFFFFFFFFFFFFFFFFFFFFFFFFFFFFFFFFFFFFFFFFFFFFFFFFFFFFFFFFFFFFFFFFFFFFBFFFFFFFFFFFFFFFFFFFFF

@GWZHISEQ02:315:C9E6MANXX:5:2105:7771:13053

CTCGGGGTTTTTCCCGGTCTTTTGGGGAACTTGAAACTGAGGTGGGACTTTCCAGAAACTGTTGCTAGTTTCGCTTTATCTGAGTACCATCTGTTCTTGGCCCTGAGCCGGGGCCCAGGTGCTCGA

+

BBBBBFFFFFFFFFFFFFFFFFFFFFFFFFFFFFFFFFFFFFFFFFFFFFFFFFFFFFFFBFFFFFFFFFFFFFF/FFFFFFFFFFFFFFFFFFFFFFFFFFFFFFFFFFFFFFFFFFFFFFFFFF

@GWZHISEQ02:315:C9E6MANXX:5:2107:15856:18514

CTTGGGGTTTTTCCCGGTCTTTTGGGGAACTTGAAACTGAGGTGGGACTTTCCAGAAACTGTTGCTAGTTTCGCTTTATCTGAGTACCATCTGTTCTTGGCCCTGAGCCGGGGCCCAGGTGCTCGA

+

BBBBBFFFFFFFFFFFFFFFFFFFFBFFFFFFFFFFFFFBFFFFFFFFFFFFFFFFFFFFFFFFFBFFFFFFFFFFFFFFFFFFFFFFFFFFFFFFFFFFFFFFFFFFFFFFFFFF/BBFFFFFFF

@GWZHISEQ02:315:C9E6MANXX:5:2108:7432:95949

CTTGGGGTTTTTCCCGGTCTTTTGGGGAACTTGAAACTGAGGTGGGACTTTCCAGAAACTGTTGCTAGTTTCGCTTTATCTGAGTACCATCTGTTCTTGGCCCTGAGCCGGGGCCCAGGTGCTCGA

+

BBBBBFFFFFFFFFFFFFFFFFFFFBFFFFFFFFFFFFFFFFFFFF<FFFFFFFFFFFFFFFFFFFFFFBFFFFFFFFFFFFFFFFFFFFFFFFFFFFFFBFFFFBFFFFFFFFFF<FFFFFBBBF

@GWZHISEQ02:315:C9E6MANXX:5:2112:1925:87577

CTTGGGGTTTTTCCCGGTCTTTTGGGGAACTTGAAACTGAGGTGGGACTTTCCAGAAACTGTTGCTAGTTTCGCTTTATCTGAGTACCATCTGTTCTTGGCCCTGAGCCGGGGCCCAGGTGCTCGA

+

<<BBBBF<FFBBBBFFF<BBBFF/F<B<</B/</BFB<B/B/F/</<//BFB<<F/<<<B/FF</B/BFFFF/FFFFFFFF/B/FFBFFFFF</</FF</</BFFFF//BBBB<BF///77/7/B<

@GWZHISEQ02:315:C9E6MANXX:5:2116:3813:98025

CTTGGGGTTTTTCCCGGTCTTTTGGGGAACTTGAAACTGAGGTGGGACTTTCCAGAAACTGTTGCTAGTTTCGCTTTATCTGAGTACCATCTGTTCTTGGCCCTGAGCCGGGGCCCAGGTGCTCGA

+

BB<BBFFFFFFFFFFBFFFFFFFFFFFF<FFF<FFFFFFFFFFFFFFFFFFFFFFFFFFFFFFFFFFBFFFFFFFFFFFFFFFBFFFFFFFFFFFFFFFFFFFFFFFFFFFBBBFFFFFFFFFFBF

@GWZHISEQ02:315:C9E6MANXX:5:2312:2898:20365

CTTGGGGTTTTTCCCGGTCTTTTGGGGAACTTGAAACTGAGGTGGGACTTTCCAGAAACTGTTGCTAGTTTCGCTTTATCTGAGTACCATCTGTTCTTGGCCCTGAGCCGGGGCCCAGGTGCTCGA

+

BBBBBF<FFFFFFFFFFFFFFFFFFFFFFFFFFFFFFFFFFFFFFFF<FFFFFFBFFFFFFFFFFFFFFBFFFFFFFFFFFFFFFFFFFFFBFFFFFFFFFFFFFFFFFFFFBFFBBFFFFFFFF/

@GWZHISEQ02:315:C9E6MANXX:5:2206:6170:66647

CGAGCACCTGGGCCCCGGCTCAGGGCCAAGAACAGATGGTACTCAGATAAAGCGAAACTAGCAACAGTTTCTGGAAAGTCCCACCTCAGTTTCAAGTTCCCCAAAAGACCGGGAAAAACCCCAAGC

+

BBBBBFFFFFFFFFFFBFFFFFFFFFFFFFFFFFFFFFFFFFFFFFFFFFFFFFFFFFFFFFFFFFFFFFFFFFFFFFFFFFFFFFFFFFFFFFFFFFFFFFFFFFFFFFFFFFFBFFFFFFBFFF

@GWZHISEQ02:315:C9E6MANXX:5:1114:15769:13282

GCTTGGGGTTTTTCCCGGTCTTTTGGGGAACTTGAAACTGAGGTGGGACTTTCCAGAAACTGTTGCTAGTTTCGCTTTATCTGAGTACCATCTGTTCTTGGCCCTGAGCCGGGGCCCAGGTGCTCG

+

BBBBBBFF<FFFFFFFFBFFFFFFFFFFBFFFFFFF<FFFFFFFBFFFFFFFFFFFFFFFFFFFFFFFF<FFFFFFFFFFFFFFFFFFFFFFFFFFFFFFFFFFFFFBBFFF<FFBFFFFFBFFFF

@GWZHISEQ02:315:C9E6MANXX:5:1204:15600:7609

GCTTGGGGTTTTTCCCGGTCTTTTGGGGAACTTGAAACTGAGGTGGGACTTTCCAGAAACTGTTGCTAGTTTCGCTTTATCTGAGTACCATCTGTTCTTGGCCCTGAGCCGGGGCCCAGGTGCTCG

+

BBBBBFFFFFFFFFFFFFFFFFFFFFFFFFFFFFFFFFFFFFFFFFFFFFFFFFFFFFFFFFFFBFFFFFFFFFF<FFBFFFFFFFFFFFFFFFFFFFFFFFFFFFFFFFFBFFFFFFFFFFFFFF

@GWZHISEQ02:315:C9E6MANXX:5:1212:18958:97124

CTTGGGGTTTTTCCCGGTCTTTTGGGGAACTTGAAACTGAGGTGGGACTTTCCAGAAACTGTTGCTAGTTTCGCTTTATCTGAGTACCATCTGTTCTTGGCCCTGAGCCGGGGCCCAGGTGCTCGC

+

/BBBBFFBFFFFFFFFB<FFFFFFBFF7FFFFFFFFFFFBFFFFB/FFFFFFFFF//BFFFFF<FFFFFFFFFF<FFFFFFBBFFFFFBFFFFFBFFF/BFF<BBFF<BFFFFFFB<FFBFFBFF/

@GWZHISEQ02:315:C9E6MANXX:5:1303:9442:51307

GCTTGGGGTTTTTCCCGGTCTTTTGGGGAACTTGAAACTGAGGTGGGACTTTCCAGAAACTGTTGCTAGTTTCGCTTTATCTGAGTACCATCTGTTCTTGGCCCTGAGCCGGGGCCCAGGTGCTCG

+

/</<<FBF<BFFFFF<FFFFFFFF<BFFBFBFFFFFF/FF/<FFFFF/BBFFB<BFBFFFFFFFFBFBFFFF/FB<FFBFBF//F/<<BBFFFFFFFFFB/FFFFFFFFFFFFBBFF<<FFBFFF/

@GWZHISEQ02:315:C9E6MANXX:5:1306:3869:42987

CCTTGGGGTTTTTCCCGGTCTTTTGGGGAACTTGAAACTGAGGTGGGACTTTCCAGAAACTGTTGCTAGTTTCGCTTTATCTGAGTACCATCTGTTCTTGGCCCTGAGCCGGGGCCCAGGTGCTCG

+

BBBBBFFFFFFFFFFFFFFFFFFFFFFFFFFFFFFFFFFFFFFFFFFFFFFFFFFFFFFFFFFFFFFFFFFFFFFFFFFFFFB<FFFFFFFFFFFFFFFFFFFFFFFFFFFFFFFFFFFFFFFFFF

@GWZHISEQ02:315:C9E6MANXX:5:2103:16764:63463

GCTTGGGGTTTTTCCCGGTCTTTTGGGGAACTTGAAACTGAGGTGGGACTTTCCAGAAACTGTTGCTAGTTTCGCTTTATCTGAGTACCATCTGTTCTTGGCCCTGAGCCGGGGCCCAGGTGCTCG

+

BBBBBFFFFFFFFFFFFFFFFFFFFBFFFFFFFFFFFFFFFFFFBFFFFFFFFFFFFFFFFFFFFFFFFFFFFFFFFFBFFFFFFBFFFFFFFFFFFFFFFFFFFFFFFFFFFFBFFBBFFFFFFB

@GWZHISEQ02:315:C9E6MANXX:5:2106:11632:35773

GCTTGGGGTTTTTCCCGGTCTTTTGGGGAACTTGAAACTGAGGTGGGACTTTCCAGAAACTGTTGCTAGTTTCGCTTTATCTGAGTACCATCTGTTCTTGGCCCTGAGCCGGGGCCCAGGTGCTCG

+

BBBBBFFFFFFFFFFFFFFFFFFFFFFFFFFFFFFFFFFFFFFFFFFFFFFFFFFFFFFFFFFFFFFFFFFFFFFFFFFFFFFFFBFFFFFFFFFFFFFBFFFFFFBFFFFFFFFFFFFFFFFFFF

@GWZHISEQ02:315:C9E6MANXX:5:2308:5092:54063

GCTTGGGGTTTTTCCCGGTCTTTTGGGGAACTTGAAACTGAGGTGGGACTTTCCAGAAACTGTTGCTAGTTTCGCTTTATCTGAGTACCATCTGTTCTTGGCCCTGAGCCGGGGCCCAGGTGCTCG

+

BBBBBBFFFFFFFFFFFFFFFFFFFFFFBFFFFBFFFFFFFFFFFFFFFFFFFFFFFFFFFFFFFFFFFFFFBFFFFFFFFFFFFFFFFFFFFFFFFFFFFFFFFFFFFFFFBFFFFFFF7BBFFB

@GWZHISEQ02:315:C9E6MANXX:5:2312:18027:7379

GCTTGGGGTTTTTCCCGGTCTTTTGGGGAACTTGAAACTGAGGTGGGACTTTCCAGAAACTGTTGCTAGTTTCGCTTTATCTGAGTACCATCTGTTCTTGGCCCTGAGCCGGGGCCCAGGTGCTCG

+

BBBBBFFFFFFFFFFFFFFFFFFFFFFB<FFFFF<FFFFFFFFFFFF/FFFFFFFFFFFFFFFFFFFFFFFFFFFFFFFFFFFFFFFFFFFFFFFFFFFFFFFFFFFFFFFFFBFFFFFFFFFFF<

@GWZHISEQ02:315:C9E6MANXX:5:1115:9517:44989

CGCTTGGGGTTTTTCCCGGTCTTTTGGGGAACTTGAAACTGAGGTGGGACTTTCCAGAAACTGTTGCTAGTTTCGCTTTATCTGAGTACCATCTGTTCTTGGCCCTGAGCCGGGGCCCAGGTGCTC

+

BBBBBF<FB<FFFFFFFFFFFFFFFFFFFFFFFFFFFFFFFFFFFFFFFFFFFFFFFFFFFFFFFFFFFFFFFFFFFFFFFFFFFFFFFFFFFFFFFFFFBFFFFFFFFFFFFFFFFFFFFFFFFF

@GWZHISEQ02:315:C9E6MANXX:5:1202:12709:27306

GGCTTGGGGTTTTTCCCGGTCTTTTGGGGAACTTGAAACTGAGGTGGGACTTTCCAGAAACTGTTGCTAGTTTCGCTTTATCTGAGTACCATCTGTTCTTGGCCCTGAGCCGGGGCCCAGGTGCTC

+

BBBBBFFFFFFFFFFFFFFBFFFFFFFFB/FFFFFFFBFFFBFFFFFFFFFFFBFBF<FFFFBFFFFFBFFFFFFFFFF<FBFF7FFFFFFFFFFFFBFFFFFFFFF<BBFFFFFBFFFFFFFBFB

@GWZHISEQ02:315:C9E6MANXX:5:2203:10931:5061

CGCTTGGGGTTTTTCCCGGTCTTTTGGGGAACTTGAAACTGAGGTGGGACTTTCCAGAAACTGTTGCTAGTTTCGCTTTATCTGAGTACCATCTGTTCTTGGCCCTGAGCCGGGGCCCAGGTGCTC

+

//<BBFF<F<B/FFFFFFF/BFFFF<7FF7<FBFFFFF<F////<FBF//FB<BFFFF/FF/FB<B/</<FBF//<FBF///</BFFFFF///FFFFFF<F/7B//<<B//7/77//<F/FF7BFB

@GWZHISEQ02:315:C9E6MANXX:5:1201:12389:67333

AGCACCTGGGCCCCGGCTCAGGGCCAAGAACAGATGGTACTCAGATAAAGCGAAACTAGCAACAGTTTCTGGAAAGTCCCACCTCAGTTTCAAGTTCCCCAAAAGACCGGGAAAAACCCCAAGCCT

+

BBBBBFFFFFFFFFFFFFFFFFFFFFFFFFFFFFFFFFFFFFFFFFFFFFFFFFFFFFFFFFFFFFFFFFFFFFFFFFFFFFFFFFFFFFFFFFFFFFFFFFFFFFFFFFFBFFFFFFFFFFFFFF

@GWZHISEQ02:315:C9E6MANXX:5:1210:10258:35446

AGCACCTGGGCCCCGGCTCAGGGCCAAGAACAGATGGTACTCAGATAAAGCGAAACTAGCAACAGTTTCTGGAAAGTCCCACCTCAGTTTCAAGTTCCCCAAAAGACCGGGAAAAACCCCAAGCCT

+

BBBBBFFFFFFFFFFFFFFFFFFFFFFFFFFFFFFFFFFFFFFFFFFFFFFFFFFFFFFFFFFFFFFFFFFFFFFFFFFFFFFFFFFFFFFFFFFFFFFFFFFFFFFFFFFFFFFFFFFF<FFFFF

@GWZHISEQ02:315:C9E6MANXX:5:1212:19496:63595

AGCACCTGGGCCCCGGCTCAGGGCCAAGAACAGATGGTACTCAGATAAAGCGAAACTAGCAACAGTTTCTGGAAAGTCCCACCTCAGTTTCAAGTTCCCCAAAAGACCGGGAAAAACCCCAAGCCT

+

BBBBBFFFFFFFFFFFFFFFFFFFFFFFFFFFFFFFFFFFFFFFFFFFFFFFFFFFFFFFFFFBFBFFFFFFFFFFFFFFFFFFFFFFBFFF<F<FFFFFF<FFFFFFFFBBFFFFFFFFFFFFFF

@GWZHISEQ02:315:C9E6MANXX:5:2201:6598:5547

AGCACCTGGGCCCCGGCTCAGGGCCAAGAACAGATGGTACTCAGATAAAGCGAAACTAGCAACAGTTTCTGGAAAGTCCCACCTCAGTTTCAAGTTCCCCAAAAGACCGGGAAAAACCCCAAGCCT

+

BBBBBFFFF/FFFFFFFFFBBFFFFFFFFFFFFFFFFFFFFFFFFFFFFFFFFFBFFFFFFFFFFFFFFFFFFFFFFFFFFFFFFFFFFFFFFFFFFFFFFFFFFFFFBFFFFFFFFFFFFFFFFF

@GWZHISEQ02:315:C9E6MANXX:5:1207:2163:59564

CACCTGGGCCCCGGCTCAGGGCCAAGAACAGATGGTACTCAGATAAAGCGAAACTAGCAACAGTTTCTGGAAAGTCCCACCTCAGTTTCAAGTTCCCCAAAAGACCGGGAAAAACCCCAAGCCTTA

+

<BBBBFFFFFFFFFFFFFFFFFFFBFFFFFFFFFFFFFFFFFFFFFFFBFFFFFFBFFFFFFFFFFFFFFBFFFFFFFFFFFFFFFFFFFFFF<BFFFF<F/BFFFFFFF/BFFFFFFFFFFFFFF

@GWZHISEQ02:315:C9E6MANXX:5:2214:15683:33822

CACCTGGGCCCCGGCTCAGGGCCAAGAACAGATGGTACTCAGATAAAGCGAAACTAGCAACAGTTTCTGGAAAGTCCCACCTCAGTTTCAAGTTCCCCAAAAGACCGGGAAAAACCCCAAGCCTTA

+

BBBBBFBBBBBFF/BFFFFF<F/<BFFFFFBFFFFFFFFF<FFFFFFFFFFFFFFFFFBFFFFBBFFFFFFFFFFFFFFFFFFFBB//FFBFFFFFFFFFFFFFFFF<FFFFF77BFFFFFFFF<B

@GWZHISEQ02:315:C9E6MANXX:5:2216:16357:58938

CACCTGTGCCCCGGCTCAGGGCCATGAACAGATGGTACTCATATAAAGCGAAACTAGCAACAGTTTCTGGAAAGTCCCACCTCAGTTTCAAGTTCCCCAAAAGACCGGGAAAAACCCCAAGCCTTA

+

B/<B<///B/<F//<FB////77////<<<F/FF/<///BF/<///<///77/<FFBF/B<<</<<<F/<////F/FFFB7F<BB//<///<///<F//7FFBFFF//7BB//7/B</77/77B7B

@GWZHISEQ02:315:C9E6MANXX:5:1204:12846:30380

CCTGGGCCCCGGCTCAGGGCCAAGAACAGATGGTACTCAGATAAAGCGAAACTAGCAACAGTTTCTGGAAAGTCCCACCTCAGTTTCAAGTTCCCCAAAAGACCGGGAAAAACCCCAAGCCTTATT

+

BBBBBFFFFFFFFFFFFFFFFFFFFFFFFFFFFFFFFFFFFFFFFFFFFFFFFFFFFFFFFFFFFFFFFFFFFFFFFFFFFFFFFFFFFFFFFFFFFFFFFFFFFFFFFFFFFFFFFFFFFFFFFF

@GWZHISEQ02:315:C9E6MANXX:5:1307:2619:53359

CCTGGGCCCCGGCTCAGGGCCAAGAACAGATGGTACTCAGATAAAGCGAAACTAGCAACAGTTTCTGGAAAGTCCCACCTCAGTTTCAAGTTCCCCAAAAGACCGGGAAAAACCCCAAGCCTTATT

+

BBBBBBFFFFF<FBFFFFFFFFFFFFFFFBBBB<BFFFBFFFFBFFFFFFFFF<FFFFFFFFFFFFBFFFFFFFFBFFFFFFFF/FFFFFB/BFFF<FFFFFFFFBBFBFFBFFFFFFFFFFFFBF

@GWZHISEQ02:315:C9E6MANXX:5:2111:19854:8987

CCTGGGCCCCGGCTCAGGGCCAAGAACAGATGGTACTCAGATAAAGCGAAACTAGCAACAGTTTCTGGAAAGTCCCACCTCAGTTTCAAGTTCCCCAAAAGACCGGGAAAAACCCCAAGCCTTATT

+

BBBBBFFF<FFFFFFFFFFBFFFFFFFFFFFFFFFFFFFFBFFFFFFF</FFFFFFFFBFFFBFFFFFFFFFFFFFFFFFFFFFFFFFFFBFFFFFFFFFFFFFFFFFFFFFFFFFFFFFFFFFFF

@GWZHISEQ02:315:C9E6MANXX:5:2204:4530:78837

CCTGGGCCCCGGCTCAGGGCCAAGAACAGATGGTACTCAGATAAAGCGAAACTAGCAACAGTTTCTGGAAAGTCCCACCTCAGTTTCAAGTTCCCCAAAAGACCGGGAAAAACCCCAAGCCTTATT

+

BBBBBFFFFFFFFFFFFFFFFFFFFFFFFFFFFFFFFFFFFFFFFFFFFFFFFFFFFFFFFFFFFFFFFFFFFFFFFFFFFFFFFFFFFFFFFFFFFFFFFFFFFFFFFFFFFFFFFFFFFFFFFF

@GWZHISEQ02:315:C9E6MANXX:5:2216:6794:80727

CCTGGGCCCCGGCTCAGGGCCAAGAACAGATGGTACTCAGATAAAGCGAAACTAGCAACAGTTTCTGGAAAGTCCCACCTCAGTTTCAAGTTCCCCAAAAGACCGGGAAAAACCCCAAGCCTTATT

+

BBBBBFFFFFFFFFFFFFFFFFFFFFFFFFFFFFFFFFFFFFFFFFFFFFFFFFFFFFFFFFFFFFFFFFFFFFFFFFFFFFFFFFFFFFFFFFFFFFFFFFFFFFFFFFFFFFFFFFFFFFFFFF

@GWZHISEQ02:315:C9E6MANXX:5:1209:18624:3390

CTGGGCCCCGGCTCAGGGCCAAGAACAGATGGTACTCAGATAAAGCGAAACTAGCAACAGTTTCTGGAAAGTCCCACCTCAGTTTCAAGTTCCCCAAAAGACCGGGAAAAACCCCAAGCCTTATTT

+

BB//BBFF<<BFFBFFFBFFF/BFBBBFFBBFFFFFF<F<BFBFFB</77<FBFFFBBFFFBFB/<FFFB<<FFFFFFFFFF<</FFFFF/BBF<FFBFFF<BF<//BB/FFFFFFFF/FFFF<FB

@GWZHISEQ02:315:C9E6MANXX:5:2209:14828:101008

CTGGGCCCCGGCTCAGGGCCAAGAACAGATGGTACTCAGATAAAGCGAAACTAGCAACAGTTTCTGGAAAGTCCCACCTCAGTTTCAAGTTCCCCAAAAGACCGGGAAAAACCCCAAGCCTTATTT

+

BBBBBFFFFFFFFFFFFFFFFFFFFFFFFFFFFFFFFFFFFFFFFFFFFFFFFFFFFFFFFFFFFFFFFFFFFFFFFFFFFFFFFFFFFFFFFFFFFFFFFFFFFFFFFFFFFFFFFFFFFFFFFF

@GWZHISEQ02:315:C9E6MANXX:5:2306:10193:42491

CTGGGCCCCGGCTCAGGGCCAAGAACAGATGGTACTCAGATAAAGCGAAACTAGCAACAGTTTCTGGAAAGTCCCACCTCAGTTTCAAGTTCCCCAAAAGACCGGGAAAAACCCCAAGCCTTATTT

+

BBBBBFFFBFFFFFFFFFFFFFFFFFFFFFFFFFFFFFFFFFFFFFFFFFFFFFFFFFFFFFFFFFFFFFFFFFFFFFFFFFFFFFFFFFFFFFFFFFFFFFFFFFFFFFFFFFFBFFFFFFFFFF

@GWZHISEQ02:315:C9E6MANXX:5:1304:8516:7002

GGCCCCGGCTCAGGGCCAAGAACAGATGGTACTCAGATAAAGCGAAACTAGCAACAGTTTCTGGAAAGTCCCACCTCAGTTTCAAGTTCCCCAAAAGACCGGGAAAAACCCCAAGCCTTATTTAAA

+

BBBBBFFFFFFFFFFFFFFFFFFFFFFFFFFFFFFFFFFFFFFFFFFFFFFFFFFFFFFFFBFFFFFFFFFFFFFFFFFBFFFFFFFFFFFFFFFFFFFFBFFFBFBFFFFFFFFFFFFBFFFFFF

@GWZHISEQ02:315:C9E6MANXX:5:1104:19868:16401

GTTTAAATAAGGCTTGGGGTTTTTCCCGGTCTTTTAGGGAACTTGAAACTGAGGTGGGACTTTCCAGAAACTGTTGCTAGTTTCGCTTTATCTGAGTACCATCTGTTCTTGGCCCTGAGCCGGGGC

+

<BBBBFFFFFFFFFFF/FFFFFFFFFFFFFFFFFFBFFFFFFFBBFFFFFFBFFFFFFFBFFFBBBBF/FF<BFFFFFBF<FFF/F/FFFFFBF/F/<BF/<FBFFFFFFBFFFFBBFBBFFFFFF

@GWZHISEQ02:315:C9E6MANXX:5:1107:8638:99343

GTTTAAATAAGGCTTGGGGTTTTTCCCGGTCTTTTGGGGAACTTGAAACTGAGGTGGGACTTTCCAGAAACTGTTGCTAGTTTCGCTTTATCTGAGTACCATCTGTTCTTGGCCCTGAGCCGGGGC

+

B<BBBBFFFFFFFFFFBFFBFFFFBBFFFFFBFFFFBFFBFFFFFBFFBFFFFFFBFBF/BFF<<BFBBF<FFFFFFFFFFFFFFFFFFFFFFBFFFFFFFFBFFFFFBF//<FFFBFFFFFFFF/

@GWZHISEQ02:315:C9E6MANXX:5:1113:8243:73876

GTTTAAATAAGGCTTGGGGTTTTTCCCGGTCTTTTGGGGAACTTGAAACTGAGGTGGGACTTTCCAGAAACTGTTGCTAGTTTCGCTTTATCTGAGTACCATCTGTTCTTGGCCCTGAGCCGGGGC

+

<B/BBFFFFFFFFFFFFFFFFFFFFFFFFFFFFFFFFFF/FFFFF/<FFFF/FFFFFFFFFFFFFFF<BFFFFFFFFFBFFFBFBFF/FFFFFFFFFFFFFFFFFFFFFFFFFFFFFFFFFFFF<B

@GWZHISEQ02:315:C9E6MANXX:5:1201:13951:31361

GTTTAAATAAGGCTTGGGGTTTTTCCCGGTCTTTTGGGGAACTTGAAACTGAGGTGGGACTTTCCAGAAACTGTTGCTAGTTTCGCTTTATCTGAGTACCATCTGTTCTTGGCCCTGAGCCGGGGC

+

BBBBBFFFFFFFFFFFFFFFFFFFFFFFFFFFFBF<<FFFFFFF<<FFF<FFFFFFFF//BFFFFF<//F<FFBFFBF/<//<<<B7FF//FF/<<<BFF/BFFFFB//</7F//7<F/FF<BB//

@GWZHISEQ02:315:C9E6MANXX:5:1204:15301:77383

GTTTAAATAAGGCTTGGGGTTTTTCCCGGTCTTTTGGGGAACTTGAAACTGAGGTGGGACTTTCCAGAAACTGTTGCTAGTTTCGCTTTATCTGAGTACCATCTGTTCTTGGCCCTGAGCCGGGGC

+

BBBBBFFFFFFFFFFFBFFFFFFFFFFFFFFFFFFBFFF/FFFFFFFFFFFFFFFFFFBFFFFFFFFFFFFFFFFFFFFFBFFFFFFFFFFFFFFFFFFFFFFBFFFBFFF/BBFFFFFFFFFFFB

@GWZHISEQ02:315:C9E6MANXX:5:1205:16918:32046

GTTTAAATAAGGCTTGGGGTTTTTCCCGGTCTTTTGGGGAACTTGAAACTGAGGTGGGACTTTCCAGAAACTGTTGCTAGTTTCGCTTTATCTGAGTACCATCTGTTCTTGGCCCTGAGCCGGGGC

+

BBBBBFFFFFFFFFFFFFFFFFFFFFFFFFFFFFFFFFFFFFFFFFFFFFFFFFFFFFFFFFFFFFFFFFFFFFFFFFFFFFFFFFFFFFFFFFFFFFFFFFFFFFFFFFFBBFFFFFFFFFBFFB

@GWZHISEQ02:315:C9E6MANXX:5:1212:15283:29145

GTTTAAATAAGGCTTGGGGTTTTTCCCGGTCTTTTGGGGAACTTGAAACTGAGGTGGGACTTTCCAGAAACTGTTGCTAGTTTCGCTTTATCTGAGTACCATCTGTTCTTGGCCCTGAGCCGGGGC

+

BBBBBFFFFFFFFFFFFFFFBFFFFFFFF<FFFFFFFFFFFFFFFFBF/FFBFFFFFFBBBFBFFFFFFFFFFFFFFFFFFFFF<BFFFFFFFBFFFFFFFFFFBBFBFFFBFFBFFFFFFFFFF<

@GWZHISEQ02:315:C9E6MANXX:5:2104:18858:41098

GTTTAAATAAGGCTTGGGGTTTTTCCCGGTCTTTTGGGGAACTTGAAACTGAGGTGGGACTTTCCAGAAACTGTTGCTAGTTTCGCTTTATCTGAGTACCATCTGTTCTTGGCCCTGAGCCGGGGC

+

BBBBBFFFFFFFFFFFFFFFFFFFFFFFFFFBFFFFFFFFFFFFBFFFFFFFFFFFFFBFFFFFFFFFFFFFFFFFFFFFFFFFBFFFFFFFFFFFFFFFFFFFFFFFFF</BFFFFFFFFFFFFB

@GWZHISEQ02:315:C9E6MANXX:5:2107:11158:18706

GTTTAAATAAGGCTTGGGGTTTTTCCCGGTCTTTTGGGGAACTTGAAACTGAGGTGGGACTTTCCAGAAACTGTTGCTAGTTTCGCTTTATCTGAGTACCATCTGTTCTTGGCCCTGAGCCGGGGC

+

BBBBBFFFFFFFFFFFFFFFFFFFFFFFFFFFFFFFFFFFFFFFFFFFFFFFFFFFFFFFFFFFFFFFFFFFFFFFFFFFFFFFFFFFFFFFFFFFFFFFFFFFFFFFFFFFFFFFFFFFFFFFFF

@GWZHISEQ02:315:C9E6MANXX:5:2109:11577:30851

GTTTAAATAAGGCTTGGGGTTTTTCCCGGTCTTTTGGGGAACTTGAAACTGAGGTGGGACTTTCCAGAAACTGTTGCTAGTTTCGCTTTATCTGAGTACCATCTGTTCTTGGCCCTGAGCCGGGGC

+

/BBBBFFF//BFBFFBFFFBFFFFFFFFF/<FFFFFFB</<FFFF<BFFFFFBFF//</BFFFFBFFFB/F/<FBBB//FF</B</B7B7FFFF/FFFF<F<F<F/7BB<//BB7//</<FBBBBF

@GWZHISEQ02:315:C9E6MANXX:5:2109:5403:68958

GTTTAAATAAGGCTTGGGGTTTTTCCCGGTCTTTTGGGGAACTTGAAACTGAGGTGGGACTTTCCAGAAACTGTTGCTAGTTTCGCTTTATCTTAGTACCATCTGTTCTTGGCCCTGAGCCGGGGC

+

B/<BBFFFFFFFFFF<FFFFBFFFFB//<FBBFFFFB<FBBBFFFB/</FB<F<F/FF/FFFB<FBFFFF/BFFFBFFBBFFF//F/FFF<FF/<FBBFFBFFFFFFFFFFF<BFFFFFFFF<FFB

@GWZHISEQ02:315:C9E6MANXX:5:2112:9119:60585

GTTTAAATAAGGCTTGGGGTTTTTCCCGGTCTTTTGGGGAACTTGAAACTGAGGTGGGACTTTCCAGAAACTGTTGCTAGTTTCGCTTTATCTGAGTACCATCTGTTCTTGGCCCTGAGCCGGGGC

+

BBBBBFFFFFFFFFFFFFFFBFFFFFFFFBFFFFFFFFFFFFFFFFFFFFFFFFFFFFBFBFFFFFFFBFFFFFFFFFFFFFFFFFBFFFFFFFFFFFFFFFFFFFFFFFBFFFFFFFFFFFFFBB

@GWZHISEQ02:315:C9E6MANXX:5:2203:14901:58192

GTTTAAATAAGGCTTGGGGTTTTTCCCGGTCTTTTGGGGAACTTGAAACTGAGGTGGGACTTTCCAGAAACTGTTGCTAGTTTCGCTTTATCTGAGTACCATCTGTTCTTGGCCCTGAGCCGGGGC

+

<BBBBBFFFFFFFFF<FFFBFFFFFFFFF<FFFFFFFFFFFFFFBFFFBFBFFFFFFFFFFFBBBFFFFFFBFFFFFFFFFFFF<FFFF<FFFFFF<BFFFFFFBBFFFFFBFBBFBFFBFFBFF<

@GWZHISEQ02:315:C9E6MANXX:5:2206:6170:66647

GTTTAAATAAGGCTTGGGGTTTTTCCCGGTCTTTTGGGGAACTTGAAACTGAGGTGGGACTTTCCAGAAACTGTTGCTAGTTTCGCTTTATCTGAGTACCATCTGTTCTTGGCCCTGAGCCGGGGC

+

BB<BBFFFFFFFFFFFFFFBFFFFFFFFFFFFFFFFFFFB<BFFFFFFFFFFF/<FFFFFFFFBFFFFFFFFFFFFFFFFFFFFFFFFFFF7FFFFFFFB7FFFFFFFFFFBBFFFFFFFFFFBF/

@GWZHISEQ02:315:C9E6MANXX:5:2209:17763:28545

GTTTAAATAAGGCTTGGGGTTTTTCCCGGTCTTTTGGGGAACTTGAAACTGAGGTGGGACTTTCCAGAAACTGTTGCTAGTTTCGCTTTATCTGAGTACCATCTGTTCTTGGCCCTGAGCCGGGGC

+

BBBBBFFFFFFFFFFFFFFFFFFFFFFFFFFFFFFFFFFFBFFFFFFFFFBFFFFFFFFFFFFFFFFFBFFFFFFFFFFFFFFFBFFFFFFFFFFFBFFFFFFFFFFBFFBBFBFBF/BFFFFFFF

@GWZHISEQ02:315:C9E6MANXX:5:2209:2555:42375

GTTTAAATAAGGCTTGGGGTTTTTCCCGGTCTTTTGGGGAACTTGAAACTGAGGTGGGACTTTCCAGAAACTGTTGCTAGTTTCGCTTTATCTGAGTACCATCTGTTCTTGGCCCTGAGCCGGGGC

+

/<<<BF/F/B/F//F/</FB/B<F/FB</<F7FFF//7//<FFF/F/<F//<//<</FFFBB/FBFBFFFB</FF<FF//<BB///7FB/7B/7/77<B/7/<B7FFBFF7FFB//7<7/BFBFB<

@GWZHISEQ02:315:C9E6MANXX:5:2209:10872:72280

GTTTAAATAAGGCTTGGGGTTTTTCCCGGTCTTTTGGGGAACTTGAAACTGAGGTGGGACTTTCCAGAAACTTTTGTTAGTTTCCCTTTTTCTGAGTACCATCTTTTTTTGGCCCTGAGCCGGGGC

+

BBBBBFFFFFFFFFFFFFF<FFFFFFFFFFFFFFFFFFFFFFFFFFFFFFFFFFFFFFFFFFFFFFFFF<///<B/<///<FB///<FB///7//<</F//////<7/<BF/<F/////77<FF7B

@GWZHISEQ02:315:C9E6MANXX:5:2209:14828:101008

GTTTAAATAAGGCTTGGGGTTTTTCCCGGTCTTTTGGGGAACTTGAAACTGAGGTGGGACTTTCCAGAAACTGTTGCTAGTTTCGCTTTATCTGAGTACCATCTGTTCTTGGCCCTGAGCCGGGGC

+

BBBBBFFFFFFFFFFFFFFFFFFFFFFFFFFFFFFFFFFFFFFFFFFFFFFFFFFFFFFFFFFFFFFFFFFFFFFFFFFFFFFFFFFFFFFFFFFFFFFFFFFFFFFFFFFFFFFFFFFFFFFFFF

@GWZHISEQ02:315:C9E6MANXX:5:2211:15640:16372

GTTTAAATAAGGCTTGGGGTTTTTCCCGGTCTTTTGGGGAACTTGAAACTGAGGTGGGACTTTCCAGAAACTGTTGCTAGTTTCGCTTTATCTGAGTACCATCTGTTCTTGGCCCTGAGCCGGGGC

+

BBBBBFFFFFFFFFFFFFFBFFFFFFFFFFFFFFFFFFFFFFFF<BFFB/</B<FF/F<F<FFFFFFB/B<F</F</FFF/F</BF/77BFF//BB<FFFF<FF<BFFFFF<FFFFBFFFFFFFFF

@GWZHISEQ02:315:C9E6MANXX:5:2304:4458:98602

GTTTAAATAAGGCTTGGGGTTTTTCCCGGTCTTTTGGGGAACTTGAAACTGAGGTGGGACTTTCCAGAAACTGTTGCTAGTTTCGCTTTATCTGAGTACCATCTGTTCTTGGCCCTGAGCCGGGGC

+

<BBBBFFFFFFFFFFFFFF<FFFFFFFFFFFFFFFFFFFFFFFFFFFFFFFFFFFFFFFBFFFFFBFFFBFFFFFFFFFFFFFFFFBFFFFFFFFFFFFFFFFF<<BFFBBFFFFFFFFBF<BFFB

@GWZHISEQ02:315:C9E6MANXX:5:2306:10193:42491

GTTTAAATAAGGCTTGGGGTTTTTCCCGGTCTTTTGGGGAACTTGAAACTGAGGTGGGACTTTCCAGAAACTGTTGCTAGTTTCGCTTTATCTGAGTACCATCTGTTCTTGGCCCTGAGCCGGGGC

+

BBBBBFFFFFFFFFFFFFFFFFFFFFFFFFFFFFFFFFF<FFFBFFBFFFFFFBFFFFFFFFFFFFFFFFFFFFFFFFFFFFFFFFFFFFFFFFFFFFFBFFFF<FFFFFFFFFFFFFFFFFFFFF

@GWZHISEQ02:315:C9E6MANXX:5:2308:19607:8227

GTTTAAATAAGGCTTGGGGTTTTTCCCGGTCTTTTGGGGAACTTGAAACTGAGGTGGGACTTTCCAGAAACTGTTGCTAGTTTCGCTTTATCTGAGTACCATCTGTTCTTGGCCCTGAGCCGGGGC

+

BBBBBFFFFFBFFFFFFFFFFFFFFFFFFFFFFFF<BFFBFFFFFBFFFFFBFFFFFFBBFFFFFFFBFFFFFFFFBFFFFF//FFFFF<FFFFFFFFFFFFFFFFFFFFFFBFFFFFFFFFFFFB

@GWZHISEQ02:315:C9E6MANXX:5:2312:8945:67883

GTTTAAATAAGGCTTGGGGTTTTTCCCGGTCTTTTGGGGAACTTGAAACTGAGGTGGGACTTTCCAGAAACTGTTGCTAGTTTCGCTTTATCTGAGTACCATCTGTTCTTGGCCCTGAGCCGGGGC

+

BBBBBFFFFFFFFFFFFFFFFFFFFFFFFFFFFFFFFFFFFFFFFFFFFFFFFFFFFFFFFFFFFFFFFFFFFFFFFFFFFFFFFFFFFFFFFFFFFFFFFFFFFFFFFFFFFFFFF<FFFFFFFF

@GWZHISEQ02:315:C9E6MANXX:5:2316:5668:44924

GTTTACATAAGGCTTGGGGTTTTTCCCGGTCTTTTGGGGAACTTGAAACTGAGGTGGGACTTTCCAGAAACTGTTGCTAGTTTCGCTTTATCTGAGTACCATCTGTTCTTGGCCCTGATCTGGGGC

+

BB<BB/FBFFFFBFFFFFFFFFFFFF</BFFFFBBFFBF/<BFFFF<F/FFFFFFFFF</FFFBBBFFFF<F/<BF/FBFFFFFFFFBF/FFFBF<7FF//F/FFBF/BFFF<FBFFB/</<F<FF

@GWZHISEQ02:315:C9E6MANXX:5:2316:21253:72570

GTTTAAATAAGGCTTGGGGTTTTTCCCGGTCTTTTGGGGAACTTGAAACTGAGGTGGGACTTTCCAGAAACTGTTGCTAGTTTCGCTTTATCTGAGTACCATCTGTTCTTGGCCCTGAGCCGGGGC

+

BBBBBFFFFFFFFFFFFFF<FFFFFFFFFFFFFFFFFFFFFFFFFFFFFFFFFFFFFFFFFFFFFFFFFFFFFFFFFFFFFFFFFFFFFFFFFFFFFFFFFFFFFFFFFFFFFBFFBFFF<B<FFB

@GWZHISEQ02:315:C9E6MANXX:5:2215:13473:32338

CCCCGGCTCAGGGCCAAGAACAGATGGTACTCAGATAAAGCGAAACTAGCAACAGTTTCTGGAAAGTCCCACCTCAGTTTCAAGTTCCCCAAAAGACCGGGAAAAACCCCAAGCCTTATTTAAACT

+

/BBBBBBF<FFBB/<FFBFFFBFFFFFFFFFFFFBF<FFFBFFFFFFFFBFFBFBBFFFFFF/FFFFFFFFFFFFF<FFFFBFBBFFFFFFFBFFFFFFFFFFFFFF<FF<BF<FF<FFFFFB/7B

@GWZHISEQ02:315:C9E6MANXX:5:1201:12389:67333

AGTTTAAATAAGGCTTGGGGTTTTTCCCGGTCTTTTGGGGAACTTGAAACTGAGGTGGGACTTTCCAGAAACTGTTGCTAGTTTCGCTTTATCTGAGTACCATCTGTTCTTGGCCCTGAGCCGGGG

+

BBBBBFFFFFFFFFFFFFFFFFFFFFFFFFFFFFFFFFFFFFFFFFFFFFFFFFFFFFFFFFFFFFFFFFFFF<FFFFFFFFFBBBFFFFFFFFFFFFFFFFFFFFFFFFFBFFFFFFFFFFFFFF

@GWZHISEQ02:315:C9E6MANXX:5:1215:13434:26747

GGGGTTTTTCCCGGTCTTTTGGGGAACTTGAAACTGAGGTGGGACTTTCCAGAAACTGTTGCTAGTTTCGCTTTATCTGAGTACCATCTGTTCTTGGCCCTGAGCCGGGGAGATCGGAAGAGCACA

+

B///<FBFFFFFFFB<</<FFF<B<FFFB/FF<FFBFF<<BBB<BF/BFFFFFFF<BFFFBB</BF/B/BBFFFFB<F<<B///<BFFFFF//FB/FF/FFF/<F7<BFFFBFFBF/</7/77F77

@GWZHISEQ02:315:C9E6MANXX:5:1213:16212:94497

CCCGGCTCAGGGCCAAGAACAGATGGTACTCAGATAAAGCGAAACTAGCAACAGTTTCTGGAAAGTCCCACCTCAGTTTCAAGTTCCCCAAAAGACCGGGAAAAACCCCAGATCGGAAGAGCGTCG

+

BBBBBFFFFFFFFFFFFFFFFFFFFFFFFFFFFFFFFFFFFFFFFFFFFFFFFFFFFFFFFFFFFFFFFFFFFFFFFFFFFFFFFFFFFFFFFFFFFFBFFFFFFFFFFFFFFFFFFFFFFFFBFF

@GWZHISEQ02:315:C9E6MANXX:5:1302:20205:53126

CCCGGCTCAGGGCCAAGAACAGATGGTACTCAGATAAAGCGAAACTAGCAACAGTTTCTGGAAAGTCCCACCTCAGGTTCAAGTTCCCCAAAAGACCGGGAAAAACCCCAAGCCTTATTTAAACAG

+

BBBBBFFFFFFFFFFFBFFFFFFFFFFFFFFFFFFFFFFFFFFFFFFFFFFFFFFFFFFFFFFFFFFFFFFFFFFFFFFFFFFFFFFFFFFFFFFFFBFFFFFFFFFFFFFFFFFFFFFFFFFFF<

@GWZHISEQ02:315:C9E6MANXX:5:2302:11666:52542

CCCGGCTCAGGGCCTAGAACAGATGGTACTCAGATAAAGCGAAACTAGCAACAGTTTCTGGAAAGTCCCACCTCAGTTTCAAGTTCCCCAAAAGACCGGGAAAAACCCCAAGCCTTATTTAAACTA

+

BBB<BBFF<BFFFF/FBFF<BF<FFBFFFFFFFFF/BFBFFFFFFFFFFBFFFFBFFFFFF<FFBFFFFFFFFFFFFFFFFFFBFFFFFFFFFBFFFFB<B<FFFFFF/FFF<FFFF<FFFBFFFF

@GWZHISEQ02:315:C9E6MANXX:5:1302:20205:53126

GTTTAAATAAGGCTTGGGGTTTTTCCCGGTCTTTTGGGGAACTTGAACCTGAGGTGGGACTTTCCAGAAACTGTTGCTAGTTTCGCTTTATCTGAGTACCATCTGTTCTTGGCCCTGAGCCGGGAG

+

/B/BBFFFFFFFFBFBBFFFBBFFFFFFF<FBFFFFFFF<FFFFFFFFFFFFFFFFBFF/FFFFFFFFFFFFFFFFFFFFFFFFFFFFB<FBFFFFFFFBFFBFFFFBFFFFFFFFFF<FBFFFF<

@GWZHISEQ02:315:C9E6MANXX:5:1105:11512:23696

CCGGCTCAGGGCCAAGAACAGATGGTACTCAGATAAAGCGAAACTAGCAACAGTTTCTGGAAAGTCCCACCTCAGTTTCAAGTTCCCCAAAAGACCGGAGATCGGAAGAGCGTCGTGTAGGGAAAG

+

BBBBBFFFFFFFFFFFFFFFFFFFFFFFFFFFFFFFFFFFFFFFFFFFFFFFFFFFFFFFFFFFFFFFFFFFFFFFFFFFFFFFFFFFFFFFFFFFFFFFBFFFFFFFFFFFFFFFFFFFBFFFFB

@GWZHISEQ02:315:C9E6MANXX:5:1108:18413:77311

CCGGCTCAGGGCCAAGAACAGATGGTACTCAGATAAAGCGAAACTAGCAACAGTTTCTGGAAAGTCCCACCTCAGTTTCAAGTTCCCCAAAAGACCGGGAAAAACCCCAAGCCTTATTTAAACTAA

+

BB<B<FFBF<<BFFBFBFFFBF/BFBFFFFFF/FFFFFBFFFFFFFFFFFFFFFFFFFF/BFFFFFFFFBF/FFFFBBFFFFFFFFFFFFFFFFFFFBBFFFF7FFFFBBFFFFFFFFFFBFFFFF

@GWZHISEQ02:315:C9E6MANXX:5:1110:14313:35245

CCGGCTCAGGGCCAAGAACAGATGGTACTCAGATAAAGCGAAACTAGCAACAGTTTCTGGAAAGTCCCACCTCAGTTTCAAGTTCCCCAAAAGACCGGGAAAAACCCCAAGCCTTATTTAAACTAA

+

BBBBBFFFFFFFFFFFFFFFFFFFFFFFFFFFFFFFFFFFFFFFFFFFFFFFFFFFFFFFFFFFFFFFFFFFFFFFFFFFFFFFFFFFFFFFFFFFFFFFFFFFFFFFFFFFFFFFFFFFFFFFFF

@GWZHISEQ02:315:C9E6MANXX:5:1302:4047:14637

CCGGCTCAGGGCCAAGAACAGATGGTACTCAGATAAAGCGAAACTAGCAACAGTTTCTGGAAAGTCCCACCTCAGTTTCAAGTTCCCCAAAAGACCGGGAAAAACCCCAAGTCTTATTTAAACTAA

+

BBBBBFFFFFFFFFFFFFFFFFFFFFFFFFFFFFFFFFFFFFFFFFFFFFFFFFFFFFFFFFFFFFFFFFFFFFFFFFFFFFFFFFFFFFFFFFFFFFFFFFFFFFFFFFFFFFFFFFFFFFFFFF

@GWZHISEQ02:315:C9E6MANXX:5:2310:19488:91176

CCGGCTCAGGGCCAAGAACAGATGGTACTCAGATAAAGCGAAACTAGCAACAGTTTCTGGAAAGTCCCACCTCAGTTTCAAGTTCCCCAAAAGACCGGGAAAAACCCCAAGTCTTATTTAAACTAA

+

BBBBBFFFFFFFFFFFFFFFFFFFFFFFFFFFFFFFFFFFFFFFFFFFFFFFFFFFFFFFFFFFFFFFFFFFFFFFFFFFFFFFFFFFFFF<FFFFFFFFFFFFFFBFFFFFFFFFFFFFFFFFFF

@GWZHISEQ02:315:C9E6MANXX:5:1105:11512:23696

CCGGTCTTTTGGGGAACTTGAAACTGAGGTGGGACTTTCCAGAAACTGTTGCTAGTTTCGCTTTATCTGAGTACCATCTGTTCTTGGCCCTGAGCCGGAGATCGGAAGAGCACACGTCTGAACTCC

+

B<BBB<FFFFFFFFFFFFFFFFFFFFFFFFFFFFFFFFFFFFFBFFFBFBFFFFFFFFFFFFFFFFFFFFFFFFFFFFFFFFFFFFFFFFFFFBFFFFFFFFFFFFFFFFFFFFFFFFFFFFFFFF

@GWZHISEQ02:315:C9E6MANXX:5:1104:1552:61263

CGGCTCAGGGCCAAGAACAGATGGTACTCAGATAAAGCGAAACTAGCAACAGTTTCTGGAAAGTCCCACCTCAGTTTCAAGTTCCCCAAAAGACCGGAAAAACCCCAAGCCTTATTTAAACTAACC

+

BBBBBFFFFFFFFFFFFFFFFFFFFFFFFFFFFFFFFFFFFFFFFFFFFFFFFFFFFFFFFFFF<FFBFF<FFFFFBFFFFFFFFFFFBFFFFFFFFF/FFFBFFFFB/FFFFFFBFFBBFFFFF<

@GWZHISEQ02:315:C9E6MANXX:5:1104:5409:75770

CGGCTCAGGGCCAAGAACAGATGGTACTCAGATAAAGCGAAACTAGCAACAGTTTCTGGAAAGTCCCACCTCAGTTTCAAGTTCCCCAAAAGACCGGGAAAAACCCCAAGCCTTATTTAAACTAAC

+

BBBBBFFFFFFFFFFFFFFFFFFFFFFFFFFFFFFFFFFFFFFFFFFFFFFFFFFFFFFFFFFFFFFFFFFFFF<FFFFFFFFFFFFFBFFFFFFFFFFFBFFFFFFFFFFFFFBFBFFFFFFFFF

@GWZHISEQ02:315:C9E6MANXX:5:1109:8132:71496

CGGCTCAGGGCCAAGAACAGATGGTACTCAGATAAAGCGAAACTAGCAACAGTTTCTGGAAAGTCCCACCTCAGTTTCAAGTTCCCCAAAAGACCGGGAAAAACCCCAAGCCTTATTTAAACTAAC

+

BBBBBFFFFFFFFFFFFFFFFFFFFFFFFFFFFFFFFFFFFFFFFFFFFFFFFFFFFFFFFFFFFFFFFFFFFFFFFFFFFFFFFFFFFFFFFFFFFFFFFFFFFFFFFFFFFFFFFFFFFFFFFF

@GWZHISEQ02:315:C9E6MANXX:5:1111:11259:10026

CGGCTCAGGGCCAAGAACAGATGGTACTCAGATAAAGCGAAACTAGCAGCAGTTTCTGGAAAGTCCCACCTCAGTTTCAAGTTCCCCAAAAGACCGGGAAAAACCCCAAGCCTTATTTAAACTAAC

+

BBBBBFFBFFFFFFFFFFFFFFFFFFFFFBFFFFFFFFFFFFFFFFFFFFFF<FFFFFFFFFFFFFF<FFFFFFFFBFFFFBFBFFFFFFFFFFFFFFFFFFFFBFBFFFFFFFFFFFFFFFFFFF

@GWZHISEQ02:315:C9E6MANXX:5:1112:5401:18543

CGGCTCAGGGCCAAGAACAGATGGTACTCAGATAAAGCGAAACTAGCAACAGTTTCTGGAAAGTCCCACCTCAGTTTCAAGTTCCCCAAAAGACCGGGAAAAACCCCAAGCCTTATTTAAACTAAC

+

BBBBBFFFFFFFFFFFFFFFFFFFFFFFFFFFFFFFFFFFFFFFFFFFFFFFFFFFFFFFFFFFFFFFFFFFFFFFFFFFFFFFFFFFFFFFFFFFFFFFFFFFFFFFFFFFFFFFFFFFFFFFFF

@GWZHISEQ02:315:C9E6MANXX:5:1211:16355:91393

CGGCTCAGGGCCAAGAACAGATGGTACTCAGATAAAGCGAAACTAGCAACAGTTTCTGGAAAGTCCCACCTCAGTTTCAAGTTCCCCAAAAGACCGGGAAAAACCCCAAGCCTTATTTAAACTAAC

+

BBBBBFBF<FFFFFB/FFFFFFBFFFFFBB/F//FFFFB/F/BB/<</F/FF/<FFFF/FFB/F</<FFFFFFFFF<FFFFFFFBFBFFFFFFFFF/B/BFBB7/<//BF/<7<BFFFFF7FF///

@GWZHISEQ02:315:C9E6MANXX:5:2102:7666:25574

CGGCTCAGGGCCAAGAACAGATGGTACTCAGATAAAGCGAAACTAGCAACAGTTTCTGGAAAGTCCCACCTCAGTTTCAAGTTCCCCAAAAGACCGGGAAAAACCCCAAGCCTTATTTAAACTAAC

+

BBBBBFFFFFFFFFFFFFFFBFFFFFFFFFFFFFFFFFFFFFFFFFFFFFFFFFFFFFFFFFFFFFFFFFFFFFFFFFFFFFFFFFFFFFFFFFFFFFFFFFFF<FFFFFFFFFFFFFFFFFFFFF

@GWZHISEQ02:315:C9E6MANXX:5:2103:12400:23501

CGGCTCAGGGCCAAGAACAGATGGTACTCAGATAAAGCGAAACTAGCAACAGTTTCTGGAAAGTCCCACCTCAGTTTCAAGTTCCCCAAAAGACCGGGAAAAACCCCAAGCCTTATTTAAACAGAT

+

BBBBBFFFFFFFFFFFFFFFFFFFFFFFFFFFFFFFFFFFFFFFFFFFFFFFFFFFFFFFFFFFFFFFFFFFFFFBFFFFFFFFFFFFFFFFFFFFFFFFFFFFFFFFFFFFFFFFFFFFFFFFFF

@GWZHISEQ02:315:C9E6MANXX:5:2105:13175:69620

CGGCTCAGGGCCAAGAACAGATGGTACTCAGATAAAGCGAAACTAGCAACAGTTTCTGGAAAGTCCCACCTCAGTTTCAAGTTCCCCAAAAGACCGGGAAAAACCCCAAGCCTTATTTAAACTAAC

+

BBBBBFFFFFFFFFFFFFFFFFFFFFFFFFFFFFFFFFFFFFFFFFFFFFFFFFFFFFFFFFFFFFFFFFFFFFFFFFFFFFFFFFFFFFFFFFFFFFFFFFFFFFFFFFFFFFFFFFFFFFFFFF

@GWZHISEQ02:315:C9E6MANXX:5:2107:15462:79842

CGGCTCAGGGCCAAGAACAGATGGTACTCAGATAAAGCGAAACTAGCAACAGTTTCTGGAAAGTCCCACCTCAGTTTCAAGTTCCCCAAAAGACCGGGAAAAACCCCAAGCCTTATTTAAACTAAC

+

BBBBBFFFBFFFFFFFFFFFFFFFFFFFFFFFFFFFFFFFFFFFFFFFFFFFFFFFFFFFFFFFFFFFFFFFFFBFFFFBFFFFFFFFFFFFFFFFFFFFFFFFFFFFFFFFFFFFFFFFFFFFFF

@GWZHISEQ02:315:C9E6MANXX:5:2109:16097:46191

CGGCTCAGGGCCAAGAACAGATGGTACTCAGATAAAGCGAAACTAGCAACAGTTTCTGGAAAGTCCCACCTCAGTTTCAAGTTCCCCAAAAGACCGGGAAAAACCCCAAGCCTTATTTAAACTAAC

+

BBBBBFFFFFFFFFFFFFFFFFFFFFFFFFFFFFFFFFFFFFFFFFFFFFFFFFFFFFFFFFFFFFFFFFFFFFFFFFFFFFFFFFFFFFFFFFFFFFFFFFFFFFFFFFFFFFFFFFFFFFFFFF

@GWZHISEQ02:315:C9E6MANXX:5:2111:1533:68735

CGGCTCAGGGCCAAGAACAGATGGTACTCAGATAAAGCGAAACTAGCAACAGTTTCTGGAAAGTCCCACCTCAGTTTCAAGTTCCCCAAAAGACCGGGAAAAACCCCAAGCCTTATTTAAACTAAC

+

B<BBBFBB//FFFFFFFFFBFBFFFFFFFF<FFF/FFFFFFFFFFFFBFFFFBF<BFFBFFFFFBFF/BBFFFFFFFFFFF<B/B/FFBF<BFFF/B<B</FFFFF<BFFFFBFFFF/BB//BFFB

@GWZHISEQ02:315:C9E6MANXX:5:2111:16461:74408

CGGCTCAGGGCCAAGAACAGATGGTACTCAGATAAAGCGAAACTAGCAACAGTTTCTGGAAAGTCCCACCTCAGTTTCAAGTTCCCCAAAAGACCGGGAAAAACCCCAAGCCTTATTTAAACTAAC

+

BBBBBFFFF<BFFFFFFFFFFFBFFFFBBFFFFFFFFFFFFFFFFFFFFFFFFFFFFFFFFFFFFF<BFBFFFFFFFFFFFFFBFFFFFFFFFFFFFFFFFFFFFFFFFFFFFFFFFFFFFFFFFF

@GWZHISEQ02:315:C9E6MANXX:5:2113:14879:28335

CGGCTCAGGGCCAAGAACAGATGGTACTCAGATAAAGCGAAACTAGCAACAGTTTCTGGAAAGTCCCACCTCAGTTTCAAGTTCCCCAAAAGACCGGGAAAAACCCCAAGCCTTATTTAAACTAAC

+

BBBBBFFFFFFFFFFFFFFFFFFFFFFFFFFFFFFFFFFFFFFFFFFFFFFFFFFFFFFFFFFFFFFFFFFFFFFFFFFFFFFFFFFFFFFFFFFFFFFFFFFFFFFFFFFFFFFFFFFFFFFFFF

@GWZHISEQ02:315:C9E6MANXX:5:2113:12981:80713

CGGCTCAGGGCCAAGAACAGATGGTACTCAGATAAAGCGAAACTAGCAACAGTTTCTGGAAAGTCCCACCTCAGTTTCAAGTTCCCCAAAAGACCGGGAAAAACCCCAAGCCTTATTTAAACTAAC

+

BBBBBFFFFFFFFFFFFFFFFFFFFFFFFFFFFFFFFFFFFFFFFFFFFFFFFFFFFFFFFFFFFFFFFFFFFFFFFFFFFFFFFFFFFFFFFFFFFFFFFFFFFFFFFFFFFFFFFFFFFFFFFF

@GWZHISEQ02:315:C9E6MANXX:5:2116:14999:82331

CGGCTCAGGGCCAAGAACAGATGGTACTCAGATAAAGCGAAACTAGCAACAGTTTCTGGAAAGTCCCACCTCAGTTTCAAGTTCCCCAAAAGACCGGAGATCGGAAGAGCGTCGTGTAGGGAAAGA

+

BBBBBBFFFFFFFFFFFFFFFFFFFFFFFFFFFFFFFFFFFFFFFFFFFFFFFFFFFFFFFFFFFFFFFFFFFFFFFFFFFFFFFFFFFFFFFFFFFFFFFFFFFFFFFFFFFFBFBFFFFFFFFF

@GWZHISEQ02:315:C9E6MANXX:5:2116:5326:87298

CGGCTCAGGGCCAAGAACAGATGGTACTCAGATAAAGCGAAACTAGCAACAGTTTCTGGAAAGTCCCACCTCAGTTTCAAGTTCCCCAAAAGACCGGGAAAAACCCCAAGCCTTATTTAAACTAAC

+

<BBBBF<FBFFFFF<//<BFBBBFFFBF///F<B<FFF//F/7FBF</FF/BFB/BFBF<//FFFFF/<BFB/<<FF/<FFBFFFFFF<BFBBFFFF///B<F<<F/F/<FBFF//77/7/BFF<F

@GWZHISEQ02:315:C9E6MANXX:5:2212:8817:72744

CGGCTCAGGGCCAAGAACAGATGGTACTCAGATAAAGCGAAACTAGCAACAGTTTCTGGAAAGTCCCACCTCAGTTTCAAGTTCCCCAAAAGACCGGGAAAAACCCCAAGCCTTATTTAAACTAAC

+

BBBBBFFFFFFFFFFFFFFFBFFFFFFFFFFFFFFFFFFFFFFFFFFFFFFFFFFFFFFFFFFFFFFFFFFFFFFFFFFFFFFFFFFFFFFFFFFFFFFFFFFFFFFFFFFFBBFFFFFFFFFFFF

@GWZHISEQ02:315:C9E6MANXX:5:2214:2287:41615

CGGCTCAGGGCCAAGAACAGATGGTACTCAGATAAAGCGAAACTAGCAACAGTTTCTGGAAAGTCCCACCTCAGTTTCAAGTTCCCCAAAAGACCGGGAAAAACCCCAAGCCTTATTTAAACTAAC

+

BBBBB<FBFF<<FFFFFBBFFFFFFFFFFFFFFFFFFFFFFFF<FFFFFFFF<FFFFFFFFFFFFFFFFFFFFFBFFFFFB<FFFFFBFFFFFFBFFF/BB/</F/7B7B<FFBBFFFFFFFFFFF

@GWZHISEQ02:315:C9E6MANXX:5:2302:8851:71875

CGGCTCAGGGCCAAGAACAGATGGTACTCAGATAAAGCGAAACTAGCAACAGTTTCTGGAAAGTCCCACCTCAGTTTCAAGTTCCCCAAAAGACCGGGAAAAACCCCAAGCCTTATTTAAACTAAC

+

BBBBBFFFFFBFFFFFFFFFFFFFFFFFFFFFFFFFFFFFFFFFFFFFFFFFFFFFFFFFFFFFFFFFFFFFFFFFFFFFFFFFFFFFFFFFFFFFFFFFFFFFFFFFFFFFFFFFFFBFFFFFFF

@GWZHISEQ02:315:C9E6MANXX:5:2312:17847:25342

CGGCTCAGGGCCAAGAACAGATGGTACTCAGATAAAGCGAAACTAGCAACAGTTTCTGGAAAGTCCCACCTCAGTTTCAAGTTCCCCAAAAGACCGGGAAAAACCCCAAGCCTTATTTAAACTAAC

+

BBBBBFFFFFFFFFFFFFFFFF/BFFFFFBFFFFFFFFFFFFFFFFF<BFFFBFFFFFFFFBFFFFFFFFFFFFFFFFFFF<FFFFFFFFFFFFFFFFFFFFFFFFFFFFFFFFFFFFFBFFFFFF

@GWZHISEQ02:315:C9E6MANXX:5:1114:15399:88022

GTTAGTTTAAATAAGGCTTGGGGTTTTTCCCGGTCTTTTGGGGAACTTGAAACTGAGGTGGGACTTTCCAGAAACTGTTGCTAGTTTCGCTTTATCTGAGTACCATCTGTTCTTGGCCCTGAGCCG

+

/BBBBFFFFFFFFFFFFFFFFFFFFFFFFFFFFFFFFFFFFBFBFFFFFFFFFFF<FFFFBFFFFFFBFFFFFFFFFFFFFFFFFFFFFFFFFFFBFFFFFFFFFFFFFFFFFF/BFFFFFFFFFF

@GWZHISEQ02:315:C9E6MANXX:5:1305:10902:27407

GTTAGTTTAAATAAGGCTTGGGGTTTTTCCCGGTCTTTTGGGGAACTTGAAACTGAGGTGGGACTTTCCAGAAACTGTTGCTAGTTTCGCTCTATCTGAGTACCATCTGTTCTTGGCCCTGAGCCG

+

BBBBBFFFFFFFFFFFFFFFFFFFFFFFFFFFFFFFFFFFFFFFFFFFFFFFFFFFFFFFFFFFFFFFFFFFFFFFFFFFFFFFFFFFFF<FFFFFFFFFFFFFFFFFFFFFFFFFFBFFFFFFFF

@GWZHISEQ02:315:C9E6MANXX:5:1314:20001:54065

GTTAGTTTAAATAAGGCTTGGGGTTTTTCCCGGTCTTTTGGGGAACTTGAAACTGAGGTGGGACTTTCCAGAAACTGTTGCTAGTTTCGCTTTATCTGAGTACCATCTGTTCTTGGCCCTGAGCCG

+

</</BFFFFFFFFFFBF<FBFFFFFFFFF<FFF7BF/FFFBBFFB/BF/FFFFFF/<FFB///FFFF</<F/F/FFFFFF<FFB/FBF<BBFFFFFFF<BFBFFFFFFBFFFFFFFFFFF<BFFF/

@GWZHISEQ02:315:C9E6MANXX:5:2103:12400:23501

GTTTAAATAAGGCTTGGGGTTTTTCCCGGTCTTTTGGGGAACTTGAAACTGAGGTGGGACTTTCCAGAAACTGTTGCTAGTTTCGCTTTATCTGAGTACCATCTGTTCTTGGCCCTGAGCCGAGAT

+

BBBBBFFFFFFFFFFFFFFFFFFFFFFFFFFFFFFFFFFFFFFFFFFFFFFFFFFFFFFFFFFFFFFFFFFFFFFFFFFFFFFFFFFFFFFFFFFFFFFFFFFFFFFFFFF<FFFFFFFFFFFFFF

@GWZHISEQ02:315:C9E6MANXX:5:2112:15622:73943

GTTAGTTTAAATAAGGCTTGGGGTTTTTCCCGGTCTTTTGGGGAACTTGAAACTGAGGTGGGACTTTCCAGAAACTGTTGCTAGTTTCGCTTTATCTGAGTACCATCTGTTCTTGGCCCTGAGCCG

+

</BBBFFFFFFFFFFFFFFFFFFFFFFFFFFBFFF<FFFFFFFFFFFFFFFFFFFFFFFFFFFFFFFBBFFFFFF<BFFFF<F<BBF/<FB<FFFFFFFFFFF<BBFFFFFFFFFFFFFFFFFFFF

@GWZHISEQ02:315:C9E6MANXX:5:2116:14999:82331

CCGGTCTTTTGGGGAACTTGAAACTGAGGTGGGACTTTCCAGAAACTGTTGCTAGTTTCGCTTTATCTGAGTACCATCTGTTCTTGGCCCTGAGCCGAGATCGGAAGAGCACACGTCTGAACTCCA

+

BBBBBFFFFFFFFFFFFFFFFFFFFFFFFFFFFFFFFBFFFFFFFFFFFFFFFFFFFFFFFFFFFFFFFFFFFFFFFFFFFFFFFFFFFFFFFFFFFFFFFFFFFFFFFFFFFFFFFFFFFFFFFB

@GWZHISEQ02:315:C9E6MANXX:5:2210:17763:6131

GTTAGTTTAAATAAGGCTTGGGGTTTTTCCCGGTCTTTTGGGGAACTTGAAACTGAGGTGGGACTTTCCAGAAACTGTTGCTAGTTTCGCTTTATCTGAGTACCATCTGTTCTTGGCCCTGAGCCG

+

BBBBBFFFFFFFFFFFFFFFFFFFFFFFFFFFFFFFFFFFFFFFFFFFFFFFFFFFFFFFFFFFFFFFFFFF/FFFFFFFFFFFFFFFFFFFFFFFFFFFFFFFFFFF<FBFFFBFBFFFFFFFF<

@GWZHISEQ02:315:C9E6MANXX:5:2210:1726:96254

GTTAGTTTAAATAAGGCTTGGGGTTTTTCCCGGTCTTTTGGGGAACTTGAAACTGAGGTGGGACTTTCCAGAAACTGTTGCTAGTTTCGCTTTATCTGAGTACCATCTGTTCTTGGCCCTGAGCCG

+

BBBBBF<FFFFF/FFFBFBFFFFBFFFFFFFFFFFFFFFFFFF/</FFF<FFFFFFFFFFFFFFF/FFFFFFBFBBFBFBFF/BFFB<F<<FF<BFFFB/FF/FFFFF7B//</<<FFBB/B//77

@GWZHISEQ02:315:C9E6MANXX:5:2214:2287:41615

GTTAGTTTAAATAAGGCTTGGGGTTTTTCCCGGTCTTTTGGGGAACTTGAAACTGAGGTGGGACTTTCCAGAAACTGTTGCTAGTTTCGCTTTATCTGAGTACCATCTGTTCTTGGCCCTGAGCCG

+

<BBBBFFFF/F/FFFFFFFFBFFFBFFFFFBFB7/FF<FFFF/BF/FF/BFFFFF<FBFFF/FFFFFFB<FBB/FF<FFFFFBBFBFFBFFFF/7</BB/FFF/<FFFFFF/BF///7<//BB7FF

@GWZHISEQ02:315:C9E6MANXX:5:2302:20404:7798

GTTAGTTTAAATAAGGCTTGGGGTTTTTCCCGGTCTTTTGGGGAACTTGAAACTGAGGTGGGACTTTCCAGAAACTGTTGCTAGTTTCGCTTTATCTGAGTACCATCTGTTCTTGGCCCTGAGCCG

+

B</BBFFFF<FFFFFBFFFFF<B/7FFFBB<F<B<BF<BBFFB/7<FFFFFFFFFB/FFFFFBFFBFFFFFFF</FFFB//</BFFF/<BFFF<FFF<B<7FF/BB/FBFFFFB////7FB/<BF/

@GWZHISEQ02:315:C9E6MANXX:5:1212:10726:37297

GGCTCAGGGCCAAGAACAGATGGTACTCAGATAAAGCGAAACTAGCAACAGTTTCTGGAAAGTCCCACCTCAGTTTCAAGTTCCCCAAAAGACCGGGAAAAACCCCAAGCCTTATTTAAACTAACC

+

BBBBBFFFFFFFFFBFFFFFFFFFFFFFFFFFFFFFFFFFFFFFFFFFBFFFFFFFFFFFFFFFFFFFBBFFFFFFFFFFBBBFFFFFFFFBFFFFFFFFFBFBFFFFFFFFFFFFFFFFFFBF<B

@GWZHISEQ02:315:C9E6MANXX:5:2316:8086:44011

GGCTCAGGGCCAAGAACAGATGGTACTCAGATAAAGCGAAACTAGCAACAGTTTCTGGAAAGTCCCACCTCAGTTTCAAGTTCCCCAAAAGACCGGGAAAAACCCCAAGCAGATCGGAAGAGCGTC

+

BBBBBFFFFFFFFFFFFFFFFFFFFFFFFFFFFFFFFFFFFFFFFFFFFFFFFFFFFFFFFFFFFFFFFFFFFFBFFFFFFFFFFFFBFFFFFFFFFFFFFBFFBBFFFFFFFFFFFBFF<FBF7B

@GWZHISEQ02:315:C9E6MANXX:5:1108:19029:18865

GGTTAGTTTAAATAAGGCTTGGGGTTTTTCCCGGTCTTTTGGGGAACTTGAAACTGAGGTGGGACTTTCCAGAAACTGTTGCTAGTTTCGCTTTATCTGAGTACCATCTGTTCTTGGCCCTGAGCC

+

BBBBBFFFFFFFFFFFFFFFFFFFFFFFFFFFFFFFFFFFFFFFFFFFFFFFFFFFFFFFFFFFFFFFFFFFFFFFFFFFFFFFFFFFFBFFFFFFFFFFFFFFFFFFFFFFFFFFFFFFFFFFFF

@GWZHISEQ02:315:C9E6MANXX:5:1306:20349:8180

GGTTAGTTTAAATAAGGCTTGGGGTTTTTCCCGGTCTTTTGGGGAACTTGAAACTGAGGTGGGACTTTCCAGAAACTGTTGCTAGTTTCGCTTTATCTGAGTACCATCTGTTCTTGGCCCTGAGCC

+

<BBBBFFFFFFFFFFFFFFFFFFFFFFFFFFFFFFFFFFFFFFF7FFFFFFFFFFFFFFFFFF<BFFFBFFF/BFFFFFFFFFB<FFFFFFFFFFFFFFBFFFFFFFFFFFFBBFFFFFFFFFFFF

@GWZHISEQ02:315:C9E6MANXX:5:1309:3196:88870

GGTTAGTTTAAATAAGGCTTGGGGTTTTTCCCGGTCTTTTGGGGAACTTGAAACTGAGGTGGGACTTTCCAGAAACTGTTGCTAGTTTCGCTTTATCTGAGTACCATCTGTTCTTGGCCCTGAGCC

+

BBBBBFFFFFFFFFFFFFFFFFFFFFFFFFFFFFFFFFFFFFFFFFFFFBFFFFFFFFFFFFFFFFFFFFFFFFFFFFFFFFFFFFFFFFFFFFFFFFFFFFFFFFFFFFFBFFFFFFFFFFFFFF

@GWZHISEQ02:315:C9E6MANXX:5:2106:6278:75812

GGTTAGTTTAAATAAGGCTTGGGGTTTTTCCCGGTCTTTTGGGGAACTTGAAACTGAGGTGGGACTTTCCAGAAACTGTTGCTAGTTTCGCTTTATCTAAGTACCATCTGTTCTTGGCCCTGAGCC

+

BBBBBFFFFFFFFFFFFFFFFFFFFFFFFFFFFFFFFFFFFFFFFFFFFFFFFFFFFFFFFFFBFFFFFFFFFFFFFFFFFFFFFFFFFFFFFFFFFFFFFFFFFFFFFFFFFFFFFFFFFF<BFF

@GWZHISEQ02:315:C9E6MANXX:5:2108:5689:73557

GGTTAGTTTAAATAAGGCTTGGGGTTTTTCCCGGTCTTTTGGGGAACTTGAAACTGAGGTGGGACTTTCCAGAAACTGTTGCTAGTTTCGCTTTATCTGAGTACCATCTGTTCTTGGCCCTGAGCC

+

BBBBBFFFFFFFFFFFFFFFFFFFFFFFFFFFFFFFFFFFFFFFBFFFFFFFFFFFFFFFFFFFFFFFFFFFFBFFFFFFFFFFFFFFFFFFFFBFFFFFFFFFFFFFFFFFFFF<BFFFB<<FFF

@GWZHISEQ02:315:C9E6MANXX:5:2113:14879:28335

GGTTAGTTTAAATAAGGCTTGGGGTTTTTCCCGGTCTTTTGGGGAACTTGAAACTGAGGTGGGACTTTCCAGAAACTGTTGCTAGTTTCGCTTTATCTGAGTACCATCTGTTCTTGGCCCTGAGCC

+

BBBBBFFFFFFFFFFFFFFFFFFFFFFFFFFFFFFFFFFFFBFFFFFFFFFFFFFFF/FFFFFFFFFFFFFFFFFFFFFFFFFFFBFFFFFFFFFFFFF/FFFBFFFFFFFFFFFFFFFFFFFFFF

@GWZHISEQ02:315:C9E6MANXX:5:2113:7415:68006

GGTTAGTTTAAATAAGGCTTGGGGTTTTTCCCGGTCTTTTGGGGAACTTGAAACTGAGGTGGGACTTTCCAGAAACTGTTGCTAGTTTCGCTTTATCTGAGTACCATCTGTTCTTGGCCCTGAGCC

+

BBBBBBFFFFFFFFFFFFFFFFFFFFFFFFFFFFBFFFFFFFFFFFFFFFBFFFFFFFFFFFFFFFFFFFFFFFFFFFFFFFFFF<FFFFFFFFFFF<<BFFFFFFFFFFFBFFFFFFFFFFFFFF

@GWZHISEQ02:315:C9E6MANXX:5:2116:19037:99769

GGTTAGTTTAAATAAGGCTTGGGGTTTTTCCCGGTCTTTTGGGGAACTTGAAACTGAGGTGGGACTTTCCAGAAACTGTTGCTAGTTTCGCTTTATCTGAGTACCATCTGTTCTTGGCCCTGAGCC

+

BBBBBFFFFFFFFFFFFFFFFFFFFFFFFFFFFFFFFFFFFFFFFFFFFFFFFFFFFFFFFFFFFFFFFFFFFFFFFFFFFFFFFFFFFFFFFFFFFFFFFFFFFFFFFFFFFFFFFFFFFFFFFF

@GWZHISEQ02:315:C9E6MANXX:5:2308:15881:88285

GGTTAGTTTAAATAAGGCTTGGGGTTTTTCCCGGTCTTTTGGGGAACTTGAAACTGAGGTGGGACTTTCCAGAAACTGTTGCTAGTTTCGCTTTATCTGAGTACCATCTGTTCTTGGCCCTGAGCC

+

BB/<BFFFFFFFFBFFBBFFFFFFFFFFFBFFFFF<FBFFFFBFFFFFFBFFFFFFFFFFFFBF/FFBBFFFB/FFBBFFFBF<FFFB/<B<FF/BFFBFBFFFFFFFBFBFFFFBFFFBFFFFBB

@GWZHISEQ02:315:C9E6MANXX:5:2316:8086:44011

GCTTGGGGTTTTTCCCGGTCTTTTGGGGAACTTGAAACTGAGGTGGGACTTTCCAGAAACTGTTGCTAGTTTCGCTTTATCTGAGTACCATCTGTTCTTGGCCCTGAGCCAGATCGGAAGAGCACA

+

BBBBBBFFFFFFFFFFFFFFFFFFFFFFFFFFFFFFFFFFFFFFFFFFFFFFFFFFFFFFFFFFFFFFFFFFFFFBFFFFFFFFFFFFFFFFFFFFFFFFFFFFFFFFFFFFFFFFFFFFFFFBFB

@GWZHISEQ02:315:C9E6MANXX:5:2114:4013:47860

GCTCAGGGCCAAGAACAGATGGTACTCAGATAAAGCGAAACTAGCAACAGTTTCTGGAAAGTCCCACCTCAGTTTCAAGTTCCCCAAAAGACCGGGAAAAACCCCAAGCCTTATTTAAACTAACCA

+

BBBBBFFFBFFBFFFFFF<FFFFFFFFFFFFBFFFFFFFFFFFFFFFFFFFFFFFFFFFFBFFFFFFFFFFFFFFFFFFFFFFFFFFFFFFFFFBFFF/FFFFFFFFFFFFFFFFFFFFBFFFFFF

@GWZHISEQ02:315:C9E6MANXX:5:1102:14341:76003

CTCAGGGCCAAGAACAGATGGTACTCAGATAAAGCGAAACTAGCAACAGTTTCTGGAAAGTCCCACCTCAGTTTCAAGTTCCCCAAAAGACCGGGAAAAACCCCAAGCCTTATTTAAACTAACCAA

+

BBBBBFFFFFFFFFFFFFFFFFFFFFFFFFFFFFFFFFFFFFFFFFFFFFFFFFFFFFFFFFFFFFFFFFFFFFFFFFFFFFFFFFFFFFFFFFFFFFFFFFFFFFFFFFFFFFFFFFFFFFFFFF

@GWZHISEQ02:315:C9E6MANXX:5:1108:17762:77555

CTCAGGGCCAAGAACAGATGGTACTCAGATAAAGCGAAACTAGCAACAGTTTCTGGAAAGTCCCACCTCAGTTTCAAGTTCCCCAAAAGACCGGGAAAAACCCCAAGCCTTATTTAAACTAACCAA

+

BBBBBFFFFFFFFFFFFFFFFFFFFFFFFFFFFFFFFFFFFFFFFFFFFFFFFFFFFFFFFFFFFFFFFFFFFFFFFFFFFFFFFFFFFFFFFFFFFFFFFFFFFFFFFFFFFFFFFFFFFFFFFF

@GWZHISEQ02:315:C9E6MANXX:5:1109:17012:22140

CTCAGGGCCAGAACAGATGGTACTCAGATAAAGCGAAACTAGCAACAGTTTCTGGAAAGTCCCACCTCAGTTTCAAGTTCCCCAAAAGACCGGGAAAAACCCCAAGCCTTATTTAAACTAACCAAT

+

BBBBBFFFFFFFFFFFFFFFFFFFFFFFFFFFFFFFFFFFFFFFFFFFFFFFFFFFFFFFFFFFFFFFFFFFFFFFFFFFFFFFFFFFFFFFFFFFFFFFFFFFFFFFFFBFFFFFFFFFFFFFFF

@GWZHISEQ02:315:C9E6MANXX:5:1113:17157:66817

CTCAGGGCCAAGAACAGATGGTACTCAGATAAAGCGAAACTAGCAACAGTTTCTGGAAAGTCCCACCTCAGTTTCAAGTTCCCCAAAAGACCGGGAAAAACCCCAAGCCTTATTTAAACTAACCAA

+

BBBBBFFFFFFFFFFFFFFFFFFFFFFFFFFFFFFFFFFFFFFFFFFFFFFFFFFFFFFFFFFFFFFFFFFFFFFFFFFFFFFFFFFFFFFFFFFFFFFFFFFFFFFFFFFFFFFFFFFFFFFFFF

@GWZHISEQ02:315:C9E6MANXX:5:1114:6108:90065

CTCAGGGCCAAGAACAGATGGTACTCAGATAAAGCGAAACTAGCAACAGTTTCTGGAAAGTCCCACCTCAGTTTCAAGTTCCCCAAAAGACCGGGAAAAACCCCAAGCCTTATTTAAACTAACCAA

+

BBBBBFFFFBFFFFFFFFFFFFFFFFFFFFFFFFFFFFFFFFFFFFFFFFFFFF<FFFFFFFFFFFFFFFFFFFFFFFFFFFFBFFFFBFFFFFFFFFFFFFFFFFFFFFFBFFFFFFFFFFFFFF

@GWZHISEQ02:315:C9E6MANXX:5:1205:6327:43703

CTCAGGGCCAAGAACAGATGGTACTCAGATAAAGCGAAACTAGCAACAGTTTCTGGAAAGTCCCACCTCAGTTTCAAGTTCCCCAAAAGACCGGGAAAAACCCCAAGCCTTATTTAAACTAACCAA

+

BBBBBFFFFFFFFFFFFFFFFFFFFFFFFFFFFFFFFFFFFFFFFFFFFFFFFFFFFFFFFFFFFFFFFFFFFFFFFFFFFFFFFFFFFFFFFFBFFFFFFFFFFFFFFFFFFFFFFFFFFFFFFF

@GWZHISEQ02:315:C9E6MANXX:5:1206:6701:39896

CTCAGGGCCAAGAACAGATGGTACTCAGATAAAGCGAAACTAGCAACAGTTTCTGGAAAGTCCCACCTCAGTTTCAAGTTCCCCAAAAGACCGGGAAAAACCCCAAGCCTTATTTAAACTAACCAA

+

BBBBBFFFFFFFFFFFFFFFFFFFFFFFFFFFFFFFFFFFFFFFFFFFFFFFFFFFFFFFFFFFFFFFFFFFFFFFFFFFFFFFFFFFFFFFFFFFFFFFFFFFFFFFFFFFFFFFFFFFFFFFFF

@GWZHISEQ02:315:C9E6MANXX:5:1210:2382:57063

CTCAGGGCCAAGAACAGATGGTACTCAGATAAAGCGAAACTAGCAACAGTTTCTGGAAAGTCCCACCTCAGTTTCAAGTTCCCCAAAAGACCGGGAAAAACCCCAAGCCTTATTTAAACTAACCAA

+

BBBBBFFFFFFFFFFFFFFFFFFFFFFFFFFFFFFFFFFFFFFFFFFFFFFFFFFFFFFFFFFFFFFFFFFFFFFFFFFFFFFFFFFFFFFFFFFFFFFFFFFFFFFFFFFFFFFFFFFFFFFFFF

@GWZHISEQ02:315:C9E6MANXX:5:1216:6196:37579

CTCAGGGCCAAGAACAGATGGTACTCAGATAAAGCGAAACTAGCAACAGTTTCTGGAAAGTCCCACCTCAGTTTCAAGTTCCCCAAAAGACCGGGAAAAACCCCAAGCCTTATTTAAACTAACCAA

+

BBBBBFFBBFFFFFFFFFFFFFFFFFFFFFFFFFFFFFFFFFFFFFFFFFFFFFFFFFFFFFFFFFFFFFFBFFFFFFFFFFFFFFFFFFFFFFFFFFFFFFFFFFFFFFFFFFFFFFFFFFFFFF

@GWZHISEQ02:315:C9E6MANXX:5:1315:18260:75259

CTCAGGGCCAAGAACAGATGGTACTCAGATAAAGCGAAACTAGCAACAGTTTCTGGAAAGTCCCACCTCAGTTTCAAGTTCCCCAAAAGACCGGGAAAAACCCCAAGCCTTATTTAAACTAACCAA

+

BBBBB/FFBFFFFBFFFF<BFFFBFFBFFFFFFFF//FF<FFF/FFFFFFFFFFFF<F//FFFFFFBFFFFF/BBFFFB/<FFFFFFFFFFFFFFFFFFFFFB<F<FFFFFF<F/FFFFFFFFFFF

@GWZHISEQ02:315:C9E6MANXX:5:2104:20640:72485

CTCAGGGCCAAGAACAGATGGTACTCAGATAAAGCGAAACTAGCAACAGTTTCTGGAAAGTCCCACCTCAGTTTCAAGTTCCCCAAAAGACCGGGAAAAACCCCAAGCCTTATTTAAACTAACCAA

+

BBBBBFFFFFFFFFBFFBBFFFFBFF/FFFFFFFFFFFF/BBFFFFFFFBFFFFFBF<FFFBFFFFBFFBFF/FFFFF<F/FBFFFFFF<FFFFFFFFFFFF<BFBFFFFFFFFFFFFFFFFBFFF

@GWZHISEQ02:315:C9E6MANXX:5:2116:18420:40013

CTCAGGGCCAAGAACAGATGGTACTCAGATAAAGCGAAACTAGCAACAGTTTCTGGAAAGTCCCACCTCAGTTTCAAGTTCCCCAAAAGACCGGGAAAAACCCCAAGCCTTATTTAAACTAACCAA

+

BBBBBFFFFFFFFFFFFFFFFFFFFFFFFFFFFFFFFFFFFFFFFFFFFFFFFFFFFFFFFFFFFFFFFFFFFFFFFFFFFFFFFFFFFF<FFFFFFFFFFFFFFFFFFFFFFFFFFFFFFFFFFF

@GWZHISEQ02:315:C9E6MANXX:5:2209:14196:76446

CTCAGGGCCAAGAACAGATGGTACTCAGATAAAGCGAAACTAGCAACAGTTTCTGGAAAGTCCCACCTCAGTTTCAAGTTCCCCAAAAGACCGGGAAAAACCCCAAGCCTTATTTAAACTAACCAA

+

BB<BBFFFFFFFFFFFFFFFFFF<FFFFFFFFFFFFFFFFFBFFFFFFFFFFFFFFFFFFFFFFFFBFFFFBFFFFFFFFFFFFFBFFFFFFFFF7<FFFFFFFFFFFFFFFFFFFFBFFFFFFFF

@GWZHISEQ02:315:C9E6MANXX:5:2302:2787:30827

CTCAGGGCCAAGAACAGATGGTACTCAGATAAAGCGAAACTAGCAACAGTTTCTGGAAAGTCCCACCTCAGTTTCAAGTTCCCCAAAAGACCGGGAAAAACCCCAAGCCTTATTTAAACTAACCAA

+

BBBBBFFFFFFFFFFFFFFFFFFFFFFFFFFFFFFFFFFFFFFFFFFFFFFFFFFFFFFFFFFFFFFFFFFBFFFFFFFFFFFFFFFFFFFFFFFFFFFFFFFFFFFFFFFBFFFFFFFFFFFFFF

@GWZHISEQ02:315:C9E6MANXX:5:2306:13630:74448

CTCAGGGCCAAGAACAGATGGTACTCAGATAAAGCGAAACTAGCAACAGTTTCTGGAAAGTCCCACCTCAGTTTCAAGTTCCCCAAAAGACCGGGAAAAACCCCAAGCCTTATTTAAACTAACCAA

+

BBBBBFFFFFFFFFFFFFFFFFFFFFFFFFFFFFFFFFFFFFFFFFFFFFFFFFFFFFFFFFFFFFFFFFFFFFFFFFFFFFFFFFFFFFFFFFFFFFFFFFFFFFFFFFFFFFFFFFFFFFFFFF

@GWZHISEQ02:315:C9E6MANXX:5:2307:17441:8256

CTCAGGGCCAAGAACAGATGGTACTCAGATAAAGCGAAACTAGCAACAGTTTCTGGAAAGTCCCACCTCAGTTTCAAGTTCCCCAAAAGACCGGGAAAAACCCCAAGCCTTATTTAAACTAACAGA

+

BBBBBFFFFFFFFFFFFFFFFFFFFFFFFFFFFFFFFFFFFFFFFFFFFFFFFFFFFFFFFFFFFFFFFFFFFFFFFFFFFFFFFFFFFFFFFFFFFFFFFFFFFFFFFFFFFFFFFFFFFFFFFF

@GWZHISEQ02:315:C9E6MANXX:5:2314:1666:18946

CTCAGGGCCAAGAACAGATGGTACTCAGATAAAGCGAAACTAGCAACAGTTTCTGGAAAGTCCCACCTCAGTTTCAAGTTCCCCAAAAGACCGGGAAAAACCCCAAGCCTTATTTAAACAGATCGG

+

BBBBBFFFFFFFFFFFFFFFFFFFFFFFFFFFFFFFFFFFFFFFFFFFFFFFFFFFFFFFFFFFFFFFFFFFFFFFFFFFFFFFFFFFFFFFFFFFFFFFFFFFFFFFFFFFFFFFFFFFFFFFFF

@GWZHISEQ02:315:C9E6MANXX:5:2314:17944:21322

CTCAGGGCCAAGAACAGATGGTACTCAGATAAAGCGAAACTAGCAACAGTTTCTGGAAAGTCCCACCTCAGTTTCAAGTTCCCCAAAAGACCGGGAAAAACCCCAAGCCTTATTTAAACTAACCAA

+

BBBBBFFFFFFFFFFFFFFFFFFFFFFFFFFFFFFFFFFFFFFFFFFFFFFFFFFFFFFFFFFFFFFFFFFFFFFFFFFFFFFFFFFFFFFFFFFFFFFFFFFFBFFFFFFFFFFFFFFFFFFFFF

@GWZHISEQ02:315:C9E6MANXX:5:2307:17441:8256

GTTAGTTTAAATAAGGCTTGGGGTTTTTCCCGGTCTTTTGGGGAACTTGAAACTGAGGTGGGACTTTCCAGAAACTGTTGCTAGTTTCGCTTTATCTGAGTACCATCTGTTCTTGGCCCTGAGAGA

+

BBBBBFFFFFFFFFFFFFFFFFFFFFFFFFFFFFFFFFFFFFFFFFFFFFFFFFFFFFFFFFFFFFFFFFFFFFFFFFFFFFFFFFFFFFFFFFFFFFFFFFFFFFFFFFFFFFBFFFFFFFFFFB

@GWZHISEQ02:315:C9E6MANXX:5:2314:1666:18946

GTTTAAATAAGGCTTGGGGTTTTTCCCGGTCTTTTGGGGAACTTGAAACTGAGGTGGGACTTTCCAGAAACTGTTGCTAGTTTCGCTTTATCTGAGTACCATCTGTTCTTGGCCCTGAGAGATCGG

+

BBBBBFFFFFFFFFFFFFF/FFFFFFFFFFFFFFFFFFFFFFFFFFFFFFFFFFFFFFFFFFFFFFFFFFFFFFFFFFFFBFFFFFFFFFFFFFFBFFFFFFBBFFFFFFBBFFFFFFFFFFFFFF

@GWZHISEQ02:315:C9E6MANXX:5:2105:18032:11712

ATTGGTTAGTTTAAATAAGGCTTGGGGTTTTTCCCGGTCTTTTGGGGAACTTGAAACTGAGGTGGGACTTTCCAGAAACTGTTGCTAGTTTCGCTTTATCTGAGTACCATCTGTTCTTGGCCCTGA

+

BBBBBFFFFFFFFFFFFFFFFFFFFFFFFFFFFFFFFBFBFFFFFFFFFFFFFFFFFFFFFFFFFFFFFFFFFFFFFFFFFFFFFFFFFFFFFFFFFFFFFFFFFFFFFFFFFFFFFFBFFFFFF/

@GWZHISEQ02:315:C9E6MANXX:5:2108:16904:24657

ATTGGTTAGTTTAAATAAGGCTTGGGGTTTTTCCCGGTCTTTTGGGGAACTTGAAACTGAGGTGGGACTTTCCAGAAACTGTTGCTAGTTTCGCTTTATCTGAGTACCATCTGTTCTTGGCCCTGA

+

BBBBBFFFFFFFFFFFFFFFFFFFFFFFFFFFFFFFFFBFFFFFFFF<FFFFF<FFF<BFFBFFFFBFFFFFFFFFFFFFFFFFFFFFBFFFBF<FFFFFFFB<FFFFFFFFFFFFFF/BFFFFF/

@GWZHISEQ02:315:C9E6MANXX:5:2210:14096:36957

ATTGGTTAGTTTAAATAAGGCTTGGGGTTTTTCCCGGTCTTTTGGGGAACTTGAAACTGAGGTGGGACTTTCCAGAAACTGTTGCTAGTTTCGCTTTATCTGAGTACCATCTGTTCTTGGCCCTGA

+

BBBBBFFFFFFFFFFFFFFFFFFFFFFFFFFFFFFFFFFFFFFFFFFFBFFFFFFFFFFFFFFFFFFFFFFFFFFFFFFFFFFFFFFFFFFFFFFFFFFFFFFFFFFFFFFFFFFFFFBFFFFFFF

@GWZHISEQ02:315:C9E6MANXX:5:1201:2122:46874

CAGGGCCAAGAACAGATGGTACTCAGATAAAGCGAAACTAGCAACAGTTTCTGGAAAGTCCCACCTCAGTTTCAAGTTCCCCAAAAGACCGGGAAAAACCCCAAGCCTTATTTAAACTAACCAATC

+

BBBBBFFFFFFBFFFFFFBFFFFFFFFFFFFFFFFFFFFFFFFFFFFFFFFFFFFFFFFFFFFFFFFFFFFFFFFFFFFFFFFFFFFFFFFFFFFFFFFFFFFFFFFFFFFFFFFFFFFFFFFFFF

@GWZHISEQ02:315:C9E6MANXX:5:1311:8766:4882

CAGGGCCAAGAACAGATGGTACTCAGATAAAGCGAAACTAGCAACAGTTTCTGGAAAGTCCCACCTCAGTTTCAAGTTCCCCAAAAGACCGGGAAAAACCCCAAGCCTTATTTAAACTAACCAATC

+

BBBBBFFFFFFFFFFFFFFFFFFFFFFFFFFFFFFFFFFFFFFFFFFFFFFFFFFFFFFFFFFFFFFFFFFFFFFFFFFFFFFFFFFFFFFFFFFFFFFFFFFFFFFFFFFFFFFFFFFFFFFFFF

@GWZHISEQ02:315:C9E6MANXX:5:2301:4648:81901

CAGGGCCAAGAACAGATGGTACTCAGATAAAGCGAAACTAGCAACAGTTTCTGGAAAGTCCCACCTCAGTTTCAAGTTCCCCAAAAGACCGGGAAAAACCCCAAGCCTTATTTAAACTAACCAATC

+

<BBBBF<BFFFFBFFBFFFFFFBFFBFFFFFFFFFFFFFFFFFFFFFFFFFFFFFFFFFFFFFFFFFFFFFFFFFBFFFFFFFFFFFFFFFFFFFF<FFFFBFBFFFFFFFFFFBFFF<FFFFFFF

@GWZHISEQ02:315:C9E6MANXX:5:2214:1949:21885

GATTGGTTAGTTTAAATAAGGCTTGGGGTTTTTCCCGGTCTTTTGGGGAACTTGAAACTGAGGTGGGACTTTCCAGAAACTGTTGCTAGTTTCGCTTTATCTGAGTACCATCGGTTCTTGGCCCTG

+

BBBBBFFFFFFFFFFFFFFFFFFFFFFFFFFFFBBFBF7/<FFFFF/FFFFFFB<<BFFFFFBFFFFFF/FFFBBFFFBFFFFFBFFFFFFFFFFFFFBFFFFFF/BFF<BB/////BFFFFFFFF

@GWZHISEQ02:315:C9E6MANXX:5:2109:16097:46191

TGATTGGTTAGTTTAAATAAGGCTTGGGGTTTTTCCCGGTCTTTTGGGGAACTTGAAACTGAGGTGGGACTTTCCAGAAACTGTTGCTAGTTTCGCTTTATCTGAGTACCATCTGTTCTTGGCCCT

+

BBBBBFFFFBFFFFFFFFFFFFFFFFFFFFFFFFFFFFFFFFFFFFFFFFBFFFFFFFFFFFFFFFFF<FFFFFFFFFBFFFFFFFFFFFFFFFFFFFFFFFFFFFFFFFFFFFFFFFFFFFFFF<

@GWZHISEQ02:315:C9E6MANXX:5:2110:9124:85678

TGATTGGTTAGTTTAAATAAGGCTTGGGGTTTTTCCCGGTCTTTTGGGGAACTTGAAACTGAGGTGGGACTTTCCAGAAACTGTTGCTAGTTTCGCTTTATCTGAGTACCATCTGTTCTTGGCCCT

+

BBBBBFFFFFFFFFFFFFFFFFFFFFFFFBFFFFFFFFFFFFFFFFFFFBFFFFFFFFFFFFFFFFFFFFFFFFFFFFFFFFFFFFFFFF/FFFFFFFFFFFBF///BFBB<//<7B/FBBFFFF/

@GWZHISEQ02:315:C9E6MANXX:5:1107:6726:29354

CTGATTGGTTAGTTTAAATAAGGCTTGGGGTTTTTCCCGGTCTTTTGGGGAACTTGAAACTGAGGTGGGACTTTCCAGAAACTGTTGCTAGTTTCGCTTTATCTGAGTACCATCTGTTCTTGGCCC

+

BBBBBFFFFFFFFFFFFFFFFFFFFFFFFFFFFFFFFFFFBFFFFFFFFFFFFFFFFFFFFFFFFFFFFFFFFFFFFFFFFFFFFFFFFFFFFFFFFFFFFFFBFFBFFFFFFFFFFFFFFFFFFF

@GWZHISEQ02:315:C9E6MANXX:5:1202:9891:19398

CTGATTGGTTAGTTTAAATAAGGCTTGGGGTTTTTCCCGGTCTTTTGGGGAACTTGAAACTGAGGTGGGACTTTCCAGAAACTGTTGCTAGTTTCGCTTTATCTGAGTACCATCTGTTCTTGGCCC

+

BBBBBFFFFFFFFFFFFFFFFFFFFFFFFFFFFFFFFFFFFFFFFFFFFFBFFFFFFFFFFF<FFFB/FBBFFFFFFFFFFFFFFFFFFFFFFFF<<FFF<FFF/<FFFFFFFFFFF<FFFFFFFF

@GWZHISEQ02:315:C9E6MANXX:5:1207:4579:21389

CTGGTTGGTTAGTTTAAATAAGGCTTGGGGTTTTTCCCGGTCTTTTGGGGAACTTGAAACTGAGGTGGGACTTTCCAGAAACTGTTGCTAGTTTCGCTTTATCTGAGTACCATCTGTTCTTGGCCC

+

BBBBBFFFFFFFFFFFFFFFFFFFFFFFFFFFFFFFFFFFFFFFFFFFFFFFFFFFFFFFFFFFFFFFFFFFFFFFFFFFFFFFFFFFFFFFFFFFFFFFFFFFFFFFFFFFFFFFFFFFFBFFFF

@GWZHISEQ02:315:C9E6MANXX:5:1216:7921:49564

CTGATTGGTTAGTTTAAATAAGGCTTGGGGTTTTTCCCGGTCTTTTGGGGAACTTGAAACTGAGGTGGGACTTTCCAGAAACTGTTGCTAGTTTCGCTTTATCTGAGTACCATCTGTTCTTGGCCC

+

BBBBBFFBFFFFFFFFFFFFFFFFFFFFFFFFFFFFFFFFFFFFFFFFFFBFFFFFFFFFFFFFFFFFFFFFFFFFFFFFFBFFFFFFFFFFFFFFFFFFFFFFBFFFFFFFFFFFFFFFFFFFFF

@GWZHISEQ02:315:C9E6MANXX:5:1304:14171:43004

CTGATTGGTTAGTTTAAATAAGGCTTGGGGTTTTTCCCGGTCTTTTGGGGAACTTGAAACTGAGGTGGGACTTTCCAGAAACTGTTGCTAGTTTCGCTTTATCTGAGTACCATCTGTTCTTGGCCC

+

//BBBFFFFFFFFFBFF<FFFFFFFFFFFF<FFFFFFFFFBFBBBFFFFF7FFFF/FFFBFFFFFFFFFF<FFFFFFFFFFF<FFFFFFFFFFF<<FFFFFF<FFFFFFFFFFFFFFFFFF/BFFF

@GWZHISEQ02:315:C9E6MANXX:5:1316:14730:71747

CTGATTGGTTAGTTTAAATAAGGCTTGGGGTTTTTCCCGGTCTTTTGGGTAACTTGAAACTGAGGGGGGACTTTCCAGAAACTGTTGCTAGTTTCGCTTTATCTGAGTACCATCTGTTATTGGCCC

+

BBBBBBF///BFFFFF</FF<FFF//7///<<////FFFFF/7BFFFFB//</BF/<BBBBF//F/<BB<F//FF/FFFFB///<B/<BF<FFFFFFFFFBBB<FFFFFF<<BFFFF///7/7B/<

@GWZHISEQ02:315:C9E6MANXX:5:2209:10172:54744

CTGATTGGTTAGTTTAAATAAGGCTTGGGGTTTTTCCCGGTCTTTTGGGGAACTTGAAACTGAGGTGGGACTTTCCAGAAACTGTTACTAGTTTCGCTTTATCTGAGTACCATCTGTTCTTGGCCC

+

BBBBBBF<BFFFFFFFFFFFFBFFFFFFF/<<7FFFFFFFBBFFFFFF<B/FF<BFFBBFFFFFFFFBFFF<BFBFFBFFFBFFFFFFBFBBBFFFF/BFFFFFFFFFFFFFFFFFFBFFFFFF<F

@GWZHISEQ02:315:C9E6MANXX:5:2305:4909:42083

CTGATTGGTTAGTTTAAATAAGGCTTGGGGTTTTTCCCGGTCTTTTGGGGAACTTGAAACTGAGGTGGGACTTTCCAGAAACTGTTGCTAGTTTCGCTTTATCTGAGTACCATCTGTTCTTGGCCC

+

BBBBBFFFFFFFFFFFFFFFFFFFFFFFFBFFFFFFFFFFFFFFFFFFFFFFFFFFFFFFFFFFFBFFFFFFFFFFFFFFFFFFFFFFFFFFFFFFFFFFFFFFFFFFFFFFFFFFFFFFFFFFFF

@GWZHISEQ02:315:C9E6MANXX:5:2110:3641:22577

GGCCAAGAACAGATGGTACTCAGATAAAGCGAAACTAGCAACAGTTTCTGGAAAGTCCCACCTCAGTTTCAAGTTCCCCAAAAGACCGGGAAAAACCCCAAGCCTTATTTAAACTAACCAATCAGC

+

BBBBBFFF/FBFFFFFFFFFFFFFFFFFBFFFFFFFFFFFFFFFBFFFFFFFFFFFFFFFBFBFFFFBFFFFFF<BFFFFFFF<FFFFFFBBFF/FFFFFFFF<FF/B/F<FFFF<FFFFFFFFFB

@GWZHISEQ02:315:C9E6MANXX:5:2205:7632:40416

GGCCAAGAACAGATGGTACTCAGATAAAGCGAAACTAGCAACAGTTTCTGGAAAGTCCCACCTCAGTTTCAAGTTCCCCAAAAGACCGGGAAAAACCCCAAGCCTTATTTAAACTAACCAATCAGC

+

BBBBBFFFFFFFFFFFFFFFFBFFFFFFFFFFFFBFFFFFFFFFFFFFFFFFFFFFFFFFF<FBFFFFFFFFFFFFFFFFFFFFFFFFFFFFFFFFFFFFFFFBFFFFFFFFFFFFFFFFFFFFFF

@GWZHISEQ02:315:C9E6MANXX:5:1110:2183:16515

GCTGATTGGTTAGTTTAAATAAGGCTTGGGGTTTTTCCCGGTCTTTTGGGGAACTTGAAACTGAGGTGGGACTTTCCAGAAACTGTTGCTAGTTTCGCTTTATCTGAGTACCATCTGTTCTTGGCC

+

BBBBBFFFFFFFFFFFFFFFFFFFFFFFFFFFFFFFFFFFFFFFFFFFFFFFFFFFFFFFFFFFBFFFFFFFFFFFFFFFFFFFFFFFFFFFFFFFFFFFFFFFFFFFFFFFFFFFFFFFFFFFFF

@GWZHISEQ02:315:C9E6MANXX:5:1306:12624:29117

GCTGATTGGTTAGTTTAAATAAGGCTTGGGGTTTTTCCCGGTCTTTTGGGGAACTTGAAACTGAGGTGGGACTTTCCAGAAACTGTTGCTAGTTTCGCTTTATCTGAGTACCATCTGTTCTTGGCC

+

BBBBBF<B</B/B<</BFFFFFFFFFFFFFB7BFFBFFF/7//7<<B/77<BBFFFF<F//////<FFBFFFFBFBB/<FFFBFBBFFFFBFFFFFFBFFFFF<<BFFFFFFFFF<BFFFFFBBFF

@GWZHISEQ02:315:C9E6MANXX:5:1312:7371:92587

GCTGATTGGTTAGTTTAAATAAGGCTTGGGGTTTTTCCCGGTCTTTTGGGGAACTTGAAACTGAGGTGGGACTTTCCAGAAACTGTTGCTAGTTTCGCTTTATCTGAGTACCATCTGTTCTTGGCC

+

BBBBBFFFFFFFFFFFFFFFFFFFFFFFFFF<FFFFFFFFFFFFFFFFF<<7FFFFFFFFFFFFFFFFFFFFFFFFFFFFFFFFFFFFFFFFFF/BBFFFFFFFFFFFFFFFFFFFBFFBFF/FFF

@GWZHISEQ02:315:C9E6MANXX:5:2205:7632:40416

GCTGATTGGTTAGTTTAAATAAGGCTTGGGGTTTTTCCCGGTCTTTTGGGGAACTTGAAACTGAGGTGGGACTTTCCAGAAACTGTTGCTAGTTTCGCTTTATCTGAGTACCATCTGTTCTTGGCC

+

//B<BBFFFFFFFFFFFFFFFFFFFFFFFFFBFFFFFFFFBFFFFFFFFB</FFFFFFFFFFFFBFFFFBFFFFFFFFFFFFFBBFFFFFFFFFFBFFFFFFBFF/F<FFFFFFFFFFFFFFFBFF

@GWZHISEQ02:315:C9E6MANXX:5:2309:12165:87666

GCTGATTGGTTAGTTTAAATAAGGCTTGGGGTTTTTCCCGGTCTTTTGGGGAACTTGAAACTGAGGTGGGACTTTCCAGAAACTGTTGCTAGTTTCGCTTTATCTGAGTACCATCTGTTCTTGGCC

+

//BBBFFFFFFFFFFFFFFFFFFFFFFFFFF<FFFFFFFFFBBBFFFFFFF<FFFFF<FFFFFFFBFFFFFFBF<<FFFFFBFF<FBF<BFBFFFFBFFFFFF/BF<F/7BB/FFFFFFBFFF<BF

@GWZHISEQ02:315:C9E6MANXX:5:1102:20741:49678

GCCAAGAACAGATGGTACTCAGATAAAGCGAAACTAGCAACAGTTTCTGGAAAGTCCCACCTCAGTTTCAAGTTCCCCAAAAGACCGGGAAAAACCCCAAGCCTTATTTAAACTAACCAATCAGCT

+

BBBBBFFFFFFFFFFFFFFFFFFFFFFFFFFFFFFFFFFFFFFFFFFFFFFFFFFFFFFFFFFFFFFFFFFFFFFFFFFFFFFFFFFFFFFFFFFBFFFFFFFFFFFFFFFFFFFFFFFFFFFFF/

@GWZHISEQ02:315:C9E6MANXX:5:1114:10457:78708

GCCAAGAACAGATGGTACTCAGATAAAGCGAAACTAGCAACAGTTTCTGGAAAGTCCCACCTCAGTTTCAAGTTCCCCAAAAGACCGGGAAAAACCCCAAGCCTTATTTAAACTAACCAATCAGCT

+

BBBBBFFFFFFFFFFFFFFFFFFFFFFFFFFFFFFFFFFFFFFFFFFFFFFFFFFFFFFFFFFFFFFFFFFFFFFFFFFFFFFFFFFFFFFFFFFFFFFFFFFFFFFFFFFFFFFFFFFFFFFFFF

@GWZHISEQ02:315:C9E6MANXX:5:2216:3919:33872

GCCAAGAACAGATGGTACTCAGATAAAGCGAAACTAGCAACAGTTTCTGGAAAGTCCCACCTCAGTTTCAAGTTCCCCAAAAGACCGGGAAAAACCCCAAGCCTTATTTAAACTAACCAATCAGCT

+

BBBBBFFFFFFFFFFFFFFFFFFFFFFFFF<FBFFFFFFFFFFFFFFFFFFFFFFFFFFFFFFFFFFFFFFFFFFFFFFFFFFFFFFFFFFFFFFFFFFFFFFFBFFFFFFBFFFFFFFFFFFFF/

@GWZHISEQ02:315:C9E6MANXX:5:2303:3385:56951

GCCAAGAACAGATGGTACTCAGATAAAGCGAAACTAGCAACAGTTTCTGGAAAGTCCCACCTCAGTTTCAAGTTCCCCAAAAGACCGGGAAAAACCCCAAGCCTTATTTAAACTAACCAATCAGCT

+

BBBBBFFFFFFFFFFFFFFFFFFFFFFFFFFFFFFFFFFFFFFFFFFFFFFFFFFFFFFFFFFFFFFFFFFFFFFFFFFFFFFFFFFFFFFFFFFFFFFFFFFFFFFFFFFFFFFFFFFFFFFFFF

@GWZHISEQ02:315:C9E6MANXX:5:1104:4984:22436

AGCTGATTGGTTAGTTTAAATAAGGCTTGGGGTTTTTCCCGGTCTTTTGGGGAACTTGAAACTGAGGTGGGACTTTCCAGAAACTGTTGCTAGTTTCGCTTTATCTGAGTACCATCTGTTCTTGGC

+

BBBBBFFFFFFFFFFFFFFFFFFFFFFFFFFFFFFFFFFFFFFFFFFFBBFFFFFFFFFFFFFFFFFFBFFFFFFFFFFFFFFFFFFFFFFFFFFFFFFFFFFFFFFFFFFFFFFFFFFFFFF<FB

@GWZHISEQ02:315:C9E6MANXX:5:1105:3431:43401

AGCTGATTGGTTAGTTTAAATAAGGCTTGGGGTTTTTCCCGGTCTTTTGGGGAACTTGAAACTGAGGTGGGACTTTCCAGAAACTGTTGCTAGTTTCGCTTTATCTGAGTACCATCTGTTCTTGGC

+

</B<BFFFFFFFFFFFFFFFFFFF<FFFFBFFFFFFFFFFFFFFFFFFFFBFBFFFFFFFFFF/BFBFFF<F<BFFBFFFFFFFF/FFFFFFFFFFFFFFFF/FFFF/FFFFFFF<B7FFFFF/<7

@GWZHISEQ02:315:C9E6MANXX:5:1109:8132:71496

AGCTGATTGGTTAGTTTAAATAAGGCTTGGGGTTTTTCCCGGTCTTTTGGGGAACTTGAAACTGAGGTGGGACTTTCCAGAAACTGTTGCTAGTTTCGCTTTATCTGAGTACCATCTGTTCTTGGC

+

BBBBBFFFFFFFFFFFFFFFFFFFFFFFFFFFFFFFFFFFFFFFFFFFBFFFFFFFFFFFFFFFFFFFFFFFFFFFFFFFFFFFFFFFFFFFFFFFFFFFFFFFFFFFFFFFFFFFFFFFFFFFFF

@GWZHISEQ02:315:C9E6MANXX:5:1114:10457:78708

AGCTGATTGGTTAGTTTAAATAAGGCTTGGGGTTTTTCCCGGTCTTTTGGGGAACTTGAAACTGAGGTGGGACTTTCCAGAAACTGTTGCTAGTTTCGCTTTATCTGAGTACCATCTGTTCTTGGC

+

BBBBBFFFFFFFFFFFFFFFFFFFFFFFFFFFFFFFFFFFFFFFFFFFFFFFFFFFFFFFFFFFFFFFFFFFFFFFFFFFFBFFFFFFFFFFFFFFFFFFFFFFFFFFFFFFFFFFFFFFFFFFFF

@GWZHISEQ02:315:C9E6MANXX:5:1115:5890:100226

AGCTGATTGGTTAGTTTAAATAAGGCTTGGGGTTTTTCCCGGTCTTTTGGGGAACTTGAAACTGAGGTGGGACTTTCCAGAAACTGTTGCTAGTTTCGCTTTATCTGAGTACCATCTGTTCTTGGC

+

BB<BBFFFFFFFFFFFFFFFFFFFFFFFFFFFFFFFFFFFFFBBBFFFF<FBBFFFFFFFFFFFFFFFFFFFFFFFFFFBFFFFFFFFFFFFFFFFFBFFFFFFFFFFFFFFFFFBFFF/FFFFFB

@GWZHISEQ02:315:C9E6MANXX:5:1204:12846:30380

GGCTGATTGGTTAGTTTAAATAAGGCTTGGGGTTTTTCCCGGTCTTTTGGGGAACTTGAAACTGAGGTGGGACTTTCCAGAAACTGTTGCTAGTTTCGCTTTATCTGAGTACCATCTGTTCTTGGC

+

BBBBBFFFFFFFFFFFFFFFFFFFFFFFFFFFFFFFFFFFFFFFFFFFFFFFFFFFFFFFFFFFFFFFFFFFFFFFFFFFFFFFFFFFFFFFFFFFFFFFFF<FFFFFFFFFFFFFFFFFFFFFF<

@GWZHISEQ02:315:C9E6MANXX:5:1204:18825:89239

AGCTGATTGGTTAGTTTAAATAAGGCTTGGGGTTTTTCCCGGTCTTTTGGGGAACTTGAAACTGAGGTGGGACTTTCCAGAAACTGTTGCTAGTTTCGCTTTATCTGAGTACCATCTGTTCTTGGC

+

BBBBBFFFFFFFFFFFFFFFFFFFFFFFFFFFFFFFFFFFBF<FFFFFFFFF<FFFFB<FFFFFFFFFFFFFFFFFFFFFFFFFFFFFFFFFFFFFFFFFFFFFFFFFFFFFFFFFFFFFFFFFFF

@GWZHISEQ02:315:C9E6MANXX:5:1207:11213:18432

AGCTGATTGGTTAGTTTAAATAAGGCTTGGGGTTTTTCCCGGTCTTTTGGGGAACTTGAAACTGAGGTGGGACTTTCCAGAAACTGTTGCTAGTTTCGCTTTATCTGAGTACCATCTGTTCTTGGC

+

BBBBBFFFFFFFFFFFFFFFFFFFFFFFFFFFFFFFFFFFFFFFBFFFFFFFFFFFFFFFFFFFFFFFFFFFFFFFFFBFFFBF/FBFBFFFFFFFFFFFFFFFFFFFBFFFFBFFFFFFFFF/FF

@GWZHISEQ02:315:C9E6MANXX:5:1209:14849:79029

AGCTGATTGGTTAGTTTAAATAAGGCTTGGGGTTTTTCCCGGTCTTTTGGGGAACTTGAAACTGAGGTGGGACTTTCCAGAAACTGTTGCTAGTTTCGCTTTATCTGAGTACCATCTGTTCTTGGC

+

BBBBBFFFFFFFFFFFFFFFFFFFFFFFFFFFFFFFFFFFFFFFFFFFFFFFFFFFFFFFFFFFFFFFFFFBFFFFFFFFFFFFFFFFFFFFFFFFFFFFFFFFBFFFFFFFFFFFFFFFFFFFFF

@GWZHISEQ02:315:C9E6MANXX:5:1313:17226:19993

GGCTGATTGGTTAGTTTAAATAAGGCTTGGGGTTTTTCCCGGTCTTTTGGGGAACTTGAAACTGAGGTGGGACTTTCCAGAAACTGTTGCTAGTTTCGCTTTATCTGAGTACCATCTGTTCTTGGC

+

BBBBBFFFFFFFFFFFFFFFFFFFFFFFFFFFFFFFFFFFFFFFFFFFFFFFBFFFFFFFFFFFFFFFFFFFFFFFFFFFFFFFFFFFBFFFFFFFFFFFFFFFFFFFFFFFFFFFFFFFFFFFBF

@GWZHISEQ02:315:C9E6MANXX:5:2110:18008:38538

GGCTGATTGGTTAGTTTAAATAAGGCTTGGGGTTTTTCCCGGTCTTTTGGGGAACTTGAAACTGAGGTGGGACTTTCCAGAAACTGTTGCTAGTTTCGCTTTATCTGAGTACCATCTGTTCTTGGC

+

/BBBBFFFFFFFFFFFFFFFFFFFFFFFFFFFFBFFF<FFFB///FFFFFFF<FF<FFFF<FFFFBFFFB//<F/F//<FB/</F/<7FBFBB/B/77B/FB<FFBF////7BBF7/B7/<FFFB/

@GWZHISEQ02:315:C9E6MANXX:5:2113:9606:29266

AGCTGATTGGTTAGTTTAAATAAGGCTTGGGGTTTTTCCCGGTCTTTTGGGGAACTTGAAACTGAGGTGGGACTTTCCAGAAACTGTTGCTAGTTTCGCTTTATCTGAGTACCATCTGTTCTTGGC

+

BBBBBFFFFFFFFFFFBFFFFFFFFFFFFFFFFFFFFFFBF7BFFFFFFFFFBBFFFFFFBFFFFFBFBFF/FBFFFFFFBF<BFF/FFB<BFBFB/F<FFFFFFFFFFBFBF<F<FFFFFFF</B

@GWZHISEQ02:315:C9E6MANXX:5:2202:7432:49833

AGCTGATTGGTTAGTTTAAATAAGGCTTGGGGTTTTTCCCGGTCTTTTGGGGAACTTGAAACTGAGGTGGGACTTTCCAGAAACTGTTGCTAGTTTCGCTTTATCTGAGTACCATCTGTTCTTGGC

+

BBBBBFFFFFFFFFFFFFFFFFFFFFFFFFFFFBFFFFFFFFFFBFFFF<FF/BFFF<<BFFFF/BBFFFBFBFFFFFFFFFFFF<FF/FF<FFFFFFF/BFBFF<B//B7BFBBFBB<//7//77

@GWZHISEQ02:315:C9E6MANXX:5:2208:19907:97882

AGCTGATTGGTTAGTTTAAATAAGGCTTGGGGTTTTTCCCGGTCTTTTGGGGAACTTGAAACTGAGGTGGGACTTTCCAGAAACTGTTGCTAGTTTCGCTTTATCTGAGTACCATCTGTTCTTGGC

+

BBBBBFFFFFFFFFFFFFFFFFFFFFFFFFFFFFFFFFFFFFFFFFFFFFFFFFFFFBFFFFFFFFFFFFFFFFFFFFFFFFFFFFFFFFFFFFFFFFFFBFFFFFFFFFFFFFFFFFFFFFFFBF

@GWZHISEQ02:315:C9E6MANXX:5:2209:14196:76446

AGCTGATTGGTTAGTTTAAATAAGGCTTGGGGTTTTTCCCGGTCTTTTGGGGAACTTGAAACTGAGGTGGGACTTTCCAGAAACTGTTGCTAGTTTCGCTTTATCTGAGTACCATCTGTTCTTGGC

+

BBBBBFFFFFFFFFFFFFFFFFFFFFFFFFFFFFFFFFFFFF/<FFFFFFFFFFFFFFFFFFFFFFFFFFFFFFFFFFFFFFFFFFFFFFFFFFFFFFBFFFFFFFFFFBFFFFFFBFFFFFFFF<

@GWZHISEQ02:315:C9E6MANXX:5:2214:19993:83030

AGCTGATTGGTTAGTTTAAATAAGGCTTGGGGTTTTTCCCGGTCTTTTGGGGAACTTGAAACTGAGGTGGGACTTTCCAGAAACTGTTGCTAGTTTCGCTTTATCTGAGTACCATCTGTTCTTGGC

+

BBBBBFFFFFFFFFFFFFFFFFFFFFFFFFFFFFFFFFFFFFFFFFFFFFFFFFFFFFFFFFFFFFFFFFFFFFFFFFFFBFFFBFFFFFFFFFFFFFFFFFFFFFFFFBFFB<FFFFFFFFFFFF

@GWZHISEQ02:315:C9E6MANXX:5:2314:17944:21322

AGCTGATTGGTTAGTTTAAATAAGGCTTGGGGTTTTTCCCGGTCTTTTGGGGAACTTGAAACTGAGGTGGGACTTTCCAGAAACTGTTGCTAGTTTCGCTTTATCTGAGTACCATCTGTTCTTGGC

+

<BBBBFFFFFFFFFFFFFFFFFFFFFFFFFFFFFFFFFFFFFFFFFFFFFFFFFFFFFFFFFFFFFFFBFFBFFFFFFFFFFFFFFFFFFFFFFFFFFFFFFFFFFBFFFFFFFFFFFFFFFF<FF

@GWZHISEQ02:315:C9E6MANXX:5:2314:1972:62206

AGCTGATTGGTTAGTTTAAATAAGGCTTGGGGTTTTTCCCGGTCTTTTGGGGAACTTGAAACTGAGGTGGGACTTTCCAGAAACTGTTGCTAGTTTCGCTTTATCTGAGTACCATCTGTTCTTGGC

+

BBBBBFFFFFFFFFFFFFFFFFFFFFFFFFFFBFFFFFFFFFBFFFFFFFFFBFFFFFBFFFFFFFFFFFFFFFFFFFFFFFFFFFFFFFFBFFFFFFFFFFFFFFFFFFFFFFF<FFBFFFFFBB

@GWZHISEQ02:315:C9E6MANXX:5:1106:15752:49527

CCAAGAACAGATGGTACTCAGATAAAGCGAAACTAGCAACAGTTTCTGGAAAGTCCCACCTCAGTTTCAAGTTCCCCAAAAGACCGGGAAAAACCCCAAGCCTTATTTAAACTAACCAATCAGCTC

+

BBBBBFFFFFFFFFFFFFFFFFFFFFFFFFFFFFFFFFFFFFFFFFFFFFFFFFFFFFFFFFFFFFFFFFFFFFFFFFFFFFFFFFFFFFFFFFFFFFFFFFFFFFFFFFFFFFFFFFFFFFFFFF

@GWZHISEQ02:315:C9E6MANXX:5:1116:10222:54363

CCAAGAACAGATGGTACTCAGATAAAGCGAAACTAGCAACAGTTTCTGGAAAGTCCCACCTCAGTTTCAAGTTCCCCAAAAGACCGGGAAAAACCCCAAGCCTTATTTAAACTAACCAATCAGCTC

+

BBBBBFFFFFFFFFFFFFFFFFFFFFFFFFFFFFFFFFFFFFFFFFFFFFFFFFFFFFFFFFFFFFFFFFFFFFFFFFFFFFFFFFFFFFFFBFFFFFFFFFFFFFFFFFFFFFFFFFFFFFFFFF

@GWZHISEQ02:315:C9E6MANXX:5:1308:6293:8839

CCAAGAACAGATGGTACTCAGATAAAGCGAAACTAGCAACAGTTTCTGGAAAGTCCCACCTCAGTTTCAAGTTCCCCAAAAGACCGGGAAAAACCCCAAGCCTTATTTAAACTAACCAATCAGCTC

+

BBBBBFFFFFFFFFFFFFFFFFFFFFFFFFFFFFFFFFFFFFFFFFFFFFFFFFFFFFFFFFFFFFFFFFFFFFFFFFFFFFFFFFFFFFFFFFFFFFFFFFFFFFFFFFFFFFFFFFFFFFFFFF

@GWZHISEQ02:315:C9E6MANXX:5:2102:2452:26346

CCAAGAACAGATGGTACTCAGATAAAGCGAAACTAGCAACAGTTTCTGGAAAGTCCCACCTCAGTTTCAAGTTCCCCAAAAGACCGGGAAAAACCCCAAGCCTTATTTAAACTAACCAATCAGCTC

+

B//<BFBFFBBFFFFFBBFFFFFFFFFFBB<<FFFF</BFFF<<BBFBFFBFFF/FF/F/FFF<<BFFFFFF<FFFBBFB<FBFFFFBFF/<FFBFBFBFFFFF<FFBFF/FF/<BB7FFFFFFFB

@GWZHISEQ02:315:C9E6MANXX:5:2115:14154:34065

CCAAGAACAGATGGTACTCAGATAAAGCGAAACTAGCAACAGTTTCTGGAAAGTCCCACCTCAGTTTCAAGTTCCCCAAAAGACCGGGAAAAACCCCAAGCCTTATTTAAACTAACCAATCAGCTC

+

BBBBBFFFFFFFFFFFFFFFFFFFFFFFFFFFFFFFFFFFFFFFFFFFFFFFFFFFFFFFFFFFFFFFFFFFBFFFFFFFFFFFFFFFFFFFFFFFFFFFFFFFFFFFFFFFFFFFFFFFFFFFFF

@GWZHISEQ02:315:C9E6MANXX:5:1116:20058:27010

GAGCTGATTGGTTAGTTTAAATAAGGCTTGGGGTTTTTCCCGGTCTTTTGGGGAACTTGAAACTGAGGTGGGACTTTCCAGAAACTGTTGCTAGTTTCTCTTTATCTGAGTACCATTTGTTCTTGG

+

BBBBBFFFFFFFFFFFFFFFFFFFFFFFFFFFFBFFFFFFFFFFB7/FFF/FFBF<BB<BFFFF/FFFFF//F/FFFFFFFFB/FF/<7F//<<<F/F//////7<////</<BF///<///7<<<

@GWZHISEQ02:315:C9E6MANXX:5:1104:18038:59881

CAAGAACAGATGGTACTCAGATAAAGCGAAACTAGCAACAGTTTCTGGAAAGTCCCACCTCAGTTTCAAGTTCCCCAAAAGACCGGGAAAAACCCCAAGCCTTATCTAAACTAACCAATCAGCTCC

+

BB<BBFFFFF/<BFFF/FFFFBB///F<//BB//<F/<FB/BF/FBFFFFFFFFFFFFFBFBF<//F/<<</FFFFF<FBB/FFFFF/<7FFFFFF/7/BF//////</<BBFBFFFF/FB/B/B/

@GWZHISEQ02:315:C9E6MANXX:5:1104:21007:64013

CAAGAACAGATGGTACTCAGATAAAGCGAAACTAGCAACAGTTTCTGGAAAGTCCCACCTCAGTTTCAAGTTCCCCAAAAGACCGGGAAAAACCCCAAGCCTTATTTAAACTAACCAATCAGCTCG

+

BBBBBFFF/FFB/<<<B/FFFBFBFFFFFFFFFFFFFFFFFFFFFFFFFFFBFFFFF<BFFFFFFFFFB/<FFFF<FFFFFFFFFFFBFFFFFFFFFFFBFFFFF<FBBFBFFFFBFFF/BF///7

@GWZHISEQ02:315:C9E6MANXX:5:1108:6501:59549

CAAGAACAGATGGTACTCAGATAAAGCGAAACTAGCAACAGTTTCTGGAAAGTCCCACCTCAGTTTCAAGTTCCCCAAAAGACCGGGAAAAACCCCAAGCCTTATTTAAACTAACCAATCAGCTCG

+

BBBBBFFFFFFFFFFFFFFFFFFFFFFFFFFFFFFFFFFFFFFFFFFFFFFFFFFFFFFFFFFFFFFFFFFFFFFFFFFFFFFFFFFFFFFFFFFFFFFFFFFFFFFFFFFFFFFFFFFFFFFFFB

@GWZHISEQ02:315:C9E6MANXX:5:1215:10498:98092

CAAGAACAGATGGTACTCAGATAAAGCGAAACTAGCAACAGTTTCTGGAAAGTCCCACCTCAGTTTCAAGTTCCCCAAAAGACCGGGAAAAACCCCAAGCCTTATTTAAACTAACCAATCAGCTCG

+

BBBBBFFFFFFFFFFFFFFFFFFFFFFFFFFFFFFFFFFFFFFFFFFFFFFFFFFFFFFFFFFFFFFFFFFFFFFFFFFFFFFFFFFFFFFFFFFFFFFFFFFFFFFFFFFFFFFFFFFFFFFBFB

@GWZHISEQ02:315:C9E6MANXX:5:1307:6212:34837

CAAGAACAGATGGTACTCAGATAAAGCGAAACTAGCAACAGTTTCTGGAAAGTCCCACCTCAGTTTCAAGTTCCCCAAAAGACCGGGAAAAACCCCAAGCCTTATTTAAACTAACCAATCAGCTCG

+

BBBBBFFFF/B/BFFFFFFFFFFFFFFFFFFFFFFFBFFFFFFFFFFFFFFFFFFBFFFFFFFFFFFFFFFFFFFFFFFFFFFFFFFFFFFFFBFFFFFFFFFFFFFFFFFFFFFFFFBFBFFFF/

@GWZHISEQ02:315:C9E6MANXX:5:1312:14223:76585

CAAGAACAGATGGTACTCAGATAAAGCGAAACTAGCAACAGTTTCTGGAAAGTCCCACCTCAGTTTCAAGTTCCCCAAAAGACCGGGAAAAACCCCAAGCCTTATTTAAACTAACCAATCAGCTCG

+

BBBBBFFFFFFFFFFFFFFFFFFFFFFFFFFFFFFFFFFFFFFFFFFFFFFFFFFFFFFFFFFFFFFFFFFFFFFFFFFFFFFFFFFFFFFFFFFFFFFFFFFFFFFFFFFFFFFFFFFFFFFFFF

@GWZHISEQ02:315:C9E6MANXX:5:2209:20653:90527

CAAGAACAGATGGTACTCAGATAAAGCGAAACTAGCAACAGTTTCTGGAAAGTCCCACCTCAGTTTCAAGTTCCCCAAAAGACCGGGAAAAACCCCAAGCCTTATTTAAACAGATCGGAAGAGCGT

+

BBBBBFFFFFFFFFFFFFFFFFFFFFFFFFFFFFFFFFFFFFFFFFFFFFFFFFFFFFFFFFFFFFFFFFFFFFFFFFFFFFFFFFFFFFFFFFFFFBFFFFFFFFFFFFFFFFFFFFFFFFFFF7

@GWZHISEQ02:315:C9E6MANXX:5:2214:13799:82981

CAAGAACAGATGGTACTCAGATAAAGCGAAACTAGCAACAGTTTCTGGAAAGTCCCACCTCAGTTTCAAGTTCCCCAAAAGACCGGGAAAAACCCCAAGCCTTATTTAAACTAACCAATCAGCTCG

+

BBBBBFFFFFFFFFFFFFFFFFFFFFFFFFFFFFFFFFFFFFFFFFFFFFFFFFFFFFFFFFFFFFBFFFFFFFFFFFFFFFFFFFFFFFFFFBFFFFFFFFFFFFFFFFFFFFFFFFFFFFFFFB

@GWZHISEQ02:315:C9E6MANXX:5:1107:16531:31165

CGAGCTGATTGGTTAGTTTAAATAAGGCTTGGGGTTTTTCCCGGTCTTTTGGGGAACTTGAAACTGAGGTGGGACTTTCCAGAAACTGTTGCTAGTTTCGCTTTATCTGAGTACCATCTGTTCTTG

+

BBBBBFFFFFFFFFFFFFFFFFFFFFFFFFFFFFBFFFFFFFFFFFFFFF<FFFFFFFFBBFFFFFFFFFFFFFFFFFFFFFFFFFFFFFFFFFFFFFBFFFFFFFFFFFFFFFFFFFFFFFFFFF

@GWZHISEQ02:315:C9E6MANXX:5:1202:18747:64379

GGAGCTGATTGGTTAGTTTAAATAAGGCTTGGGGTTTTTCCCGGTCTTTTGGGGAACTTGAAACTGAGGTGGGACTTTCCAGAAAATGTTGCTAGTTTCGCTTTATCTGAGTACCATCTGTTCTTG

+

/<BB</BBFFBF/FFFF<F<F<FF/</B//7F/<<FFFFFFF//B/BBF///FFBFFFFFFFFB///<<<F<F/<F/<<<FFFFB////F//7//<7BF<//<F/F</F//7FFFB/7///</<F/

@GWZHISEQ02:315:C9E6MANXX:5:2203:4944:21266

CGAGCTGATTGGTTAGTTTAAATAAGGCTTGGGGTTTTTCCCGGTCTTTTGGGGAACTTGAAACTGAGGTGGGACTTTCCAGAAACTGTTGCTAGTTTCGCTTTATCTGAGTACCATCTGTTCTTG

+

BBBBBFFFFFFFFFFFFFFFFFFFFFFFFFFFFFBFFFFFFFFF/<FFFFFFFF//FFFFFFFFFF<FFF<FF<FFFFFF<FFFF<7BFBFFFFFFFF<BF<7BFFFF</FFFFFFFFFFFFFFFF

@GWZHISEQ02:315:C9E6MANXX:5:2209:20653:90527

GTTTAAATAAGGCTTGGGGTTTTTCCCGGTCTTTTGGGGAACTTGAAACTGAGGTGGGACTTTCCAGAAACTGTTGCTAGTTTCGCTTTATCTGAGTACCATCTGTTCTTGAGATCGGAAGAGCAC

+

BBBBBFFFFFFFFFFFFFFFFFFFFFFFFFFFFFFFFFFFFFFFFFFFFFFFFFFFFFFFFFFFFFFFFFFFFFFFFFFFFFFFFFFFFFFFFFFFFFFFFFFBF<FFFFFFFFFFFFFFFFFFFF

@GWZHISEQ02:315:C9E6MANXX:5:1301:1712:70178

AGAACAGATGGTACTCAGATAAAGCGAAACTAGCAACAGTTTCTGGAAAGTCCCACCTCAGTTTCAAGTTCCCCAAAAGACCGGGAAAAACCCCAAGCCTTATTTAAACTAACCAATCAGCTCGCT

+

BBBBBFFFFFFFFFFFFFFFFFFFFFFFFFFFFFFFFFFFFFFFFFFFFFFFFFFFFFFFFFFFFFFFFFFFFFFFFFFFFFFFFFFFFFFFFFFFFFFFFFFFFFFFFFFFFFFFFFFFFFFFFF

@GWZHISEQ02:315:C9E6MANXX:5:1306:4228:48030

AGAACAGATGGTACTCAGATAAAGCGAAACTAGCAACAGGTTCTGGAAAGTCCCACCTCAGGTTCAAGTTCCCCAAAAGACCGGGAAAAACCCCAAGCCTTATTTAAACTAACCAATCAGCTCGCT

+

//B//F///F//B/<<</</BF//<7F7F////<<//B</////<////<F/F/</</<F//<<</FF//////<FF</B</FF<</<///<//<7//7/<FF//<F/<</B/7</BFB/////7/

@GWZHISEQ02:315:C9E6MANXX:5:2310:2313:3239

AGAACAGATGGTACTCAGATAAAGCGAAACTAGCAACAGTTTCTGGAAAGTCCCACCTCAGTTTCAAGTTCCCCAAAAGACCGGGAAAAACCCCAAGCCTTATTTAAACTAACCAATCAGCTCGCT

+

BBBBBFFFFFFFFFFFFFFFFFFFFFFFFFFFFFFFFFFFFFFFFFFFFFFFFFFFFFFFFFFFFFFFFFFFFFFFFFFFFFFFFFFFFFFFFFFFFFFFFFFBFFFFFFFFFFFFFFFFFFFFFF

@GWZHISEQ02:315:C9E6MANXX:5:2311:15778:12796

AGAACAGATGGTACTCAGATAAAGCGAAACTAGCAACAGTTTCTGGAAAGTCCCACCTCAGTTTCAAGTTCCCCAAAAGACCGGGAAAAACCCCAAGCCTTATTTAAACTAACCAATCAGCTCGCT

+

BBBBBFFFFFFFFFFFFBFFFFFFFFFFFFFFFFFFFFFBFFFFFFFFFFFFFFFFFFFFFFFFFFFFFFFFFFFFFFFFFFFFFFFFFFFFFFFFFFFFFFFFFFFFFFFFFFFFFFFFFFFFFF

@GWZHISEQ02:315:C9E6MANXX:5:2211:20272:13631

GGCGAGCTGATTGGTTAGTTTAAATAAGGCTTGGGGTTTTTCCCGGTCTTTTGGGGAACTTGAAACTGAGGTGGGACTTTCCAGAAACTGTTGCTAGTTTCGCTTTATCTGAGTACCATCTGTTCT

+

BBBB<<F<FBFFFBFFFFFFF/B/F/FFFFFBFFFF7BFFFBFFBB/7<FFFFFBBBFFFFFF/FFFFFBFFBFFFFF<FFFFFFFFBFFFFFFFFFFFFF/B7FF<FFFBFFB7BFBFFFFBB7F

@GWZHISEQ02:315:C9E6MANXX:5:1207:17708:94205

GAACAGATGGTACTCAGATAAAGCGAAACTAGCAACAGTTTCTGGAAAGTCCCACCTCAGTTTCAAGTTCCCCAAAAGACCGGGAAAAACCCCAAGCCTTATTTAAACTAACCAATCAGCTCGCTT

+

BBBBBFFFFFFFFFFFFFFFFFFFFFFFFFFFFFFFFFFFFFFFFFFFFFFFFFFFFFFFFFFFFFFFFFFFFFFFFFFFFFFFFFFFFFFFFFFFFFFFFFFFFFFFFFFFFFFFFFFFFFFFFF

@GWZHISEQ02:315:C9E6MANXX:5:2312:20829:13328

GAACAGATGGTACTCAGATAAAGCGAAACTAGCAACAGTTTCTGGAAAGTCCCACCTCAGTTTCAAGTTCCCCAAAAGACCGGGAAAAACCCCAAGCCTTATTTAAACTAACCAATCAGCTCGCTT

+

BBBBBFFFFFFFFFFFFFFFFFFFFFFFFFFFFFFFFFFFFFFFFFFFFFFFFFFFFFFFFFFFFFFFFFFFFFFFFFFFFFFFFFFFFBFFFFFFFFFFFFFFFFFFFFFFFFFFFFFFFFFFFF

@GWZHISEQ02:315:C9E6MANXX:5:2315:9328:46536

GAACAGATGGTACTCAGATAAAGCGAAACTAGCAACAGTTTCTGGAAAGTCCCACCTCAGTTTCAAGTTCCCCAAAAGACCGGGAAAAACCCCAAGCCTTATTTAAACTAACCAATCAGCTCGCTT

+

BBBBBFFFFFFFFFFFFFFFFFFFFFFFFFFFFFFFFFFFFFFFFFFFFFFFFFFFFFFFFFFFFFFFFFFFFFFFFFFFFFFFFFFFFFFFFFFFFFFFFFFFFFFFFFFFFFFFFFFFFFFFFF

@GWZHISEQ02:315:C9E6MANXX:5:1213:4227:19669

AACAGATGGTACTCAGATAAAGCGAAACTAGCAACAGTTTCTGGAAAGTCCCACCTCAGTTTCAAGTTCCCCAAAAGACCGGGAAAAACCCCAAGCCTTATTTAAACTAACCAATCAGCTCGCTTC

+

BBBBBFFFFF<FFFFFFFFFFFFFFFFFFFFFFFFFFFFFFFFFFFFFFFFFFFFFFFFFFFFFFF<FFBFFFBBBFFBFFFFF<FFFFBFFFFFFFFFFFFBFFFFFFFFB<FFFFFFBFFFFFF

@GWZHISEQ02:315:C9E6MANXX:5:2114:12293:80632

AACAGATGGTACTCAGATAAAGCGAAACTAGCAACAGTTTCTGGAAAGTCCCACCTCAGTTTCAAGTTCCCCAAAAGACCGGGAAAAACCCCAAGCCTTATTTAAACTAACCAATCAGCTCGCTTC

+

BBBBBFFFFFFFFFFFFFFFFFFFFFFFFFFFFFFFFFFFFFFFFFFFFFFFFFFFFFFFFFFFFFFFFFFFFFFFFFFFFFFFFFFFFFFFFFFFFFFFFFFFFFFFFFFFFFFFFFFFFFFFFF

@GWZHISEQ02:315:C9E6MANXX:5:1114:7718:77995

CAGATGGTACTCAGATAAAGCGAAACTAGCAACAGTTTCTGGAAAGTCCCACCTCAGTTTCAAGTTCCCCAAAAGACCGGGAAAAACCCCAAGCCTTATTTAAACTAACCAATCAGCTCGCTTCTC

+

BBBBBFFFFFFFFFFFFFFFFFFFFFFFFFFFFFFFFFFFFFFFFFFFFFFFFFFFFFFFFFFFFFFFFFF<FFFFFFFFFFFFFFFFFFFFFFFFFFFFFFFFFFFFFFFFFFFFFFFFFFFFFF

@GWZHISEQ02:315:C9E6MANXX:5:2208:20446:97541

CAGATGGTACTCAGATAAAGCGAAACTAGCAACAGTTTCTGGAAAGTCCCACCTCAGTTTCAAGTTCCCCAAAAGACCGGGAAAAACCCCAAGCCTTATTTAAACTAACCAATCAGCTCGCTTCTC

+

BBBBBFFFFFFFFFFFFFFFFFFFFFFFFFFFFFFFFFFFFFFFFFFFFFFFFFFFFFFFFFFFFFFFFFFFFFFFFFFFFFFFFFFFFFFFFFFFFFFFFFFFFFFFFFFFFFFFFFFFFFFFFF

@GWZHISEQ02:315:C9E6MANXX:5:2302:10115:2638

CAGATGGTACTCAGATAAAGCGAAACTAGCAACAGTTTCTGGAAAGTCCCACCTCAGTTTCAAGTTCCCCAAAAGACCGGGAAAAACCCCAAGCCTTATTTAAACTAACCAATCAGCTCGCTTCTC

+

BBBBBFFFFFFFFFFFFFFFFFFFFFFFFFFF<FFFFFFFFFFFFFFFFFFFFFFFFFFFFFFFFFFFFFFFFFFFFFFFFFFFFFFFFFFFFFFFFFFFFFFFFFFFFFFFFFFFFFFFFFFFFF

@GWZHISEQ02:315:C9E6MANXX:5:1204:2721:88177

AGATGGTACTCAGATAAAGCGAAACTAGCAACAGTTTCTGGAAAGTCCCACCTCAGTTTCAAGTTCCCCAAAAGACCGGGAAAAACCCCAAGCCTTATTTAAACTAACCAATCAGCTCGCTTCTCG

+

BBBBBFFFFFFFFFFFFFFFFFFFFFFFFFFFFFFFFFFFFFFFFFFFFFFFFFFFFFFFFFFFFFFFFFFFFFFFFFFFFFFFFFFFFFFFFFFFFFFFFFFFFFFFFFFFFFFFFFFFFFFFFF

@GWZHISEQ02:315:C9E6MANXX:5:2312:10870:46087

CGAGAAGCGAGCTGATTGGTTAGTTTAAATAAGGCTTGGGGTTTTTCCCGGTCTTTTGGGGAACTTGAAACTGAGGTGGGACTTTCCAGAAACTGTTGCTAGTTTCGCTTTATCTGAGTACCATCT

+

BBBBBFFFFFFFFF/FFFFFFFFFFFFFFFFFFFFFFFFFF<FFFFFFFFFFFFFFFFFFFFFFFFF</FFFFBFFFFFF/FFFFBFBFFFFFFFFFFFFFFFBFFFFFFFFFFF7FFFBFFFFFF

@GWZHISEQ02:315:C9E6MANXX:5:1203:2846:30002

GATGGTACTCAGATAAAGCGAAACTAGCAACAGTTTCTGGAAAGTCCCACCTCAGTTTCAAGTTCCCCAAAAGACCGGGAAAAACCCCAAGCCTTATTTAAACTAACCAATCAGCTCGCTTCTCGC

+

BBBBBFFFFFFFFFFFFFFFFFFFFFFFFFFFFFFFFFFFFFFFFFFFFFFFFFFFFFFFFFFFFFFFFFFFFFFFFFFFFFFFFFFFFFFFFFFFFFFFFFFFFFFFFFFFFFFFFFFFFFFFFF

@GWZHISEQ02:315:C9E6MANXX:5:1104:3985:31060

ATGGTACTCAGATAAAGCGAAACTAGCAACAGTTTCTGGAAAGTCCCACCTCAGTTTCAAGTTCCCCAAAAGACCGGGAAAAACCCCAAGCCTTATTTAAACTAACAGATCGGAAGAGCGTCGTGT

+

B<B<<FFFFFFFFBF<<FFFFFBBFFB/FF/FB/BFBF<<FFFFFFF<FFFFFF<FFFFF<<FFBFBF<FFFFFFFBFFFFFFF<FFFFFF/FFBFFFFFFFFFFBFFFFFFFFFFFFFFBBF7B7

@GWZHISEQ02:315:C9E6MANXX:5:1109:11293:80911

ATGGTACTCAGATAAAGCGAAACTAGCAACAGTTTCTGGAAAGTCCCACCTCAGTTTCAAGTTCCCCAAAAGACCGGGAAAAACCCCAAGCCTTATTTAAACTAACCAATCAGCTCGCTTCTCGCT

+

BBBBBFFFFFFFFFFFBFFFBFFFFFFFFFFFFFFFFFFFFFFFFFFFFFFFFFFFFFFBFFBFFFFFFFFFFFFFFFBF/FFFFFFFFFFFFFFFFFFFFFFFFFFFFFFFF<F7FFFFFFFFFF

@GWZHISEQ02:315:C9E6MANXX:5:1104:3985:31060

GTTAGTTTAAATAAGGCTTGGGGTTTTTCCCGGTCTTTTGGGGAACTTGAAACTGAGGTGGGACTTTCCAGAAACTGTTGCTAGTTTCGCTTTATCTGAGTACCATAGATCGGAAGAGCACACGTC

+

BBBBB<FFFFF<FFFFFFFFFFFFFFFFFFFFFBFBFFFFFFFFFFFFFFFFF</FFFFFFFFFFFFFF<FFFF/FFFFFFFFFFFFFFFFFFFFFFFFFFFFF<FFFFFFFFFFFFFFFBFBB7B

@GWZHISEQ02:315:C9E6MANXX:5:2216:2470:44291

AGCGAGAAGCGAGCTGATTGGTTAGTTTAGATAAGGCTTGGGGTTTTTCCCGGTCTTTTGGGGGACTTGAAACTGAGGTGGGACTTTCCAGAAACTGTTGCTAGTTTCGCTTTATCTGAGTACCAT

+

BBBBBFFFFFFFF<FFBFFFFFFFFFFFFFFFFFFFFFFFFFFFFFFFFFFFBFFFFFFFFFFFFFFFFB<BFFF/BFFFFFBB7FFFFFFFF/FFFFFFBFFFFFFFBFFFFFFFFF<FFFFFBF

@GWZHISEQ02:315:C9E6MANXX:5:2308:15369:99667

TGGTACTCAGATAAAGCGAAACTAGCAACAGTTTCTGGAAAGTCCCACCTCAGTTTCAAGTTCCCCAAAAGACCGGGAAAAACCCCAAGCCTTATTTAAACTAACCAATCAGCTCGCTTCTCGCTT

+

BBBBBFFFFFFFFFFFFFFFFFFFFFFFFFFFFFFFFFFFFFFFFFFFFFFFFFFFFFFFFFFFFFFFFFFFFFFFFFFFFFFFFFFFFFFFFFFFFFFFFFFFFFFFFFFFFFFFFFFFFFFFFF

@GWZHISEQ02:315:C9E6MANXX:5:2303:2281:12173

AAGCGAGAAGCGAGCTGATTGGTTAGTTTAAATAAGGCTTGGGGTTTTTCCCGGTCTTTTGGGGAACTTGAAACTGAGGTGGGACTTTCCAGAAACTGTTGCTAGTTTCGCTTTATCTGAGTACCA

+

BBBBBFFFFFF<FFFFFFFFFFFFFFFFFFFFFFFFFFFFFBFFFFFFFFFFFB7FFFFFFFFF<FFFFFFFFB/FFFFFFFFBFFFFFFBF/FF<<FFFFFFFFFFFFFFFFF7FBFF</7/BFF

@GWZHISEQ02:315:C9E6MANXX:5:1114:6108:90065

AGAAGCGAGAAGCGAGCTGATTGGTTAGTTTAAATAAGGCTTGGGGTTTTTCCCGGTCTTTTGGGGAACTTGAAACTGAGGTGGGACTTTCCAGAAACTGTTGCTAGTTTCGCTTTATCTGAGTAC

+

B/BBBFFFFFFFFFFFBFFBFFFFFFFFFFFFFFFFFFFFFFFFFF/FFFFFFFFBBFFFFFFFFFFBFFFFFFFFFFFFFFFFFBFFFFFFFFFFFFFFFFFFFFFBFFFFFFFFFFFFFFFFF7

@GWZHISEQ02:315:C9E6MANXX:5:1303:2483:57711

AGAAGCGAGAAGCGAGCTGATTGGTTAGTTTAAATAAGGCTTGGGGTTTTTCCCGGTCTTTTGGGGAACTTGAAACTGAGGTGGGACTTTCCAGAAACTGTTGCTAGTTTCGCTTTATCTGAGTAC

+

BBBBBFFFFFFFFFFFFFFFFFFFFFFFFFFFFFFFFFFFFFFFFFFFFFFFFFFFBFFFFFFFFFFFFFFF<FFFFFFFF<FFF<<FFFFFFFFFFFFFFFFFBFFFFFFFFFFFFFFFFFFFFB

@GWZHISEQ02:315:C9E6MANXX:5:2205:8679:57794

AGAAGCGAGAAGCGAGCTGATTGGTTAGTTTAAATAAGGCTTGGGGTTTTTCCCGGTCTTTTGGGGAACTTGAAACTGAGGTGGGACTTTCCAGAAACTGTTGCTAGTTTCGCTTTATCTGAGTAC

+

BBBBBF<BFFFFFFFFB/BFFFFFFFFFFFFFFFFFF<FFFFF<FFFFFFFFFFFB/FFFBFFFBFBFFFFFFFF<F/FFFFF/FBFFFFFFF<BFFFFFFFFFF<FFFFFFFFFFBBBF<FF/7B

@GWZHISEQ02:315:C9E6MANXX:5:2308:18771:45161

AGAAGCGAGAAGCGAGCTGATTGGTTAGTTTAAATAAGGCTTGGGGTTTTTCCCGGTCTTTTGGGGAACTTGAAACTGAGGTGGGACTTTCCAGAAACTGTTGCTAGTTTCGCTTTATCTGAGTAC

+

BBBBBFFFFFFFFFFFFFFFFFFFFFFFFFFFFFFFFFFFFFFFFFFFFFFFFFFFFFFFFFFFFFFFFFFFFFFFFFFFFFFFFFFFFFFFFFFFFFFFFFFFFFFFFFFFFFFFFFFFFFFFFF

@GWZHISEQ02:315:C9E6MANXX:5:2206:14787:40608

CAGAAGCGAGAAGCGAGCTGATTGGTTAGTTTAAATAAGGCTTGGGGTTTTTCCCGGTCTTTTGGGGAACTTGAAACTGAGGTGGGACTTTCCAGAAACTGTTGCTAGTTTCGCTTTATCTGAGTA

+

BBBBBFFFFFFFFFFFFFFFFFFFFFFFFFFFFFFFFFFFFFFFFFFFFFFFFFFFFBBFFFFFFFF<FFFFFFFFFFFFFFFFFFFFFFFFFFFFFFFFFFFFFFFFFFFFFFFFFFFFFFFFFF

@GWZHISEQ02:315:C9E6MANXX:5:1202:3730:43740

ACTCAGATAAAGCGAAACTAGCAACAGTTTCTGGAAAGTCCCACCTCAGTTTCAAGTTCCCCAAAAGACCGGGAAAAACCCCAAGCCTTATTTAAACTAACCAATCAGCTCGCTTCTCGCTTCTGT

+

BBBBBFFBFFFFFFFFFFFFFFFFFFFFFFFFFFFFFFFFFFFFFFFFFBBBBFFFFFFFFFFFFFF<BFFFFBFFFFF<<BFBFFFB<F</B<FFFF/BBFFFBFBFFFBFFFBFFFFBBFF<BB

@GWZHISEQ02:315:C9E6MANXX:5:2216:12109:16613

ACTCAGATAAAGCGAAACTAGCAACAGTTTCTGGAAAGTCCCACCTCAGTTTCAAGTTCCCCAAAAGACCGGGAAAAACCCCAAGCCTTATTTAAACTAACCAATCAGCTCGCTTCTCGCTTCTGT

+

BBBBBFBFFFFFFFF/BFF<BFFFFBFBFFFFFFFFFFFFBFFFBFBF/FFB<F<FBBFFFFFFBFFBBBFBFFF<FBFFFFFFFFFFFFFFFF/7F<F<FBFFFFFF<FFFFFF<FF//BFFFFF

@GWZHISEQ02:315:C9E6MANXX:5:1106:15752:49527

ACAGAAGCGAGAAGCGAGCTGATTGGTTAGTTTAAATAAGGCTTGGGGTTTTTCCCGGTCTTTTGGGGAACTTGAAACTGAGGTGGGACTTTCCAGAAACTGTTGCTAGTTTCGCTTTATCTGAGT

+

BBBBBFFFFFFFFFFFFFFFFFFFFFFFFFFFFFFFFFFFFFFFFFFFFFFFFFFFFFFFFFFFFFFFFFFFFFFFFFFFFFFFFFFFFFFFFFFFFFFFFFFFBBFFFFFFFFFF<FFBFFFFFB

@GWZHISEQ02:315:C9E6MANXX:5:1107:1408:79724

ACAGAAGCGAGAAGCGAGCTGATTGGTTAGTTTAAATAAGGCTTGGGGTTTTTCCCGGTCTTTTGGGGAACTTGAAACTGAGGTGGGACTTTCCAGAAACTGTTGCTAGTTTCGCTTTATCTGAGT

+

BBBBBFFFFFFFFFFFBFFBFFFFFFFFFFFFFFFFFFFFFFFFFFFF7BFFFFFFFFBFFFFFFFFFFFFFFFFFFFFFFFFFFFFBFBFFFFFFFFFFFFFFFFFFFBFFFFB7/BFFFFFFF/

@GWZHISEQ02:315:C9E6MANXX:5:1110:20868:44138

CTCAGATAAAGCGAAACTAGCAACAGTTTCTGGAAAGTCCCACCTCAGTTTCAAGTTCCCCAAAAGACCGGGAAAAACCCCAAGCCTTATTTAAACTAACCAATCAGCTCGCTTCTCGCTTCTGTA

+

BBBBBFFFFFFFFFFFFFFFFFFFFFFFFFFFFFFFFFFFFFFFFFFFFFFFFFFFFFFFFFFFFFFFFFFFFFFFFFFFFFFFFFFFFFFFFFFFFFFFFFFFFFFFFFFFFFFFFFFFFFFFFF

@GWZHISEQ02:315:C9E6MANXX:5:1111:15950:31915

CTCAGATAAAGCGAAACTAGCAACAGTTTCTGGAAAGTCCCACCTCAGTTTCAAGTTCCCCAAAAGACCGGGAAAAACCCAGATCGGAAGAGCGTCGTGTAGGGAAAGAGTGTTAAGATTAGTGTA

+

BBBBBFFFFFFFFFFFFFFFFFFFFFFFFFFFFFFFFFFFFFFFFFFFFFFFFFFFFFFFFFFFFFFFFFFFFFFFFFFFFFFFFFFFFFFFFFBFFBFFFFFFFFFFFFBFFFFFFFFFFFFBFF

@GWZHISEQ02:315:C9E6MANXX:5:1111:19459:66226

CTCAGATAAAGCGAAACTAGCAACAGTTTCTGGAAAGTCCCACCTCAGTTTCAAGTTCCCCAAAAGACCGGGAAAAACCCCAAGCCTTATTTAAACTAACCAATCAGCTCGCTTCTCGCTTCTGTA

+

BBBBBFB/<BFBFBFFFFFFFFFFFFFFFFFFFFFFBFFFFFFFFFFFFFFFFFFFFFFFFFFFFFFFFFFFFFFFFFBFFFFFFFFFFFFFFFFFFFFFFFFFFFFFB<FFFBFFFBFFFFFFFF

@GWZHISEQ02:315:C9E6MANXX:5:1114:3042:50610

CTCAGATAAAGCGAAACTAGCAACAGTTTCTGGAAAGTCCCACCTCAGTTTCAAGTTCCCCAAAAGACCGGGAAAAACCCCAAGCCTTATTTAAACTAACCAATCAGCTCGCTTCTCGCTTCTGTA

+

BBBBBFFFFFFFFFFFFFFFFFFFFFFFFFFFFFFFFFFFFFFFFFFFFFFFFFFFFFFFFFFFFFFFFFFFFFFFFFFFFFFFFFFFFFFFFFFFFFFFFFFFFFFFFFFFFFFFFFFFFFFFFF

@GWZHISEQ02:315:C9E6MANXX:5:1202:17938:10425

CTCAGATAAAGCGAAACTAGCAACAGTTTCTGGAAAGTCCCACCTCAGTTTCAAGTTCCCCAAAAGACCGGGAAAAACCCCAAGCCTTATTTAAACTAACCAATCAGCTCGCTTCTCGCTTCTGTA

+

BBBBBFFFFFFFFFFFFFFFFFFFFFFFFFFFFFFFFFFFFFFFFFFFFFFFFFFFFFFFFFFFFFFFBBFFFFFFFFFFFFFFFFFFFFFFFFFFFFFFFFFFFFFFFFFFFFFFFFFFFFFFFF

@GWZHISEQ02:315:C9E6MANXX:5:1208:15672:27385

CTCAGATAAAGCGAAACTAGCAACAGTTTCTGGAAAGTCCCACCTCAGTTTCAAGTTCCCCAAAAGACCGGGAAAAACCCCAAGCCTTATTTAAACTAACCAATCAGCTCGCTTCTCGCTTCTGTA

+

BBBBBFFFFFFFFFFFFFFFFFFFFFFFFFFFFFFFFFFFFFFFFFFFBFFFFFFFFFFFFFFFFFFFFFFFFFFFFFFFFFFFFFFFFFFFFFFFFFFFFFFFFFFFFFFFFFFBFFFFFFFFFF

@GWZHISEQ02:315:C9E6MANXX:5:1211:11977:46048

CTCAGATAAAGCGAAACTAGCAACAGTTTCTGGAAAGTCCCACCTCAGTTTCAAGTTCCCCAAAAGACCGGGAAAAACCCCAAGCCTTATTTAAACTAACCAATCAGCTCGCTTCTCGCTTCTGTA

+

BBBBBFFFFFFFFFFFFFFFFFFFFFFFFFFFFFFFFFFFFFFFFFFFFFFFFFFFFFFFFFFFFFFFFFFFFFFFFFFFFFFFFFFFFFFFFFFFFFFFFFFFFFFFFFFFFFFFFFFFFFFFFF

@GWZHISEQ02:315:C9E6MANXX:5:1212:17187:67206

CTCAGATAAAGCGAAACTAGCAACAGTTTCTGGAAAGTCCCACCTCAGTTTCAAGTTCCCCAAAAGACCGGGAAAAACCCCAAGCCTTATTTAAACTAACCAATCAGCTCGCTTCTCGCTTCTGTA

+

BBBBBFFFFFFFBFFFFFFFFFFFFFFFFFFFFFFFFFFFFFFFFFFFFFFFFFFFFFFFFFFFFFFFFFFFFFFFFFBFFFFFFFFFFFFFFFFFFFFFFFFFFFFFFFFFFFFFFFFFFFFFFF

@GWZHISEQ02:315:C9E6MANXX:5:1302:5594:5878

CTCAGATAAAGCGAAACTAGCAACAGTTTCTGGAAAGTCCCACCTCAGTTTCAAGTTCCCCAAAAGACCGGGAAAAACCCCAAGCCTTATTTAAACTAACCAATCAGCTCGCTTCTCGCTTCTGTA

+

BBBBBFFFFFFFFFFFFFFFFFFFFFFFFFFFFFFFFFFFFFFFFFFFFFFFFFFFFFFFFFFFFFFFFFFFFFFFFFFFFFFFFFFFFFFFFFFFFFFFFFFFFFFFFFFFFFFFFFFFFFFFFF

@GWZHISEQ02:315:C9E6MANXX:5:1305:13551:7545

CTCAGATAAAGCGAAACTAGCAACAGTTTCTGGAAAGTCCCACCTCAGTTTCAAGTTCCCCAAAAGACCGGGAAAAACCCCAAGCCTTATTTAAACTAACCAATCAGCAGATCGGAAGAGCGTCGT

+

BBBBBFFFFFFFFFFFFFFFFFFFFFFFFFFFFFFFFFFFBFFFFFFFFFFFFFFBFFBFFFFFFFFFFFFBFFFFFFFFBFFFFFFFFFFFFFFFFFFFFFFFFFFFFFFBFFFFFFFFFFFFF7

@GWZHISEQ02:315:C9E6MANXX:5:1305:9175:59723

CTCAGATAAAGCGAAACTAGCAACAGTTTCTGGAAAGTCCCACCTCAGTTTCAAGTTCCCCAAAAGACCGGGAAAAACCCCAAGCCTTATTTAAACTAACCAATCAGCTCGCTTCTCGCTTCTGTA

+

BBBBBFBFFFFFFFFFFFFFFFFFFFFFFFFFFFFFFFFFFFFFFFFFFFFFFBF<FFFFFFFFFFFFFFFFBFFFFFFFFFFFFFFFFFFFBBFFFBBFFFFFFFFFFFFFFFFFFFFFFFFFFF

@GWZHISEQ02:315:C9E6MANXX:5:1305:2274:76722

CTCAGATAAAGCGAAACTAGCAACAGTTTCTGGAAAGTCCCACCTCAGTTTCAAGTTCCCCAAAAGACCGGGAAAAACCCCAAGCCTTATTTAAACTAACCAATCAGCTCGCTTCTCGCTTCTGTA

+

BBBBBFFFFFFFFFFFFFFFFFFFFFFFFFFFFFFFFFFFFFFFFFFFFFFFFFFFFFFFFFFFFFFFFFFFFFFFFFFFFFFFFFFFFFFFFFFFFFFFFFFFFFFFFFFFFFFFFFFFFFFFFF

@GWZHISEQ02:315:C9E6MANXX:5:1313:8198:62093

CTCAGATAAAGCGAAACTAGCAACAGTTTCTGGAAAGTCCCACCTCAGTTTCAAGTTCCCCAAAAGACCGGGAAAAACCCCAAGCCTTATTTAAACTAACCAATCAGCTCGCTTCTCGCTTCTGTA

+

BBBBBFFFFFFFFFFFFFFFFFFFFFFFFFFFFFFFFFFFFFFFFFFFFFFFFFFFFFFFFFFFFFFFFFFFFFFFFFFFFFFFFFFFFFFFFFFFFFFFFFFFFFFFFFFFFFFFFFFFFFFFFF

@GWZHISEQ02:315:C9E6MANXX:5:1314:12348:30292

CTCAGATAAAGCGAAACTAGCAACAGTTTCTGGAAAGTCCCACCTCAGTTTCAAGTTCCCCAAAAGACCGGGAAAAACCCCAAGCCTTATTTAAACTAACCAATCAGCTCGCTTCTCGCTTCTGTA

+

BBBBBFFFFFFFFFFFFFFFFFFFFFFFFFFFFFFFFFFFFFFFFFFFFFFFFFFFFFFFFFFFFFFFFFFFFFFFFFFFFFFFFFFFFFFFFFFFFFFFFFFFFFFFFFFFBFFFFFFFFFFFFB

@GWZHISEQ02:315:C9E6MANXX:5:1314:9567:95560

CTCAGATAAAGCGAAACTAGCAACAGTTTCTGGAAAGTCCCACCTCAGTTTCAAGTTCCCCAAAAGACCGGGAAAAACCCCAAGCCTTATTTAAACTAACCAATCAGCTCGCTTCTCGCTTCTGTA

+

BBBBBFFFFFFFFFBB/FFFFFFFFFBFFFFFFBFFFFFFFFFFFFFF<FFFFFFFFFFFFFFFFFFFFFF<BFFFFFBFFFFFFFFFFFFFFFBFFFFFFFFFFFFFFFFFFFFBF7BFBFFFFF

@GWZHISEQ02:315:C9E6MANXX:5:2101:2051:97894

CTCAGATAAAGCGAAACTAGCAACAGTTTCTGGAAAGTCCCACCTCAGTTTCAAGTTCCCCAAAAGACCGGGAAAAACCCCAAGCCTTATTTAAACTAACCAATCAGCTCGCTTCTCGCTTCTGTA

+

BBBBBFFFFFFFFFFFFFFFFFFFFFFFFFFFFFFFFFFFFFFFFFFFFFFFFFFFFFFFFFFFFFFFFFFFFFFFFFFFFFFFFFFFFFFFFFFFFFFFFFFFFFFFFFFFFFFFFFFFFFFFFF

@GWZHISEQ02:315:C9E6MANXX:5:2104:8377:71119

CTCAGATAAAGCGAAACTAGCAACAGTTTCTGGAAAGTCCCACCTCAGTTTCAAGTTCCCCAAAAGACCGGGAAAAACCCCAAGCCTTATTTAAACTAACCAATCAGCTCGCTTCTCGCTTCTGTA

+

BBBBB<F<FFFFFFFBFFFFFFBF/B<FFBFBFFFFFFFBF/F<FFFFFFFFF<FF<B</BBFFF<FFBFFFFFFFB/FFFFBFFFFFFFBFFBFFFFFFBF/FFFBFF<BBFFFFFFBFFFFFFF

@GWZHISEQ02:315:C9E6MANXX:5:2109:19261:92573

CTCAGATAAAGCGAAACTAGCAACAGTTTCTGGAAAGTCCCACCTCAGTTTCAAGTTCCCCAAAAGACCGGGAAAAACCCCAAGCCTTATTTAAACTAACCAATCAGCTCGCTTCTCGCTTCTGTA

+

BBBBBFFFFFFFFFFFFFFFFFFFFFFFFFFFFFFFFFFFFFFFFFFFFFFFFFFFFFFFFFFFFFFFFFFFFFFFFFFFFFFFFFFFFFFFFFFFFFFFFBFFFFFFFFFFFFFFFFFFFFFFFF

@GWZHISEQ02:315:C9E6MANXX:5:2202:5015:84504

CTCAGATAAAGCGAAACTAGCAACAGTTTCTGGAAAGTCCCACCTCAGTTTCAAGTTCCCCAAAAGACCGGGAAAAACCCCAAGCCTTATTTAAACTAACCAATCAGCTCGCTTCTCGCTTCTGTA

+

BBBBBFFFBFFFFBFFFFBFFBFFFFFFFFFFFFFFFFFFFFFFFFFFBFFFFFF<FFFFFFFFFFFFFFBBFFFFFFFFFBFFFFFFFFFF/FFFFFFFFFFFFFFFFFFFFBBFFFFFFFFFFB

@GWZHISEQ02:315:C9E6MANXX:5:2205:5077:75331

CTCAGATAAAGCGAAACTAGCAACAGTTTCTGGAAAGTCCCACCTCAGTTTCAAGTTCCCCAAAAGACCGGGAAAAACCCCAAGCCTTATTTAAACTAACCAATCAGCTCGCTTCTCGCTTCTGTA

+

BBBBBFFFFFFFFFFFFFFFFFF<FFFFFFFFFFFFFFFFFFFFFFFFFFFFFFFFFFFFFFFFFFFFFFFFFFFFFFFFFFFFFFFFFFFFFFFFFFFFFFFFFFBFFFFFFFFFFFFFFFFFFF

@GWZHISEQ02:315:C9E6MANXX:5:2206:19629:33262

CTCAGATAAAGCGAAACTAGCAACAGTTTCTGGAAAGTCCCACCTCAGTTTCAAGTTCCCCAAAAGACCGGGAAAAACCCCAAGCCTTATTTAAACTAACCAATCAGCTCGCTTCTCGCTTCTGTA

+

/BB<BFFFFFFFFFFFFFFFF<FFBFFFFFFFFFFFFFFFFFFBFFFFFFBFFFFBFFFFFFFFFFFFFFFFB<FFFFFFFFFFFFFBFFFFFFFFFFFFFFFFFFFFFFFFBFFFFFFFFFFFFF

@GWZHISEQ02:315:C9E6MANXX:5:2206:13950:61250

CTCAGATAAAGCGAAACTAGCAACAGTTTCTGGAAAGTCCCACCTCAGTTTCAAGTTCCCCAAAAGACCGGGAAAAACCCCAAGCCTTATTTAAACTAACCAATCAGCTCGCTTCTCGCTTCTGTA

+

BBBBBFFFFFFFFFFFFFFFFFFFFFFFFFFFFFFFFFFFFFFFFFFFFFFFFFFFFFFFFFFFFFFFFFFFFFFFFFFFFFFFFFFFFFFFFFFFFFFFFFFFFFFFFFFFFFFFFFFFFFFFFF

@GWZHISEQ02:315:C9E6MANXX:5:2206:17750:85696

CTCAGATAAAGCGAAACTAGCAACAGTTTCTGGAAAGTCCCACCTCAGTTTCAAGTTCCCCAAAAGACCGGGAAAAACCCCAAGCCTTATTTAAACTAACCAATCAGCTCGCTTCTCGCTTCTGTA

+

BBBBBFFFFFFFFFFFFFFFFFFFFFFFFFFFFFFFFFFFFFFFFFFFFFFFFFFFFFFFFFFFFFFFFFFFFFFFFFFFFFFFFFFFFFFFFFFFFFFFFFFFFFFFFFFFFFFFFFFFFFFFFF

@GWZHISEQ02:315:C9E6MANXX:5:2208:14341:55349

CTCAGATAAAGCGAAACTAGCAACAGTTTCTGGAAAGTCCCACCTCAGTTTCAAGTTCCCCAAAAGACCGGGAAAAACCCCAAGCCTTATTTAAACTAACCAATCAGCTCGCTTCTCGCTTCTGTA

+

BBBBBFFFFFFFFFFFFFFFFFFFFFFFFFFFFFFFFFFFFFFFFFFFFFFFFFFFFFFFF<FFFFFFFFFF<FFFFFFFFFFFFFFFFFFFFFFFFFFFFFBFFFFFFFFFFFFFFFFFBBFFFF

@GWZHISEQ02:315:C9E6MANXX:5:2209:6807:11846

CTCAGATAAAGCGAAACTAGCAACAGTTTCTGGAAAGTCCCACCTCAGTTTCAAGTTCCCCAAAAGACCGGGAAAAACCCCAAGCCTTATTTAAACAGATCGGAAGAGCGTCGTGTAGGGAAAGAG

+

BBBBBFFFFFFFFFFFFFFFFFFFFFFFFFFFFFFFFFFFFFFFFFFFFFFFFFFFFFFFFFFFFFFFFFFFFFFFFFFFFFFFFFFFFFFFFFFFFFFFFFFFBFFFFFFFF7BF<FFFFFFFF<

@GWZHISEQ02:315:C9E6MANXX:5:2214:14017:63178

CTCAGATAAAGCGAAACTAGCAACAGTTTCTGGAAAGTCCCACCTCAGTTTCAAGTTCCCCAAAAGACCGGGAAAAACCCCAAGCCTTATTTAAACTAACCAATCAGCTCGCTTCTCGCTTCTGTA

+

B/</BFFFFFFFBBF<BFFFFFFFFFFFFFFFFFBF<BF<FFFFFFF<<FBFBFBFFFFFBFFB<FFFFFFFFFFFFFFBFBFFFFFFBFFFFFFFFBFFFFFBFFFFFFFFFFFBFFFFFFFFFF

@GWZHISEQ02:315:C9E6MANXX:5:2307:17132:99116

CTCAGATAAAGCGAAACTAGCAACAGTTTCTGGAAAGTCCCACCTCAGTTTCAAGTTCCCCAAAAGACCGGGAAAAACCCCAAGCCTTATTTAAACTAACCAATCAGCTCGCTTCTCGCTTCTGTA

+

BBBBBFFFFFFFFFFFFFFFFFFFFFFFFFFFFFFFFFFFFFFFFFFFFFFFFFFFFFFFFFFFFFFFFFFFFFFFFFFFFFFFFFFFFFFFFFFFFFFFFFFFFFFFFFFFFFFFFFFFFFFFFF

@GWZHISEQ02:315:C9E6MANXX:5:2309:10498:73934

CTCAGATAAAGCGAAACTAGCAACAGTTTCTGGAAAGTCCCACCTCAGTTTCAAGTTCCCCAAAAGACCGGGAAAAACCCCAAGCCTTATTTAAACTAACCAATCAGCTCGCTTCTCGCTTCTGTA

+

BBBBBFFFFFFFFFFFFFFFFFFFFFFFFFFFFFFFFFFFFFFFFFFFFFFFFFFFFFFFFFFFFFFFFFFFFFFFFFFFFFFFFFFFFFFFFFFFFFFFFFFFFFFFFFFFFFFFFFFFFFFFFF

@GWZHISEQ02:315:C9E6MANXX:5:2313:12054:81374

CTCAGATAAAGCGAAACTAGCAACAGTTTCTGGAAAGTCCCACCTCAGTTTCAAGTTCCCCAAAAGACCGGGAAAAACCCCAAGCCTTATTTAAACTAACCAATAGATCGGAAGAGCGTCGTGTAG

+

BBBBBFFFFFFFFFFFFFFFFFFFFFFFFFFFFFFFFFFFFFFFFFFFFFFFFFFFFFFFFFFFFFFFFFFFFFFFFFFFFFFFFFFFFFFFFFFFBFFBFFFFFFFFFFFFFF<FFFFFFFFBFF

@GWZHISEQ02:315:C9E6MANXX:5:2314:3962:43123

CTCAGATAAAGCGAAACTAGCAACAGTTTCTGGAAAGTCCCACCTCAGTTTCAAGTTCCCCAAAAGACCGGGAAAAACCCCAAGCCTTATTTAAACTAACCAATCAGCTCGCTTCTCGCTTCTGTA

+

BBBBBFFFFFFFFFFFFFFFFFFFFFFFFFFFFFFFFFFFFFFFFFFFFFFFFFFFFFFFFFFFFFFFFFFFFFFFFFFFFFFFFFFFFFFFFFFFFFFFFFFFFFFFFFFFFFFFFFFFFFFFFF

@GWZHISEQ02:315:C9E6MANXX:5:2315:13961:21167

CTCAGATAAAGCGAAACTAGCAACAGTTTCTGGAAAGTCCCACCTCAGTTTCAAGTTCCCCAAAAGACCGGGAAAAACCCCAAGCCTTATTTAAACTAACCAATCAGCTCGCTTCTCGCTTCTGTA

+

B/<<BFFFBF</<FFBBFFFFFFFFF<FFFBFF</BFFFFFFFFFFFF<FFFFFFFFFFB/FFFB/<FFF/B//FBFB</FF/</<BFFBFFBFFFFFB/FFB/BFFF<FB<77BFFFFBFF//BF

@GWZHISEQ02:315:C9E6MANXX:5:2315:16591:90637

CTCAGATAAAGCGAAACTAGCAACAGTTTCTGGAAAGTCCCACCTCAGTTTCAAGTTCCCCAAAAGACCGGGAAAAACCCCAAGCCTTATTTAAACTAACCAATCAGCTCGCTTCTCGCTTCTGTA

+

BBB<BFFFFFFFFFFFFFFFFFFFFFFFFBFFFFFFFFFFFFFFFFFFFB<FBFFFFF<FFFFFFFFFFFFFF<FFFFFFFF<FFFFFFFFFFFBFFFFFFFFFFBFFFFFFFFFFFFFFFFFFFF

@GWZHISEQ02:315:C9E6MANXX:5:2316:4811:4426

CTCAGATAAAGCGAAACTAGCAACAGTTTCTGGAAAGTCCCACCTCAGTTTCAAGTTCCCCAAAAGACCGGGAAAAACCCCAAGCCTTATTTAAACTAACCAATCAGCTCGCTTCTCGCTTCTGTA

+

BB<BBFFFFFFFFFFFFFFFFFFFFFFFFFFFFBFFFFFFFFFFFFFFFFFFFFFFF<FFFFFFFFFFFFFFFFFFFFFFFFFFFFFFFFFFF<BFFFFFFFFFFFFFFFFFFFFFFFFFFFFFFF

@GWZHISEQ02:315:C9E6MANXX:5:2316:17050:74928

CTCAGATAAAGCGAAACTAGCAACAGTTTCTGGAAAGTCCCACCTCAGTTTCAAGTTCCCCAAAAGACCGGGAAAAACCCCAAGCCTTATTTAAACTAACCAATCAGCTCGCTTCTCGCTTCTGTA

+

BBBBBFFFFFFFFFFFFFFBFFFFFFFFFFFFFFFFFFFFFFFFFFFFFBFFFFFFFFFFFFFFFFFFFFFFFFFFFFFFFFFFFFFFFFFFFFFFFFFFFFFFFFFFFFFFFFFFFFFFFFFFFF

@GWZHISEQ02:315:C9E6MANXX:5:1111:15950:31915

GGGTTTTTCCCGGTCTTTTGGGGAACTTGAAACTGAGGTGGGACTTTCCAGAAACTGTTGCTAGTTTCGCTTTATCTGAGAGATCGGAAGAGCACACGTCTGAACTCCAGTCACATTACTCGATCT

+

BBBBBFFFFFFFFFFFFFFFFFFFFFFFFFFFFFFFFFFFFFFFFFFFFFFFFFFFFFFFFFFFFFFFFFFFFFFFFFFFFFFFFFFFFFFFFFFFFFFFFFFFFFFFFFFFFFFFFFFFFFFFBB

@GWZHISEQ02:315:C9E6MANXX:5:1305:13551:7545

GCTGATTGGTTAGTTTAAATAAGGCTTGGGGTTTTTCCCGGTCTTTTGGGGAACTTGAAACTGAGGTGGGACTTTCCAGAAACTGTTGCTAGTTTCGCTTTATCTGAGAGATCGGAAGAGCACACG

+

BBBBBFFFFFFFFBFFFF<BFFFFFFFFFFFFFFFFFFFFBFFFBFFFFFF<FFFFFFFFFFFBFFFFFFFFFFFFFFFFF/FFFFFFFFFFFFFFBFFBFFFF<FFF/FBFBFFBBFFFFFFFFF

@GWZHISEQ02:315:C9E6MANXX:5:2114:13553:89329

TACAGAAGCGAGAAGCGAGCTGATTGGTTAGTTTAAATAAGGCTTGGGGTTTTTCCCGGTCTTTTGGGGAACTTGAAACTGAGGTGGGACTTTCCAGAAACTGTTGCTAGTTTCGCTTTATCTGAG

+

BBBBBFFFFFFFFFFFFFFFFFFFFFFFFFFFFFFFFFFFFFFFFFFFFFFFFFFFFFFBFFFFFFFFFFFFFFFFFFFFFFFFBFFFBFBFFFFFFFFFFFFFFFFFFFFFFFFFFFFFFFFFFF

@GWZHISEQ02:315:C9E6MANXX:5:2209:6807:11846

GTTTAAATAAGGCTTGGGGTTTTTCCCGGTCTTTTGGGGAACTTGAAACTGAGGTGGGACTTTCCAGAAACTGTTGCTAGTTTCGCTTTATCTGAGAGATCGGAAGAGCACACGTCTGAACTCCAG

+

BBBBBBFFFFFFFFFFFFFFFFFFFFFFFFFFFFFFBFFBFFFFFFFFFFFFFFFFFFFFFFFFFFFFFFFFFFFFFFFFFFFFFFFFFFFFF/FBFFFFFFFFFFFFFF<FFFFFFFFFFFFFF7

@GWZHISEQ02:315:C9E6MANXX:5:2313:12054:81374

ATTGGTTAGTTTAAATAAGGCTTGGGGTTTTTCCCGGTCTTTTGGGGAACTTGAAACTGAGGTGGGACTTTCCAGAAACTGTTGCTAGTTTCGCTTTATCTGAGAGATCGGAAGAGCACACGTCTG

+

<BBBBFFFFFFFFFFFFFFBFFFFFFFFFFFFFFFFFFFFFFFFFFFBFFFFFFBFFFF<BFFFFFFFFFBFFFFFFFFFFFFBFFFFFFFFFFFFFFBFBFFFFFFFFFFFFFFFFFFFFFFFFB

@GWZHISEQ02:315:C9E6MANXX:5:2316:13171:82165

TACAGAAGCGAGAAGCGAGCTGATTGGTTAGTTTAAATAAGGCTTGGGGTTTTTCCCGGTCTTTTGGGGAACTTGAAACTGAGGTGGGACTTTCCAGAAACTGTTGCTAGTTTCGCTTTATCTGAG

+

BBBBBFFFFFFFFFFFFFFFFFFFFFFFFFFFFFFFFFFFFFFFFFFFFFFFFFFFFFFFFFFFFFFFFFFFFFFFFFFFFFFFFFFFFFFFFFFFFFFFFFFFFFFFFFFFFFFFFFFFFFB/B7

@GWZHISEQ02:315:C9E6MANXX:5:1106:16002:29431

TCAGATAAAGCGAAACTAGCAACAGTTTCTGGAAAGTCCCACCTCAGTTTCAAGTTCCCCAAAAGACCGGGAAAAACCCCAAGCCTTATTTAAACTAACCAATCAGCTCGCTTCTCGCTTCTGTAA

+

BBBBBFFFFFFFFFFFFFFFFFFFFFFFFFFFFFFFFFFFFFFFFFFFFFFFFFF<FFFFFFFFFF<<BB/F<BBFFFFFFFFFFFFFFFFFFBFFFFFFBFF<<F//F/7//7B7/B/F<FFFB7

@GWZHISEQ02:315:C9E6MANXX:5:1102:20741:49678

TTACAGAAGCGAGAAGCGAGCTGATTGGTTAGTTTAAATAAGGCTTGGGGTTTTTCCCGGTCTTTTGGGGAACTTGAAACTGAGGTGGGACTTTCCAGAAACTGTTGCTAGTTTCGCTTTATCTGA

+

BBBBBFFFFFFFFFFFFFFFFFFFFFFFFFFFFFFFFFFFFFFFFFFFFFFFFFFFFFFF7B/FFFFFFFFFFFFFFFFFFFFFFFFFFBF<FFFFFFFFFF7FFFFFFFFFF<FBFFFFBBFFF/

@GWZHISEQ02:315:C9E6MANXX:5:2103:12429:65091

TTACAGAAGCGAGAAGCGAGCTGATTGGTTAGTTTAAATAAGGCTTGGGGTTTTTCCCGGTCTTTTGGGGAACTTGAAACTGAGGTGGGACTTTCCAGAAACTGTTGCTAGTTTCGCTTTATCTGA

+

BBBBBFFFFFFFFFFFFFFFFFFFFFFFFFFFFFFFFFFFFFFFFFFFFFBFFFFFFFFFBFFFFFFFFB<FFFFFFFFFFFFFFFFFFB/7FFFFFFFFFFF<FFFFF7FFFFF<<BFFFFFFF7

@GWZHISEQ02:315:C9E6MANXX:5:1214:19777:38170

GTTACAGAAGCGAGAAGCGAGCTGATTGGTTAGTTTAAATAAGGCTTGGGGGTTTTCCCGGTCTTTTGGGGAACTTGAAACTGAGGTGGGACTTTCCAGAAACTGTTGCTAGTTTCGCTTTATCTG

+

/<<BB/<B<FFFFB//</F/FFF/</<</</BF<//<//B//F<FF<BBBB/<<BFFFFBB<F//FFFBBFBFFFBF/B////<7/FFF</FFF/7FF/////FFF//F/FB/7B/BFBF/FF/77

@GWZHISEQ02:315:C9E6MANXX:5:2102:13083:79901

GTTACAGAAGCGAGAAGCGAGCTGATTGGTTAGTTTAAATAAGGCTTGGGGTTTTTCCCGGTCTTTTGGGGAACTTGAAACTGAGGTGGGACTTTCCAGAAACTGTTGCTAGTTTCGCTTTATCTG

+

BBBBBFFFFFFFFFFFFFF<FFFFFFFFFFFFFFFFFFFFFFFFFFFFFFFFFFFFFFFFFFFFFFFFBFF<BFFFFFFFFFFFBFFFFFFFFFFFFFFFFFFFFFFFFFFFFFFFFFBFFBFFF7

@GWZHISEQ02:315:C9E6MANXX:5:2107:4831:98719

GTTACAGAAGCGAGAAGCGAGCTGATTGGTTAGTTTAAATAAGGCTTGGGGTTTTTCCCGGTCTTTTGGGGAACTTGAAACTGAGGTGGGACTTTCCAGAAACTGTTGCTAGTTTCGCTTTATCTG

+

BBBBB<FFFFFFFBFFFFFFFFFFFFFFFFFFFFFFFFFFFFFFFFFFFFF/FFFFFFFBFFFFFFF<B<F<FFFFFBFFFFFFFFBFFFFFFFFFFFFFFFBFFFFFFFFFFBFB<BFFFFFFFB

@GWZHISEQ02:315:C9E6MANXX:5:2214:16874:26530

GTTACAGAAACGAGAAGCGAGCTGATTGGTTAGTTTAAATAAGGCTTGGGGTTTTTCCCGGTCTTTTGGGGAACTTGAAACTGAGGTGGGACTTTCCAGAAACTGTTGCTAGTTTCGCTTTATCTG

+

BBBBBFFFFFFFFFFFFFFFFFFFFFFFFFFFFFFFFFFFFFFFFFFFFFFFFFFFFFFFFFFFFFFFFFFFFFFFFFFFFFFFFFFFFFBFFFFFFFFFFFFFFFFFFFFFFFFFFFFFFFFFFF

@GWZHISEQ02:315:C9E6MANXX:5:2316:20131:23398

AGATAAAGCGAAACTAGCAACAGTTTCTGGAAAGTCCCACCTCAGTTTCAAGTTCCCCAAAAGACCGGGAAAAACCCCAAGCCTTATTTAAACTAACCAATCAGCTCGCTTCTCGCTTCTGTAACC

+

BBBBBFFFFFFFFBFFFFFFFFFFFFFFFFFFFFFFFFFFFFFFFFFFFFFFFFFFFFFFFFFFFFFFFFFFFFFFFFFFFFFFFFFFFFFFFFFFFFFFFFFFFFFFFFFFFFFFFFFFFF<FFF

@GWZHISEQ02:315:C9E6MANXX:5:1113:13534:88029

GGTTACAGAAGCGAGAAGCGAGCTGATTGGTTAGTTTAAATAAGGCTTGGGGTTTTTCCCGGTCTTTTGGGGAACTTGAAACTGAGGTGGGACTTTCCAGAAACTGTTGCTAGTTTCGCTTTATCT

+

BBBBBFFBBFFFFFFFFFFFFFFFFFFFFFFFFFFFFFFFFFFFFFFFFFFFBFFFFFFFFF<FBFFFFFFFFFFFFFFFFFFFFFFFFFFFFFFFFFFFFFFFFFFFFFFFF<FFFFFF/BFFFF

@GWZHISEQ02:315:C9E6MANXX:5:1205:4572:65745

GGTTACAGAAGCGAGAAGCGAGCTGATTGGTTAGTTTAAATAAGGCTTGGGGTTTTTCCCGGTCTTTTGGGGAACTTGAAACTGAGGTGGGACTTTCCAGAAACTGTTGCTAGTTTCGCTTTATCT

+

BBBBBFFFFFFFFFFFFFFFFFFFFFFFFFFFFFFFFFFFFFFFFFFFFFFFFFFFFFFFFFFFFFFFFFFFBFFFFFFFFFFFFFFFFFFFFFFFFFFFFFFFFFFFFFFFFFFFFFFFFFFFFB

@GWZHISEQ02:315:C9E6MANXX:5:1213:11410:80714

GGTTACAGAAGCGAGAAGCGAGCTGATTGGTTAGTTTAAATAAGGCTTGGGGTTTTTCCCGGTCTTTTGGGGAACTTGAAACTGAGGTGGGACTTTCCAGAAACTGTTGCTAGTTTCGCTTTATCT

+

BBBBBBFFFFFFFFFFFFFFFFFFFFFFFFFFFFFFFFFFFFFFFFFFFFFFFFFFFFFFFFFFFFFFFFFFFFFFFFFFFFFFFFFBFFFFFFFFFFFFFFFFFFFFFFFFFFFFFFFFBFFFFF

@GWZHISEQ02:315:C9E6MANXX:5:2214:14017:63178

GGTTACAGAAGCGAGAAGCGAGCTGATTGGTTAGTTTAAATAAGGCTTGGGGTTTTTCCCGGTCTTTTGGGGAACTTGAAACTGAGGTGGGACTTTCCAGAAACTGTTGCTAGTTTCGCTTTATCT

+

BBBBB<FFFFFFFFFFFF<//<BBFF/<FFFFBFF<FBFFFFF<FFFFFFFFFFFBF/FFF/<FFFFFF<<</FFFFFBFBFFFFF<BF<FFF<7BF<FFFFFFFBFFBFF<F/7<7BFFFFFFBF

@GWZHISEQ02:315:C9E6MANXX:5:2307:17132:99116

GGTTACAGAAGCGAGAAGCGAGCTGATTGGTTAGTTTAAATAAGGCTTGGGGTTTTTCCCGGTCTTTTGGGGAACTTGAAACTGAGGTGGGACTTTCCAGAAACTGTTGCTAGTTTCGCTTTATCT

+

BBBBBFFFFFFFFFFFFFFFFFFFFFFFFFFFFFFFFFFFFFFFFFFFFFFFFFFFFFFFFFFFFFFFFFFFBFFFFFFFFFFFFFFFFFFFFFFFFFFFFFFFFFFFFFFFFFFFFFFFFFFFFF

@GWZHISEQ02:315:C9E6MANXX:5:1108:18413:77311

CGGTTACAGAAGCGAGAAGCGAGCTGATTGGTTAGTTTAAATAAGGCTTGGGGTTTTTCCCGGTCTTTTGGGGAACTTGAAACTGAGGTGGGACTTTCCAGAAACTGTTGCTAGTTTCGCTTTATC

+

/<B<<F<BFF<FFFFFBBFF<FFBFFFFFFBFFFFFFFF<FFFFFFFFFFFFFF<BFFBBBFF/BBF<FFFFF/B7BF<FFBFF/<7/</FF<<F/F<F/7FFB//<F<<F/7FFBFFFF/7FBF/

@GWZHISEQ02:315:C9E6MANXX:5:1113:17157:66817

CGGTTACAGAAGCGAGAAGCGAGCTGATTGGTTAGTTTAAATAAGGCTTGGGGTTTTTCCCGGTCTTTTGGGGAACTTGAAACTGAGGTGGGACTTTCCAGAAACTGTTGCTAGTTTCGCTTTATC

+

BBBBBFFFFFFFFFFFFFFFFFFFFFFFFFFFFFFFFFFFFFFFFFFFFFFFFFFFFFFFFFFFFBFFFFFFFFFFFFFFFFFFFFFFFFFFFFFFFFFFFFFFFFFFFFFFFFFFFFFFFFFFFB

@GWZHISEQ02:315:C9E6MANXX:5:1114:21325:45825

CGGTTACAGAAGCGAGAAGCGAGCTGATTGGTTAGTTTAAATAAGGCTTGGGGTTTTTCCCGGTCTTTTGGGGAACTTGAAACTGAGGTGGGACTTTCCAGAAACTGTTGCTAGTTTCGCTTTATC

+

BBBBBFFFFFFFFBFFFFFFFFFFFFFFFFF/FFFFFFFFFFFFFFFFFFFFFBFFFF<<FFFB<FFF<BBFFFFFBFBFFF//FFFF//FFB<BB/BBFFFFB<B<FBF7BF<FBFFFFBB7FFB

@GWZHISEQ02:315:C9E6MANXX:5:1307:6212:34837

CGGTTACAGAAGCGAGAAGCGAGCTGATTGGTTAGTTTAAATAAGGCTTGGGGTTTTTCCCGGTCTTTTGGGGAACTTGAAACTGAGGTGGGACTTTCCAGAAACTGTTGCTAGTTTCGCTTTATC

+

BBB<BBFFBFFFBFBFFFFFFB<B<BFFFFFFF<FFFBFFFFFFFFFFFFFFFBF<FF/<BFFB/7/<<BBFF7/<BFF<FB/FFFBF/<//FFBFF/FFFF<FFFFFFFFBFFFFF/<BFFFBF/

@GWZHISEQ02:315:C9E6MANXX:5:2115:18890:11954

CGGTTACAGAAGCGAGAAGCGAGCTGATTGGTTAGTTTAAATAAGGCTTGGGGTTTTTCCCGGTCTTTTGGGGAACTTGAAACTGAGGTGGGACTTTCCAGAAACTGTTGCTAGTTTCGCTTTATC

+

BBBBBFFBFFFFFFFFFFFFFBF<FFFFFBFFFFFFFFFFFFFFFFFFFFFFF<BBF<FFFFBFBFBFFFFFF77BBFFFFFFBF/BF/FFFBFBFBFFFFFBFFFFFBFF<FFBFF<FF<FB7FB

@GWZHISEQ02:315:C9E6MANXX:5:2115:19519:25736

CGGTTACAGAAGCGAGAAGCGAGCTGATTGGTTAGTTTAAATAAGGCTTGGGGTTTTTCCCGGTCTTTTGGGGAACTTGAAACTGAGGTGGGACTTTCCAGAAACTGTTGCTAGTTTCGCTTTATC

+

BBBBBFFFFFFFFFFFFFFFFFFFFFFFFFFFFFFFFFFFFFFFFFFFFFFFF7FFFFFFFFF7F<FFFFFBF/FFFFBFFFFFF<FFFBFFFFFFBFFFFFFFFFFFFFFFFBFFFFFFBFFFBF

@GWZHISEQ02:315:C9E6MANXX:5:2209:6370:36263

CGGTTACAGAAGCGAGAAGCGAGCTGATTGGTTAGTTTAAATAAGGCTTGGGGTTTTTCCCGGTCTTTTGGGGAACTTGAAACTGAGGTGGGACTTTCCAGAAACTGTTGCTAGTTTCGCTTTATC

+

BBBBBFFFFFFFFFFFFFFFFFFFFFFFFFFFFFFFFFFFFFFFFFFFFFFFFBFFFFFFFFFFFFFFFFFFFFFFFFFFFFFFFFFFFFFFFFFFFFFFFFFFFFFFFFFFFFFFFFFFFFFFFF

@GWZHISEQ02:315:C9E6MANXX:5:2212:15501:83468

AGGTTACAGAAGCGAGAAGCGAGCTGATTGGTTAGTTTAAATAAGGCTTGGGGTTTTTCCCGGTCTTTTGGGGAACTTGAAACTGAGGTGGGACTTTCCAGAAACTGTTGCTAGTTTCGCTTTATC

+

BBBBBFFFFFFFFFFFFFFFFFFFFFFFFFFFFFFFFFFFFFFFFFFFFFFFFFFFFFFFFFFFFFFFFFFFFFFFFFFFFFFFFFFFFFFFFFFFFFFFFFFFFFFFFFFFFFFFFFFFFFFFF<

@GWZHISEQ02:315:C9E6MANXX:5:1207:17708:94205

GCGGTTACAGAAGCGAGAAGCGAGCTGATTGGTTAGTTTAAATAAGGCTTGGGGTTTTTCCCGGTCTTTTGGGGAACTTGAAACTGAGGTGGGACTTTCCAGAAACTGTTGCTAGTTTCGCTTTAT

+

BBBBBFFFFFFFFFFFFFFFFFFFFFFFFFFFFFFFFFFFFFFFFFFFFFFFFFFFFFFFFFFFFFFFFFFFFFFFFFFFFFFFFFFFFFFFFFFFFFFFFFFFFFFFFFFFFFFFFFFFFFFFFF

@GWZHISEQ02:315:C9E6MANXX:5:1301:1712:70178

GCGGTTACAGAAGCGAGAAGCGAGCTGATTGGTTAGTTTAAATAAGGCTTGGGGTTTTTCCCGGTCTTTTGGGGAACTTGAAACTGAGGTGGGACTTTCCAGAAACTGTTGCTAGTTTCGCTTTAT

+

BBBBBFFFFFFFFFFFFFFFFFFFFFFFFFFFFFFFFFFFFFFFFFFFFFFFFF<FFFFFFFFFFFFFFFFFFFFFFFFFFFFFFFFFFFFFFFFFFFFFFFFFFFFFFFFFFFFFFFFFF<FFFF

@GWZHISEQ02:315:C9E6MANXX:5:2102:19793:73202

GTGGTTACAGAAGCGAGAAGCGAGCTGATTGGTTAGTTTAAATAAGGCTTGGGGTTTTTCCCGGTCTTTTGGGGAACTTGAAACTGAGGTGGGACTTTCCAGAAACTGTTGCTAGTTTCGCTTTAT

+

BBBBBFFFFFFFFFFFFFFFFFFFFFFFFFFFFFFFFFFFFFFFFFFFFFFFFF/<FFFFFFFFBFFFFFFFFF/BBFFFFFFFFFFFFBFFFBFFFFFFFFFFF/FFFFFFFFFFFFFBFFBFFF

@GWZHISEQ02:315:C9E6MANXX:5:2214:6053:83011

GCGGTTACAGAAGCGAGAAGCGAGCTGATTGGTTAGTTTAAATAAGGCTTGGGGTTTTTCCCGGTCTTTTGGGGAACTTGAAACTGAGGTGGGACTTTCCAGAAACTGTTGCTAGTTTCGCTTTAT

+

B/<BB</BFFFFFFFBFFFFFFFFFFFFFFFFFFFFFBFFFFFFBFFFFFFFFFFBFBFFFFFFBF/FFFFFFFFFFFFFFFFFFFFFBFFFF<<FFFFFFF/F/BFFBBBBFFFFFFFFFFFFB<

@GWZHISEQ02:315:C9E6MANXX:5:2309:5424:29166

GCGGTTACAGAAGCGAGAAGCGAGCTGATTGGTTAGTTTAAATAAGGCTTGGGGTTTTTCCCGGTCTTTTGGGGAACTTGAAACTGAGGTGGGACTTTCCAGAAACTGTTGCTAGTTTCGCTTTAT

+

BBBBBFFFFFFFFFFFFFFFFFFFFFFFFFFFFFFFFFFFFFFFFFFFFFFFFF<FFFFFFFFFFFFFFFFFFFFFFFFFFFFFFFFFFFFFFFFFFFFFFFFFFFFFFFFFFFFFFFFFFF<FFF

@GWZHISEQ02:315:C9E6MANXX:5:2315:9328:46536

GCGGTTACAGAAGCGAGAAGCGAGCTGATTGGTTAGTTTAAATAAGGCTTGGGGTTTTTCCCGGTCTTTTGGGGAACTTGAAACTGAGGTGGGACTTTCCAGAAACTGTTGCTAGTTTCGCTTTAT

+

BBBBBFFFFFFFFFFFFFFFFFFFFFFFFFFFFFFFFFFFFFFFFFFFFFFFFFFBFFFFFFFFFFFFFFFFFBFFFFFFFFFFFFFFFFFFFFFFFFFFFFFFFFFFFFFFFFFFFFFFFFBFFF

@GWZHISEQ02:315:C9E6MANXX:5:1108:6501:59549

CGCGGTTACAGAAGCGAGAAGCGAGCTGATTGGTTAGTTTAAATAAGGCTTGGGGTTTTTCCCGGTCTTTTGGGGAACTTGAAACTGAGGTGGGACTTTCCAGAAACTGTTGCTAGTTTCGCTTTA

+

BBBBBFFFFFFFFFFFFFFFFFFFFFFFFFFFFFFFFFFFFFFFFFFFFFFFFFFFFFFFFFFFF<FFFFFBFFFFFFFFFFFFFFFFFFFFFFFFFFFFFFFFFFFFFFFFFFFFFFFFFFFFF7

@GWZHISEQ02:315:C9E6MANXX:5:1111:19459:66226

CGCGGTTACAGAAGCGAGAAGCGAGCTGATTGGTTAGTTTAAATAAGGCTTGGGGTTTTTCCCGGTCTTTTGGGGAACTTGAAACTGAGGTGGGACTTTCCAGAAACTGTTGCTAGTTTCGCTTTA

+

BBBBBBFFFFFFFFFFF</FBBF/FFFF/FBFFBFFFFF/F/FFFFFFFFFFFFF<BFFFFF/FBFFFFFFFFBF7BFFFFBFFFF<FFFFFFFFFFFFBFFFFFFFBF7BF/FBF7BFFBBFFF/

@GWZHISEQ02:315:C9E6MANXX:5:1112:16815:79486

TGCGGTTACAGAAGCGAGAAGCGAGCTGATTGGTTAGTTTAAATAAGGCTTGGGGTTTTTCCCGGTCTTTTGGGGAACTTGAAACTGAGGTGGGACTTTCCAGAAACTGTTGCTAGTTTCGCTTTA

+

<</</FBBBFFFFF<FFFBBFFF/F<BF/FF<FF<BFFFFBFFFFFFFBFFFFFFBFFFFBFFFFF/7FFFFBBFFFFFFFFFF/BFF<F/B<FBFFFFFFFFF/FFFFFFFFFFFFBF<F<B/B/

@GWZHISEQ02:315:C9E6MANXX:5:1204:14033:10006

AGCGGTTACAGAAGCGAGAAGCGAGCTGATTGGTTAGTTTAAATAAGGCTTGGGGTTTTTCCCGGTCTTTTGGGGAACTTGAAACTGAGGTGGGACTTTCCAGAAACTGTTGCTAGTTTCGCTTTA

+

BBBBBFFFFFFFFFFFFFBFFFF<BFFFFFFFFFFFFFFFFFFFFFFFFFFFFFFFFF/BBFFFFBFFFFFFFFFBBFFFFF<FFBFFFFFFFFFF<F<FFFFFB/BFFFFFFBFFF<FBFFFFF/

@GWZHISEQ02:315:C9E6MANXX:5:1215:10498:98092

CGCGGTTACAGAAGCGAGAAGCGAGCTGATTGGTTAGTTTAAATAAGGCTTGGGGTTTTTCCCGGTCTTTTGGGGAACTTGAAACTGAGGTGGGACTTTCCAGAAACTGTTGCTAGTTTCGCTTTA

+

BBBBBFFFFFFFFFFFFFFFFFFFFFFFFFFFFFFFFFFFFFFFFFFFFFFFFFFBFFFFFFFFFFFFFFFFFFFFFFFFFFFFFFFFFFFFFFFFFFFFFFFFFFFFFFFFFFFFFFFFFFFFFF

@GWZHISEQ02:315:C9E6MANXX:5:1314:9496:46989

GTCGGTTACAGAAGCGAGAAGCGAGCTGATTGGTTAGTTTAAATAAGGCTTGGGGTTTTTCCCGGTCTTTTGGGGAACTTGAAACTGAGGTGGGACTTTCCAGAAACTGTTGCTAGTTTCGCTTTA

+

//</BBB//FBBFFBFBF/BF<<BFFFFFFFFBFFFFFFFBFFFFFFBFFFF<FF<BFFFFFFFFF<FB<BF/BFFFFFFB//7F<FBFFFFBBBF/<B<7/<//<7/FFFBFFBBBBFFFFFBF7

@GWZHISEQ02:315:C9E6MANXX:5:1314:7031:58369

CGCGGTTACAGAAGCGAGAAGCGAGCTGATTGGTTAGTTTAAATAAGGCTTGGGGTTTTTCCCGGTCTTTTGGGGAACTTGAAACTGAGGTGGGACTTTCCAGAAACTGTTGCTAGTTTCGCTTTA

+

BBBBBFFFFFFFFFFFFFFFFFFFFFFFFFFFFFFFFFFFFFFFFFFFFFFFFFF<FFFFFFFFFFFFFFFFFFFBFFFF<FBFFFFFFFFFFFFFFFFFFFFFFFFFFFFFFFFFFFBFFFFFFB

@GWZHISEQ02:315:C9E6MANXX:5:2106:16797:46760

CGCGGTTACAGAAGCGAGAAGCGAGCTGATTGGTTAGTTTAAATAAGGCTTGGGGTTTTTCCCGGTCTTTTGGGGAACTTGAAACTGAGGTGGGACTTTCCAGAAACTGTTGCTAGTTTCGCTTTA

+

BB<</FBFFFFFFFFBFFFBFFFBFFFFFBBFFFFBFFFFFBFFFFFFFFFFFFF<FFFFFFFFFBFFFFFFFFF/FB7FFFFFFFF/FFFFFF//FFFFF<BFF/BB/F<FB/B<BFB7<<FBF/

@GWZHISEQ02:315:C9E6MANXX:5:2201:8647:61634

GGCGGTTACAGAAGCGAGAAGCGAGCTGATTGGTTAGTTTAAATAAGGCTTGGGGTTTTTCCCGGTCTTTTGGGGAACTTGAAACTGAGGTGGGACTTTCCAGAAACTGTTGCTAGTTTCGCTTTA

+

BBBBBFFFFFFFFFFFFFFFFFFFFFFFFFFFFFFFFFFFFFFFFFFFFFFFFFFFFFFFFFFFBFFFFFFFFFFFFFFFFFFFFFFFFFFFFFBFFFFFFFFFFFFFFFFBFFFFFFFFFFFBF7

@GWZHISEQ02:315:C9E6MANXX:5:2202:5015:84504

CGCGGTTACAGAAGCGAGAAGCGAGCTGATTGGTTAGTTTAAATAAGGCTTGGGGTTTTTCCCGGTCTTTTGGGGAACTTGAAACTGAGGTGGGACTTTCCAGAAACTGTTGCTAGTTTCGCTTTA

+

BB/BBBBBFFFBF/BBFFFF<FF<FF/FF/FFFFFFFBBBBFFFFFFFFFBF7FF7FFF<FFFF<<BBF/B<B<B/B/F/FBBF<FFBFFFFBB<FFFFFFFFBFFFBFF7F<BFFBFFFFF<FF7

@GWZHISEQ02:315:C9E6MANXX:5:2205:15828:46876

CTCGGTTACAGAAGCGAGAAGCGAGCTGATTGGTTAGTTTAAATAAGGCTTGGGGTTTTTCCCGGTCTTTTGGGGAACTTGAAACTGAGGTGGGACTTTCCAGAAACTGTTGCTAGTTTCGCTTTA

+

BBBBBFFFFFFFFFFFFFFFFFFFFFFFFFFFFFFFFFFFFFFFFFFFFFFFFFFFFFFFFFFFFBFFFFFFFFFFFFFFFFFFFFFFFFFFFFFFFFFFFFFFFFBBFFFBFFFFFFFFBFFFFF

@GWZHISEQ02:315:C9E6MANXX:5:2215:1907:67565

CGCGGTTACAGAAGCGAGAAGCGAGCTGATTGGTTAGTTTAAATAAGGCTTGGGGTTTTTCCCGGTCTTTTGGGGAACTCGAAACTGAGGTGGGACTTTCCAGAAACTGTTGCTAGTTTCGCTTTA

+

B</B<<FFFFF/FFFFFF7FF/F<FBFFBFFFFFFFFFFF/BFFFFFFFFFFFFF<FFFFF<BFF<FFFFFB/<</FBF7</7B<///<B7<BFBFFFF/B/B/B/FFFFFBF<FFF/7FFFFFFB

@GWZHISEQ02:315:C9E6MANXX:5:1309:2346:45323

AAGCGAAACTAGCAACAGTTTCTGGAAAGTCCCACCTCAGTTTCAAGTTCCCCAAAAGACCGGGAAAAACCCCAAGCCTTATTTAAACTAACCAATCAGCTCGCTTCTCGCTTCTGTAACCGCGCT

+

BBBBBFFFFFFFFFFFFFFFFFFFFFFFFFFFFFFFFFFFFFFFFFFFFFFFFFFFFFFFFFFFFFFFFFFFFFFFFFFFFFFFFFFFFFFFFFFFFFFFBFFFFFFFFFFFFFFFFFFFFFFFF/

@GWZHISEQ02:315:C9E6MANXX:5:2212:8817:72744

AGCGTGGTTACAGAAGCGAGAAGCGAGCTGATTGGTTAGTTTAAATAAGGCTTGGGGTTTTTCCCGGTCTTTTGGGGAACTTGAAACTGAGGTGGGACTTTCCAGAAACTGTTGCTAGTTTCGCTT

+

BBBBB//FFFFFFFFFFFFFFFFFFFFFFFFFFFFFFFFFFFFFFFFFFFFFFFFFF<</FFFFFFFFFFFFFFFFFFFFFFFFFFFFFFFFFFFBFFFFFBFFFFFFFFFFFFFFFFFFFFFFFF

@GWZHISEQ02:315:C9E6MANXX:5:1303:4124:87151

AGCGAAACTAGCAACAGTTTCTGGAAAGTCCCACCTCAGTTTCAAGTTCCCCAAAAGACCGGGAAAAACCCCAAGCCTTATTTAAACTAACCAATCAGCTCGCTTCTCGCTTCTGTAACCGCGCTT

+

BBBBBFFFFFFFFFFFFFFFFFFFFFFFFFFFFFFFFFFFFFFFFFFFFFFFFFFFFFFFFFFFFFFFFFFFFFFFFFFFFFFFFFFFFFFFFFFFFFFFFFFFFFFFFFFFFFFFFFFFFFFFFF

@GWZHISEQ02:315:C9E6MANXX:5:1314:12504:80720

AGCGAAACTAGCAACAGTTTCTGGAAAGTCCCACCTCAGTTTCAAGTTCCCCAAAAGACCGGGAAAAACCCCAAGCCTTATTTAAACTAACCAATCAGCTCGCTTCTCGCTTCTGTAACCGCGCTT

+

BBBBBFFFFFFFFFFFFFFFFFFFFFFFFFFFFFFFFFFFFFFFFFFFFFFFFFFFFFFFFFFFFFFFFFFFFFFFFFFFFFFFFFFFFFFFFFFFFFFFFFFFFFFFFFFFFFFFFFFFFFFFFF

@GWZHISEQ02:315:C9E6MANXX:5:2109:4939:25748

AGCGAAACTAGCAACAGTTTCTGGAAAGTCCCACCTCAGTTTCAAGTTCCCCAAAAGACCGGGAAAAACCCCAAGCCTTATTTAAACTAACCAATCAGCTCGCTTCTCGCTTCTGTAACCGCGCTT

+

BBBBBFFFFFFFFFFFFFFFFFFFFFFFFFFFFFFFFFFFFFFFFFFFFFFFFFFFFFFFFFFFFFFFFFFFFFFFFFFFFFFFFFFFFFFFFFFFFFFFFFFFFFFFFFFFFFFFFFFFFFFFFF

@GWZHISEQ02:315:C9E6MANXX:5:2203:8573:44614

AGCGAAACTAGCAACAGTTTCTGGAAAGTCCCACCTCAGTTTCAAGTTCCCCAAAAGACCGGGAAAAACCCCAAGCCTTATTTAAACTAACCAATCAGCTCGCTTCTCGCTTCTGTAACCGCGCTT

+

BBBB<FFFF/BFFFBFBFFFBFFFBFFFF</BF<<FBFFFFFFFFFFFFFFFFFFFFFFFFFFFB<BBFBFFFFFFFFFFFFFFFFFFFFFFFFFFFFFFFFFFFFFFFFFFBFFFFFFBFF<BFF

@GWZHISEQ02:315:C9E6MANXX:5:2206:14148:99238

AGCGAAACTAGCAACAGTTTCTGGAAAGTCCCACCTCAGTTTCAAGTTCCCCAAAAGACCGGGAAAAACCCCAAGCCTTATTTAAACTAACCAATCAGCTCGCTTCTCGCTTCTGTAACCGAGATC

+

BBBBBFBFB/B/FBFFBF/FFFB//<BF<BBFF/FFF<FFBFFFFFFFFFFBFFFFFFFFFBB/BBFF/7FFFF<FFFFFF</F<FFFFFBFFFFFFBF7F/BBFFFBFFFFFFFBFFF/BBFBFF

@GWZHISEQ02:315:C9E6MANXX:5:2216:9731:95239

AGCGAAACTAGCAACAGTTTCTGGAAAGTCCCACCTCAGTTTCAAGTTCCCCAAAAGACCGGGAAAAACCCCAAGCCTTATTTAAACTAACCAATCAGCTCGCTTCTCGCTTCTGTAACCGCGCTT

+

BBBBBFFFFFFFFFFFFFFFFFFFFFFFFFFFFFFFFFFFFFFFFFFFFFFFFFFFFFFFFFFFFFFFFFFFFBFFFFFFFFFFFFFFFFFFFFFFFFFFFFFFFF<FFFFFFFFFFFFFFFFFFF

@GWZHISEQ02:315:C9E6MANXX:5:2301:15248:50187

AGCGAAACTAGCAACAGTTTCTGGAAAGTCCCACCTCAGTTTCAAGTTCCCCAAAAGACCGGGAAAAACCCCAAGCCTTATTTAAACTAACCAATCAGCTCGCTTCTCGCTTCTGTAACCGCGCTT

+

BBBBBFFFFFFFFFFFFFFFFFFFFFFFFFFFFFFFFFFFFFFFFFFFFFFFFFFFFFFFFFFFFFFFFFFFFFFFFFFFFFFFFFFFFFFFFFFFFFFFFFFFFFFFFFFFFFFFFFFFFFFFBF

@GWZHISEQ02:315:C9E6MANXX:5:2312:9373:50920

AGCGAAACTAGCAACAGTTTCTGGAAAGTCCCACCTCAGTTTCAAGTTCCCCAAAAGACCGGGAAAAACCCCAAGCCTTATTTAAACTAACCAATCAGCTCGCTTCTCGCTTCTGTAACCAGATCG

+

BBBBBFFFFFFFFFFFFFFFFFFFFFFBFFFFFFFFFFFFFFFFFFFFFFFFFFFFFFFFFFFFFFFFFFFFFFFFFFFFFFFFFFFFFFFFFFFFFFFFFFFFFFFFFFFFFFFFBFFFFFFFFF

@GWZHISEQ02:315:C9E6MANXX:5:2206:14148:99238

CGGTTACAGAAGCGAGAAGCGAGCTGATTGGTTAGTTTAAATAAGGCTTGGGGTTTTTCCCGGTCTTTTGGGGAACTTGAAACTGAGGTGGGACTTTCCAGAAACTGTTGCTAGTTTCGCTAGATC

+

BBBBBFFFFFFFFFF<FFFFFFFFFFFB<FF/FFFFFFFFFFFFFFFFFFFFFF<F<<BFFFBBFF<FBF<FFFFFFFFFFFBFF<BFBFFFBFFFFFFFFFFFFFBFBFFFFFFFFFFFFFFFFF

@GWZHISEQ02:315:C9E6MANXX:5:2312:9373:50920

GGTTACAGAAGCGAGAAGCGAGCTGATTGGTTAGTTTAAATAAGGCTTGGGGTTTTTCCCGGTCTTTTGGGGAACTTGAAACTGAGGTGGGACTTTCCAGAAACTGTTGCTAGTTTCGCTAGATCG

+

BBBBBFFF<FFFFFFFFFFFFFFFFFFFFFFFFFFFFBFFFFFFFFFFFFFFFFFFFFFFFBFBBFBFFFFFFFFFFFBFFFFFFFFFFFFFFFFFFFFFFFFFFBFFFFFFFFBFFFFFFF<FFF

@GWZHISEQ02:315:C9E6MANXX:5:1311:19197:13002

GCGAAACTAGCAACAGTTTCTGGAAAGTCCCACCTCAGTTTCAAGTTCCCCAAAAGACCGGGAAAAACCCCAAGCCTTATTTAAACTAACCAATCAGCTCGCTTCTCGCTTCTGTAACCGCGCTTT

+

BBBBBFFFFFFFFFFFFFFFFFFFFFFFFFFFFFFFFFFFFFFFFFFFFFFFFFFFFFFFFFFFFFFFFFFFFFFFFFFFFFFFFFFFFFFFFFFFFFFFFFFFFFFFFFFFFFFFFFFFFFFFFF

@GWZHISEQ02:315:C9E6MANXX:5:2110:15639:63208

GCGAAACTAGCAACAGTTTCTGGAAAGTCCCACCTCAGTTTCAAGTTCCCCAAAAGACCGGGAAAAACCCCAAGCCTTATTTAAACTAACCAATCAGCTCGCTTCTCGCTTCTGTAACCGAGATCG

+

BBBBBFFFFFFFFFFFFFFFFFFFFFFFFFFFFFFFFFFFFFFF<BFFFFFFFFFFFFFFFFFFFFFFFFFFFFFFFFFFFFFFFFFFFFFFFFFFFFFFFFFFFFFFFFFFFFFFFFFFFFFFFF

@GWZHISEQ02:315:C9E6MANXX:5:2113:8262:82861

GCGAAACTAGCAACAGTTTCTGGAAAGTCCCACCTCAGTTTCAAGTTCCCCAAAAGACCGGGAAAACCCCCAAGCCTTATTTAAACTAACCAATCAGCTCGCTTCTCGCTTCTGTAACCGCGCTTT

+

BBB<BBFFFFFFBFFFFFFFFFFFFFFFFFFFFFFFFFFFFFFFFFFFFFFFFFFFFFFFFFFFFF/FFFFFFFFFFFFFFFFFFFFFFFFFFFFFFFFFFFFFFFFFFFFFFFFFFFFFFFFFFF

@GWZHISEQ02:315:C9E6MANXX:5:2204:12933:53140

GCGAAACTAGCAACAGTTTCTGGAAAGTCCCACCTCAGTTTCAAGTTCCCCAAAAGACCGGGAAAAACCCCAAGCCTTATTTAAACTAACCAATCAGCTCGCTTCTCGCTTCTGTAACCGCGCTTT

+

B/BBB<<FBFBFFFFB/BFBFFFFFF/<F/FFFFFFFFFFFFFFFFFFFBBBFFFBBFFFFF<FFFFB<FFFFFFFFF<FFFFFFFFFFFFBFFFFFFFFB<FFBBFFFFFBFFFFFFF</BFFFF

@GWZHISEQ02:315:C9E6MANXX:5:2213:5958:60723

GCGAAACTAGCAACAGTTTCTGGAAAGTCCCACCTCAGTTTCAAGTTCCCCAAAAGACCGGGAAAAACCCCAAGCCTTATTTAAACTAACCAATCAGCTCGCTTCTCGCTTCTGTAACCGCGCTTT

+

BBBBBFFFFFFFFFFFFFFFFFFFFFFFFFFFFFBFFFFFFFFFFFFFFFFFFFFFFFFFFFFFFFFFFFFFFFFFFFFFFFFFFFFFFFFFFFFFFFFFBBFFFFFFFBFFB/B/FFFBFFFFFF

@GWZHISEQ02:315:C9E6MANXX:5:2110:15639:63208

CGGTTACAGAAGCGAGAAGCGAGCTGATTGGTTAGTTTAAATAAGGCTTGGGGTTTTTCCCGGTCTTTTGGGGAACTTGAAACTGAGGTGGGACTTTCCAGAAACTGTTGCTAGTTTCGCAGATCG

+

BBBBBFFFFFFFFFFFFFFFFFFFFFFFFFFFFFFFFFFFFFFFFFFFFFFFFFFFFFFFFFFFFFFFFFFFFFFFFFFFFFFFFFFFFFFFFFFFFFFFFFFFFFFFFFFFFFFFFFFFFFFFFF

@GWZHISEQ02:315:C9E6MANXX:5:2205:20235:89664

CGAAACTAGCAACAGTTTCTGGAAAGTCCCACCTCAGTTTCAAGTTCCCCAAAAGACCGGGAAAAACCCCAAGCCTTATTTAAACTAACCAATCAGCTCGCTTCTCGCTTCTGTAACCGCGCTTTT

+

BBBBBFFFFFFFFFFFFFFFFFFFFFFFFFFFFFFFFFFFFFFFFFFFFFFFFFFFFFFFFFFFFFFFFFFFFFFFFFFFFFFFFFFFFFFFFFFFFFFFFFFFFFFFFFFFFFFFFFFFFFFFFF

@GWZHISEQ02:315:C9E6MANXX:5:2207:12740:65362

CGAAACTAGCAACAGTTTCTGGAAAGTCCCACCTCAGTTTCAAGTTCCCCAAAAGACCGGGAAAAACCCCAAGCCTTATTTAAACTAACCAATCAGCTCGCTTCTCGCTTCTGTAACCGCGCTTTT

+

BBBBBFFFFFFFFFFFFFFFFFFFFFFFFFFFFFFFFFFFFFFFFFFFFFFFFFFFFFFFFFFFFFFFFFFFFFFFFFFFFFFFFFFFFFFFFFFFFFFFFFFFFFFFFFFFFFFFFFFFFFFFFF

@GWZHISEQ02:315:C9E6MANXX:5:2311:18116:32990

CGAAACTAGCAACAGTTTCTGGAAAGTCCCACCTCAGTTTCAAGTTCCCCAAAAGACCGGGAAAAACCCCAAGCCTTATTTAAACTAACCAATCAGCTCGCTTCTCGCTCCTGTAACCGCGCTCTT

+

BBBBBFFFBBBBFBBFBBFFFFFFBFFFFB<FFFBFF<FBF<<FFB/FFFFB/FBB/<7FB/BF/<<FF//<BFFBFBFFFF/FF/<FF7F////<BFF<<//</BB<F/7/<FFFFFFFFFF///

@GWZHISEQ02:315:C9E6MANXX:5:2313:9662:92009

CGAAACTAGCAACAGTTTCTGGAAAGTCCCACCTCAGTTTCAAGTTCCCCAAAAGACCGGGAAAAACCCCAAGCCTTATTTAAACTAACCAATCAGCTCGCTTCTCGCTTCTGTAACCGCGCTTTT

+

BBBBBFFFFFBFFFFFFFFFFFFFFFFFFFFBFFFFFFFFFFFFFFFFFFFFFFFFFFFFFBFFFFFFFFBFFFFFFFFFFFFFFFFFFFFFFFFFFFFFFFFFFFFFBFFFFFFFFFFFFFFFFF

@GWZHISEQ02:315:C9E6MANXX:5:2316:11479:5971

CGAAACTAGCAACAGTTTCTGGAAAGTCCCACCTCAGTTTCAAGTTCCCCAAAAGACCGGGAAAAACCCCAAGCCTTATTTAAACTAACCAATCAGCTCGCTTCTCGCTTCTGTAACCGCGCTTTT

+

BBBBBFFFFFFFFFFFFFFFFBFFFFFFFFFFFFFFFFFFFFFFFFFFFFFFFFFFFFFFFFBFFFFFFFFFFFFFFFFFFFFFFFFFFFFFFFFFFFFFFFFFFFFFFFFFFFFFFFFFFFBFFF

@GWZHISEQ02:315:C9E6MANXX:5:2316:4165:9216

CGAAACTAGCAACAGTTTCTGGAAAGTCCCACCTCAGTTTCAAGTTCCCCAAAAGACCGGGAAAAACCCCAAGCCTTATTTAAACTAACCAATCAGCTCGCTTCTCGCTTCTGTAACCGCGCTTTT

+

BBBBBBFBBFFFF<BFFFFFF<F/FFFBFFFFFFFFFFFFFFFF<FFFFBFFFFFBFFFFFBFF<FBBFFFFB/FFFFFFFFBFFFFFFFFFBFFBFFFFFF/<FFFFFFFBBF<FF/<F<BBFBF

@GWZHISEQ02:315:C9E6MANXX:5:2316:12288:71124

CGAAACTAGCAACAGTTTCTGGAAAGTCCCACCTCAGTTTCAAGTTCCCCAAAAGACCGGGAAAAACCCCAAGCCTTATTTAAACTAACCAATCAGCTCGCTTCTCGCTTCTGTAACCGCGCTTTT

+

BBBBBFFFFFFFFFFFFFFFFFFFFFFFFFFFFFFFFFFFFFFFFFFFFFFFFFFFFFFFFFFFFFFFFFFFFFFFFFFFFFFFFFFFFFFFFFFFFFFFFFFFFFFFFFFFFFFFFFFFFFFFFF

@GWZHISEQ02:315:C9E6MANXX:5:1104:9676:97510

GAAACTAGCAACAGTTTCTGGAAAGTCCCACCTCAGTTTCAAGTTCCCCAAAAGACCGGGAAAAACCCCAAGCCTTATTTAAACTAACCAATCAGCTCGCTTCTCGCTTCTGTAACCGCGCTTTTT

+

BBBBBFFFFFFFFFFFFFFFFFFFFFFFFFFFFFFFFFFFFFFFFFFFFFFFFFFFFFFFFFFFFFFFFFFFFFFFFFFFFFFFFFFFFFFFFFFFFFFFFFFFFFFFFFFFFFFFFFFFFFFFFF

@GWZHISEQ02:315:C9E6MANXX:5:2111:16461:74408

GCAAAAAGCGCGGTTACAGAAGCGAGAAGCGAGCTGATTGGTTAGTTTAAATAAGGCTTGGGGTTTTTCCCGGTCTTTTGGGGAACTTGAAACTGAGGTGGGACTTTCCAGAAACTGTTGCTAGTT

+

<BBBBFFFFFFFFFFFFFFFFFFFFFFFFFFFFBFFFFFFFFFFFFFFFFFFBFFFFFFFFFFFFF<FBFFBFFBFFFFFFFFFFFFFFFFFFFFF<FFFFFFF<FFFBFFFFBFFFFFFFBFFFF

@GWZHISEQ02:315:C9E6MANXX:5:2114:20641:54068

GCAAAAAGCGCGGTTACAGAAGCGAGAAGCGAGCTGATTGGTTAGTTTAAATAAGGCTTGGGGTTTTTCCCGGTCTTTTGGGGAACTTGAAACTGAGGTGGGACTTTCCAGAAACTGTTGCTAGTT

+

BBBBBFFFFFFFFFFFFFFFFFFFFFFFFFFFFBFF<FFFFFFFFFFFFFFFFFFFFFFFFFFFFFFFFFF<BFFFBFFBBFFFFFBFF/FFFFFFFFFFFFFFFFFFFFFFFFFBFFFBFF/B7F

@GWZHISEQ02:315:C9E6MANXX:5:1205:6327:43703

AGCAAAAAGCGCGGTTACAGAAGCGAGAAGCGAGCTGATTGGTTAGTTTAAATAAGGCTTGGGGTTTTTCCCGGTCTTTTGGGGAACTTGAAACTGAGGTGGGACTTTCCAGAAACTGTTGCTAGT

+

BBBBBFFFFFFFFFFFFFFFFFFFFFFFFFFFFFFFFFFFFFFFFFFFFFFFFFFFFFFFFFFF<BFFFFFFFFFFFFFFFFFFFFFFFFFF<FFFF<FFFFFFFFFFFFFFFFFFFFFFFFFFFB

@GWZHISEQ02:315:C9E6MANXX:5:1212:10726:37297

AGCAAAAAGCGCGGTTACAGAAGCGAGAAGCGAGCTGATTGGTTAGTTTAAATAAGGCTTGGGGTTTTTCCCGGTCTTTTGGGGAACTTGAAACTGAGGTGGGACTTTCCAGAAACTGTTGCTAGT

+

BBBBBFFFFFFFFFFFFFFFFFFFFFFFFFFF/BFFFFFFFFFFFFFBFFFFFFBFFF<<FFFF<FFFFFFFFFFFFFFFFFFFFFFFFFFFFFFFFFFFFFFFFBFFFFFFFBFFFFFFFFFFFF

@GWZHISEQ02:315:C9E6MANXX:5:1110:18575:75772

CTAGCAACAGTTTCTGGAAAGTCCCACCTCAGTTTCAAGTTCCCCAAAAGACCGGGAAAAACCCCAAGCCTTATTTAAACTAACCAATCAGCTCGCTTCTCGCTTCTGTAACCGCGCTTTTTGCTC

+

BBBBBF/B//<<FBFBFFFFBFFFFFFBFFFF<FB/<FFF//FFFF<<FF<BBFFB/F<FFFFFFBBBBFFFFFF<FFBBFFFFFBBBF7B//F<FF7FFFFFFFFFFFBFFB7B7BBBFFBFFFF

@GWZHISEQ02:315:C9E6MANXX:5:2306:14045:10719

CTAGCAACAGTTTCTGGAAAGTCCCACCTCAGTTTCAAGTTCCCCAAAAGACCGGGAAAAACCCCAAGCCTTATTTAAACTAACCAATCAGCTCGCTTCTCGCTTCTGTAACCGCGCTTTTTGCTC

+
[truncated: 16,747,637 more chars]
