# Supplementary material for: Characterization of a new case of XMLV (Bxv1) contamination in the human cell line Hep2 (clone 2B)
Source: Sci Rep. 2020 Sep 29;10:16046. doi: 10.1038/s41598-020-73169-y (PMC7524804; doi:10.1038/s41598-020-73169-y)
Supplement: Supplementary file 3 — Supplementary Information 3. [file 41598_2020_73169_MOESM3_ESM.docx]

>1-hg19_dnarange=chr12:132414659-1324148595'pad=03'pad=0strand=+repeatMasking=none

AGGAGCTCGCGTGCAGCAGGAGCAGTGAAAGCTGCTTGGGCCTGCACCTAAATCCTGCCG

GACCCGCCGCTACCTGGGCGACCGCTCCTGCACCTAAACC--------------------

------------------------------------------------------------

------------------------------------------------------------

------------------------------------------------------------

------------------------------------------------------------

------------------------------------------------------------

------------------------------------------------------------

------------------------------------------------------------

------------------------------------------------------------

------------------------------------------------------------

------------------------------------------------------------

------------------------------------------------------------

------------------------------------------------------------

------------------------------------------------------------

------------------------------------------------------------

------------------------------------------------------------

------------------------------------------------------------

------------------------------------------------------------

------------------------------------------------------------

------------------------------------------------------------

------------------------------------------------------------

------------------------------------------------------------

------------------------------------------------------------

------------------------------------------------------------

------------------------------------------------------------

------------------------------------------------------------

------------------------------------------------------------

------------------------------------------------------------

------------------------------------------------------------

------------------------------------------------------------

------------------------------------------------------------

------------------------------------------------------------

------------------------------------------------------------

------------------------------------------------------------

------------------------------------------------------------

------------------------------------------------------------

------------------------------------------------------------

------------------------------------------------------------

------------------------------------------------------------

------------------------------------------------------------

------------------------------------------------------------

------------------------------------------------------------

------------------------------------------------------------

------------------------------------------------------------

------------------------------------------------------------

------------------------------------------------------------

------------------------------------------------------------

------------------------------------------------------------

------------------------------------------------------------

------------------------------------------------------------

------------------------------------------------------------

------------------------------------------------------------

------------------------------------------------------------

------------------------------------------------------------

------------------------------------------------------------

------------------------------------------------------------

------------------------------------------------------------

------------------------------------------------------------

------------------------------------------------------------

------------------------------------------------------------

------------------------------------------------------------

------------------------------------------------------------

------------------------------------------------------------

------------------------------------------------------------

------------------------------------------------------------

------------------------------------------------------------

------------------------------------------------------------

------------------------------------------------------------

------------------------------------------------------------

------------------------------------------------------------

------------------------------------------------------------

------------------------------------------------------------

------------------------------------------------------------

------------------------------------------------------------

------------------------------------------------------------

------------------------------------------------------------

------------------------------------------------------------

------------------------------------------------------------

------------------------------------------------------------

------------------------------------------------------------

------------------------------------------------------------

------------------------------------------------------------

------------------------------------------------------------

------------------------------------------------------------

------------------------------------------------------------

------------------------------------------------------------

------------------------------------------------------------

------------------------------------------------------------

------------------------------------------------------------

------------------------------------------------------------

------------------------------------------------------------

------------------------------------------------------------

------------------------------------------------------------

------------------------------------------------------------

------------------------------------------------------------

------------------------------------------------------------

------------------------------------------------------------

------------------------------------------------------------

------------------------------------------------------------

------------------------------------------------------------

------------------------------------------------------------

------------------------------------------------------------

------------------------------------------------------------

------------------------------------------------------------

------------------------------------------------------------

------------------------------------------------------------

------------------------------------------------------------

------------------------------------------------------------

------------------------------------------------------------

------------------------------------------------------------

------------------------------------------------------------

------------------------------------------------------------

------------------------------------------------------------

------------------------------------------------------------

------------------------------------------------------------

------------------------------------------------------------

------------------------------------------------------------

------------------------------------------------------------

------------------------------------------------------------

------------------------------------------------------------

------------------------------------------------------------

------------------------------------------------------------

------------------------------------------------------------

------------------------------------------------------------

------------------------------------------------------------

------------------------------------------------------------

------------------------------------------------------------

------------------------------------------------------------

------------------------------------------------------------

------------------------------------------------------------

------------------------------------------------------------

------------------------------------------------------------

------------------------------------------------------------

------------------------------------------------------------

------------------------------------------------------------

------------------------------------------------------------

------------------------------------------------------------

------------------------------------------------------------

------------------------------------------------------------

------------------------------------------------------------

------------------------------------------------------------

------------------------------------------------------------

------------------------------------------------------------

------------------------------------------------------------

------------------------------------------------------------

-CAGCCGGACCCACCGCTTCCCCGGCGGCCGCACCTGCACCTAAACCCCGCGGGACCTGC

CGCTTCCCGGGCGGCCACACCTGCATGCCGTGGTAGCCCTTG

>2-JF908815.1XenotropicmurineleukemiavirusisolateVCaP,completegenome

------------------------------------------------------------

----------------------------------------TGAAAGACCCCACCATAAGG

CTTAGCAAGCTAGCTGCAGTAACGCCATTTTGCAAGGCATGAAAAAGTACCAGAGCTGAG

TTCTCAAAAGTCACAAGGAAGTTTAGTTAAAGAATAAGGCTGAACAAAACTGGGACAGGG

GCCAAACAGGATATCTGTGGTCGAGCACCTGGGCCCCGGCTCAGGGCCAAGAACAGATGG

TACTCAGATAAAGCGAAACTAGCAACAGTTTCTGGAAAGTCCCACCTCAGTTTCAAGTTC

CCCAAAAGACCGGGAAAAACCCCAAGCCTTATTTAAACTAACCAATCAGCTCGCTTCTCG

CTTCTGTAACCGCGCTTTTTGCTCCCCAGCCCTATAAAAAGGGTAAAAACCCCACACTCG

GTGCGCCAGTCATCCGATAGACTGAGTCGCCCGGGTACCCGTGTTCCCAATAAAGCCTTT

TGCTGTTTGCATCCGAAACGTGGCCTCGCTGTTCCTTGGGAGGGTCTCCTCAGAGTGATT

GACTACCCAGCTCGGGGGTCTTTCATTTGGGGGCTCGTCCGGGATTTGGAGACCCCCGCC

CAGGGACCACCGACCCACCGTCGGGAGGTAAGCTGGCCAGCGATCGTTTTGTCTCCGTCT

CTGTCTTTGTGCGTGTGTGTGTGTGCCGGCATCTACTTTTTGCGCCTGCGTCTGAATCTG

TACTAGTTAGCTAACTAGATCTGTATCTGGCGGTTCCGTGGAAGAACTGACGAGTTCGTA

TTCCCGACCGCAGCCCTGGGAGACGTCTCAGAGGCATCAGGGGCCCGCTGGGTGGCCCAA

TCAGTAAGTCCGAGTCCTGACCGATTCGGACTATTTGGAGCCCCTCCTTTGTCGGAGGGG

TACGTGGTTCTTTTAGGAGACGAGAGGTCCAAGCCCTCGCCGCCTCCATCTGAATTTTTG

CTTTCGGTTTTTCGCCGAAACCGCGCCGCGCGTCTTGTCTGTCTCAGTGTTGTTTTGTCA

TTTGTCTGTTCGTTATTGTTTTGGACCGTTTCTAAAAATATGGGACAGACCGTAACCACC

CCTCTGAGTCTGACCCTAGAACACTGGGGAGACGTCCAGCGCATCGCGTCCAACCAGTCC

GTGGACGTCAAGAAGAGACGCTGGGTCACCTTCTGCTCTGCCGAGTGGCCAACTTTCGGT

GTAGGGTGGCCGCAAGATGGTACTTTTAATTTGGACATTATTTTACAGGTTAAATCTAAG

GTGTTCTCTCCCGGTCCCCACGGACACCCGGATCAGGTCCCATACATTGTCACCTGGGAG

GCTATTGCCTATGAACCCCCTCCGTGGGTCAAACCTTTTGTCTCTCCCAAACTCTCCCTC

TCTCCAACCGCTCCCATCCTCCCATCCGGTCCTTCGACCCAACCTCCGCCCCGATCTGCC

CTTTACCCTGCTCTTACCCCCTCTATAAAACCCAGACCTTCTAAACCTCAGGTTCTCTCC

GATAATGGCGGACCTCTCATTGACCTTCTCACAGAAGACCCTCCGCCGTACGGAGAACAG

GGACCGTCCTCCTCTGACGGAGATGGCGACAGAGAAGAGGCCACCTCCACTCCTGAGATT

CCTGCCCCCTCTCCCATGGTGTCTCGCTTGCGGGGCAAAAGAGACCCCCCCGCGGCAGTT

TCCACCACCTCTCGGGCTTTCCCACTCCGTTTGGGGGGTAATGGTCAGTTGCAGTACTGG

CCGTTTTCCTCCTCGGATCTATATAACTGGAAAAATAATAACCCTTCCTTCTCTGAAGAT

CCAGGTAAATTGACTGCCTTAATCGAGTCTGTCCTCACCACCCACCAGCCTACTTGGGAT

GACTGTCAACAGTTGCTGGGGACTCTGCTGACAGGAGAAGAAAAGCAGCGGGTGCTCCTG

GAAGCCAGAAAGGCAGTCCGGGGCGACGATGGCCGCCCCACCCAATTGCCCAATGAGATC

GAGGCTGCCTTTCCCCTCGAACGTCCCGACTGGGACTACACCACCCTTAGAGGTAGGAAC

CACCTAGTTCTCTATCGCCAGCTGCTCTTGGCGGGTCTCCAAAATGCGGGCAGGAGCCCC

ACCAATTTGGCTAAGGTAAAAGGAATAACCCAGGGGTCCAACGAGTCGCCCTCGGCCTTT

CTAGAGAGACTCAAAGAGGCCTATCGCAGATACACTCCTTATGACCCTGAGGACCCTGGG

CAAGAAACCAATGTATCCATGTCGTTCATCTGGCAGTCTGCTCCAGACATTGGTCGAAAG

TTAGAGCGGTTAGAAGACTTAAAAAATAAGACCTTAGGGGACTTAGTGAGAGAAGCAGAA

AGGATCTTTAATAAGAGAGAGACCCCAGAAGAGAGAGAAGAACGTATTAAGAGAGAAACA

GAGGAAAAAGAGGAGCGCCGTAGGGCAGAGGATGAGCAGAAAGAGAAAGAGAGGGACCGC

AGAAGACAGAGAGAAATGAGCAAACTCTTGGCCACCGTAGTTACAGGTCAGAGACAGGAT

AGACAGGGGGGAGAGCGAAGGAGGCCCCAACTCGATAAGGACCAATGCGCCTACTGCAAA

GAAAAGGGACACTGGGCTAGGGATTGCCCAAAGAAGCCACGGGGGCCCCGAGGACCGAGG

CCCCAGACCTCCCTCCTGACCCTAGATGACTAGGGAGGTCAGGGTCAGGAGCCCCCCCCT

GAACCCAGGATAACCCTTACTGTCGGGGGGCAACCAGTCACCTTCCTGGTGGATACTGGG

GCCCAACACTCCGTGCTGACCCAGAACCCTGGACCCCTAAGTGACAGGTCTGCCTGGGTC

CAAGGGGCTACTGGAGGAAAGCGGTATCACTGGACCACAGATCGCAAGGTGCACCTGGCT

ACCGGTAAGGTCACTCACTCTTTCCTCCATGTGCCGGACTGCCCTTATCCTTTGCTAGGA

AGGGACTTGTTGACTAAGTTAAAGGCCCAGATCCACTTCGAGGGATCGGGAGCTCAGGTT

GTGGGACCAAAAGGACAGCCCCTGCAGGTGTTGACCCTTGGCATAGAGGATGAGTATCGG

CTACATGAGACCTCAACAGAGCCGGATGTTTCTCTAGGGTCCACCTGGCTTTCTGACTTT

CCCCAGGCCTGGGCAGAAACCGGGGGCATGGGACTGGCAGTTCGCCAAGCGCCTCTGATT

ATACCTCTAAAGGCAACCTCCACCCCTGTGTCCATCAAACAGTACCCCATGTCACACGAA

GCCAGACTGGGGATCAAGCCCCACATACAGAGACTGTTGGACCAGGGAATATTGGTACCT

TGCCAGTCCCCCTGGAACACACCCCTGCTGCCCGTTAAGAAACCAGGGACTAATGATTAC

AGGCCTGTCCAGGATCTGAGAGAAGTCAACAAGCGGGTGGAAGATATCCACCCCACCGTG

CCCAATCCTTACAACCTCTTAAGTGGACTCCCTCCGTCCCACCAGTGGTACACTGTGCTT

GATTTAAAAGATGCCTTTTTCTGCCTGAGACTCCACCCCACCAGTCAGCCTCTCTTTGCC

TTTGAGTGGAGAGATCCAGAAATGGGAATCTCTGGACAATTGACCTGGACCAGACTCCCA

CAGGGTTTCAAAAACAGTCCCACCCTGTTTGATGAGGCATTGCACAGAGACCTAGCAGAC

TTCCGGATCCAGCACCCAGACTTGATCCTGCTACAGTACGTGGATGACTTACTGCTGGCC

GCTACTTCCGAACTAGACTGCCAACAAGGTACTCGGGCCCTTCTACAAACCCTAGGGGAC

CTCGGATACCGGGCCTCGGCCAAGAAAGCCCAAATCTGCCAGAAACAGGTTAAATACCTG

GGGTACCTTCTGAGGGAGGGTCAGAGATGGCTGACTGAGGCTAGAAAAGAGACTGTGATG

GGGCAACCCGTTCCAAAGACTCCTCGACAACTAAGGGAGTTCCTAGGGACGGCAGGCTTC

TGCCGCCTCTGGATCCCTGGGTTTGCGGAAATGGCGGCCCCCTTGTATCCTCTTACCAAA

ACGGGGACTCTGTTTAATTGGGGCCCAGACCAGCAAAAGGCCTATCAAGAAATCAAACAG

GCCCTTCTAACTGCCCCCGCCCTGGGATTGCCAGATTTGACTAAGCCCTTTGAACTCTTT

GTCGACGAGAAGCAGGGCTACGCCAAAGGCGTCCTAACGCAAAAACTGGGACCTTGGCGT

CGGCCTGTGGCCTACCTGTCCAAAAAGCTAGACCCAGTGGCAGCCGGGTGGCCCCCTTGC

CTACGGATGGTAGCAGCCATTGCCGTTCTGACAAAAGATGCAGGCAAGCTAACTATGGGA

CAGCCGCTAGTCATCCTGGCCCCCCATGCAGTAGAGGCACTGGTCAAGCAACCCCCTGAC

CGCTGGCTATCCAACGCCCGCATGACCCACTACCAGGCAATGCTCCTAGACACTGACCGA

GTTCAGTTCGGACCAGTGGTGGCCCTCAATCCTGCCACCTTGCTCCCTCTACCGGAAAAA

GGAGCCCCCCATGATTGCCTCGAGATCTTGGCTGAAACGCATGGAACCAGACCGGATCTC

ACCGACCAGCCCATCCCAGACGCCGACCACACCTGGTATACCGATGGGAGCAGCTTTCTG

CAAGAAGGACAGCGAAAGGCTGGGGCAGCAGTGACGACTGAAACCGAGGTAATCTGGGCG

AGGGCCCTGCCAGCTGGAACGTCAGCCCAGCGAGCCGAACTGATCGCACTCACCCAAGCC

CTGAAAATGGCAGAAGGTAAGAAGCTAAATGTTTACACTGATAGCCGCTATGCCTTCGCT

ACGGCCCATGTTCATGGGGAAATATATAGGAGACGGGGGTTGCTGACCTCAGAAGGCAAG

GAAATCAAGAACAAAAGCGAGATCCTAGCCTTGCTGAAAGCCCTCTTTTTGCCAAAGAGA

CTCAGTATTATCCATTGCCCAGGACATCAGAAAGGAGACAGTGCCGAAGCCAGAGGCAAC

CGTATGGCAGACCAGGCGGCCCGAGAGGCAGCCACAAAAACAGTTCCAGAAGCCTCTACA

CTCCTTATAGAGGACTCGACCCCGTACACGCCTGCCTATCTCCATTACACCGAAACAGAT

CTAAAAAGATTGCGAGAACTGGGGGCCACCTATAATCAGATAAAAGGATATTGGGTCCTA

CAAGGCAAGCCGGTGATGCCCGATCAGTTTGTGTTTGAATTATTAGACTCCCTTCATAGA

CTCACCCATCTCAGCCCTCAAAAGATGAAGGCGCTCCTTGACAGAGAAGAAAGCCCCTAC

TACATGTTAAACAGGGACAGAACTCTTCAGTATGTGGCAGAATCCTGCACAGTCTGTGCT

CAAGTAAATGCTAGTAAAGCCAAAATCGGGGCAGGGGTACGAGTACGCGGACATCGACCA

GGTACCCATTGGGAAATTGACTTCACTGAAGTTAAACCAGGGCTGTACGGGTACAAGTAC

CTCCTGGTGTTCGTAGACACCTTCTCTGGCTGGGTGGAAGCCTTCCCAACTAAACGTGAA

ACTGCCAAGGTTGTGACCAAGAAGCTATTAGAAGAAATATTCCCAAGATTCGGGATGCCA

CAGGTATTGGGTTCCGATAATGGGCCTGCCTTCGTCTCCCAGGTAAGTCAGTCGGTGGCC

GATTTACTGGGGATCGATTGGAAATTACATTGTGCTTATAGACCCCAGAGTTCAGGTCAG

GTAGAAAGAATGAATAGAACCATCAAGGAGACTCTAACTAAATTAACGCTTGCAGCTGGC

ACTAGAGACTGGGTACTCCTACTCCCCTTAGCCCTCTACCGAGCCCGGAACACTCCGGGC

CCCCATGGACTGACTCCGTATGAAATTCTGTATGGGGCACCCCCGCCCCTTGTCAATTTT

CATGATCCTGAAATGTCAAAGTTAACTAATAGTCCCTCTCTCCAAGCTCACTTACAGGCC

CTCCAAGCAGTACAACGAGAGGTCTGGAAGCCGCTGGCCGCTGCTTATCAGGACCAGCTA

GATCAGCCAGTGATACCACACCCCTTCCGTGTCGGTGACGCCGTGTGGGTACGCCGGCAC

CAGACTAAGAACTTGGAACCTCGCTGGAAAGGACCCTACACCGTCCTGCTGACCACCCCC

ACCGCTCTCAAAGTTGACGGCATCTCTGCGTGGATACACGCCGCTCACGTAAAGGCGGCG

ACAACTCCTCCGGCCGGAGCAGCATGGAAGGTCCAGCGTTCTCAAAACCCCTTAAAGATA

AGATTAACCCGTGGGGCCCCCTAATAGTTATAGGGATCTTGGTGAGGGCAGGAGCCTCGG

TACAACGTGACAGCCCTCACCAGGTCTTCAATGTCACTTGGAGAGTTACCAACCTAATGA

CAGGACAAACAGCTAACGCTACCTCCCTCCTGGGGACGATGACAGACACCTTCCCTAAAC

TATATTTTGACTTGTGTGATTTAGTTGGAGACCATTGGGATGACCCAGAACCCGATATTG

GAGATGGTTGCCGCTCTCCGGGGGGAAGAAAAAGGACAAGACTGTATGACTTCTATGTTT

GCCCCGGTCATACTGTACCAATAGGGTGTGGAGGGCCGGGAGAGGGCTACTGTGGCAAAT

GGGGATGTGAGACCACTGGACAGGCATACTGGAAGCCATCATCATCATGGGACCTAATTT

CCCTTAAGCGAGGAAACACTCCTAAGGATCAGGGCCCCTGTTATGATTCCTCGGTCTCCA

GTGGCGTCCAGGGTGCCACACCGGGGGGTCGATGCAACCCCCTAGTCTTAGAATTCACTG

ACGCGGGTAAAAAGGCCAGCTGGGATGCCCCCAAAGTTTGGGGACTAAGACTCTACCGAT

CCACGGGGGCCGACCCGGTGACCCGGTTCTCTTTGACCCGCCAGGTCCTCAATGTAGGAC

CCCGCGTCCCCATTGGGCCTAATCCCGTGATCACTGAACAGCTACCCCCCTCCCAACCCG

TGCAGATCATGCTCCCCAGGCCTCCTCATCCTCCTCCTTCAGGCGCGGCCTCTATGGTGC

CTGGGGCTCCCCCGCCTTCTCAACAACCTGGGACGGGGGACAGGCTGCTAAACCTAGTAA

AAGGAGCCTATCAAGCACTCAACCTCACCAGTCCCGACAGAACCCAAGAGTGCTGGCTGT

GTCTGGTATCGGGACCCCCCTACTACGAAGGGGTTGCCGTCCTAGGTACCTACTCCAACC

ATACCTCTGCCCCAGCTAACTGCTCCGTGGCCTCCCAACACAAGCTGACCCTGTCCGAAG

TGACCGGGCAGGGACTCTGCGTAGGAGCAGTTCCCAAAACCCATCAGGCCCTGTGTAATA

CCACCCAGAAGGCGAGCGACGGGTCCTACTATCTGGCTGCTCCCGCCGGGACCATCTGGG

CTTGCAACACCGGGCTCACTCCCTGCCTATCTACCACTGTACTCAACCTCACCACCGATT

ACTGTGTCCTGGTTGAGCTCTGGCCAAAGGTGACCTACCACTCCCCTGGTTATGTTTATG

ACCAGTTTGAGAGAAAAACCAAATATAAAAGAGAGCCGGTGTCATTAACTCTGGCCCTGC

TGTTGGGAGGACTTACTATGGGCGGCATAGCTGCAGGAGTAGGAACAGGGACTACAGCCC

TAGTGGCCACCAAACAATTCGAGCAGCTCCAGGCAGCCATACATACAGACCTTGGGGCCT

TAGAAAAATCAGTCAGTGCCCTAGAAAAGTCTCTGACCTCGTTGTCTGAGGTGGTCCTAC

AGAACCGGAGAGGATTAGATCTGCTGTTCCTAAAAGAAGGAGGATTATGTGCTGCCCTAA

AAGAAGAATGCTGTTTCTATGCAGACCACACTGGCGTAGTAAGGGATAGCATGGCTAAGC

TAAGAGAAAGGCTAAACCAGAGGCAAAAATTGTTCGAATCAGGACAAGGGTGGTTTGAGG

GACTGTTTAACAGGTCCCCATGGTTCACGACCCTGATATCCACCATTATGGGCCCTCTGA

TAGTACTTTTATTAATCCTACTCCTCGGACCCTGCATTCTCAACCGCTTGGTCCAGTTTG

TAAAAGACAGAATTTCGGTGGTGCAGGCCCTGGTTCTGACCCAACAGTATCACCAACTCA

AATCAATAGATCCAGAAGAAGTAGAATCGCGTGAATAAAAGATTTTATTCAGTTTCCAGA

AAGAGGGGGGAATGAAAGACCCCACCATAAGGCTTAGCAAGCTAGCTGCAGTAACGCCAT

TTTGCAAGGCATGAAAAAGTACCAGAGCTGAGTTCTCAAAAGTCACAAGGAAGTTTAGTT

AAAGAATAAGGCTGAACAAAACTGGGACAGGGGCCAAACAGGATATCTGTGGTCGAGCAC

CTGGGCCCCGGCTCAGGGCCAAGAACAGATGGTACTCAGATAAAGCGAAACTAGCAACAG

TTTCTGGAAAGTCCCACCTCAGTTTCAAGTTCCCCAAAAGACCGGGAAAAACCCCAAGCC

TTATTTAAACTAACCAATCAGCTCGCTTCTCGCTTCTGTAACCGCGCTTTTTGCTCCCCA

GCCCTATAAAAAGGGTAAAAACCCCACACTCGGTGCGCCAGTCATCCGATAGACTGAGTC

GCCCGGGTACCCGTGTTCCCAATAAAGCCTTTTGCTGTTTGCATCCGAAACGTGGCCTCG

CTGTTCCTTGGGAGGGTCTCCTCAGAGTGATTGACTACCCAGCTCGGGGGTCTTTCA

>3-GWZHISEQ02:315:C9E6MANXX:5:2305:13740:62280 2:N:0:GAGTGG

--------------------------------------------------------GCCG

GACCCGCCGCTACCTGGGCGACCGCTCCTGCACCTAAACCTGAAAGACCCCACCATAAGG

CTTAGCAAGCTAGCTGCAGTAACGCCATTTTGCAAGGCATGAAAAAGTACCAGAGCTGAG

TT

>4-GWZHISEQ02:315:C9E6MANXX:5:1204:5121:8932 1:N:0:GAGTGG

---------------------------------------------CCCTAAATCCTGCCG

GACCCGCCGCTACCTGGGCGACCGCTCCTGCACCTAAACCTGAAAGACCCCACCATAAGG

CTTAGCAAGCTAGCTGCAGTAACGCCATTTTGCAAGGCATGAAAAAGTACC

>5-GWZHISEQ02:315:C9E6MANXX:5:1108:9651:16292 2:N:0:GAGTGG

----------------------------------------------------------CG

GACCCGCCGCTACCTGGGCGACCGCTCCTGCACCTAAACCTGAAAGACCCCACCATAAGG

CTTAGCAAGCTAGCTGCAGTAACGCCATTTTGCAAGGCATGAAAAAGTACCAGAGCTGAG

TTCT

>6-GWZHISEQ02:315:C9E6MANXX:5:2314:5533:122892:N:0:GAGTGG

------------------------------------------------------------

------------------------------------------------------------

------------------------------------------------------------

------------------------------------------------------------

------------------------------------------------------------

------------------------------------------------------------

------------------------------------------------------------

------------------------------------------------------------

------------------------------------------------------------

------------------------------------------------------------

------------------------------------------------------------

------------------------------------------------------------

------------------------------------------------------------

------------------------------------------------------------

------------------------------------------------------------

------------------------------------------------------------

------------------------------------------------------------

------------------------------------------------------------

------------------------------------------------------------

------------------------------------------------------------

------------------------------------------------------------

------------------------------------------------------------

------------------------------------------------------------

------------------------------------------------------------

------------------------------------------------------------

------------------------------------------------------------

------------------------------------------------------------

------------------------------------------------------------

------------------------------------------------------------

------------------------------------------------------------

------------------------------------------------------------

------------------------------------------------------------

------------------------------------------------------------

------------------------------------------------------------

------------------------------------------------------------

------------------------------------------------------------

------------------------------------------------------------

------------------------------------------------------------

------------------------------------------------------------

------------------------------------------------------------

------------------------------------------------------------

------------------------------------------------------------

------------------------------------------------------------

------------------------------------------------------------

------------------------------------------------------------

------------------------------------------------------------

------------------------------------------------------------

------------------------------------------------------------

------------------------------------------------------------

------------------------------------------------------------

------------------------------------------------------------

------------------------------------------------------------

------------------------------------------------------------

------------------------------------------------------------

------------------------------------------------------------

------------------------------------------------------------

------------------------------------------------------------

------------------------------------------------------------

------------------------------------------------------------

------------------------------------------------------------

------------------------------------------------------------

------------------------------------------------------------

------------------------------------------------------------

------------------------------------------------------------

------------------------------------------------------------

------------------------------------------------------------

------------------------------------------------------------

------------------------------------------------------------

------------------------------------------------------------

------------------------------------------------------------

------------------------------------------------------------

------------------------------------------------------------

------------------------------------------------------------

------------------------------------------------------------

------------------------------------------------------------

------------------------------------------------------------

------------------------------------------------------------

------------------------------------------------------------

------------------------------------------------------------

------------------------------------------------------------

------------------------------------------------------------

------------------------------------------------------------

------------------------------------------------------------

------------------------------------------------------------

------------------------------------------------------------

------------------------------------------------------------

------------------------------------------------------------

------------------------------------------------------------

------------------------------------------------------------

------------------------------------------------------------

------------------------------------------------------------

------------------------------------------------------------

------------------------------------------------------------

------------------------------------------------------------

------------------------------------------------------------

------------------------------------------------------------

------------------------------------------------------------

------------------------------------------------------------

------------------------------------------------------------

------------------------------------------------------------

------------------------------------------------------------

------------------------------------------------------------

------------------------------------------------------------

------------------------------------------------------------

------------------------------------------------------------

------------------------------------------------------------

------------------------------------------------------------

------------------------------------------------------------

------------------------------------------------------------

------------------------------------------------------------

------------------------------------------------------------

------------------------------------------------------------

------------------------------------------------------------

------------------------------------------------------------

------------------------------------------------------------

------------------------------------------------------------

------------------------------------------------------------

------------------------------------------------------------

------------------------------------------------------------

------------------------------------------------------------

------------------------------------------------------------

------------------------------------------------------------

------------------------------------------------------------

------------------------------------------------------------

------------------------------------------------------------

------------------------------------------------------------

------------------------------------------------------------

------------------------------------------------------------

------------------------------------------------------------

------------------------------------------------------------

------------------------------------------------------------

------------------------------------------------------------

------------------------------------------------------------

------------------------------------------------------------

------------------------------------------------------------

------------------------------------------------------------

------------------------------------------------------------

------------------------------------------------------------

------------------------------------------------------------

------------------------------------------------------------

------------------------------------------------------------

------------------------------------------------------------

------------------------------------------------------------

------------------------------------------------------------

------------------------------------------------------------

------CTTGGGAGGGTCTCCTCAGAGTGATTGACTACCCAGCTCGGGGGTCTTTCAAAC

CCAGCCGGACCCACCGCTTCCCCGGCGGCCGCACCTGCACCTAAACCCCGCGGGACCTGC

CGCTTCCCGGGC--------------------
