# Supplementary material for: Characterization of a new case of XMLV (Bxv1) contamination in the human cell line Hep2 (clone 2B)
Source: Sci Rep. 2020 Sep 29;10:16046. doi: 10.1038/s41598-020-73169-y (PMC7524804; doi:10.1038/s41598-020-73169-y)
Supplement: Supplementary file 4 — Supplementary Information 4. [file 41598_2020_73169_MOESM4_ESM.docx]

>gi|336462515|gb|JF908815.1|_Xenotropic_murine_leukemia_virus_isolate_VCaP

TGAAAGACCCCACCATAAGGCTTAGCAAGCTAGCTGCAGTAACGCCATTTTGCAAGGCAT

GAAAAAGTACCAGAGCTGAGTTCTCAAAAGTCACAAGGAAGTTTAGTTAAAGAATAAGGC

TGAACAAAACTGGGACAGGGGCCAAACAGGATATCTGTGGTCGAGCACCTGGGCCCCGGC

TCAGGGCCAAGAACAGATGGTACTCAGATAAAGCGAAACTAGCAACAGTTTCTGGAAAGT

CCCACCTCAGTTTCAAGTTCCCCAAAAGACCGGGAAAAACCCCAAGCCTTATTTAAACTA

ACCAATCAGCTCGCTTCTCGCTTCTGTAACCGCGCTTTTTGCTCCCCAGCCCTATAAAAA

GGGTAAAAACCCCACACTCGGTGCGCCAGTCATCCGATAGACTGAGTCGCCCGGGTACCC

GTGTTCCCAATAAAGCCTTTTGCTGTTTGCATCCGAAACGTGGCCTCGCTGTTCCTTGGG

AGGGTCTCCTCAGAGTGATTGACTACCCAGCTCGGGGGTCTTTCATTTGGGGGCTCGTCC

GGGATTTGGAGACCCCCGCCCAGGGACCACCGACCCACCGTCGGGAGGTAAGCTGGCCAG

CGATCGTTTTGTCTCCGTCTCTGTCTTTGTGCGTGTGTGTGTGTGCCGGCATCTACTTTT

TGCGCCTGCGTCTGAATCTGTACTAGTTAGCTAACTAGATCTGTATCTGGCGGTTCCGTG

GAAGAACTGACGAGTTCGTATTCCCGACCGCAGCCCTGGGAGACGTCTCAGAGGCATCAG

GGGCCCGCTGGGTGGCCCAATCAGTAAGTCCGAGTCCTGACCGATTCGGACTATTTGGAG

CCCCTCCTTTGTCGGAGGGGTACGTGGTTCTTTTAGGAGACGAGAGGTCCAAGCCCTCGC

CGCCTCCATCTGAATTTTTGCTTTCGGTTTTTCGCCGAAACCGCGCCGCGCGTCTTGTCT

GTCTCAGTGTTGTTTTGTCATTTGTCTGTTCGTTATTGTTTTGGACCGTTTCTAAAAATA

TGGGACAGACCGTAACCACCCCTCTGAGTCTGACCCTAGAACACTGGGGAGACGTCCAGC

GCATCGCGTCCAACCAGTCCGTGGACGTCAAGAAGAGACGCTGGGTCACCTTCTGCTCTG

CCGAGTGGCCAACTTTCGGTGTAGGGTGGCCGCAAGATGGTACTTTTAATTTGGACATTA

TTTTACAGGTTAAATCTAAGGTGTTCTCTCCCGGTCCCCACGGACACCCGGATCAGGTCC

CATACATTGTCACCTGGGAGGCTATTGCCTATGAACCCCCTCCGTGGGTCAAACCTTTTG

TCTCTCCCAAACTCTCCCTCTCTCCAACCGCTCCCATCCTCCCATCCGGTCCTTCGACCC

AACCTCCGCCCCGATCTGCCCTTTACCCTGCTCTTACCCCCTCTATAAAACCCAGACCTT

CTAAACCTCAGGTTCTCTCCGATAATGGCGGACCTCTCATTGACCTTCTCACAGAAGACC

CTCCGCCGTACGGAGAACAGGGACCGTCCTCCTCTGACGGAGATGGCGACAGAGAAGAGG

CCACCTCCACTCCTGAGATTCCTGCCCCCTCTCCCATGGTGTCTCGCTTGCGGGGCAAAA

GAGACCCCCCCGCGGCAGTTTCCACCACCTCTCGGGCTTTCCCACTCCGTTTGGGGGGTA

ATGGTCAGTTGCAGTACTGGCCGTTTTCCTCCTCGGATCTATATAACTGGAAAAATAATA

ACCCTTCCTTCTCTGAAGATCCAGGTAAATTGACTGCCTTAATCGAGTCTGTCCTCACCA

CCCACCAGCCTACTTGGGATGACTGTCAACAGTTGCTGGGGACTCTGCTGACAGGAGAAG

AAAAGCAGCGGGTGCTCCTGGAAGCCAGAAAGGCAGTCCGGGGCGACGATGGCCGCCCCA

CCCAATTGCCCAATGAGATCGAGGCTGCCTTTCCCCTCGAACGTCCCGACTGGGACTACA

CCACCCTTAGAGGTAGGAACCACCTAGTTCTCTATCGCCAGCTGCTCTTGGCGGGTCTCC

AAAATGCGGGCAGGAGCCCCACCAATTTGGCTAAGGTAAAAGGAATAACCCAGGGGTCCA

ACGAGTCGCCCTCGGCCTTTCTAGAGAGACTCAAAGAGGCCTATCGCAGATACACTCCTT

ATGACCCTGAGGACCCTGGGCAAGAAACCAATGTATCCATGTCGTTCATCTGGCAGTCTG

CTCCAGACATTGGTCGAAAGTTAGAGCGGTTAGAAGACTTAAAAAATAAGACCTTAGGGG

ACTTAGTGAGAGAAGCAGAAAGGATCTTTAATAAGAGAGAGACCCCAGAAGAGAGAGAAG

AACGTATTAAGAGAGAAACAGAGGAAAAAGAGGAGCGCCGTAGGGCAGAGGATGAGCAGA

AAGAGAAAGAGAGGGACCGCAGAAGACAGAGAGAAATGAGCAAACTCTTGGCCACCGTAG

TTACAGGTCAGAGACAGGATAGACAGGGGGGAGAGCGAAGGAGGCCCCAACTCGATAAGG

ACCAATGCGCCTACTGCAAAGAAAAGGGACACTGGGCTAGGGATTGCCCAAAGAAGCCAC

GGGGGCCCCGAGGACCGAGGCCCCAGACCTCCCTCCTGACCCTAGATGACTAGGGAGGTC

AGGGTCAGGAGCCCCCCCCTGAACCCAGGATAACCCTTACTGTCGGGGGGCAACCAGTCA

CCTTCCTGGTGGATACTGGGGCCCAACACTCCGTGCTGACCCAGAACCCTGGACCCCTAA

GTGACAGGTCTGCCTGGGTCCAAGGGGCTACTGGAGGAAAGCGGTATCACTGGACCACAG

ATCGCAAGGTGCACCTGGCTACCGGTAAGGTCACTCACTCTTTCCTCCATGTGCCGGACT

GCCCTTATCCTTTGCTAGGAAGGGACTTGTTGACTAAGTTAAAGGCCCAGATCCACTTCG

AGGGATCGGGAGCTCAGGTTGTGGGACCAAAAGGACAGCCCCTGCAGGTGTTGACCCTTG

GCATAGAGGATGAGTATCGGCTACATGAGACCTCAACAGAGCCGGATGTTTCTCTAGGGT

CCACCTGGCTTTCTGACTTTCCCCAGGCCTGGGCAGAAACCGGGGGCATGGGACTGGCAG

TTCGCCAAGCGCCTCTGATTATACCTCTAAAGGCAACCTCCACCCCTGTGTCCATCAAAC

AGTACCCCATGTCACACGAAGCCAGACTGGGGATCAAGCCCCACATACAGAGACTGTTGG

ACCAGGGAATATTGGTACCTTGCCAGTCCCCCTGGAACACACCCCTGCTGCCCGTTAAGA

AACCAGGGACTAATGATTACAGGCCTGTCCAGGATCTGAGAGAAGTCAACAAGCGGGTGG

AAGATATCCACCCCACCGTGCCCAATCCTTACAACCTCTTAAGTGGACTCCCTCCGTCCC

ACCAGTGGTACACTGTGCTTGATTTAAAAGATGCCTTTTTCTGCCTGAGACTCCACCCCA

CCAGTCAGCCTCTCTTTGCCTTTGAGTGGAGAGATCCAGAAATGGGAATCTCTGGACAAT

TGACCTGGACCAGACTCCCACAGGGTTTCAAAAACAGTCCCACCCTGTTTGATGAGGCAT

TGCACAGAGACCTAGCAGACTTCCGGATCCAGCACCCAGACTTGATCCTGCTACAGTACG

TGGATGACTTACTGCTGGCCGCTACTTCCGAACTAGACTGCCAACAAGGTACTCGGGCCC

TTCTACAAACCCTAGGGGACCTCGGATACCGGGCCTCGGCCAAGAAAGCCCAAATCTGCC

AGAAACAGGTTAAATACCTGGGGTACCTTCTGAGGGAGGGTCAGAGATGGCTGACTGAGG

CTAGAAAAGAGACTGTGATGGGGCAACCCGTTCCAAAGACTCCTCGACAACTAAGGGAGT

TCCTAGGGACGGCAGGCTTCTGCCGCCTCTGGATCCCTGGGTTTGCGGAAATGGCGGCCC

CCTTGTATCCTCTTACCAAAACGGGGACTCTGTTTAATTGGGGCCCAGACCAGCAAAAGG

CCTATCAAGAAATCAAACAGGCCCTTCTAACTGCCCCCGCCCTGGGATTGCCAGATTTGA

CTAAGCCCTTTGAACTCTTTGTCGACGAGAAGCAGGGCTACGCCAAAGGCGTCCTAACGC

AAAAACTGGGACCTTGGCGTCGGCCTGTGGCCTACCTGTCCAAAAAGCTAGACCCAGTGG

CAGCCGGGTGGCCCCCTTGCCTACGGATGGTAGCAGCCATTGCCGTTCTGACAAAAGATG

CAGGCAAGCTAACTATGGGACAGCCGCTAGTCATCCTGGCCCCCCATGCAGTAGAGGCAC

TGGTCAAGCAACCCCCTGACCGCTGGCTATCCAACGCCCGCATGACCCACTACCAGGCAA

TGCTCCTAGACACTGACCGAGTTCAGTTCGGACCAGTGGTGGCCCTCAATCCTGCCACCT

TGCTCCCTCTACCGGAAAAAGGAGCCCCCCATGATTGCCTCGAGATCTTGGCTGAAACGC

ATGGAACCAGACCGGATCTCACCGACCAGCCCATCCCAGACGCCGACCACACCTGGTATA

CCGATGGGAGCAGCTTTCTGCAAGAAGGACAGCGAAAGGCTGGGGCAGCAGTGACGACTG

AAACCGAGGTAATCTGGGCGAGGGCCCTGCCAGCTGGAACGTCAGCCCAGCGAGCCGAAC

TGATCGCACTCACCCAAGCCCTGAAAATGGCAGAAGGTAAGAAGCTAAATGTTTACACTG

ATAGCCGCTATGCCTTCGCTACGGCCCATGTTCATGGGGAAATATATAGGAGACGGGGGT

TGCTGACCTCAGAAGGCAAGGAAATCAAGAACAAAAGCGAGATCCTAGCCTTGCTGAAAG

CCCTCTTTTTGCCAAAGAGACTCAGTATTATCCATTGCCCAGGACATCAGAAAGGAGACA

GTGCCGAAGCCAGAGGCAACCGTATGGCAGACCAGGCGGCCCGAGAGGCAGCCACAAAAA

CAGTTCCAGAAGCCTCTACACTCCTTATAGAGGACTCGACCCCGTACACGCCTGCCTATC

TCCATTACACCGAAACAGATCTAAAAAGATTGCGAGAACTGGGGGCCACCTATAATCAGA

TAAAAGGATATTGGGTCCTACAAGGCAAGCCGGTGATGCCCGATCAGTTTGTGTTTGAAT

TATTAGACTCCCTTCATAGACTCACCCATCTCAGCCCTCAAAAGATGAAGGCGCTCCTTG

ACAGAGAAGAAAGCCCCTACTACATGTTAAACAGGGACAGAACTCTTCAGTATGTGGCAG

AATCCTGCACAGTCTGTGCTCAAGTAAATGCTAGTAAAGCCAAAATCGGGGCAGGGGTAC

GAGTACGCGGACATCGACCAGGTACCCATTGGGAAATTGACTTCACTGAAGTTAAACCAG

GGCTGTACGGGTACAAGTACCTCCTGGTGTTCGTAGACACCTTCTCTGGCTGGGTGGAAG

CCTTCCCAACTAAACGTGAAACTGCCAAGGTTGTGACCAAGAAGCTATTAGAAGAAATAT

TCCCAAGATTCGGGATGCCACAGGTATTGGGTTCCGATAATGGGCCTGCCTTCGTCTCCC

AGGTAAGTCAGTCGGTGGCCGATTTACTGGGGATCGATTGGAAATTACATTGTGCTTATA

GACCCCAGAGTTCAGGTCAGGTAGAAAGAATGAATAGAACCATCAAGGAGACTCTAACTA

AATTAACGCTTGCAGCTGGCACTAGAGACTGGGTACTCCTACTCCCCTTAGCCCTCTACC

GAGCCCGGAACACTCCGGGCCCCCATGGACTGACTCCGTATGAAATTCTGTATGGGGCAC

CCCCGCCCCTTGTCAATTTTCATGATCCTGAAATGTCAAAGTTAACTAATAGTCCCTCTC

TCCAAGCTCACTTACAGGCCCTCCAAGCAGTACAACGAGAGGTCTGGAAGCCGCTGGCCG

CTGCTTATCAGGACCAGCTAGATCAGCCAGTGATACCACACCCCTTCCGTGTCGGTGACG

CCGTGTGGGTACGCCGGCACCAGACTAAGAACTTGGAACCTCGCTGGAAAGGACCCTACA

CCGTCCTGCTGACCACCCCCACCGCTCTCAAAGTTGACGGCATCTCTGCGTGGATACACG

CCGCTCACGTAAAGGCGGCGACAACTCCTCCGGCCGGAGCAGCATGGAAGGTCCAGCGTT

CTCAAAACCCCTTAAAGATAAGATTAACCCGTGGGGCCCCCTAATAGTTATAGGGATCTT

GGTGAGGGCAGGAGCCTCGGTACAACGTGACAGCCCTCACCAGGTCTTCAATGTCACTTG

GAGAGTTACCAACCTAATGACAGGACAAACAGCTAACGCTACCTCCCTCCTGGGGACGAT

GACAGACACCTTCCCTAAACTATATTTTGACTTGTGTGATTTAGTTGGAGACCATTGGGA

TGACCCAGAACCCGATATTGGAGATGGTTGCCGCTCTCCGGGGGGAAGAAAAAGGACAAG

ACTGTATGACTTCTATGTTTGCCCCGGTCATACTGTACCAATAGGGTGTGGAGGGCCGGG

AGAGGGCTACTGTGGCAAATGGGGATGTGAGACCACTGGACAGGCATACTGGAAGCCATC

ATCATCATGGGACCTAATTTCCCTTAAGCGAGGAAACACTCCTAAGGATCAGGGCCCCTG

TTATGATTCCTCGGTCTCCAGTGGCGTCCAGGGTGCCACACCGGGGGGTCGATGCAACCC

CCTAGTCTTAGAATTCACTGACGCGGGTAAAAAGGCCAGCTGGGATGCCCCCAAAGTTTG

GGGACTAAGACTCTACCGATCCACGGGGGCCGACCCGGTGACCCGGTTCTCTTTGACCCG

CCAGGTCCTCAATGTAGGACCCCGCGTCCCCATTGGGCCTAATCCCGTGATCACTGAACA

GCTACCCCCCTCCCAACCCGTGCAGATCATGCTCCCCAGGCCTCCTCATCCTCCTCCTTC

AGGCGCGGCCTCTATGGTGCCTGGGGCTCCCCCGCCTTCTCAACAACCTGGGACGGGGGA

CAGGCTGCTAAACCTAGTAAAAGGAGCCTATCAAGCACTCAACCTCACCAGTCCCGACAG

AACCCAAGAGTGCTGGCTGTGTCTGGTATCGGGACCCCCCTACTACGAAGGGGTTGCCGT

CCTAGGTACCTACTCCAACCATACCTCTGCCCCAGCTAACTGCTCCGTGGCCTCCCAACA

CAAGCTGACCCTGTCCGAAGTGACCGGGCAGGGACTCTGCGTAGGAGCAGTTCCCAAAAC

CCATCAGGCCCTGTGTAATACCACCCAGAAGGCGAGCGACGGGTCCTACTATCTGGCTGC

TCCCGCCGGGACCATCTGGGCTTGCAACACCGGGCTCACTCCCTGCCTATCTACCACTGT

ACTCAACCTCACCACCGATTACTGTGTCCTGGTTGAGCTCTGGCCAAAGGTGACCTACCA

CTCCCCTGGTTATGTTTATGACCAGTTTGAGAGAAAAACCAAATATAAAAGAGAGCCGGT

GTCATTAACTCTGGCCCTGCTGTTGGGAGGACTTACTATGGGCGGCATAGCTGCAGGAGT

AGGAACAGGGACTACAGCCCTAGTGGCCACCAAACAATTCGAGCAGCTCCAGGCAGCCAT

ACATACAGACCTTGGGGCCTTAGAAAAATCAGTCAGTGCCCTAGAAAAGTCTCTGACCTC

GTTGTCTGAGGTGGTCCTACAGAACCGGAGAGGATTAGATCTGCTGTTCCTAAAAGAAGG

AGGATTATGTGCTGCCCTAAAAGAAGAATGCTGTTTCTATGCAGACCACACTGGCGTAGT

AAGGGATAGCATGGCTAAGCTAAGAGAAAGGCTAAACCAGAGGCAAAAATTGTTCGAATC

AGGACAAGGGTGGTTTGAGGGACTGTTTAACAGGTCCCCATGGTTCACGACCCTGATATC

CACCATTATGGGCCCTCTGATAGTACTTTTATTAATCCTACTCCTCGGACCCTGCATTCT

CAACCGCTTGGTCCAGTTTGTAAAAGACAGAATTTCGGTGGTGCAGGCCCTGGTTCTGAC

CCAACAGTATCACCAACTCAAATCAATAGATCCAGAAGAAGTAGAATCGCGTGAATAAAA

GATTTTATTCAGTTTCCAGAAAGAGGGGGGAATGAAAGACCCCACCATAAGGCTTAGCAA

GCTAGCTGCAGTAACGCCATTTTGCAAGGCATGAAAAAGTACCAGAGCTGAGTTCTCAAA

AGTCACAAGGAAGTTTAGTTAAAGAATAAGGCTGAACAAAACTGGGACAGGGGCCAAACA

GGATATCTGTGGTCGAGCACCTGGGCCCCGGCTCAGGGCCAAGAACAGATGGTACTCAGA

TAAAGCGAAACTAGCAACAGTTTCTGGAAAGTCCCACCTCAGTTTCAAGTTCCCCAAAAG

ACCGGGAAAAACCCCAAGCCTTATTTAAACTAACCAATCAGCTCGCTTCTCGCTTCTGTA

ACCGCGCTTTTTGCTCCCCAGCCCTATAAAAAGGGTAAAAACCCCACACTCGGTGCGCCA

GTCATCCGATAGACTGAGTCGCCCGGGTACCCGTGTTCCCAATAAAGCCTTTTGCTGTTT

GCATCCGAAACGTGGCCTCGCTGTTCCTTGGGAGGGTCTCCTCAGAGTGATTGACTACCC

AGCTCGGGGGTCTTTCA

>1F_Bxv1-2-R_B11_Run_3130_2020-02-03_16-08_2856 (reversed)

------------------------------------------------------------

------------------------------------------------------------

------------------------------------------------------------

------------------------------------------------------------

------------------------------------------------------------

------------------------------------------------------------

------------------------------------------------------------

------------------------------------------------------------

------------------------------------------------------------

------------------------------------------------------------

------------------------------------------------------------

------------------------------------------------------------

------------------------------------------------------------

------------------------------------------------------------

------------------------------------------------------------

------------------------------------------------------------

------------------------------------------------------------

------------------------------------------------------------

------------------------------------------------------------

------------------------------------------------------------

------------------------------------------------------------

------------------------------------------------------------

------------------------------------------------------------

------------------------------------------------------------

------------------------------------------------------------

------------------------------------------------------------

------------------------------------------------------------

------------------------------------------------------------

------------------------------------------------------------

------------------------------------------------------------

------------------------------------------------------------

------------------------------------------------------------

------------------------------------------------------------

------------------------------------------------------------

------------------------------------------------------------

------------------------------------------------------------

------------------------------------------------------------

------------------------------------------------------------

------------------------------------------CCCCAGAAGAGAGAGAAG

AACGTATTAAGAGAGAAACAGAGGAAAAAGAGGAGCGCCGTAGGGCAGAGGATGAGCAGA

AAGAGAAAGAGAGGGACCGCAGAAGACAGAGAGAAA------------------------

------------------------------------------------------------

------------------------------------------------------------

------------------------------------------------------------

------------------------------------------------------------

------------------------------------------------------------

------------------------------------------------------------

------------------------------------------------------------

------------------------------------------------------------

------------------------------------------------------------

------------------------------------------------------------

------------------------------------------------------------

------------------------------------------------------------

------------------------------------------------------------

------------------------------------------------------------

------------------------------------------------------------

------------------------------------------------------------

------------------------------------------------------------

------------------------------------------------------------

------------------------------------------------------------

------------------------------------------------------------

------------------------------------------------------------

------------------------------------------------------------

------------------------------------------------------------

------------------------------------------------------------

------------------------------------------------------------

------------------------------------------------------------

------------------------------------------------------------

------------------------------------------------------------

------------------------------------------------------------

------------------------------------------------------------

------------------------------------------------------------

------------------------------------------------------------

------------------------------------------------------------

------------------------------------------------------------

------------------------------------------------------------

------------------------------------------------------------

------------------------------------------------------------

------------------------------------------------------------

------------------------------------------------------------

------------------------------------------------------------

------------------------------------------------------------

------------------------------------------------------------

------------------------------------------------------------

------------------------------------------------------------

------------------------------------------------------------

------------------------------------------------------------

------------------------------------------------------------

------------------------------------------------------------

------------------------------------------------------------

------------------------------------------------------------

------------------------------------------------------------

------------------------------------------------------------

------------------------------------------------------------

------------------------------------------------------------

------------------------------------------------------------

------------------------------------------------------------

------------------------------------------------------------

------------------------------------------------------------

------------------------------------------------------------

------------------------------------------------------------

------------------------------------------------------------

------------------------------------------------------------

------------------------------------------------------------

------------------------------------------------------------

------------------------------------------------------------

------------------------------------------------------------

------------------------------------------------------------

------------------------------------------------------------

------------------------------------------------------------

------------------------------------------------------------

------------------------------------------------------------

------------------------------------------------------------

------------------------------------------------------------

------------------------------------------------------------

------------------------------------------------------------

------------------------------------------------------------

------------------------------------------------------------

------------------------------------------------------------

------------------------------------------------------------

------------------------------------------------------------

------------------------------------------------------------

------------------------------------------------------------

------------------------------------------------------------

------------------------------------------------------------

------------------------------------------------------------

------------------------------------------------------------

------------------------------------------------------------

------------------------------------------------------------

------------------------------------------------------------

------------------------------------------------------------

------------------------------------------------------------

------------------------------------------------------------

------------------------------------------------------------

------------------------------------------------------------

------------------------------------------------------------

------------------------------------------------------------

------------------------------------------------------------

------------------------------------------------------------

------------------------------------------------------------

------------------------------------------------------------

------------------------------------------------------------

------------------------------------------------------------

------------------------------------------------------------

-----------------

>1R_Bxv1-2-F_A11_Run_3130_2020-02-03_16-08_2856

------------------------------------------------------------

------------------------------------------------------------

------------------------------------------------------------

------------------------------------------------------------

------------------------------------------------------------

------------------------------------------------------------

------------------------------------------------------------

------------------------------------------------------------

------------------------------------------------------------

------------------------------------------------------------

------------------------------------------------------------

------------------------------------------------------------

------------------------------------------------------------

------------------------------------------------------------

------------------------------------------------------------

------------------------------------------------------------

------------------------------------------------------------

------------------------------------------------------------

------------------------------------------------------------

------------------------------------------------------------

------------------------------------------------------------

------------------------------------------------------------

------------------------------------------------------------

------------------------------------------------------------

------------------------------------------------------------

------------------------------------------------------------

------------------------------------------------------------

------------------------------------------------------------

------------------------------------------------------------

------------------------------------------------------------

------------------------------------------------------------

------------------------------------------------------------

------------------------------------------------------------

------------------------------------------------------------

------------------------------------------------------------

------------------------------------------------------------

------------------------------------------------------------

------------------------------------------------------------

------------------------------------------------------------

--------------------------------------------------------CAGA

AAGAGAAAGAGAGGGACCGCAGAAGACAGAGAGAAATGAGCAAACTCTTGGCCACCGTAG

TTACAGGTCAGAGACAGGATAGACAGGGGGGAGAGCGAAGGAGGCCCCAACTCGATAAGG

ACCAATG-----------------------------------------------------

------------------------------------------------------------

------------------------------------------------------------

------------------------------------------------------------

------------------------------------------------------------

------------------------------------------------------------

------------------------------------------------------------

------------------------------------------------------------

------------------------------------------------------------

------------------------------------------------------------

------------------------------------------------------------

------------------------------------------------------------

------------------------------------------------------------

------------------------------------------------------------

------------------------------------------------------------

------------------------------------------------------------

------------------------------------------------------------

------------------------------------------------------------

------------------------------------------------------------

------------------------------------------------------------

------------------------------------------------------------

------------------------------------------------------------

------------------------------------------------------------

------------------------------------------------------------

------------------------------------------------------------

------------------------------------------------------------

------------------------------------------------------------

------------------------------------------------------------

------------------------------------------------------------

------------------------------------------------------------

------------------------------------------------------------

------------------------------------------------------------

------------------------------------------------------------

------------------------------------------------------------

------------------------------------------------------------

------------------------------------------------------------

------------------------------------------------------------

------------------------------------------------------------

------------------------------------------------------------

------------------------------------------------------------

------------------------------------------------------------

------------------------------------------------------------

------------------------------------------------------------

------------------------------------------------------------

------------------------------------------------------------

------------------------------------------------------------

------------------------------------------------------------

------------------------------------------------------------

------------------------------------------------------------

------------------------------------------------------------

------------------------------------------------------------

------------------------------------------------------------

------------------------------------------------------------

------------------------------------------------------------

------------------------------------------------------------

------------------------------------------------------------

------------------------------------------------------------

------------------------------------------------------------

------------------------------------------------------------

------------------------------------------------------------

------------------------------------------------------------

------------------------------------------------------------

------------------------------------------------------------

------------------------------------------------------------

------------------------------------------------------------

------------------------------------------------------------

------------------------------------------------------------

------------------------------------------------------------

------------------------------------------------------------

------------------------------------------------------------

------------------------------------------------------------

------------------------------------------------------------

------------------------------------------------------------

------------------------------------------------------------

------------------------------------------------------------

------------------------------------------------------------

------------------------------------------------------------

------------------------------------------------------------

------------------------------------------------------------

------------------------------------------------------------

------------------------------------------------------------

------------------------------------------------------------

------------------------------------------------------------

------------------------------------------------------------

------------------------------------------------------------

------------------------------------------------------------

------------------------------------------------------------

------------------------------------------------------------

------------------------------------------------------------

------------------------------------------------------------

------------------------------------------------------------

------------------------------------------------------------

------------------------------------------------------------

------------------------------------------------------------

------------------------------------------------------------

------------------------------------------------------------

------------------------------------------------------------

------------------------------------------------------------

------------------------------------------------------------

------------------------------------------------------------

------------------------------------------------------------

------------------------------------------------------------

-----------------

>2F_Bxv1-3-R_D11_Run_3130_2020-02-03_16-08_2856 (reversed)

------------------------------------------------------------

------------------------------------------------------------

------------------------------------------------------------

------------------------------------------------------------

------------------------------------------------------------

------------------------------------------------------------

------------------------------------------------------------

------------------------------------------------------------

------------------------------------------------------------

------------------------------------------------------------

------------------------------------------------------------

------------------------------------------------------------

------------------------------------------------------------

------------------------------------------------------------

------------------------------------------------------------

------------------------------------------------------------

------------------------------------------------------------

------------------------------------------------------------

------------------------------------------------------------

------------------------------------------------------------

------------------------------------------------------------

------------------------------------------------------------

------------------------------------------------------------

------------------------------------------------------------

------------------------------------------------------------

------------------------------------------------------------

------------------------------------------------------------

------------------------------------------------------------

------------------------------------------------------------

------------------------------------------------------------

------------------------------------------------------------

------------------------------------------------------------

------------------------------------------------------------

------------------------------------------------------------

------------------------------------------------------------

------------------------------------------------------------

------------------------------------------------------------

------------------------------------------------------------

------------------------------------------------------------

------------------------------------------------------------

------------------------------------------------------------

------------------------------------------------------------

------------------------------------------------------------

------------------------------------------------------------

------------------------------------------------------------

------------------------------------------------------------

------------------------------------------------------------

------------------------------------------------------------

------------------------------------------------------------

------------------------------------------------------------

------------------------------------------------------------

------------------------------------------------------------

------------------------------------------------------------

------------------------------------------------------------

------------------------------------------------------------

------------------------------------------------------------

------------------------------------------------------------

------------------------------------------------------------

----------------------------GAGAGATCCAGAAATGGGAATCTCTGGACAAT

TGACCTGGACCAGACTCCCACAGGGTTTCAAAAACAGTCCCACCCTGTTTGATGAGGCAT

TGCACAGAGACCTAGCAGACTTCCGGATCCAGCACCC-----------------------

------------------------------------------------------------

------------------------------------------------------------

------------------------------------------------------------

------------------------------------------------------------

------------------------------------------------------------

------------------------------------------------------------

------------------------------------------------------------

------------------------------------------------------------

------------------------------------------------------------

------------------------------------------------------------

------------------------------------------------------------

------------------------------------------------------------

------------------------------------------------------------

------------------------------------------------------------

------------------------------------------------------------

------------------------------------------------------------

------------------------------------------------------------

------------------------------------------------------------

------------------------------------------------------------

------------------------------------------------------------

------------------------------------------------------------

------------------------------------------------------------

------------------------------------------------------------

------------------------------------------------------------

------------------------------------------------------------

------------------------------------------------------------

------------------------------------------------------------

------------------------------------------------------------

------------------------------------------------------------

------------------------------------------------------------

------------------------------------------------------------

------------------------------------------------------------

------------------------------------------------------------

------------------------------------------------------------

------------------------------------------------------------

------------------------------------------------------------

------------------------------------------------------------

------------------------------------------------------------

------------------------------------------------------------

------------------------------------------------------------

------------------------------------------------------------

------------------------------------------------------------

------------------------------------------------------------

------------------------------------------------------------

------------------------------------------------------------

------------------------------------------------------------

------------------------------------------------------------

------------------------------------------------------------

------------------------------------------------------------

------------------------------------------------------------

------------------------------------------------------------

------------------------------------------------------------

------------------------------------------------------------

------------------------------------------------------------

------------------------------------------------------------

------------------------------------------------------------

------------------------------------------------------------

------------------------------------------------------------

------------------------------------------------------------

------------------------------------------------------------

------------------------------------------------------------

------------------------------------------------------------

------------------------------------------------------------

------------------------------------------------------------

------------------------------------------------------------

------------------------------------------------------------

------------------------------------------------------------

------------------------------------------------------------

------------------------------------------------------------

------------------------------------------------------------

------------------------------------------------------------

------------------------------------------------------------

------------------------------------------------------------

------------------------------------------------------------

------------------------------------------------------------

------------------------------------------------------------

------------------------------------------------------------

------------------------------------------------------------

------------------------------------------------------------

------------------------------------------------------------

------------------------------------------------------------

------------------------------------------------------------

------------------------------------------------------------

-----------------

>2R_Bxv1-3-F_C11_Run_3130_2020-02-03_16-08_2856

------------------------------------------------------------

------------------------------------------------------------

------------------------------------------------------------

------------------------------------------------------------

------------------------------------------------------------

------------------------------------------------------------

------------------------------------------------------------

------------------------------------------------------------

------------------------------------------------------------

------------------------------------------------------------

------------------------------------------------------------

------------------------------------------------------------

------------------------------------------------------------

------------------------------------------------------------

------------------------------------------------------------

------------------------------------------------------------

------------------------------------------------------------

------------------------------------------------------------

------------------------------------------------------------

------------------------------------------------------------

------------------------------------------------------------

------------------------------------------------------------

------------------------------------------------------------

------------------------------------------------------------

------------------------------------------------------------

------------------------------------------------------------

------------------------------------------------------------

------------------------------------------------------------

------------------------------------------------------------

------------------------------------------------------------

------------------------------------------------------------

------------------------------------------------------------

------------------------------------------------------------

------------------------------------------------------------

------------------------------------------------------------

------------------------------------------------------------

------------------------------------------------------------

------------------------------------------------------------

------------------------------------------------------------

------------------------------------------------------------

------------------------------------------------------------

------------------------------------------------------------

------------------------------------------------------------

------------------------------------------------------------

------------------------------------------------------------

------------------------------------------------------------

------------------------------------------------------------

------------------------------------------------------------

------------------------------------------------------------

------------------------------------------------------------

------------------------------------------------------------

------------------------------------------------------------

------------------------------------------------------------

------------------------------------------------------------

------------------------------------------------------------

------------------------------------------------------------

------------------------------------------------------------

------------------------------------------------------------

------------------------------------------------------------

----------------------------------CAGTCCCACCCTGTTTGATGAGGCAT

TGCACAGAGACCTAGCAGACTTCCGGATCCAGCACCCAGACTTGATCCTGCTACAGTACG

TGGATGACTTACTGCTGGCCGCTACTTCCGAACTAGACTGCCAACAAGGTACTCGGGCCC

TTCTAC------------------------------------------------------

------------------------------------------------------------

------------------------------------------------------------

------------------------------------------------------------

------------------------------------------------------------

------------------------------------------------------------

------------------------------------------------------------

------------------------------------------------------------

------------------------------------------------------------

------------------------------------------------------------

------------------------------------------------------------

------------------------------------------------------------

------------------------------------------------------------

------------------------------------------------------------

------------------------------------------------------------

------------------------------------------------------------

------------------------------------------------------------

------------------------------------------------------------

------------------------------------------------------------

------------------------------------------------------------

------------------------------------------------------------

------------------------------------------------------------

------------------------------------------------------------

------------------------------------------------------------

------------------------------------------------------------

------------------------------------------------------------

------------------------------------------------------------

------------------------------------------------------------

------------------------------------------------------------

------------------------------------------------------------

------------------------------------------------------------

------------------------------------------------------------

------------------------------------------------------------

------------------------------------------------------------

------------------------------------------------------------

------------------------------------------------------------

------------------------------------------------------------

------------------------------------------------------------

------------------------------------------------------------

------------------------------------------------------------

------------------------------------------------------------

------------------------------------------------------------

------------------------------------------------------------

------------------------------------------------------------

------------------------------------------------------------

------------------------------------------------------------

------------------------------------------------------------

------------------------------------------------------------

------------------------------------------------------------

------------------------------------------------------------

------------------------------------------------------------

------------------------------------------------------------

------------------------------------------------------------

------------------------------------------------------------

------------------------------------------------------------

------------------------------------------------------------

------------------------------------------------------------

------------------------------------------------------------

------------------------------------------------------------

------------------------------------------------------------

------------------------------------------------------------

------------------------------------------------------------

------------------------------------------------------------

------------------------------------------------------------

------------------------------------------------------------

------------------------------------------------------------

------------------------------------------------------------

------------------------------------------------------------

------------------------------------------------------------

------------------------------------------------------------

------------------------------------------------------------

------------------------------------------------------------

------------------------------------------------------------

------------------------------------------------------------

------------------------------------------------------------

------------------------------------------------------------

------------------------------------------------------------

------------------------------------------------------------

------------------------------------------------------------

------------------------------------------------------------

------------------------------------------------------------

------------------------------------------------------------

-----------------

>3F_Bxv1-5-R_H11_Run_3130_2020-02-03_16-08_2857 (reversed)

------------------------------------------------------------

------------------------------------------------------------

------------------------------------------------------------

------------------------------------------------------------

------------------------------------------------------------

------------------------------------------------------------

------------------------------------------------------------

------------------------------------------------------------

------------------------------------------------------------

------------------------------------------------------------

------------------------------------------------------------

------------------------------------------------------------

------------------------------------------------------------

------------------------------------------------------------

------------------------------------------------------------

------------------------------------------------------------

------------------------------------------------------------

------------------------------------------------------------

------------------------------------------------------------

------------------------------------------------------------

------------------------------------------------------------

------------------------------------------------------------

------------------------------------------------------------

------------------------------------------------------------

------------------------------------------------------------

------------------------------------------------------------

------------------------------------------------------------

------------------------------------------------------------

------------------------------------------------------------

------------------------------------------------------------

------------------------------------------------------------

------------------------------------------------------------

------------------------------------------------------------

------------------------------------------------------------

------------------------------------------------------------

------------------------------------------------------------

------------------------------------------------------------

------------------------------------------------------------

------------------------------------------------------------

------------------------------------------------------------

------------------------------------------------------------

------------------------------------------------------------

------------------------------------------------------------

------------------------------------------------------------

------------------------------------------------------------

------------------------------------------------------------

------------------------------------------------------------

------------------------------------------------------------

------------------------------------------------------------

------------------------------------------------------------

------------------------------------------------------------

------------------------------------------------------------

------------------------------------------------------------

------------------------------------------------------------

------------------------------------------------------------

------------------------------------------------------------

------------------------------------------------------------

------------------------------------------------------------

------------------------------------------------------------

------------------------------------------------------------

------------------------------------------------------------

------------------------------------------------------------

------------------------------------------------------------

------------------------------------------------------------

------------------------------------------------------------

------------------------------------------------------------

------------------------------------------------------------

------------------------------------------------------------

------------------------------------------------------------

------------------------------------------------------------

------------------------------------------------------------

CAGGCAAGCTAACTATGGGACAGCCGCTAGTCATCCTGGCCCCCCATGCAGTAGAGGCAC

TGGTCAAGCAACCCCCTGACCGCTGGCTATCCAACGCCCGCATGACCCACTACCAGGCAA

TGCTCCTAGACACTGACCGAGTTCAGTTCGGACCAGTGGTGGCCCTCAATCCTGCCACCT

TGCTCCCTCTACCGGAAAAAGGAGCCCCCCATGATTGCCTCGAGATCTTGGCTGAAACGC

ATGGAACCAGACCGGATCTCACCGACCAGCCCATCCCAGACGCCGACCACACC-------

------------------------------------------------------------

------------------------------------------------------------

------------------------------------------------------------

------------------------------------------------------------

------------------------------------------------------------

------------------------------------------------------------

------------------------------------------------------------

------------------------------------------------------------

------------------------------------------------------------

------------------------------------------------------------

------------------------------------------------------------

------------------------------------------------------------

------------------------------------------------------------

------------------------------------------------------------

------------------------------------------------------------

------------------------------------------------------------

------------------------------------------------------------

------------------------------------------------------------

------------------------------------------------------------

------------------------------------------------------------

------------------------------------------------------------

------------------------------------------------------------

------------------------------------------------------------

------------------------------------------------------------

------------------------------------------------------------

------------------------------------------------------------

------------------------------------------------------------

------------------------------------------------------------

------------------------------------------------------------

------------------------------------------------------------

------------------------------------------------------------

------------------------------------------------------------

------------------------------------------------------------

------------------------------------------------------------

------------------------------------------------------------

------------------------------------------------------------

------------------------------------------------------------

------------------------------------------------------------

------------------------------------------------------------

------------------------------------------------------------

------------------------------------------------------------

------------------------------------------------------------

------------------------------------------------------------

------------------------------------------------------------

------------------------------------------------------------

------------------------------------------------------------

------------------------------------------------------------

------------------------------------------------------------

------------------------------------------------------------

------------------------------------------------------------

------------------------------------------------------------

------------------------------------------------------------

------------------------------------------------------------

------------------------------------------------------------

------------------------------------------------------------

------------------------------------------------------------

------------------------------------------------------------

------------------------------------------------------------

------------------------------------------------------------

------------------------------------------------------------

------------------------------------------------------------

------------------------------------------------------------

------------------------------------------------------------

------------------------------------------------------------

------------------------------------------------------------

------------------------------------------------------------

------------------------------------------------------------

------------------------------------------------------------

-----------------

>3R_Bxv1-5-F_G11_Run_3130_2020-02-03_16-08_2857

------------------------------------------------------------

------------------------------------------------------------

------------------------------------------------------------

------------------------------------------------------------

------------------------------------------------------------

------------------------------------------------------------

------------------------------------------------------------

------------------------------------------------------------

------------------------------------------------------------

------------------------------------------------------------

------------------------------------------------------------

------------------------------------------------------------

------------------------------------------------------------

------------------------------------------------------------

------------------------------------------------------------

------------------------------------------------------------

------------------------------------------------------------

------------------------------------------------------------

------------------------------------------------------------

------------------------------------------------------------

------------------------------------------------------------

------------------------------------------------------------

------------------------------------------------------------

------------------------------------------------------------

------------------------------------------------------------

------------------------------------------------------------

------------------------------------------------------------

------------------------------------------------------------

------------------------------------------------------------

------------------------------------------------------------

------------------------------------------------------------

------------------------------------------------------------

------------------------------------------------------------

------------------------------------------------------------

------------------------------------------------------------

------------------------------------------------------------

------------------------------------------------------------

------------------------------------------------------------

------------------------------------------------------------

------------------------------------------------------------

------------------------------------------------------------

------------------------------------------------------------

------------------------------------------------------------

------------------------------------------------------------

------------------------------------------------------------

------------------------------------------------------------

------------------------------------------------------------

------------------------------------------------------------

------------------------------------------------------------

------------------------------------------------------------

------------------------------------------------------------

------------------------------------------------------------

------------------------------------------------------------

------------------------------------------------------------

------------------------------------------------------------

------------------------------------------------------------

------------------------------------------------------------

------------------------------------------------------------

------------------------------------------------------------

------------------------------------------------------------

------------------------------------------------------------

------------------------------------------------------------

------------------------------------------------------------

------------------------------------------------------------

------------------------------------------------------------

------------------------------------------------------------

------------------------------------------------------------

------------------------------------------------------------

------------------------------------------------------------

------------------------------------------------------------

------------------------------------------------------------

------------------------------------------------------------

-------GCAACCCCCTGACCGCTGGCTATCCAACGCCCGCATGACCCACTACCAGGCAA

TGCTCCTAGACACTGACCGAGTTCAGTTCGGACCAGTGGTGGCCCTCAATCCTGCCACCT

TGCTCCCTCTACCGGAAAAAGGAGCCCCCCATGATTGCCTCGAGATCTTGGCTGAAACGC

ATGGAACCAGACCGGATCTCACCGACCAGCCCATCCCAGACGCCGACCACACCTGGTATA

CCGATGGGAGCAGCTTTCTGCAAGAAGGACAGCGAAAGGCTGGGGCAGCAGTGACGACTG

AAACCGAGGTAATCTGGG------------------------------------------

------------------------------------------------------------

------------------------------------------------------------

------------------------------------------------------------

------------------------------------------------------------

------------------------------------------------------------

------------------------------------------------------------

------------------------------------------------------------

------------------------------------------------------------

------------------------------------------------------------

------------------------------------------------------------

------------------------------------------------------------

------------------------------------------------------------

------------------------------------------------------------

------------------------------------------------------------

------------------------------------------------------------

------------------------------------------------------------

------------------------------------------------------------

------------------------------------------------------------

------------------------------------------------------------

------------------------------------------------------------

------------------------------------------------------------

------------------------------------------------------------

------------------------------------------------------------

------------------------------------------------------------

------------------------------------------------------------

------------------------------------------------------------

------------------------------------------------------------

------------------------------------------------------------

------------------------------------------------------------

------------------------------------------------------------

------------------------------------------------------------

------------------------------------------------------------

------------------------------------------------------------

------------------------------------------------------------

------------------------------------------------------------

------------------------------------------------------------

------------------------------------------------------------

------------------------------------------------------------

------------------------------------------------------------

------------------------------------------------------------

------------------------------------------------------------

------------------------------------------------------------

------------------------------------------------------------

------------------------------------------------------------

------------------------------------------------------------

------------------------------------------------------------

------------------------------------------------------------

------------------------------------------------------------

------------------------------------------------------------

------------------------------------------------------------

------------------------------------------------------------

------------------------------------------------------------

------------------------------------------------------------

------------------------------------------------------------

------------------------------------------------------------

------------------------------------------------------------

------------------------------------------------------------

------------------------------------------------------------

------------------------------------------------------------

------------------------------------------------------------

------------------------------------------------------------

------------------------------------------------------------

------------------------------------------------------------

------------------------------------------------------------

------------------------------------------------------------

------------------------------------------------------------

-----------------

>4F_Bxv1-4-R_F11_Run_3130_2020-02-03_16-08_2857 (reversed)

------------------------------------------------------------

------------------------------------------------------------

------------------------------------------------------------

------------------------------------------------------------

------------------------------------------------------------

------------------------------------------------------------

------------------------------------------------------------

------------------------------------------------------------

------------------------------------------------------------

------------------------------------------------------------

------------------------------------------------------------

------------------------------------------------------------

------------------------------------------------------------

------------------------------------------------------------

------------------------------------------------------------

------------------------------------------------------------

------------------------------------------------------------

------------------------------------------------------------

------------------------------------------------------------

------------------------------------------------------------

------------------------------------------------------------

------------------------------------------------------------

------------------------------------------------------------

------------------------------------------------------------

------------------------------------------------------------

------------------------------------------------------------

------------------------------------------------------------

------------------------------------------------------------

------------------------------------------------------------

------------------------------------------------------------

------------------------------------------------------------

------------------------------------------------------------

------------------------------------------------------------

------------------------------------------------------------

------------------------------------------------------------

------------------------------------------------------------

------------------------------------------------------------

------------------------------------------------------------

------------------------------------------------------------

------------------------------------------------------------

------------------------------------------------------------

------------------------------------------------------------

------------------------------------------------------------

------------------------------------------------------------

------------------------------------------------------------

------------------------------------------------------------

------------------------------------------------------------

------------------------------------------------------------

------------------------------------------------------------

------------------------------------------------------------

------------------------------------------------------------

------------------------------------------------------------

------------------------------------------------------------

------------------------------------------------------------

------------------------------------------------------------

------------------------------------------------------------

------------------------------------------------------------

------------------------------------------------------------

------------------------------------------------------------

------------------------------------------------------------

------------------------------------------------------------

------------------------------------------------------------

------------------------------------------------------------

------------------------------------------------------------

------------------------------------------------------------

------------------------------------------------------------

------------------------------------------------------------

------------------------------------------------------------

------------------------------------------------------------

------------------------------------------------------------

------------------------------------------------------------

------------------------------------------------------------

------------------------------------------------------------

------------------------------------------------------------

------------------------------------------------------------

------------------------------------------------------------

------------------------------------------------------------

------------------------------------------------------------

------------------------------------------------------------

------------------------------------------------------------

------------------------------------------------------------

------------------------------------------------------------

------------------------------------------------------------

------------------------------------------------------------

------------------------------------------------------------

------------------------------------------------------------

------------------------------------------------------------

------------------------------------------------------------

------------------------------------------------------------

------------------------------------------------------------

------------------------------------------------------------

------------------------------------------------------------

------------------------------------------------------------

------------------------------------------------------------

------------------------------------------------------------

------------------------------------------------------------

------------------------------------------------------------

------------------------------------------------------------

------------------------------------------------------------

------------------------------------------------------------

------------------------------------------------------------

------------------------------------------------------------

------------------------------------------------------------

------------------------------------------------------------

------------------------------------------------------------

------------------------------------------------------------

------------------------------------------------------------

------------------------------------------------------------

------------------------------------------------------------

------------------------------------------------------------

------------------------------------------------------------

------------------------------------------------------------

------------------------------------------------------------

------------------------------------------------------------

------------------------------------------------------------

------------------------------------------------------------

----------------------------------CCTTYTCAACAACCTGGGACGGGGGA

CAGGCTGCTAAACCTAGTAAAAGGAGCCTATCAAGCACTCAACCTCACCAGTCCCGACAG

AACCCAAGAGTGCTGGCTGTGTCTGGTATCGGGACCCCCCTACTACGAAGGGGTTGCCGT

CCT---------------------------------------------------------

------------------------------------------------------------

------------------------------------------------------------

------------------------------------------------------------

------------------------------------------------------------

------------------------------------------------------------

------------------------------------------------------------

------------------------------------------------------------

------------------------------------------------------------

------------------------------------------------------------

------------------------------------------------------------

------------------------------------------------------------

------------------------------------------------------------

------------------------------------------------------------

------------------------------------------------------------

------------------------------------------------------------

------------------------------------------------------------

------------------------------------------------------------

------------------------------------------------------------

------------------------------------------------------------

------------------------------------------------------------

------------------------------------------------------------

------------------------------------------------------------

------------------------------------------------------------

------------------------------------------------------------

-----------------

>4R_Bxv1-4-F_E11_Run_3130_2020-02-03_16-08_2857

------------------------------------------------------------

------------------------------------------------------------

------------------------------------------------------------

------------------------------------------------------------

------------------------------------------------------------

------------------------------------------------------------

------------------------------------------------------------

------------------------------------------------------------

------------------------------------------------------------

------------------------------------------------------------

------------------------------------------------------------

------------------------------------------------------------

------------------------------------------------------------

------------------------------------------------------------

------------------------------------------------------------

------------------------------------------------------------

------------------------------------------------------------

------------------------------------------------------------

------------------------------------------------------------

------------------------------------------------------------

------------------------------------------------------------

------------------------------------------------------------

------------------------------------------------------------

------------------------------------------------------------

------------------------------------------------------------

------------------------------------------------------------

------------------------------------------------------------

------------------------------------------------------------

------------------------------------------------------------

------------------------------------------------------------

------------------------------------------------------------

------------------------------------------------------------

------------------------------------------------------------

------------------------------------------------------------

------------------------------------------------------------

------------------------------------------------------------

------------------------------------------------------------

------------------------------------------------------------

------------------------------------------------------------

------------------------------------------------------------

------------------------------------------------------------

------------------------------------------------------------

------------------------------------------------------------

------------------------------------------------------------

------------------------------------------------------------

------------------------------------------------------------

------------------------------------------------------------

------------------------------------------------------------

------------------------------------------------------------

------------------------------------------------------------

------------------------------------------------------------

------------------------------------------------------------

------------------------------------------------------------

------------------------------------------------------------

------------------------------------------------------------

------------------------------------------------------------

------------------------------------------------------------

------------------------------------------------------------

------------------------------------------------------------

------------------------------------------------------------

------------------------------------------------------------

------------------------------------------------------------

------------------------------------------------------------

------------------------------------------------------------

------------------------------------------------------------

------------------------------------------------------------

------------------------------------------------------------

------------------------------------------------------------

------------------------------------------------------------

------------------------------------------------------------

------------------------------------------------------------

------------------------------------------------------------

------------------------------------------------------------

------------------------------------------------------------

------------------------------------------------------------

------------------------------------------------------------

------------------------------------------------------------

------------------------------------------------------------

------------------------------------------------------------

------------------------------------------------------------

------------------------------------------------------------

------------------------------------------------------------

------------------------------------------------------------

------------------------------------------------------------

------------------------------------------------------------

------------------------------------------------------------

------------------------------------------------------------

------------------------------------------------------------

------------------------------------------------------------

------------------------------------------------------------

------------------------------------------------------------

------------------------------------------------------------

------------------------------------------------------------

------------------------------------------------------------

------------------------------------------------------------

------------------------------------------------------------

------------------------------------------------------------

------------------------------------------------------------

------------------------------------------------------------

------------------------------------------------------------

------------------------------------------------------------

------------------------------------------------------------

------------------------------------------------------------

------------------------------------------------------------

------------------------------------------------------------

------------------------------------------------------------

------------------------------------------------------------

------------------------------------------------------------

------------------------------------------------------------

------------------------------------------------------------

------------------------------------------------------------

------------------------------------------------------------

------------------------------------------------------------

------------------------------------------------------------

------------------------------------------------------------

------------------------------------------------------------

------------------------------------------------------------

----------------------------------GCACTCAACCTCACCAGTCCCGACAG

AACCCAAGAGTGCTGGCTGTGTCTGGTATCGGGACCCCCCTACTACGAAGGGGTTGCCGT

CCTAGGTACCTACTCCAACCATACCTCTGCCCCAGCTAACTGCTCCGTGGCCTCCCAACA

CAAGCTGACCCTGT----------------------------------------------

------------------------------------------------------------

------------------------------------------------------------

------------------------------------------------------------

------------------------------------------------------------

------------------------------------------------------------

------------------------------------------------------------

------------------------------------------------------------

------------------------------------------------------------

------------------------------------------------------------

------------------------------------------------------------

------------------------------------------------------------

------------------------------------------------------------

------------------------------------------------------------

------------------------------------------------------------

------------------------------------------------------------

------------------------------------------------------------

------------------------------------------------------------

------------------------------------------------------------

------------------------------------------------------------

------------------------------------------------------------

------------------------------------------------------------

------------------------------------------------------------

------------------------------------------------------------

-----------------
