# Supplementary material for: Characterization of a new case of XMLV (Bxv1) contamination in the human cell line Hep2 (clone 2B)
Source: Sci Rep. 2020 Sep 29;10:16046. doi: 10.1038/s41598-020-73169-y (PMC7524804; doi:10.1038/s41598-020-73169-y)
Supplement: Supplementary file 5 — Supplementary Information 5. [file 41598_2020_73169_MOESM5_ESM.docx]

**Supplementary information** of article entitled « Characterization of a new case of XMLV (Bxv1) contamination in the human cell line Hep2 (clone 2B) »

By Vincent Loiseau, Richard Cordaux, Isabelle Giraud, Agnès Beby-Defaux, Nicolas Lévêque, Clément Gilbert

This file contains supplementary figure 1 and supplementary tables 1-3.

**Supplementary Figure 1: Bxv1 PCR products visualized on an agarose gel.** Lane 1: ladder; Lane 2: a band of the expected size (400 bp) obtained on the 2018 Hep2 (clone 2B) batch; Lane 3: a band of the expected size (400 bp) obtained on the 2013 Hep2 (clone 2B) batch 400-bp band A band is visualised on a 1.5% agarose gel after PCR on Hep2 DNA; Lane 4: H2O.

**Supplementary Table 1: 23 SNPs between the HPV18 sequence characterized in this study and the HPV18 reference genome (GQ180792).** The SNPs exactly match to the HeLa-specific HPV18 genome identified by Cantalupo et al. (2015).

| Gene | Position | SNP |
| --- | --- | --- |
| E6 | 104 | T 🡪 C |
|  | 287 | C 🡪 G |
|  | 485 | T 🡪 C |
|  | 549 | C 🡪 A |
|  |  |  |
| E7 | 751 | C 🡪 T |
|  | 806 | G 🡪 A |
|  |  |  |
| E1 | 1012 | A 🡪 T |
|  | 1194 | C 🡪 A |
|  | 1353 | T 🡪 A |
|  | 1807 | T 🡪 C |
|  | 1843 | T 🡪 G |
|  | 2269 | C 🡪 T |
|  |  |  |
| L1 | 5875 | C 🡪 A |
|  | 6401 | A 🡪 G |
|  | 6460 | C 🡪 G |
|  | 6625 | C 🡪 G |
|  | 6842 | C 🡪 G |
|  | 7258 | T 🡪 A |
|  | 7486 | C 🡪 T |
|  |  |  |
| LCR | 7529 | C 🡪 A |
|  | 7567 | A 🡪 C |
|  | 7592 | T 🡪 C |
|  | 7670 | A 🡪 T |
|  |  |  |

**Supplementary Table 2: Characteristics of all HeLa – HPV18 junctions**. Most of junctions involve the E1 HPV18 gene and 8q24.21 human chromosome band.

| **Samples with the detected breakpoint** | **Chimeric reads covering the junction** | **Viral breakpoint pos.** | **Involved viral genes** | **Human breakpoint pos.** | **Human chr. Band** | **Involved human genes** |
| --- | --- | --- | --- | --- | --- | --- |
|  |  |  |  |  |  |  |
| RNA & DNA | 273 (272+1, respectively) | 929 | E1 | 128241377 | 8q24.21 | BC106081-exon |
| RNA & DNA | 122 (97+25, respectively) | 5735 | L1 | 128230628 | 8q24.21 | CCAT1-intron |
| RNA | 68 | 22 | / | 128231054 | 8q24.21 | CCAT1-intron |
| RNA & DNA | 58 (57+1, respectively) | 2497 | E1 | 128241551 | 8q24.21 | / |
| RNA | 52 | 929 | E1 | 128241370 | 8q24.21 | BC106081-exon |
| RNA & DNA | 16 (15+1, respectively) | 930 | E1 | 128231213 | 8q24.21 | CCAT1-exon |
| RNA | 10 | 929 | E1 | 128240876 | 8q24.21 | BC106081-exon |
| RNA | 8 | 929 | E1 | 128239788 | 8q24.21 | / |
| RNA | 5 | 929 | E1 | 128221964 | 8q24.21 | CCAT1-intron |
| RNA | 5 | 942 | E1 | 128241377 | 8q24.21 | BC106081-exon |
| RNA | 4 | 1357 | E1 | 128241379 | 8q24.21 | BC106081-exon |
| RNA | 4 | 21 | / | 128231059 | 8q24.21 | CCAT1-intron |
| RNA | 4 | 929 | E1 | 128235913 | 8q24.21 | / |
| RNA | 3 | 414 | E6 | 128231052 | 8q24.21 | CCAT1-intron |
| RNA | 3 | 929 | E1 | 128241374 | 8q24.21 | BC106081-exon |
| RNA | 3 | 931 | E1 | 128241507 | 8q24.21 | / |
| RNA | 2 | 1890 | E1 | 128239463 | 8q24.21 | / |
| RNA | 2 | 2289 | E1 | 128241082 | 8q24.21 | BC106081-exon |
| RNA | 2 | 7454 | / | 128233698 | 8q24.21 | / |
| RNA | 2 | 776 | E7 | 128241375 | 8q24.21 | BC106081-exon |
| RNA | 2 | 903 | E1 | 128241375 | 8q24.21 | BC106081-exon |
| RNA | 2 | 908 | E1 | 128241341 | 8q24.21 | BC106081-exon |
| RNA | 2 | 929 | E1 | 128200362 | 8q24.21 | JX003871-exon, CASC19-intron |
| RNA | 2 | 930 | E1 | 128232653 | 8q24.21 | / |
| RNA | 1 | 120 | E6 | 128241337 | 8q24.21 | BC106081-exon |
| RNA | 1 | 1500 | E1 | 128232770 | 8q24.21 | / |
| RNA | 1 | 1539 | E1 | 98115605 | 12q23.1 | LOC643711-intron |
| RNA | 1 | 1539 | E1 | 68132275 | 15q23 | / |
| RNA | 1 | 1539 | E1 | 180949634 | 3q26.33 | SOX2-OT-intron |
| RNA | 1 | 1552 | E1 | 128241418 | 8q24.21 | / |
| RNA | 1 | 1556 | E1 | 133216516 | 4q28.3 | / |
| RNA | 1 | 1890 | E1 | 128233294 | 8q24.21 | / |
| RNA | 1 | 1987 | E1 | 128241150 | 8q24.21 | BC106081-exon |
| RNA | 1 | 21 | / | 128235782 | 8q24.21 | / |
| RNA | 1 | 2105 | E1 | 128237342 | 8q24.21 | / |
| RNA | 1 | 2254 | E1 | 9934741 | 3p25.3 | JAGN1-exon |
| RNA | 1 | 23 | / | 128231055 | 8q24.21 | CCAT1-exon |
| RNA | 1 | 233 | E6 | 128174331 | 8q24.21 | / |
| RNA | 1 | 557 | E6 | 128239674 | 8q24.21 | / |
| RNA | 1 | 5811 | L1 | 128230765 | 8q24.21 | CCAT1-intron |
| RNA | 1 | 6365 | L1 | 169742576 | 6q27 | / |
| RNA | 1 | 680 | E7 | 128241019 | 8q24.21 | BC106081-exon |
| RNA | 1 | 7702 | / | 893593 | 19p13.3 | / |
| RNA | 1 | 860 | E7 | 70824505 | 12q15 | KCNMB4-exon |
| RNA | 1 | 884 | E7 | 128241206 | 8q24.21 | BC106081-exon |
| RNA | 1 | 926 | E1 | 128241377 | 8q24.21 | BC106081-exon |
| RNA | 1 | 928 | E1 | 128241374 | 8q24.21 | BC106081-exon |
| RNA | 1 | 929 | E1 | 128091380 | 8q24.21 | PCAT2-intron |
| RNA | 1 | 929 | E1 | 128231391 | 8q24.21 | CCAT1-exon |
| RNA | 1 | 930 | E1 | 128181364 | 8q24.21 | / |
| RNA | 1 | 930 | E1 | 128200133 | 8q24.21 | JX003871-intron, CASC19-intron |
| RNA | 1 | 930 | E1 | 128215467 | 8q24.21 | / |
| RNA | 1 | 931 | E1 | 128236722 | 8q24.21 | / |
| RNA | 1 | 931 | E1 | 128241374 | 8q24.21 | BC106081-exon |
| RNA | 1 | 936 | E1 | 128241377 | 8q24.21 | BC106081-exon |
| RNA | 1 | 978 | E1 | 128241348 | 8q24.21 | BC106081-exon |
| RNA | 1 | 979 | E1 | 128233301 | 8q24.21 | / |
| DNA | 1 | 1395 | E1 | 15446806 | 5p15.1 | / |
| DNA | 1 | 6171 | L1 | 92915224 | 6q15 | / |
| DNA | 1 | 6371 | L1 | 129014698 | 9q33.3 | / |
| DNA | 1 | 692 | E7 | 64100489 | 1p31.3 | PGM1-intron |
| DNA | 1 | 7326 | / | 203156088 | 1q32.1 | / |
| DNA | 1 | 741 | E7 | 48589338 | 12q13.11 | / |
| DNA | 1 | 7460 | / | 56118967 | 5q11.2 | MAP3K1-intron |

**Supplementary Table 3. Characteristics of transcripts expressed in the Hep2 (Clone 2B) cells.** Transcripts are ranked by decreasing mean coverage. In total, 50,000 transcripts were found with a mean coverage higher than zero. The table only lists the 50 most covered transcripts (which include Bxv1) as well as APOBEC3G which ranks 37,053.

| **Transcript ID** | **Transcript length (bp)** | **mean coverage** | **Number of reads mapping on the transcript** | **Name of the gene** |
| --- | --- | --- | --- | --- |
| NR_137295 | 1559 | 359858.32 | 4452532 | 16S_rRNA |
| NR_001445 | 332 | 262384.85 | 691363 | 7SK_snRNA |
| NM_001402 | 3512 | 22816.76 | 635971 | EEF1A1 |
| NR_137294 | 954 | 71336.77 | 540121 | 12S_rRNA |
| NM_000584 | 1642 | 39664.38 | 516896 | CXCL8 |
| NR_002715 | 299 | 135469.85 | 321472 | RN7SL1_scRNA |
| NR_027260 | 299 | 122533.48 | 290773 | RN7SL2_scRNA |
| NR_003051 | 277 | 107237.4 | 235752 | RNase_MRP_RNA |
| NR_002312 | 341 | 75880.71 | 205359 | RNase_P_RNA |
| NM_000291 | 4812 | 3557.48 | 135862 | PG_kinase1 |
| NM_000968 | 2741 | 6157.53 | 133950 | RPL4 |
| NR_131012 | 22743 | 738.92 | 133374 | NEAT1_lncRNA |
| NM_000422 | 1517 | 9633.32 | 115982 | Keratin17 |
| NM_001961 | 3158 | 4480.07 | 112286 | EEF2 |
| NM_001006 | 869 | 16226.33 | 111910 | RPS3A |
| NM_000963 | 4510 | 2710.62 | 97022 | NA |
| NM_001010 | 1369 | 8263.22 | 89780 | NA |
| NM_001631 | 3241 | 3411.51 | 87751 | NA |
| NM_021009 | 2193 | 4779.2 | 83180 | NA |
| NR_002819 | 8779 | 1174.39 | 81825 | NA |
| NM_003299 | 2782 | 3704.44 | 81791 | NA |
| NR_144567 | 8545 | 1204.46 | 81683 | NA |
| NM_006098 | 1140 | 8852.17 | 80091 | NA |
| NM_002422 | 1822 | 5520.61 | 79829 | NA |
| NM_001000 | 390 | 25597.94 | 79231 | NA |
| NR_144568 | 8302 | 1201.1 | 79139 | NA |
| NM_002032 | 1203 | 8188.73 | 78182 | NA |
| NR_145459 | 8121 | 1189.91 | 76692 | NA |
| NM_001369451 | 1737 | 5355.97 | 73835 | NA |
| NM_001404 | 1446 | 6321.4 | 72545 | NA |
| NM_005063 | 5245 | 1732.18 | 72105 | NA |
| NM_003380 | 2154 | 4000.5 | 68389 | NA |
| NM_022551 | 549 | 15030.73 | 65491 | NA |
| NM_001354840 | 672 | 11837.18 | 63131 | NA |
| NM_000976 | 634 | 11425.54 | 57490 | NA |
| NM_014220 | 1555 | 4652.11 | 57412 | NA |
| NM_003246 | 7789 | 907.09 | 56073 | NA |
| NM_001007 | 1474 | 4776.16 | 55873 | NA |
| NM_002421 | 1971 | 3516.11 | 55001 | NA |
| NM_006597 | 2264 | 3039.96 | 54622 | NA |
| NM_021109 | 622 | 10805.24 | 53340 | NA |
| NM_001012 | 778 | 8609 | 53157 | NA |
| JF908815 | 8657 | 768.63 | 52809 | Bxv1 |
| NM_000972 | 887 | 7488.24 | 52714 | NA |
| NR_046235 | 13357 | 493.32 | 52296 | NA |
| NR_146117 | 13373 | 490.16 | 52023 | NA |
| NM_001165 | 6877 | 901.57 | 49206 | NA |
| NM_004370 | 11725 | 528.77 | 49204 | NA |
| NR_145819 | 13351 | 459.08 | 48644 | NA |
| NR_146179 | 2145 | 3.49 | 59 | APOBEC3G |
