# Supplementary material for: Characterization of a new case of XMLV (Bxv1) contamination in the human cell line Hep2 (clone 2B)
Source: Sci Rep. 2020 Sep 29;10:16046. doi: 10.1038/s41598-020-73169-y (PMC7524804; doi:10.1038/s41598-020-73169-y)
Supplement: Supplementary file 6 — Supplementary Legends. [file 41598_2020_73169_MOESM6_ESM.docx]

**Legends of supplementary data**

**Supplementary Data 1: Fastq file containing all raw DNA-seq reads aligning on the Bxv1 (JF908815) genome.**

**Supplementary Data 2:** **Fastq file containing all raw RNA-seq reads aligning on the Bxv1 (JF908815) genome.**

**Supplementary Data 3: Alignment of DNA-seq reads supporting the Bxv1 proviral locus characterized in this study.** Aligned sequences are numbered from 1 to 6. 1: the sequence of the human genome flanking the Bxv1 proviral locus, comprising 100 bp upstream and downstream of the provirus, located in the second intron of the *PUS1* gene. 2: full length Bxv1 genome sequenced from the VCaP prostate cancer cell line. 3 – 5: three reads supporting the virus-cell 5’ junction. 6: Read supporting the virus-cell 3’ junction. Note that the 3’ junction is also supported by one RNA-seq read. The integration generated a 5-bp target site duplication (AAACC). The alignment is provided in docx format. It can be pasted and visualized in any alignment viewer such as BioEdit or Geneious.

**Supplementary Data 4: Alignment of Bxv1 (JF908815) with Sanger-sequencing reads produced during this study.** The name of each read begins with a number corresponding to the region illustrated on Figure 2A. F: forward read. R: reverse read. The name of the read also contains the name of the primers used to PCR-amplify the four Bxv1 regions.
